# Systematic review of hematophagous arthropods present in cattle in France[Fn FN1]

**DOI:** 10.1051/parasite/2023059

**Published:** 2023-12-12

**Authors:** Jorian Prudhomme, Jérôme Depaquit, Johanna Fite, Elsa Quillery, Emilie Bouhsira, Emmanuel Liénard

**Affiliations:** 1 InTheres, University of Toulouse, INRAE, ENVT 31300 Toulouse France; 2 Université de Reims Champagne-Ardenne, Faculté de Pharmacie, EA7510 EpidémioSurveillance et Circulation de Parasites dans les Environnements, and ANSES, USC Pathogènes-Environnement-Toxoplasme-Arthropodes-Réservoirs-bioDiversité Reims France; 3 Centre Hospitalo-Universitaire, Laboratoire de Parasitologie-Mycologie 51092 Reims France; 4 French Agency for Food, Environmental and Occupational Health & Safety, Risk Assessment Department Maisons-Alfort Cedex France

**Keywords:** Systematic review, PRISMA, Hematophagous, Arthropods, Cattle

## Abstract

The arrival of pathogens, whether zoonotic or not, can have a lasting effect on commercial livestock farms, with dramatic health, social and economic consequences. However, available data concerning the arthropod vectors present and circulating on livestock farms in France are still very imprecise, fragmentary, and scattered. In this context, we conducted a systematic review of the hematophagous arthropod species recorded on different types of cattle farms in mainland France (including Corsica). The used vector “groups” studied were biting flies, biting midges, black flies, fleas, horse flies, lice, louse flies, mosquitoes, sand flies, and ticks. A large number of documents were selected (*N* = 9,225), read (*N* = 1,047) and analyzed (*N* = 290), allowing us to provide distribution and abundance maps of different species of medical and veterinary interest according to literature data. Despite the large number of documents collected and analyzed, there are few data provided on cattle farm characteristics. Moreover, data on all arthropod groups lack numerical detail and are based on limited data in time and/or space. Therefore, they are not generalizable nor comparable. There is still little information on many vectors (and their pathogens) and still many unknowns for most studied groups. It appears necessary to provide new, updated and standardized data, collected in different geographical and climatological areas. Finally, this work highlights the lack of entomologists, funding, training and government support, leading to an increased risk of uncontrolled disease emergence in cattle herds.

## Abstract (fr)

L’arrivée d’agents pathogènes, même non zoonotiques, peut affecter durablement les élevages commerciaux, avec des conséquences sanitaires, sociales et économiques dramatiques. Cependant, les données disponibles concernant les arthropodes vecteurs présents et circulants dans les élevages français sont encore très imprécises, fragmentaires et dispersées. Dans ce contexte, nous avons réalisé une revue systématique des espèces d’arthropodes hématophages présentes dans différents types d’élevages bovins en France métropolitaine (Corse comprise). Les “groupes” de vecteurs retenus et étudiés étaient les mouches piqueuses, les culicoïdes, les simulies, les puces, les taons, les poux, les hippobosques, les moustiques, les phlébotomes et les tiques. Un grand nombre de documents ont été sélectionnés (*N* = 9 225), lus (*N* = 1 047) et analysés (*N* = 290), ce qui nous a permis de fournir des cartes de distribution et d’abondance des différentes espèces d’intérêt médical et vétérinaire en fonction des données de la littérature. Malgré le grand nombre de documents collectés et analysés, peu de données sont fournies sur les caractéristiques des élevages bovins. En outre, les données sur tous les groupes d’arthropodes manquent de détails numériques et sont basées sur des informations limitées dans le temps et/ou l’espace. Elles ne sont donc ni généralisables ni comparables. Il existe encore peu d’informations sur de nombreux vecteurs (et leurs agents pathogènes) et de multiples inconnues subsistent pour les groupes les plus étudiés. Il apparaît nécessaire de collecter de nouvelles données, actualisées et standardisées dans différentes zones géographiques et climatiques. Enfin, ce travail met en évidence le manque d’entomologistes, de financements, de formations et de soutien gouvernemental, ce qui accroît le risque d’émergence non contrôlée de maladies dans les troupeaux de bovins.

## 1 Introduction

Global changes observed for several decades, such as the intensification of international trade, agricultural encroachment on natural systems, and climate change, facilitate the spread, emergence, or re-emergence of diseases affecting human, animal, and plant health on a global scale. Many pathogens are therefore likely to appear at the borders of the European Union and of France each year, but few of them manage to establish themselves durably, according to the “10% rule” developed by Williamson and Fitter [104]. However, the health, social, and economic consequences can be dramatic.

The arrival of pathogens, even when they are not zoonotic, can have a lasting effect on commercial livestock farms. The sudden arrival of bluetongue in France in 2006 is a typical illustration. This virus, which affects sheep and cattle farms in particular, has spread very rapidly in France due to its vectorial mode of transmission and has become enzootic [61]. There are other risks of introduction of potentially vector-borne pathogens into France, such as African swine fever virus [88] or *Trypanosoma evansi* [24]. These examples show that pathogens may become established long-term in France, because the proven or potential vectors are already present.

However, available data concerning the arthropod vectors present and circulating on livestock farms are still very imprecise, fragmentary, and scattered. In order to predict the evolution of any vector-borne disease and to control its spread, it is essential to identify and characterize the potentially involved vectors. These data are crucial, particularly for cattle breeding in France, because of its economic and social importance at the national and international levels. According to Food and Agriculture Organization (FAO) data, France has the largest suckling and dairy cattle population in the European Union [38]. Vector control therefore has a dual objective: to limit vector distribution ranges, and to prevent the transmission of pathogens to animals (and to humans for those that are zoonotic), as well as the associated production losses and economic costs.

In this context, we conducted a systematic review in order to carry out a bibliographic inventory of the hematophagous arthropods species present in different types of cattle farms in mainland France (including Corsica), and to estimate the distribution and abundance of different species of medical and veterinary interest according to the data reported in the literature. The objectives of this study were to provide a bibliographic inventory including works on hematophagous arthropods present on cattle farms in France, and to identify the parameters and breeding practices that are favorable to the presence of these arthropods, as well as possible bibliographic gaps.

## 2 Methods

### 2.1 Research question

A Population and Outcome (PO) statement (Table 1) was developed to answer the following question: Which are the species of hematophagous arthropods present in different types of cattle farms in mainland France?

**Table 1:** Definition of PO statement.

	Definition
Population	Hematophagous arthropods present on different types of cattle farms in mainland France
Outcomes	Entomological indicators

A systematic review was conducted by following the reporting checklist of the Preferred Reporting Items for Systematic Reviews and Meta-Analyses (PRISMA) [79, 80]. A protocol was developed in advance detailing the method of analysis and the inclusion criteria.

### 2.2 Search strategy

We conducted a systematic electronic search of the literature, with no restriction on language, using PubMed, Web of Science, Scopus and CAB direct databases. Databases were selected from the 14 recommended as well-suited for systematic reviews described in Gusenbauer and Haddaway [48]. For the choice of these databases, special attention was paid to the following criteria: subject, Boolean and parenthesis functional, number of accessible documents, and bulk download.

The following search terms were used to identify references relevant to the research question: (France OR Corsica OR French) AND (cattle OR livestock OR bovine OR cow OR beef OR calf OR calves OR heifer) AND (haematophag* OR hematophag* OR vector* OR arthropod* OR insect* OR tick* OR mite* OR acari*). The last search was performed on March 28, 2022. The bibliographies of identified references were also searched and references of interest were added and analyzed following the same process as described below.

### 2.3 Screening process and study selection

First, a comprehensive literature search was performed on PubMed, Web of Science, Scopus and CAB direct databases. All articles were exported for analysis in CADIMA [60], an online open access tool designed for conducting systematic reviews. After removing duplicates, an initial review of the title and abstract, or only the title when the abstract was not available, was performed. Each publication was reviewed by a single person (JP) against inclusion criteria defined to identify documents containing information on hematophagous arthropods on cattle in France, regardless of the year of publication. Exclusion criteria included: (a) publication not concerning the French territory; (b) studies on animals other than cattle; (c) research not based on blood-feeding arthropods; or (d) publications in a language other than English or French. During this check, if the first criterion was negative, the others were also defined as negative, and so on for all criteria in the following order: a, b, and c. In order to obtain as much information as possible, if a criterion was considered uncertain, *i.e.*, if the title or abstract did not allow us to decide with certainty on the presence of the inclusion criterion, the criterion was declared positive.

If books or whole journal issues were added at this stage, each chapter or article was reviewed individually as described above (title and abstract, then full reading). Documents selected for full reading were then collected (and sometimes excluded if not available). The collection was carried out: (1) by using the above-mentioned databases, (2) by contacting the authors or journals, (3) by scanning the documents in the archives of Toulouse university libraries, and (4) through interlibrary loans (mainly books and theses). This process was achieved on February 3rd, 2023.

Secondly, each publication selected was reviewed in full text for inclusion or exclusion according to the following eligibility criteria: publication including information (a) from France or Corsica; (b) on cattle; (c) on hematophagous arthropods; (d) in English or French, and (e) with entomological indicators (presence or abundance). Finally, another round of review was performed, as a quality control measure, on the excluded articles.

### 2.4 Data extraction and analysis

A qualitative analysis was conducted on the included studies to account for the wide variety of publication styles and research methods presented. From the included studies, data were extracted to determine the taxa of hematophagous arthropods, their spatio-temporal distribution, their presence or abundance (if available), their direct and/or indirect roles in pathogen transmission, the trapping methods used, the study duration, the trapping period, the latitude, the altitude, and the factors allowing the presence and maintenance of arthropod populations on cattle farms according to their ecology and the type of production (*e.g.*, organic, conventional, mixed, *etc.*) and breeding (*e.g.*, milk, meat or mixed).

Data extraction and analysis were carried out by a single person (JP) after validation of the document selection process and data extraction methods by all project partners (JP, JF, EQ, JD, EB and EL).

The distribution maps of the different hematophagous arthropods species were produced using the website of the Institut National de l’Information Géographique et Forestière (IGN) [54].

### 2.5 Study limitations

Due to the large number of documents selected (*N* = 9,225), read (*N* = 1,047), and analyzed (*N* = 290), the grey literature was not collected. Although the bibliography study of the identified references limited the number of documents that escaped the databases used, it is possible that some documents were not identified in our systematic review. However, the most exhaustive bibliographic research possible was carried out and provides a good overview of current knowledge on the various hematophagous arthropods present on cattle farms in France. This work identifies the gaps in knowledge and the aspects that can be quantitatively evaluated.

The strategy to obtain data was developed and discussed between the project partners and the research questions and analysis methodology were validated by all partners. However, only one person (JP) evaluated and analyzed all the references, which may lead to subjectivity bias as consensus between two independent evaluators was not possible [14].

## 3 Results

### 3.1 Bibliometric data

The full search resulted in 9,225 documents (8,955 publications in initial search, 258 by publications bibliographies check, and 12 from experts). A total of 1,781 duplicate documents were removed. Then, 7,174 documents were reviewed, 6,761 on title and abstract, and 413 on title (abstract not available). Amongst these documents, 6,322 were excluded using the criteria described above, (a) the publication was not carried out in France (*n* = 3,763); (b) research carried out on an animal other than bovines (*n* = 1,381); or (c) the study was not based on hematophagous arthropods (*n* = 1,178).

**Figure 1:**
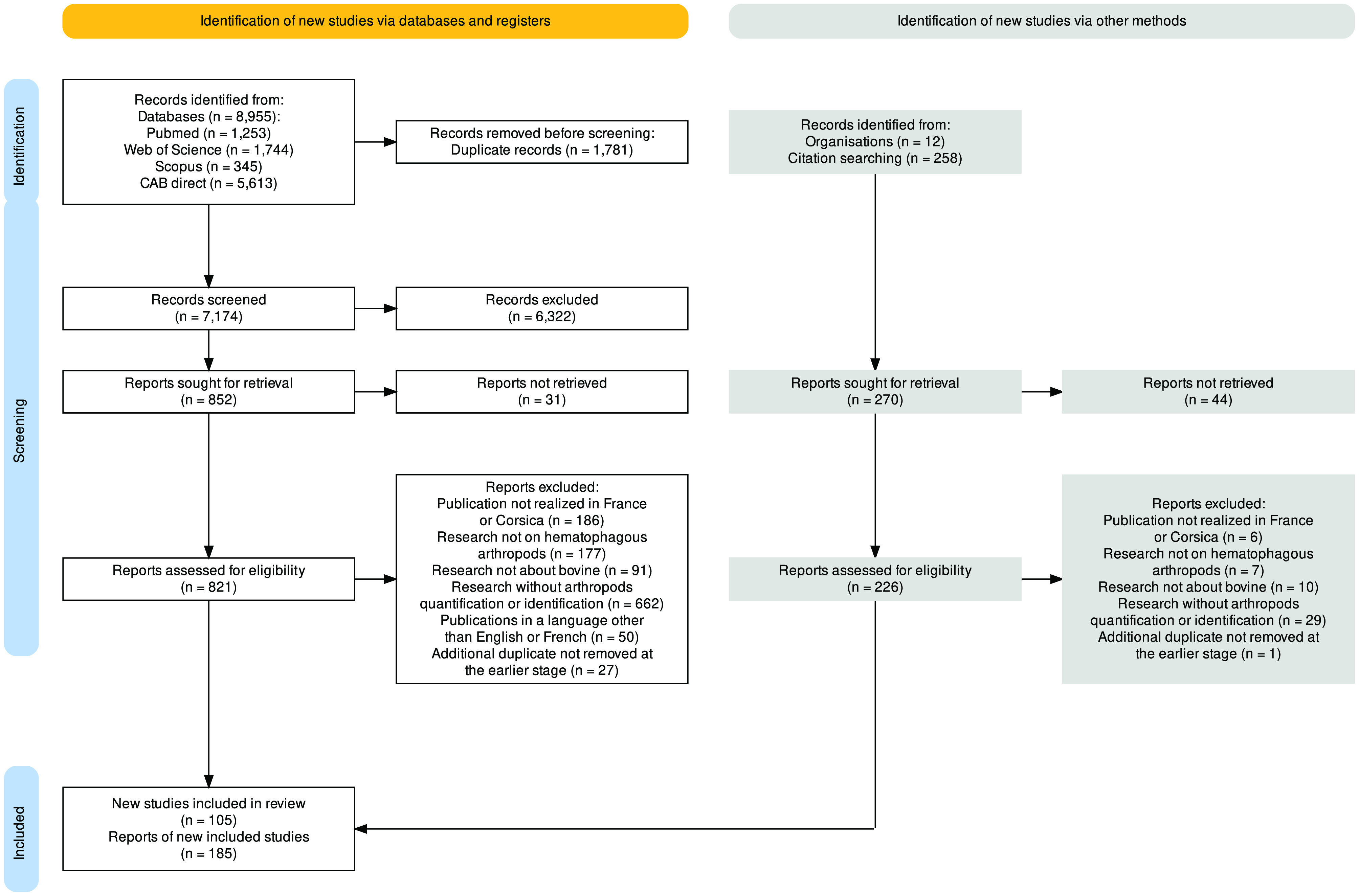
PRISMA flow chart of the study selection process.

Of the 1,122 reports sought for retrieval, 16 records were recovered through the authors and 75 were not accessible through institutional library channels. A total of 1,047 articles were read in their entirety, if written in English or French. Then, 761 full-text articles were excluded for (a) publication not concerning mainland France or Corsica (*n* = 192); (b) research not on hematophagous arthropods (*n* = 184); (c) about animals other than bovines (*n* = 101); (d) without arthropod quantification or identification (*n* = 691); (e) publications in a language other than English or French (*n* = 50); or (f) additional duplicate not removed at the earlier stage (*n* = 28). A total of 290 titles were incorporated into the final study results. The PRISMA flow diagram of the screening process is available in Figure 1 and the full list of documents in Supplementary Table 1.

**Figure 2:**
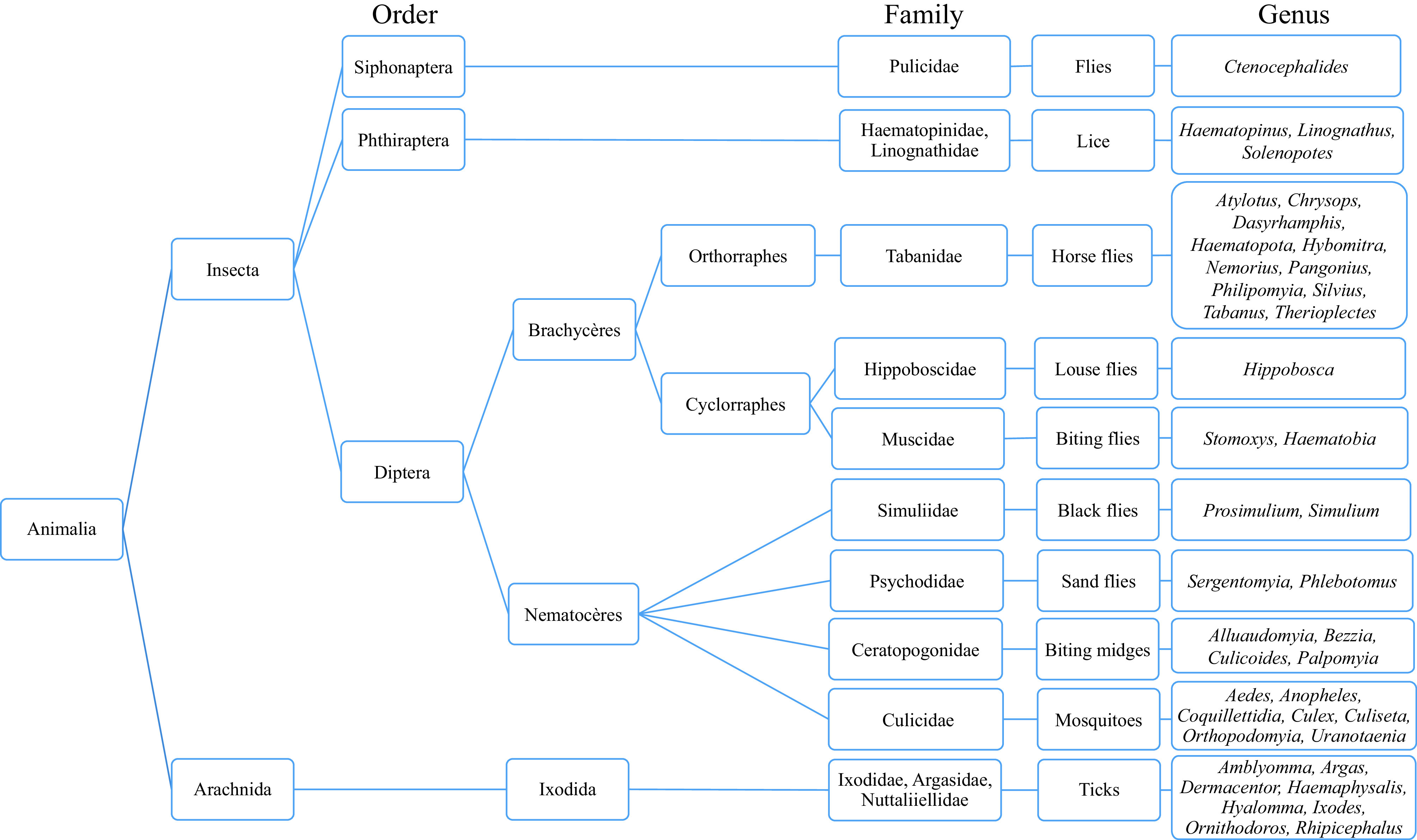
Simplified systematic classification of the different arthropod groups studied.

**Figure 3:**
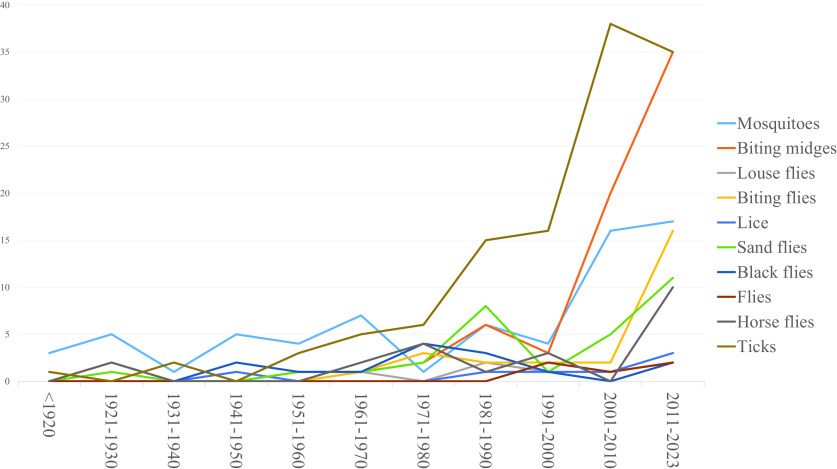
Characteristics of included studies based on their publication dates by arthropod groups.

Of these 290 documents (including books, theses, and journal issues), 331 insect capture description reports were extracted and analyzed. Different taxonomic levels were used for the definition of the different vector groups and their analysis according to the number of species, the accuracy and the availability of the collected data (order, family, genus, and species). Thus, the vector “groups” used were fleas (= *Ctenocephalides felis*), lice (= Phthiraptera), louse flies (= *Hippobosca equina*), black flies (= Simuliidae), horse flies (= Tabanidae), biting flies (= Muscidae), sand flies (= Psychodidae), mosquitoes (= Culicidae), biting midges (= Ceratopogonidae), and ticks (= Ixodida). The simplified systematic classification of the different groups studied is summarized in Figure 2. The characteristics of included studies examining hematophagous arthropods are detailed in Table 2 and Figure 3.

**Table 2:** Characteristics of included studies based on their publication dates.

Year	<1920	1921–1930	1931–1940	1941–1950	1951–1960	1961–1970	1971–1980	1981–1990	1991–2000	2001–2010	2011–2023	Total (%)
Number of extracted reports*	4	9	3	7	10	14	22	39	33	78	112	331
Fleas	0	0	0	0	0	0	0	0	2	1	2	5 (1.5)
Lice	0	0	0	1	0	0	0	1	1	1	3	7 (2.1)
Louse flies	0	2	0	0	1	1	0	2	1	1	2	10 (3)
Black flies	0	0	0	2	1	1	4	3	1	0	2	14 (4.2)
Horse flies	0	2	0	0	0	2	4	1	3	0	10	22 (6.6)
Biting flies	0	2	0	0	0	1	3	2	2	2	16	28 (8.5)
Sand flies	0	1	0	0	1	1	2	8	1	5	11	30 (9.1)
Mosquitoes	3	5	1	5	4	7	1	6	4	16	17	69 (20.8)
Biting midges	0	1	0	0	1	2	2	6	3	20	35	70 (21.1)
Ticks	1	0	2	0	3	5	6	15	16	38	35	121 (36.6)

* Some documents provide information on several arthropod groups.

### 3.2 Cattle farm data

**Figure 4:**
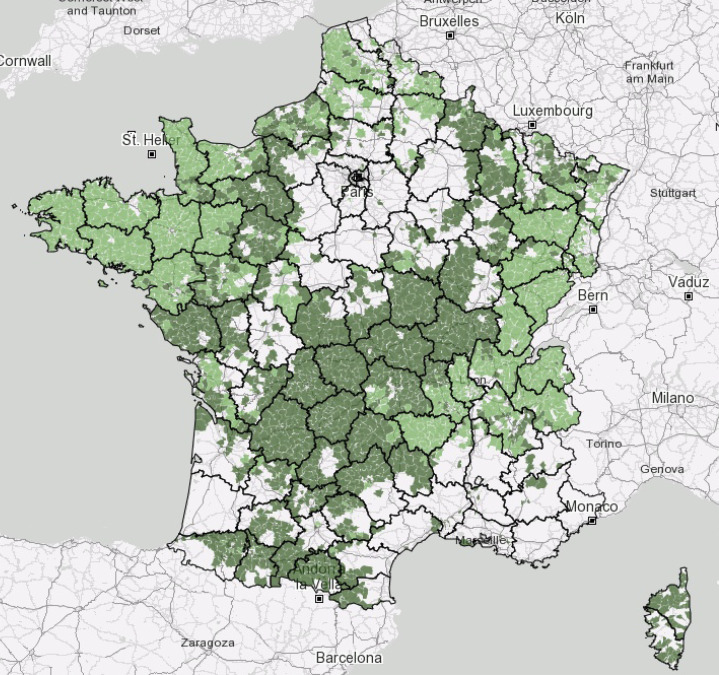
Distribution of cattle farms in France, with dairy farms in light green and other cattle and buffalo farms in dark green [34].

France has the largest cattle population in the European Union and is Europe’s leading meat and milk producer [38]. In 2020, France had more than 17 million cattle [33], with cattle farms present across almost the whole country (Fig. 4) [34]. The data search about the cattle farms in the systematic review covered: cattle breeds, livestock size, production (milk, meat or mixed) and farming types (organic, conventional, mixed, *etc.*). Unfortunately, it was not possible to collect these data. Indeed, 91.5% of the references did not provide information on production type (milk, meat or mixed) and 99.1% on breeding type (conventional or mixed) (Table 3). The cattle breeds identified were: Abondance, Vosgienne, Tarentaise, Brune des Alpes, Charolaise, Limousine, Montbéliarde, Normande, Rouge Flamande, Salers, Gasconne, Holstein-Friesian, Hollandaise, Schwytz, Aubrac, Prim’hostein (= Frisonne Pie-Noir), Blonde d’Aquitaine and Bleue du Nord. However, 92.1% of the references did not provide the breed (Table 3). Similarly, 85.2% of the references did not indicate the size of the herd, and of the few studies providing this data, it is often unsourced, variable (*e.g.*, between 12 cows on a farm and 3,094 individuals in the department), and based on different measures (number on the farm (*N* = 35; 71.4%), average number per farm (*N* = 10; 20.4%), number per m^2^ (*N* = 2; 4.1%), number per hectare of grass (*N* = 1; 2%), number in France (*N* = 1; 2%)) (Table 3). The absence or disparity of these data made it impossible to determine whether these factors statistically influence the presence and/or abundance of hematophagous arthropods.

**Table 3:** Summary of data searched and obtained on cattle farms.

Data		Number of documents	Percentage (%)
Production type	Milk	14	4.2
	Meat	3	0.9
	Mixed	11	3.3
	Data not provided	303	91.5
Breeding type	Conventional	1	0.3
	Mixed	2	0.6
	Data not provided	328	99.1
Cattle breed	Abondance	1	1.6
	Vosgienne	2	3.2
	Tarentaise	1	1.6
	Brune des Alpes	1	1.6
	Charolaise	1	1.6
	Limousine	7	11.1
	Montbéliarde	1	1.6
	Normande	2	3.2
	Rouge Flamande	1	1.6
	Salers	4	6.3
	Gasconne	8	12.7
	Holstein-Friesian	9	14.3
	Hollandaise	9	14.3
	Schwytz	10	15.9
	Aubrac	2	3.2
	Prim’hostein(= Frisonne Pie-Noir)	1	1.6
	Blonde d’Aquitaine	2	3.2
	Bleue du Nord	1	1.6
	Data not provided	305	92.1
Livestock size	Number on the farm	35	71.4
	Number in France	1	2
	Number per m^2^	2	4.1
	Average number per farm	10	20.4
	Number per hectare of grass	1	2
	Data not provided	282	85.2

### 3.3 Hematophagous arthropods data

Cattle-feeding hematophagous arthropods belong to eight “groups”, and 13 families: louse flies (Hippoboscidae), fleas (Pulicidae), lice (Haematopinidae, Lignognathidae and Nuttalliellidae), ticks (Ixodidae and Argasidae), mosquitoes (Culicidae), biting midges (Ceratopogonidae), biting flies (Muscidae), sand flies (Psychodidae, Phlebotominae), black flies (Simuliidae), and horse flies (Tabanidae) (Fig. 2). The first four orders are obligatorily host-dependent for the duration of their evolutionary cycle, while the following orders are only temporary or occasional parasites.

Despite the large number of documents retrieved and analyzed, there are only few data on the presence/abundance of cattle-feeding hematophagous arthropods (Table 2, Fig. 3). In addition, most groups (louse flies, fleas, lice, biting flies, sand flies, black flies, and horse flies) are poorly documented (< 30 references), with old sources (most often before 1980), lacking in detail numbers, and based on limited data.

The different species distributions in France by department are described in Suppl. Fig. 1 and Suppl. Tab. 2 for fleas (S1), lice (S2), horse flies (S3), louse flies (S4), biting flies (S5), black flies (S6), sand flies (S7), mosquitoes (S8), biting midges (S9), and ticks (S10).

### 3.4 Insecta, Siphonaptera: Fleas

*Bio-ecology*. Fleas are small (0.8–9 mm), apterous insects, bilaterally compressed, ectoparasites in the adult stage, hematophagous and mainly parasites of mammals and birds. In France, our systematic review has highlighted only one species identified and reported on cattle: *Ctenocephalides felis*. This species is cosmopolitan and the main species found on domestic carnivores. Sporadic infestations may be described in production animals, especially cattle, generally in association with the presence of parasitized farm cats. This species is present across the whole country (Suppl. Fig. 1 and Suppl. Tab. 2).

**Figure 5:**
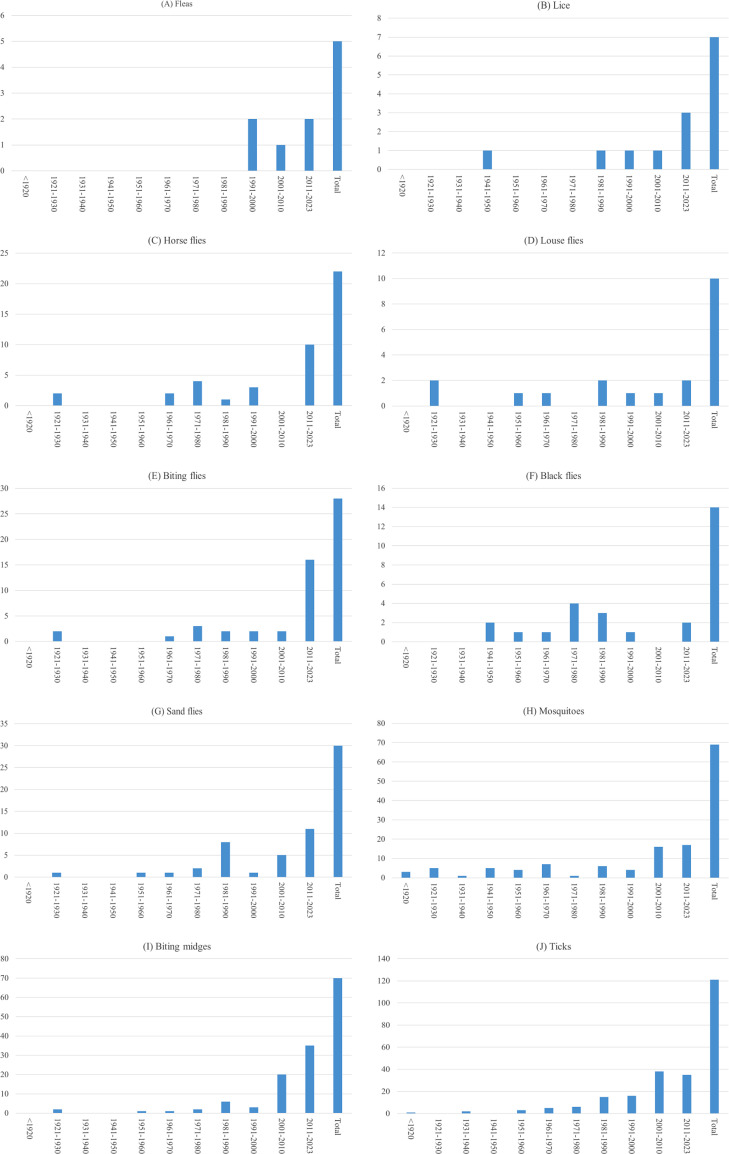
Number of studies carried out on cattle farms, according to their publication dates, for fleas (A), lice (B), horse flies (C), louse flies (D), biting flies (E), black flies (F), sand flies (G), mosquitoes (H), biting midges (I), and ticks (J).

*Bibliometric data*. Only 5 documents were collected and analyzed describing the presence of fleas on cattle farms. All documents were published from the year 1991 onwards (Fig. 5A).

*Trapping methods*. When the trapping method is described (*N* = 2/5; 40%), it is generally direct sampling on the fur with a comb (Suppl. Tab. 2). The abundance of fleas on cattle farms is never described.

*Veterinary importance*. Fleas directly affect their hosts by feeding on blood. Heavily infested animals may become emaciated, anemic, and susceptible to secondary infections, especially younger animals. However, no vectorial role has been demonstrated in cattle.

### 3.5 Insecta, Phthiraptera: Lice

*Bio-ecology*. Lice are small insects (0.35–10 mm for adults), apterous with a dorso-ventrally flattened body. Host specificity varies according to species. Indeed, some species have a high host specificity (*e.g.*, *Haematomyzus elephantis* infecting only elephants), while others have a less strict specificity (*e.g.*, *Linognathus africanus* infecting goats, sheep, and deer) [73]. Lice can be shared into two groups: mallophagous and haematophagous (= true lice). The Mallophaga includes species with crusher-type mouthpieces and will therefore not be discussed here. The second group includes, according to the literature, the three species found in France and parasiting cattle: *Haematopinus eurysternus*, *Linognathus vituli* and *Solenopotes capillatus*. These species have a wide distribution linked to their hosts and are present across the whole country (Suppl. Fig. 1 and Suppl. Tab. 2).

*Bibliometric data*. Only 7 documents could be recovered and analyzed. The documents were mostly published from the 1980s onwards (Fig. 5B). All publications present data with entomological indicators (*e.g.*, species, presence, and abundance). However, the number of references, on cattle farms, for this group remains very low (*N* = 7).

*Trapping methods*. The method of capture is often described (*N* = 5/7; 71%). Lice are usually collected with a comb or directly with the fingers from the coat of the infested host (Suppl. Tab. 2). Abundance is described in 57% of references (*N* = 4/7). However, abundance data are variable between references, expressed as (1) percentage of parasitized cows (9.7–18.5%, 4.6–8%, 3% for *H. eurysternus*, *L. vituli*, *S. capillatus*, respectively [22, 27]); (2) percentage of farms with parasitized animals (45%, 25%, 20% for *H. eurysternus*, *L. vituli*, *S. capillatus*, respectively [25]) and; (3) average number of individuals per cow (14.5 and 39.6 for *H. eurysternus* and *L. vituli* respectively [72]). These data are very localized (Corrèze, Rhône and Saône-et-Loire). Therefore, it is not possible to generalize to the whole country.

*Veterinary importance*. Parasite loads can be high (> 1,000 lice by cattle) [43], particularly in young, old, sick or stressed animals. The presence of these hematophagous insects can have an economic impact due to their negative influence on cattle growth and milk production [43]. However, no vectorial role has been demonstrated in cattle.

### 3.6 Insecta, Diptera, Brachycera, Orthorrhapha, Tabanidae: Horse flies

*Bio-ecology*. Horse flies are stocky and medium to large in size (6–30 mm). Their head is as wide as their thorax. Males have large, touching eyes, while females have smaller, separate eyes. Tabanidae include more than 4,400 species and subspecies spread all over the world [6]. However, their biology and behavior are still poorly understood. Their rearing in the laboratory is difficult because of their long developmental period, particularly the larval stages which can last several months to several years, the diversity of environments colonized by these stages and their diet which varies according to the species (*e.g.*, cannibalism, predation of other arthropod species, *etc.*). At this time and to our knowledge, no permanent colony has been successfully established.

In our systematic review, 83 species have been recorded in France on cattle farms: *Atylotus agrestis, Atylotus flavoguttatus, Atylotus fulvus, Atylotus intermedius, Atylotus latistriatus, Atylotus loewianus, Atylotus plebeius, Atylotus quadrifarius, Atylotus rusticus, Chrysops caecutiens, Chrysops flavipes, Chrysops italicus, Chrysops parallelogrammus, Chrysops pictus, Chrysops relictus, Chrysops rufipes, Chrysops sepulcralis, Chrysops viduatus, Dasyrhamphis anthracinus, Dasyrhamphis ater, Haematopota bigoti, Haematopota crassicornis, Haematopota grandis, Haematopota italica, Haematopota lambi, Haematopota ocelligera, Haematopota pluvialis, Haematopota scutellata, Heptatoma pellucens, Hybomitra acuminata, Hybomitra aterrima, Hybomitra auripila, Hybomitra bimaculata, Hybomitra borealis, Hybomitra caucasica, Hybomitra ciureai, Hybomitra distinguenda, Hybomitra erberi, Hybomitra expollicata, Hybomitra lundbecki, Hybomitra lurida, Hybomitra micans, Hybomitra montana, Hybomitra muhlfeldi, Hybomitra olsufievina, Hybomitra solstitialis, Hybomitra tropica, Hybomitra vittata, Nemorius vitripennis, Pangonius haustellatus, Pangonius micans, Philipomyia aprica, Philipomyia graeca, Silvius algirus, Silvius alpinus, Silvius variegatus, Tabanus autumnalis, Tabanus bifarius, Tabanus bovinus, Tabanus briani, Tabanus bromius, Tabanus cordiger, Tabanus darimonti, Tabanus eggeri, Tabanus exclusus, Tabanus glaucopis, Tabanus lateralis, Tabanus lunatus, Tabanus maculicornis, Tabanus miki, Tabanus nemoralis, Tabanus paradoxus, Tabanus quatuornotatus, Tabanus rectus, Tabanus regularis, Tabanus rupium, Tabanus spectabilis, Tabanus spodopterus, Tabanus sudeticus, Tabanus tergestinus, Tabanus tinctus, Tabanus unifasciatus*, and *Therioplectes gigas*. The different species distributions in France by department are described in Suppl. Fig. 1 and Suppl. Tab. 2.

Female horse flies have an opportunistic feeding behavior with a preference for large mammals [5, 7, 31]. However, they can also have a highly varied host range (*e.g.* dogs, rabbits, wild rodents, lizards, turtles, birds, and humans) [5, 31].

*Bibliometric data*. The number of documents retrieved and analyzed for horse flies is slightly higher (*N* = 22) than for previous groups. However, apart from some species (*e.g.*, *T. bromius*, *T. sudeticus*), there are limited data on horse fly presence/abundance in France. Moreover, for the most frequently described species, data are highly variable, for example between 26 and 6,270 and 1 and 38 individuals captured, depending on the study, for *T. bromius* and *T. sudeticus*, respectively. On average, from 1960s, three references are published by decade (Fig. 5C).

*Trapping methods*. The capture method is described in 59.1% of the documents (*N* = 13/22). Numerous techniques are reported: manual capture (larvae and adults) and the use of traps to capture adults. The main traps used are Vavoua, N’zi, Malaise or Manitoba traps (with or without odorous bait) (Suppl. Tab. 2). Abundance is rarely described (*N* = 6/22; 27.2%), highly variable and preferably expressed as the number of individuals captured. However, these results are noticeably localized (Bouches-du-Rhône, Hautes-Alpes, Ille-et-Vilaine, Loire-Atlantique, Maine-et-Loire, and Pyrénées-Orientales) and therefore cannot be generalized to the whole of France, particularly as the presence of adults can be occasional (a few days to a few weeks).

*Veterinary importance*. In most temperate zones, adult horse flies are a nuisance to cattle because of their painful bites and aggressiveness [8]. They can mechanically transmit many and various pathogens through contaminated blood on their large mouthparts [6] (Suppl. Tab. 2).

### 3.7 Insecta, Diptera, Brachycera, Cyclorrhapha, Hippoboscidae: Louse flies

*Bio-ecology*. Adult size ranges from 1.5 to 12 mm, with a dorso-ventrally flattened, robust and relatively hairless body. Both genders are hematophagous and parasite birds and mammals. Host specificity varies considerably between species. Some are restricted to a single host species (*e.g.*, *Pseudolynchia canariensis* parasiting pigeons), while others have a wide host range (*e.g.*, *Hippobosca longipennis* infecting dogs, foxes, mongooses, hyenas, and cats) [73]. In France, on cattle, only one species was identified in the systematic review: *Hi. equina*. This species, widespread in the Old World, is a parasite of equids but can also infest cattle and is present across the whole country (Suppl. Fig. 1 and Suppl. Tab. 2).

*Bibliometric data*. A total of 10 documents were retrieved and analyzed; there are limited data on the presence/abundance of louse flies in cattle farms. On average, one reference is published by decade (Fig. 5D). The number of publications is very low, suggesting that this insect is probably not a particular problem in cattle farms in France.

*Trapping methods*. When the trapping method is described (*N* = 5/10; 50%), collection was carried out directly on the host (*e.g.*, hand net) (Suppl. Tab. 2). Abundance is rarely described (*N* = 1/10; 10%), averaging 5.9 individuals per cow in a study conducted in Saône-et-Loire [72]. This result can obviously not be generalized to the whole territory.

*Veterinary importance*. Heavily infested animals can become anemic and more susceptible to secondary infections. No vectorial role was found in cattle in the systematic review.

### 3.8 Insecta, Diptera, Brachycera, Cyclorrhapha, Muscidae: Biting flies

*Bio-ecology*. Adult biting flies are 4 to 12 mm long with wings extending posteriorly beyond the abdomen. Even if they look like common flies, unlike them, these species have a biting mouthparts (proboscis) non-retractable. The Muscidae are divided into two subfamilies: Fanniinae and Muscinae. The Fanniinae includes only non-biting flies and will therefore not be discussed here. The second includes non-biting species and 3 biting species of interest in France: *Haematobia irritans*, *Haematobia stimulans*, and *Stomoxys calcitrans*. For these 3 species, both sexes are hematophagous at the adult stage, the larvae and pupae can be found in a large collection of organic substrates. These 3 species are present throughout France (Suppl. Fig. 1 and Suppl. Tab. 2).

*Bibliometric data*. A total of 28 documents were collected and analyzed. Although biting flies are widespread throughout the country, few data on their respective abundances on cattle farms are available. On average, from the 1960s onwards, 4 references are published per decade (Fig. 5E).

*Trapping methods*. The collection method is described in 53.6% of the documents (*N* = 15/28). Many methods can be used: manual capture (*e.g.*, hand net), adult traps (Vavoua, Malaise, CDC, N’zi, emergence trap), aspirator, attractive sticky trap and screen (*e.g.*, TDV, impregnated with deltamethrin, blue polyethylene) (Suppl. Tab. 2). Abundance is poorly described (*N* = 11/28; 39.3%); highly variable and expressed in number of larvae, in total number of individuals captured, in individual numbers per trap, per day or per year. The wide disparity of results does not allow for an evaluation and comparison of abundances between capture sites.

*Veterinary importance*. Biting flies are responsible for direct nuisance (*e.g.*, painful bites, stress, *etc.*). However, their contribution to economic losses, although significant, is poorly documented in France. No vectorial role was described in the systematic review for the species *Ha. irritans* and *Ha. stimulans* (Suppl. Tab. 2). The species *S. calcitrans* is known as a mechanical or biological vector of numerous pathogens (protozoa, viruses, bacteria) such as: *Besnoitia besnoiti* (currently in expansion in France and Europe [1]), *Dermatophilus congolensis*, *Anaplasma marginale*, *Bacillus anthracis*, *Trypanosoma evansi*, bovine viral diarrhea virus, lumpy skin disease virus, Rift Valley fever virus, bovine leukosis virus, and West Nile virus (Suppl. Tab. 2).

### 3.9 Insecta, Diptera, Nematocera, Simuliidae: Black flies

*Bio-ecology*. Black flies are small (1–5 mm), robust insects, with different color according to the species and a characteristic humped profile. The immature stages are aquatic and filter feeders. After the adult simultaneous emergences, most species undertake short dispersal flights, usually less than 5 km. Males disperse to find mates and a source of sugar, while females, the only hematophagous gender, also seek a blood meal and oviposition sites. The majority of black fly species worldwide feed mainly on mammals, although some feed on birds [19].

In our systematic review, 35 species have been recorded in France on cattle farms: *Prosimulium hirtipes, Prosimulium latimucro, Prosimulium rufipes, Prosimulium tomosvaryi, Simulium angustipes, Simulium angustitarse, Simulium argenteostriatum, Simulium argyreatum, Simulium aureum, Simulium auricoma, Simulium bertrandi, Simulium bezzii, Simulium brevidens, Simulium carthusiense, Simulium costatum, Simulium cryophilum, Simulium equinum, Simulium erythrocephalum, Simulium intermedium, Simulium latigonium, Simulium latipes, Simulium lineatum, Simulium monticola, Simulium noelleri, Simulium ornatum, Simulium posticatum, Simulium pseudequinum, Simulium reptans, Simulium rheophilum, Simulium trifasciatum, Simulium tuberosum, Simulium variegatum, Simulium rubzovianum, Simulium vernum*, and *Simulium xanthinum*. The different species distributions by department in France are described in Suppl. Fig. 1 and Suppl. Tab. 2.

*Bibliometric data*. We collected and analyzed 14 documents, with little data available on the presence/abundance of black flies in many regions of France. On average, two references are published every 10 years (Fig. 5F). The low number of publications indicates the need for further studies on these vectors, which are not well known in France, although they display an important vectorial role in other regions of the world, particularly in Africa.

*Trapping methods*. They are described for 69.2% of the documents (*N* = 9/13). Several methods can be used: collection of larvae and/or pupae (drift and benthic sampling) and adults (hand net, CDC trap) (Suppl. Tab. 2). Abundance is rarely described (*N* = 3/13; 23.1%), highly variable and expressed as a percentage of captures or as total of larvae per square meter (m^2^) (*e.g.*, 20 000–100 000 larvae/m^2^ in the Vosges department in 1978 [77]). These highly localized data do not allow for generalization to the whole territory.

*Veterinary importance*. They are responsible for direct and indirect nuisance (vector role). However, economic losses due to black flies are poorly documented. There are 3 reports of cattle deaths due to these insect bites in France [77]. Indeed, the bite number per cattle can be extremely high, ranging from 25,000 in May to 60,000 in July in the Vosges region (1978) [16]. The most frequent cause of mortality is attributed to acute toxic shock caused by the various salivary components, some of which are venomous, injected during the bite. However, no recent publication reports important or problematic abundance of this insect in France.

*Simulium ornatum* might be a vector of the dermal filaria *Onchocerca lienalis* (previously identified as *Onchocerca gutturosa* [4]) in the Vosges region (inoculation of microfilaria during the blood meal) (Suppl. Tab. 2). However, we did not collect any document more recent than the only publication of 1978 indicating a still active circulation of this worm little pathogenic.

### 3.10 Insecta, Diptera, Nematocera, Psychodidae: Sand flies

*Bio-ecology*. Sand flies are small (2–5 mm), humped insects with a hairy appearance due to an abundance of short setae on the head, thorax, wings, and abdomen. Adults range in color from light beige to dark brown. Adult sand flies feed on plant sap, nectar, and honeydew. In addition, females need blood for the development of their eggs. Adults rest near the larval sites and usually near hosts that provide a blood source. Seven species have been recorded in France: *Phlebotomus ariasi*, *Phlebotomus mascittii*, *Phlebotomus papatasi*, *Phlebotomus perfiliewi*, *Phlebotomus perniciosus*, *Phlebotomus sergenti*, and *Sergentomyia minuta*. The different species distributions in France by department are described in Suppl. Fig. 1 and Suppl. Tab. 2.

*Bibliometric data*. For sand flies, 30 records were retrieved and analyzed. Few data are available for most species (*e.g. Ph. mascittii*). On average, from the 1970s, 5 references are published every decade (Fig. 5G).

*Trapping methods*. Capture methods are described in 50% of the documents (*N* = 15/30). The traps most commonly used are: sticky traps, CDC traps and mouth aspirators (Suppl. Tab. 2). Abundance is also poorly described (*N* = 10/30; 33.3%), highly variable and preferably expressed as the number of individuals captured or as a percentage of species capture by traps or by trapping period. However, these results are also localized and therefore cannot be generalized to the whole territory.

*Veterinary importance*. Sand flies are a direct nuisance in areas where they are abundant, due to their painful bite. They are vectors, particularly in Southern France, of *Leishmania infantum*. They are also vectors of Toscana and Massilia viruses (Suppl. Tab. 2).

### 3.11 Insecta, Diptera, Nematocera, Culicidae: Mosquitoes

*Bio-ecology*. Mosquitoes are small to medium sized (between 5 and 15 mm). They are slender, with thin legs and narrow, elongated wings. Their bodies are covered with scales and bristles, creating characteristic markings and colors for each species. The larvae are aquatic.

There are about 3,500 species of mosquitoes [73]. In our systematic review 73 species were identified near cattle breeding: *Aedes aegypti, Aedes albopictus, Aedes annulipes, Aedes berlandi, Aedes cantans, Aedes caspius, Aedes cataphylla, Aedes cinereus, Aedes communis, Aedes detritus/coluzzii (Aedes detritus, Aedes coluzzii), Aedes diantaeus, Aedes dorsalis, Aedes echinus, Aedes excrucians, Aedes flavescens, Aedes geminus, Aedes geniculatus, Aedes japonicus, Aedes mariae, Aedes nigrinus, Aedes nigripes, Aedes pulcritarsis, Aedes pullatus, Aedes punctor, Aedes refiki, Aedes rossicus, Aedes rusticus, Aedes sticticus, Aedes surcoufi, Aedes vexans, Aedes vittatus, Anopheles algeriensis, Anopheles beklemishevi, Anopheles claviger, Anopheles hyrcanus, Anopheles maculipennis* s.l. (*Anopheles atroparvus, Anopheles labranchiae, Anopheles maculipennis* s.s.*, Anopheles melanoon, Anopheles messeae* et *Anopheles sacharovi*)*, Anopheles marteri, Anopheles petragnani, Anopheles plumbeus, Anopheles pseudopictus, Anopheles sinensis, Anopheles superpictus, Coquillettidia buxtoni, Coquillettidia richiardii, Culex apicalis, Culex brumpti, Culex hortensis, Culex impudicus, Culex laticinctus, Culex martinii, Culex mimeticus, Culex modestus, Culex pipiens, Culex territans, Culex theileri, Culex torrentium, Culex univittatus, Culiseta alaskaensis, Culiseta annulata, Culiseta fumipennis, Culiseta glaphyroptera, Culiseta litorea, Culiseta longiareolata, Culiseta morsitans, Culiseta subochrea, Orthopodomyia pulcripalpis*, and *Uranotaenia unguiculata*.

The two species of the *Aedes detritus/coluzzii* complex will be treated together. The same will be done for the species of the Maculipennis subgroup. At this time, according to the latest classification [49], ten species are officially recognized in this subgroup, of which 6 species are recorded in our study (*An. atroparvus*, *An. labranchiae*, *An. maculipennis*, *An. melanoon*, *An. messeae* and *An. sacharovi*) and 4 not reported (*Anopheles artemievi, Anopheles daciae, Anopheles martinius*, and *Anopheles persiensis*).

The trophic preferences of the different species are summarized in the Table 4 [85] and their distributions in France by department described in Suppl. Fig. 1 and Suppl. Tab. 2.

**Table 4:** Summary of the trophic preferences of the different mosquito species recorded in this systematic review.

Species	Trophic preference
*Uranotaenia unguiculata*	Autogenous (would not bite mammals)
*Aedes rossicus*	Biology not well known
*Culex brumpti*	
*Anopheles pseudopictus*	Biology not well known, doubtful species
*Culex apicalis*	Zoophilic (batrachians, reptiles)
*Culex hortensis*	
*Culex impudicus*	
*Culex martinii*	
*Culex territans*	
*Culex laticinctus*	Zoophilic (mammals)
*Orthopodomyia pulcripalpis*	
*Aedes flavescens*	
*Aedes cantans*	
*Aedes diantaeus*	
*Aedes excrucians*	
*Aedes nigripes*	
*Anopheles algeriensis*	
*Anopheles beklemishevi*	
*Anopheles claviger*	
*Anopheles hyrcanus*	
*Anopheles maculipennis* s.l.	
*Anopheles marteri*	
*Anopheles petragnani*	
*Anopheles superpictus*	
*Coquillettidia buxtoni*	
*Coquillettidia richiardii*	
*Culex modestus*	
*Culex theileri*	
*Culiseta alaskaensis*	
*Culiseta subochrea*	
*Aedes echinus*	
*Aedes aegypti*	Zoophilic (mammals, anthropophilic preference)
*Aedes albopictus*	
*Aedes annulipes*	
*Aedes berlandi*	
*Aedes cataphylla*	
*Aedes cinereus*	
*Aedes dorsalis*	
*Aedes geminus*	
*Aedes nigrinus*	
*Aedes pullatus*	
*Aedes refiki*	
*Aedes sticticus*	
*Aedes surcoufi*	
*Anopheles sinensis*	
*Culex pipiens*	
*Culex mimeticus*	Zoophilic (birds)
*Culex torrentium*	
*Culiseta annulata*	
*Culiseta glaphyroptera*	
*Culiseta longiareolata*	
*Culiseta morsitans*	
*Aedes caspius*	Zoophilic (birds, mammals)
*Aedes communis*	
*Aedes geniculatus*	
*Aedes japonicus*	
*Aedes mariae*	
*Aedes pulcritarsis*	
*Aedes punctor*	
*Aedes rusticus*	
*Aedes vexans*	
*Aedes vittatus*	
*Culex univittatus*	
*Aedes detritus/coluzzii*	Zoophilic (birds, mammals, anthropophilic preference)
*Culiseta fumipennis*	Zoophilic (birds, reptiles)
*Anopheles plumbeus*	Zoophilic (birds, reptiles, mammals)
*Culiseta litorea*	Zoophilic (opportunistic, ornithophilic preference)

*Bibliometric data*. A total of 69 documents were retrieved and analyzed, with a significant amount of data available on the presence/abundance of mosquitoes in many regions of France. On average, since the 1960s, eight references have been published every decade (Fig. 5H). However, most publications address the species distributions without mentioning the importance of cattle farming on these distributions.

*Trapping methods*. Capture methods are described for 32 documents (*N* = 32/69; 46.4%). Several methods can be used: collection of eggs, larvae and/or pupae (manual collection, *e.g.* dipping) and adults (hand net, aspirator (natural environment, human or host capture), CDC trap (with or without CO_2_), BG-sentinel, Ovitrap). However, CDC traps have more or less replaced human and animal bait as a routine sampling method [91]. Indeed, along with the ovitraps, they have become the reference traps for monitoring mosquito populations. Abundance is rarely described (*N* = 7/69; 10.1%), highly variable and expressed as percentage of captures or total number of captures (larvae or adults). However, these results are localized and cannot be generalized. Indeed, the species abundance is extremely dependent on local and environmental conditions as well as on their bio-ecology.

*Veterinary importance*. In addition to their importance as vectors of animal diseases, mosquitoes are also a nuisance causing irritation, blood loss, and allergic reactions. However, these nuisances appear not to be documented in France. Mosquitoes can transmit, especially to humans, numerous pathogens, some of which circulate or have circulated in France (*e.g.*, Chikungunya virus, Dengue virus, West Nile virus, Usutu virus, *Spiroplasma cantharis*, *Spiroplasma sabaudiense, Dirofilaria immitis, Dirofilaria repens, Filaria brancrofti, Plasmodium danilewskyi, Plasmodium vivax, Plasmodium falciparum,* and *Plasmodium relictum*) (Suppl. Tab. 2).

### 3.12 Insecta, Diptera, Nematocera, Ceratopogonidae: Biting midges

*Bio-ecology*. Biting midges are small (1–2.5 mm) with scale-free wings and light and dark areas (used for species identification). There are more than 1,300 species, almost all of which are hematophagous [32]. These insects bite mammals, birds, and reptiles. In our systematic review, 98 species were recorded in France: *Alluaudomyia needhami, Bezzia flavicornis, Bezzia pygmaea, Culicoides abchazicus, Culicoides accraensis, Culicoides achrayi, Culicoides alazanicus, Culicoides albicans, Culicoides albipennis, Culicoides begueti, Culicoides brunnicans, Culicoides cameroni, Culicoides cataneii, Culicoides caucoliberensis, Culicoides chiopterus, Culicoides circumscriptus, Culicoides clastrieri, Culicoides clintoni, Culicoides comosioculatus, Culicoides corsicus, Culicoides deltus, Culicoides derisor, Culicoides dewulfi, Culicoides duddingstoni, Culicoides dzhafarovi, Culicoides fagineus, Culicoides fascipennis, Culicoides festivipennis, Culicoides flavipulicaris, Culicoides furcillatus, Culicoides gejgelensis, Culicoides gornostaevae, Culicoides griseidorsum, Culicoides grisescens, Culicoides haranti, Culicoides heliophilus, Culicoides heteroclitus, Culicoides ibericus, Culicoides imicola, Culicoides impunctatus, Culicoides indistinctus, Culicoides jumineri, Culicoides jurensis, Culicoides kibunensis, Culicoides kurensis, Culicoides longipennis, Culicoides lupicaris, Culicoides malevillei, Culicoides manchuriensis, Culicoides maritimus, Culicoides minutissimus, Culicoides montanus, Culicoides musicola, Culicoides newsteadi, Culicoides nubeculosus, Culicoides obsoletus, Culicoides odiatus, Culicoides pallidicornis, Culicoides paolae, Culicoides paradisionensis, Culicoides paradoxalis, Culicoides parroti, Culicoides pictipennis, Culicoides picturatus, Culicoides poperinghensis, Culicoides pseudoheliophilus, Culicoides pseudopallidus, Culicoides pulicaris, Culicoides pumilus, Culicoides punctatus, Culicoides puncticollis, Culicoides reconditus, Culicoides riebi, Culicoides riethi, Culicoides riouxi, Culicoides saevus, Culicoides sahariensis, Culicoides salinarius, Culicoides santonicus, Culicoides scoticus, Culicoides segnis, Culicoides sejfadinei, Culicoides semimaculatus, Culicoides sergenti, Culicoides shaklawensis, Culicoides sigrosignatus, Culicoides simulator, Culicoides sphagnumensis, Culicoides stigma, Culicoides subfagineus, Culicoides subfasciipennis, Culicoides tauricus, Culicoides tbilisicus, Culicoides truncorum, Culicoides univittatus, Culicoides vexans, Culicoides vidourlensis*, and *Palpomyia lineata*.

The classification of biting midges is still poorly defined [50]. Consequently, for the purposes of this study, some species will be clustered together in “groups”, as defined by French research teams [9–12]. These groups have no taxonomic significance but allow the classification of species present in France. The groups are : Obsoletus (*C. obsoletus* and *C. scoticus*), Pulicaris (*C. pulicaris*, *C. lupicaris* and *C. flavipulicaris*) Punctatus (*C. punctatus* and *C. newsteadi*), Nubeculosus (*C. nubeculosus, C. puncticollis* and *C. riethi*), Achrayi (*C. achrayi, C. fascipennis*, *C. pallidicornis, C. picturatus* and *C. subfasciipennis*), Circumscriptus (*C. circumscriptus, C. salinarius* and *C. sphagnumensis*), Fagineus (*C. fagineus and C. subfagineus*), and Festivipennis (*C. festivipennis, C. clastrieri, C. paolae* and *C. shaklawensis*). Similarly, morphologically related species grouped together in some publications will be processed together: *Culicoides cataneii*/*gejgelensis* (*C. cataneii* and *C. gejgelensis*). In addition, two species (*C. musicola* and *C. sigrosignatus*), mentioned only once in 1925 [56], are not referenced in the world catalogue of *Culicoides* [17] and will therefore not be considered in our study. Finally, the species *C. sejfadinei* referenced in two documents [41, 87] and always in association with *C. tauricus* will be processed with the latter (*Culicoides sejfadinei/tauricus*). The different species distributions by department in France are described in Suppl. Fig. 1 and Suppl. Tab. 2.

*Bibliometric data*. A total of 70 documents were collected and analyzed. Numerous publications (*N* = 23) are available on the abundance of biting midges in France. On average, since the 1960s, 11 references have been published every decade (Fig. 5I). Moreover, an increase in publications can be observed from the 2000s onwards. This is the result of the appearance in France of Bluetongue then Schmallenberg viruses (of which *Culicoides* are vectors) and has enabled the setting up of a surveillance network. As a result, we have standardized and detailed data on the abundance of many species [41] (Suppl. Fig. 1).

*Trapping methods*. Capture methods are described for 49 documents (46.4%). Several methods can be used: collection of larvae (soil sampling), adults capture by emergence trap, CDC trap (with or without UV), hosted capture, and OVI trap. The OVI trap method was used in 59.2% (*N* = 29/49) of the documents. Indeed, this trap was employed in the *Culicoides* population monitoring system deployed in France [12]. Moreover, it allows the capture of large numbers of individuals, and is a reliable and practical method for determining the presence/abundance of biting midges in a given area [102].

Abundance is poorly described (*N* = 25/70; 35.7%), and is expressed as a number or percentage of captures. However, the setting up of the monitoring network has enabled us to obtain standardized and comparable data on many species abundance [41] (Suppl. Fig. 1).

*Veterinary importance*. Due to their high abundance, they can represent a direct nuisance (*e.g.*, > 13,000 individuals in a trap in a few hours [109]). In addition, they are vectors of numerous pathogens (*e.g.*, > 35 arboviruses [73]). In France, due to the successive outbreaks of Bluetongue virus (BTV) in 2006–2008 [71], most studies have focused on this virus. However, the systematic review also highlighted the role *Culicoides* as vectors of *Chlamydia* sp. (*C. clastrieri* and *C. festivipennis*), *Onchocerca cervicalis*, and *Onchocerca reticulata* (*C. nubeculosus*) (Suppl. Tab. 2).

### 3.13 Arachnida, Ixodida: Ticks

*Bio-ecology*. Adult ticks measure about 3 to 5 mm depending on age, sex, species and their engorgement level. They are external parasites that live by feeding on the blood of mammals, birds and sometimes reptiles and amphibians. The Ixodida include three families: (1) Ixodidae, the hard ticks, (2) Argasidae, the soft ticks, and (3) Nuttalliellidae, with the only known species, *Nuttalliella namaqua*, present in Eastern and Southern Africa and will not be discussed here [73]. In our systematic review, 30 species were recorded: *Amblyomma variegatum, Argas reflexus, Argas vespertilionis, Dermacentor marginatus, Dermacentor reticulatus, Haemaphysalis concinna, Haemaphysalis inermis, Haemaphysalis punctata, Haemaphysalis sulcata, Hyalomma aegyptium, Hyalomma detritum, Hyalomma excavatum, Hyalomma lusitanicum, Hyalomma marginatum, Hyalomma scupense, Ixodes acuminatus, Ixodes canisuga, Ixodes festai, Ixodes frontalis, Ixodes hexagonus, Ixodes ricinus, Ixodes trianguliceps, Ixodes ventalloi, Ixodes vespertilionis, Ornithodoros coniceps, Rhipicephalus annulatus, Rhipicephalus bursa, Rhipicephalus pusillus, Rhipicephalus sanguineus*, and *Rhipicephalus turanicus.* The different species distributions in France by department are described in Suppl. Fig. 1 and Suppl. Tab. 2.

*Bibliometric data*. We collected and analyzed a large number of papers (*N* = 122). This is the arthropod group with the most publications in our systematic review. On average, 22 references have been published every 10 years since the 1970s (Fig. 5J).

*Trapping methods*. Capture methods are most often described (*N* = 92/121; 76%). Two methods are used: flagging (*N* = 65; 53.7%) and manual capture on host (*N* = 45; 37.2%). Abundance was described in 62 documents (51.2%). Data are very variable and expressed as the total number of individuals captured (larvae and/or pupae and/or adults) or as a percentage of capture (*N* = 50/62; 80.6%), as the number of individuals (larvae and/or pupae and/or adults) per 100 m^2^ (*N* = 2/62; 3.2%), per 1 0 m^2^ (*N* = 3/62; 4.8%), per animal (*N* = 2/62; 3.2%), in 1 h on 2,000 m^2^ (*N* = 1/62; 1.6%), per hour/flag (*N* = 1/62; 1.6%), by monthly pupal density index (*N* = 1/62; 1.6%), in maximum average abundance (*N* = 1/62; 1.6%), in average population size (*N* = 1/62; 1.6%). Due to the diversity of the results, they cannot be generalized to the whole country.

*Veterinary importance*. Ticks are vectors of many pathogens, such as bacteria (*Anaplasma* spp., *Bartellona* spp., *Borrelia* spp., *Coxiella burnetii*, *Ehrlichia* spp., *Francisella* spp., *Rickettsia* spp., and *Candidatus* spp.), protozoa (*Babesia* sp., *Theileria* sp.), and viruses. Pathogens and their tick vectors, identified in our systematic review, are detailed in the Table 5.

**Table 5:** Summary of pathogens and their tick vectors.

Pathogen		Species
*Anaplasma*	*A. centrale*	*Ixodes ricinus*
	*A. marginale*	*Dermacentor marginatus, Dermacentor reticulatus, Haemaphysalis punctata, Hyalomma marginatum, Ixodes acuminatus, Ixodes ricinus, Rhipicephalus annulatus, Rhipicephalus bursa, Rhipicephalus sanguineus*
	*A. ovis*	*Dermacentor marginatus, Haemaphysalis punctata*
	*A. phagocytophilum*	*Dermacentor marginatus, Haemaphysalis punctata, Hyalomma marginatum, Hyalomma scupense, Ixodes ricinus, Rhipicephalus bursa, Rhipicephalus pusillus, Rhipicephalus sanguineus*
*Babesia*	*B. annulatus*	*Ixodes ricinus*
	*B. bigemina*	*Ixodes ricinus, Rhipicephalus bursa*
	*B. bovis*	*Dermacentor marginatus, Dermacentor reticulatus, Haemaphysalis punctata, Ixodes ricinus, Rhipicephalus bursa, Rhipicephalus sanguineus*
	*B. caballi*	*Dermacentor marginatus*
	*B. divergens*	*Dermacentor marginatus, Dermacentor reticulatus, Haemaphysalis punctata, Ixodes acuminatus, Ixodes ricinus*
	*B. major*	*Haemaphysalis punctata, Ixodes ricinus*
	*B. microti*	*Ixodes ricinus*
	*B. ovis*	*Rhipicephalus bursa*
	*B. venatorum*	*Ixodes ricinus*
*Bartonella*	*Ba. capreoli*	*Ixodes ricinus*
	*Ba. grahamii*	*Ixodes ricinus*
	*Ba. henselae*	*Dermacentor marginatus, Haemaphysalis punctata, Hyalomma marginatum, Ixodes ricinus, Rhipicephalus annulatus, Rhipicephalus bursa, Rhipicephalus sanguineus*
*Borrelia*	*Bo. afzelii*	*Ixodes ricinus*
	*Bo. burgdorferi*	*Dermacentor reticulatus, Haemaphysalis punctata, Hyalomma marginatum, Ixodes ricinus*
	*Bo. caballi*	*Haemaphysalis punctata*
	*Bo. garinii*	*Ixodes ricinus*
	*Bo. lusitaniae*	*Ixodes ricinus*
	*Bo. miyamotoi*	*Haemaphysalis punctata, Ixodes ricinus*
	*Bo. spielmanii*	*Ixodes ricinus*
	*Bo. turdi / lusitaniae*	*Ixodes ricinus*
	*Bo. valaisiana*	*Ixodes ricinus*
*Candidatus*	*C. barbariae*	*Rhipicephalus bursa*
	*C. mikurensis*	*Ixodes ricinus*
	*C. urmitei*	*Rhipicephalus bursa*
*Coxiella*	*Co. burnetii*	*Dermacentor marginatus, Dermacentor reticulatus, Haemaphysalis punctata, Ixodes ricinus, Rhipicephalus sanguineus*
*Ehrlichia*	*E. canis*	*Ixodes ricinus*
	*E. minasensis*	*Hyalomma marginatum, Rhipicephalus bursa*
*Francisella*	*F. philomiragia*	*Dermacentor marginatus, Dermacentor reticulatus, Ixodes ricinus*
	*F. tularensis*	*Dermacentor marginatus, Dermacentor reticulatus, Ixodes ricinus*
*Rickettsia*	*R. aeschlimannii*	*Haemaphysalis punctata, Hyalomma marginatum, Hyalomma scupense, Ixodes ricinus, Rhipicephalus bursa, Rhipicephalus sanguineus*
	*R. africae*	*Amblyomma variegatum*
	*R. canadensis*	*Ixodes ricinus*
	*R. conorii*	*Dermacentor marginatus, Ixodes ricinus, Rhipicephalus sanguineus*
	*R. felis*	*Ixodes ricinus*
	*R. helvetica*	*Ixodes ricinus*
	*R. raoultii*	*Dermacentor reticulatus*
	*R. slovaca*	*Dermacentor marginatus, Hyalomma scupense, Rhipicephalus sanguineus*
*Theileria*	*T. annulata*	*Ixodes ricinus*
	*T. buffeli*	*Dermacentor marginatus, Haemaphysalis punctata, Ixodes ricinus*
	*T. orientalis*	*Haemaphysalis punctata, Ixodes ricinus*
Virus	Tick-borne Jingmenvirus	*Ixodes ricinus*
	Tick Borne Encephalitis	*Ixodes ricinus*
	Virus Omsk	*Ixodes ricinus*
	Parapoxvirus	*Hyalomma marginatum, Hyalomma scupense, Rhipicephalus bursa*

## 4 Discussion

### 4.1 Livestock

Of the 331 reports collected in the systematic review, over 85% did not provide information on the cattle farms in which the study was conducted (*e.g.*, cattle breeds, farm size, production, and breeding types). The absence of data (and their disparity for those available) make it impossible to determine the favorable factors allowing the presence and maintenance of arthropod populations on cattle farms.

In view of these results, it would seem necessary to define a series of recommendations concerning the minimum farm characteristics to be included in future publications as well as the presence or absence of livestock near the trap. In addition, the use of different capture methods does not enable direct comparison of the entomological data collected [68]. Therefore, the publication of a standardized capture method, general (*e.g.*, field of forensic entomology [2]) and/or by insect order (*e.g.*, standardization of *Culicoides* capture and counting methods by the surveillance network [41]), should be discussed, described, and published. Consideration should be given to create a document representing the opinion and guidelines of the specialized expert committees on minimum standards in veterinary entomology. The main objective of this document would be to encourage a high level of competence and to promote and establish common standards of practice, particularly with regard to the collection of environmental data and blood-feeding arthropods. It could be divided into a general protocol and specific methodologies. First, general protocol would include generally accepted and mandatory principles for the practice of veterinary entomology (*e.g.*, data to be collected on livestock and environmental conditions). Variations from this protocol due to environmental or specific factors are not expected. Second, specific methodologies would be practical recommendations by arthropod “groups”, identifying a particular strategy (*e.g.*, collection, counting, and identification methods); variations may be acceptable if motivated or based on specific cases.

Using a standardized method for collecting data would: (1) improve knowledge of the hematophagous arthropods biology and ecology (*e.g.*, trophic behavior); (2) promote a multidisciplinary approach; (3) improve the articles citation index related to these topics; and (4) minimize the wide variety of publication styles and research methods.

### 4.2 Arthropods

*Fleas*. Only one species was found on cattle in our systematic review: the cat flea (*Ctenocephalides felis*) without any mention of other species such as *Ctenocephalides canis* or *Pulex irritans*. The cat flea is an important ectoparasite for mammals due to its wide host range (*e.g.*, humans, cats, dogs, cattle, horses, sheep, *etc.*) [51, 73]. Occasionally, fleas can infest calves in large numbers and cause anemia and susceptibility to secondary infections [29, 30, 107]. In cattle, they do not seem to be involved in the vectorial transmission of pathogens. However, due to the very low number of publications (*N* = 5), and the lack of data on the prevalence and abundance of fleas on cattle farms, further studies are needed to determine the economic impact of infestations on production (low *vs.* massive, with thresholds to be defined).

*Lice*. They are extremely common parasites in production animals. Dairy, meat, and mixed breeds are affected. Livestock lice are responsible for economic losses worldwide [73]. These losses can be due to a direct effect of the infestation (*e.g.*, weight loss, skin infections, blood loss, anemia, *etc.*). Effects, documented when 10 or more lice per inch square are present on the animal [100], cost more than 125 million US$ per year in the United States [19]. The skin lesions are responsible for a depreciation of the leather, resulting for example in a loss of GB£ 35 million in potential revenue for the British leather industry [20]. No vectorial role has been reported in the analysis. Further studies are needed to determine the prevalence of infestations in cattle in France, and the possible impact of lice on animal health and production, especially in case of massive infestations (with a threshold of > 50 lice per inch square) [100].

*Horse flies*. Many species (*N* = 83) are present on the French territory. Horse flies are a significant nuisance as their abundance can be comparable as the one of biting flies [101]. Their bites are painful and serious local reactions can develop. Blood loss from bites can be significant (up to 0.5 mL per fly) [36]. In addition to their nuisance, horse flies are mechanical vectors (*i.e.*, act as a contaminated syringe) of many pathogens in France [6], including viruses (equine infectious anemia virus, bovine leukemia virus, bovine viral diarrhea virus, Rinderpest virus, and tick-borne encephalitis virus), bacteria (*Anaplasma marginale, Francisella tularensis, Bacillus anthracis, Borrelia burgdorferi, Coxiella burnetii, Pasteurella multocida, Brucella* sp., *Listeria monocytogenes*, and *Erysipelothrix rhusiopathiae*), protozoa (*Haemoproteus metchnikovi, Besnoitia besnoiti, Trypanosoma evansi Trypanosoma theileri*, and *Trypanosoma equiperdum*). Horse flies are also biological vectors of filarial nematodes (*Elaeophora schneideri*) and protozoa (*Trypanosoma theileri* and *Haemoproteus metchnikovi*) [6]. However, studies reporting the confirmation of their vectorial role under experimental conditions are rare, mainly due to the absence of laboratory colonies for experimental trials. Furthermore, these suspected transmission events should be viewed with caution, especially the epidemiological importance which would require further evaluation [73].

Horse flies are widely distributed on the French territory. However, at the species taxonomic level, data are lacking regarding their distribution, temporality, relative abundance and vector role. In addition, it would be necessary to determine their economic impact on French cattle farms. These data are available, for example, in the United States (US$ 40 million per year in beef cattle production losses [36], decrease in average gain of 0.09 kg per animal per day [84]).

*Louse flies*. Our systematic review has listed only one species identified and reported on cattle in France: *Hi. equina*. This species is normally a parasite of equids (*e.g.*, horses and donkeys) but can be a facultative parasite of cattle. This species is widespread and common on a wide variety of domestic animals [73]. The bites are painful and these insects could potentially carry protozoa responsible for equine piroplasmosis, and bacteria (*Coxiella burnetii*, *Rickettsia* sp.) [19]. Again, few studies have been performed on this model. The importance of *Hi. equina* in the transmission of pathogens could be underestimated. Indeed, this species transmits *Corynebacterium pseudotuberculosis* [42], a bacterium responsible for significant economic losses in sheep (17 million US$ of losses in wool production in Australia [83]) and also in cattle (US$ 17 000 of losses for a farm in Israel [106], and a high slaughter rate (16.3%) and decrease in average monthly milk production (6%) [105]). This pathogen is present in England (in goats since 1990, and in sheep since 1991 [21]) and in France (human cases in 2006 [55]). Therefore, further investigations are needed to assess the potential impact of louse flies on animal production and health.

*Biting flies*. Three species are present on the whole French territory: *Ha. irritans, Ha. stimulans*, and *S. calcitrans*. All three are responsible for nuisance and important economic losses for the bovine sector (milk or meat) by a direct (harassment, blood spoliation up to 1 L of blood per day [13]) and indirect (vectors) roles, which is more studied for *S. calcitrans*. Indeed, this species is a potential vector of many pathogens (*e.g.* Bacteria (*Dermatophilus congolensis, Anaplasma marginale,* and *Bacillus anthracis*), Protozoa (*Besnoitia besnoiti*, *Trypanosoma evansi*), and viruses (bovine viral diarrhea virus, lumpy skin disease virus, Rift Valley fever virus, bovine leukosis virus, and West Nile virus) [92].

The economic losses due to biting flies are very high, with an estimated economic impact threshold for stable fly of about 25 individuals/animal/day [96]. In Brazil [46] and Mexico [86], the economic losses for the cattle industry represent US$ 2,558.32 and 231.67 million, respectively for *Ha. irritans*, and US$ 335.46 and 6.79 million for *S. calcitrans*, respectively. Similarly, in the United States, the economic impact of *S. calcitrans* has been estimated to be more than US$ 400 million per year for the beef industry [28, 97] and more than US$ 1 billion for the dairy industry [98]. However, little research has been carried out in France on these insects in relation to our literature collection. Consequently, it would be necessary to determine their economic impact on French cattle farms as well as the role and importance of stable flies in the epidemiology of these different pathogens.

*Black flies*. Numerous species are present on the territory (*N* = 35). However, in France, few documents are available on black flies, clinical consequences and economic losses due to these insects. These data exist in other countries in the tourism or cattle industries, for example in the United States (US$ 27,202 of losses due to nuisance in a South Carolina golf club [44]), in Turkey (US$ 5.45 million of economic losses in the Cappadocia region following an outbreak of *Simulium* sp. [89]) and in Canada (~US$ 3 million of losses in meat and milk production [39]). Moreover, these economic losses do not usually take into account additional indirect costs (*e.g.*, extra feed, fence repairs, extra staff salaries, increased insurance costs, veterinary fees, and medication costs) [39]. Black flies represent a nuisance that seems to be punctual and localized. There are few old reports of cattle deaths following a massive and simultaneous attack by swarms of black flies and of transmission by *Simulium ornatum* of the low pathogenic filaria *O. lienalis* (previously identified as *O. gutturosa* [4]) in the Vosges in 1978. Based on the documents collected, black flies do not appear to be an important cause of nuisance or economic loss on French cattle farms, but this may be underestimated. Therefore, further studies would be required to evaluate their health and economic impacts on cattle farms in France more precisely.

*Sand flies*. To our knowledge, they are a relatively rare nuisance in cattle, only in precise localized areas, where they are very abundant. The economic impact of their nuisance on cattle farms remains unknown [73]. However, some species constitute a large portion of the barns microfauna (*e.g.*, *Ph. perniciosus*, *Ph. papatasi*) and the presence of cattle is correlated with a higher densities of sand fly populations [40, 47, 64]. Moreover, they are vectors of *Le. infantum*, Toscana, and Massilia viruses. Of the 7 species recorded in France, two are the main vectors (*Ph. perniciosus* and *Ph. ariasi*). Few data are available on the involvement and importance of cattle in the cycle of these different pathogens.

In 2009, a case of autochthonous symptomatic bovine visceral leishmaniasis, *Leishmania martiniquensis* (formerly wrongly identified as *Le. siamensis*), was identified in Switzerland [63]. The main vector has not yet been identified, but there is a possibility that cattle could act as a reservoir host (*e.g.* Kala-azar in India [57]). This highlights the need to define the vector risk, but also to monitor the evolution, arrival, and possible spread of this disease in France. The reservoir of Toscana virus is not yet known [74]. At present, the epidemiological role (*e.g.*, reservoir, accidental host) of cattle is not defined.

Therefore, sand flies would appear to be of low economic importance in cattle farms, but this is probably due to a lack of studies. Their health importance remains to be investigated. Further studies on these vectors and the pathogens they may transmit to cattle should be encouraged.

*Mosquitoes*. Mosquitoes are a nuisance that disrupt the normal behavior of livestock. Increased scratching behavior can lead to skin lesions, hair loss, and secondary infections. In cattle, mosquito bites can lead to decreased weight gain and milk production and cause farmers to change their grazing practices [81]. For example, cattle production losses due to mosquitoes are estimated to be US$ 38.7 million annually in the United States [19]. The average daily gain is correlated with the abundance of mosquitoes [94] and the control of hematophagous arthropods allows an increase in the average gain between 8 and 20 kg per animal per year [95]. Cattle deaths due to anemia and stress have also been reported [73]. In addition, mosquitoes transmit many pathogens for which cattle can be a reservoir/amplifier (*e.g.*, Rift Valley fever virus [76, 81, 99]). Moreover, the presence and density of livestock favors the colonization and abundance of mosquitoes [18, 66]. However, few studies, particularly in France, have been carried out on the impact of mosquitoes and their pathogens on milk and meat production or on the role of cattle in transmission cycles (*e.g.*, cattle playing a major role in the transmission and circulation of Rift Valley fever virus in Tanzania [52]).

*Biting midges*. They can be a major nuisance, causing, for example, recurrent summer dermatitis in horses through their saliva. To our knowledge, no documents are available on their economic impacts on cattle farms in France. However, such data exist, for example, in India (4–19% reduction in milk production [75, 82]). They are also vectors of numerous pathogens such as *O. gutturosa* [4, 26]. The bluetongue virus has been the subject of numerous studies and has led to the establishment of a surveillance network following its introduction into Corsica in 2000 [12]. Bluetongue outbreaks have resulted in significant economic losses (*e.g.* the 1978 outbreak occasioned a loss of US$ 6 million in the United States [70]). *Culicoides imicola* is the main vector but many species are also considered vectors: *C. newsteadi*, *C. obsoletus*, *C. scoticus*, *C. dewulfi*, *C. chiopterus*, and *C. pulicaris* [103]. The presence of this surveillance network has provided decision makers with essential information to identify periods and areas at risk and to guide the allocation of resources for surveillance and control. However, in the context of global changes, it is important to maintain this surveillance in order to be able to estimate and model the Bluetongue risk of establishment and spread but also for other pathogens. Indeed, other pathogens, transmitted by *Culicoides*, should be monitored for cattle in France: Epizootic haemorrhagic disease virus, Palyam viruses, Bovine Ephemeral Fever virus, Akabane virus, and Schmallenberg virus [59, 69, 90] (data summarized in Table 6).

**Table 6:** Summary of pathogens, transmitted by Culicoides, to be monitored for cattle in France.

Pathogen	Potential vectorpresent	Distribution	Citation
Epizootic Hemorrhagic Disease virus	Unknown	America, Africa, Southeast Asia, Japan and Australia	Mellor *et al.* 2000 [69]
Palyam virus	*Culicoides imicola*	Africa, Asia and Australia	Mellor *et al.* 2000 [69]
Bovine Ephemeral Fever virus	*Culicoides imicola*	Africa, Middle East, India, China, Southeast Asia, Japan, Indonesia and Australia	Mellor *et al.* 2000 [69]
Akabane virus	*Culicoides imicola*	Africa, Middle East, Southeast Asia and Australia	Mellor *et al.* 2000 [69]
Schmallenberg virus*	*Culicoides imicola, Culicoides newsteadi, Culicoides obsoletus/Culicoides scoticus*	Europe	Ségard *et al.* 2018 [90]; Koenraadt *et al.* 2014 [59]

* Virus present in France.

*Ticks*. They have both a direct pathogenic role (*e.g.*, blood spoliation, toxicosis, allergic reactions, superinfection, *etc.*) and an indirect role through the transmission of numerous pathogens (bacteria (*Anaplasma* sp., *Bartonella* sp., *Candidatus* sp., *Coxiella burnetii*, *Ehrlichia* sp., *Francisella* sp., and *Rickettsia* sp.), protozoa (*Babesia* sp., *Theileria* sp.), and various viruses as reported in Table 5) [65]. Ticks are responsible for significant economic losses worldwide (*e.g.*, *Anaplasma phagocytophilum* responsible for productivity losses in dairy cows [15]). Indeed, according to the FAO, these losses are estimated at US$ 7.3/animal/year (production losses and control costs) [37]. However, few documents exist on their economic impact on cattle farms in France. Such data are available from many countries, such as, for example, India (US$ 595.07 million in milk loss, treatment costs, and leather loss [93]), Uganda (US$ 308,144 for tick control and tick-borne diseases in Lake Mburo National Park [78]), and Tanzania (US$ 364 million in losses due to tick-borne diseases [58]). Further studies are needed to determine the prevalence of infestations in cattle in France, and the impact of ticks on animal health and production. Indeed, tick-borne pathogens are actively circulating in France (*e.g.*, Crimean-Congo hemorrhagic fever virus in Corsica [45], *Borrelia burgdorferi* s.l. present throughout France and responsible, on average, for 53 cases/100,000 inhabitants of Lyme disease per year [35]). Moreover, some of these pathogens could be associated with new transmission risks. Indeed, the tick-borne encephalitis virus could survive in milk used to manufacture dairy products [53]. Further studies should be conducted to assess the infectious risk associated with milk and dairy products.

Although more data are available for ticks than for other hematophagous arthropods groups, many data are still missing (*e.g.*, abundance on the French territory, economic impact). In the context of global changes, tick populations must be monitored. Indeed, tick abundance is more influenced by environmental factors (*e.g.*, temperature, humidity) than by host density, even though hosts play an important role in tick dissemination, especially for ubiquitous ticks (*e.g.*, *I. ricinus*) [65]. Furthermore, at this time, primary prevention against ticks remains the most effective method to avoid both human and animal vector-borne diseases [65].

### 4.3 Entomological expertise in France

The number of medical and veterinary entomologists in France is very low (about 100 in 2002 [23]). Among these, systematists/taxonomists are even less numerous. Some groups of hematophagous arthropods (1) no longer have an entomologist expert in France (*e.g.*, black flies and fleas), (2) have a bio-ecology that is still poorly known (*e.g.*, horse flies, sand flies), and (3) present uncertain or unknown vector risks (*e.g.*, biting flies, sand flies). There is a significant under-representation of entomologist experts in some insect orders (*e.g.*, biting flies, black flies, horse flies, sand flies, lice, fleas, and louse flies) associated with an aging entomologist population. The risk of appearance or reappearance in France of diseases (emerging or re-emerging) through vectors (indigenous or invasive) is non-negligible for cattle herds.

Regarding the most neglected vectors, the numerous gaps in knowledge about these arthropods, associated with the lack of experts, do not allow at present, and in the future if the situation remains the same, to (1) identify and characterize vectors, (2) implement control strategies, (3) prevent the transmission and spread of pathogens, and (4) reduce production losses and associated economic costs.

Therefore, it would be necessary to (1) support the training and recruitment of veterinary entomologists, (2) encourage entomological studies on cattle, particularly on the most neglected vectors, (3) set up standardized studies to be able to compare the abundance of arthropods on the territory and thus identify the parameters and the breeding practices favorable to their presence, and finally (4) carry out trapping to characterize the presence of hematophagous arthropods on different types of cattle farms on the national territory.

## 5 Conclusion

In this work, we showed that there is no standardization and indicators regarding arthropod collection on cattle farms. Indeed, more than half the documents collected do not provide a description of cattle farms (*e.g.*, farm size, cattle breed, *etc.*). This result demonstrates the need to define a set of recommendations regarding minimum cattle farm characteristics (*e.g.*, presence/absence of livestock in the area, farm size, *etc.*) to be included in scientific publications. These data on cattle should be associated with standardized capture methods.

Despite the large number of documents collected and analyzed, there is little data on the presence/abundance of cattle hematophagous arthropods. Some arthropod groups are clearly under-documented (louse flies, fleas, lice, biting flies, sand flies, biting midges, and horse flies), and all groups have sources that lack numerical detail and are based on limited data in time and/or space and therefore not generalizable or comparable. There is still little information on many vectors (and their pathogens) and still many unknowns for the most studied groups (*e.g.*, information on *Babesia major* and *Theileria orientalis*, transmitted by ticks, is very sparse and insufficient [62]).

It appears necessary to provide new, updated, and standardized data, collected in different geographical and climatological areas (*e.g.*, vector abundance, parasite prevalence, clinical incidence, *etc.*) [62]. Vectors and their pathogens can only be controlled within a reasonable time frame if the details of their bio-ecology (*e.g.*, life cycle, trophic and reproductive behavior, sensitivity to control measures, *etc.*) are known [67]. In addition, the lack of experts in medical and veterinary entomology, associated with the lack of funding, training and government support, has existed for several decades and has thus led to an accumulation of increasingly unresolved problems caused by ticks and insects (*e.g.*, resistance to insecticides, unknown importance of mechanical transmission, lack of methods to exclude or minimize long-distance transport of potentially infected vectors, *etc.*), especially in the era of intensive globalization and global warming [67].

Finally, the influence of global warming on the transmission of parasitic diseases requires further research. These changes will alter biotic and abiotic conditions, changing ecological barriers, and thus redrawing the current distribution maps of pathogens, their vectors, and their hosts [3]. For example, an increase in temperature may allow vectors to migrate to new areas, increase their abundance, extend their activity period, or contribute to more rapid development of the vector or pathogen [108]. These areas need to be studied intensively in order to avoid the occurrence of epizootic outbreaks with medical, veterinary, and economic consequences.

## Data Availability

All resources used in this article are provided in the Supporting Information and all the analyses are detailed allowing the assessment or verification of the manuscript’s findings.

## Supplementary material

The Supplementary materials of this article are available at https://www.parasite-journal.org/10.1051/parasite/2023059/olm. *Supplementary Figure 1*. Distribution map of the different hematophagous arthropods identified in the systematic review by department according to the number of references and, if available, the percentage of capture. *Supplementary Table 1*. Complete list of references included in the systematic review. *Supplementary Table 2*. Description of captures by species of the different hematophagous arthropods identified in the systematic review.
