# Supplementary material for: Systematic review of hematophagous arthropods present in cattle in France
Source: Parasite. 2023 Dec 12;30:56. doi: 10.1051/parasite/2023059 (PMC10714678; doi:10.1051/parasite/2023059)
Supplement: Supplementary file 1 — Supplementary Figure 1. Distribution map of the different hematophagous arthropods identified in the systematic review by department according to the number of references and, if available, the percentage of capture. Supplementary Table 1. Complete list of references included in the systematic review. Supplementary Table 2. Description of captures by species of the different hematophagous arthropods identified in the systematic review. [file parasite-30-56-s1.zip › Suppl_Fig_1_corrected.pdf]

Supplementary Figure legend

Supplementary Figure S1. Distribution map of *Ctenocephalides felis* by department according to the number of references. 4

Supplementary Figure S2. Distribution map of *Haematopinus eurysternus* (1), *Linognathus vituli* (2) and *Solenopotes capillatus* (3) by department according to the number of references. 5

Supplementary Figure S3. Distribution map of *Atylotus agrestis* (1), *Atylotus flavoguttatus* (2), *Atylotus fulvus* (3), *Atylotus intermedius* (4), *Atylotus latistriatus* (5), *Atylotus loewianus* (6), *Atylotus plebeius* (7), *Atylotus quadrifarius* (8), *Atylotus rusticus* (9), *Chrysops caecutiens* (10), *Chrysops flavipes* (11), *Chrysops italicus* (12), *Chrysops parallelogrammus* (13), *Chrysops pictus* (14), *Chrysops relictus* (15), *Chrysops rufipes* (16), *Chrysops sepulcralis* (17), *Chrysops viduatus* (18), *Dasyrhamphis anthracinus* (19), *Dasyrhamphis ater* (20), *Haematopota bigoti* (21), *Haematopota crassicornis* (22), *Haematopota grandis* (23), *Haematopota italica* (24), *Haematopota lambi* (25), *Haematopota ocelligera* (26), *Haematopota pluvialis* (27), *Haematopota scutellata* (28), *Heptatoma pellucens* (29), *Hybomitra acuminata* (30), *Hybomitra aterrima* (31), *Hybomitra auripila* (32), *Hybomitra bimaculata* (33), *Hybomitra borealis* (34), *Hybomitra caucasica* (35), *Hybomitra ciureai* (36), *Hybomitra distinguenda* (37), *Hybomitra erberi* (38), *Hybomitra expollicata* (39), *Hybomitra lundbecki* (40), *Hybomitra lurida* (41), *Hybomitra micans* (42), *Hybomitra montana* (43), *Hybomitra muhlfeldi* (44), *Hybomitra olsufievina* (45), *Hybomitra solstitialis* (46), *Hybomitra tropica* (47), *Hybomitra vittata* (48), *Nemorius vitripennis* (49), *Pangonius haustellatus* (50), *Pangonius micans* (51), *Philipomyia aprica* (52), *Philipomyia graeca* (53), *Silvius algirus* (54), *Silvius alpinus* (55), *Silvius variegatus* (56), *Tabanus autumnalis* (57), *Tabanus bifarius* (58), *Tabanus bovinus* (59), *Tabanus briani* (60), *Tabanus bromius* (61), *Tabanus cordiger* (62), *Tabanus darimonti* (63), *Tabanus eggeri* (64), *Tabanus exclusus* (65), *Tabanus glaucopis* (66), *Tabanus lateralis* (67), *Tabanus lunatus* (68), *Tabanus maculicornis* (69), *Tabanus miki* (70), *Tabanus nemoralis* (71), *Tabanus paradoxus* (72), *Tabanus quatuornotatus* (73), *Tabanus rectus* (74), *Tabanus regularis* (75), *Tabanus rupium* (76), *Tabanus spectabilis* (77), *Tabanus spodopterus* (78), *Tabanus sudeticus* (79), *Tabanus tergestinus* (80), *Tabanus tinctus* (81), *Tabanus unifasciatus* (82) and *Therioplectes gigas* (83) by department according to the number of references. 27

Supplementary Figure S4. Distribution map of *Hippobosca equina* by department according to the number of references. 28

Supplementary Figure S5. Distribution map of *Haematobia irritans* (1), *Haematobia stimulans* (2) and *Stomoxys calcitrans* (3) by department according to the number of references. 29

Supplementary Figure S6. Distribution map of *Prosimulium hirtipes* (1), *Prosimulium latimucro* (2), *Prosimulium rufipes* (3), *Prosimulium tomosvaryi* (4), *Simulium angustipes* (5), *Simulium angustitarse* (6), *Simulium argenteostriatum* (7), *Simulium argyreatum* (8), *Simulium aureum* (9), *Simulium auricoma* (10), *Simulium bertrandi* (11), *Simulium bezzii* (12), *Simulium brevidens* (13), *Simulium carthusiense* (14), *Simulium costatum* (15), *Simulium cryophilum* (16), *Simulium equinum* (17), *Simulium erythrocephalum* (18), *Simulium intermedium* (19), *Simulium latigonium* (20), *Simulium latipes* (21), *Simulium lineatum* (22), *Simulium monticola* (23), *Simulium noelleri* (24), *Simulium ornatum* (25), *Simulium posticatum* (26), *Simulium pseudequinum* (27), *Simulium reptans* (28), *Simulium rheophilum* (29), *Simulium rubzovianum* (30), *Simulium trifasciatum* (31), *Simulium tuberosum* (32), *Simulium variegatum* (33), *Simulium vernum* (34) and *Simulium xanthinum* (35) by department according to the number of references. 39

Supplementary Figure S7. Distribution map of *Phlebotomus ariasi* (1), *Phlebotomus mascittii* (2), *Phlebotomus papatasi* (3), *Phlebotomus perfiliewi* (4), *Phlebotomus perniciosus* (5), *Phlebotomus sergenti* (6) and *Sergentomyia minuta* (7) by department according to the number of references. 41

Supplementary Figure S8. Distribution map of *Aedes aegypti* (1), *Aedes albopictus* (2), *Aedes annulipes* (3), *Aedes berlandi* (4), *Aedes cantans* (5), *Aedes caspius* (6), *Aedes cataphylla* (7), *Aedes cinereus* (8), *Aedes communis* (9), *Aedes detritus/coluzii* (10), *Aedes diantaeus* (11), *Aedes dorsalis* (12), *Aedes echinus* (13), *Aedes excrucians* (14), *Aedes flavescens* (15), *Aedes geminus* (16), *Aedes geniculatus* (17), *Aedes japonicus* (18), *Aedes mariae* (19), *Aedes nigrinus* (20), *Aedes nigripes* (21), *Aedes pulcritarsis* (22), *Aedes pullatus* (23), *Aedes punctor* (24), *Aedes refiki* (25), *Aedes rusticus* (26), *Aedes sticticus* (27), *Aedes surcoufi* (28), *Aedes vexans* (29), *Aedes vittatus* (30), *Aedes rossicus* (31), *Anopheles algeriensis* (32), *Anopheles beklemishevi* (33), *Anopheles claviger* (34), *Anopheles hyrcanus* (35), *Anopheles maculipennis* s.l. (36), *Anopheles marteri* (37), *Anopheles petragnani* (38), *Anopheles plumbeus* (39), *Anopheles pseudopictus* (40), *Anopheles sinensis* (41), *Anopheles superpictus* (42), *Coquillettidia buxtoni* (43), *Coquillettidia richiardii* (44), *Culex apicalis* (45), *Culex brumpti* (46), *Culex hortensis* (47), *Culex impudicus* (48), *Culex laticinctus* (49), *Culex martinii* (50), *Culex mimeticus* (51), *Culex modestus* (52), *Culex pipiens* (53), *Culex territans* (54), *Culex theileri* (55), *Culex torrentium* (56), *Culex univittatus* (57), *Culiseta alaskaensis* (58), *Culiseta annulata* (59), *Culiseta fumipennis* (60), *Culiseta glaphyroptera* (61), *Culiseta litorea* (62), *Culiseta longiareolata* (63), *Culiseta morsitans* (64), *Culiseta subochrea* (65), *Orthopodomyia pulcripalpis* (66) and *Uranotaenia unguiculata* (67) by department according to the number of references. 59

Supplementary Figure S9. Distribution map of *Alluaudomyia needhami* (1A), *Bezzia flavicornis* (2A), *Bezzia pygmaea* (3A), *Culicoides abchazicus* (4A, 4B), *Culicoides accraensis* (5), *Culicoides alazanicus* (6A, 6B), *Culicoides albicans* (7), *Culicoides albipennis* (8), *Culicoides begueti* (9A, 9B), *Culicoides brunnicans* (10A, 10B), *Culicoides cameroni* (11A, 11B), *Culicoides cataneii / gejgelensis* (12A, 12B), *Culicoides caucoliberensis* (13A), *Culicoides chiopterus* (14A, 14B),

Culicoides clintoni (15A), *Culicoides comosioculatus* (16A), *Culicoides corsicus* (17A), *Culicoides deltus* (18A, 18B), *Culicoides derisor* (19A), *Culicoides dewulfi* (20A, 20B), *Culicoides duddingstoni* (21A, 21B), *Culicoides dzhafarovi* (22A), *Culicoides furcillatus* (23A, 23B), *Culicoides gornostaevae* (24A), *Culicoides griseidorsum* (25A, 25B), *Culicoides grisescens* (26A, 26B), *Culicoides haranti* (27A, 27B), *Culicoides heliophilus* (28A, 28B), *Culicoides heteroclitus* (29A, 29B), *Culicoides ibericus* (30A), *Culicoides imicola* (31A, 31B), *Culicoides impunctatus* (32A, 32B), *Culicoides indistinctus* (33A, 33B), *Culicoides jumineri* (34A, 34B), *Culicoides jurensis* (35A), *Culicoides kibunensis* (36A, 36B), *Culicoides kurensis* (37A, 37B), *Culicoides longipennis* (38A, 38B), *Culicoides malevillei* (39A), *Culicoides manchuriensis* (40A, 40B), *Culicoides maritimus* (41A, 41B), *Culicoides minutissimus* (42A, 42B), *Culicoides montanus* (43A), *Culicoides odiatus* (44A, 44B), *Culicoides paradisionensis* (45A, 45B), *Culicoides paradoxalis* (46A), *Culicoides parroti* (47A, 47B), *Culicoides pictipennis* (48A, 48B), *Culicoides poperinghensis* (49A, 49B), *Culicoides pseudoheliophilus* (50A), *Culicoides pseudopallidus* (51A), *Culicoides pumilus* (52A), *Culicoides reconditus* (53A, 53B), *Culicoides riebi* (54A), *Culicoides riouxi* (55A, 55B), *Culicoides saevus* (56A, 56B), *Culicoides sahariensis* (57A), *Culicoides salinarius* (58A, 58B), *Culicoides santonicus* (59A, 59B), *Culicoides segnis* (60A, 60B), *Culicoides semimaculatus* (61A, 61B), *Culicoides sergenti* (62A), *Culicoides shaklawensis* (63A, 63B), *Culicoides simulator* (64A, 64B), *Culicoides stigma* (65A, 65B), *Culicoides tauricus* (66A, 66B), *Culicoides tbilisicus* (67A), *Culicoides truncorum* (68A), *Culicoides univittatus* (69A, 69B), *Culicoides vexans* (70A, 70B), *Culicoides vidourlensis* (71A), Achrayi Group (72A, 72B), Circumscriptus Group (73A, 73B), Fagineus Group (74A, 74B), Festivipennis Group (75A, 75B), Nubeculosus Group (76A, 76B), Obsoletus Group (77A, 77B), Pulicaris Group (78A, 78B), Punctatus Group (79A, 79B) and *Palpomyia lineata* (80A) by department according to the number of references (A) and, if available, the percentage of capture (B). 93

Supplementary Figure S10. Distribution map of *Amblyomma variegatum* (1), *Argas reflexus* (2), *Argas vespertilionis* (3), *Dermacentor marginatus* (4), *Dermacentor reticulatus* (5), *Haemaphysalis concinna* (6), *Haemaphysalis inermis* (7), *Haemaphysalis punctata* (8), *Haemaphysalis sulcata* (9), *Hyalomma aegyptium* (10), *Hyalomma detritum* (11), *Hyalomma excavatum* (12), *Hyalomma lusitanicum* (13), *Hyalomma marginatum* (14), *Hyalomma scupense* (15), *Ixodes acuminatus* (16), *Ixodes canisuga* (17), *Ixodes festai* (18), *Ixodes frontalis* (19), *Ixodes hexagonus* (20), *Ixodes ricinus* (21), *Ixodes trianguliceps* (22), *Ixodes ventalloi* (23), *Ixodes vespertilionis* (24), *Ornithodoros coniceps* (25), *Rhipicephalus annulatus* (26), *Rhipicephalus bursa* (27), *Rhipicephalus pusillus* (28), *Rhipicephalus sanguineus* (29) and *Rhipicephalus turanicus* (30) by department according to the number of references. 101

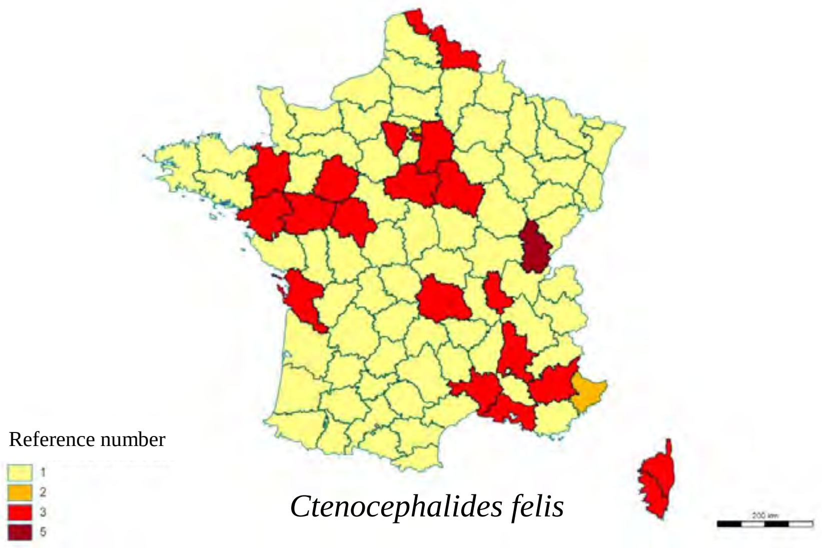

Supplementary Figure S1. Distribution map of *Ctenocephalides felis* by department according to the number of references.

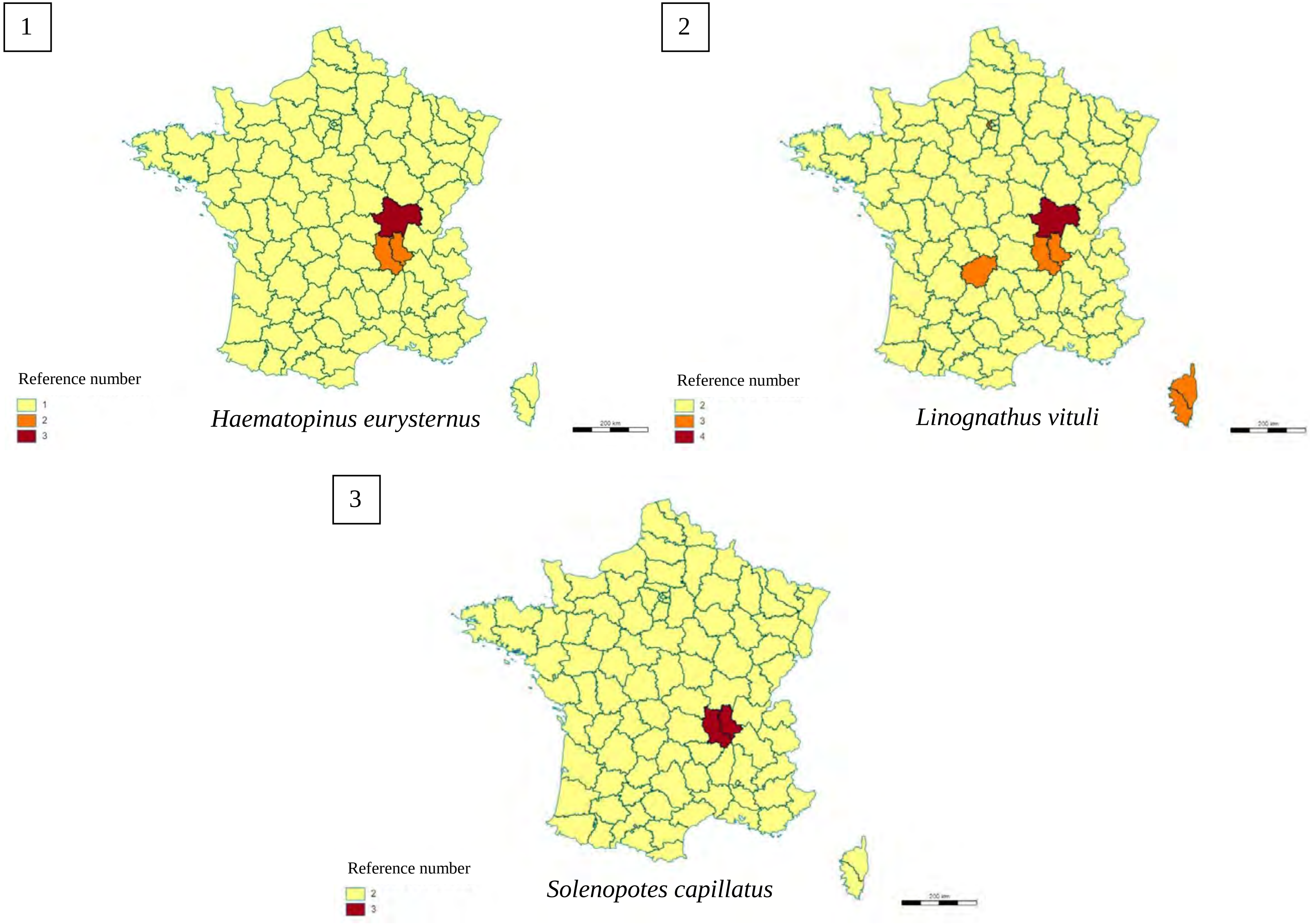

Supplementary Figure S2. Distribution map of *Haematopinus eurysternus* (1), *Linognathus vituli* (2) and *Solenopotes capillatus* (3) by department according to the number of references.

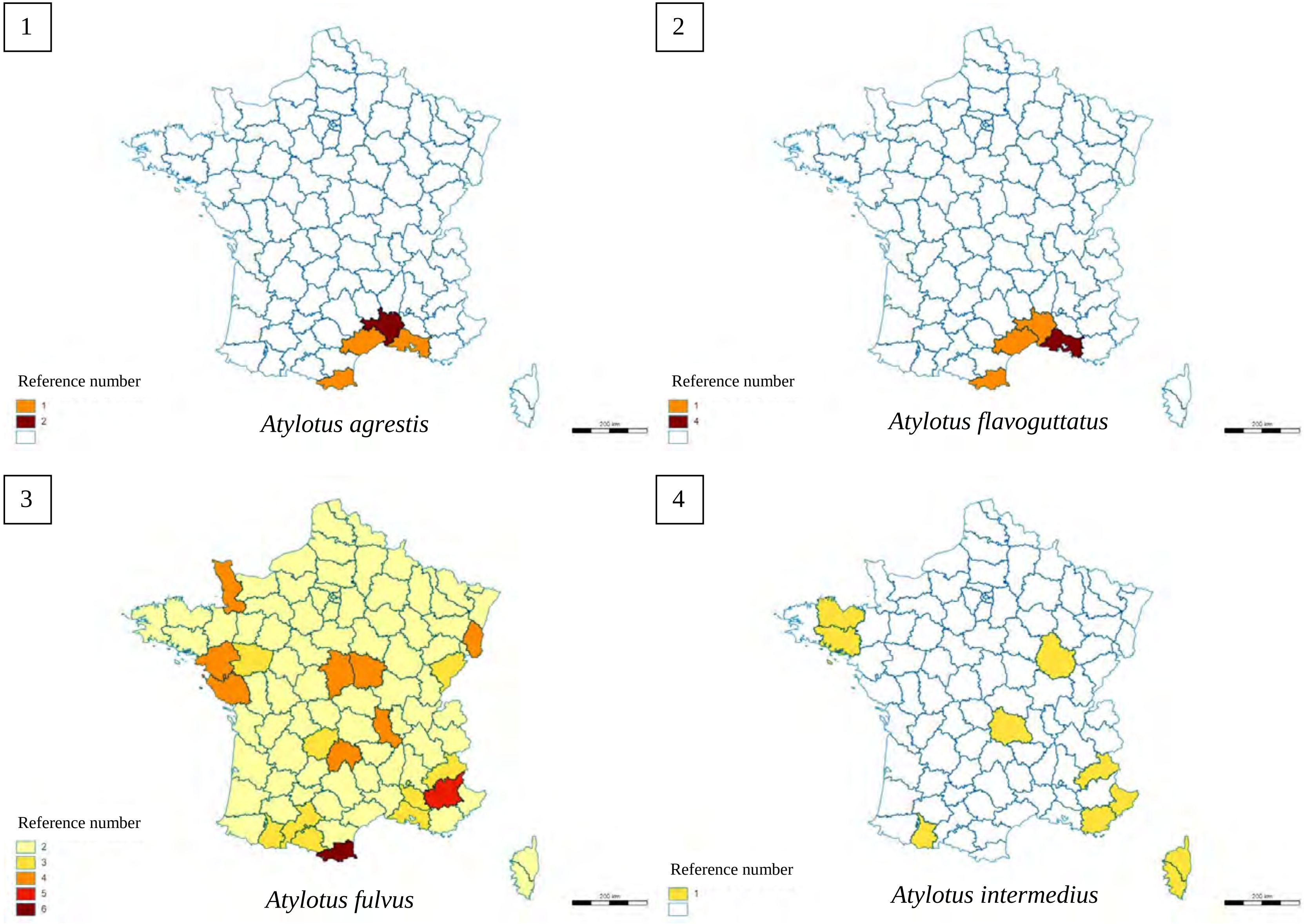
1
Reference number
1
2
Atylotus agrestis
2
Reference number
1
4
Atylotus flavoguttatus
3
Reference number
2
3
4
5
6
Atylotus fulvus
4
Reference number
1
Atylotus intermedius

5

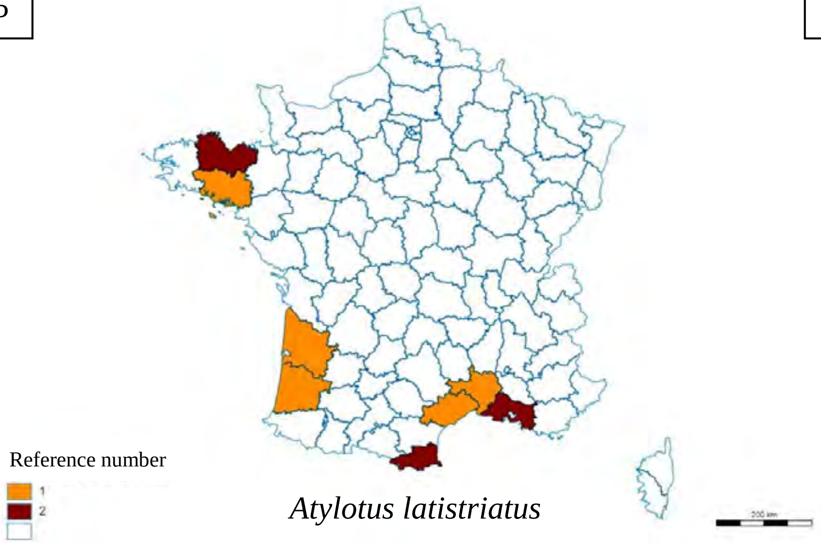

Atylotus latistriatus

6

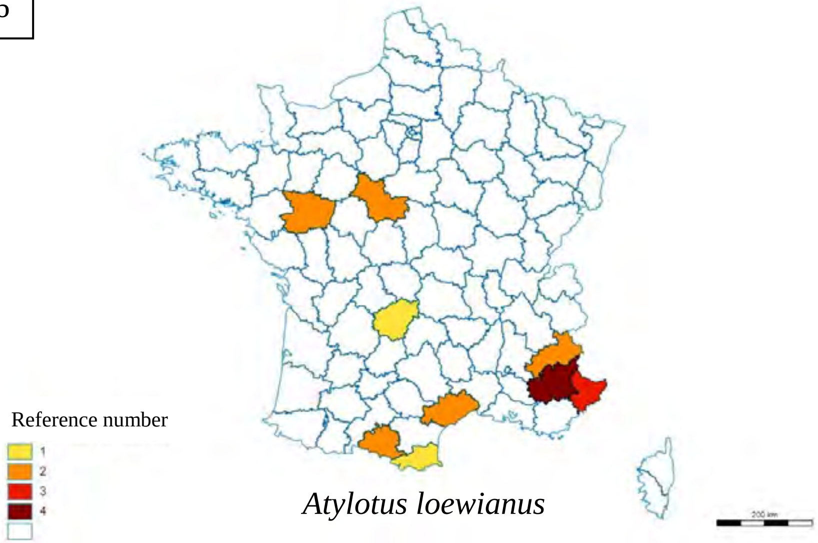

Atylotus loewianus

7

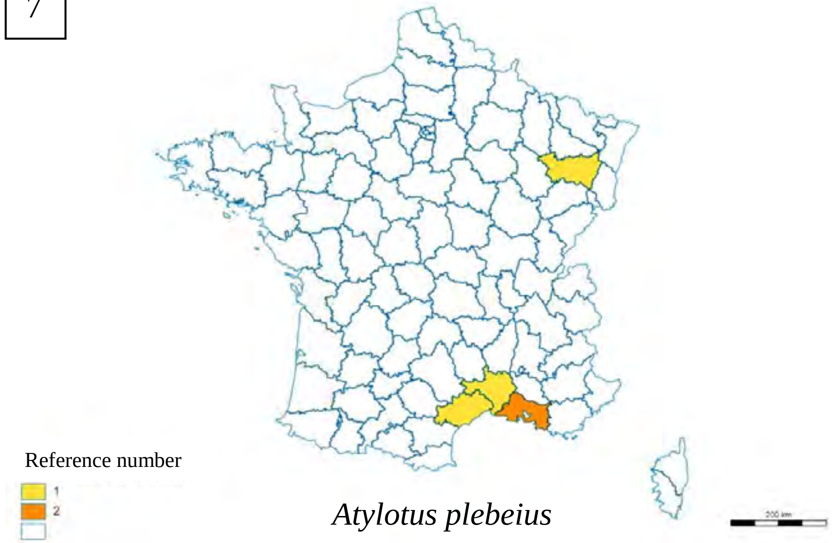

Atylotus plebeius

8

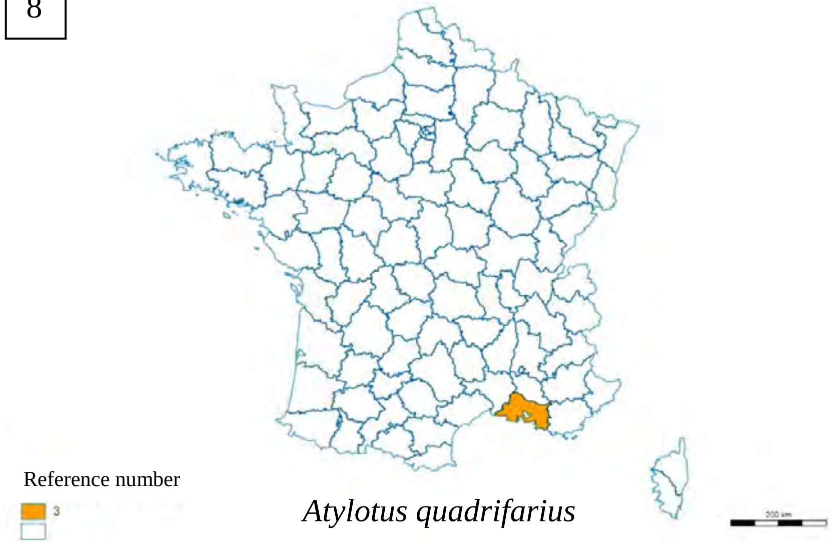

Atylotus quadrifarius

9

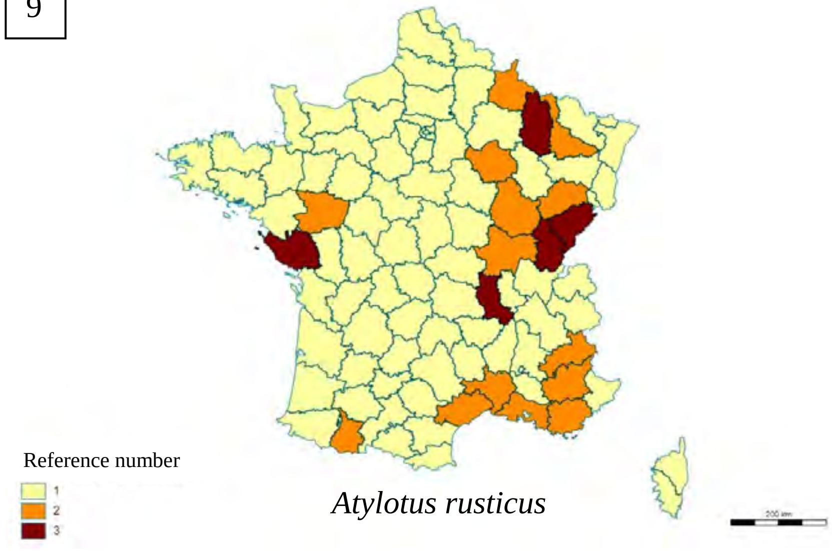

Atylotus rusticus

10

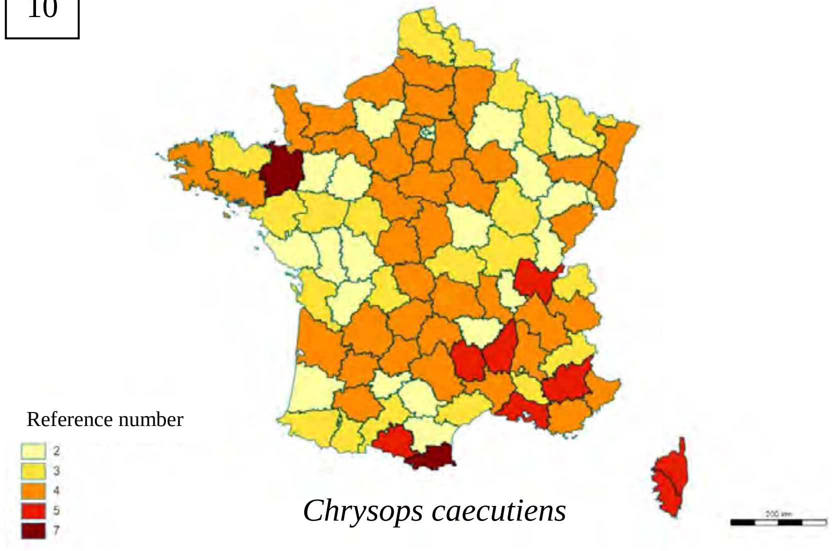

Chrysops caecutiens

11

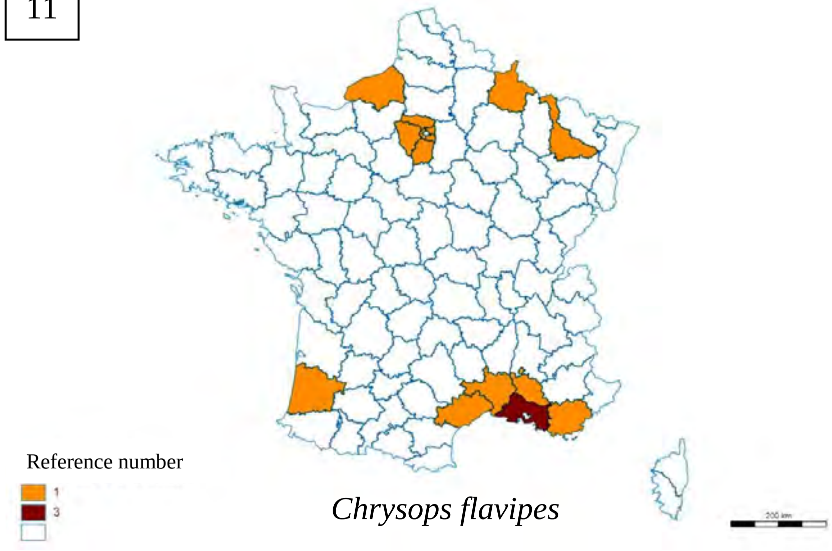

Chrysops flavipes

12

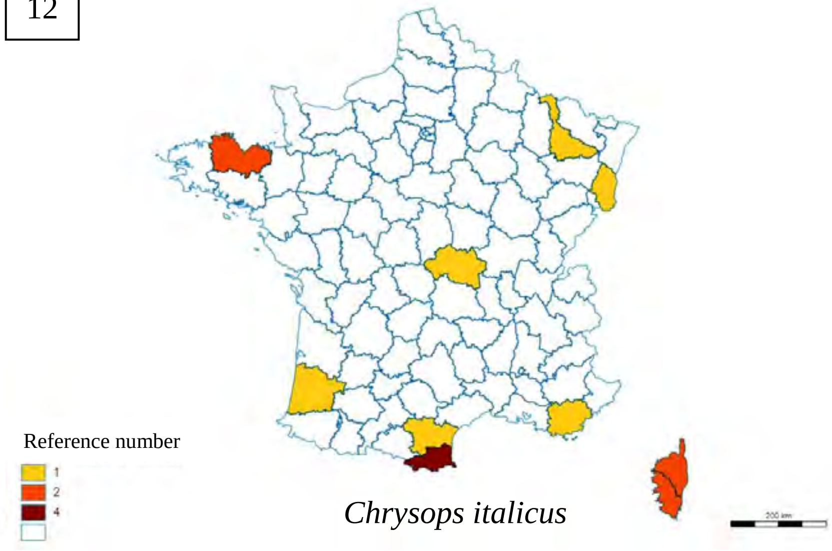

Chrysops italicus

13

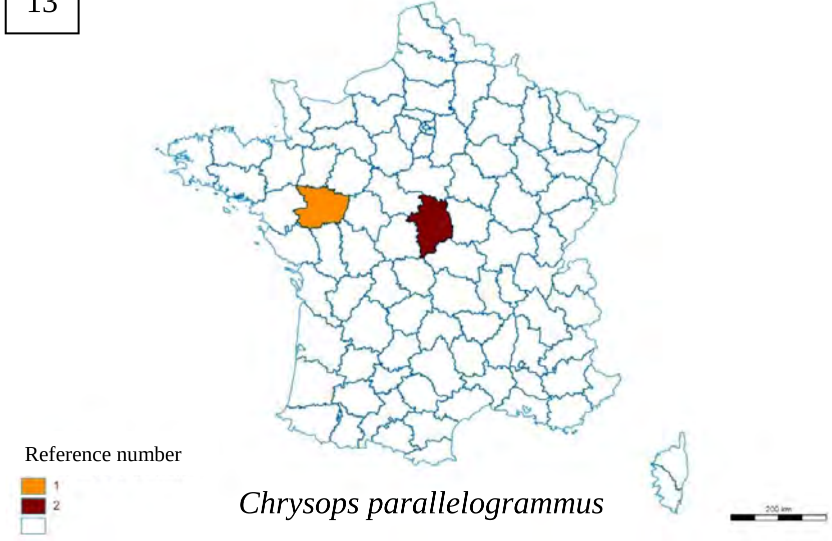

Chrysops parallelogrammus

14

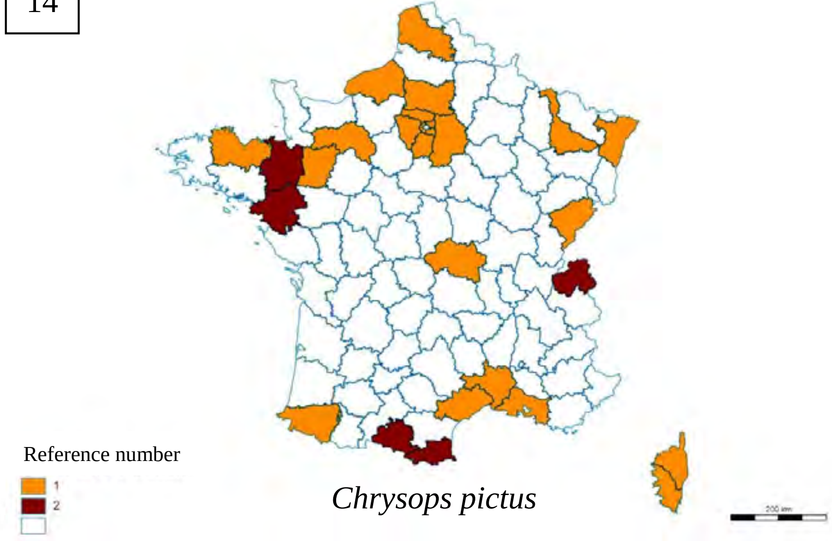

Chrysops pictus

15

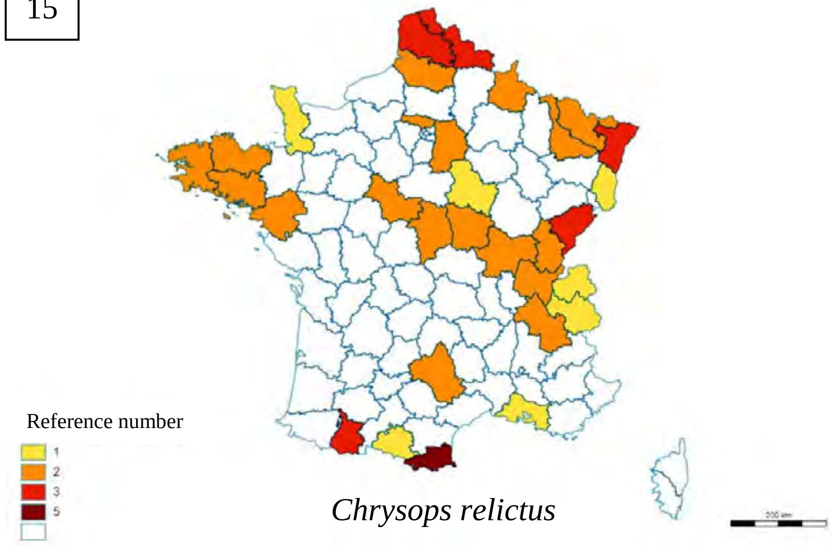

Chrysops relictus

16

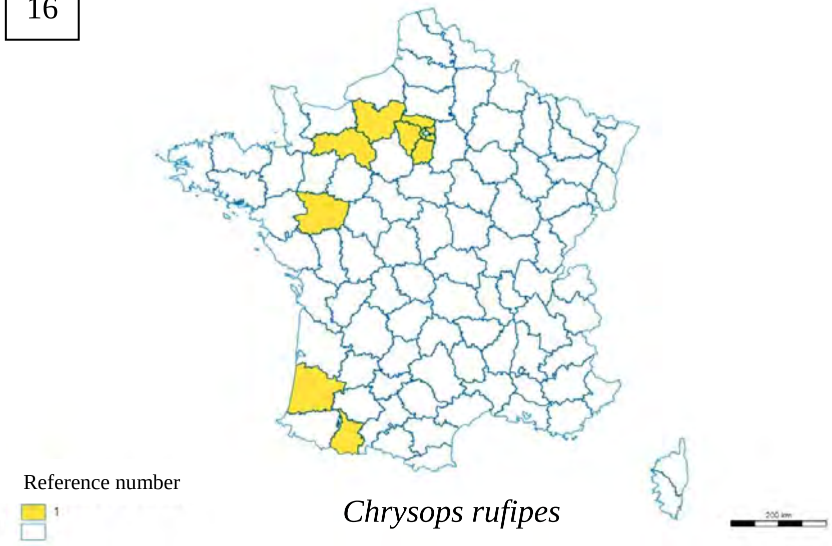

Chrysops rufipes

17

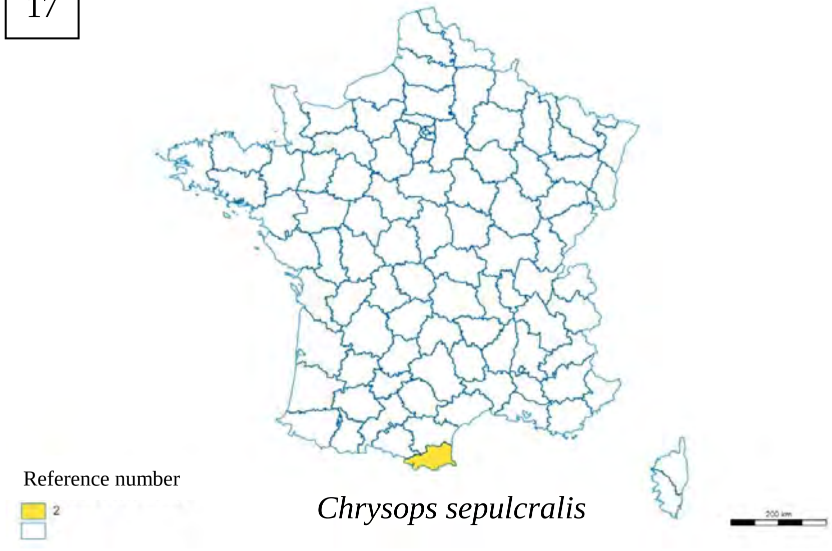
Reference number
2
Chrysops sepulcralis

18

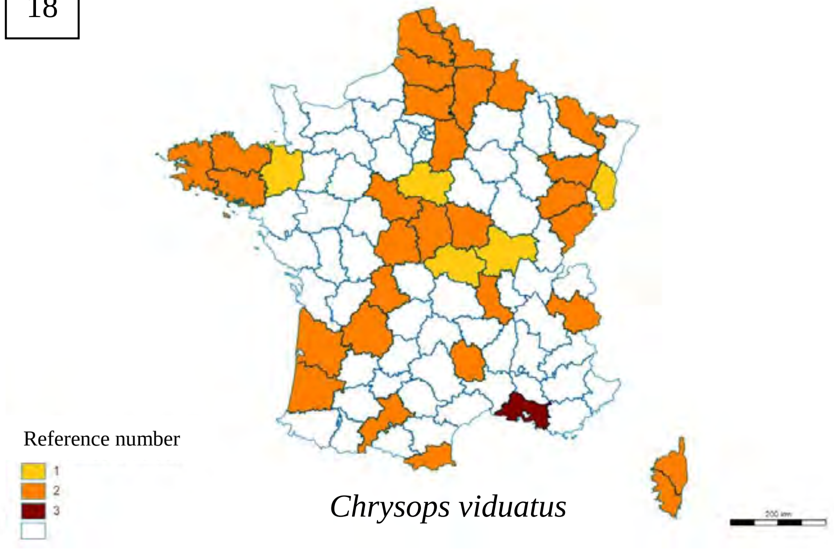
Reference number
1
2
3
Chrysops viduatus

19

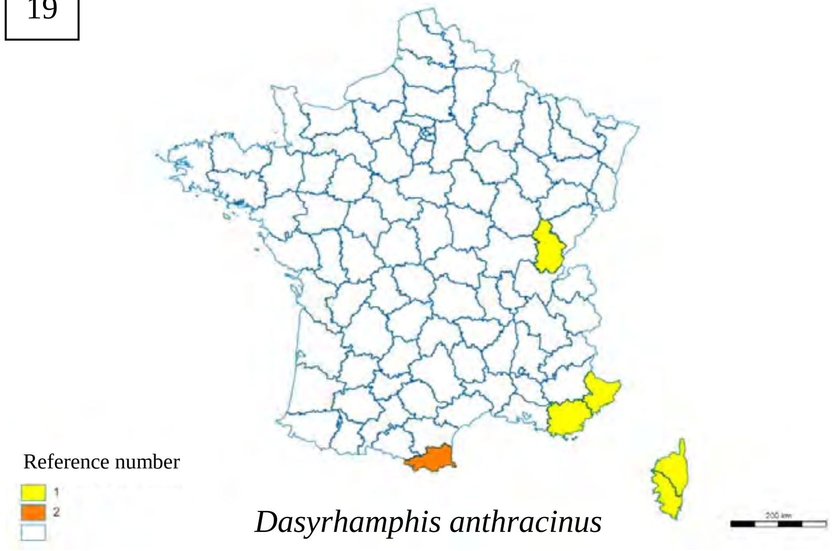
Reference number
1
2
Dasyrhamphis anthracinus

20

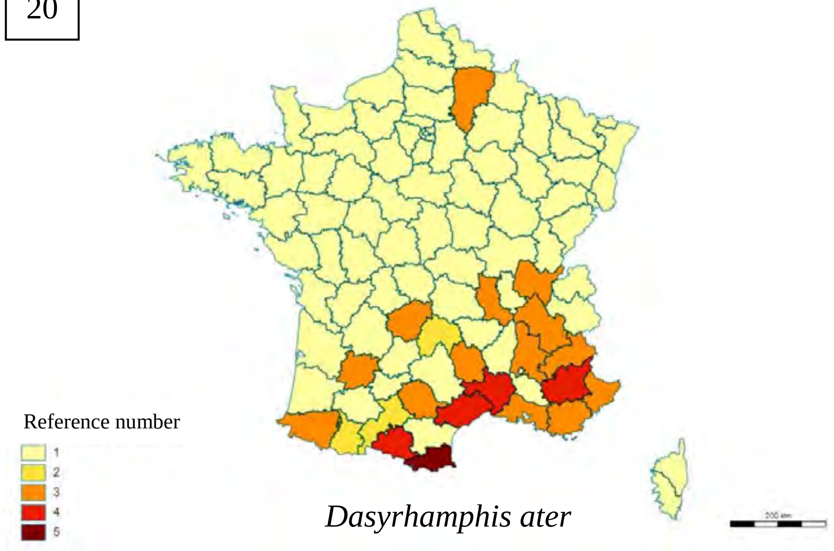
Reference number
1
2
3
4
5
Dasyrhamphis ater

21

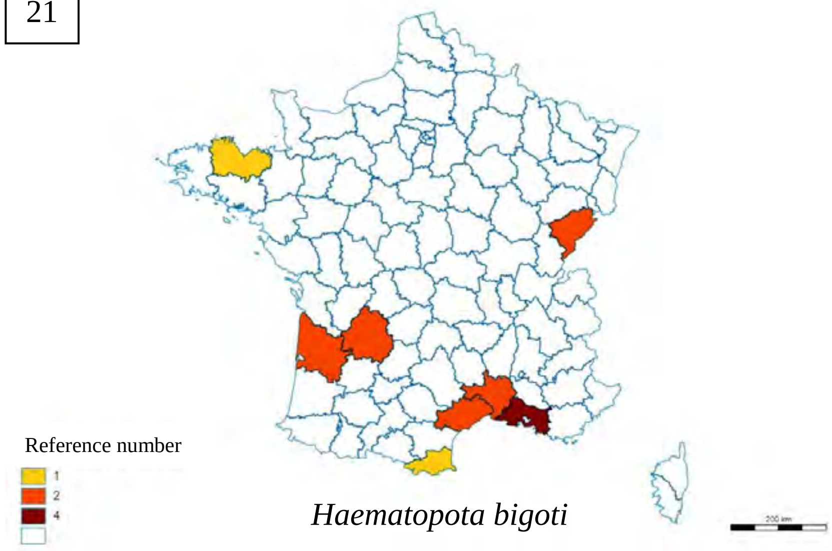

Haematopota bigoti

22

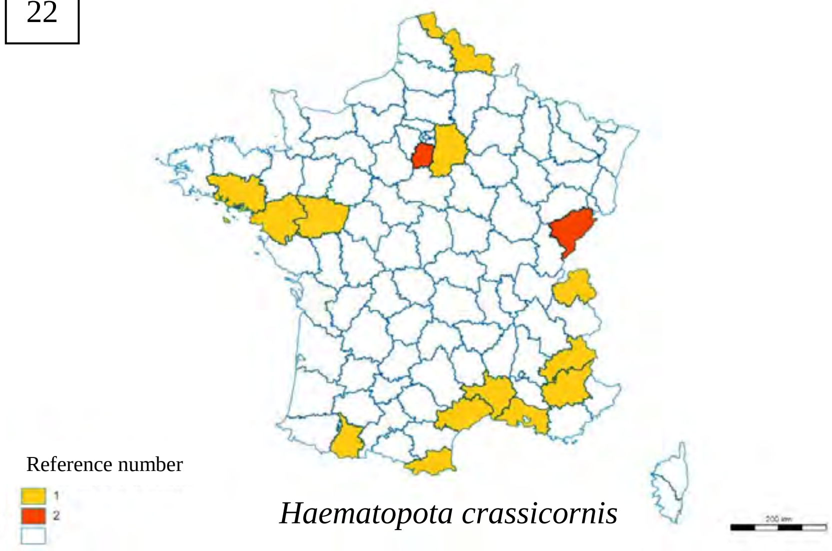

Haematopota crassicornis

23

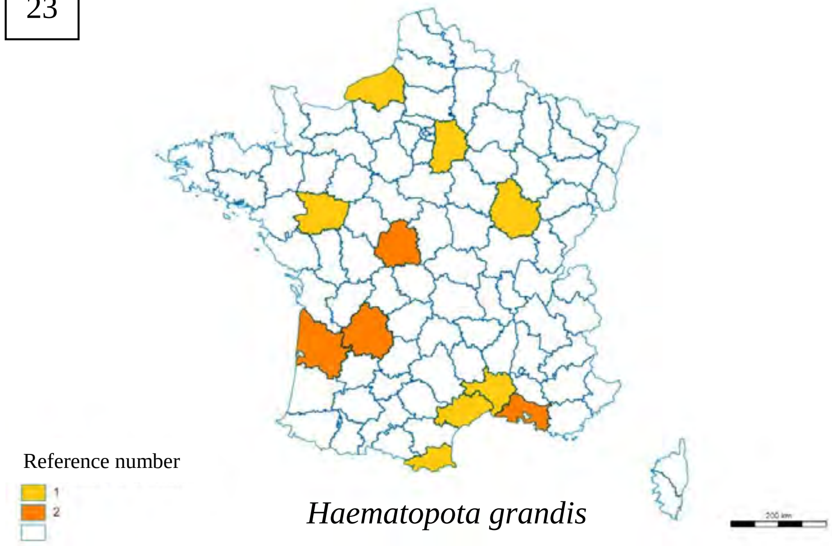

Haematopota grandis

24

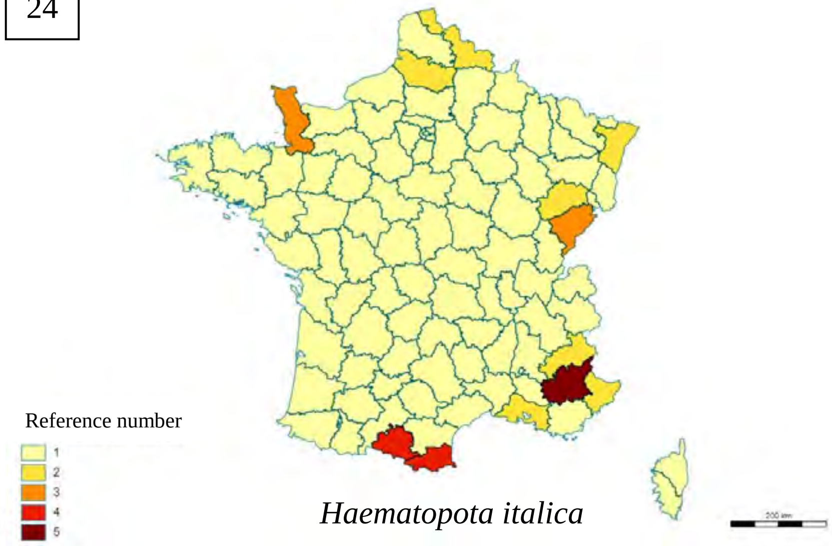

Haematopota italica

25

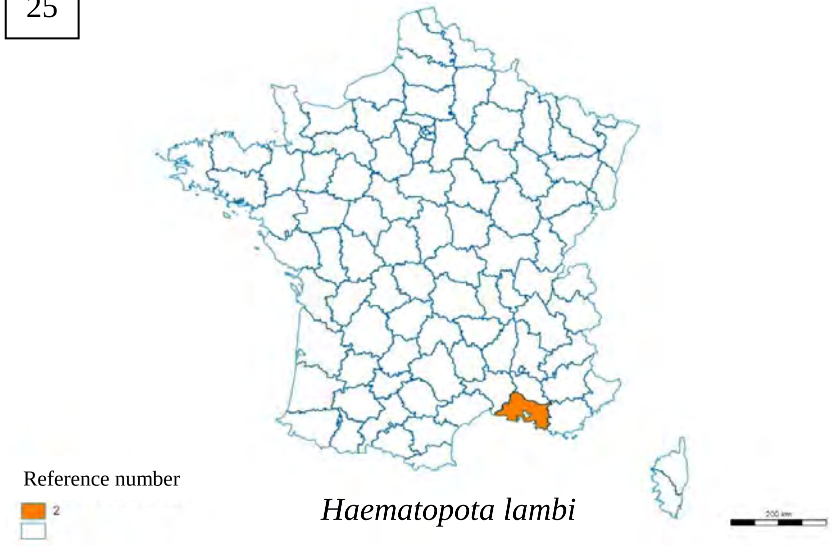

Haematopota lambi

26

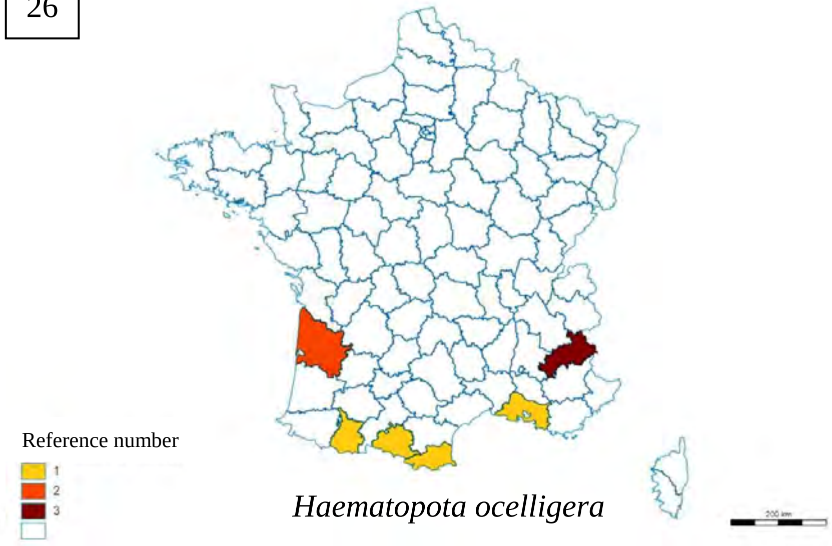

Haematopota ocelligera

27

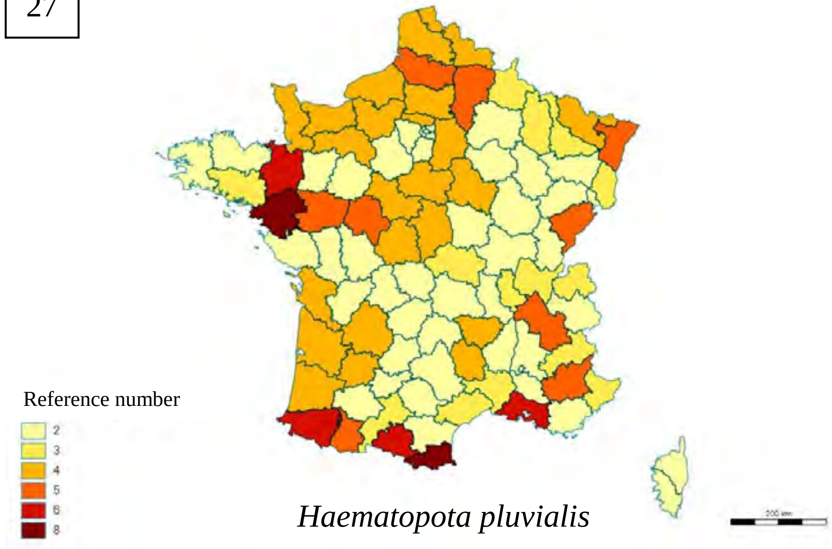

Haematopota pluvialis

28

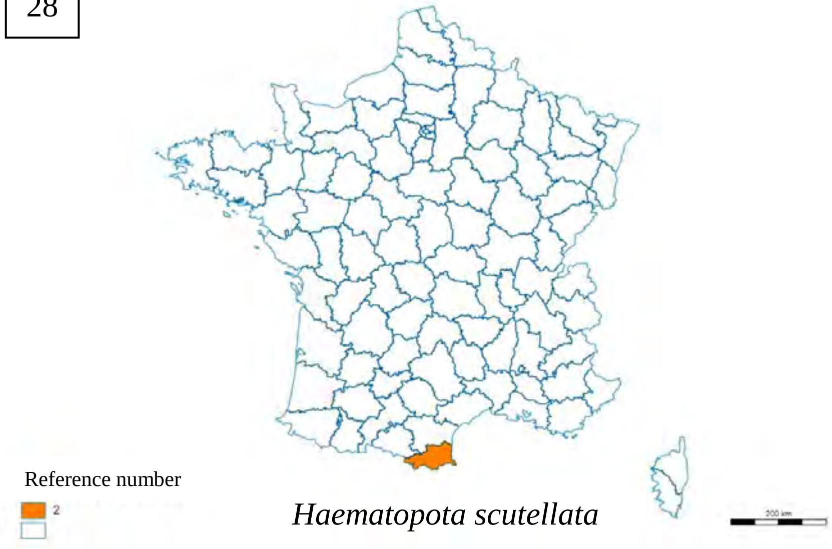

Haematopota scutellata

29

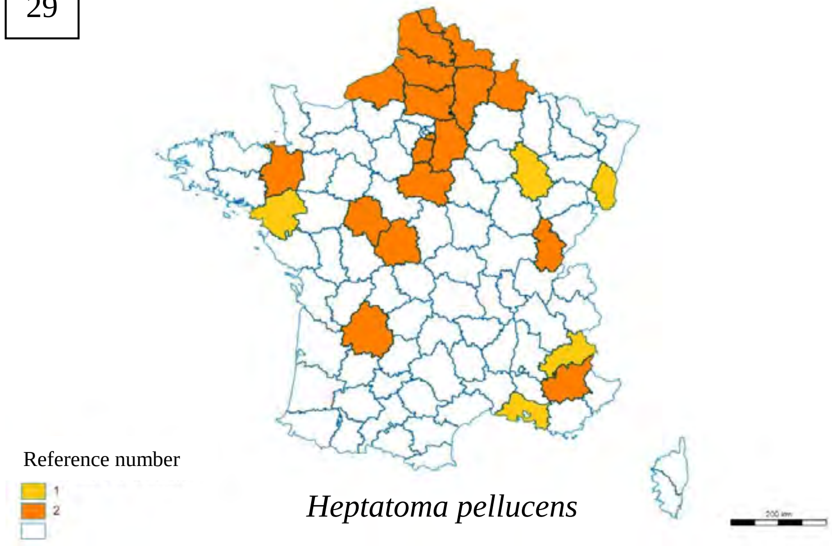

Heptatoma pellucens

30

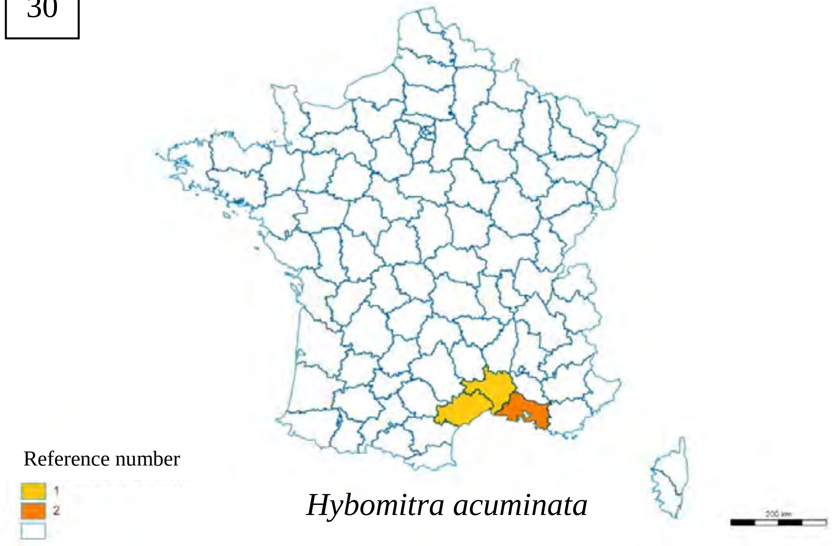

Hybomitra acuminata

31

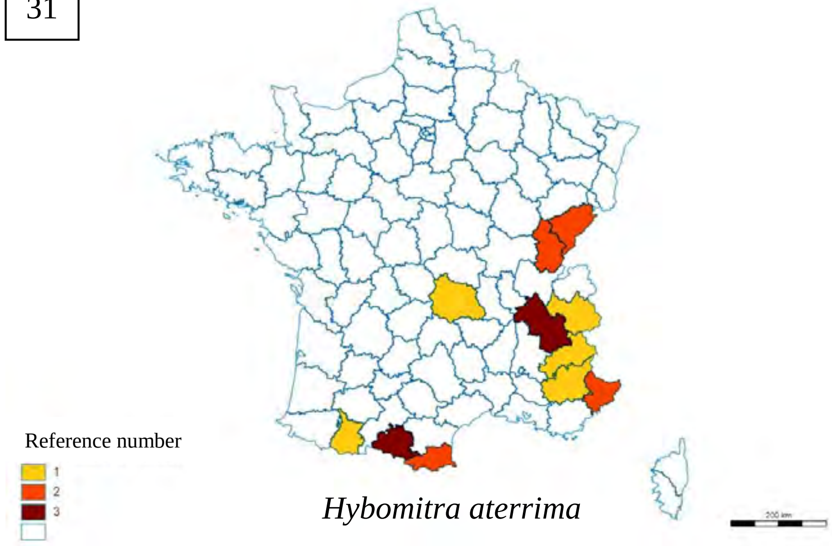

Hybomitra aterrima

32

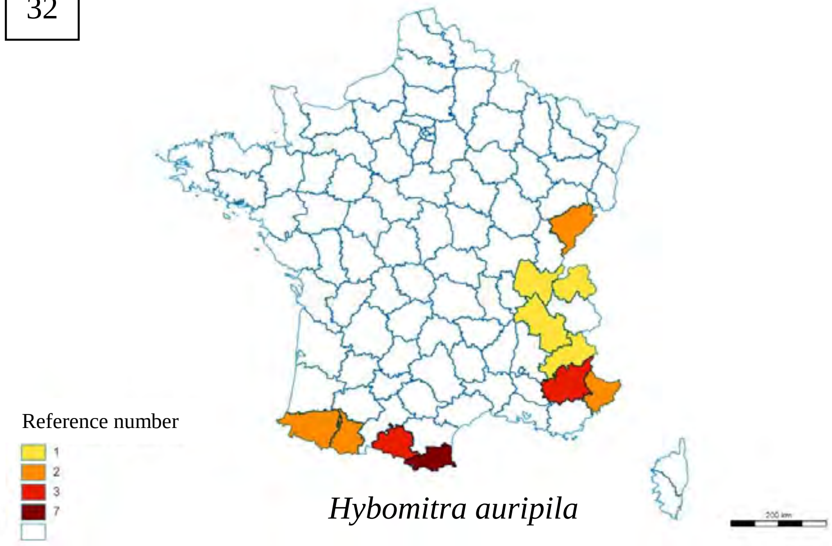

Hybomitra auripila

33

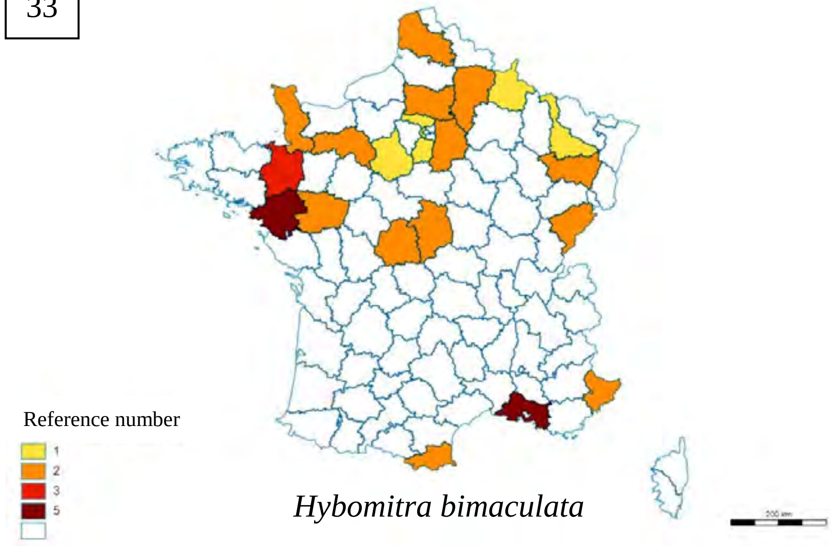

Hybomitra bimaculata

34

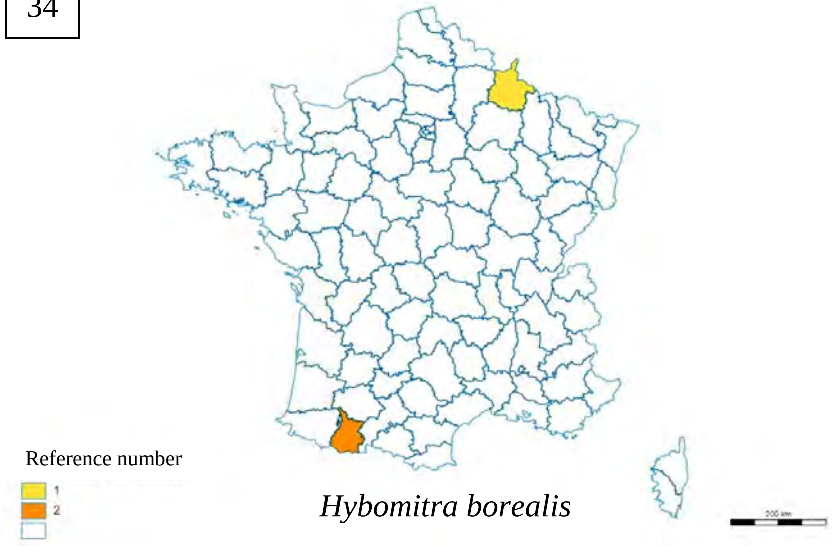

Hybomitra borealis

35

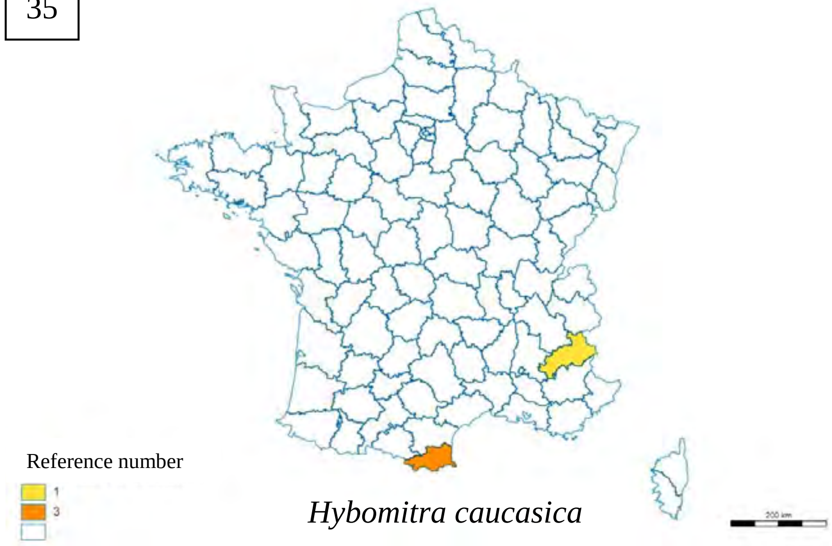

Hybomitra caucasica

36

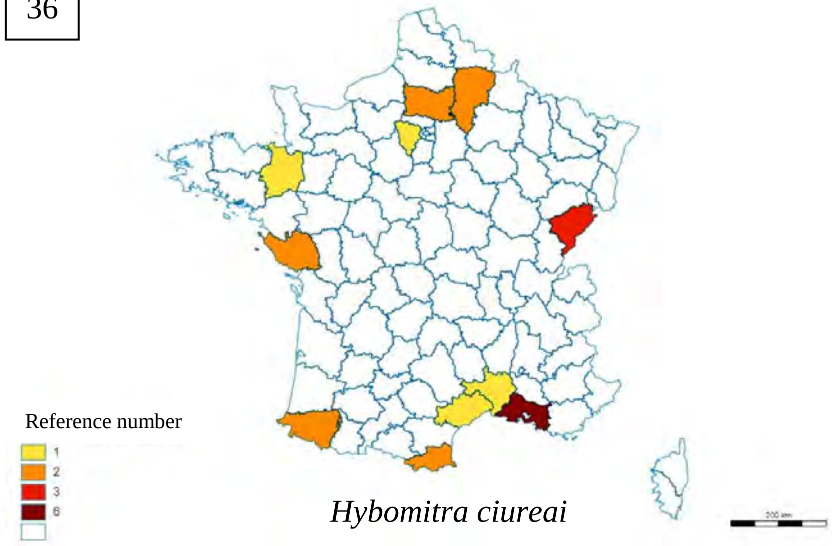

Hybomitra ciureai

37

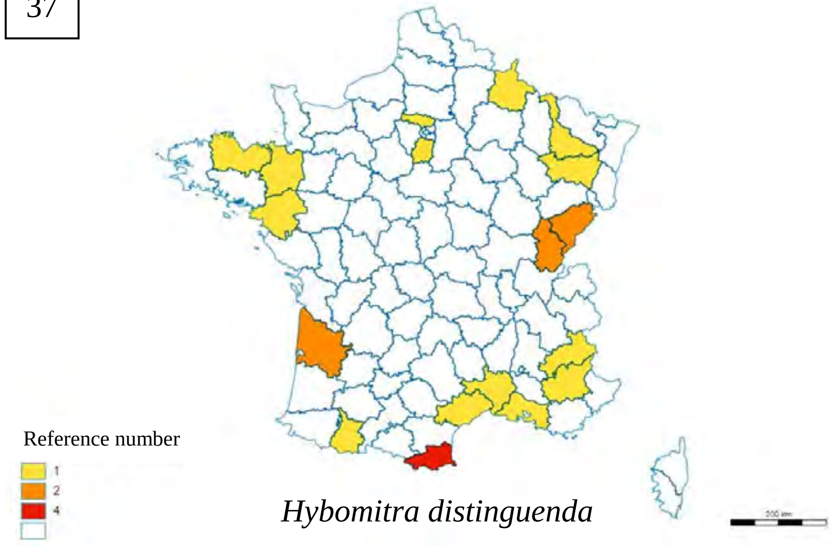

Hybomitra distinguenda

38

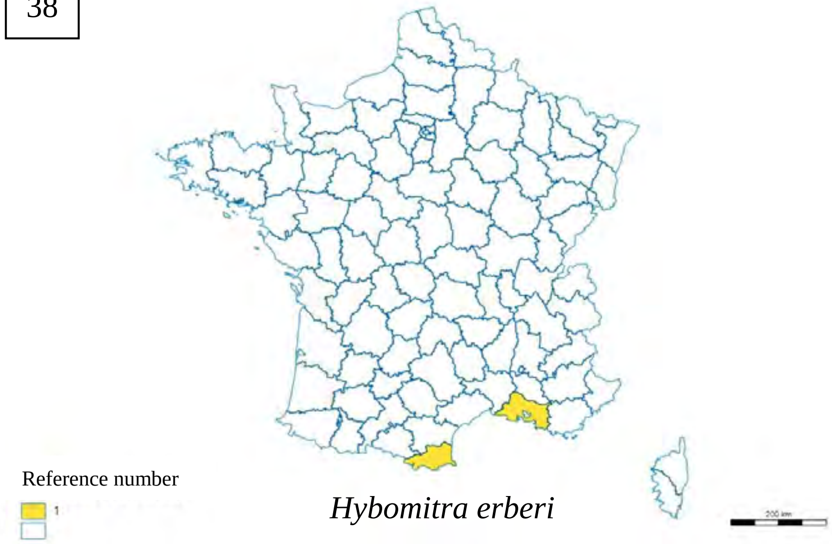

Hybomitra erberi

39

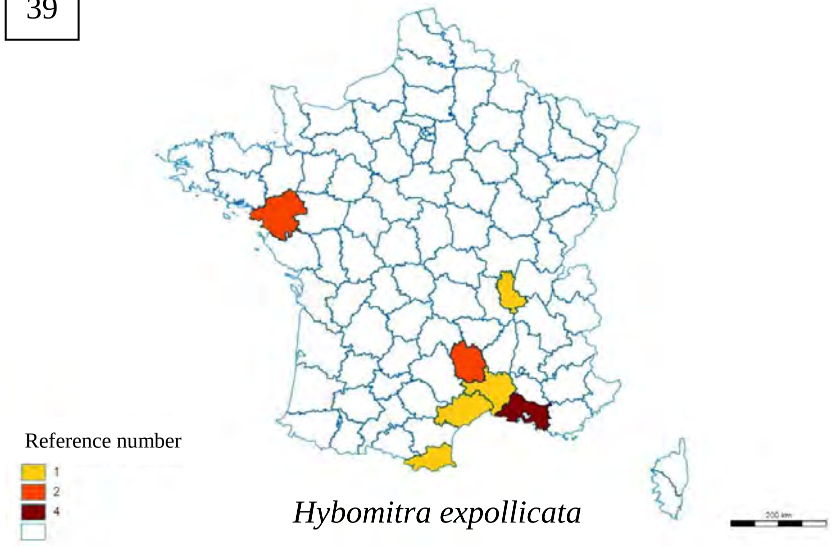

Hybomitra expollicata

40

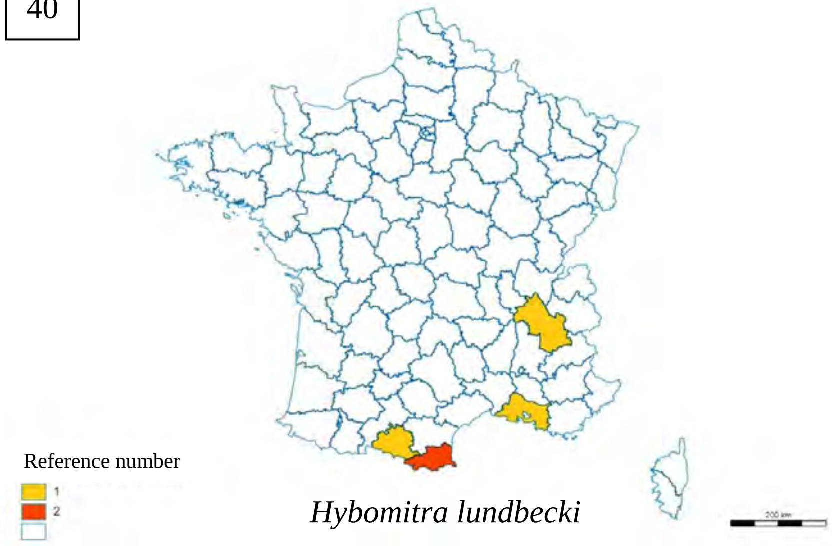

Hybomitra lundbecki

41

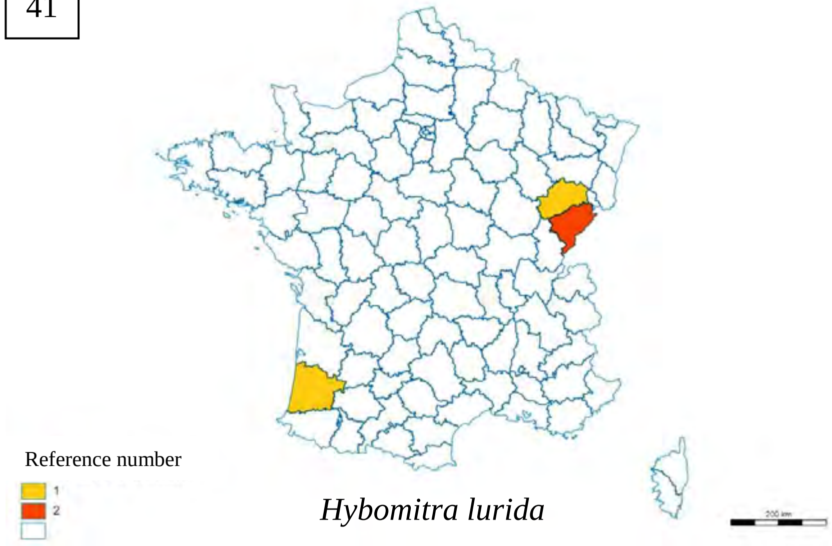
Reference number
1
2
Hybomitra lurida

42

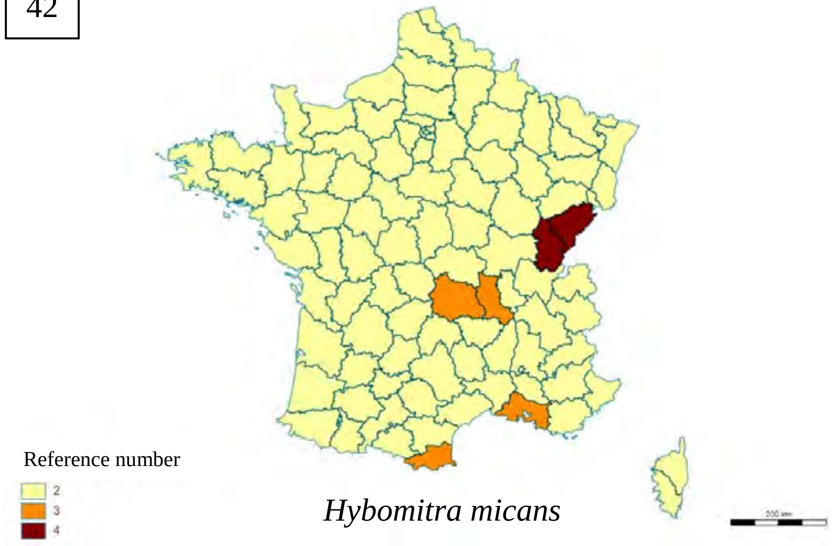
Reference number
2
3
4
Hybomitra micans

43

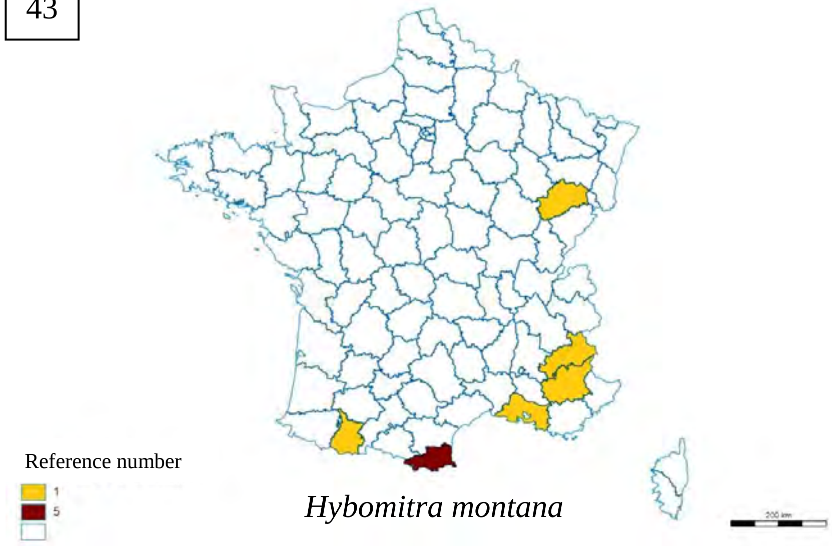
Reference number
1
5
Hybomitra montana

44

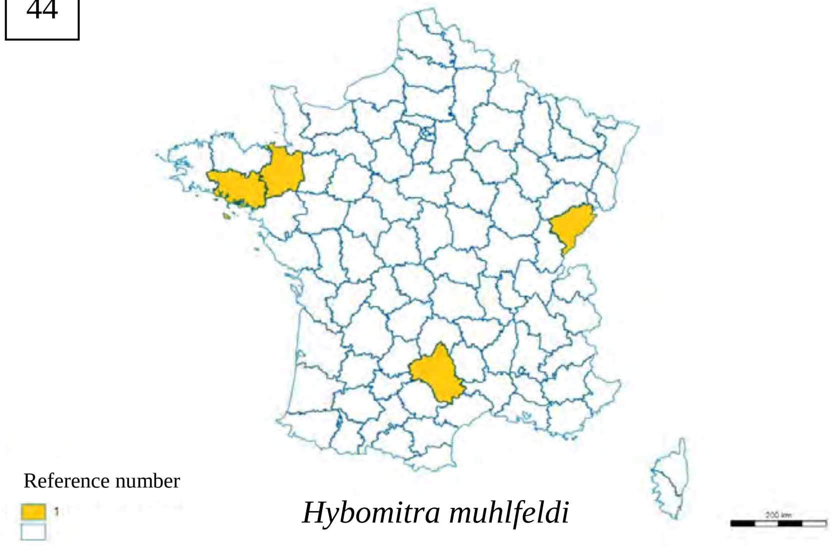
Reference number
1
Hybomitra muhlfeldi

45

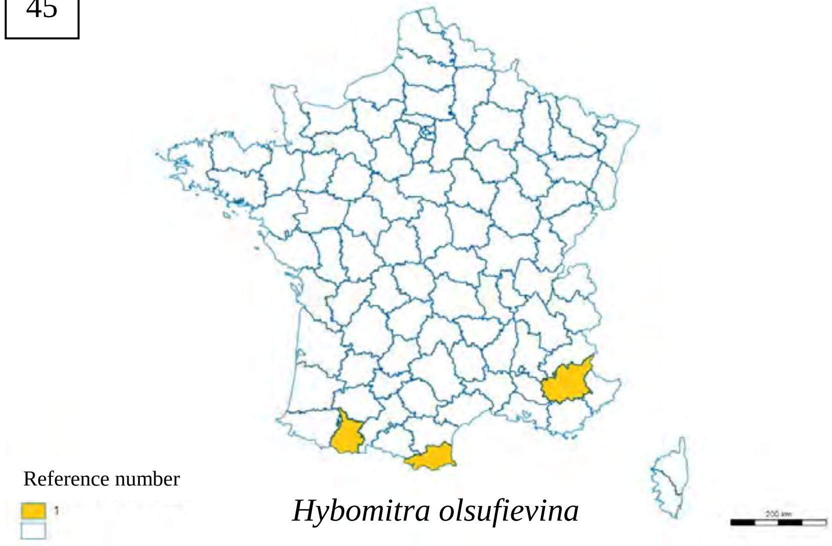

Hybomitra olsufievina

46

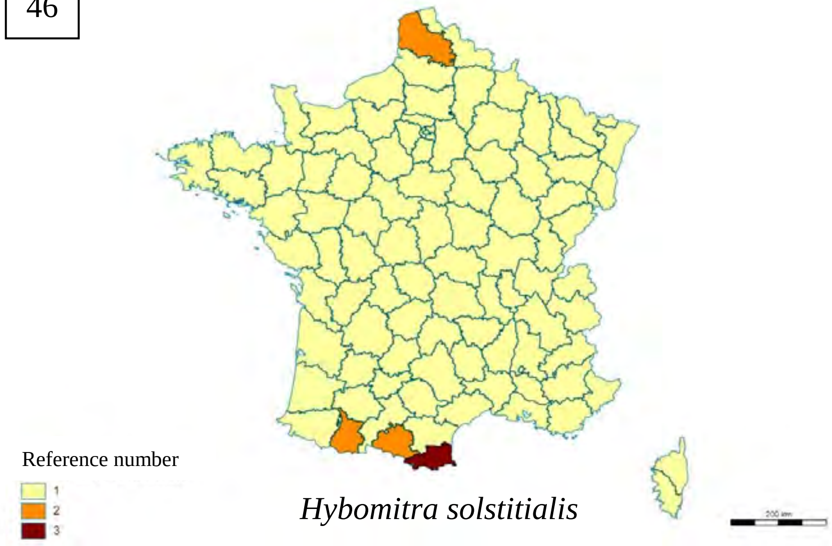

Hybomitra solstitialis

47

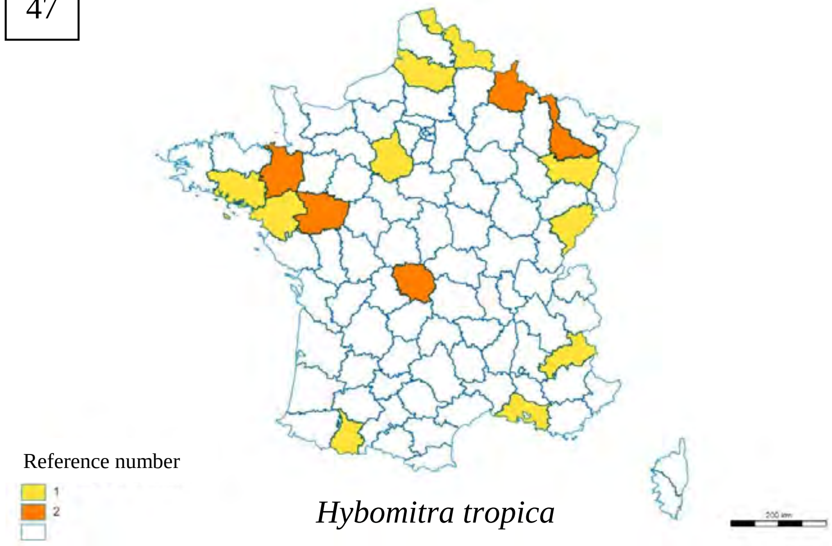

Hybomitra tropica

48

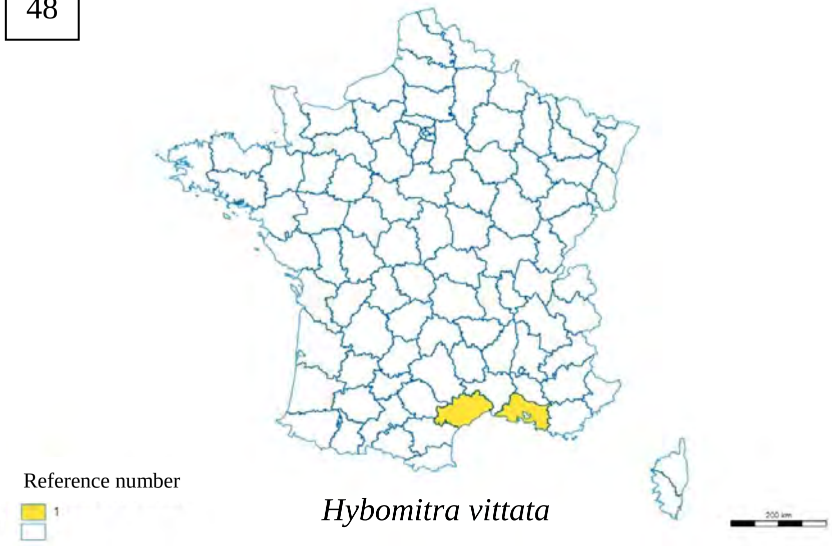

Hybomitra vittata

49

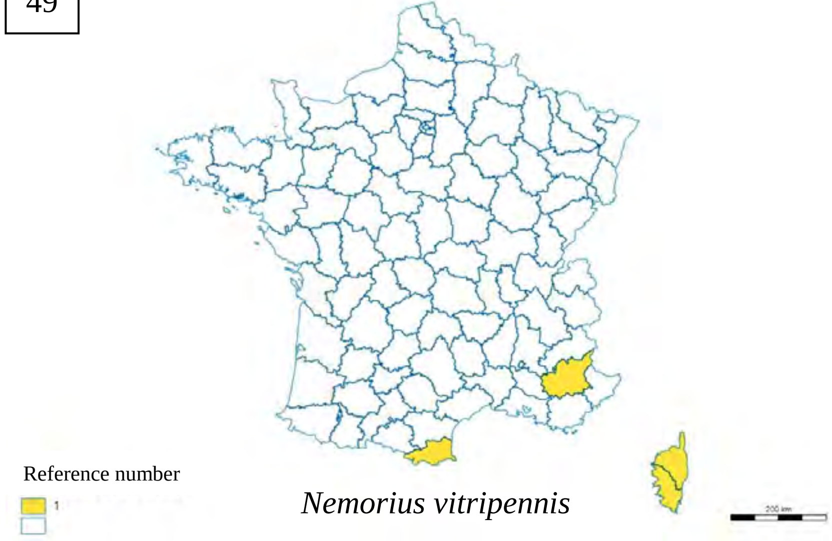
Reference number
1
Nemorius vitripennis

50

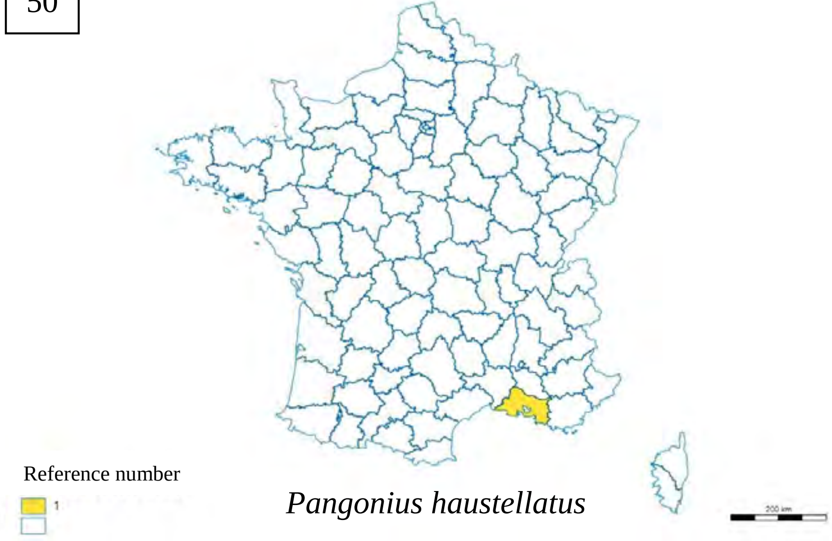
Reference number
1
Pangonius haustellatus

51

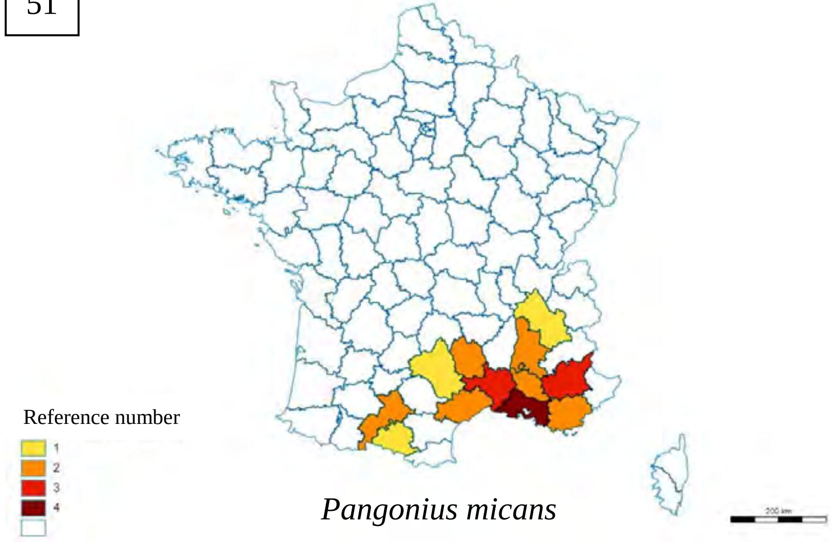
Reference number
1
2
3
4
Pangonius micans

52

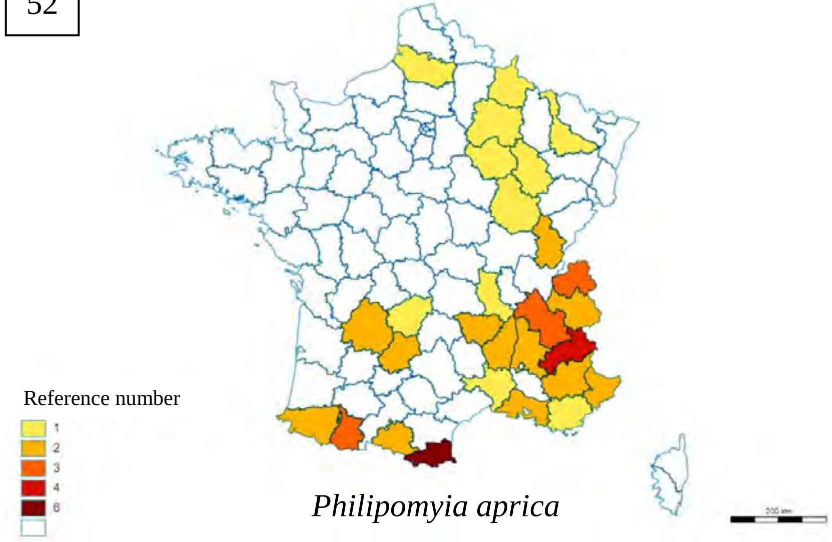
Reference number
1
2
3
4
6
Philipomyia aprica

53

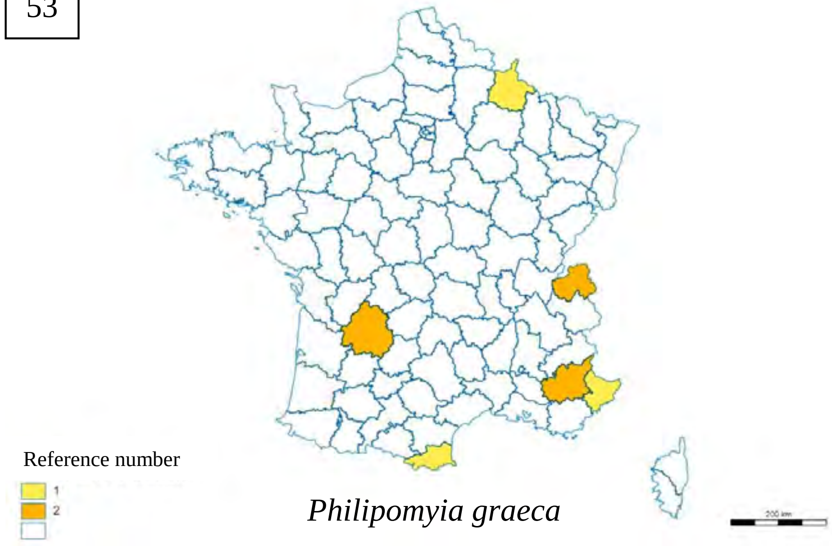

Philipomyia graeca

54

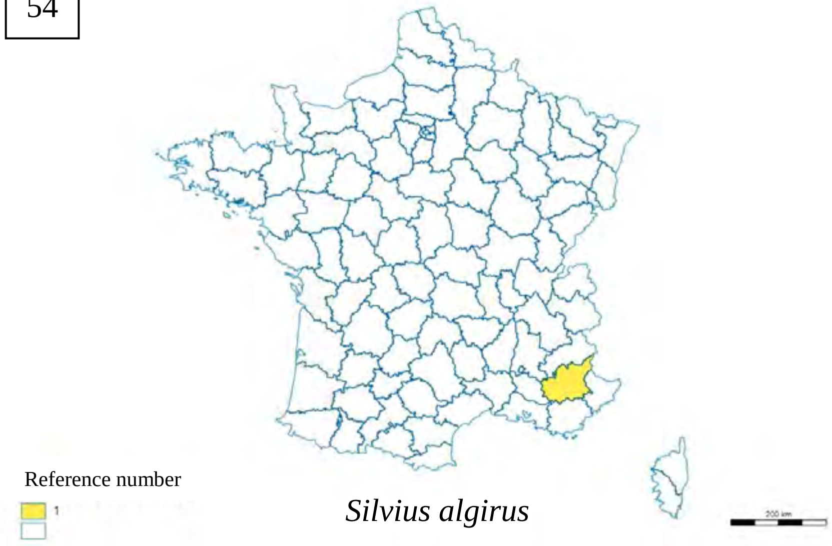

Silvius algirus

55

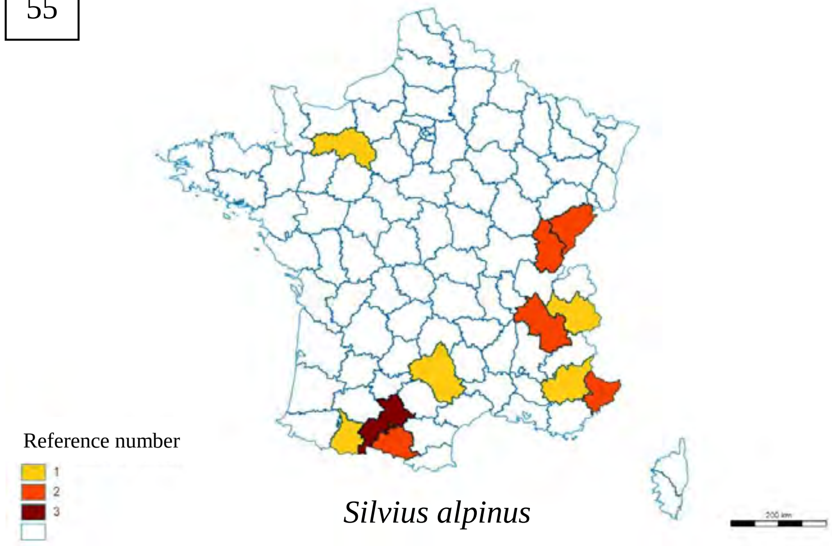

Silvius alpinus

56

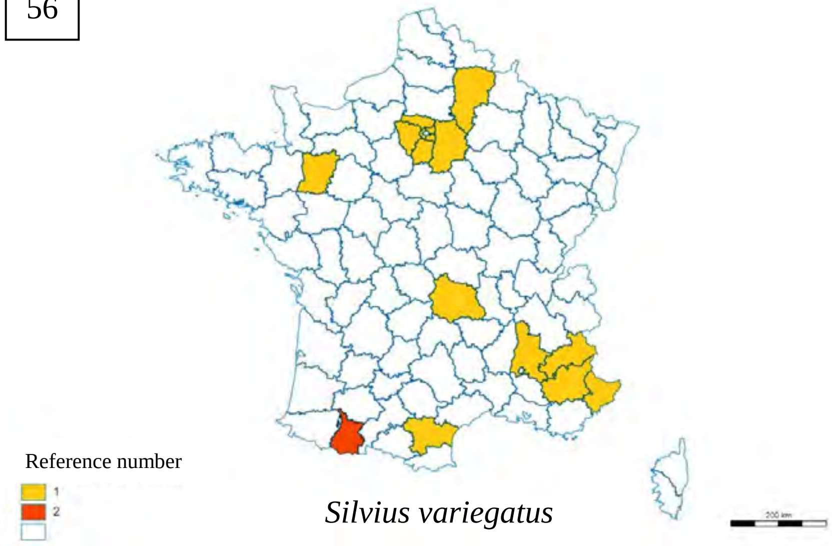

Silvius variegatus

57

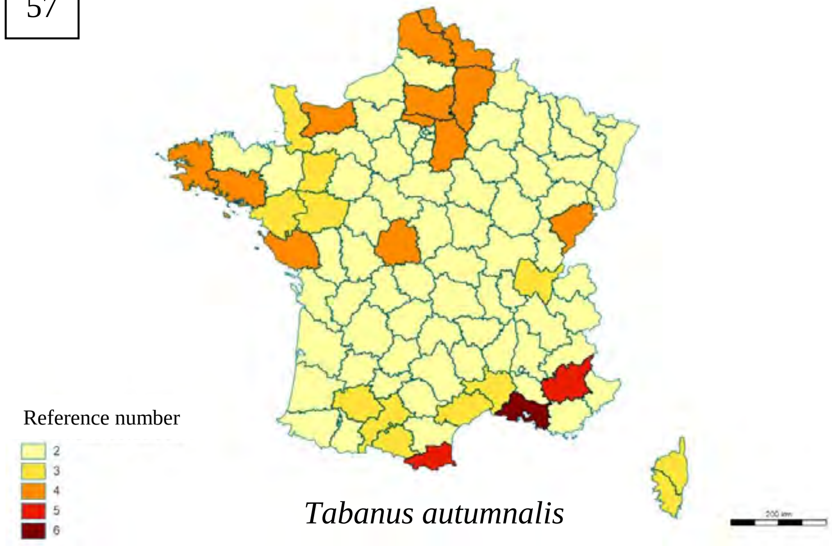

Tabanus autumnalis

58

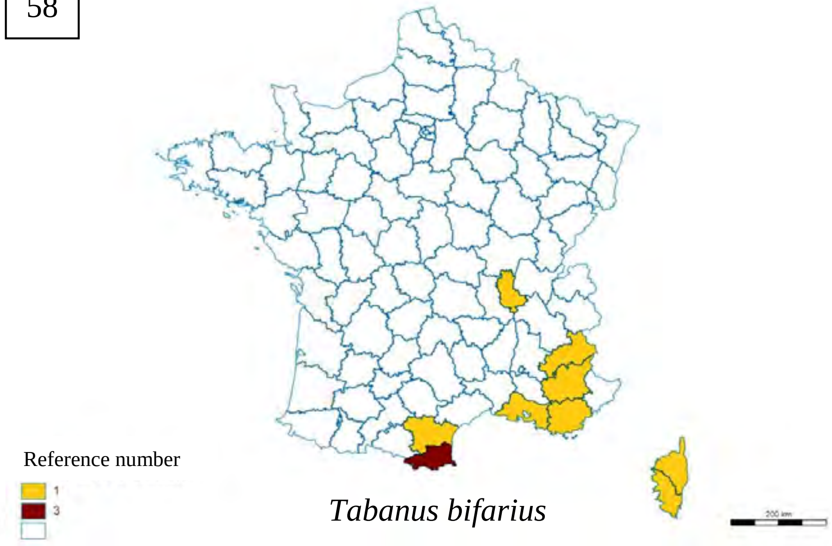

Tabanus bifarius

59

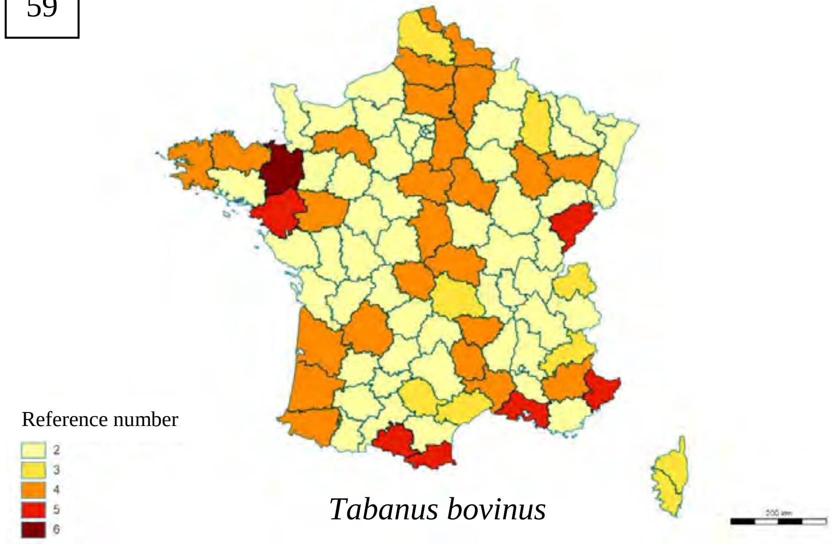

Tabanus bovinus

60

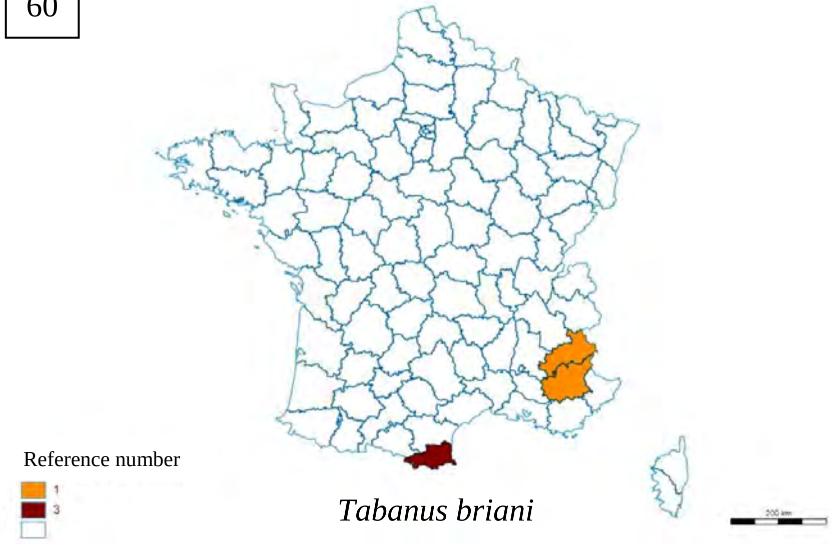

Tabanus briani

61

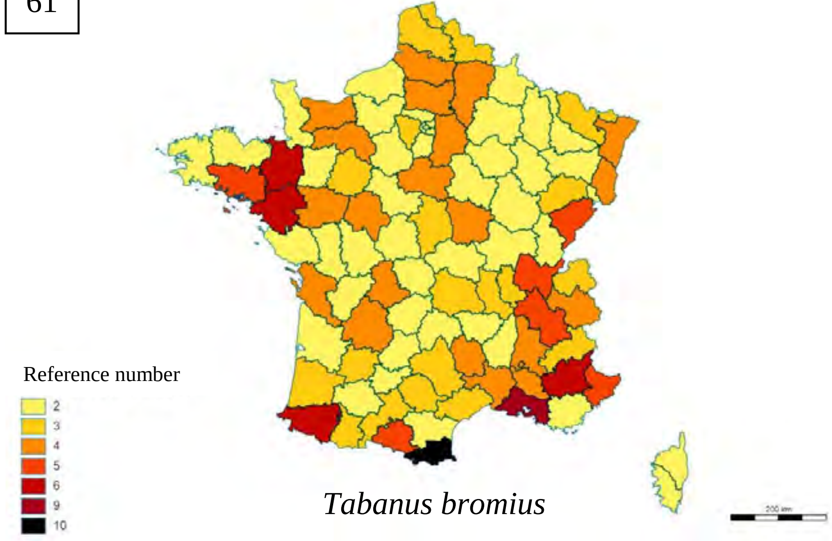

Tabanus bromius

62

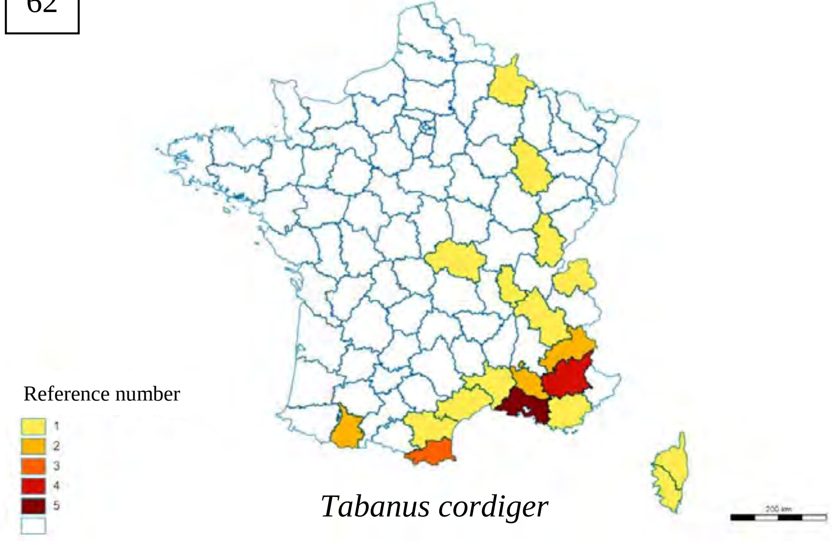

Tabanus cordiger

63

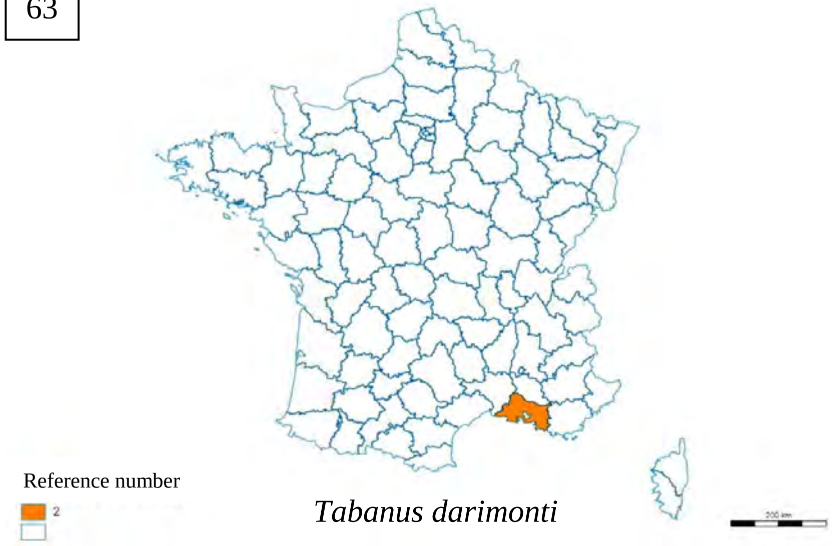

Tabanus darimonti

64

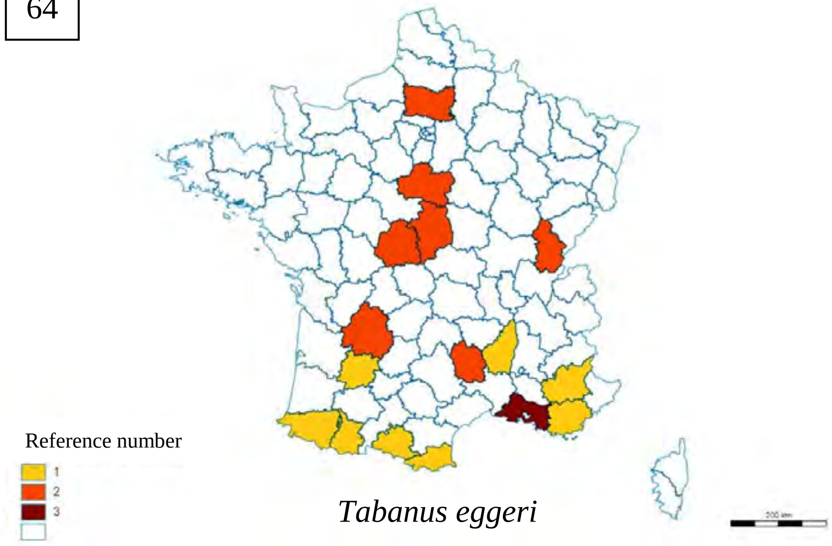

Tabanus eggeri

65

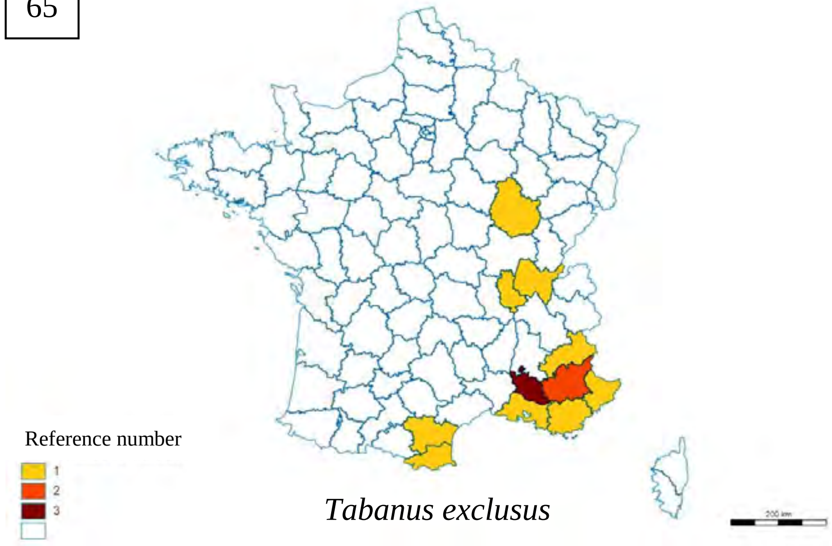

Tabanus exclusus

66

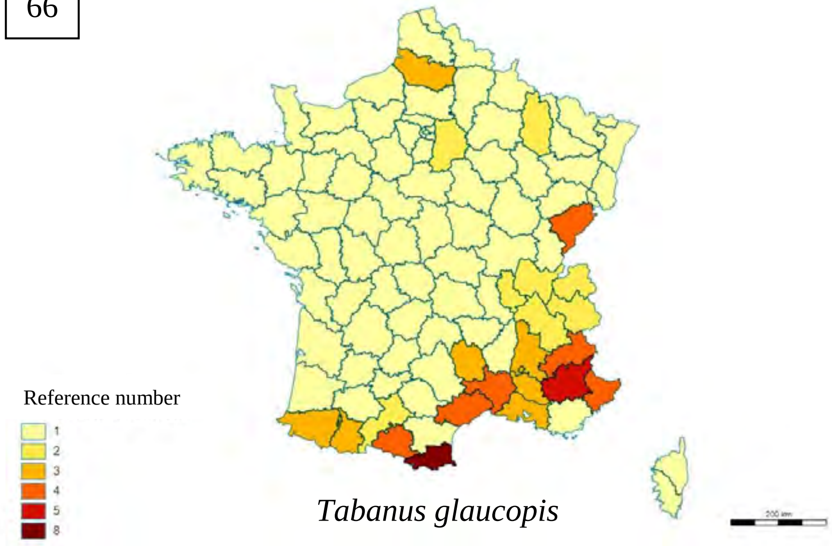

Tabanus glaucopis

67

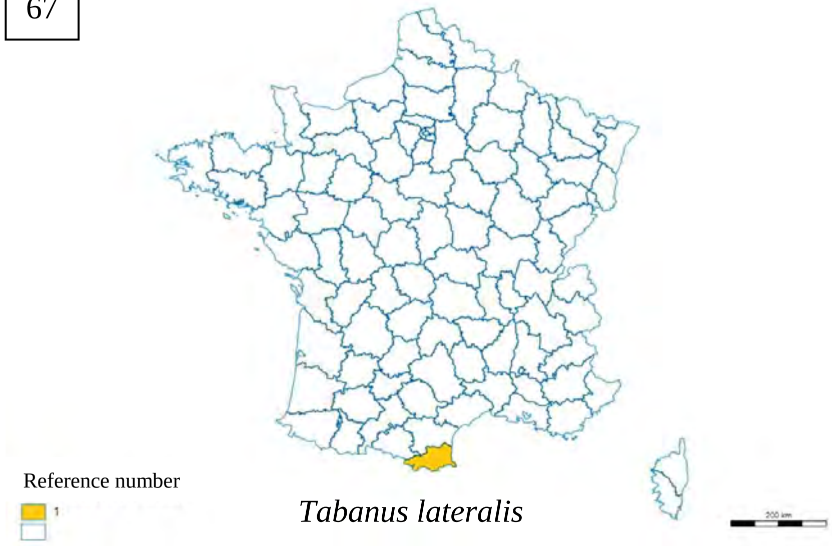

Tabanus lateralis

68

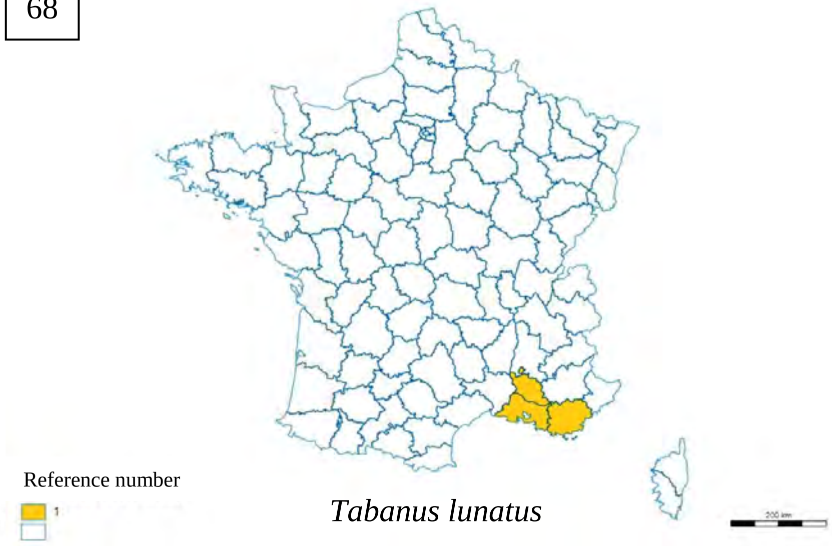

Tabanus lunatus

69

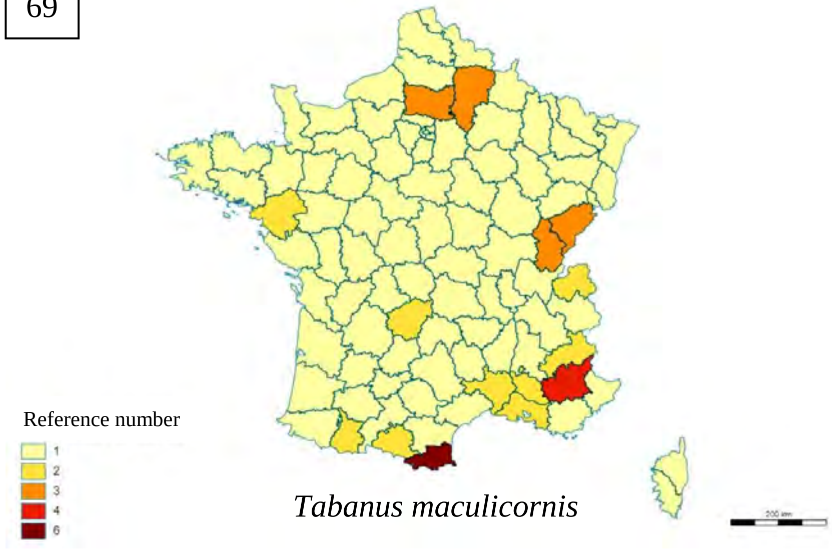

Tabanus maculicornis

70

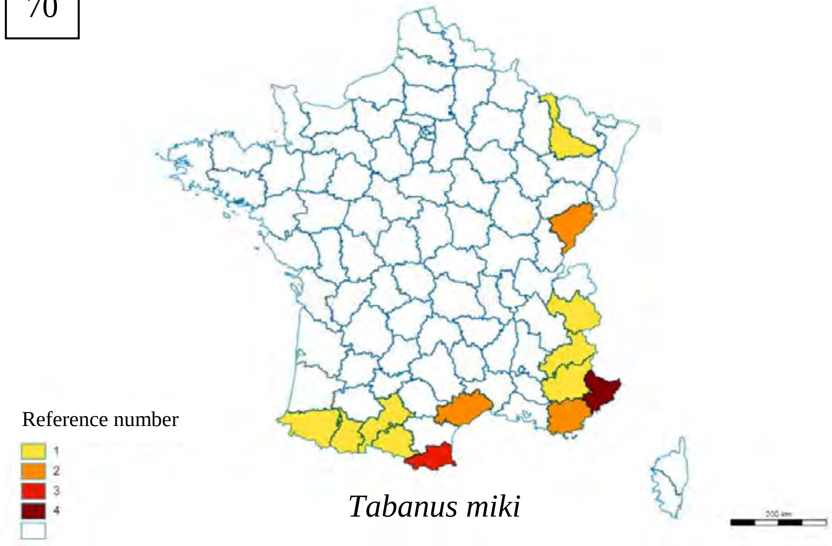

Tabanus miki

71

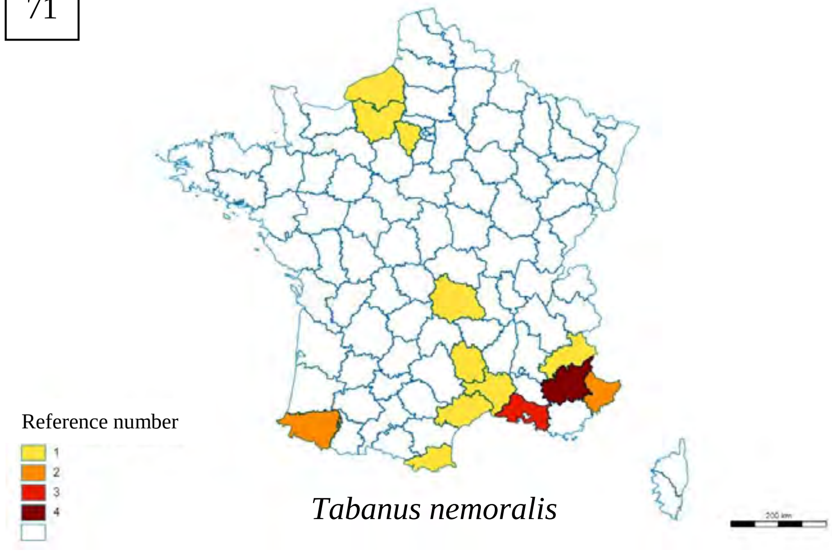

Tabanus nemoralis

72

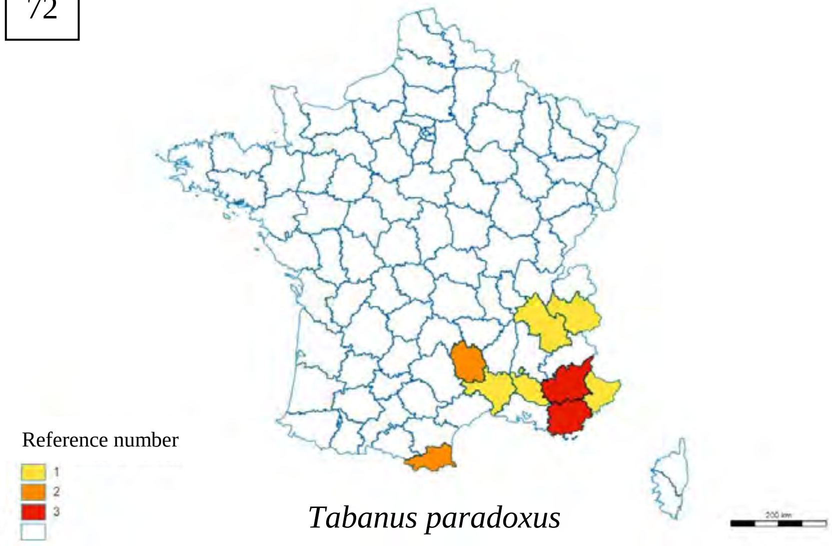

Tabanus paradoxus

73

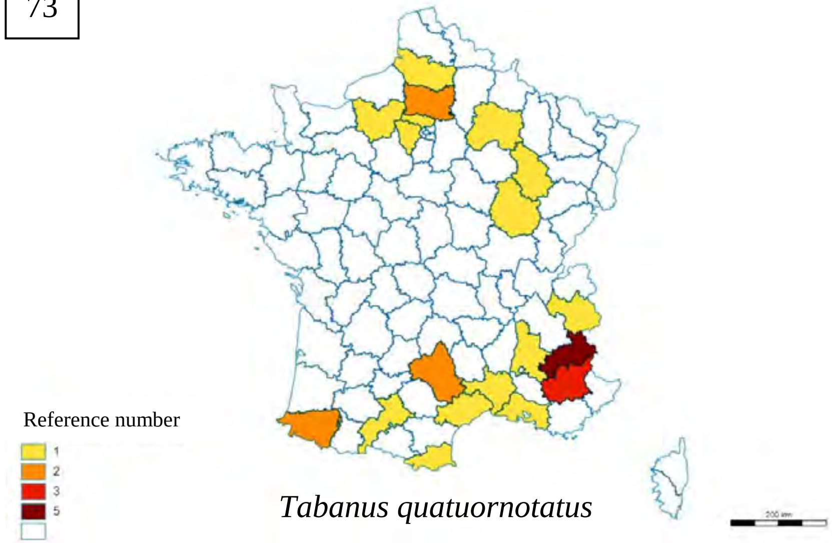

Tabanus quatuornotatus

74

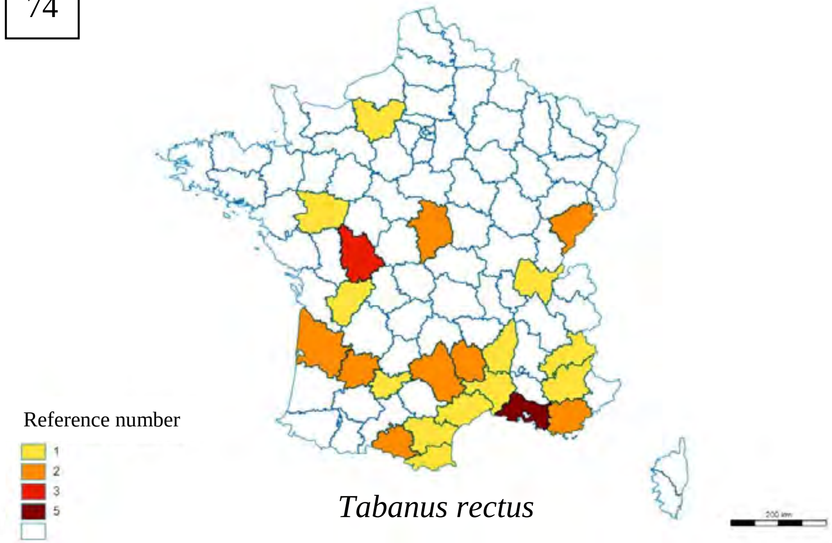

Tabanus rectus

75

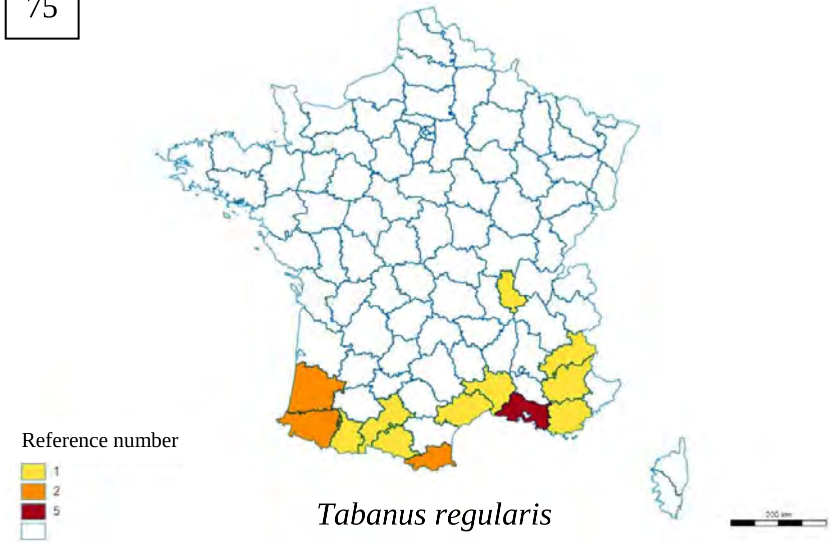

Tabanus regularis

76

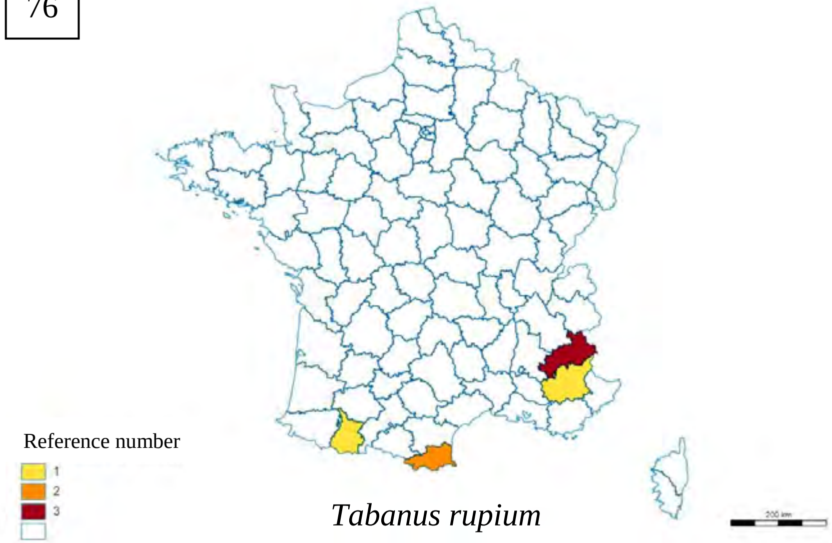

Tabanus rupium

77

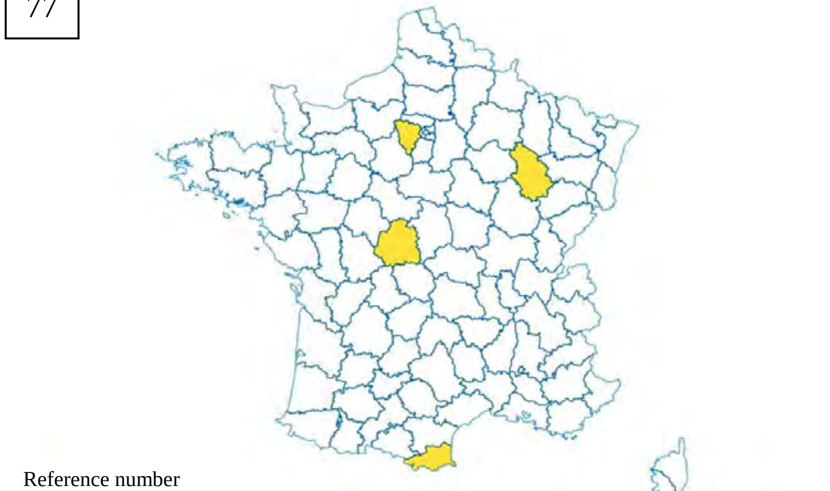

Tabanus spectabilis

78

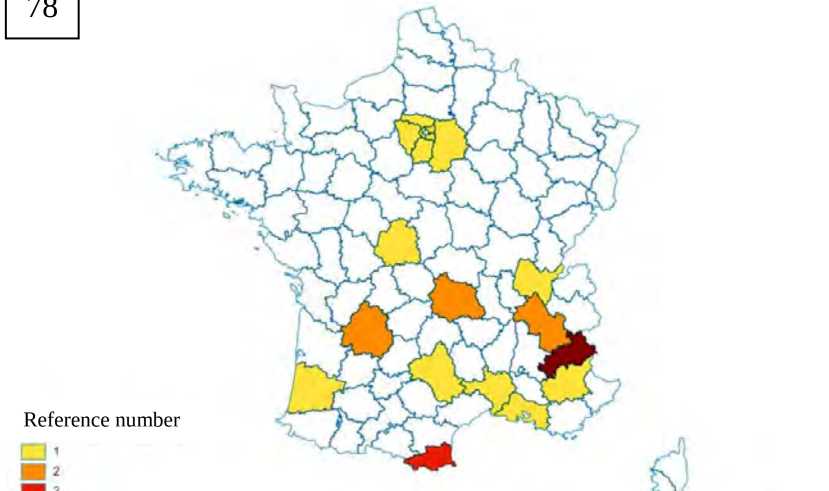

Tabanus spodopterus

79

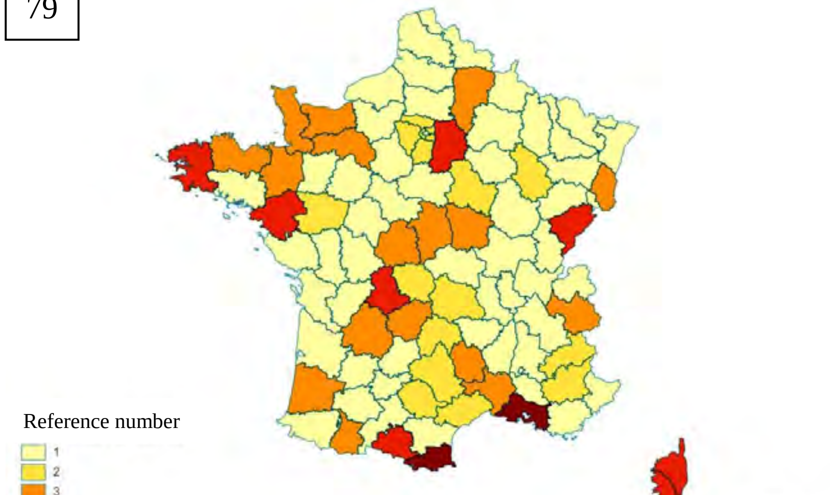

Tabanus sudeticus

80

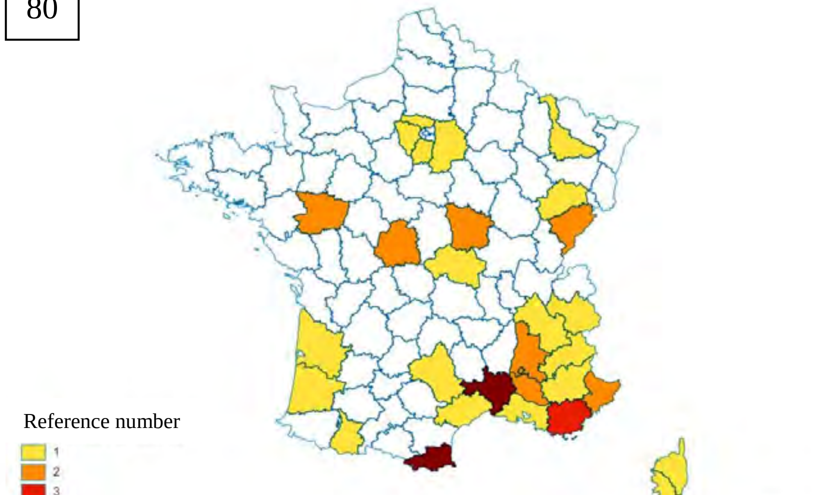

Tabanus tergestinus

81

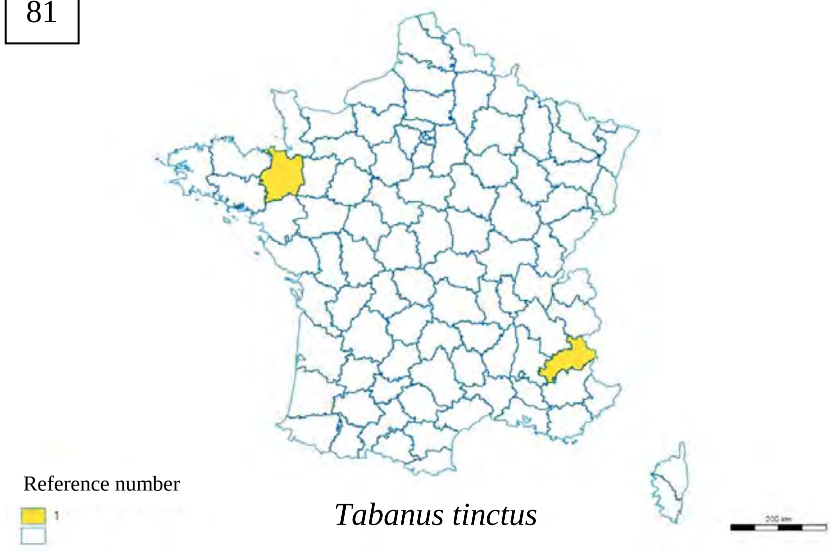

Tabanus tinctus

82

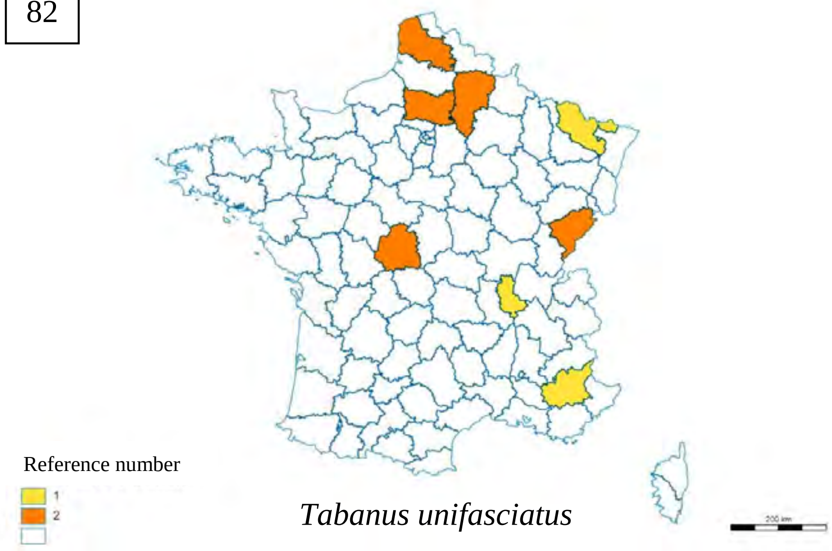

Tabanus unifasciatus

83

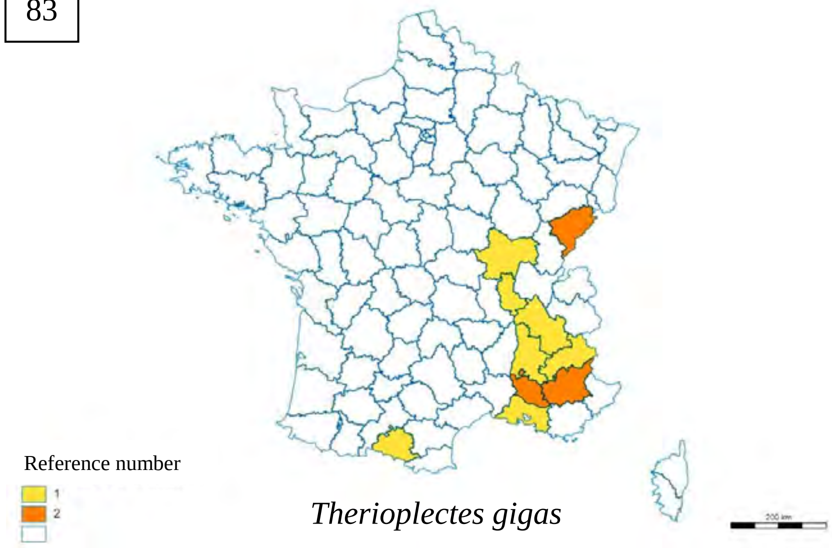

Therioplectes gigas

Supplementary Figure S3. Distribution map of *Atylotus agrestis* (1), *Atylotus flavoguttatus* (2), *Atylotus fulvus* (3), *Atylotus intermedius* (4), *Atylotus latistriatus* (5), *Atylotus loewianus* (6), *Atylotus plebeius* (7), *Atylotus quadrifarius* (8), *Atylotus rusticus* (9), *Chrysops caecutiens* (10), *Chrysops flavipes* (11), *Chrysops italicus* (12), *Chrysops parallelogrammus* (13), *Chrysops pictus* (14), *Chrysops relictus* (15), *Chrysops rufipes* (16), *Chrysops sepulcralis* (17), *Chrysops viduatus* (18), *Dasyrhamphis anthracinus* (19), *Dasyrhamphis ater* (20), *Haematopota bigoti* (21), *Haematopota crassicornis* (22), *Haematopota grandis* (23), *Haematopota italica* (24), *Haematopota lambi* (25), *Haematopota ocelligera* (26), *Haematopota pluvialis* (27), *Haematopota scutellata* (28), *Heptatoma pellucens* (29), *Hybomitra acuminata* (30), *Hybomitra aterrima* (31), *Hybomitra auripila* (32), *Hybomitra bimaculata* (33), *Hybomitra borealis* (34), *Hybomitra caucasica* (35), *Hybomitra ciureai* (36), *Hybomitra distinguenda* (37), *Hybomitra erberi* (38), *Hybomitra expollicata* (39), *Hybomitra lundbecki* (40), *Hybomitra lurida* (41), *Hybomitra micans* (42), *Hybomitra montana* (43), *Hybomitra muhlfeldi* (44), *Hybomitra olsufievina* (45), *Hybomitra solstitialis* (46), *Hybomitra tropica* (47), *Hybomitra vittata* (48), *Nemorius vitripennis* (49), *Pangonius haustellatus* (50), *Pangonius micans* (51), *Philipomyia aprica* (52), *Philipomyia graeca* (53), *Silvius algirus* (54), *Silvius alpinus* (55), *Silvius variegatus* (56), *Tabanus autumnalis* (57), *Tabanus bifarius* (58), *Tabanus bovinus* (59), *Tabanus briani* (60), *Tabanus bromius* (61), *Tabanus cordiger* (62), *Tabanus darimonti* (63), *Tabanus eggeri* (64), *Tabanus exclusus* (65), *Tabanus glaucopis* (66), *Tabanus lateralis* (67), *Tabanus lunatus* (68), *Tabanus maculicornis* (69), *Tabanus miki* (70), *Tabanus nemoralis* (71), *Tabanus paradoxus* (72), *Tabanus quatuornotatus* (73), *Tabanus rectus* (74), *Tabanus regularis* (75), *Tabanus rupium* (76), *Tabanus spectabilis* (77), *Tabanus spodopterus* (78), *Tabanus sudeticus* (79), *Tabanus tergestinus* (80), *Tabanus tinctus* (81), *Tabanus unifasciatus* (82) and *Therioplectes gigas* (83) by department according to the number of references.

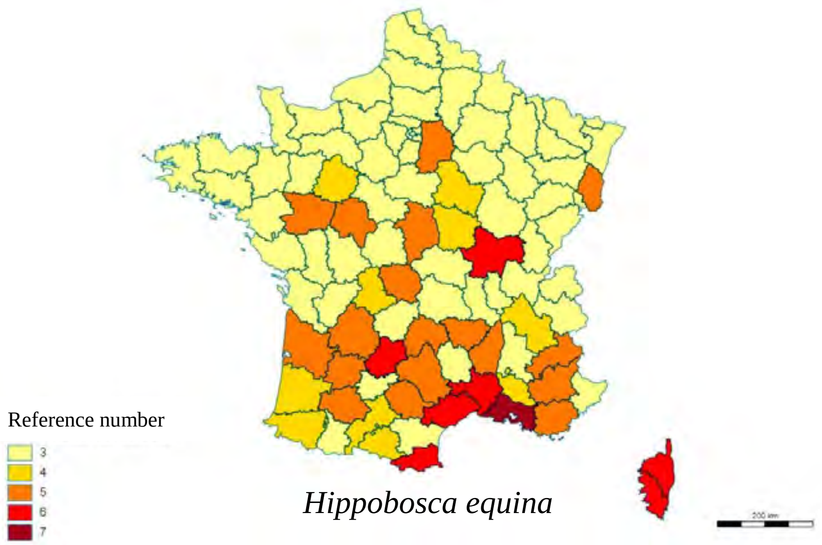

Supplementary Figure S4. Distribution map of *Hippobosca equina* by department according to the number of references.

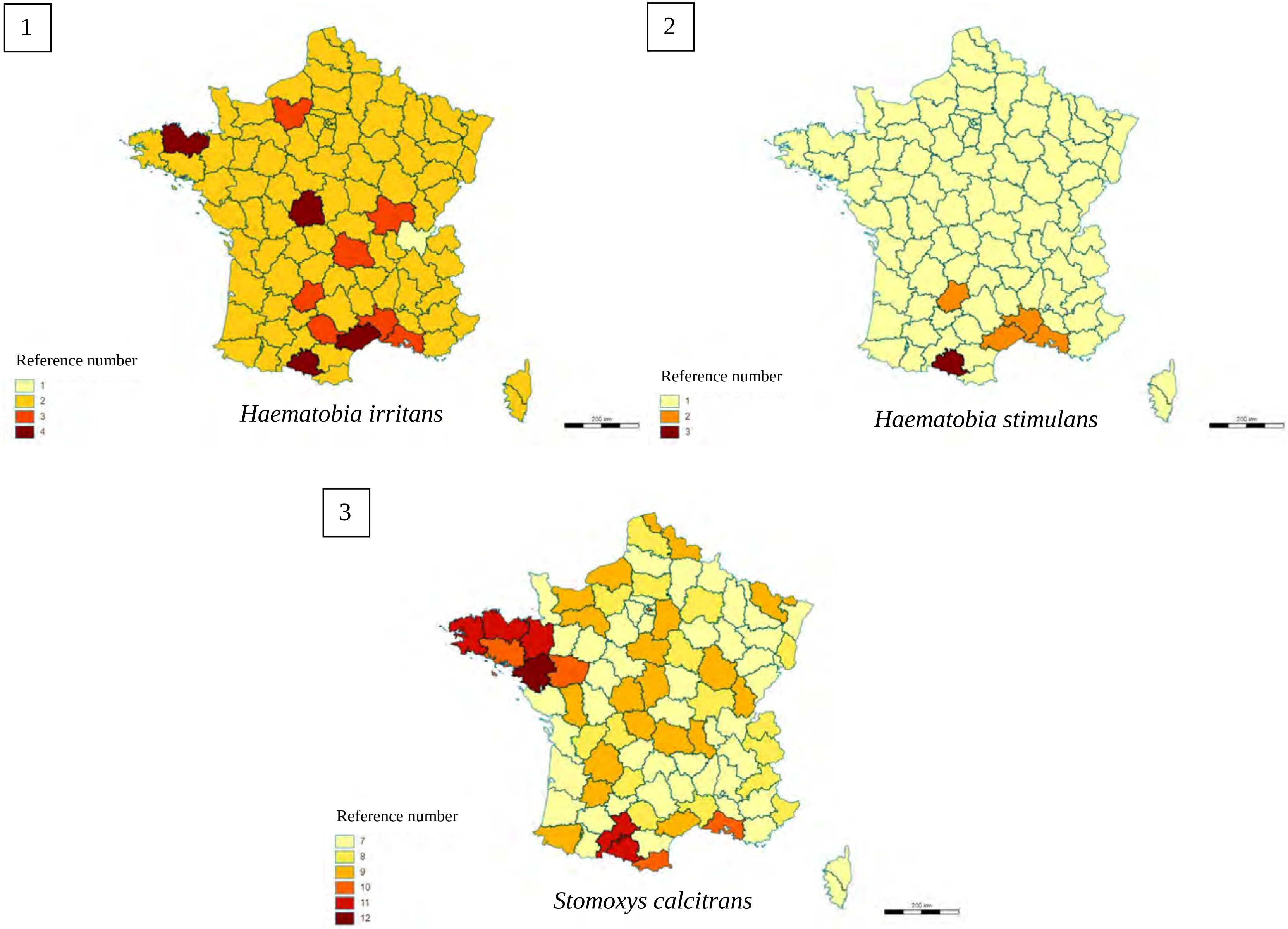

Supplementary Figure S5. Distribution map of *Haematobia irritans* (1), *Haematobia stimulans* (2) and *Stomoxys calcitrans* (3) by department according to the number of references.

1

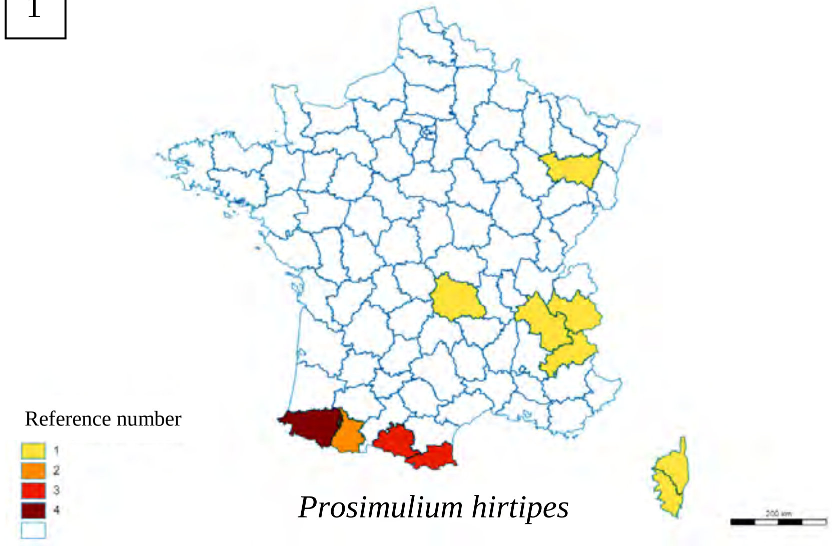
Reference number
1
2
3
4
Prosimulium hirtipes

2

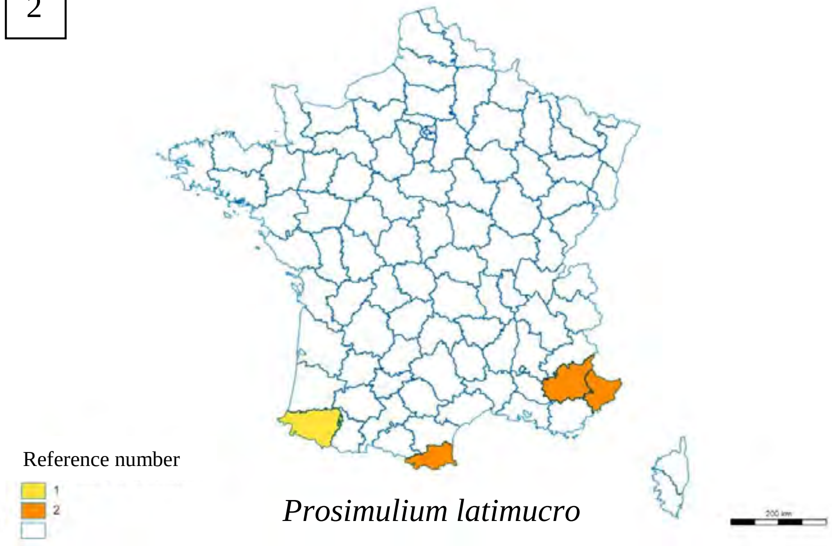
Reference number
1
2
Prosimulium latimucro

3

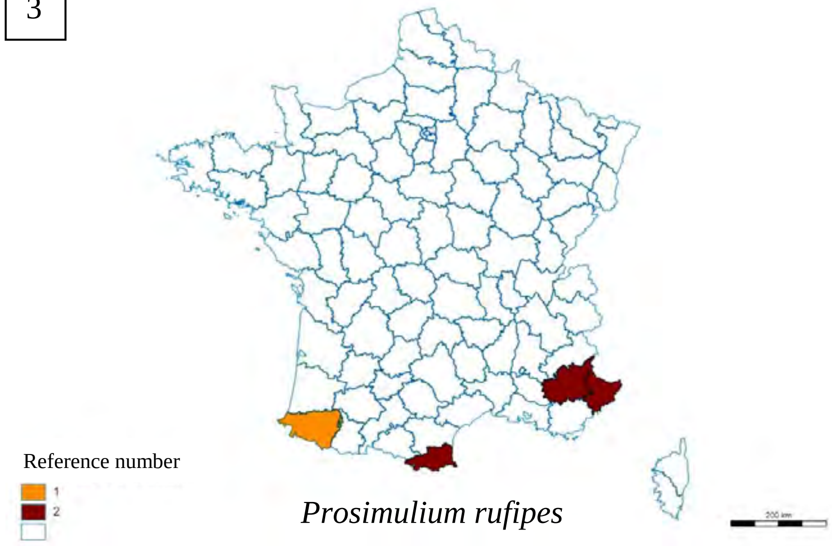
Reference number
1
2
Prosimulium rufipes

4

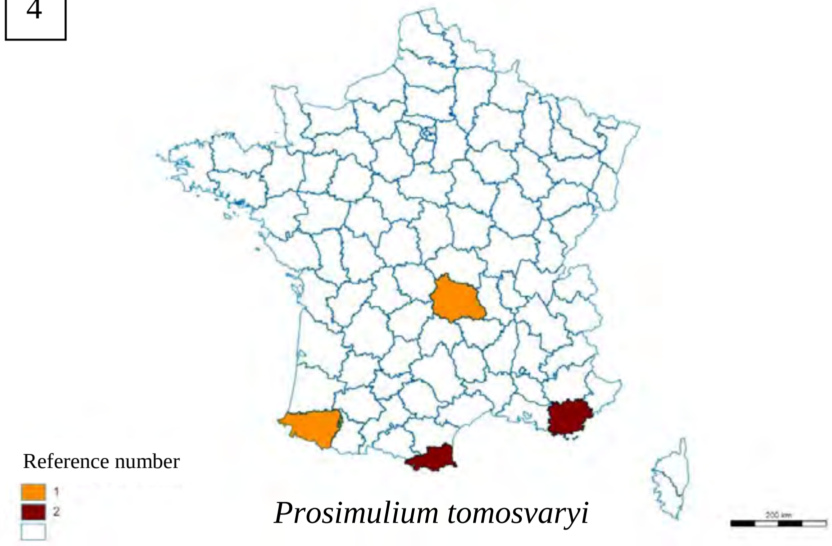
Reference number
1
2
Prosimulium tomosvaryi

5

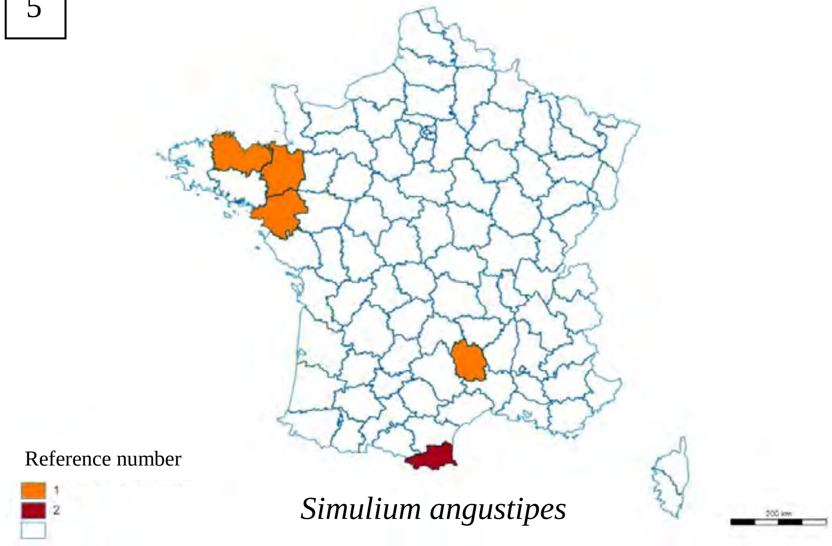

Simulium angustipes

6

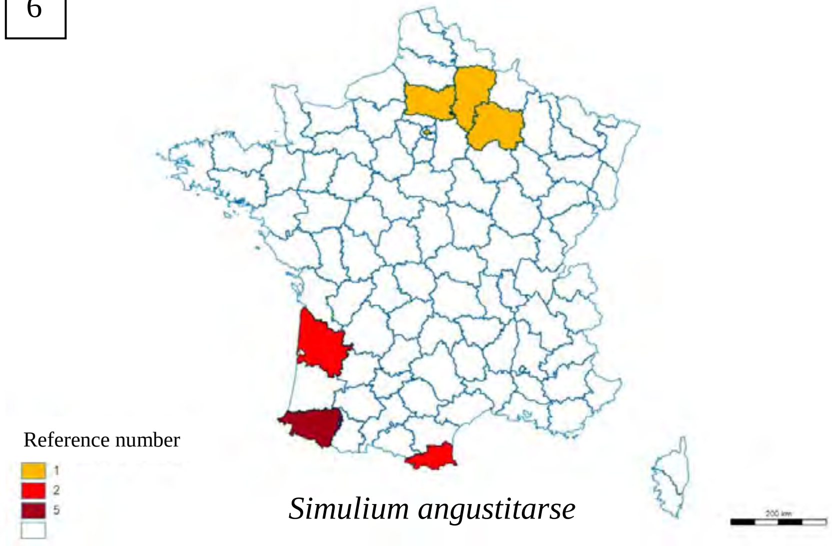

Simulium angustitarse

7

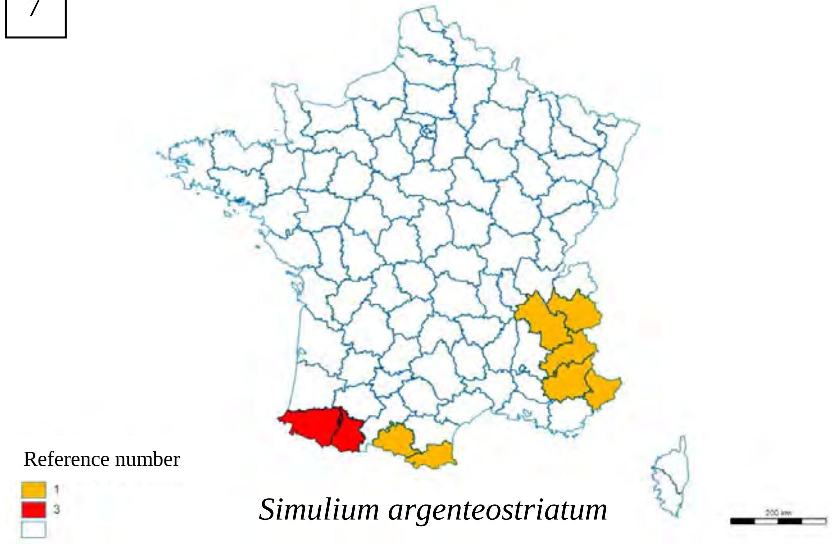

Simulium argenteostriatum

8

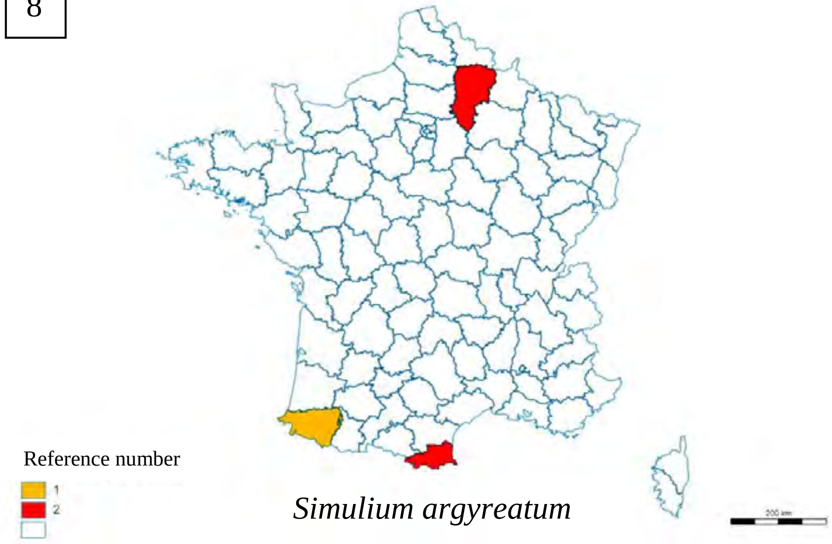

Simulium argyreatum

9

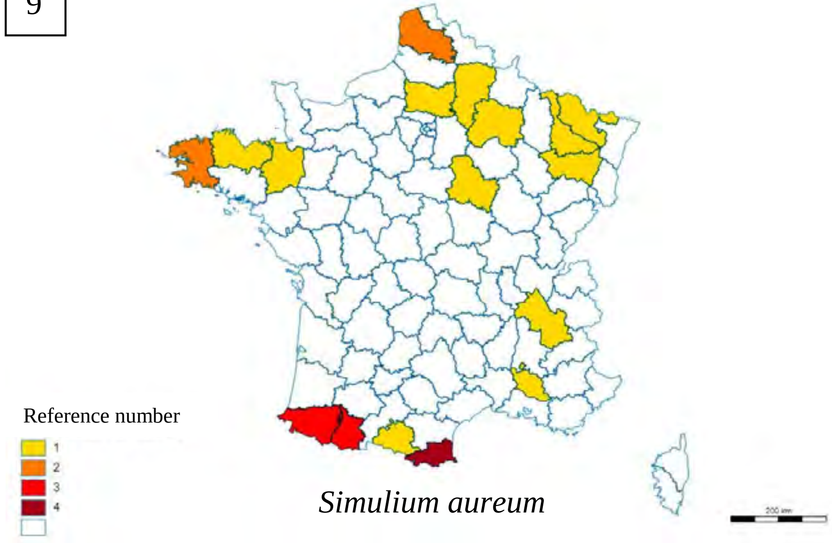
Reference number
1
2
3
4
Simulium aureum

10

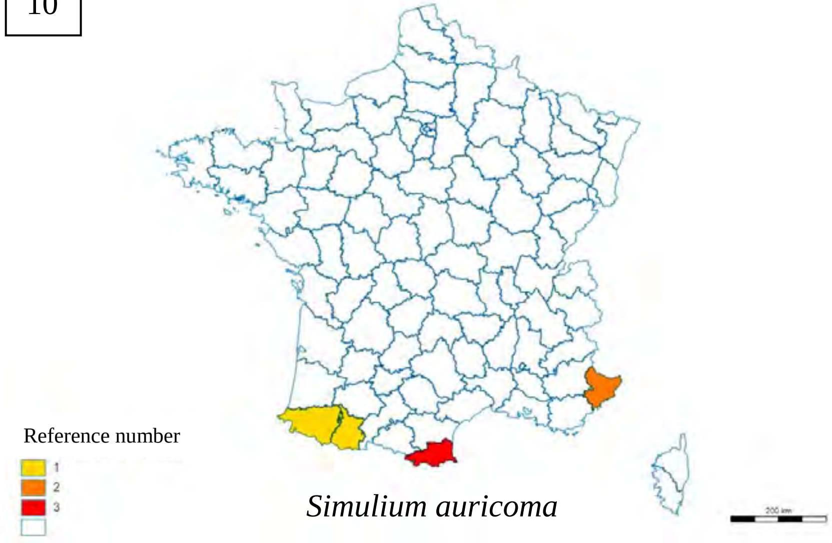
Reference number
1
2
3
Simulium auricoma

11

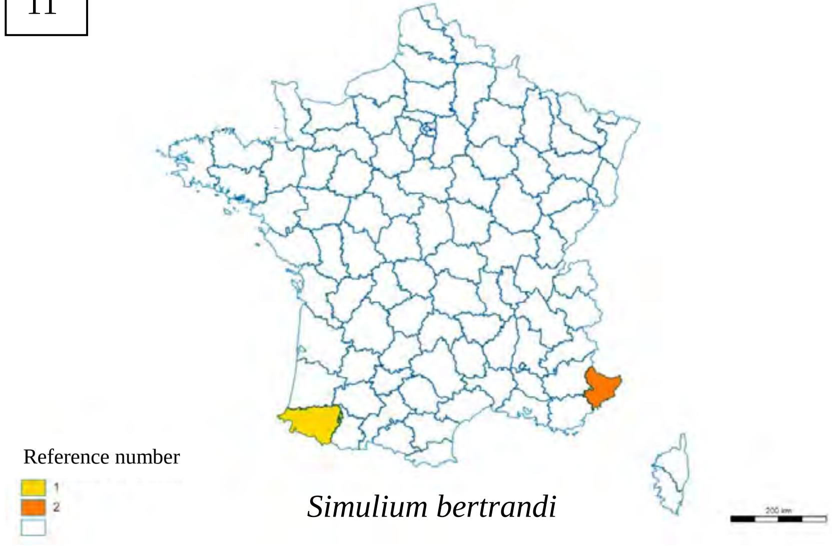
Reference number
1
2
Simulium bertrandi

12

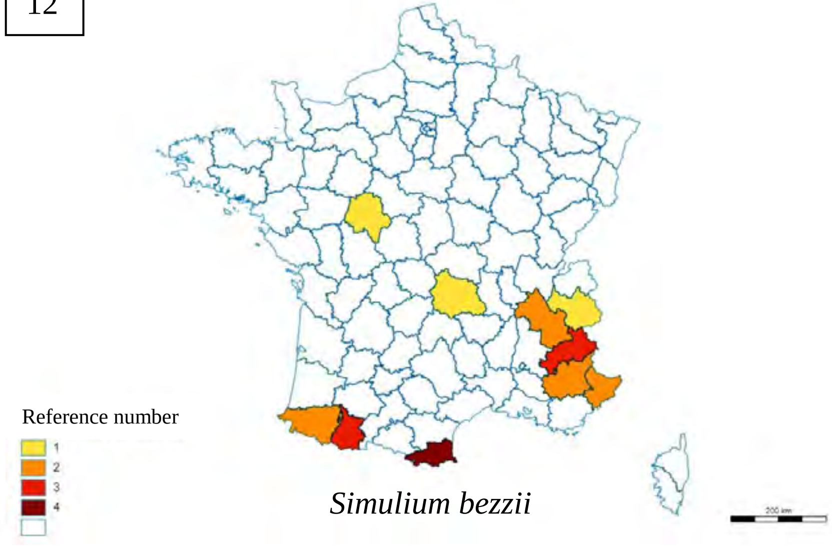
Reference number
1
2
3
4
Simulium bezzii

13

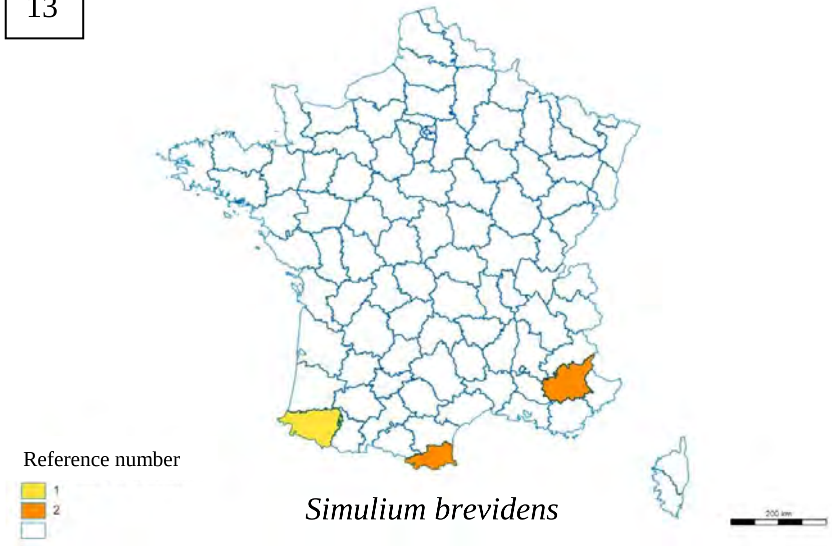

Simulium brevidens

14

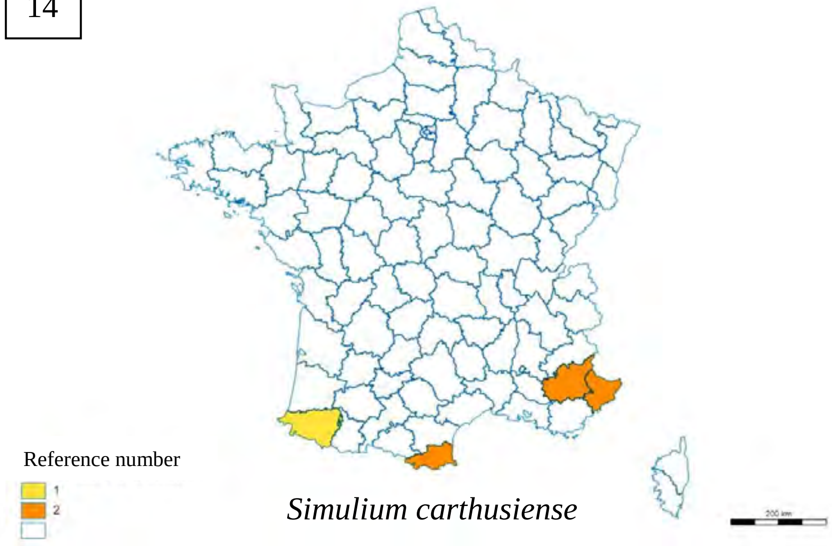

Simulium carthusiense

15

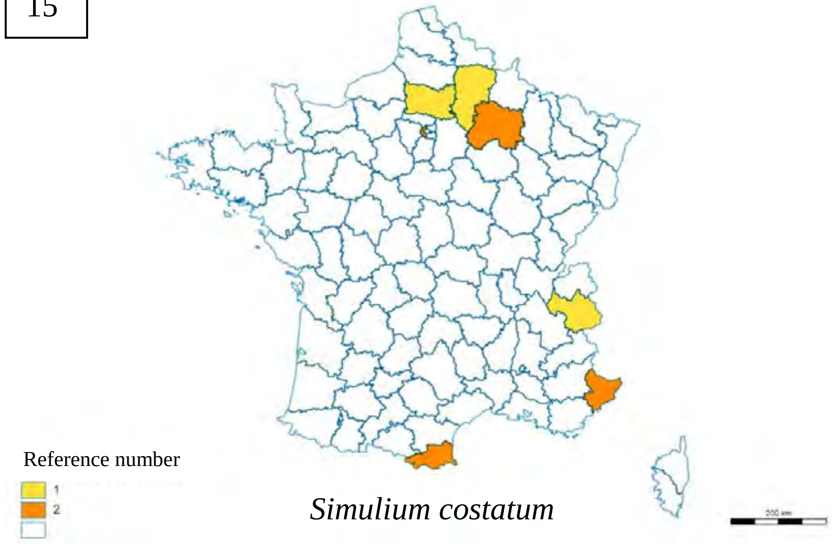

Simulium costatum

16

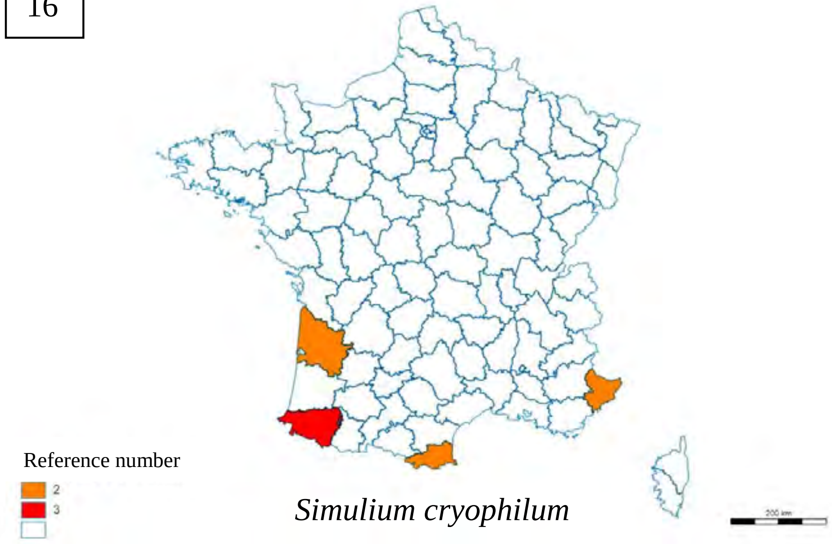

Simulium cryophilum

17

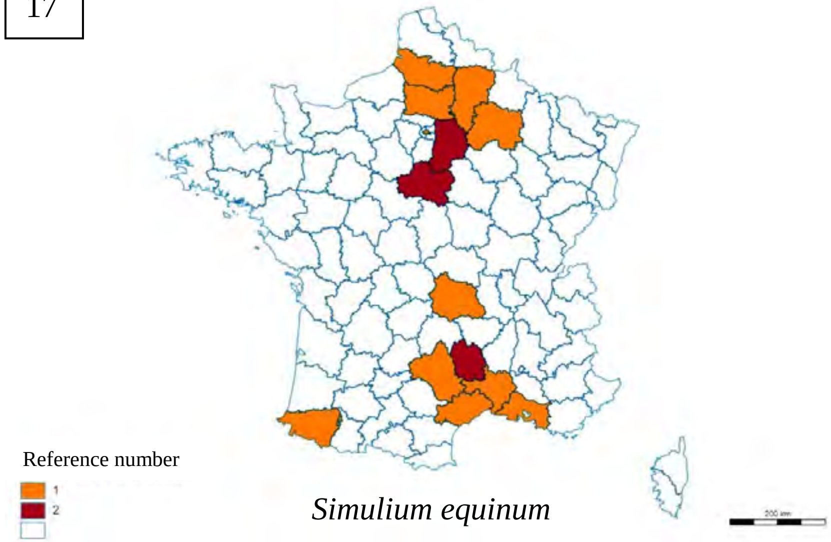

Simulium equinum

18

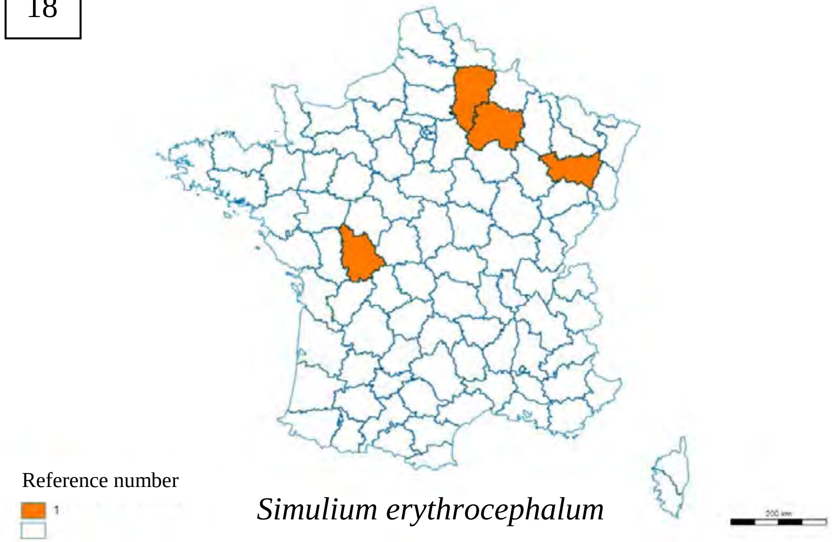

Simulium erythrocephalum

19

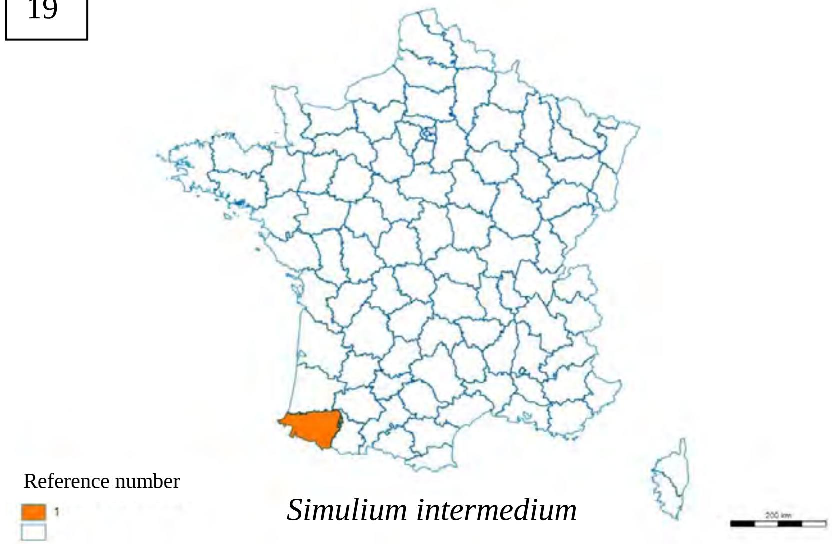

Simulium intermedium

20

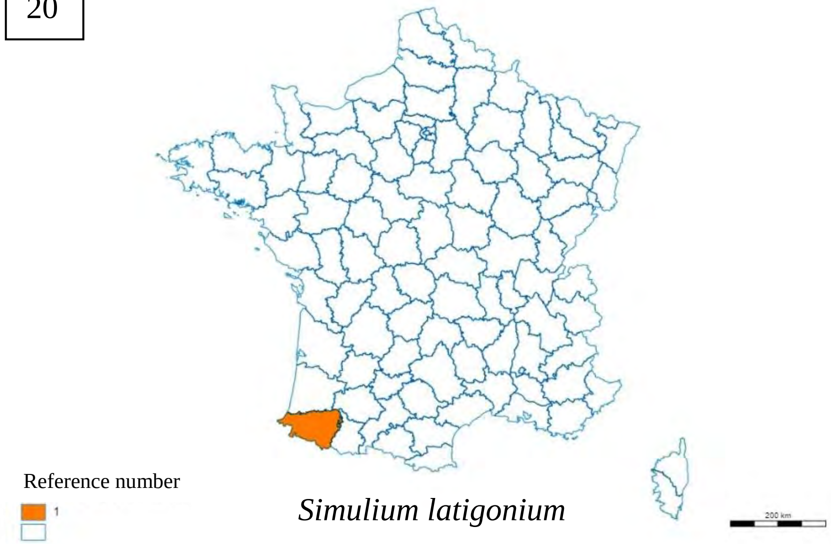

Simulium latigonium

21

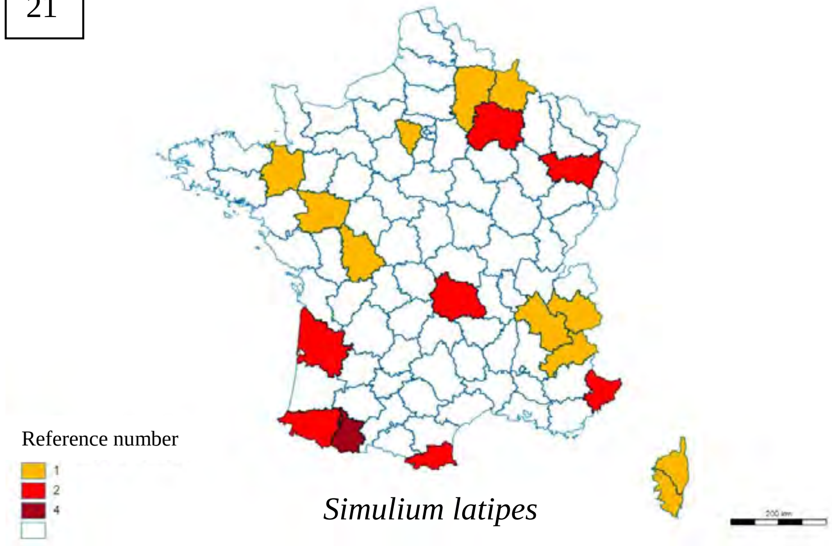

Simulium latipes

22

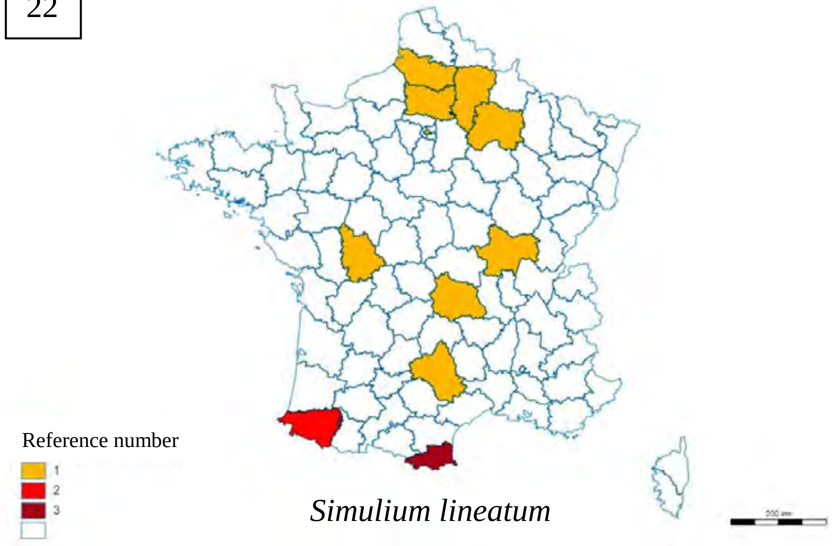

Simulium lineatum

23

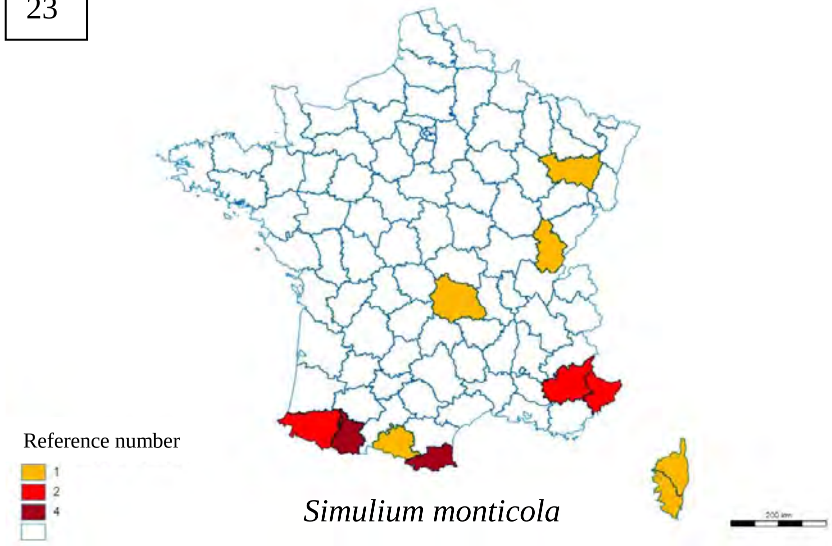

Simulium monticola

24

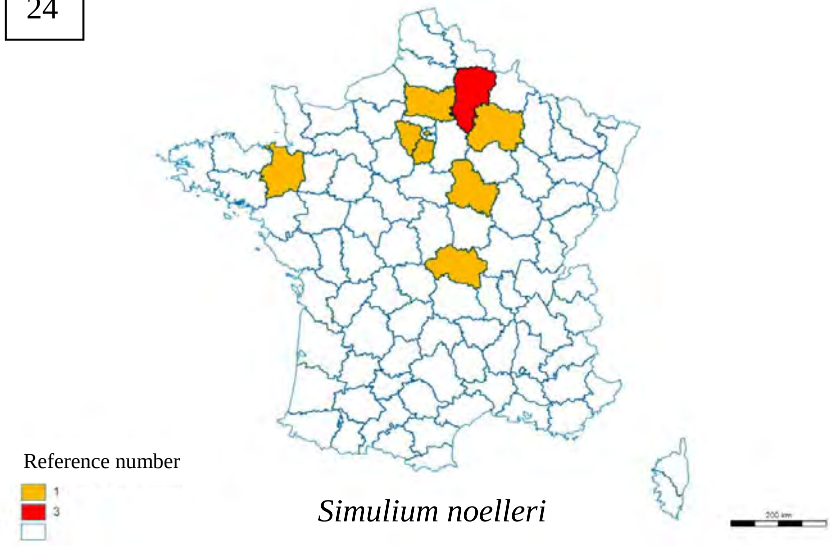

Simulium noelleri

25

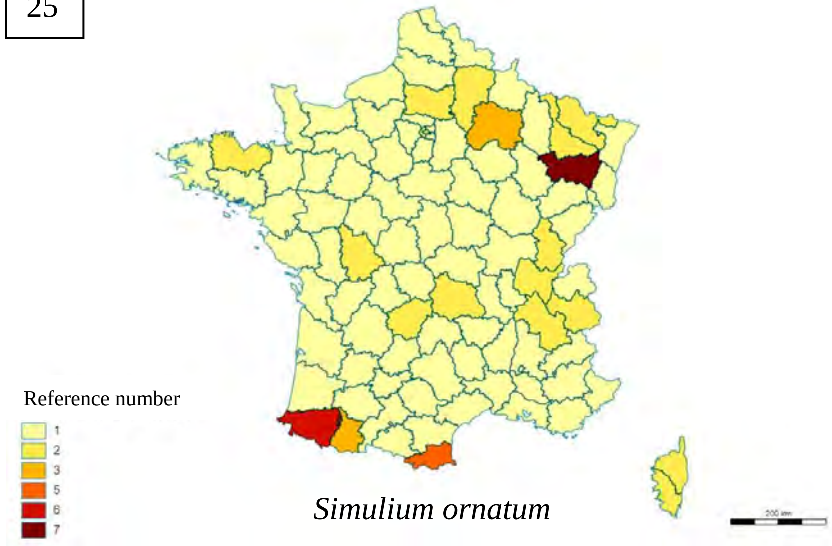
Reference number
1
2
3
5
6
7
Simulium ornatum

26

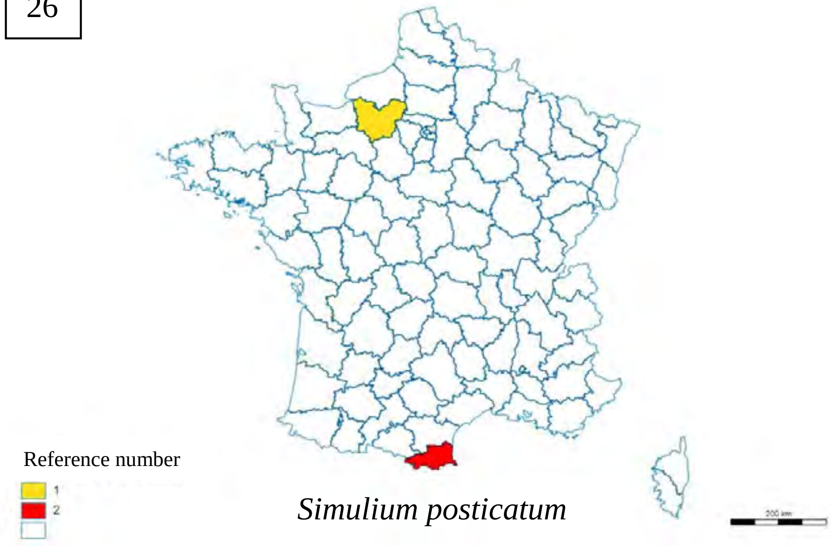
Reference number
1
2
Simulium posticatum

27

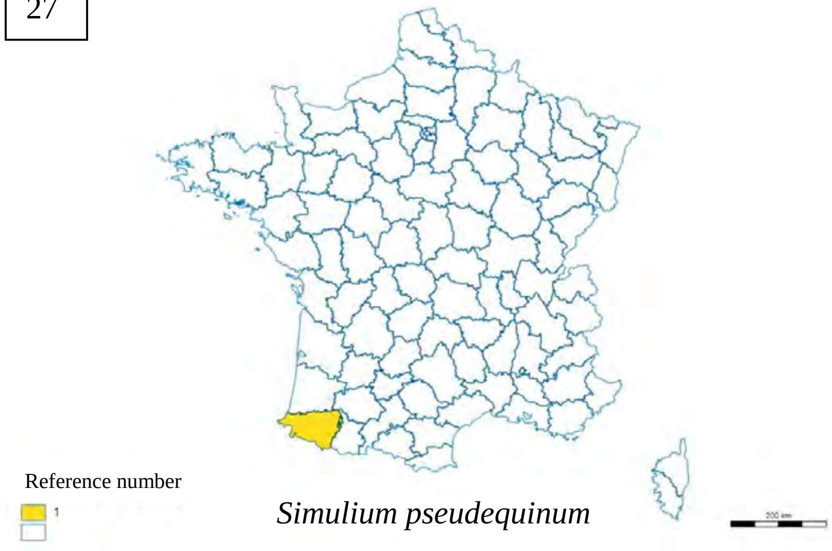
Reference number
1
Simulium pseudequinum

28

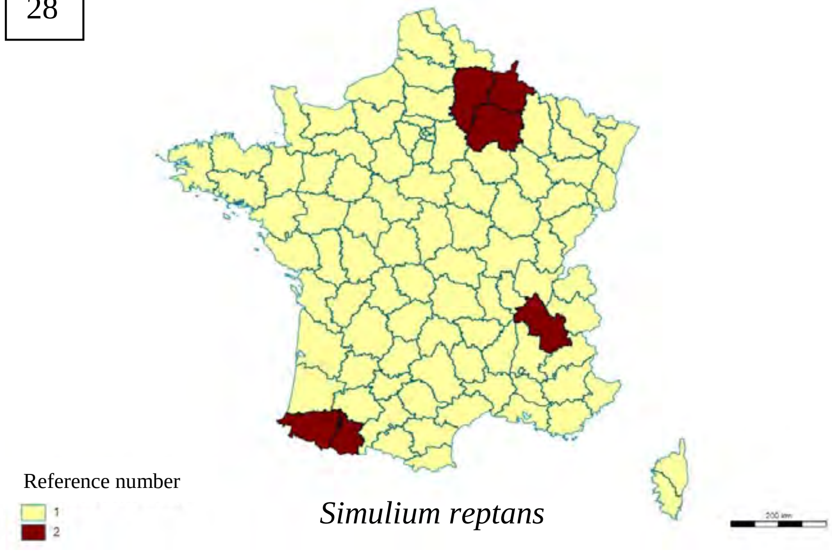
Reference number
1
2
Simulium reptans

29

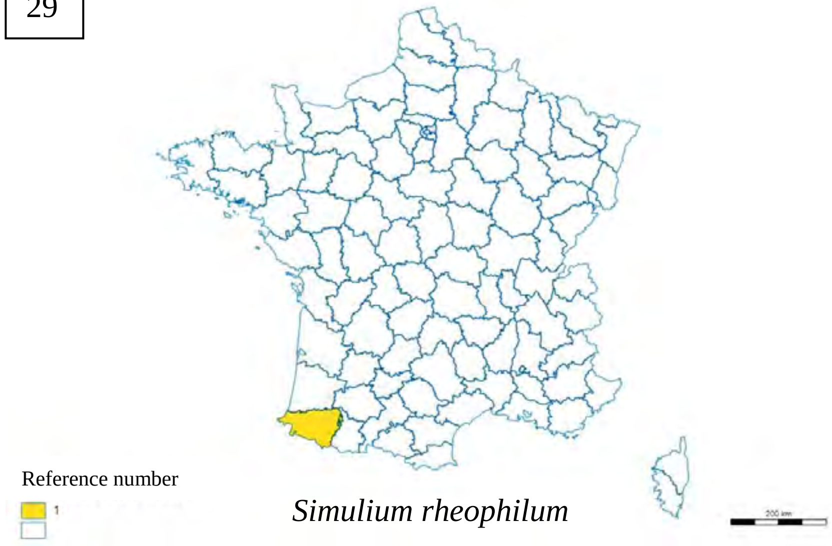

Simulium rheophilum

30

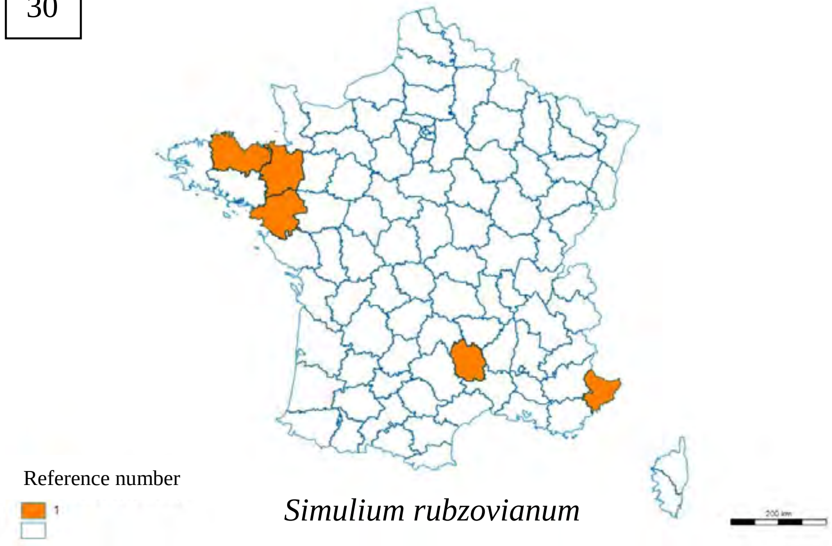

Simulium rubzovianum

31

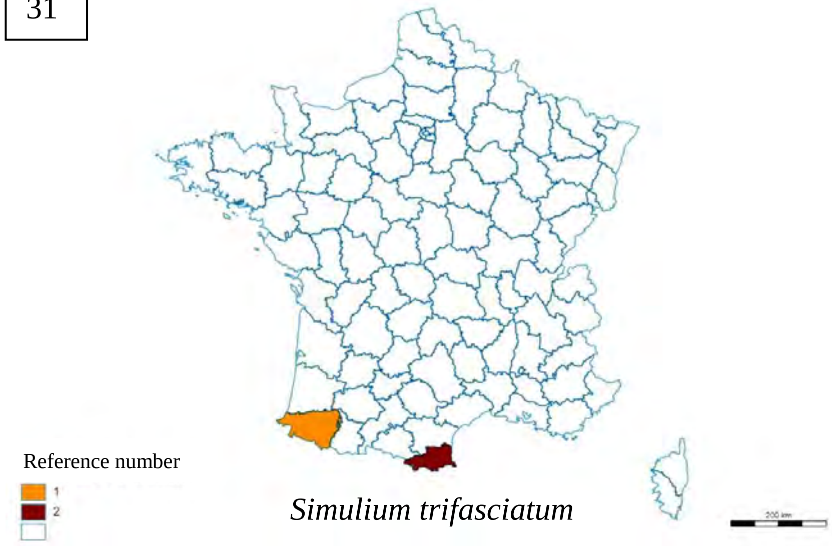

Simulium trifasciatum

32

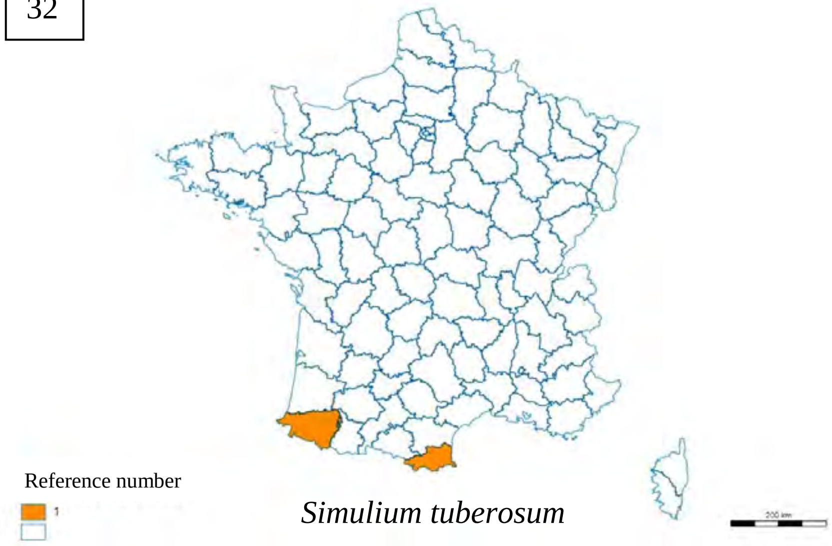

Simulium tuberosum

33

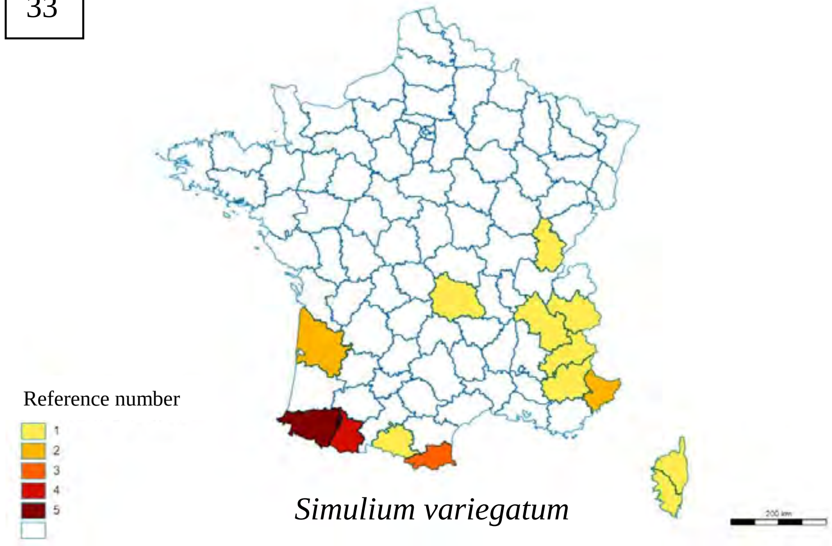

Simulium variegatum

34

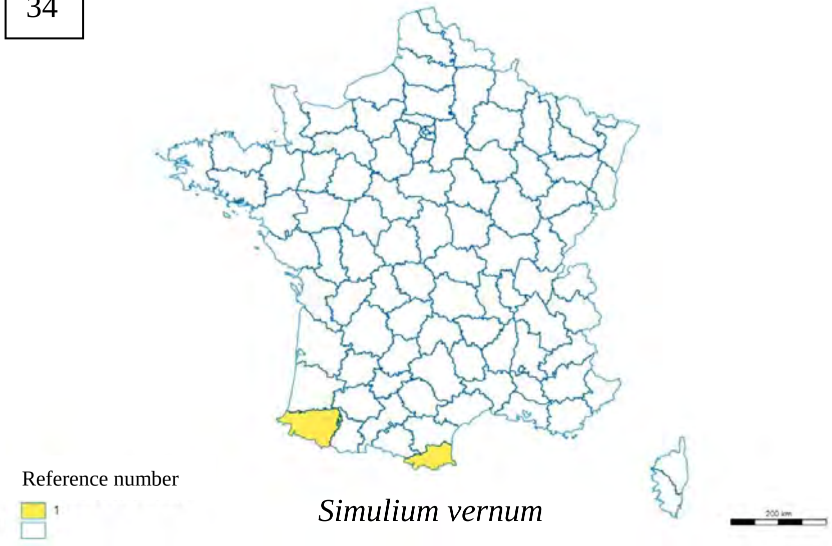

Simulium vernum

35

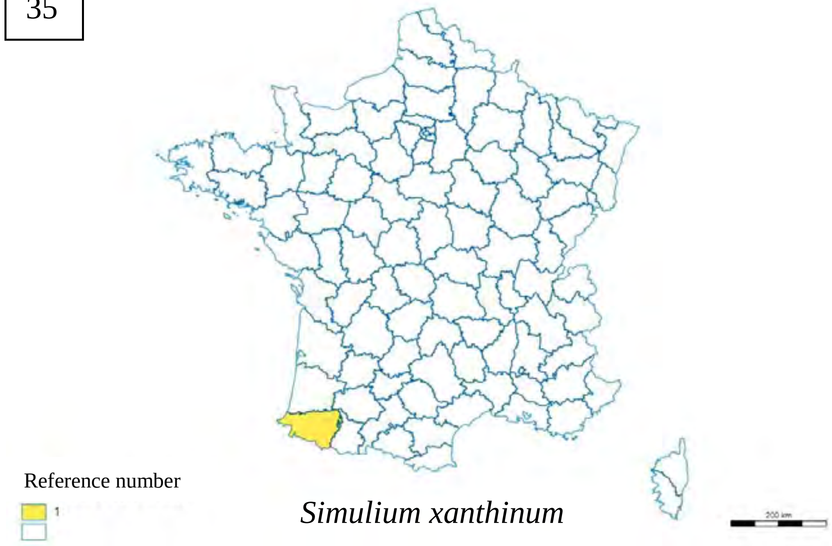

Simulium xanthinum

Supplementary Figure S6. Distribution map of *Prosimulium hirtipes* (1), *Prosimulium latimucro* (2), *Prosimulium rufipes* (3), *Prosimulium tomosvaryi* (4), *Simulium angustipes* (5), *Simulium angustitarse* (6), *Simulium argenteostriatum* (7), *Simulium argyreatum* (8), *Simulium aureum* (9), *Simulium auricoma* (10), *Simulium bertrandi* (11), *Simulium bezzii* (12), *Simulium brevidens* (13), *Simulium carthusiense* (14), *Simulium costatum* (15), *Simulium cryophilum* (16), *Simulium equinum* (17), *Simulium erythrocephalum* (18), *Simulium intermedium* (19), *Simulium latigonium* (20), *Simulium latipes* (21), *Simulium lineatum* (22), *Simulium monticola* (23), *Simulium noelleri* (24), *Simulium ornatum* (25), *Simulium posticatum* (26), *Simulium pseudequinum* (27), *Simulium reptans* (28), *Simulium rheophilum* (29), *Simulium rubzovianum* (30), *Simulium trifasciatum* (31), *Simulium tuberosum* (32), *Simulium variegatum* (33), *Simulium vernum* (34) and *Simulium xanthinum* (35) by department according to the number of references.

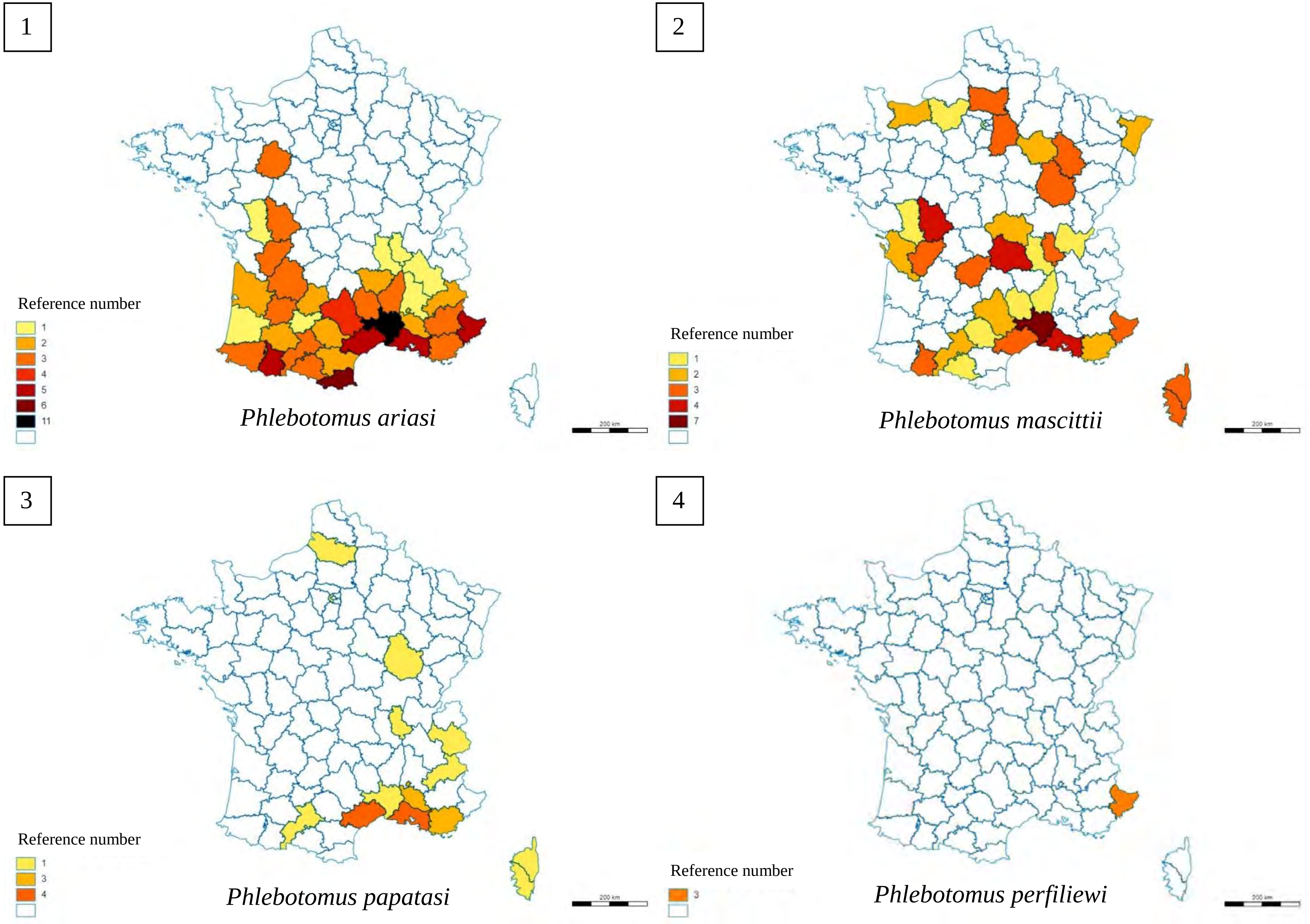
1
Reference number
1
2
3
4
5
6
11
Phlebotomus ariasi
200 km
2
Reference number
1
2
3
4
7
Phlebotomus mascittii
200 km
3
Reference number
1
3
4
Phlebotomus papatasi
200 km
4
Reference number
3
Phlebotomus perfiliewi
200 km

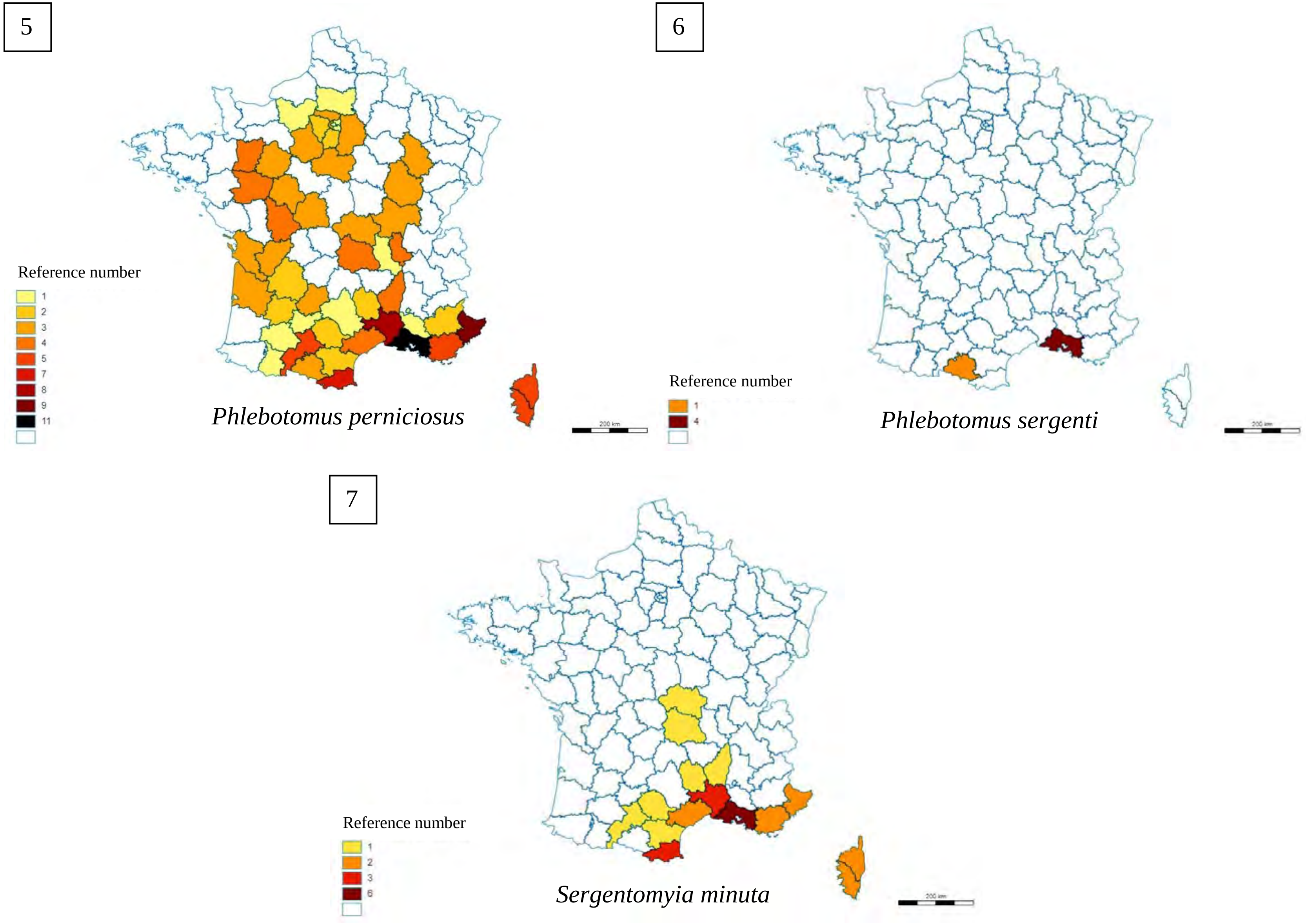

Supplementary Figure S7. Distribution map of *Phlebotomus ariasi* (1), *Phlebotomus mascittii* (2), *Phlebotomus papatasi* (3), *Phlebotomus perfiliewi* (4), *Phlebotomus perniciosus* (5), *Phlebotomus sergenti* (6) and *Sergentomyia minuta* (7) by department according to the number of references.

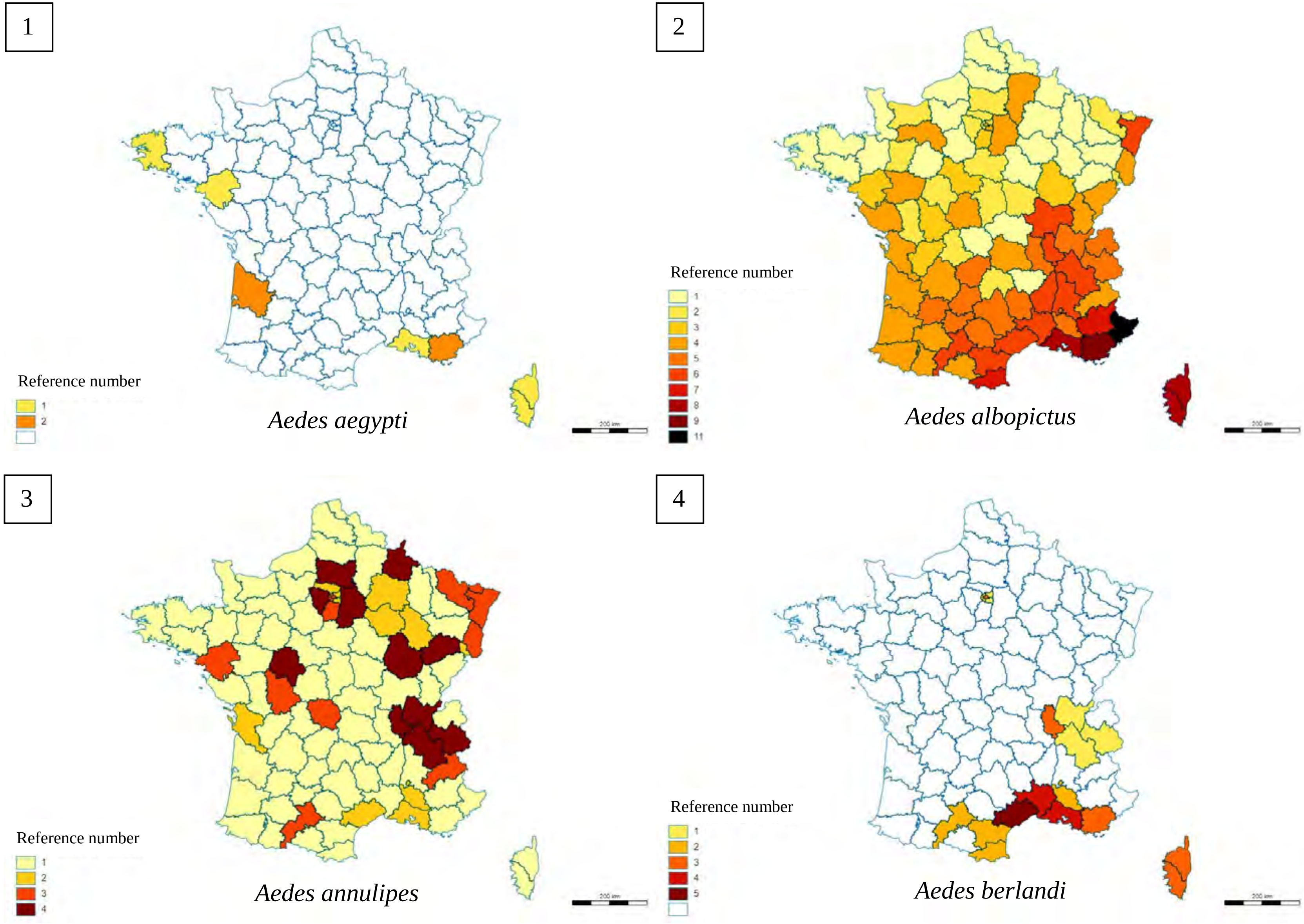
1
Reference number
1
2
Aedes aegypti
2
Reference number
1
2
3
4
5
6
7
8
9
11
Aedes albopictus
3
Reference number
1
2
3
4
Aedes annulipes
4
Reference number
1
2
3
4
5
Aedes berlandi

5

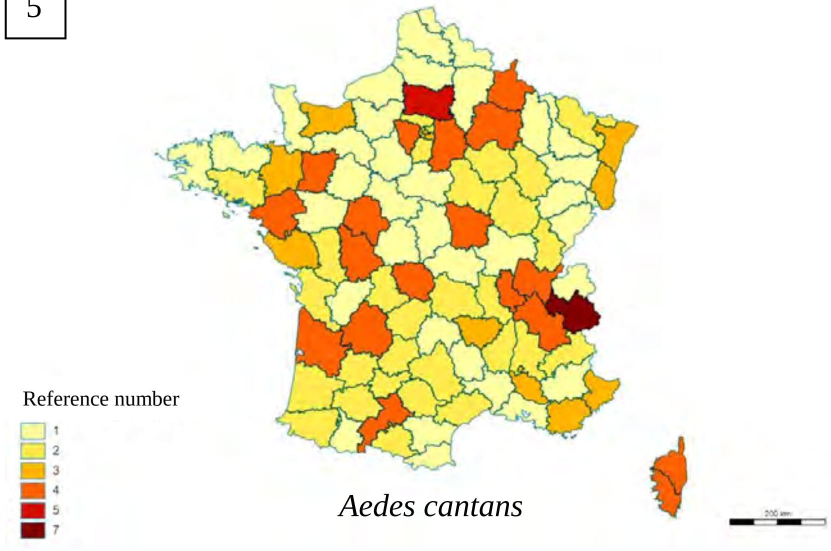
Reference number
1
2
3
4
5
7
Aedes cantans

6

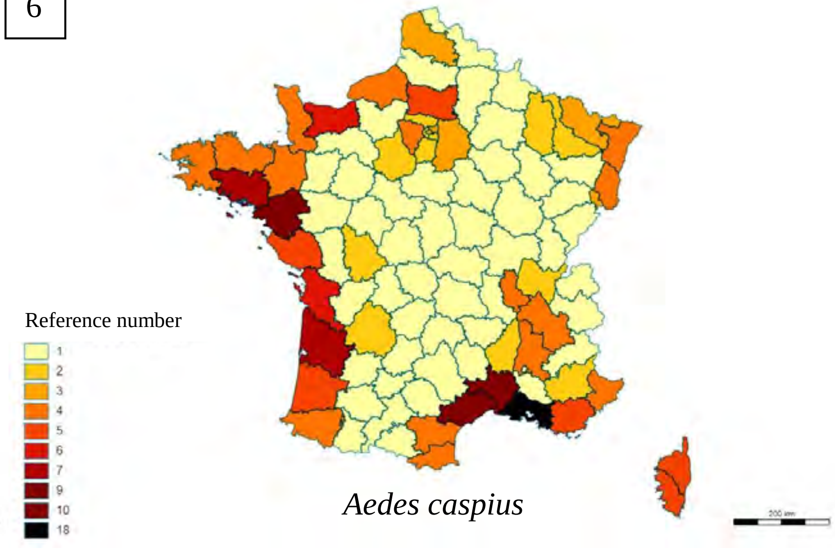
Reference number
1
2
3
4
5
6
7
9
10
18
Aedes caspius

7

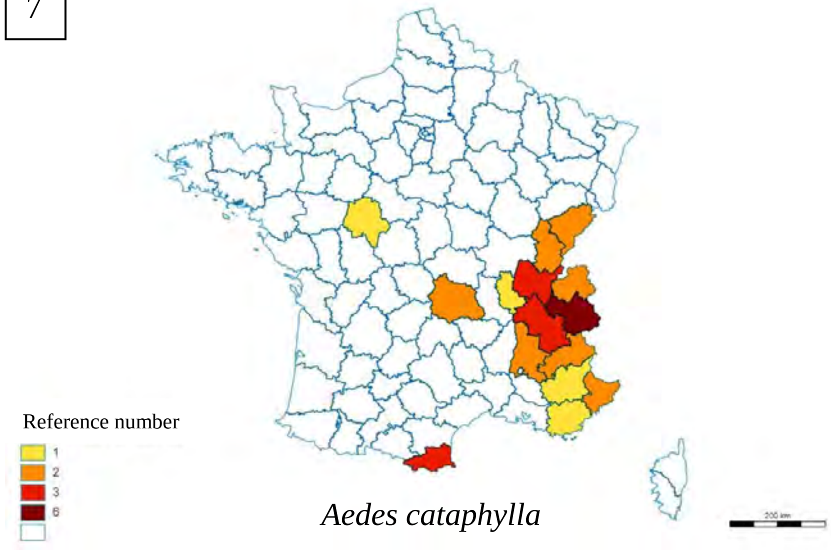
Reference number
1
2
3
6
Aedes cataphylla

8

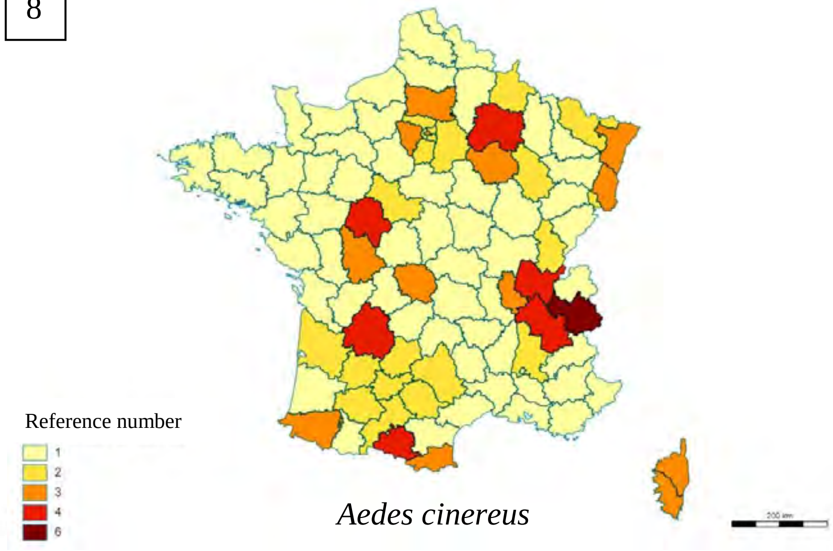
Reference number
1
2
3
4
6
Aedes cinereus

9

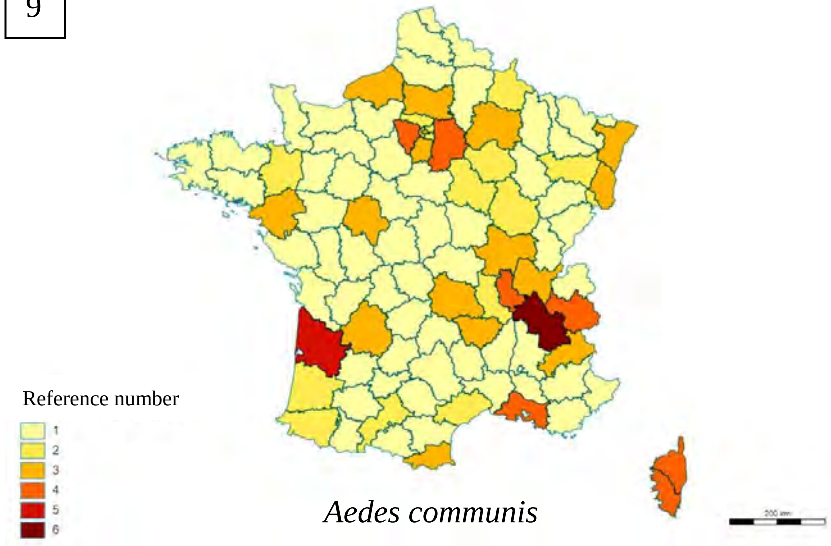

Aedes communis

10

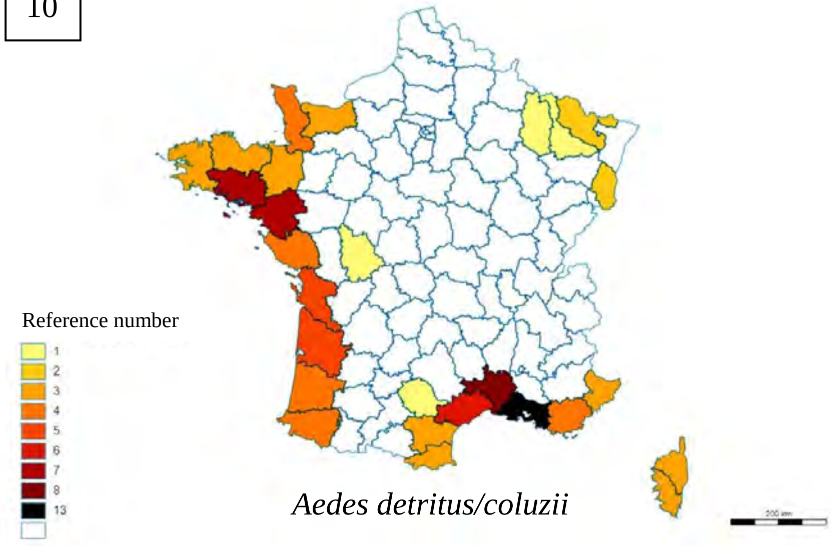

Aedes detritus/coluzii

11

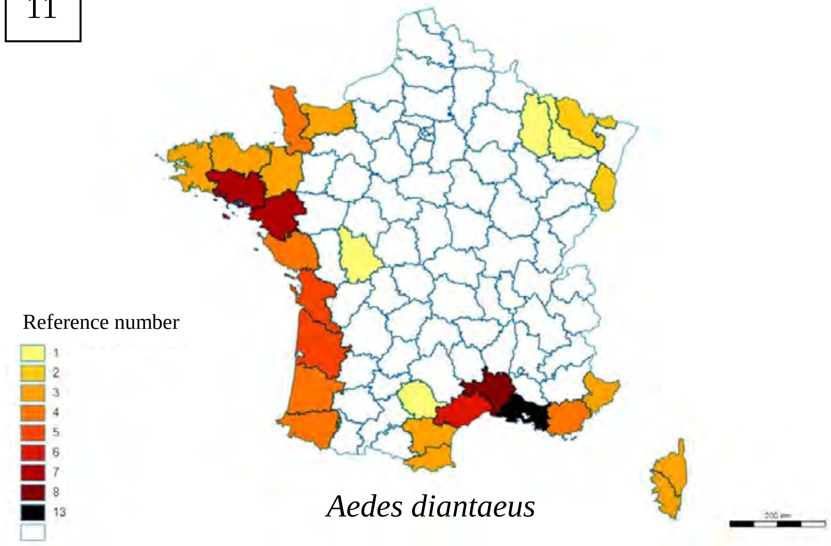

Aedes diantaeus

12

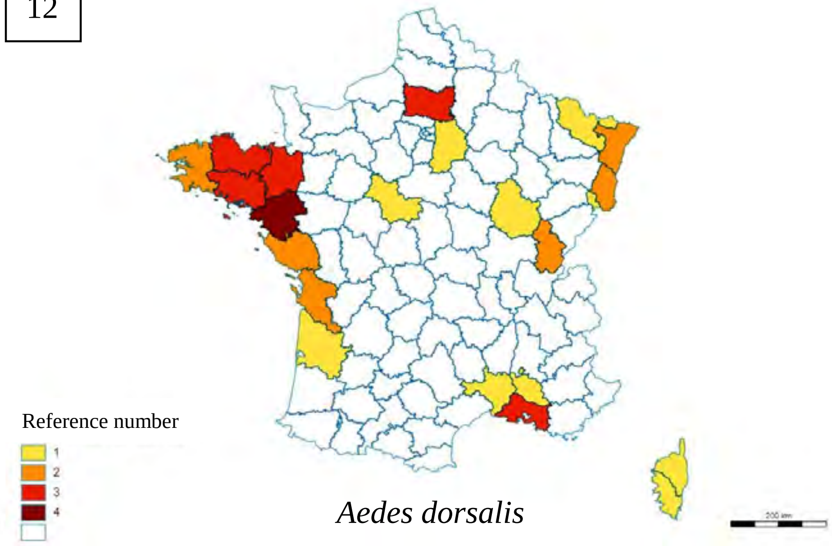

Aedes dorsalis

13

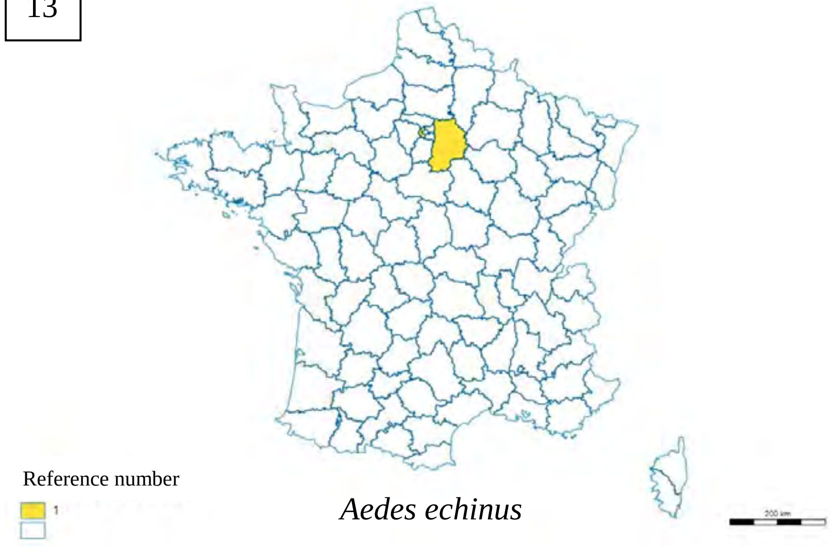
Reference number
1
Aedes echinus

14

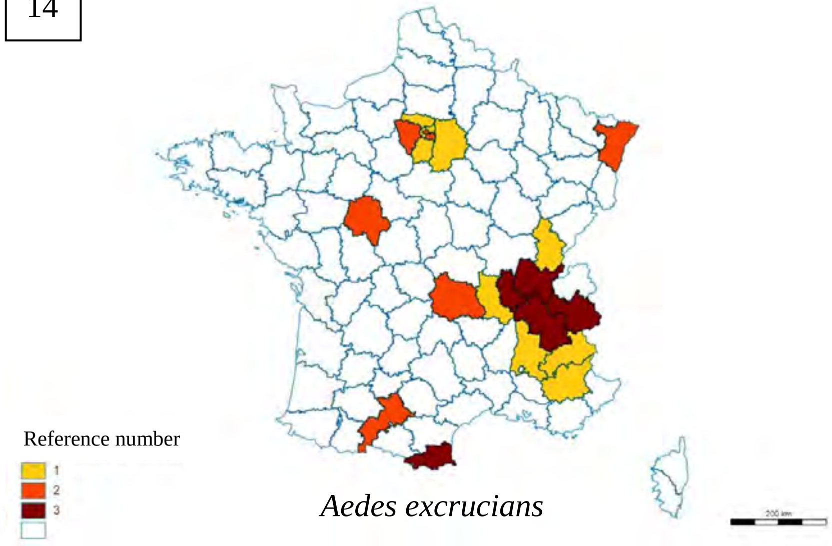
Reference number
1
2
3
Aedes excrucians

15

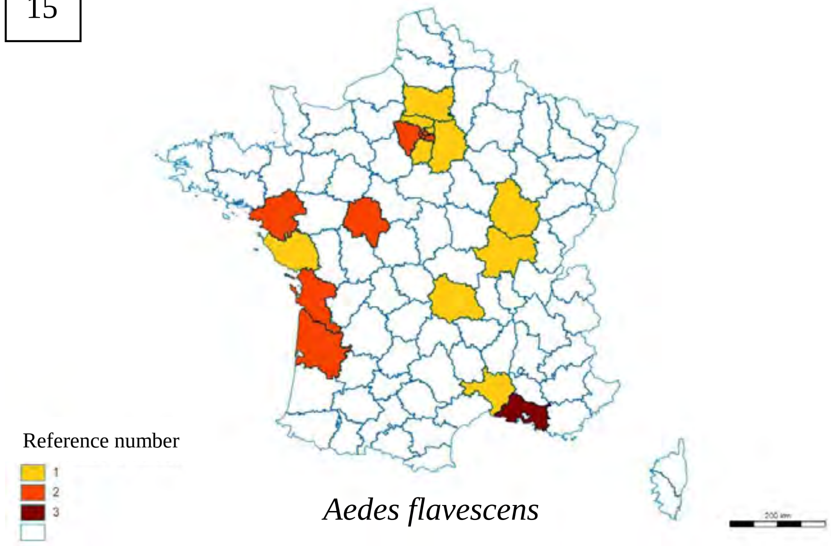
Reference number
1
2
3
Aedes flavescens

16

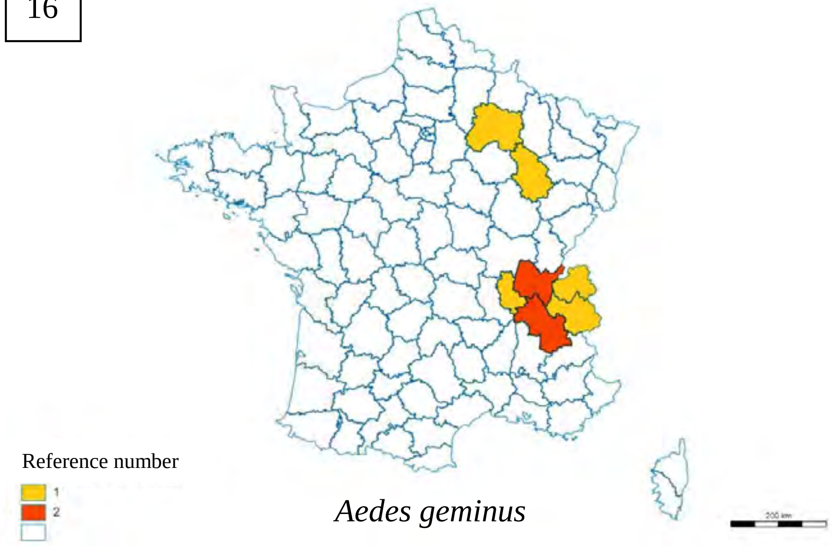
Reference number
1
2
Aedes geminus

17

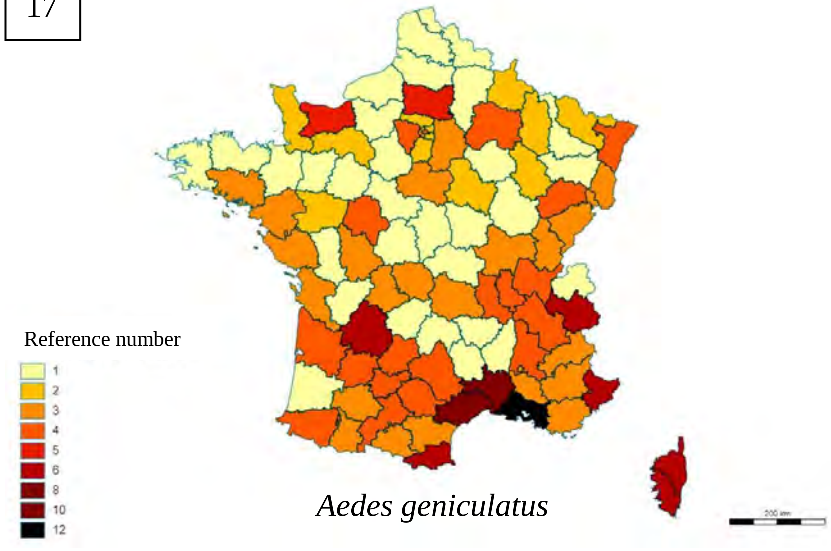

Aedes geniculatus

18

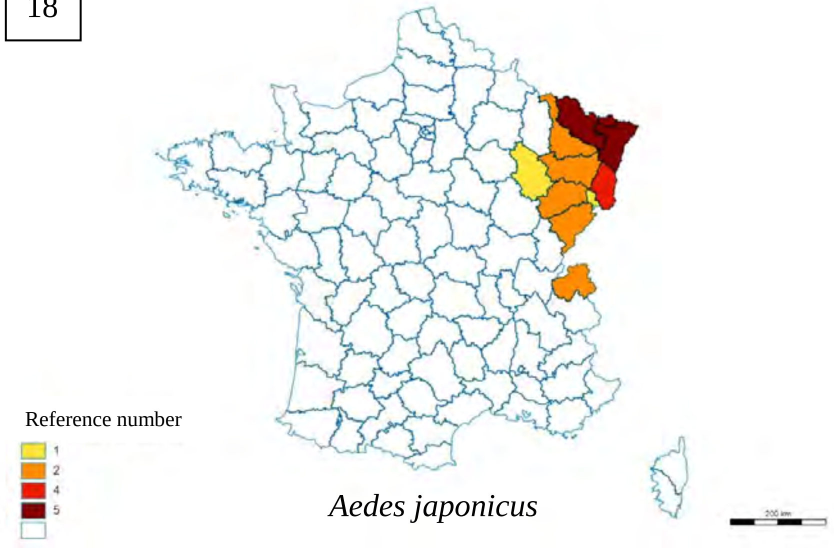

Aedes japonicus

19

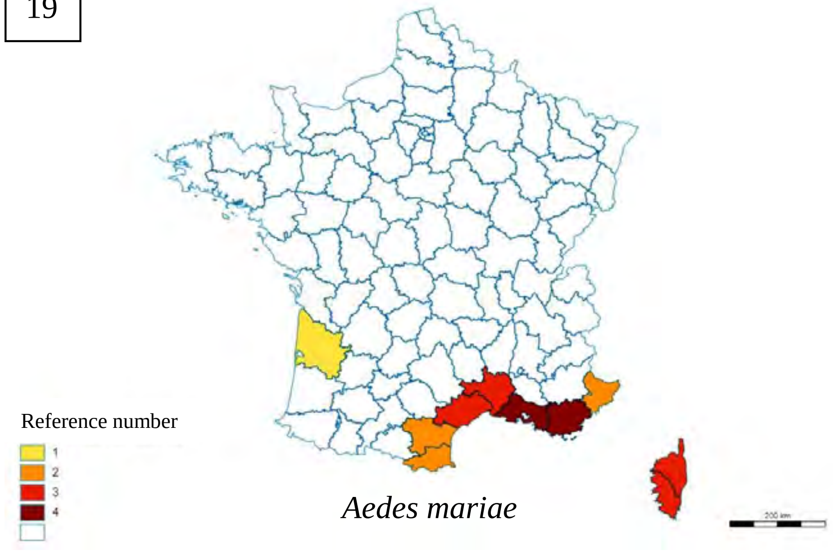

Aedes mariae

20

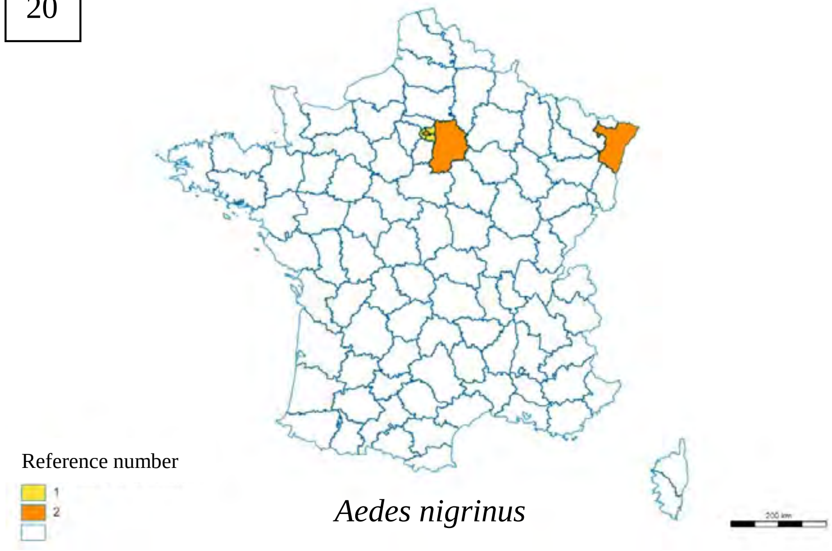

Aedes nigrinus

21

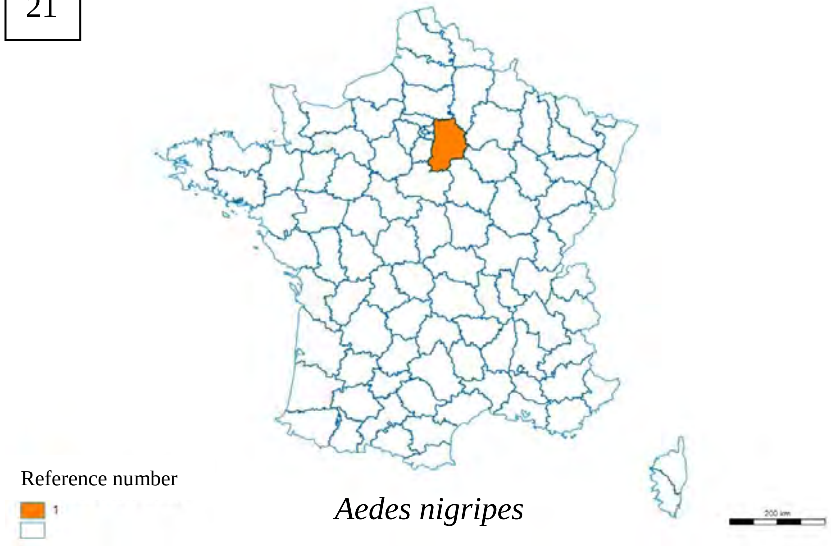
Reference number
1
Aedes nigripes

22

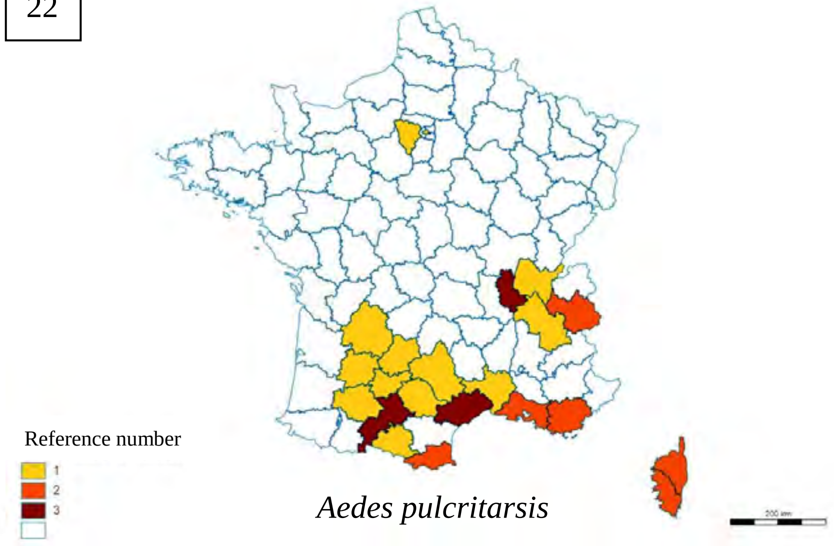
Reference number
1
2
3
Aedes pulcritarsis

23

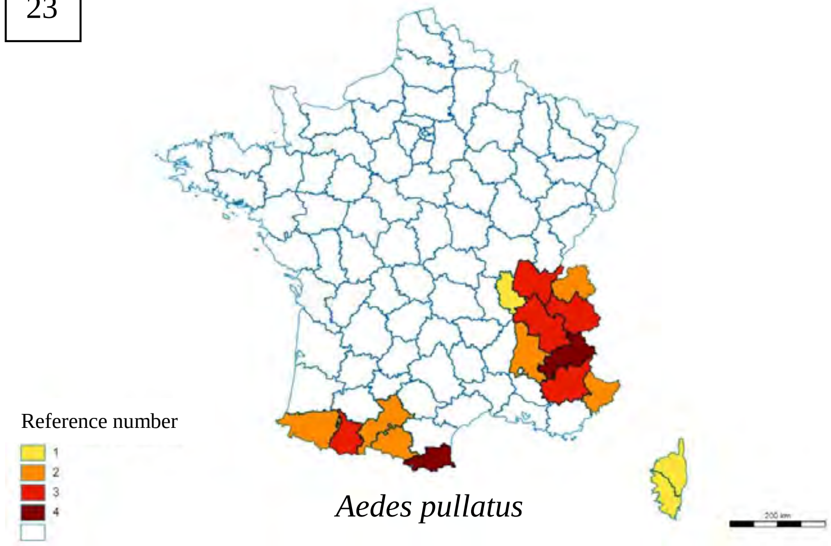
Reference number
1
2
3
4
Aedes pullatus

24

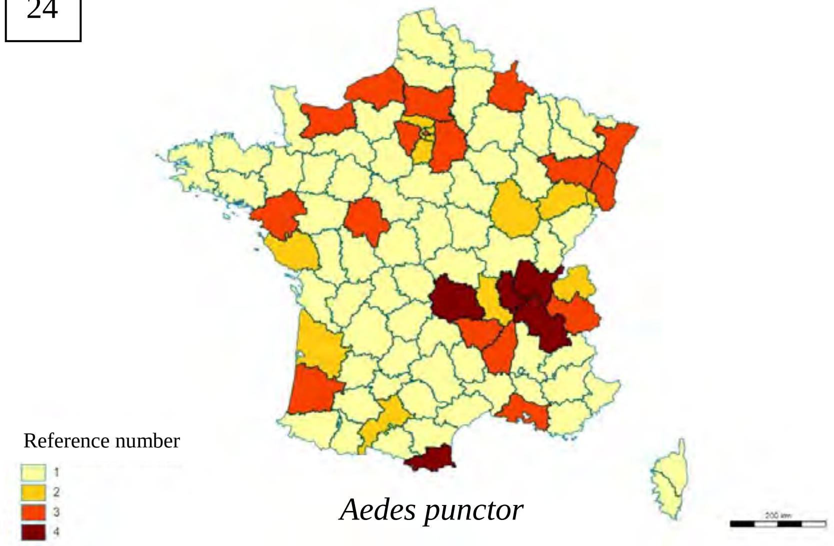
Reference number
1
2
3
4
Aedes punctor

25

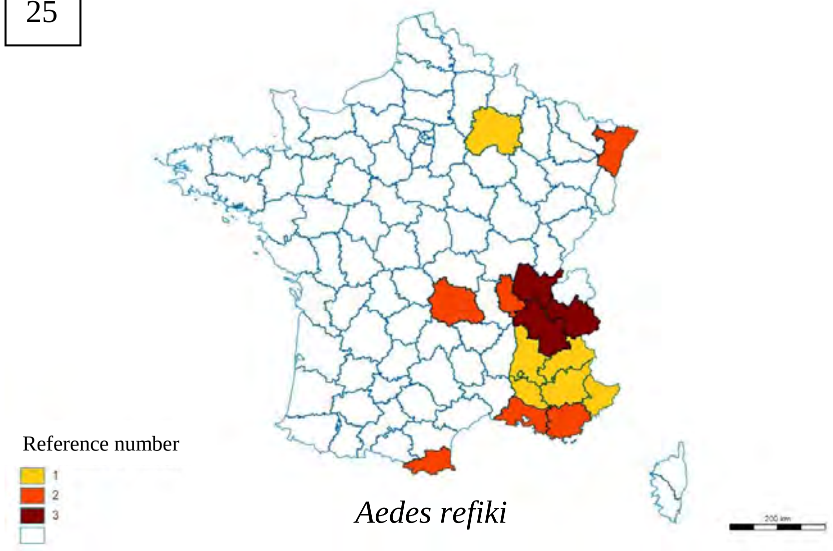

Aedes refiki

26

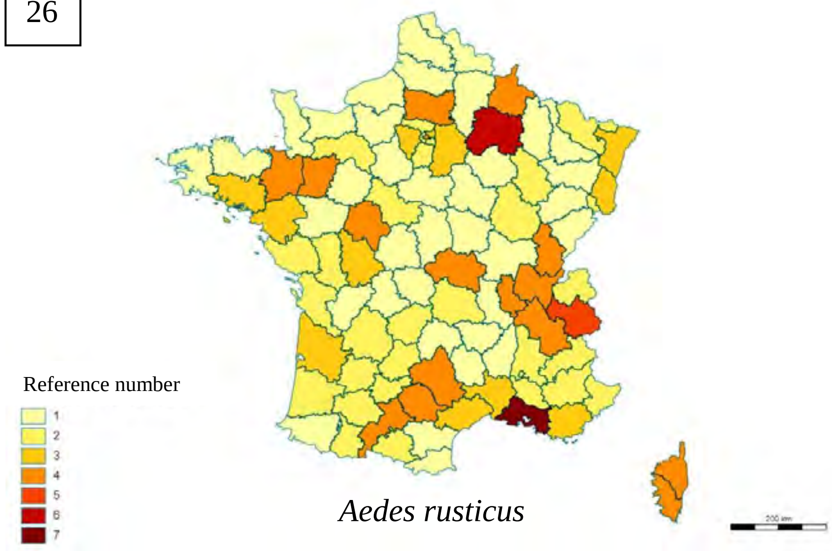

Aedes rusticus

27

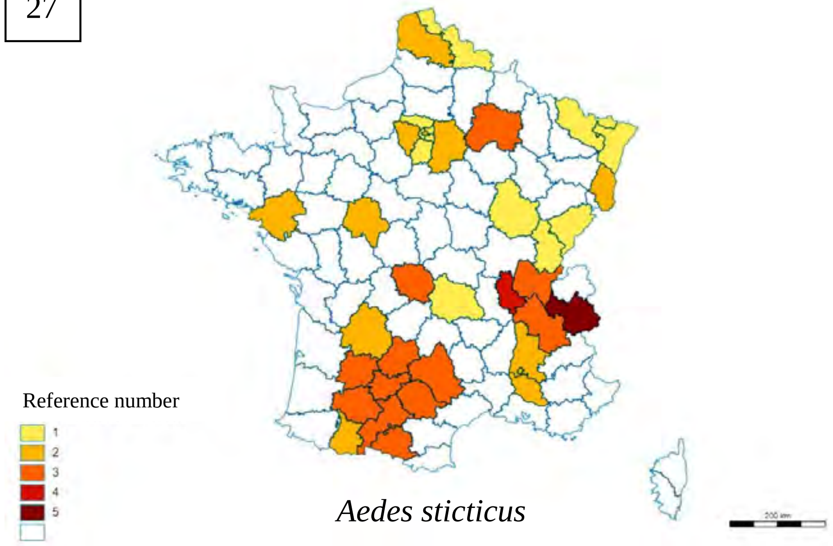

Aedes sticticus

28

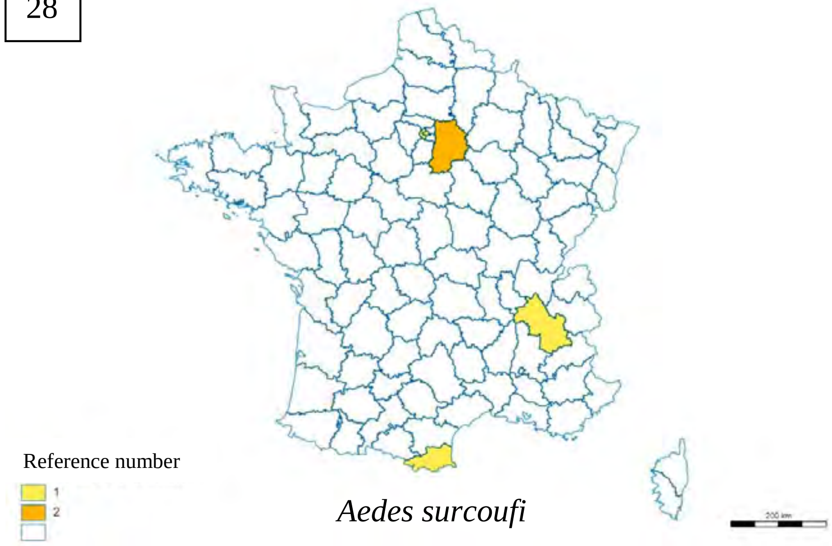

Aedes surcoufi

29

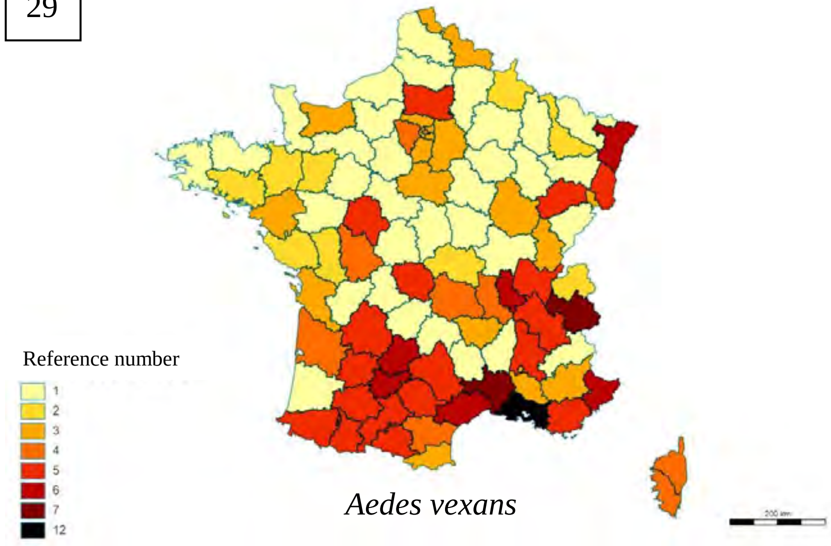

Aedes vexans

30

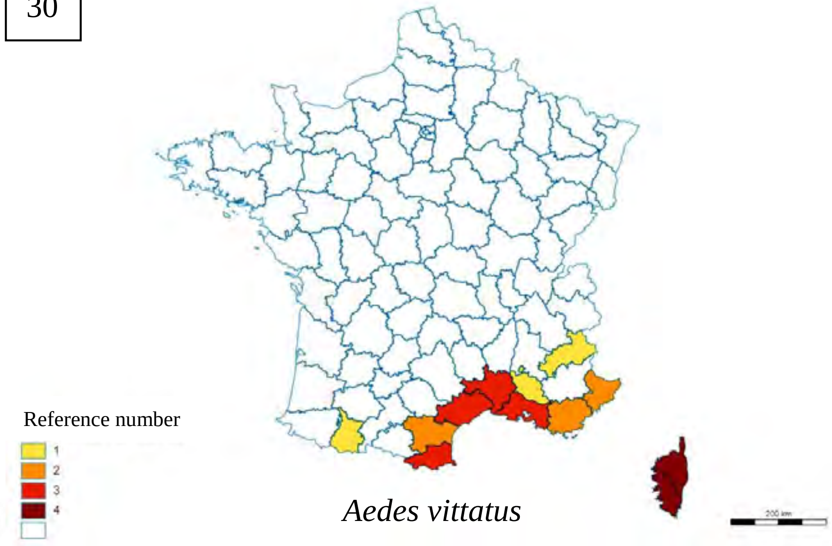

Aedes vittatus

31

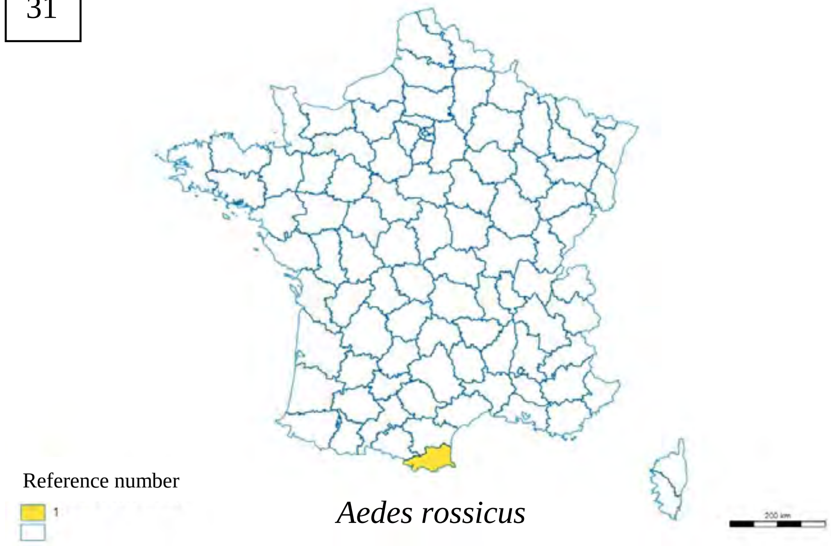

Aedes rossicus

32

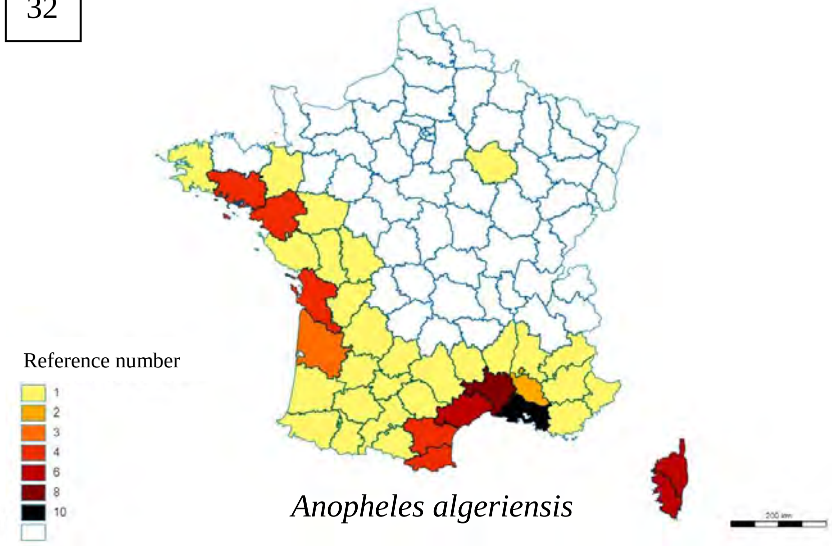

Anopheles algeriensis

33

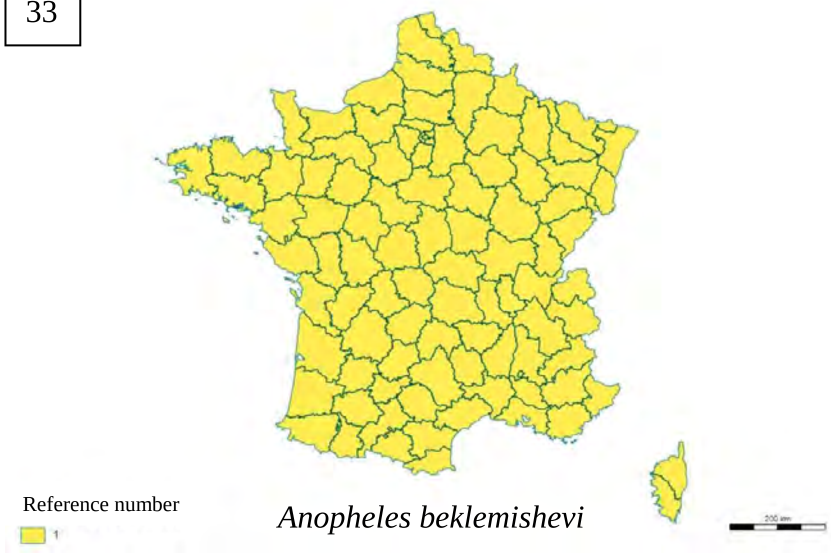

Anopheles beklemishevi

34

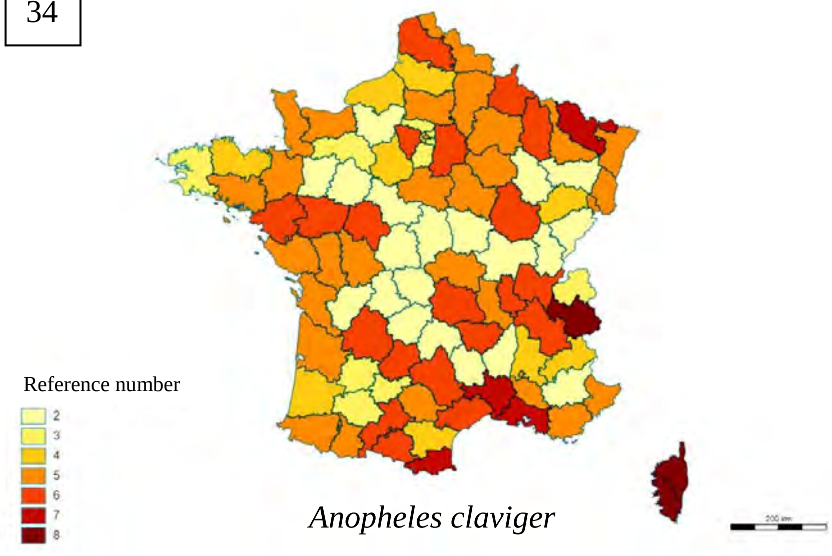

Anopheles claviger

35

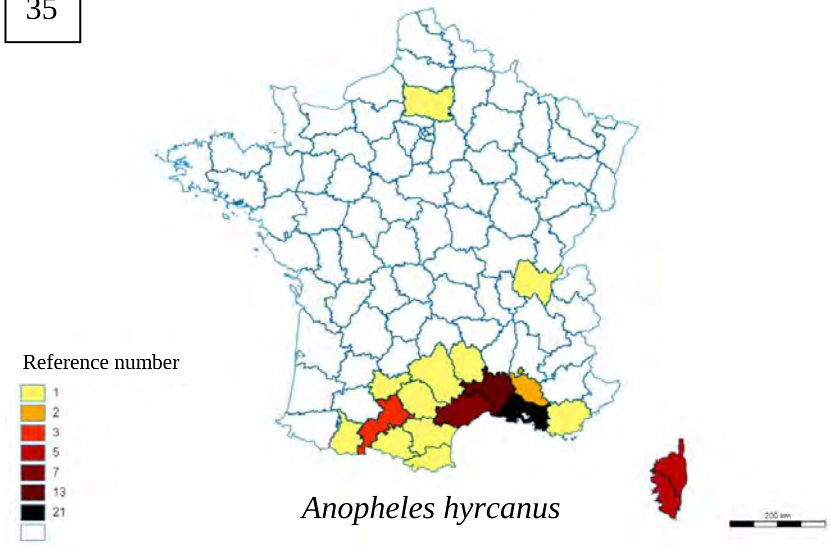

Anopheles hyrcanus

36

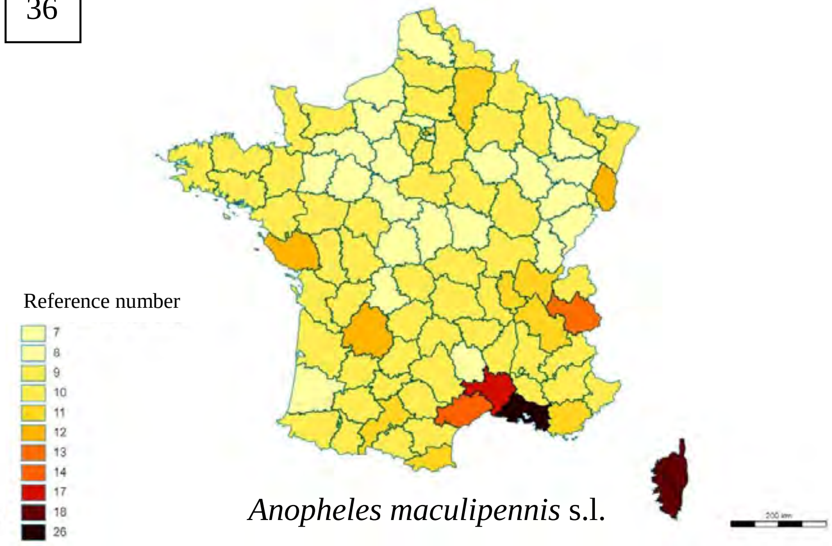

Anopheles maculipennis s.l.

37

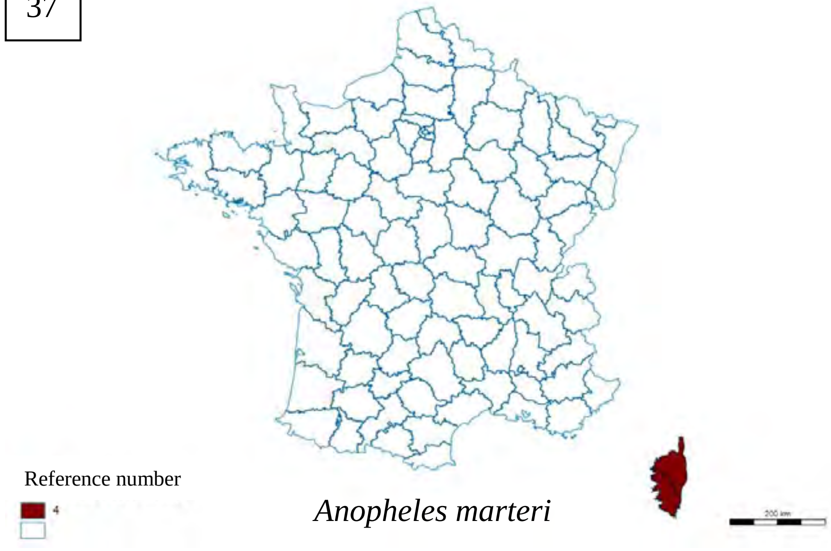
Reference number
4
Anopheles marteri

38

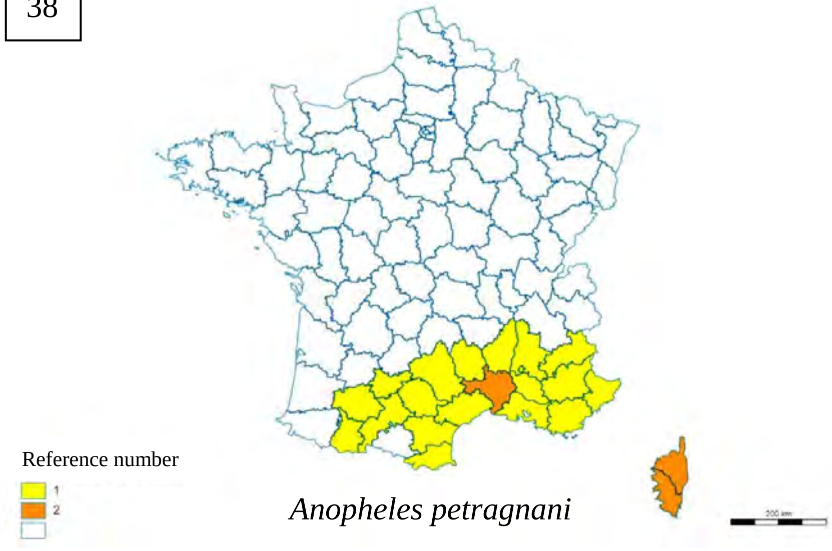
Reference number
1
2
Anopheles petragnani

39

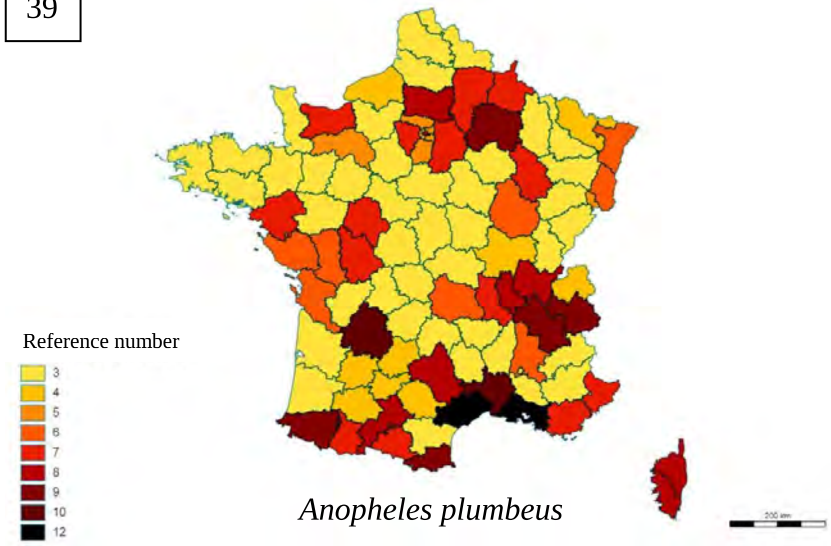
Reference number
3
4
5
6
7
8
9
10
12
Anopheles plumbeus

40

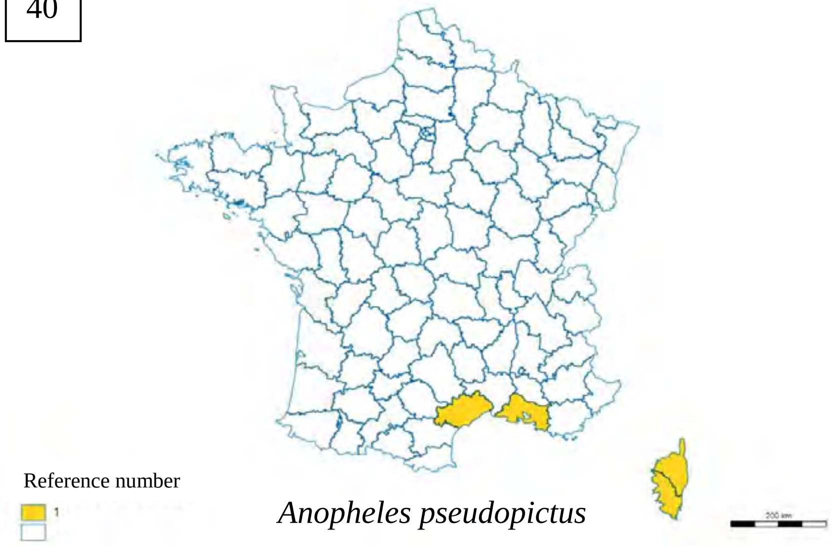
Reference number
1
Anopheles pseudopictus

41

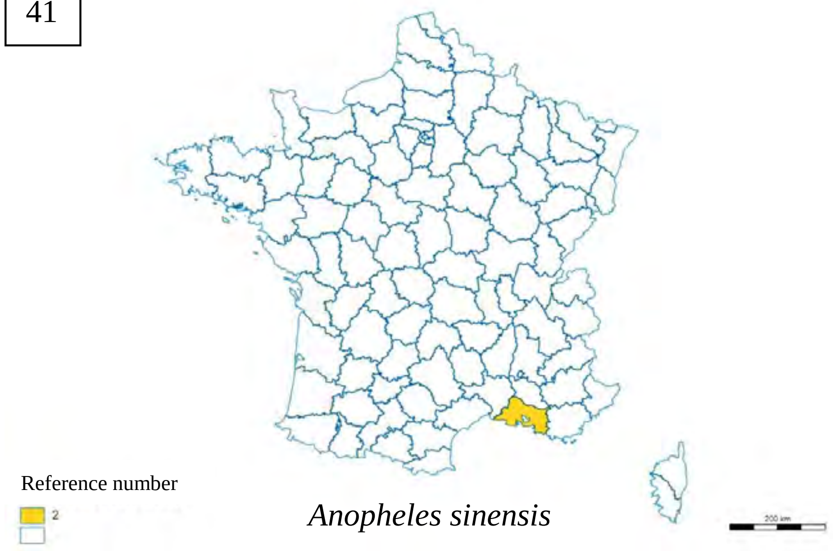

Anopheles sinensis

42

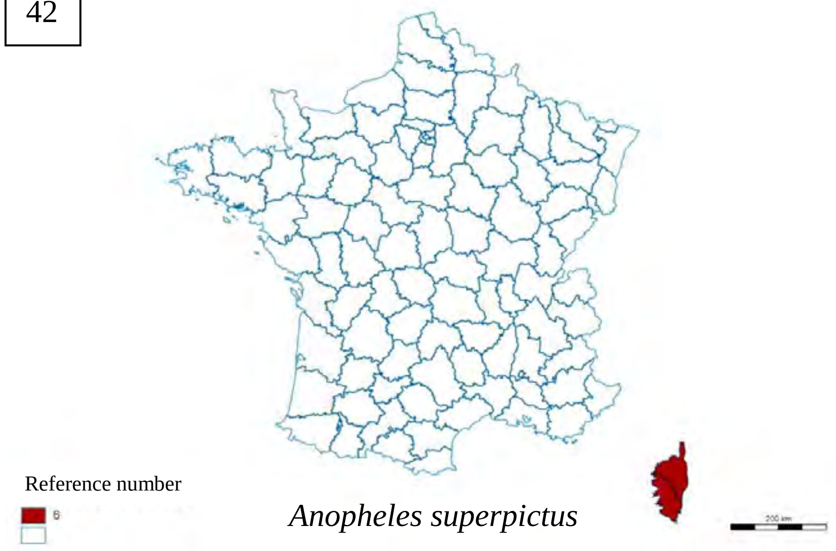

Anopheles superpictus

43

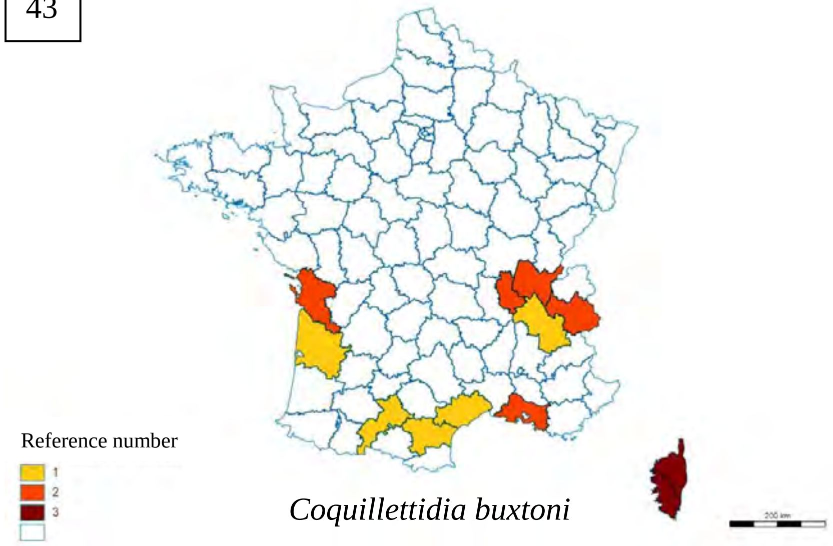

Coquillettidia buxtoni

44

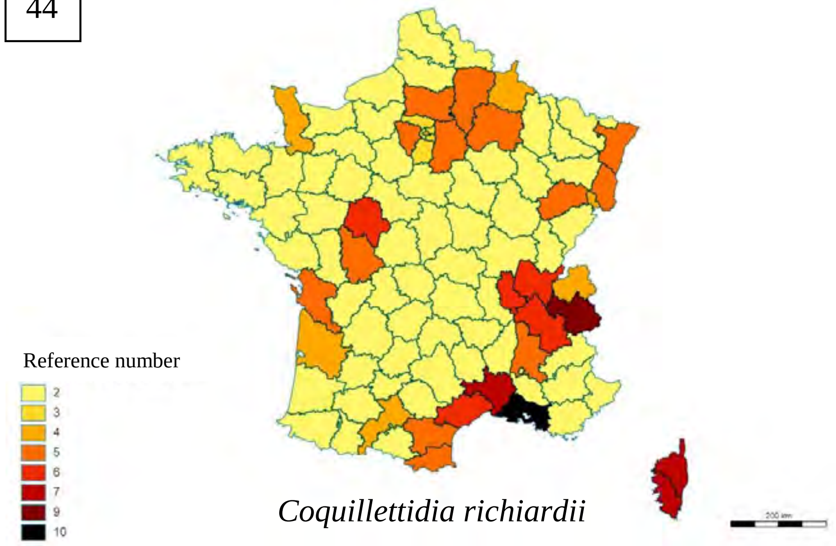

Coquillettidia richiardii

45

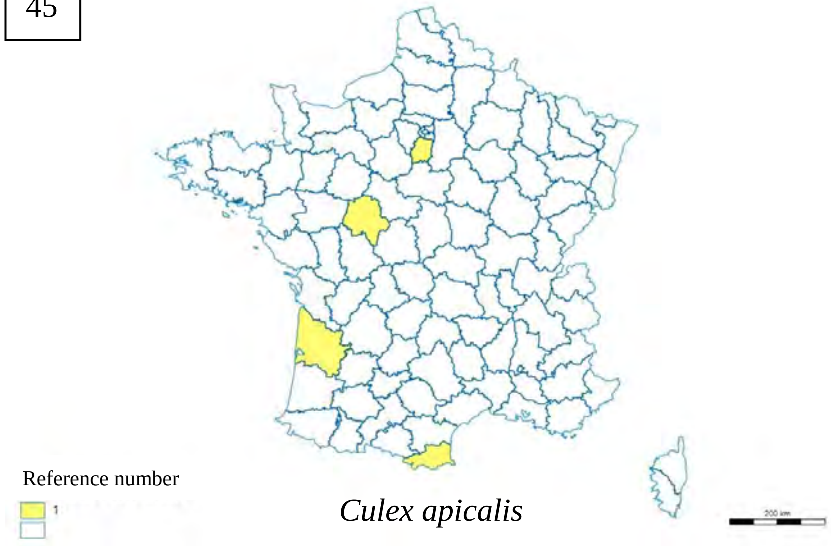

Culex apicalis

46

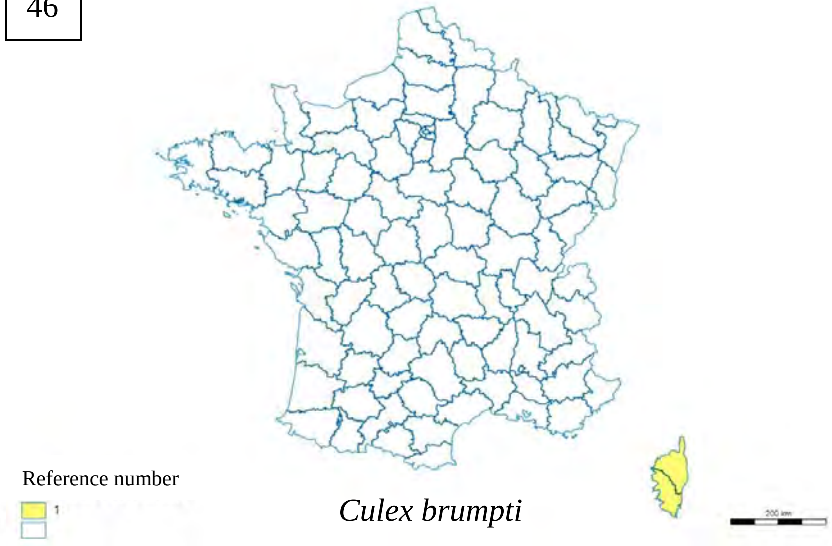

Culex brumpti

47

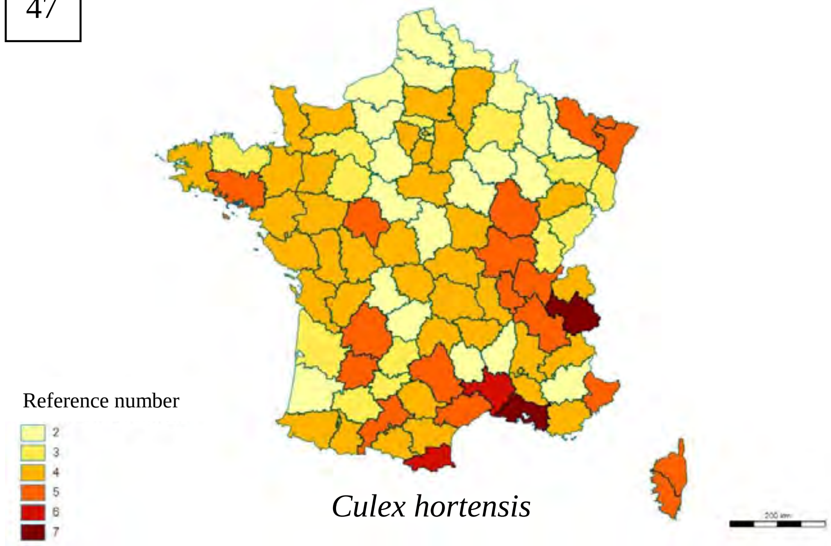

Culex hortensis

48

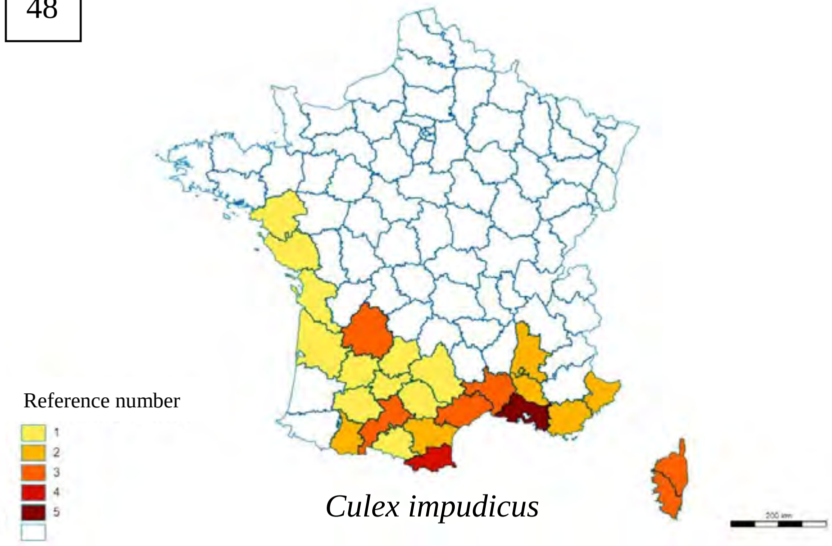

Culex impudicus

49

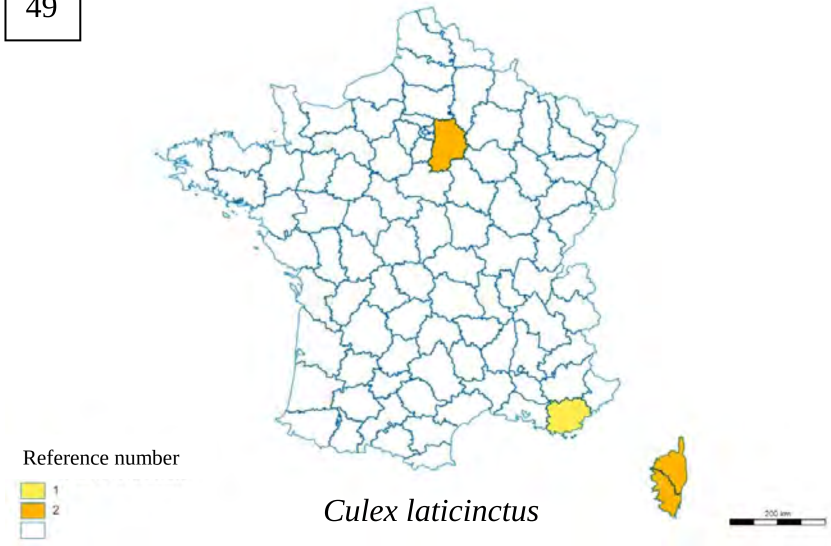

Culex laticinctus

50

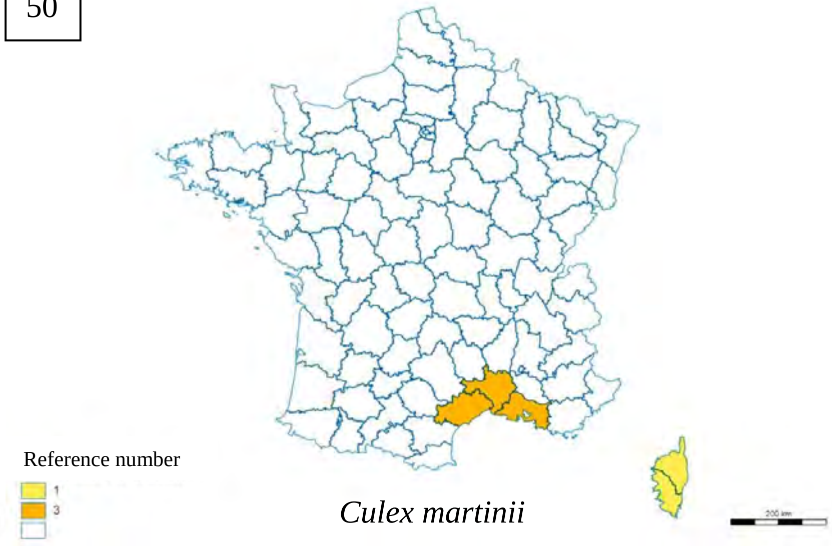

Culex martinii

51

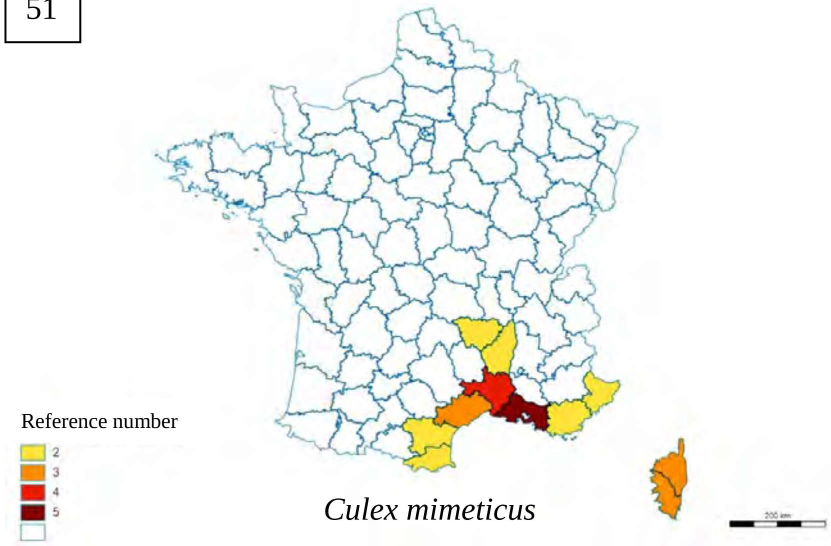

Culex mimeticus

52

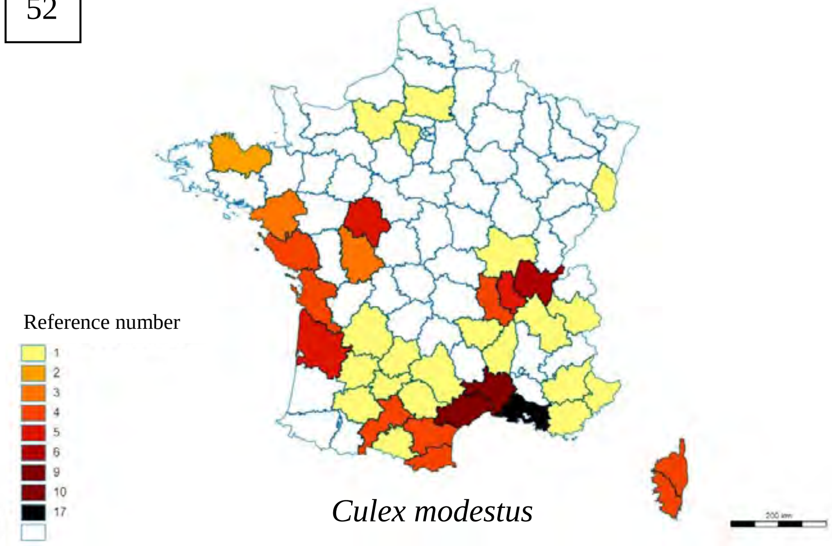

Culex modestus

53

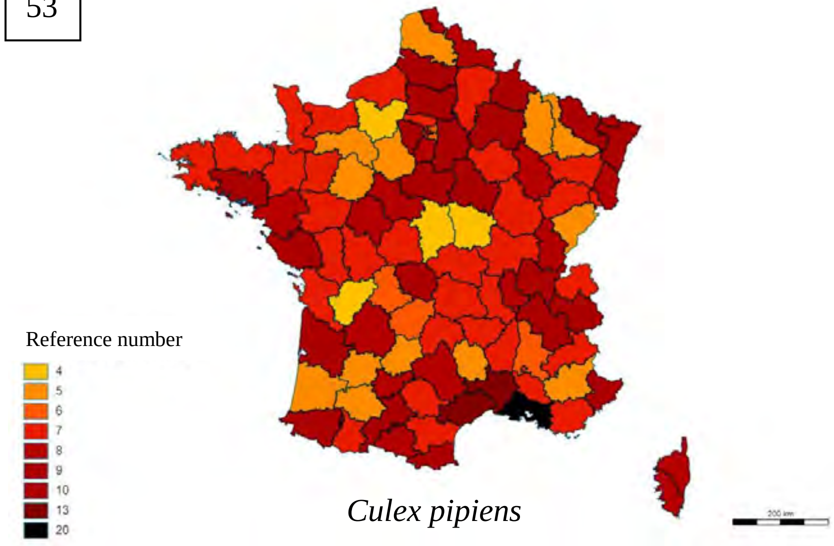

Culex pipiens

54

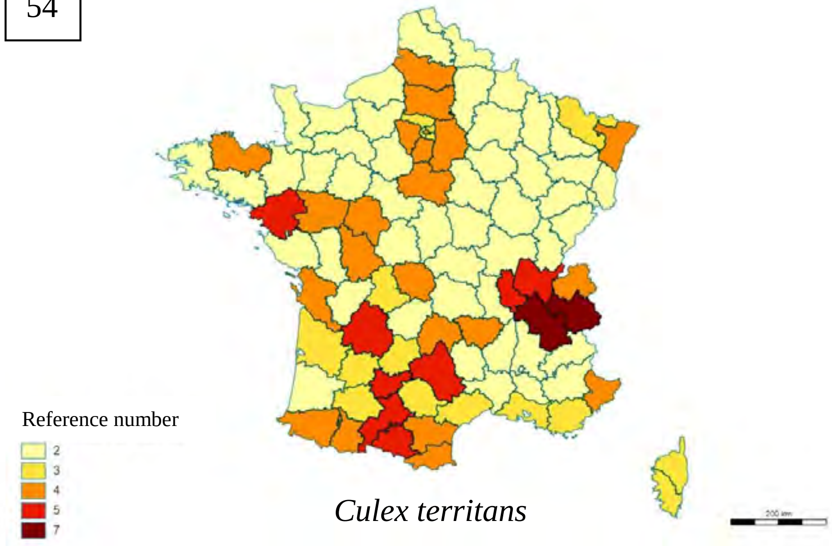

Culex territans

55

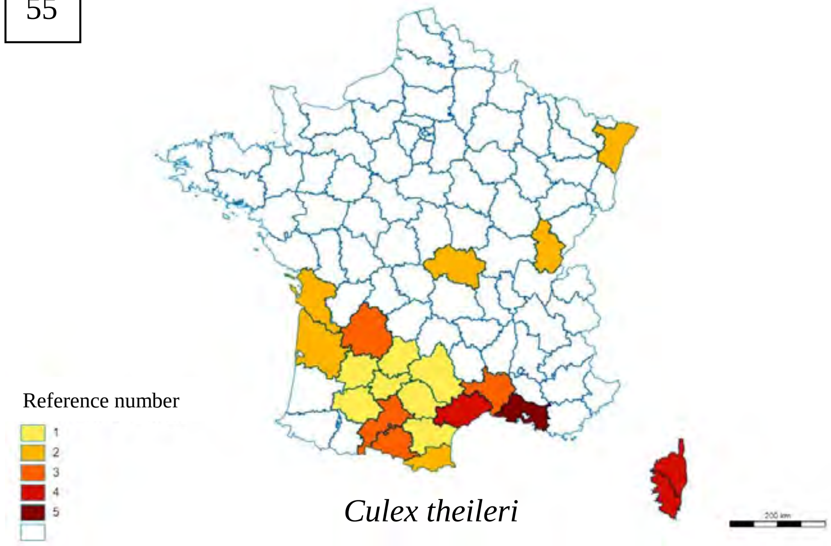

Culex theileri

56

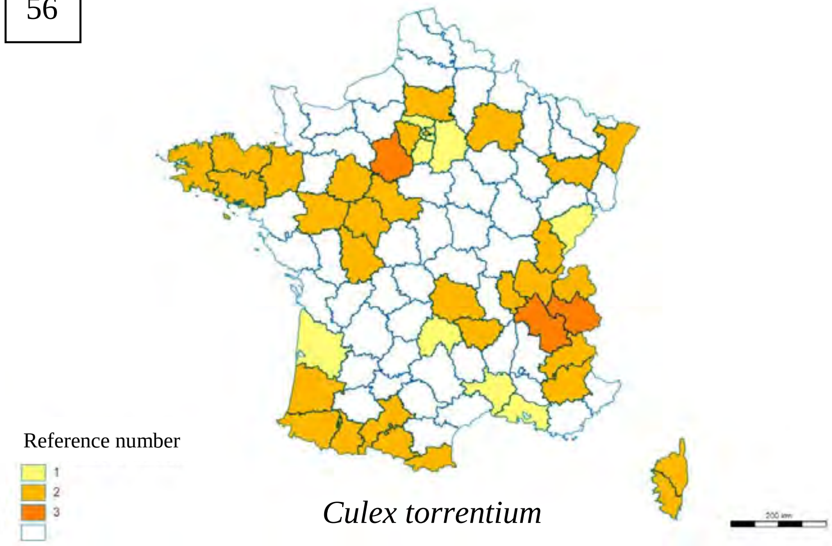

Culex torrentium

57

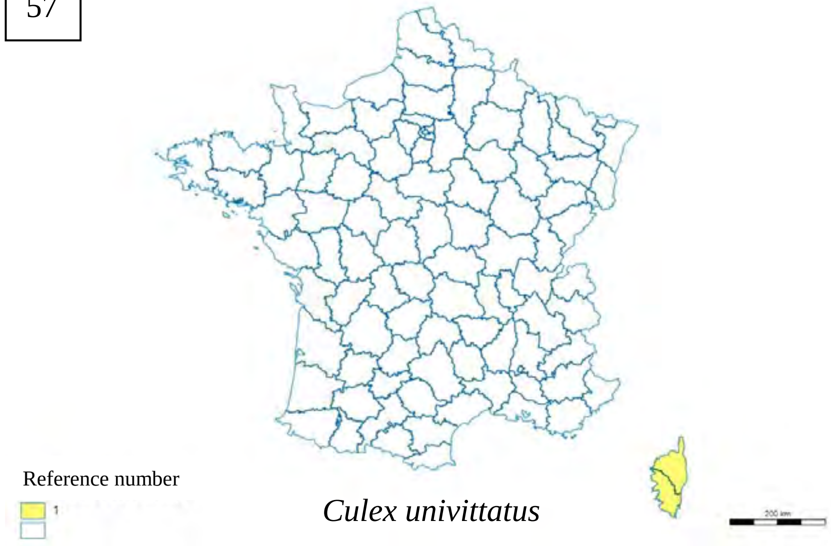

Culex univittatus

58

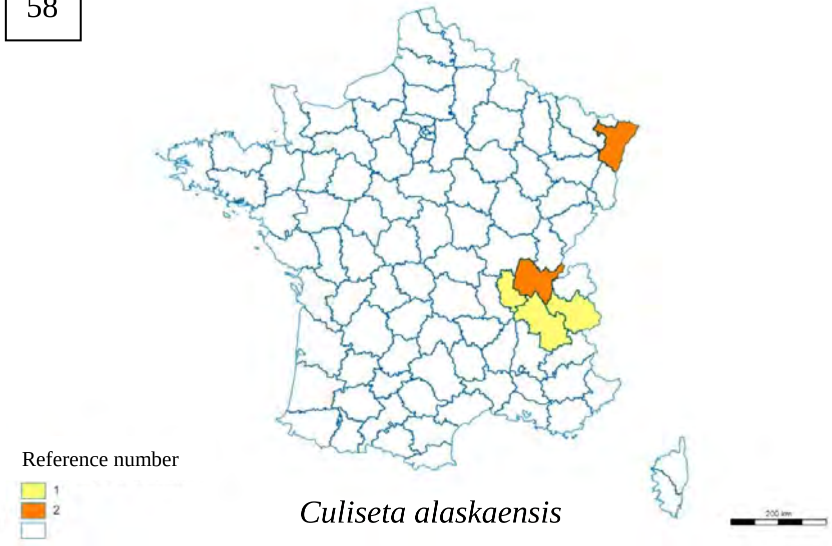

Culiseta alaskaensis

59

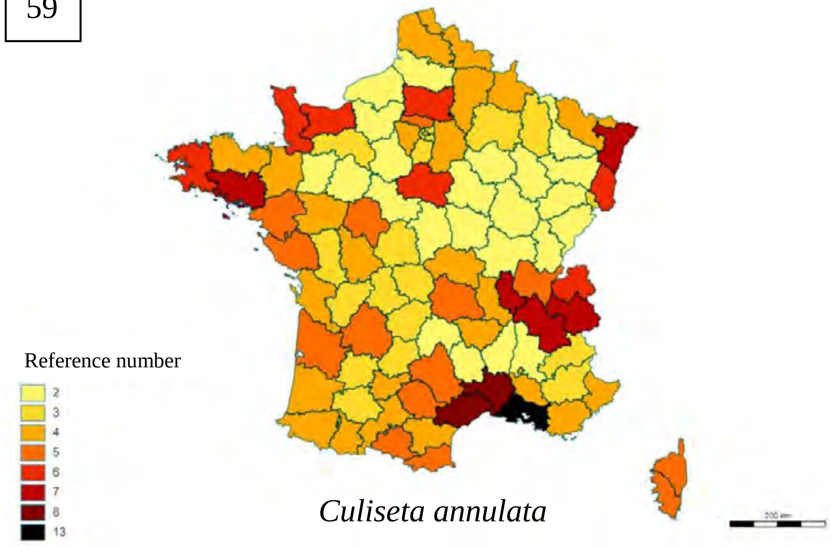

Culiseta annulata

60

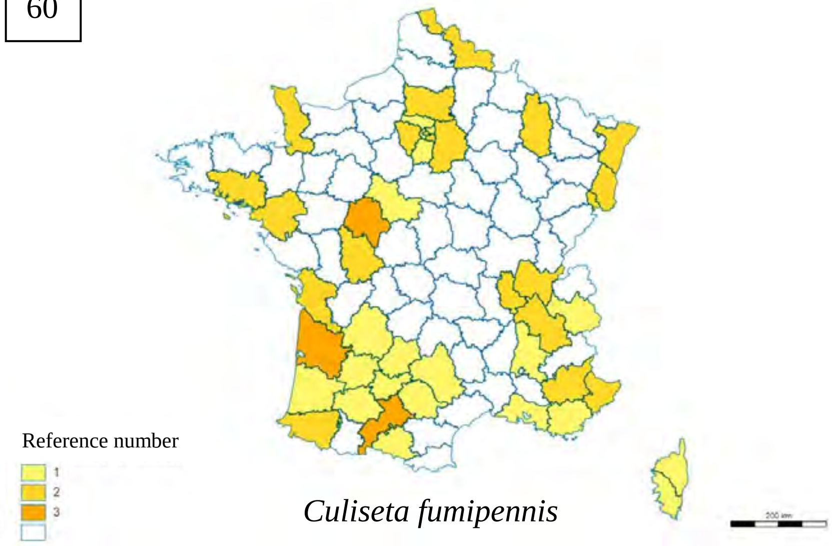

Culiseta fumipennis

61

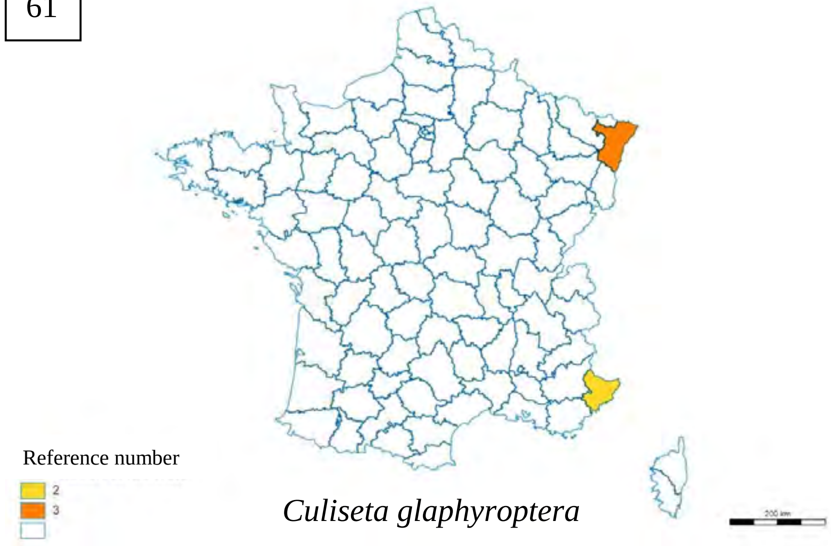

Culiseta glaphyroptera

62

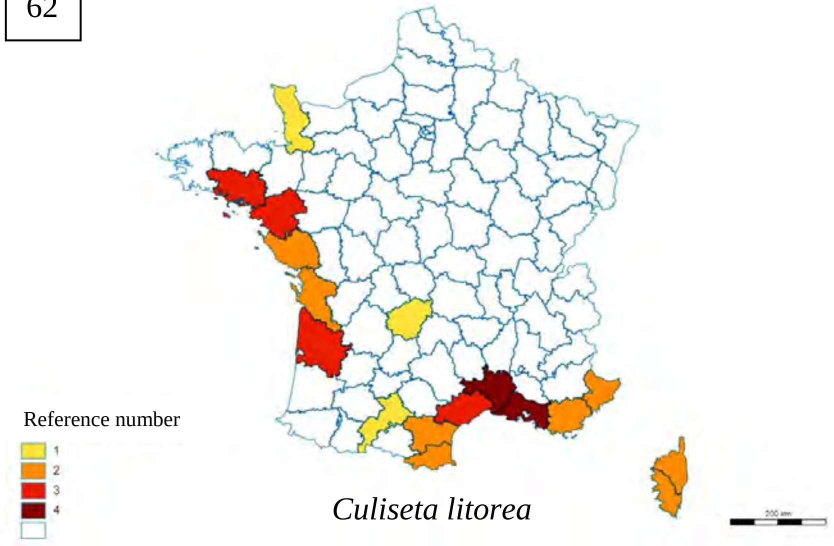

Culiseta litorea

63

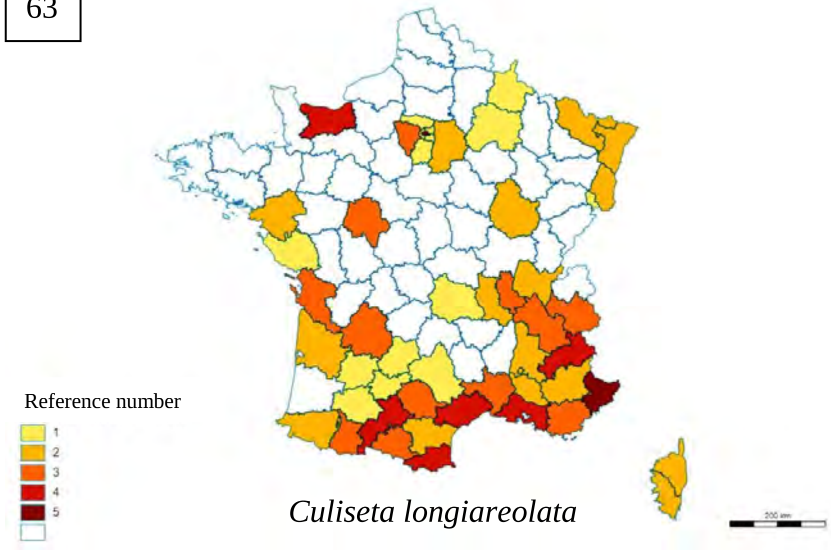

Culiseta longiareolata

64

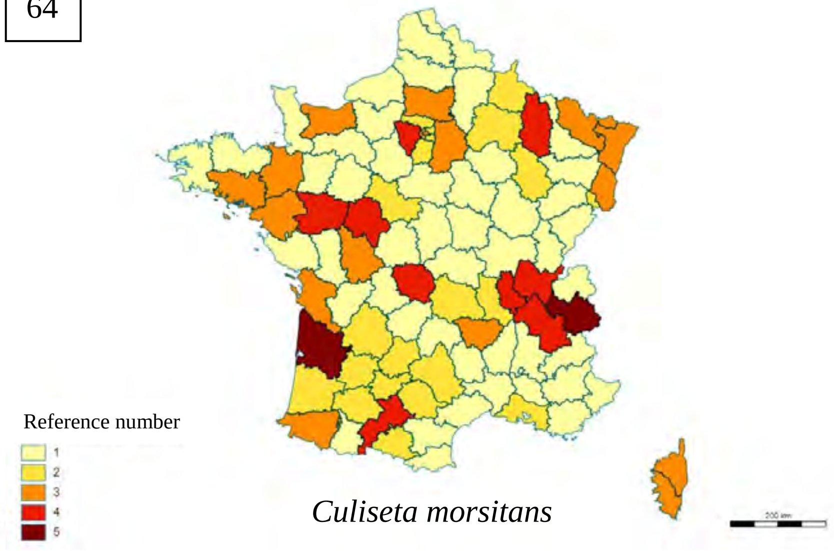

Culiseta morsitans

65

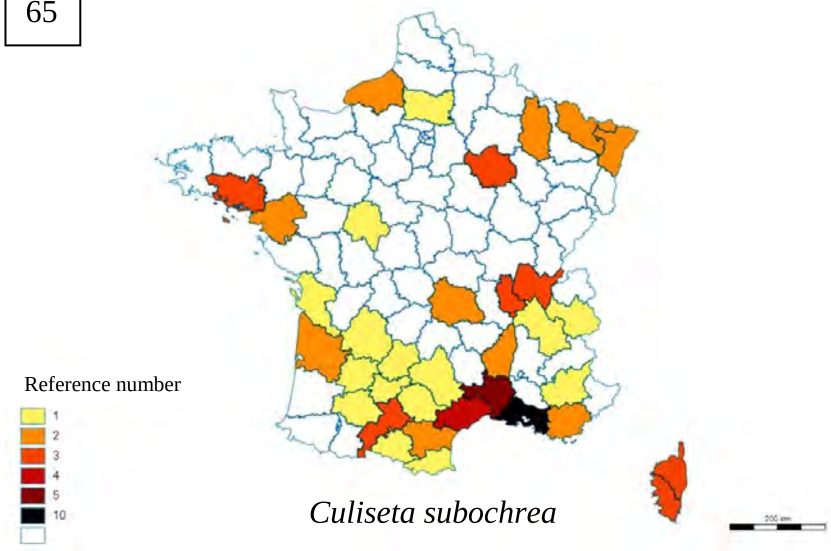

Culiseta subochrea

66

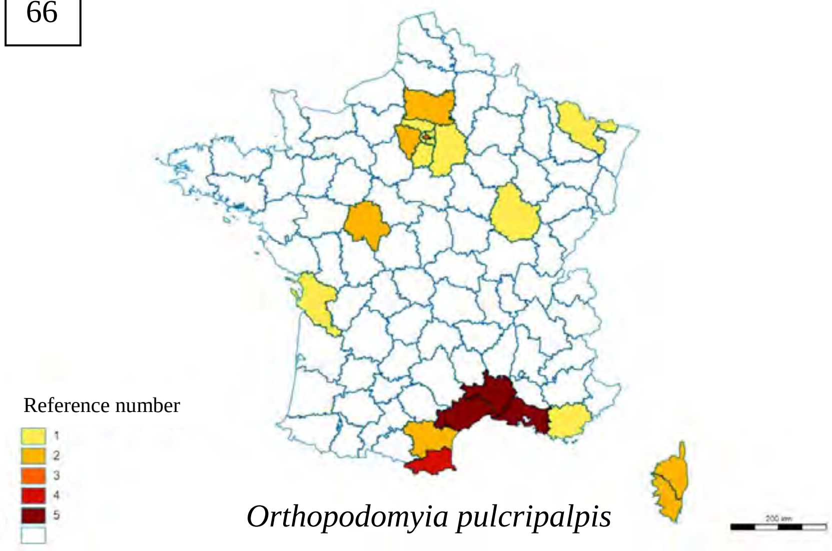

Orthopodomyia pulcripalpis

67

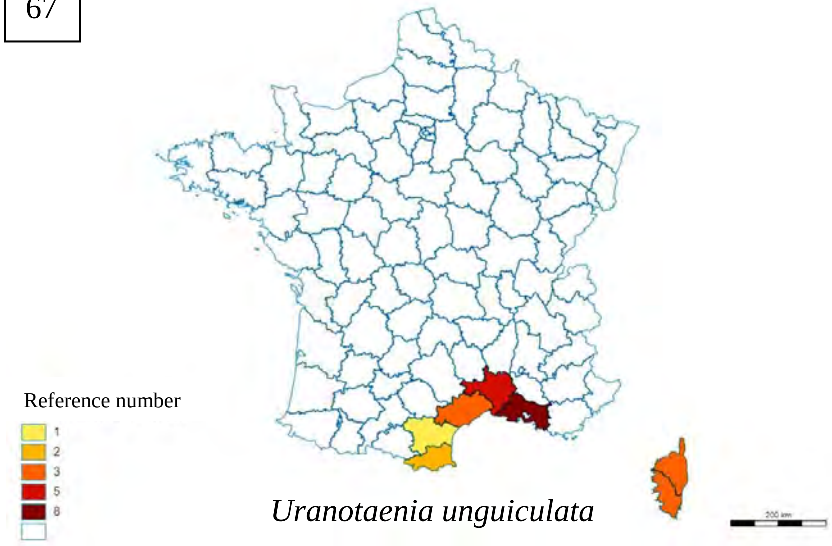

Uranotaenia unguiculata

Supplementary Figure S8. Distribution map of *Aedes aegypti* (1), *Aedes albopictus* (2), *Aedes annulipes* (3), *Aedes berlandi* (4), *Aedes cantans* (5), *Aedes caspius* (6), *Aedes cataphylla* (7), *Aedes cinereus* (8), *Aedes communis* (9), *Aedes detritus/coluzii* (10), *Aedes diantaeus* (11), *Aedes dorsalis* (12), *Aedes echinus* (13), *Aedes excrucians* (14), *Aedes flavescens* (15), *Aedes geminus* (16), *Aedes geniculatus* (17), *Aedes japonicus* (18), *Aedes mariae* (19), *Aedes nigrinus* (20), *Aedes nigripes* (21), *Aedes pulcritarsis* (22), *Aedes pullatus* (23), *Aedes punctor* (24), *Aedes refiki* (25), *Aedes rusticus* (26), *Aedes sticticus* (27), *Aedes surcoufi* (28), *Aedes vexans* (29), *Aedes vittatus* (30), *Aedes rossicus* (31), *Anopheles algeriensis* (32), *Anopheles beklemishevi* (33), *Anopheles claviger* (34), *Anopheles hyrcanus* (35), *Anopheles maculipennis* s.l. (36), *Anopheles marteri* (37), *Anopheles petragnani* (38), *Anopheles plumbeus* (39), *Anopheles pseudopictus* (40), *Anopheles sinensis* (41), *Anopheles superpictus* (42), *Coquillettidia buxtoni* (43), *Coquillettidia richiardii* (44), *Culex apicalis* (45), *Culex brumpti* (46), *Culex hortensis* (47), *Culex impudicus* (48), *Culex laticinctus* (49), *Culex martinii* (50), *Culex mimeticus* (51), *Culex modestus* (52), *Culex pipiens* (53), *Culex territans* (54), *Culex theileri* (55), *Culex torrentium* (56), *Culex univittatus* (57), *Culiseta alaskaensis* (58), *Culiseta annulata* (59), *Culiseta fumipennis* (60), *Culiseta glaphyroptera* (61), *Culiseta litorea* (62), *Culiseta longiareolata* (63), *Culiseta morsitans* (64), *Culiseta subochrea* (65), *Orthopodomyia pulcripalpis* (66) and *Uranotaenia unguiculata* (67) by department according to the number of references.

1A

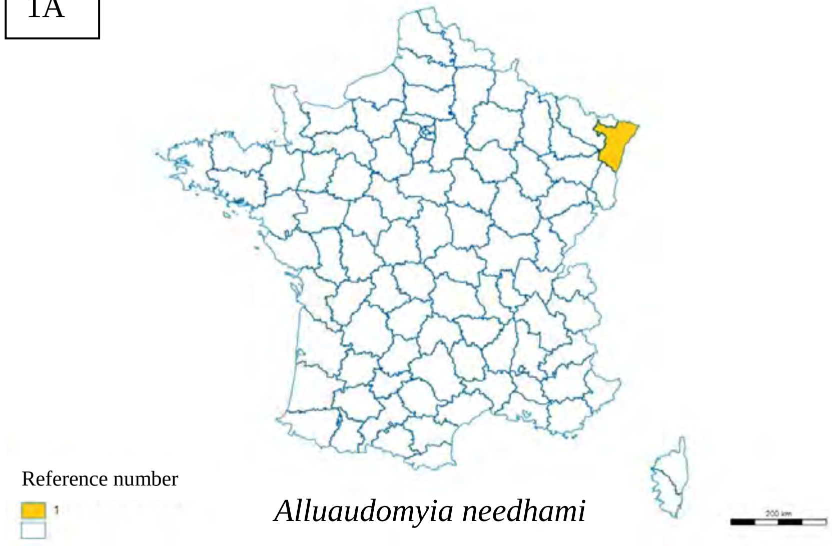

Alluaudomyia needhami

2A

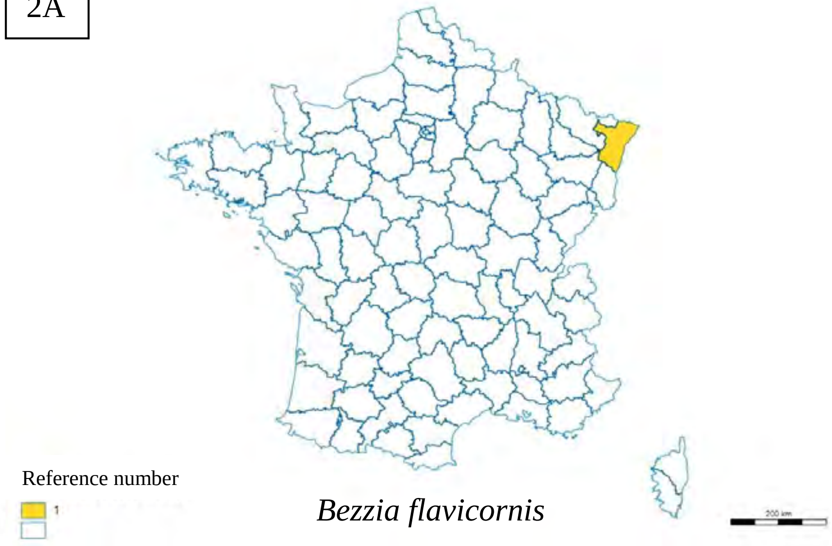

Bezzia flavicornis

3A

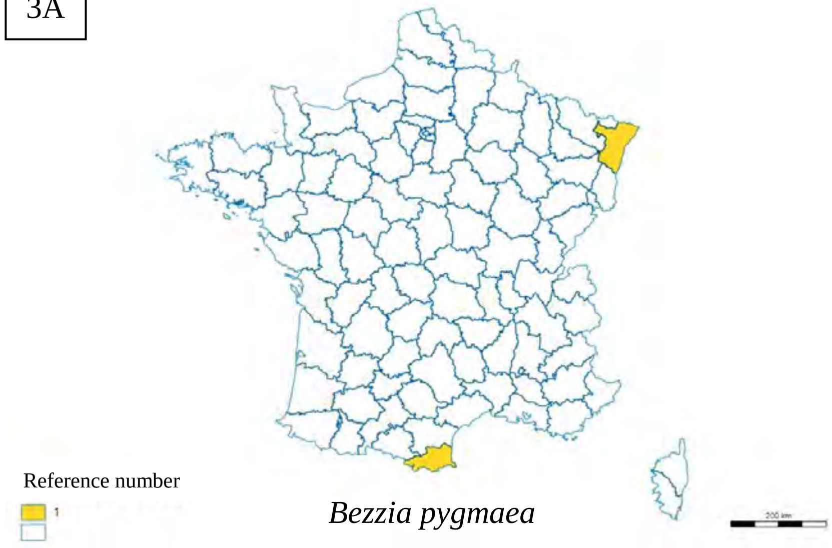

Bezzia pygmaea

4A

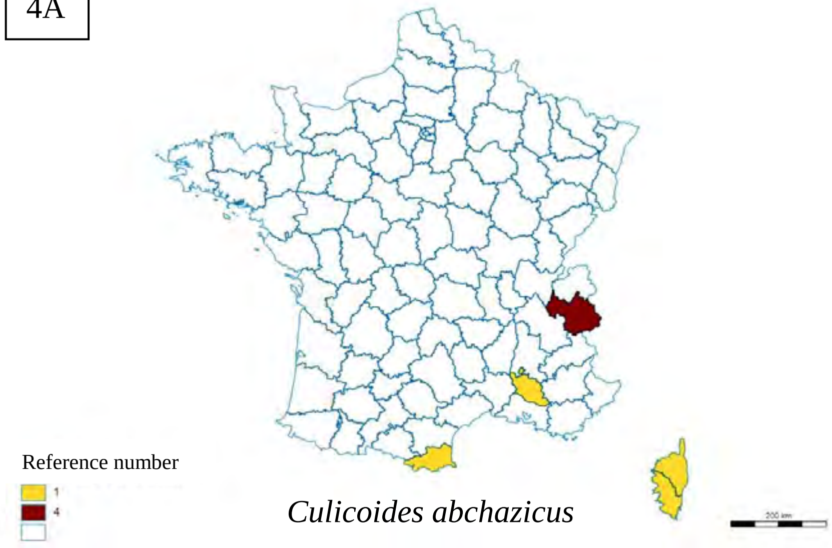

Culicoides abchazicus

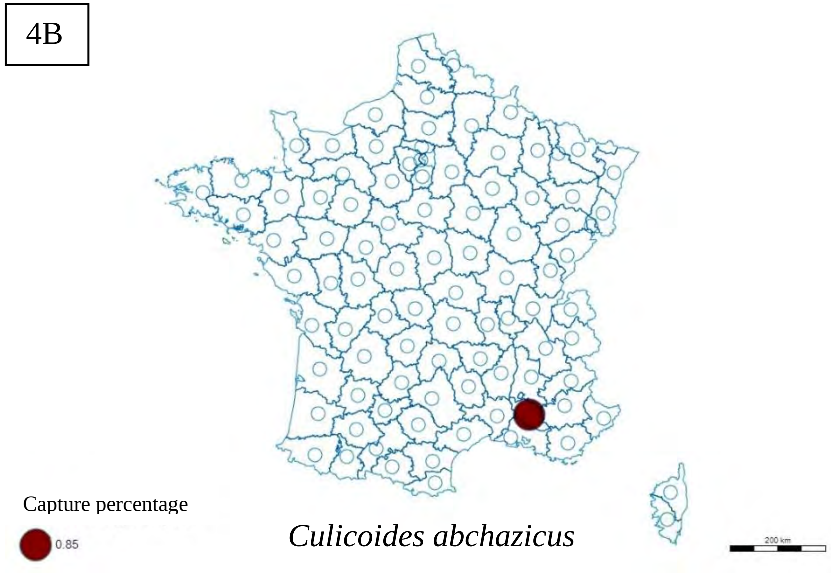
4B
Capture percentage
0.85
Culicoides abchazicus

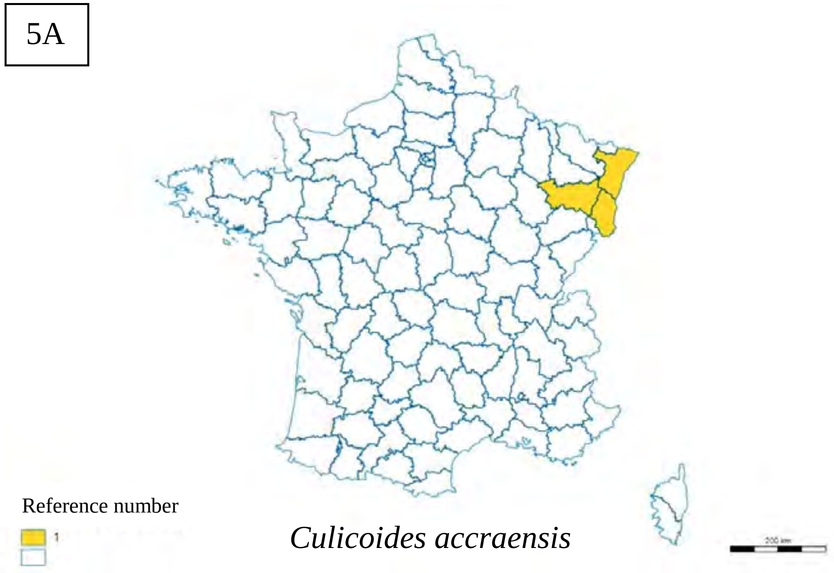
5A
Reference number
1
Culicoides accraensis

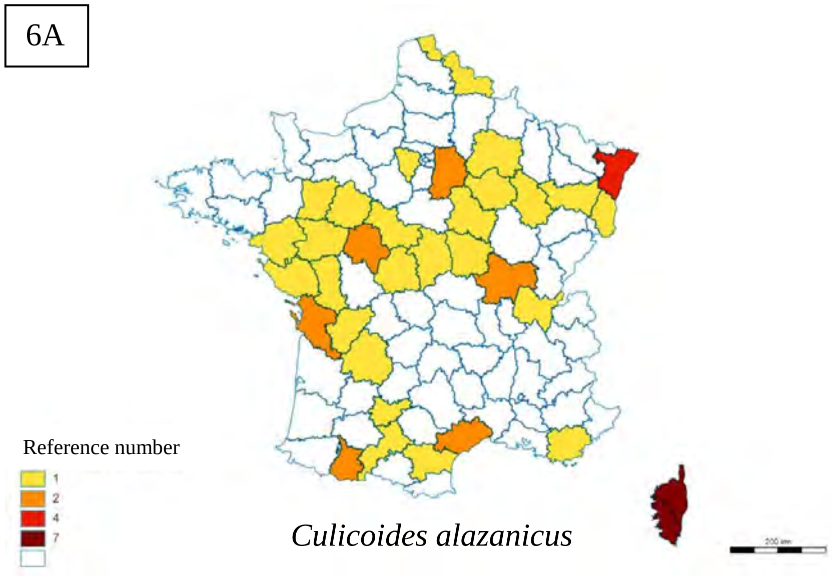
6A
Reference number
1
2
4
7
Culicoides alazanicus

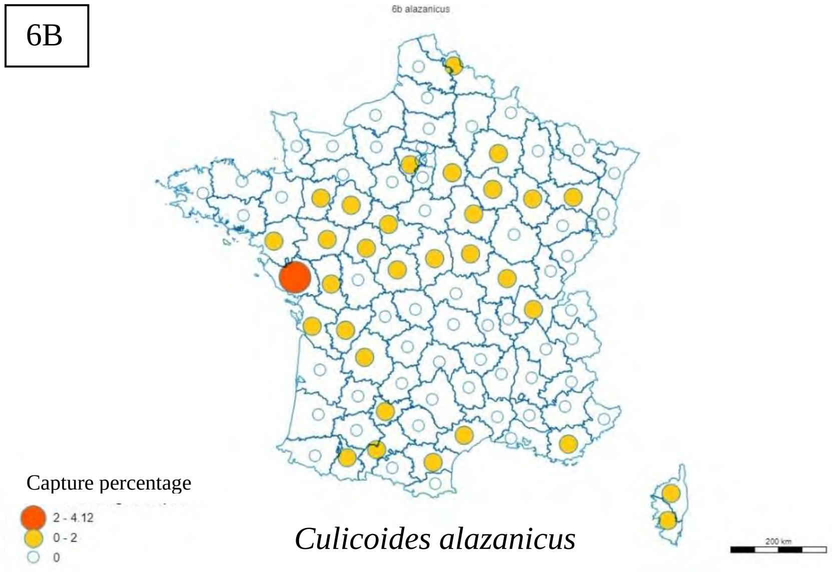
6B
6b alazanicus
Capture percentage
2 - 4.12
0 - 2
0
Culicoides alazanicus

7A

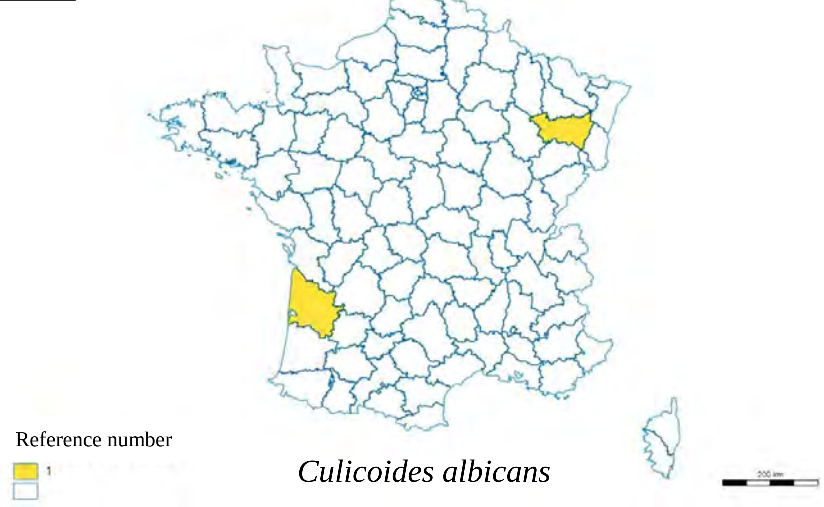
Reference number
1
Culicoides albicans

8A

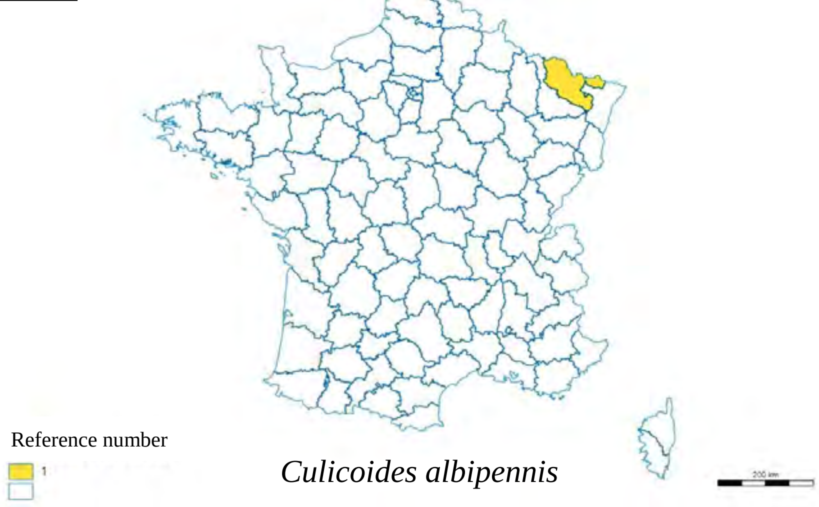
Reference number
1
Culicoides albipennis

9A

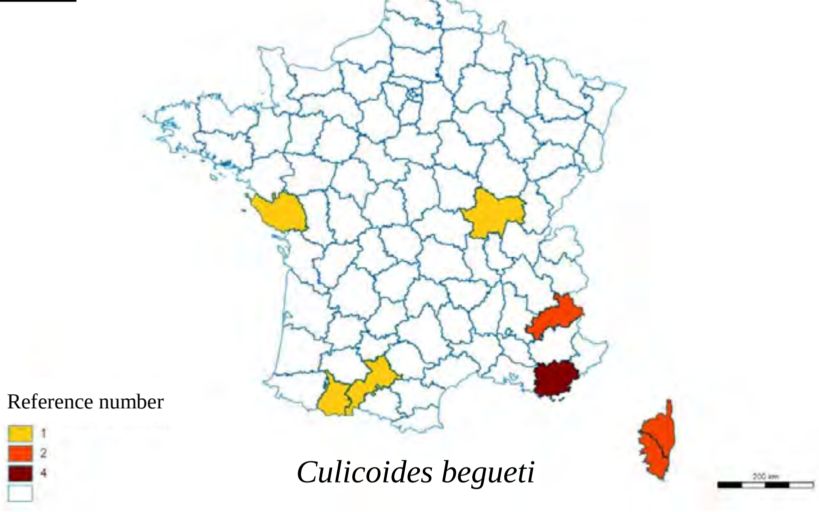
Reference number
1
2
4
Culicoides begueti

9B

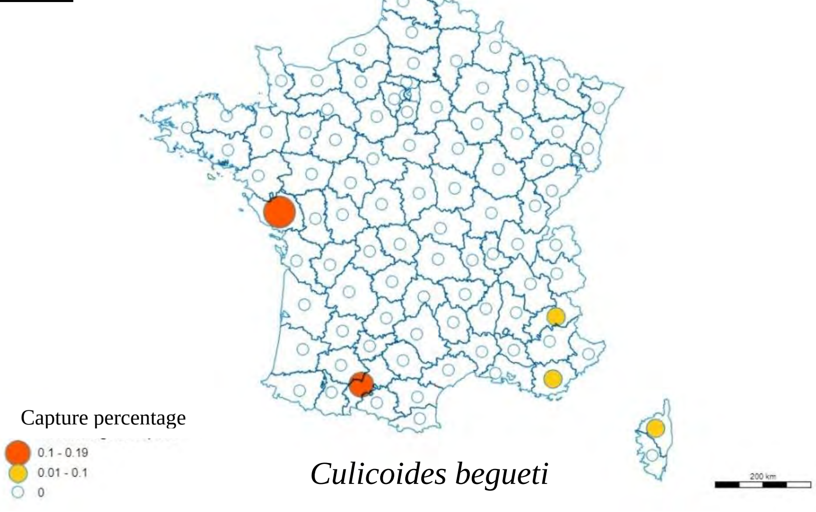
Capture percentage
0.1 - 0.19
0.01 - 0.1
0
Culicoides begueti

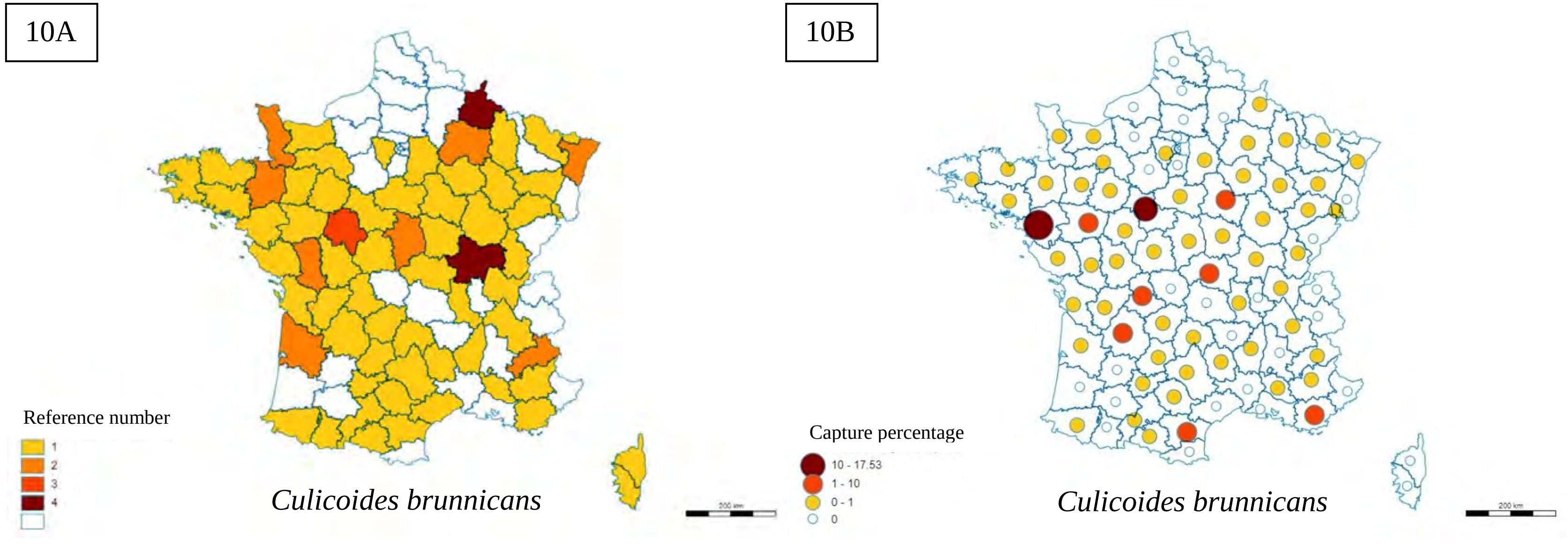
10A
Reference number
1
2
3
4
Culicoides brunnicans
10B
Capture percentage
10 - 17.53
1 - 10
0 - 1
0
Culicoides brunnicans

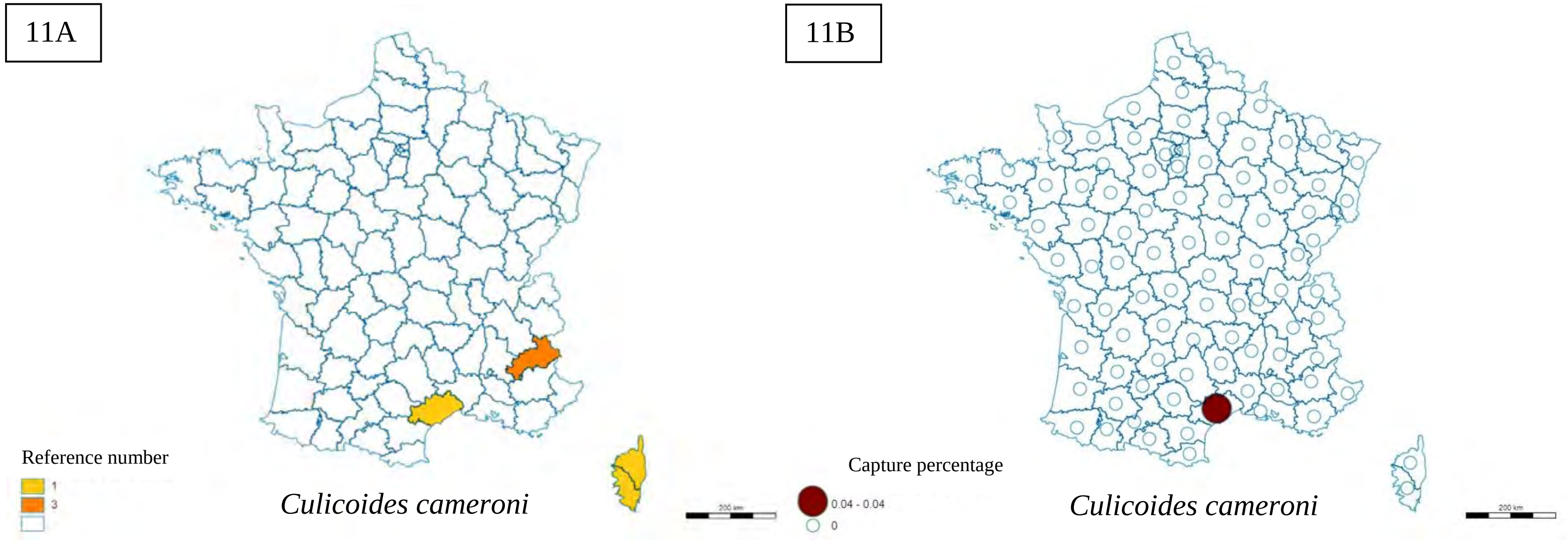
11A
Reference number
1
3
Culicoides cameroni
11B
Capture percentage
0.04 - 0.04
0
Culicoides cameroni

12A

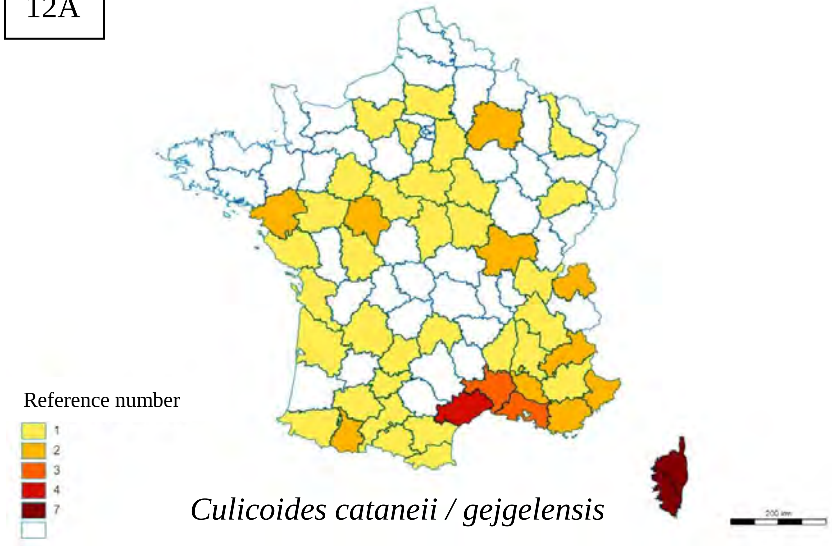

12B

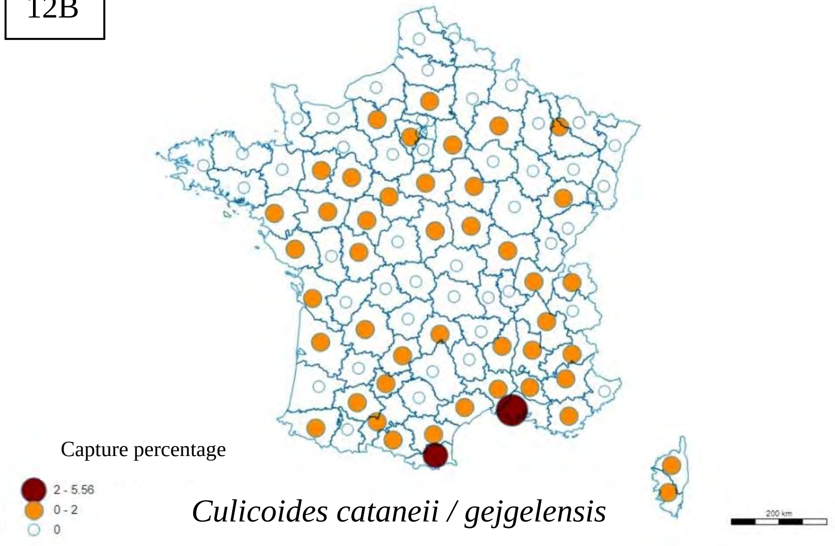

13A

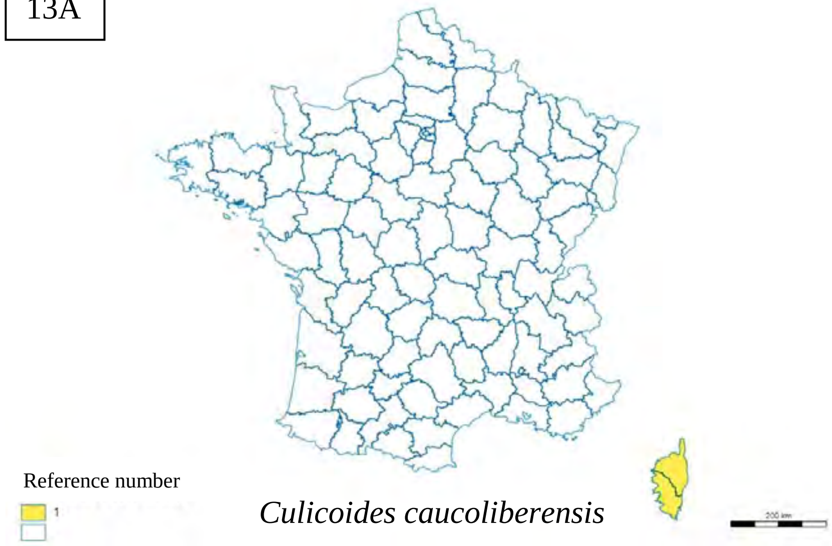

14A

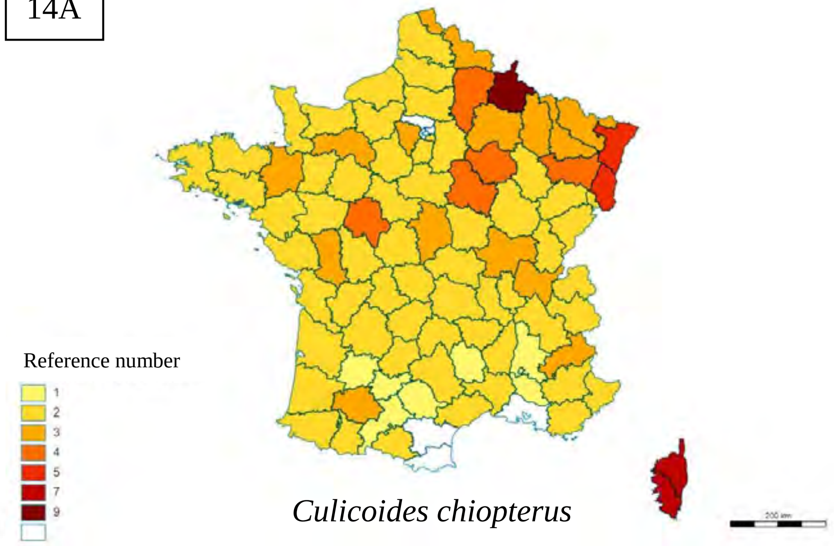

14B

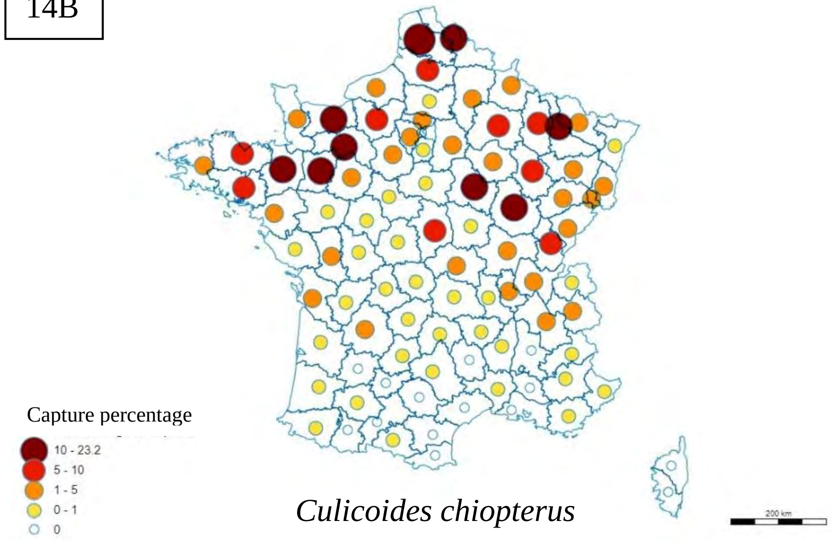

Culicoides chiopterus

15A

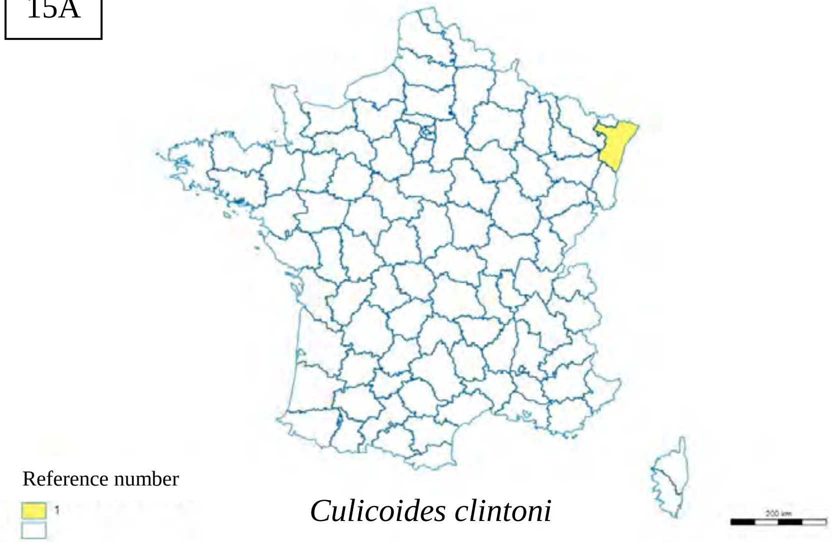

Culicoides clintoni

16A

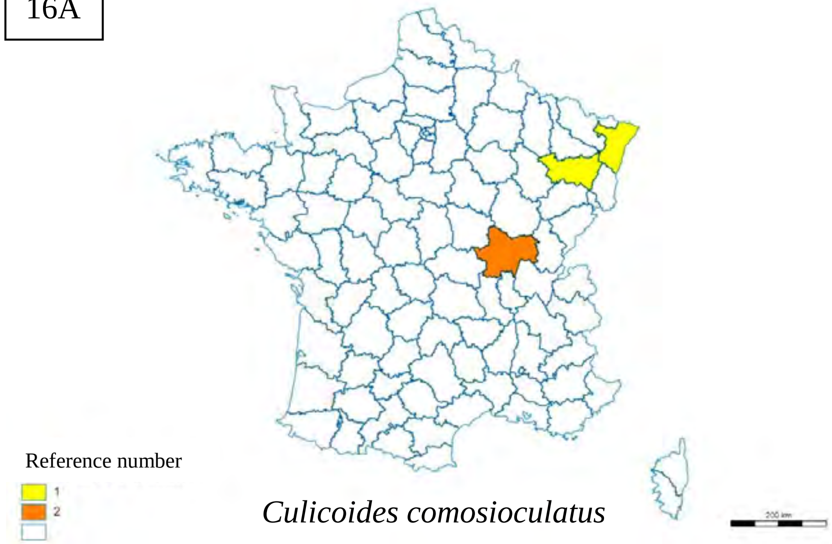

Culicoides comosioculatus

17A

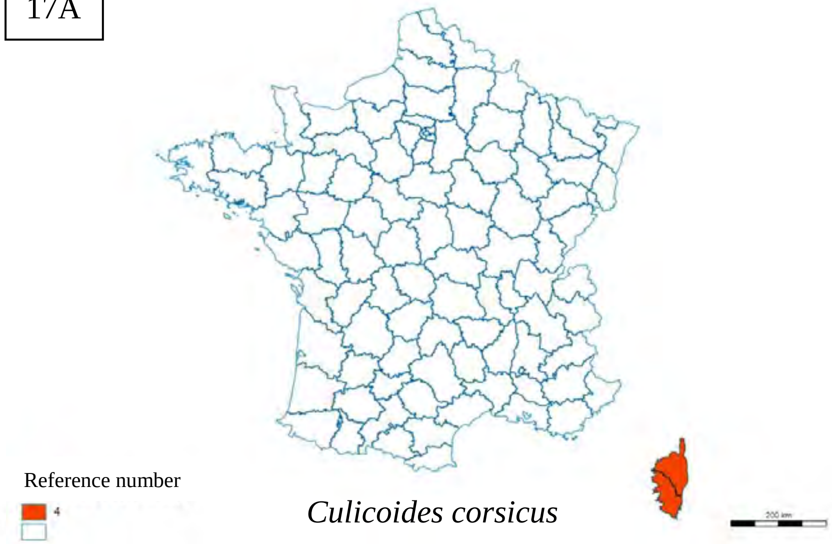

Culicoides corsicus

18A

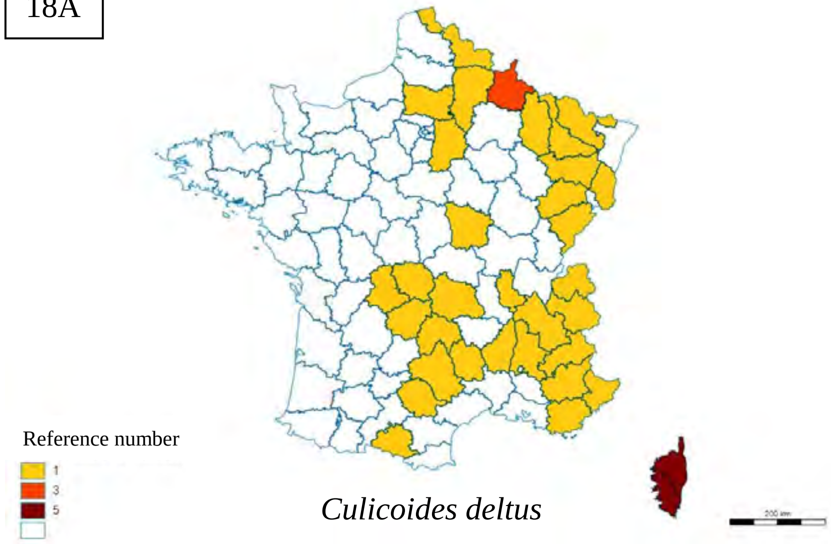

Culicoides deltus

18B

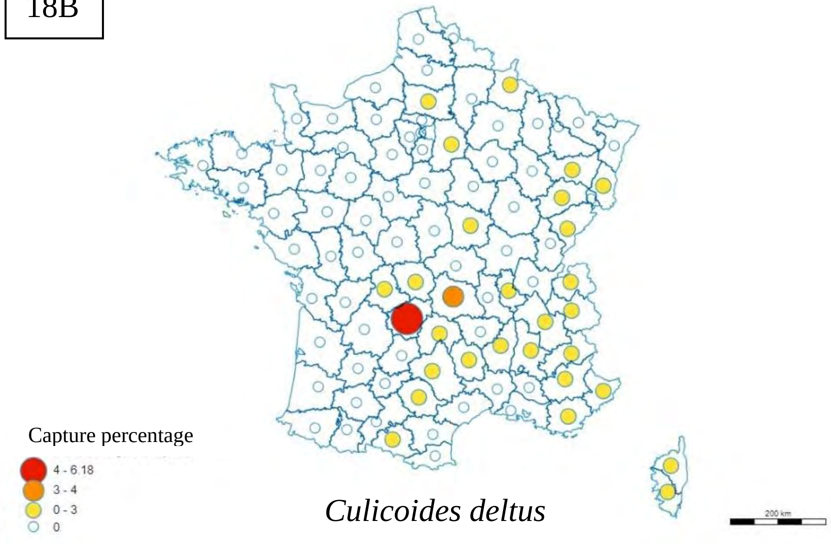

Culicoides deltus

19A

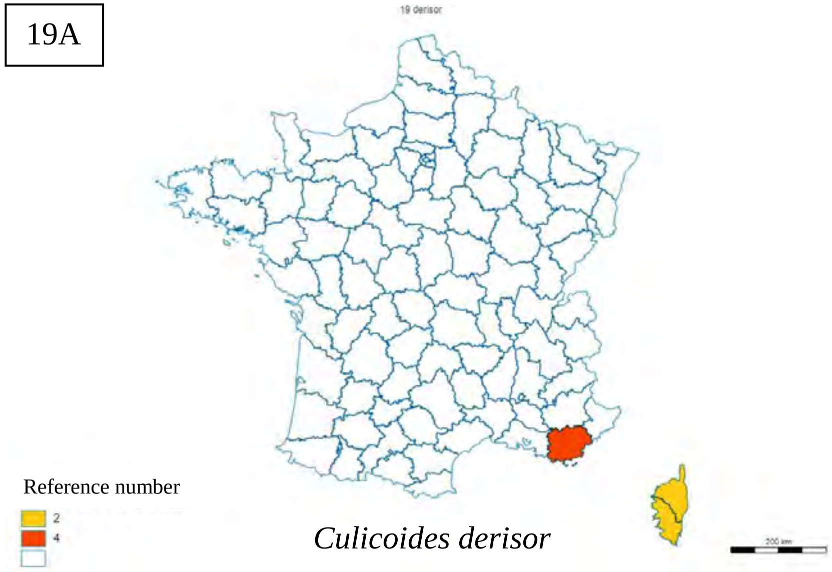

Culicoides derisor

20A

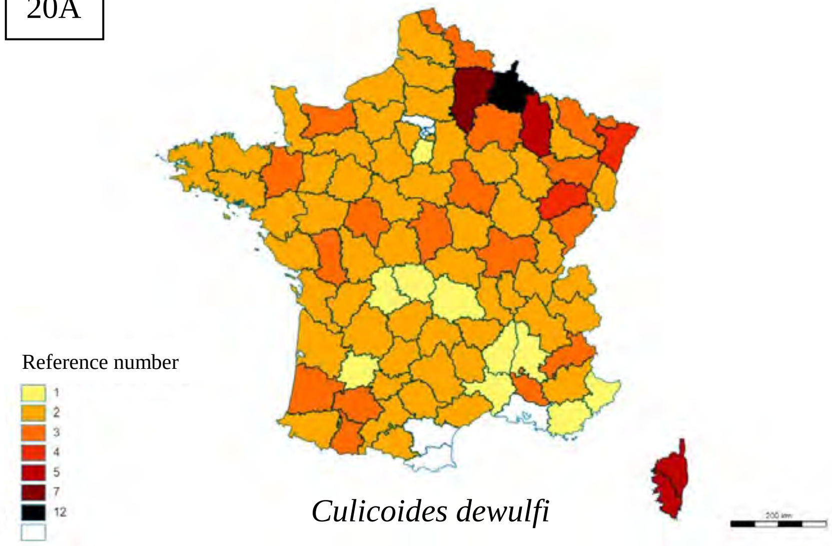

Culicoides dewulfi

20B

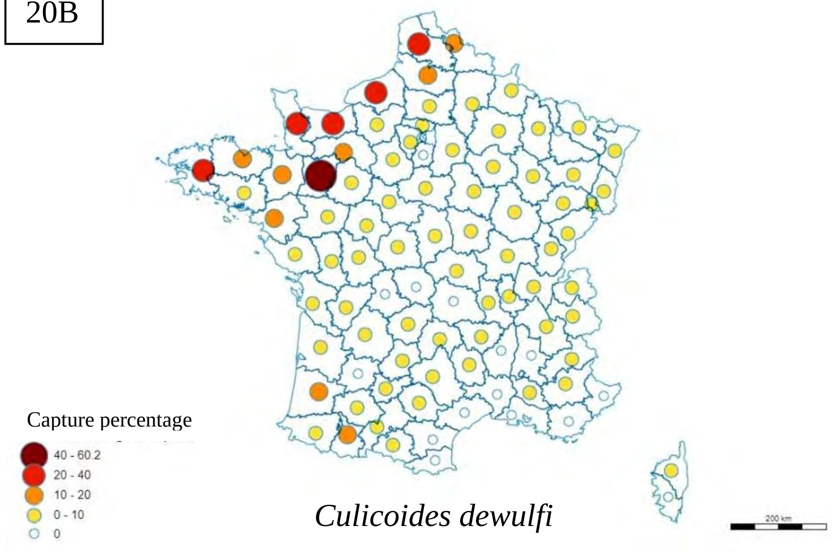

21A

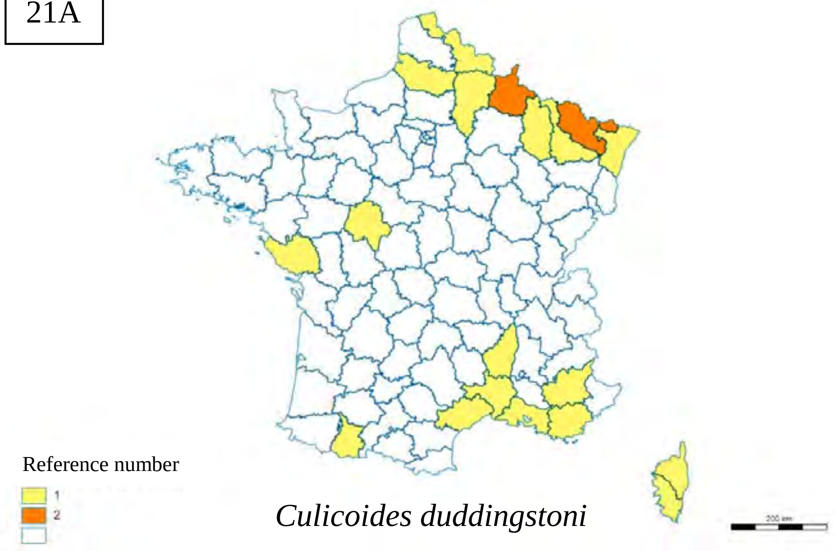

21B

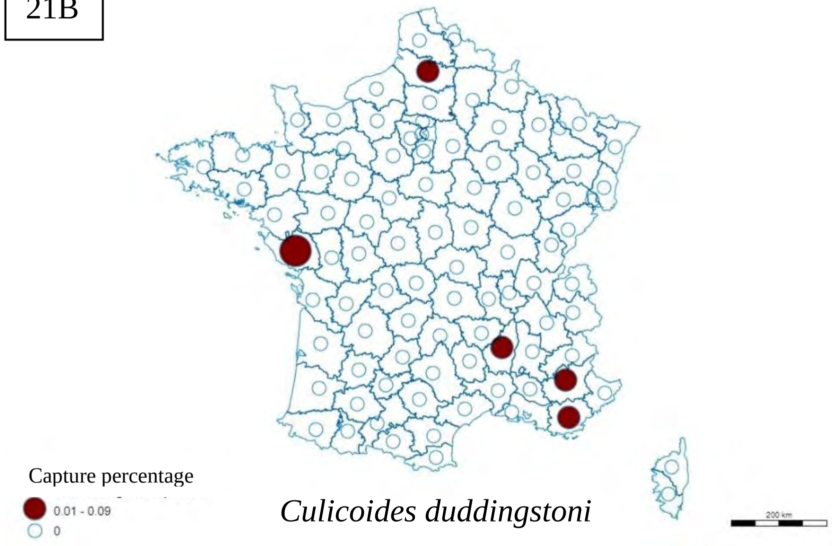

22A

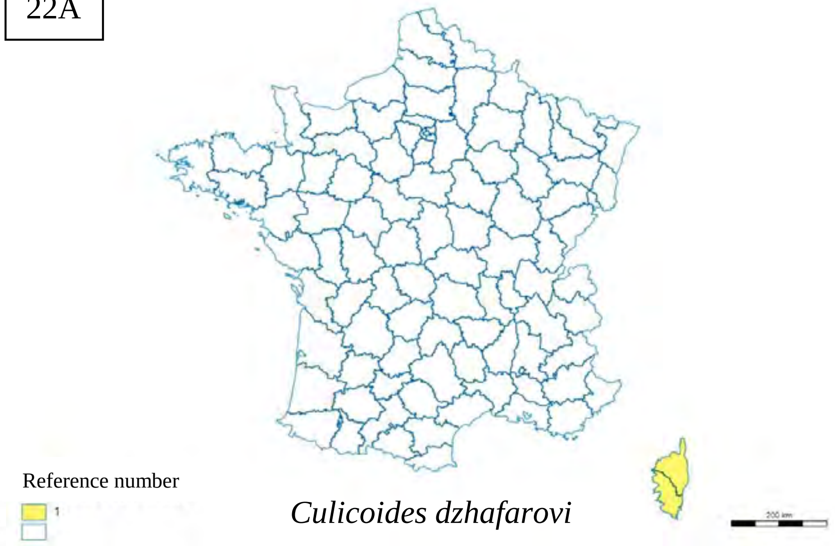

23A

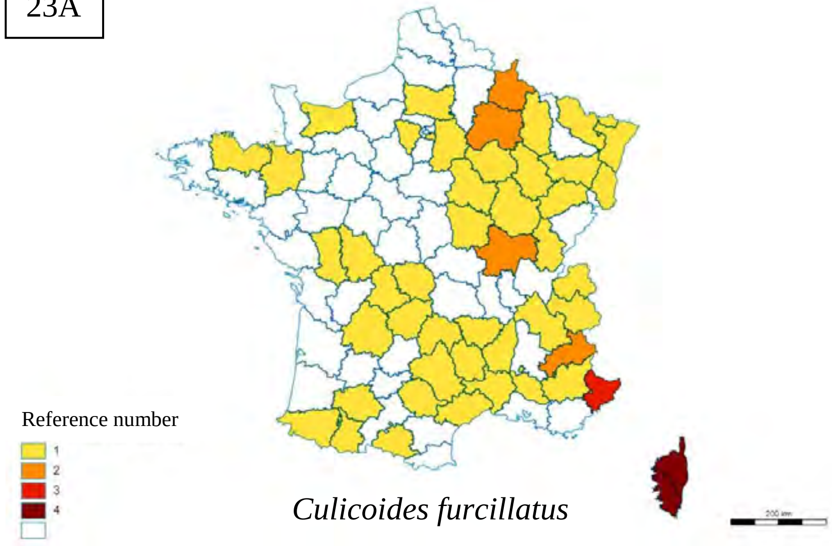

23B

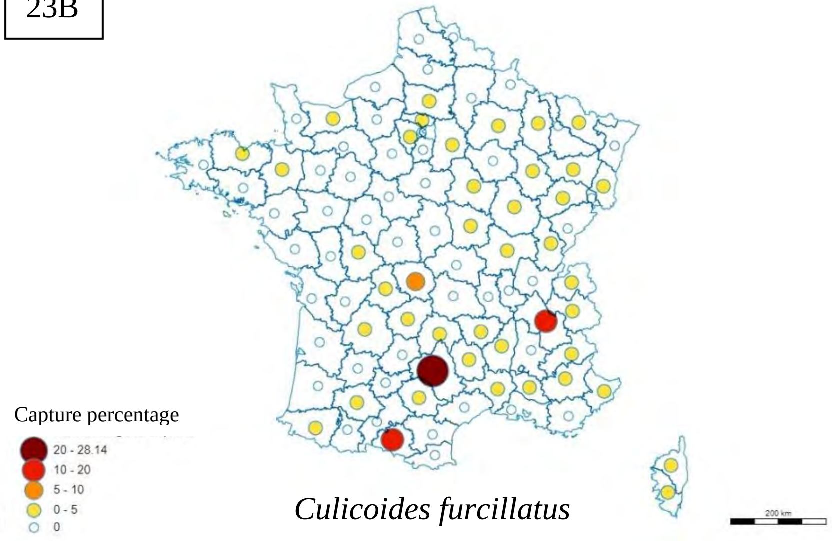

24A

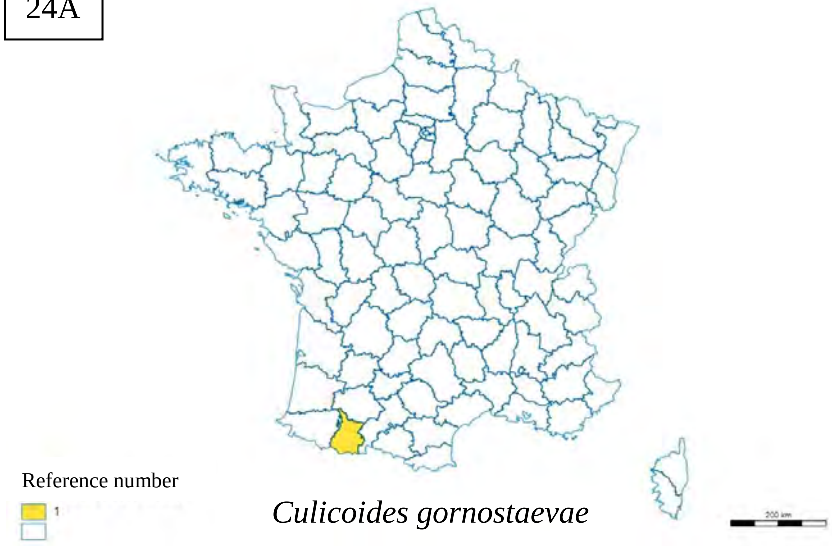

25A

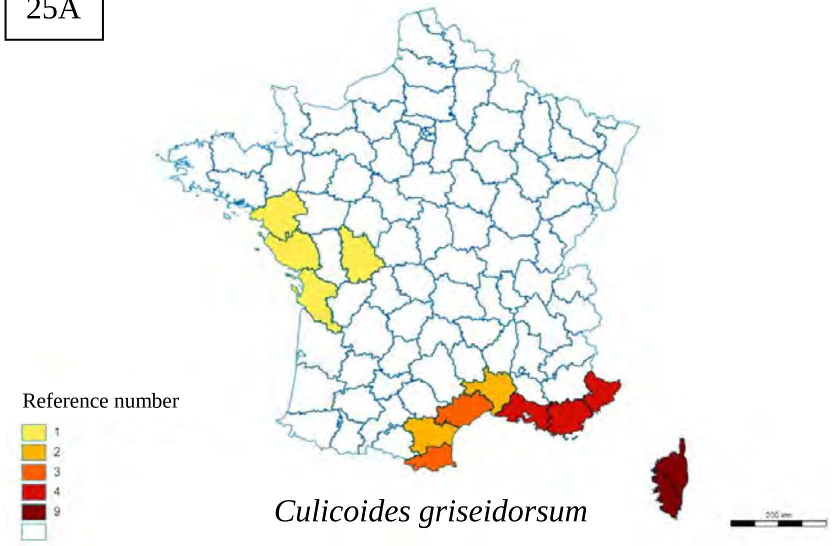

25B

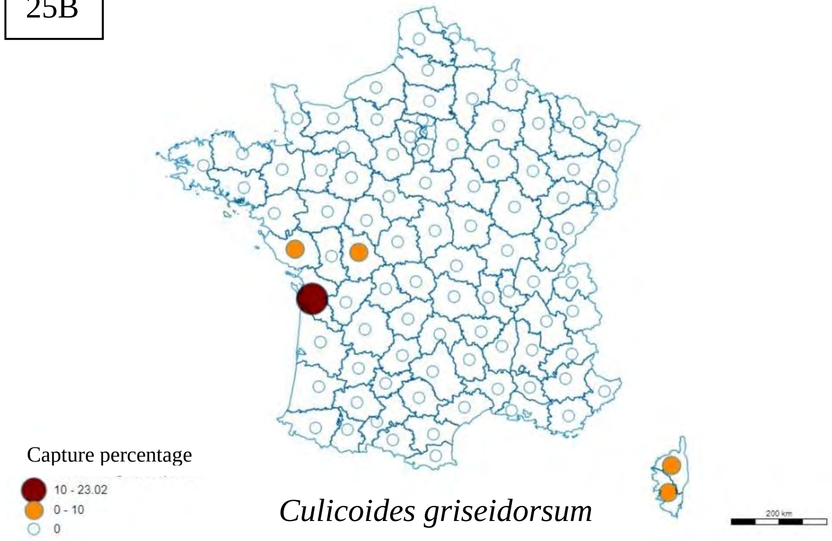

Culicoides griseidorsum

26A

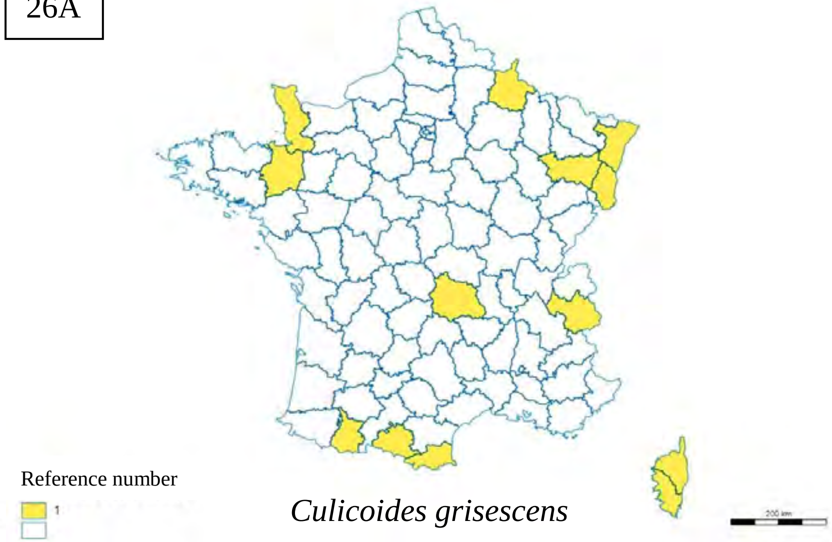

Culicoides grisescens

26B

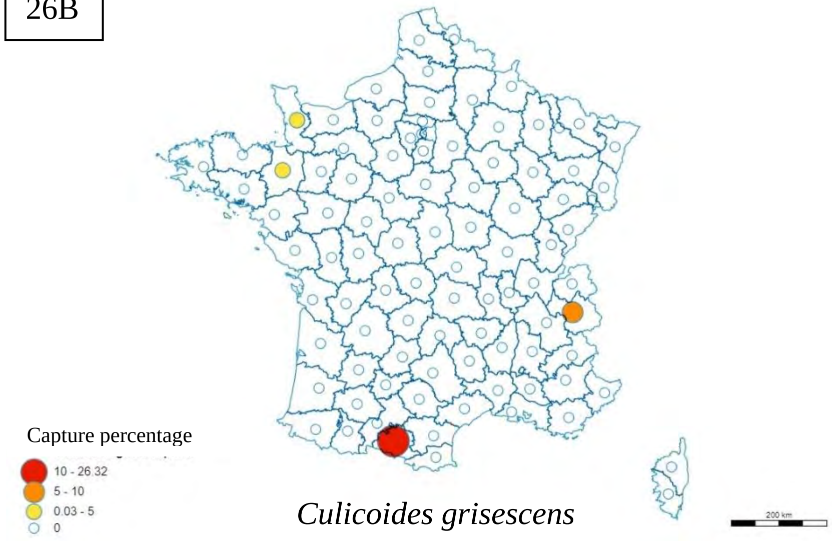

Culicoides grisescens

27A

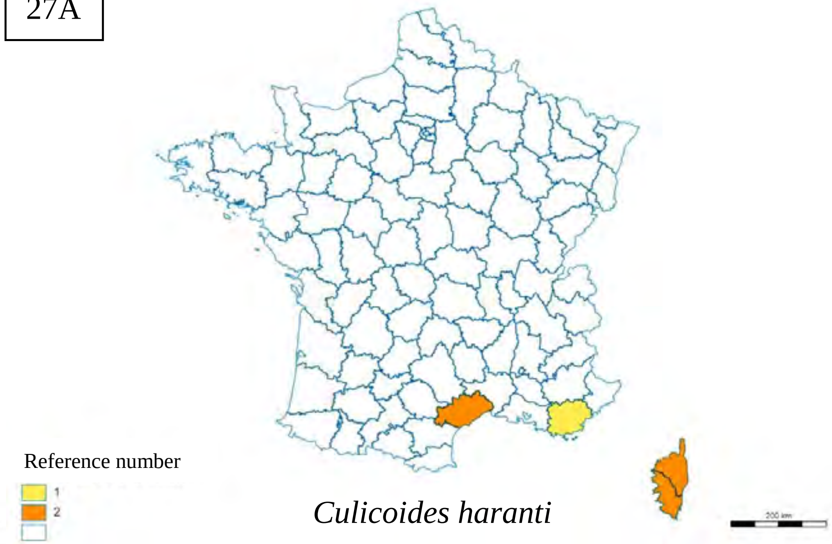

Culicoides haranti

27B

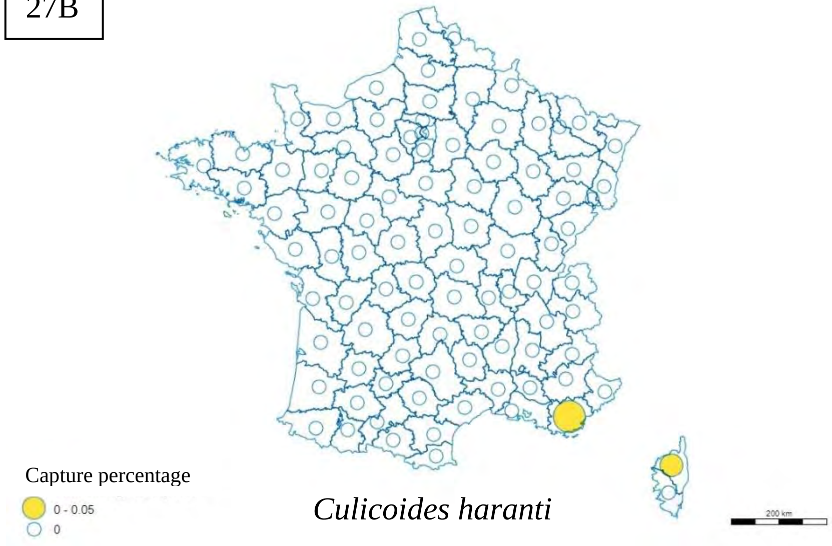

Culicoides haranti

28A

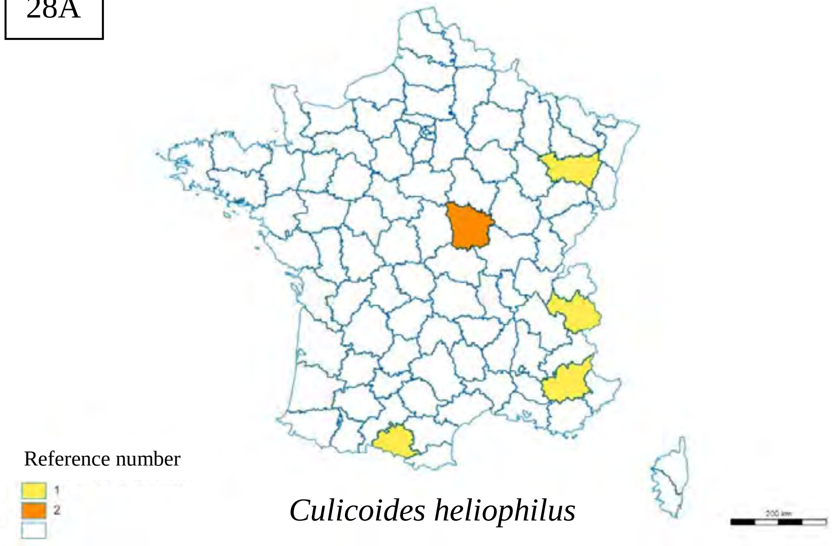

Culicoides heliophilus

28B

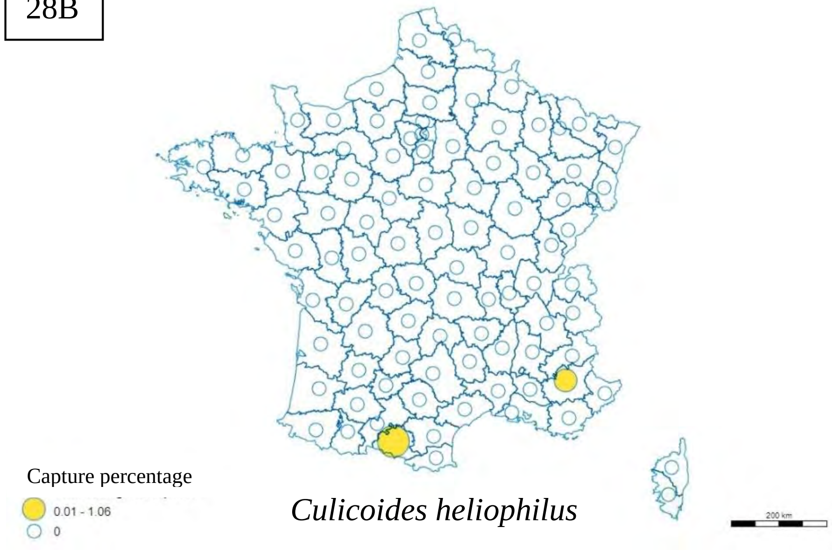

Culicoides heliophilus

29A

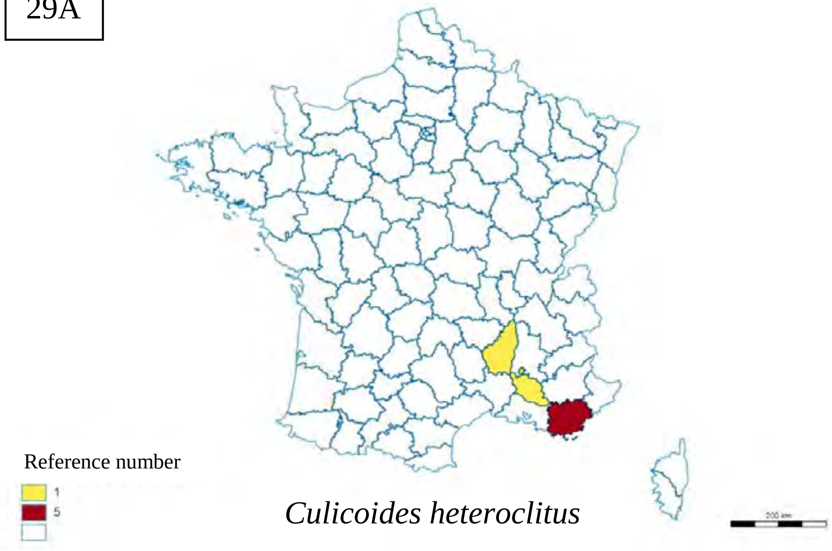

Culicoides heteroclitus

29B

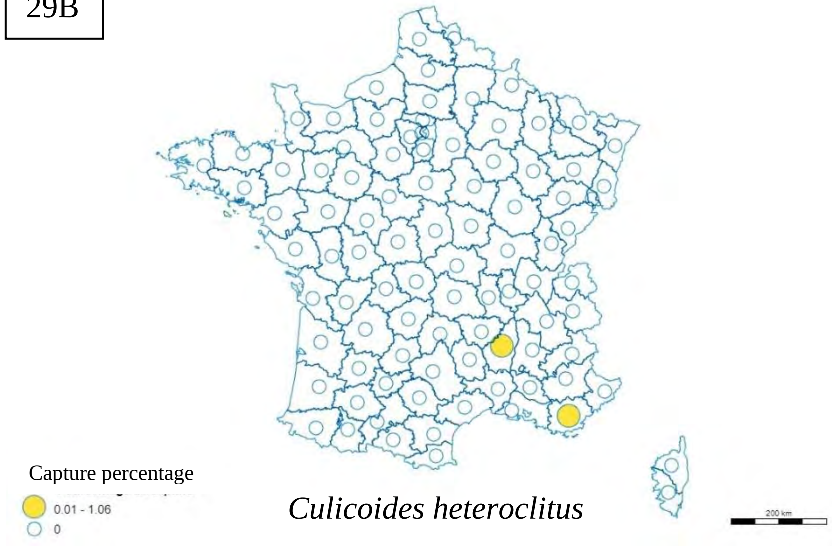

Culicoides heteroclitus

30A

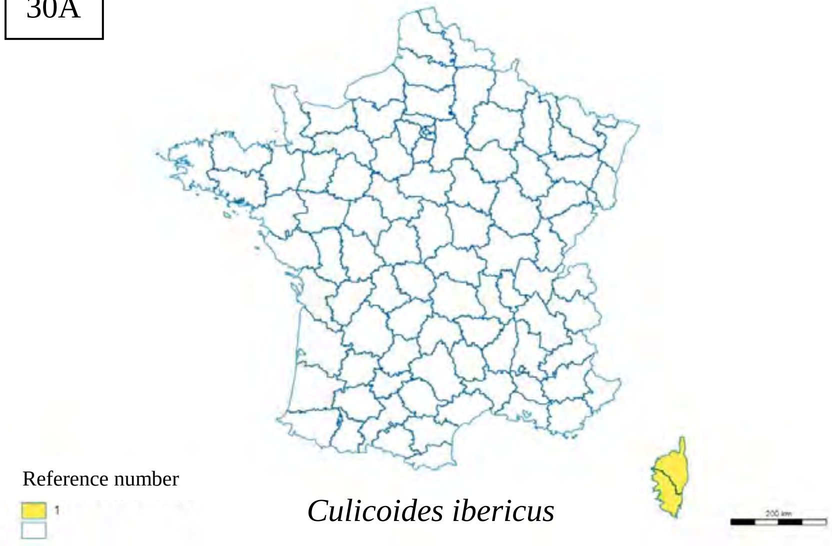

Culicoides ibericus

31A

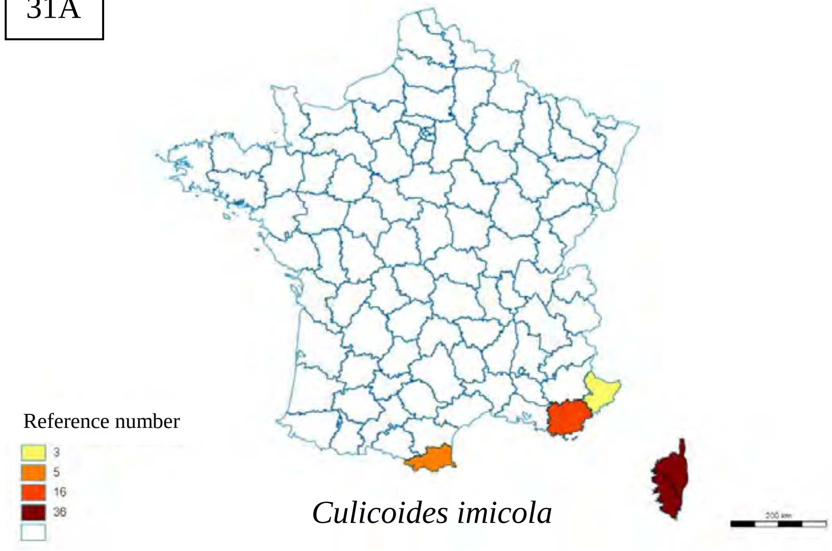

Culicoides imicola

31B

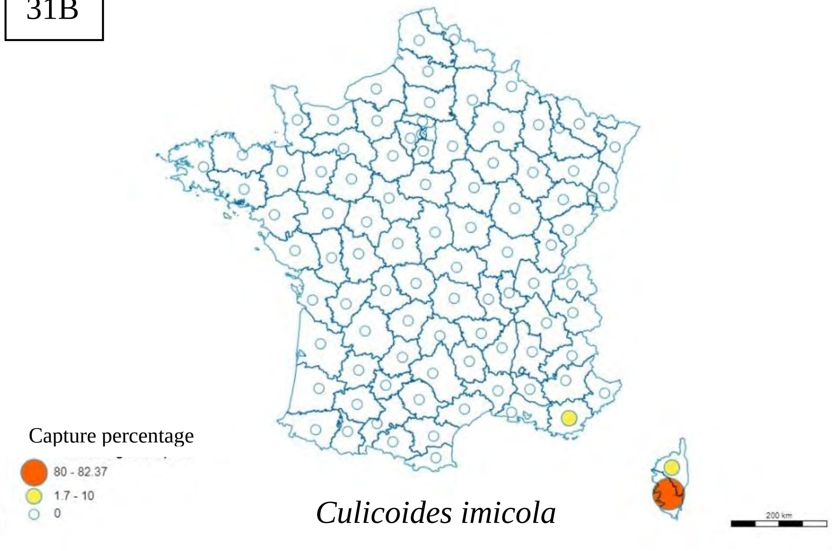

Culicoides imicola

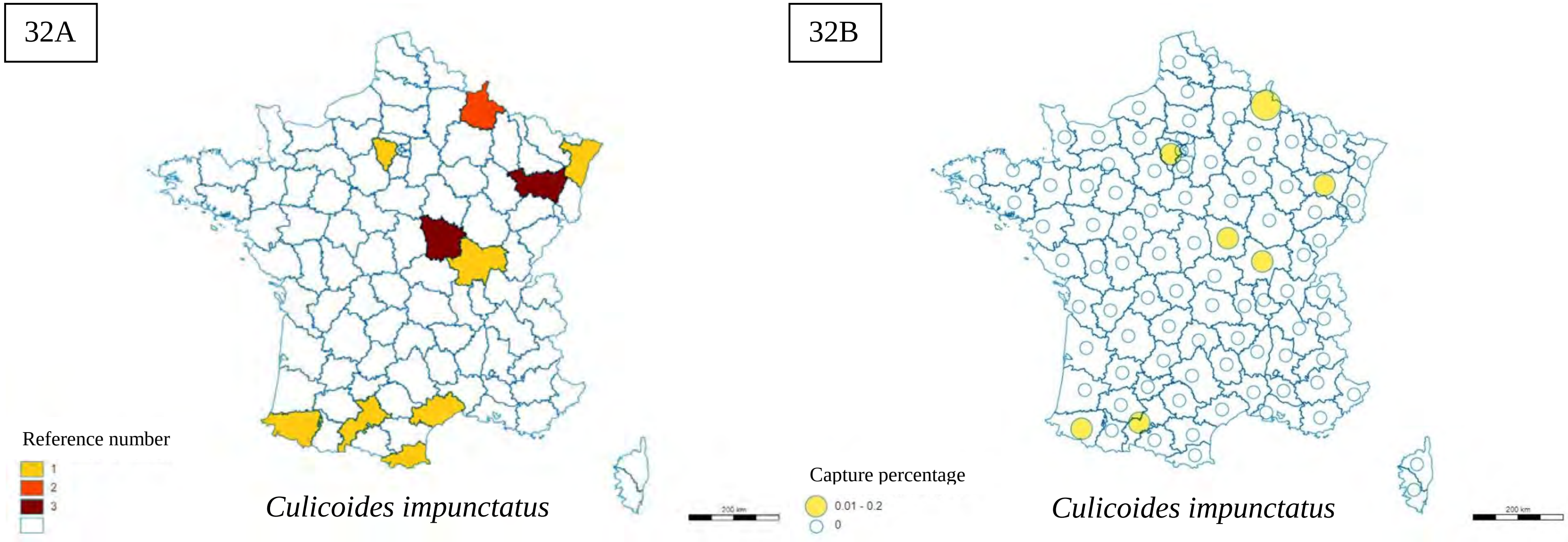
32A
Reference number
1
2
3
Culicoides impunctatus
200 km
32B
Capture percentage
0.01 - 0.2
0
Culicoides impunctatus
200 km

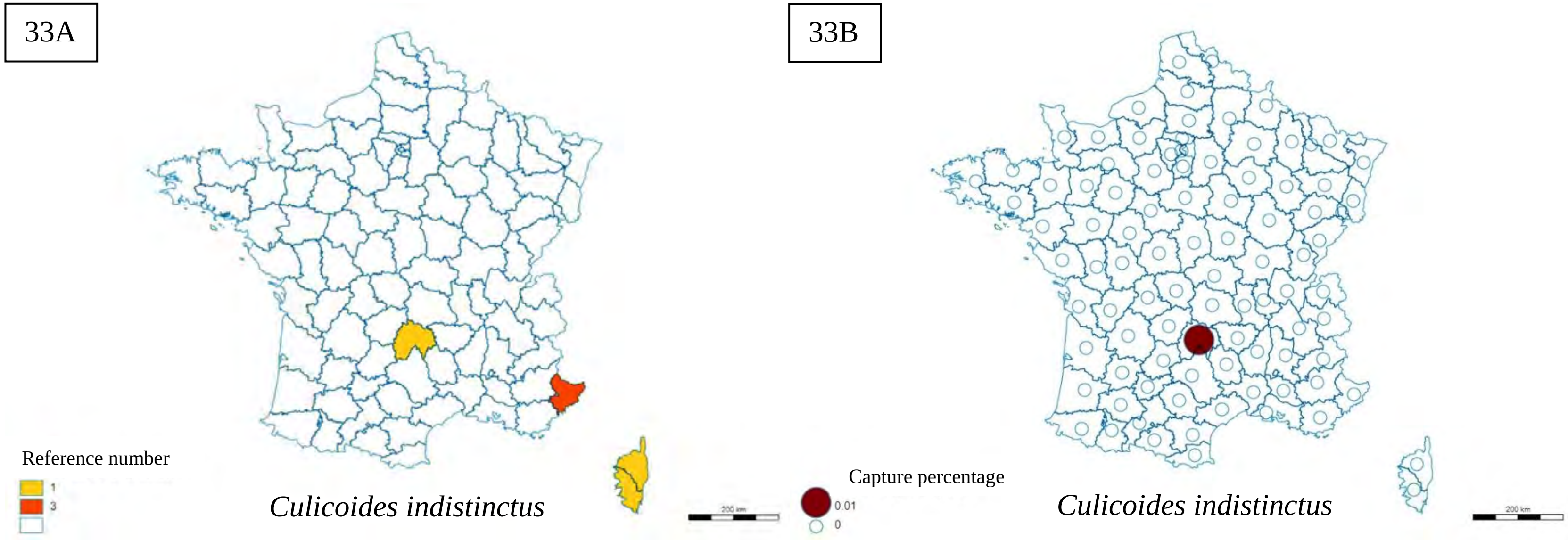
33A
Reference number
1
3
Culicoides indistinctus
200 km
33B
Capture percentage
0.01
0
Culicoides indistinctus
200 km

34A

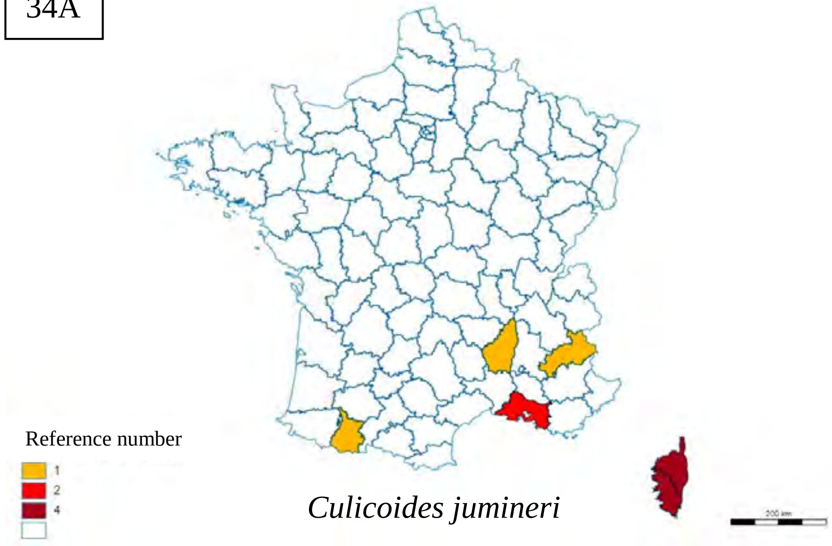

34B

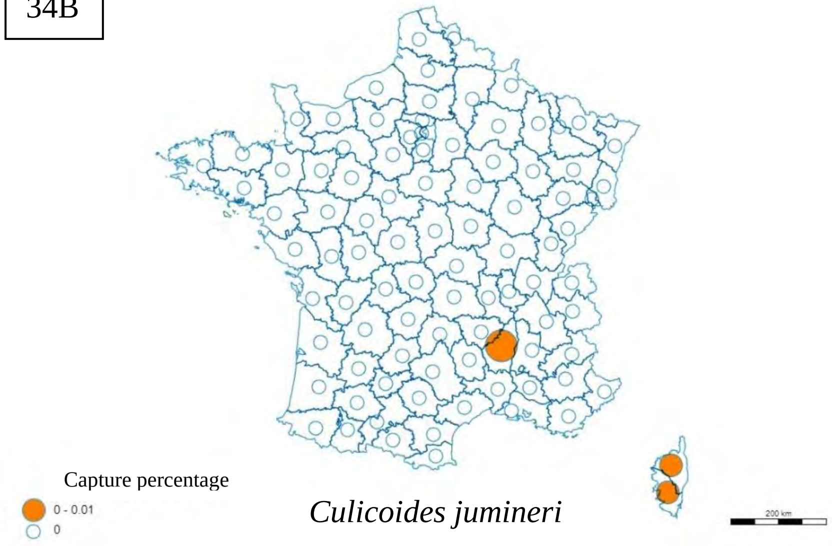

35A

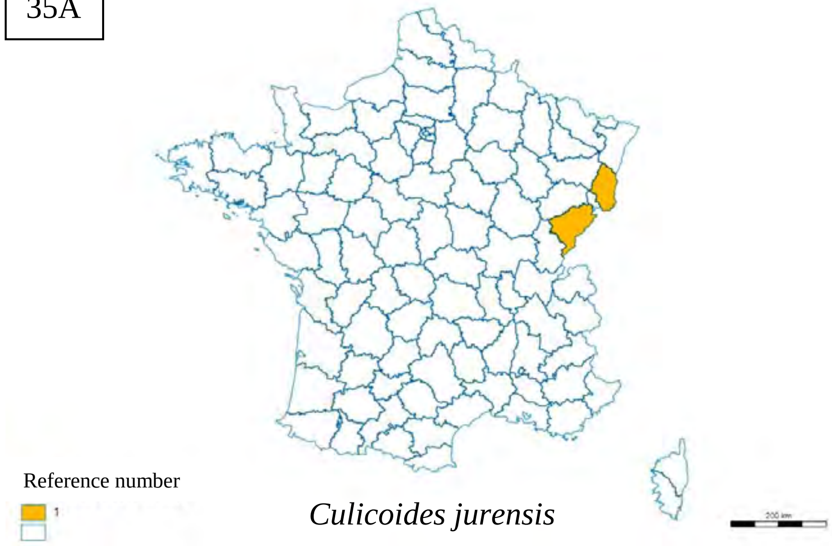

36A

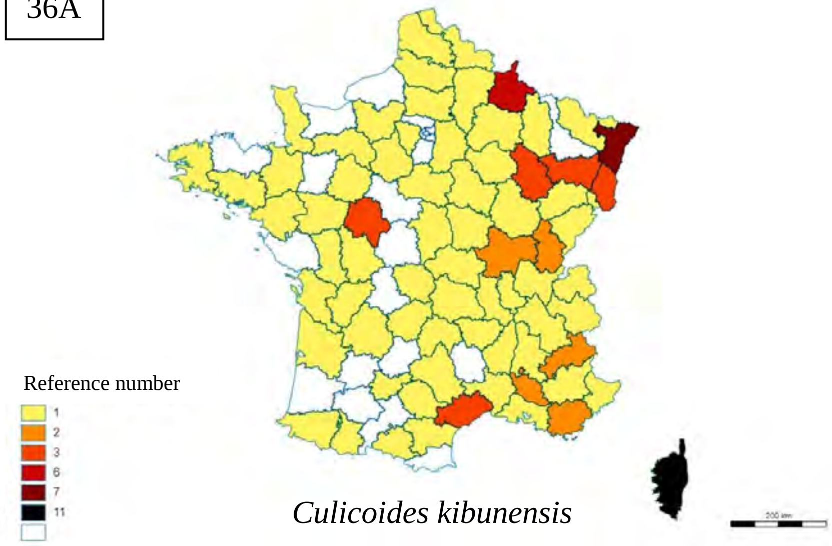

36B

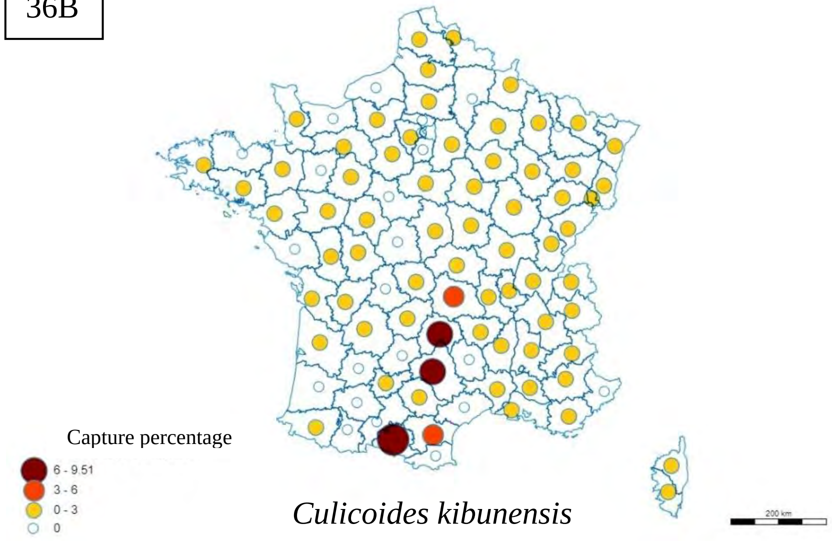

Culicoides kibunensis

37A

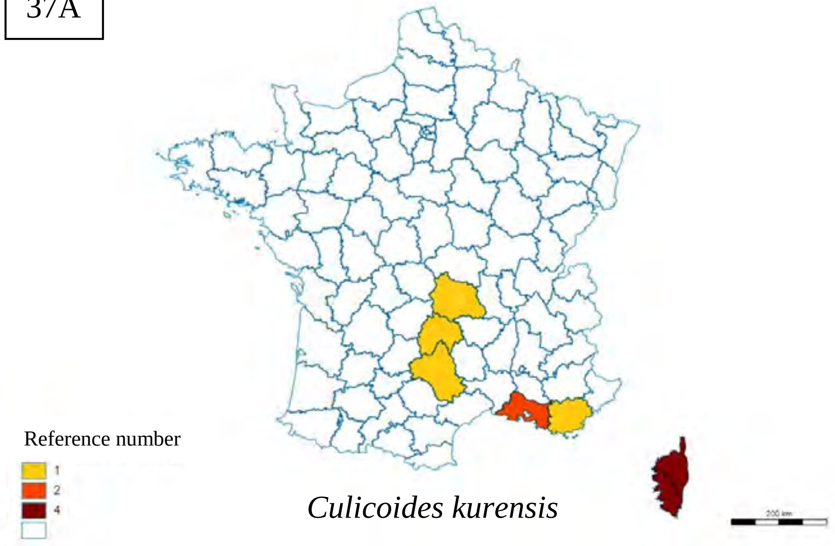

Culicoides kurensis

37B

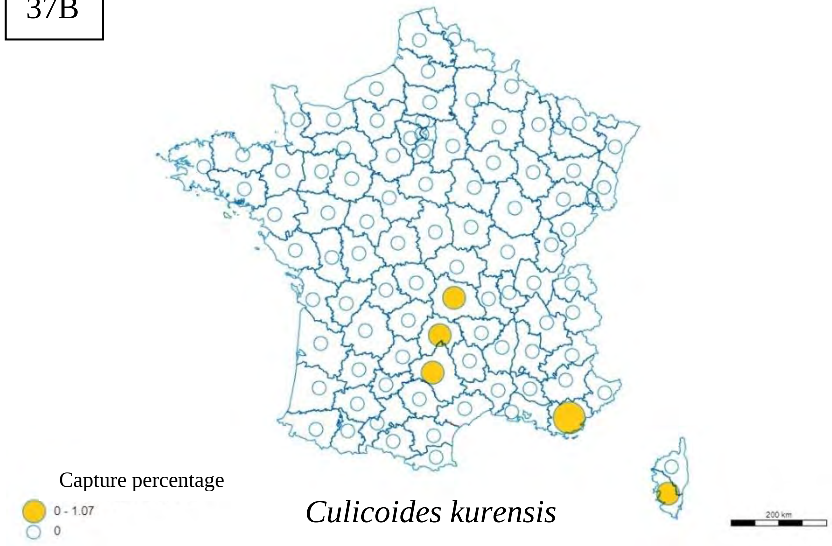

Culicoides kurensis

38A

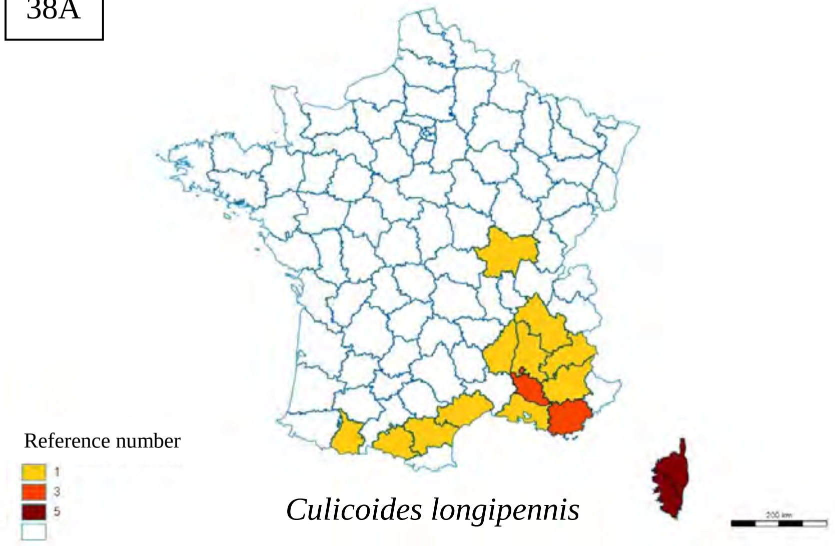

Culicoides longipennis

38B

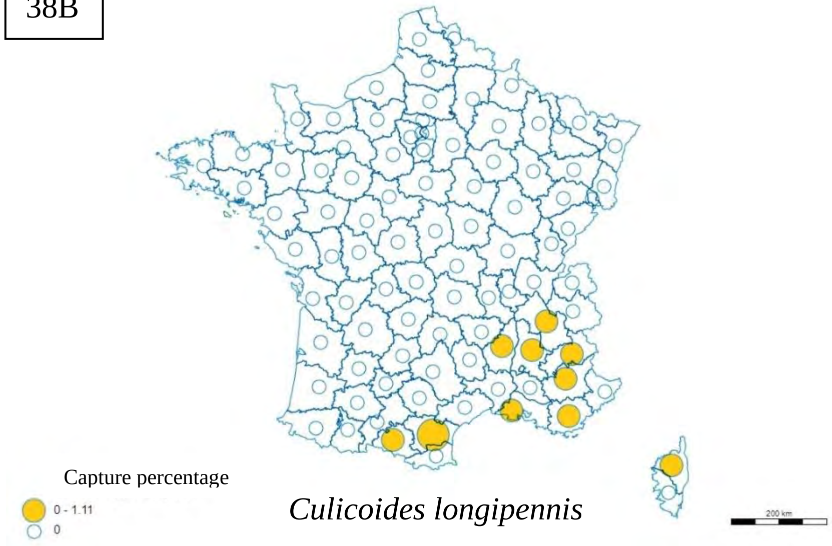

Culicoides longipennis

39A

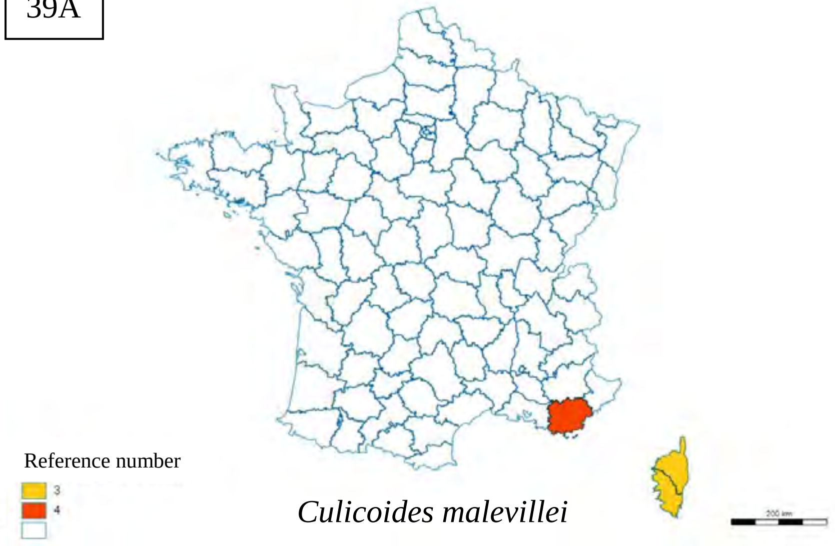

Culicoides malevillei

40A

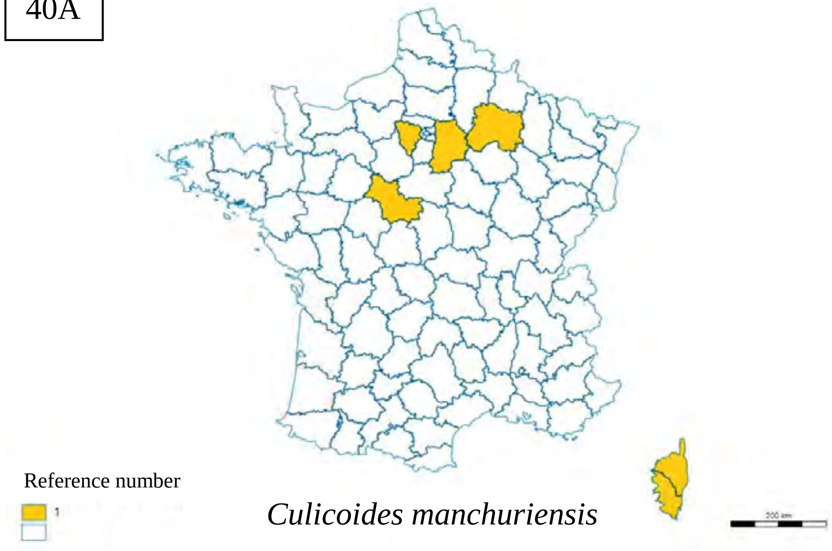

Culicoides manchuriensis

40B

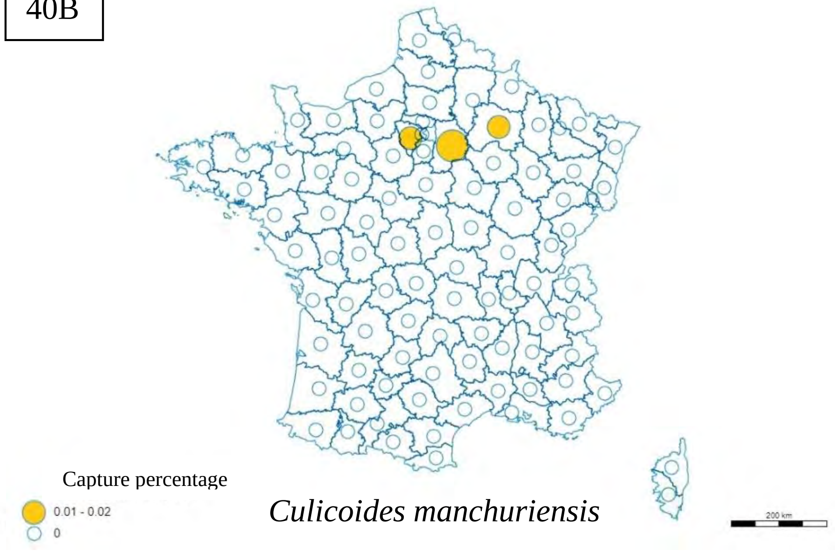

Culicoides manchuriensis

41A

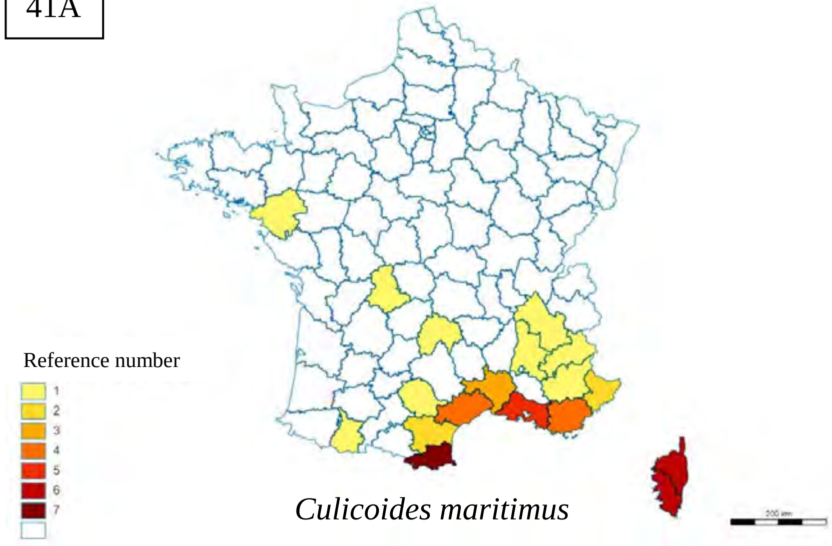

41B

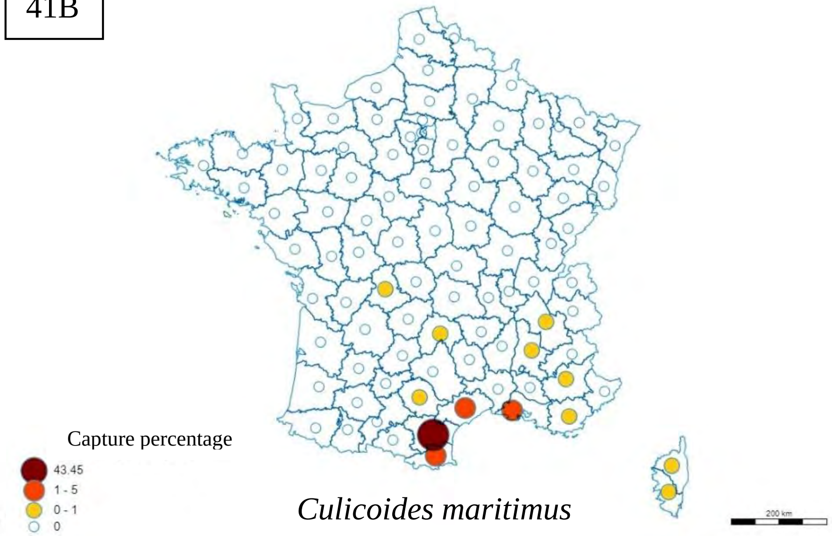

42A

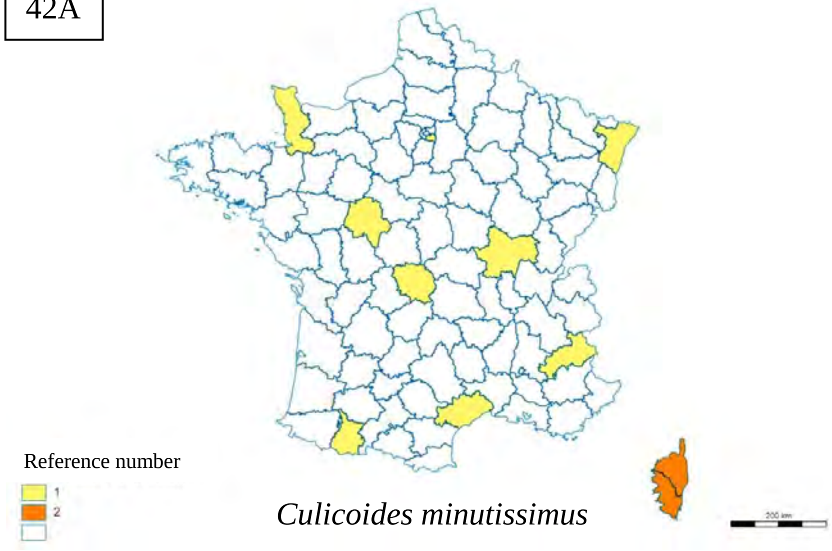

42B

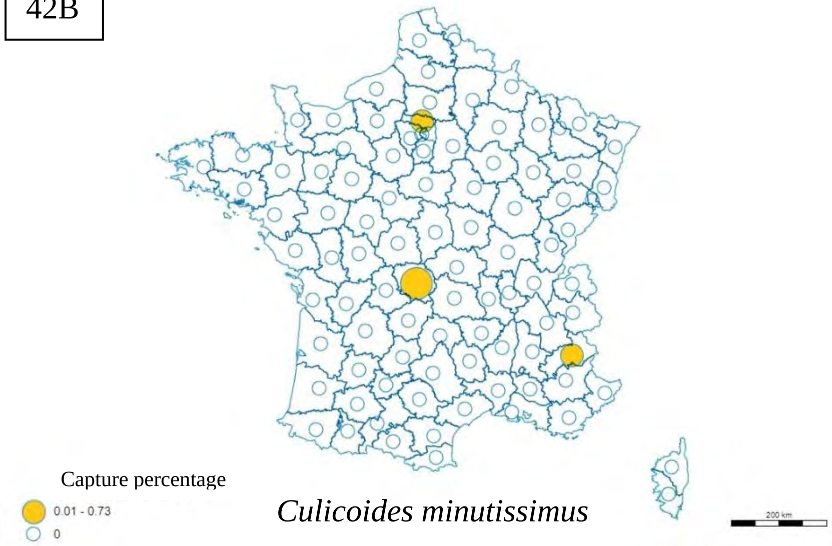

43A

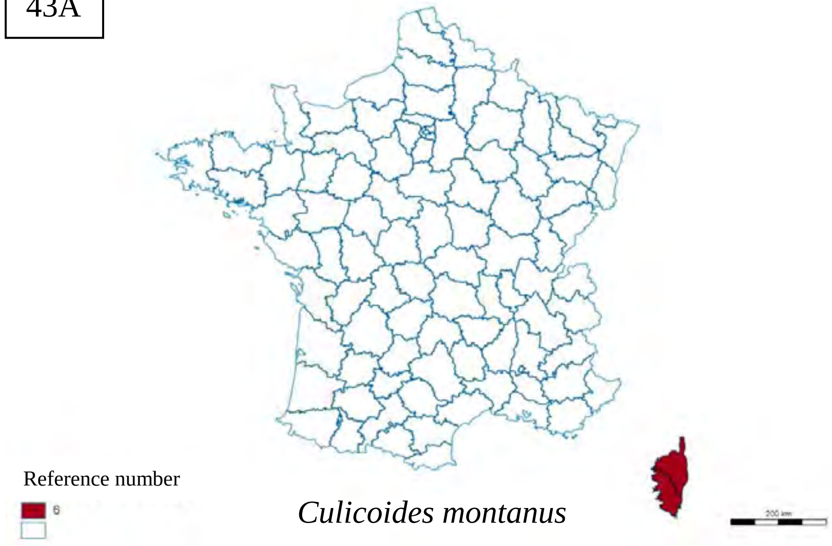

Culicoides montanus

44A

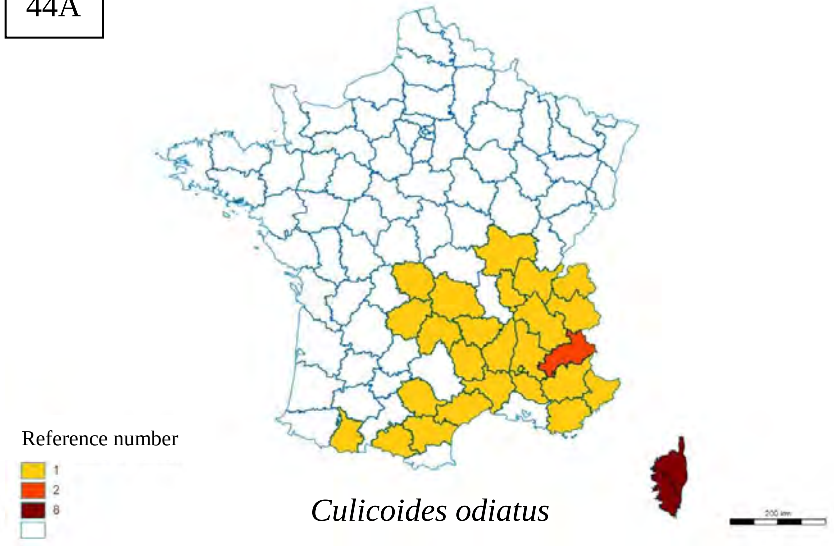

Culicoides odiatus

44B

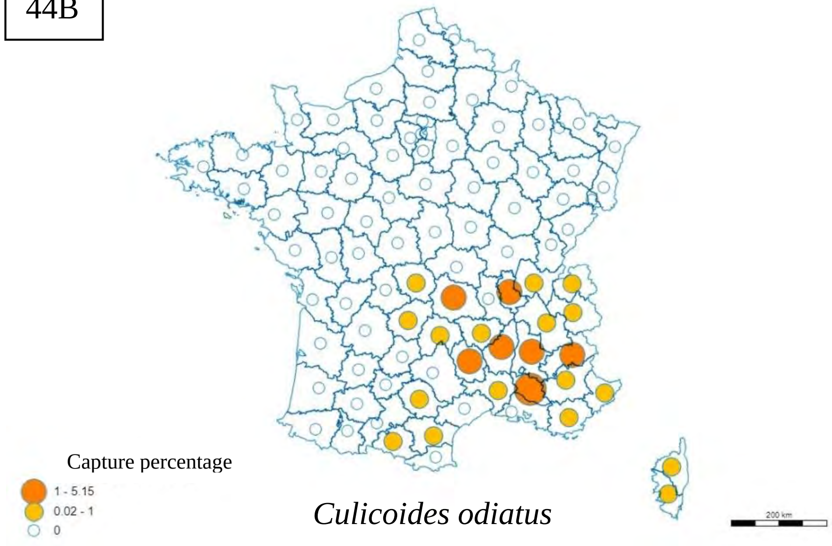

Culicoides odiatus

45A

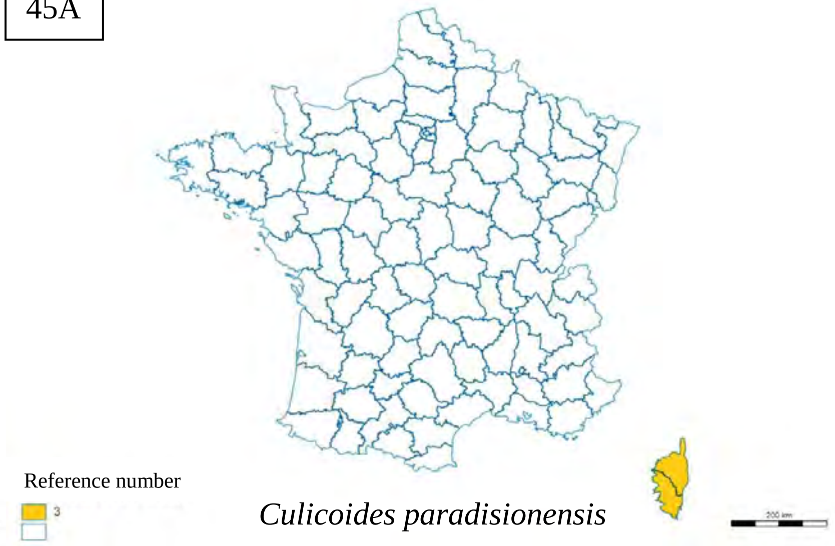

Culicoides paradisionensis

45B

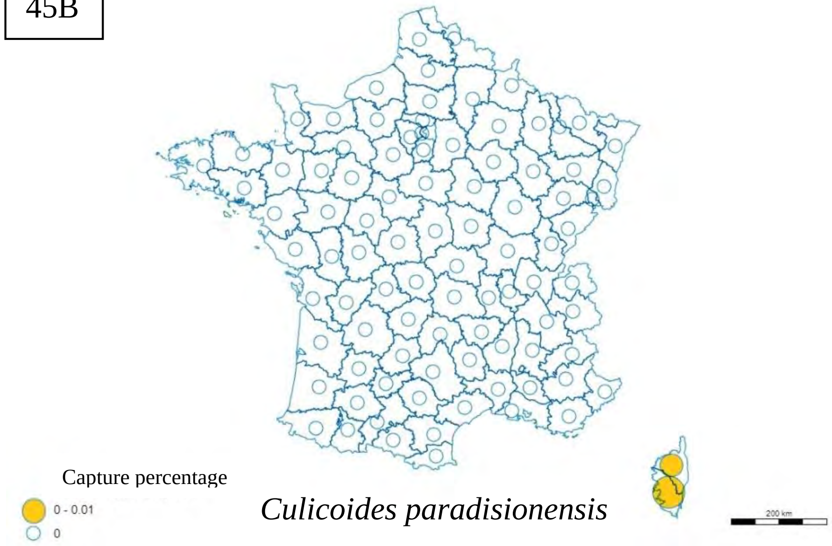

Culicoides paradisionensis

46A

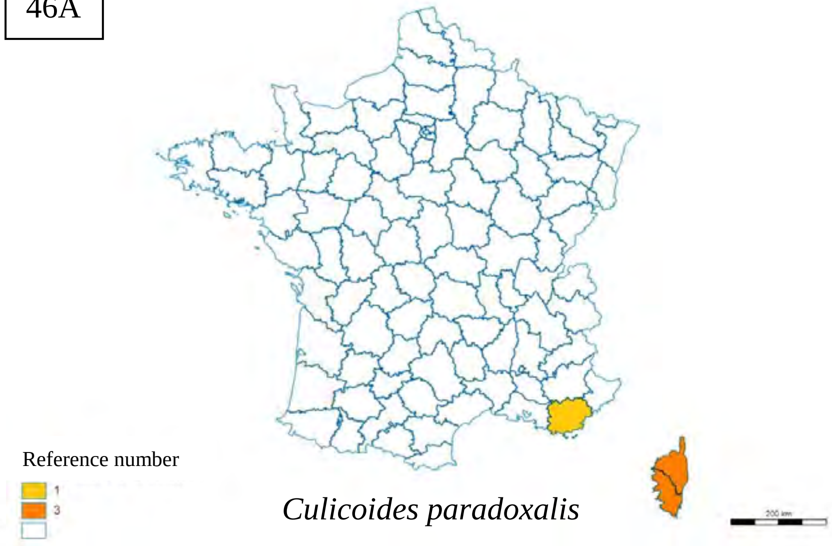

Culicoides paradoxalis

47A

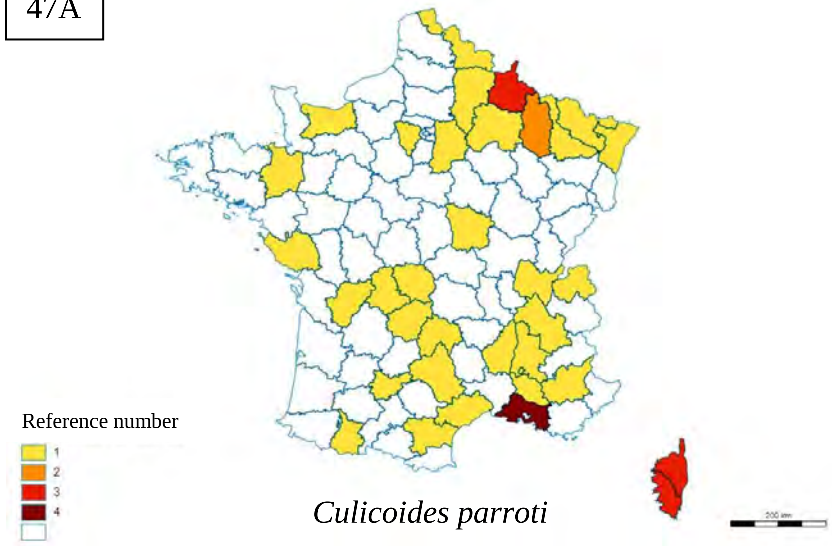

Culicoides parroti

47B

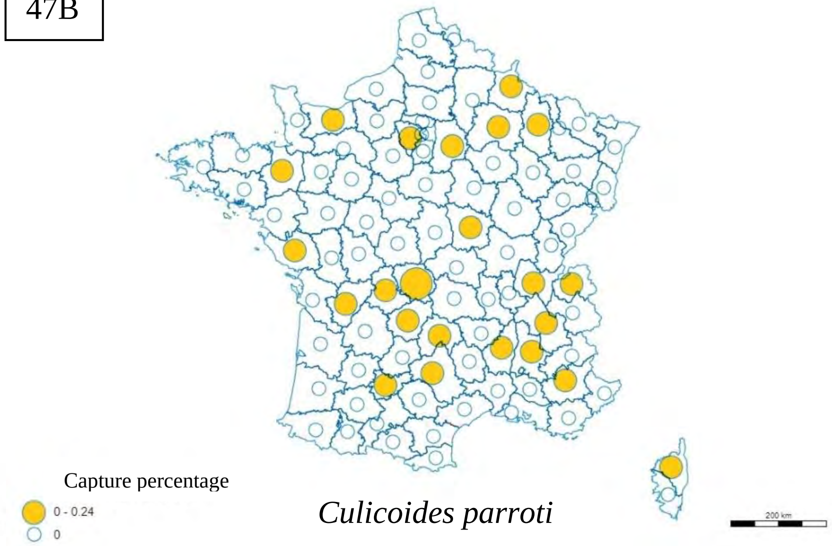

Culicoides parroti

48A

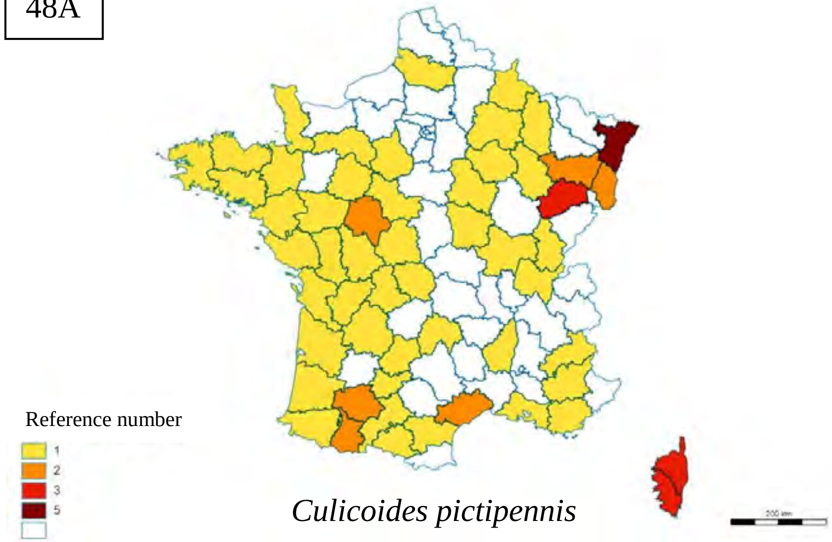

48B

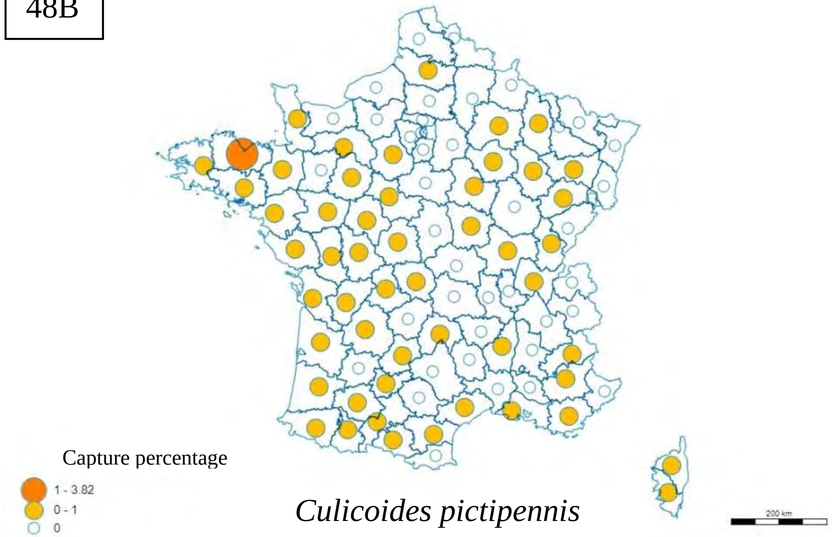

49A

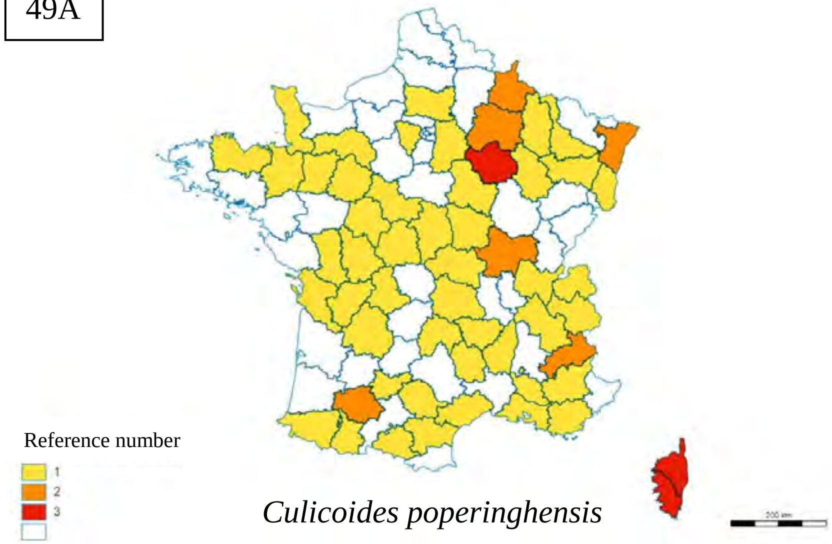

49B

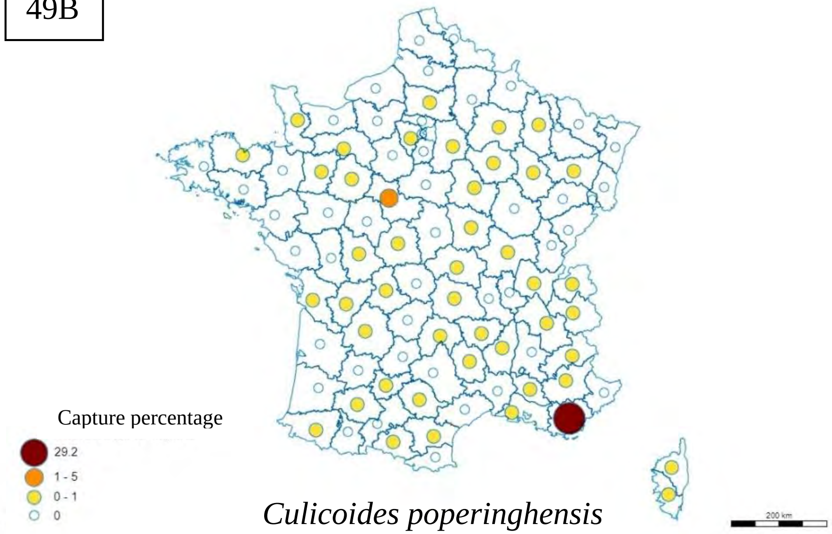

50A

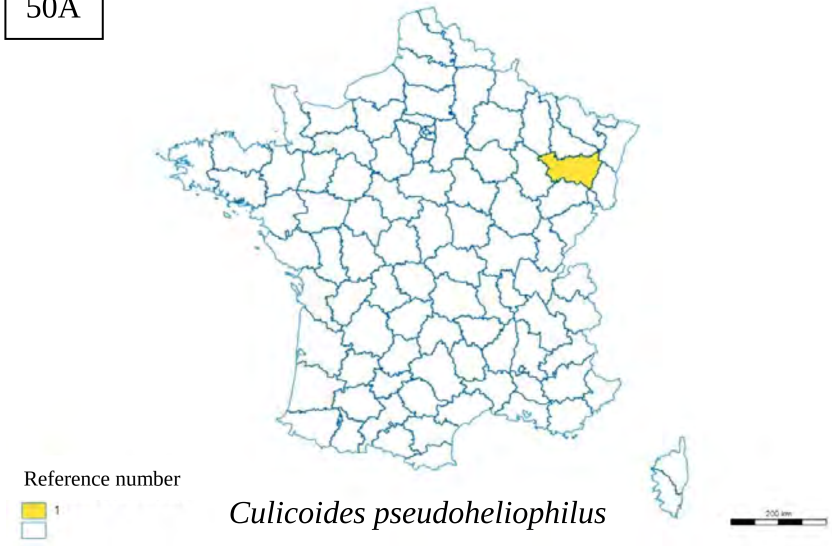

Culicoides pseudoheliophilus

51A

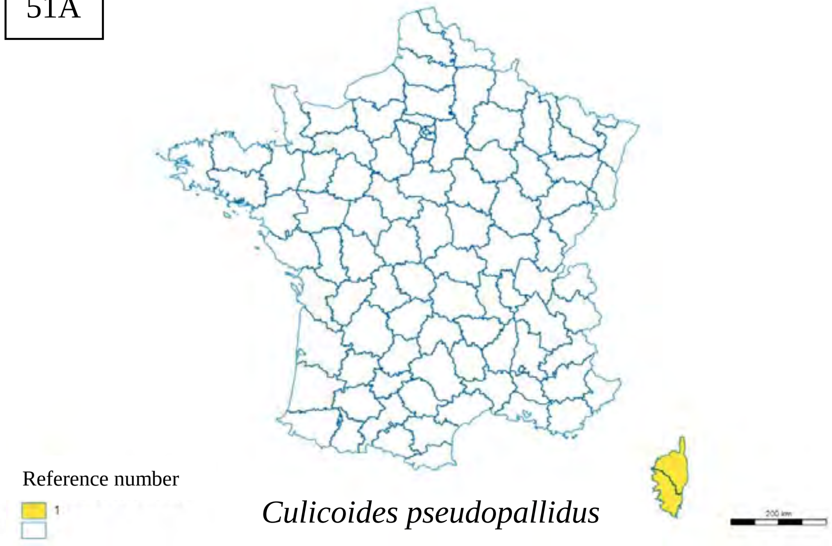

Culicoides pseudopallidus

52A

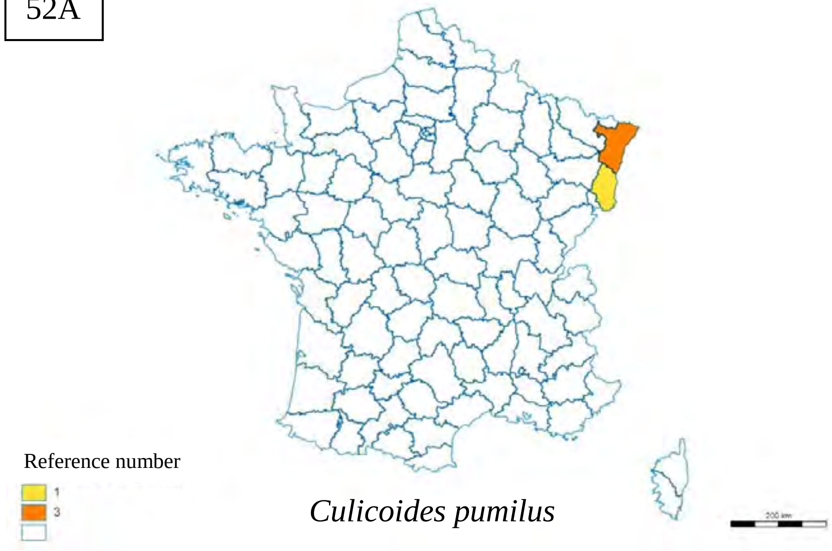

Culicoides pumilus

53A

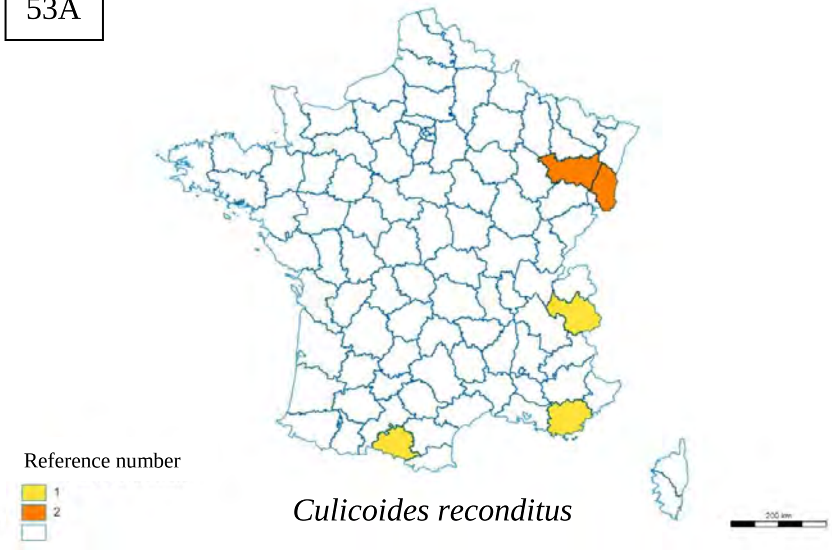

Culicoides reconditus

53B

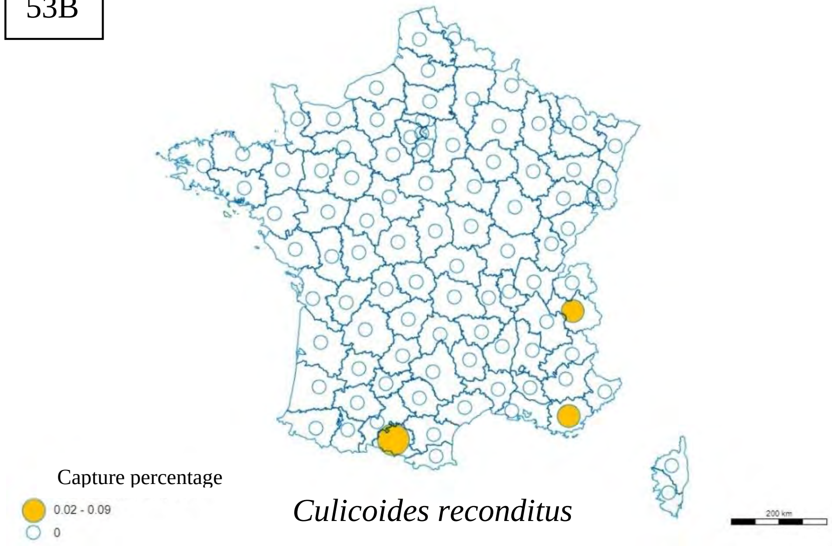

54A

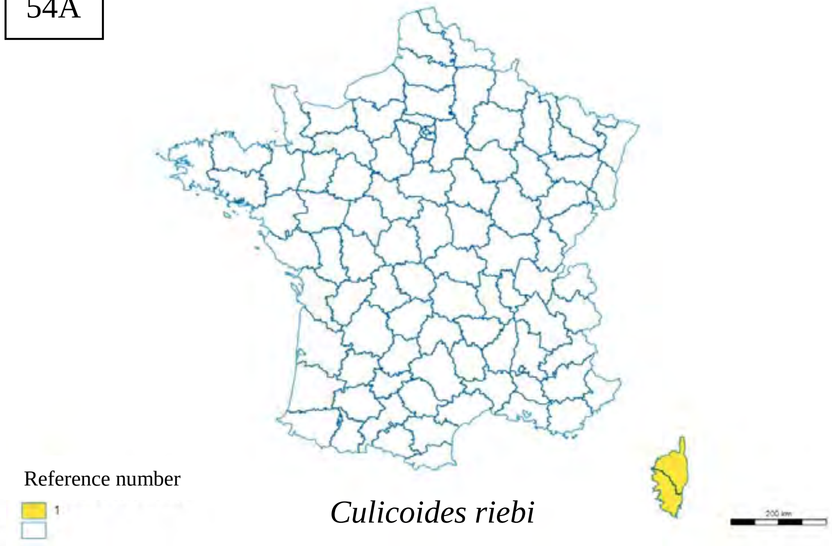

55A

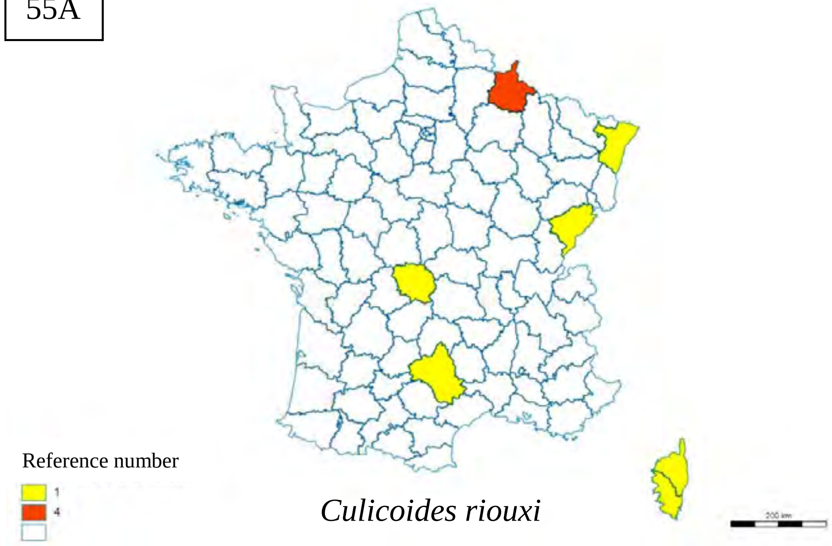

55B

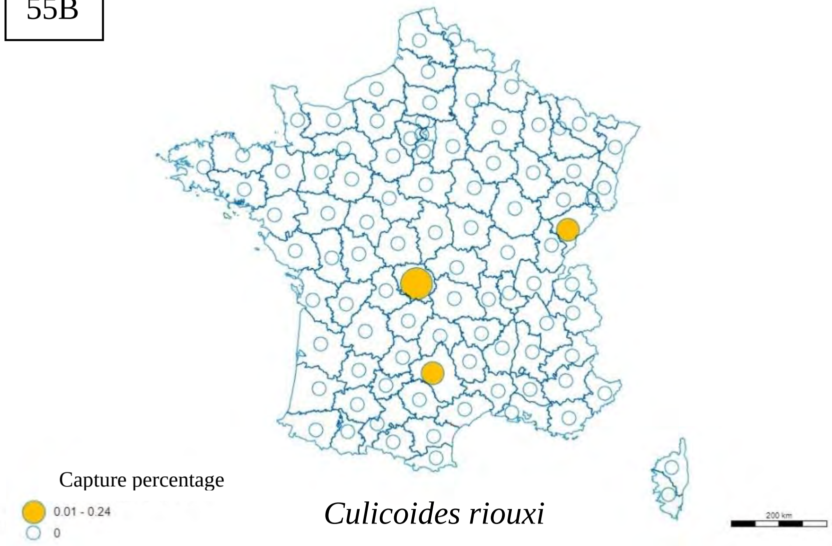

56A

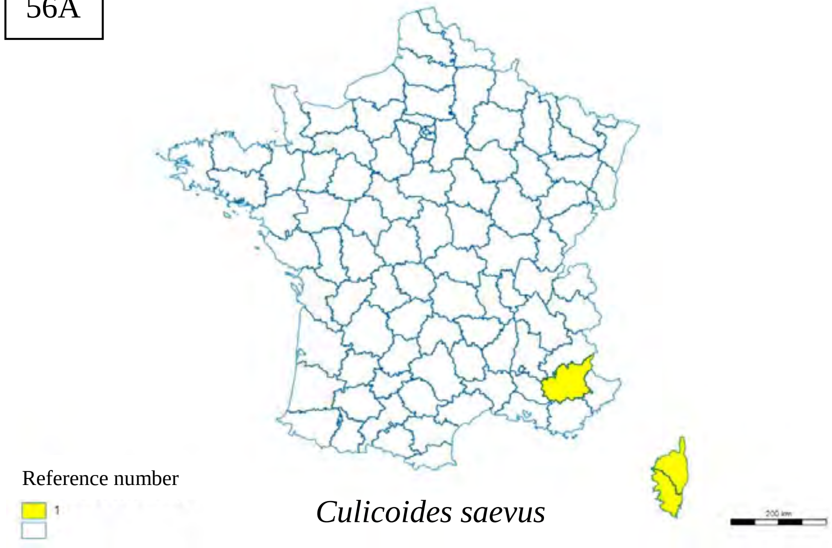

56B

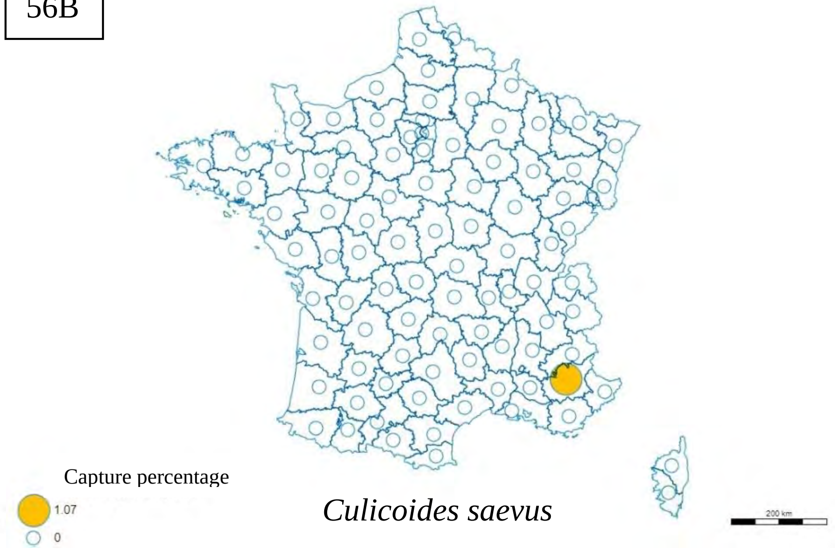

57A

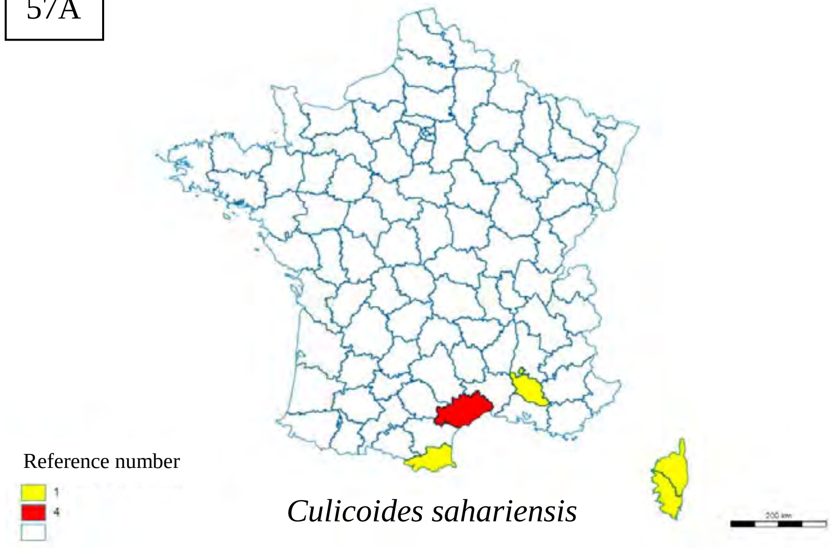

58A

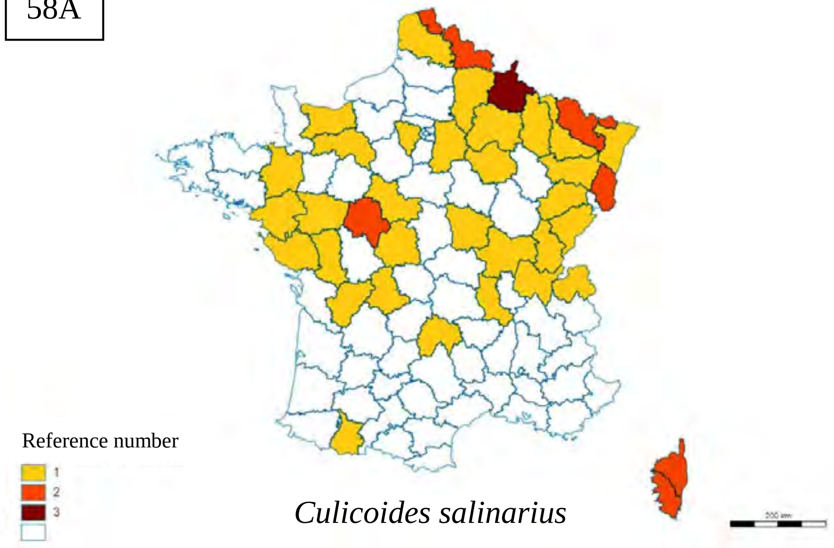

58B

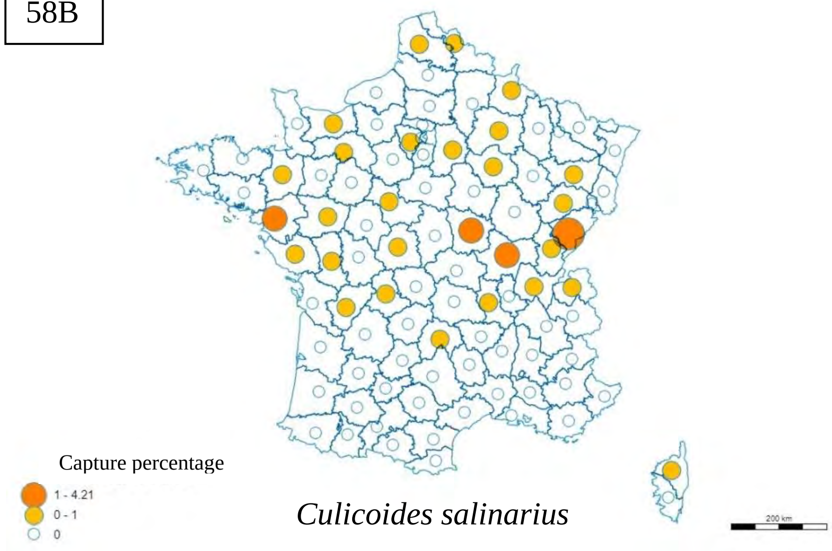

59A

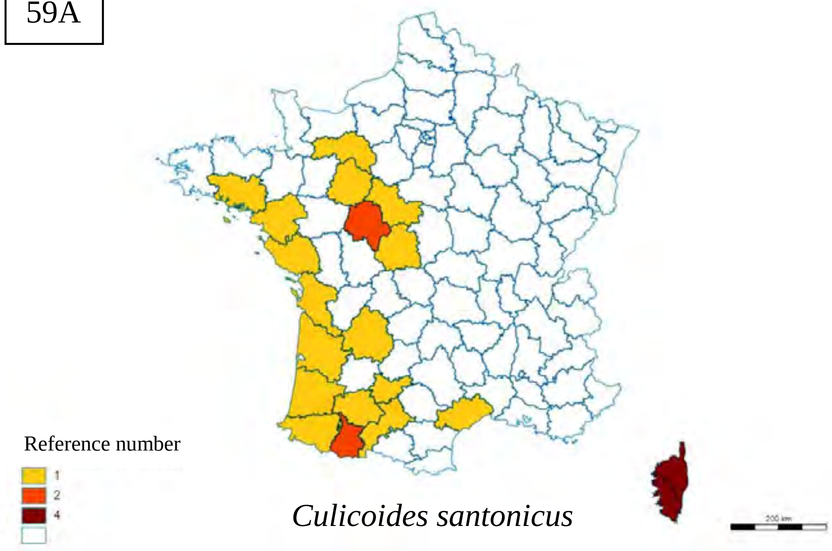

59B

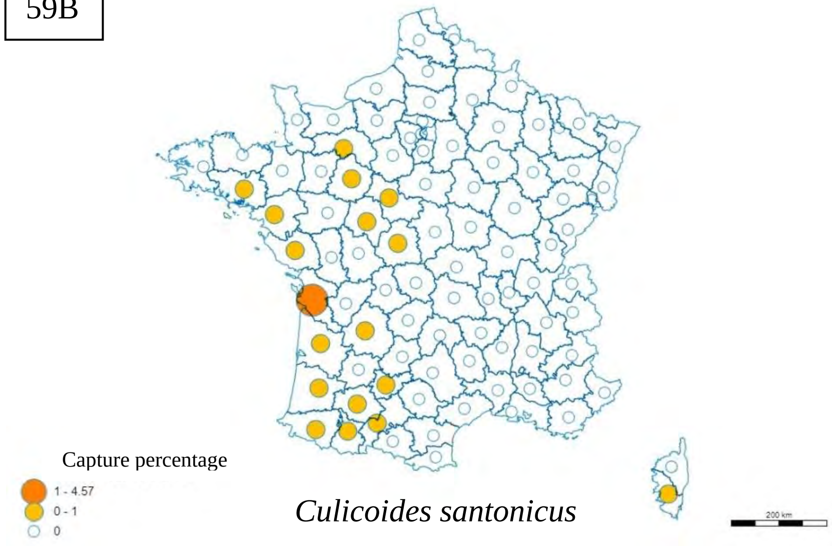

60A

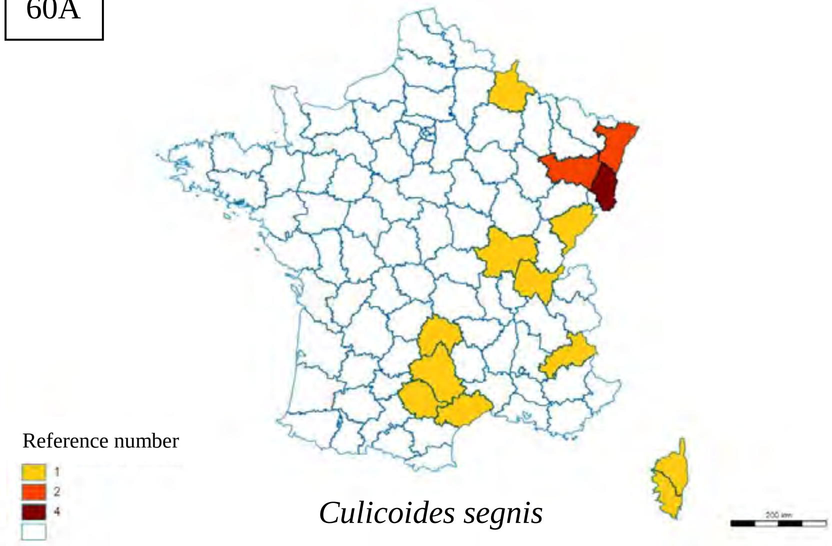

60B

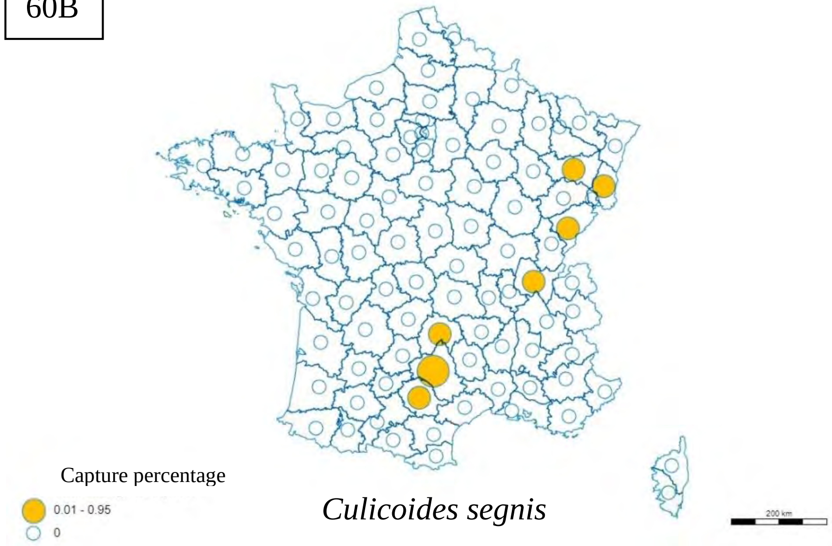

61A

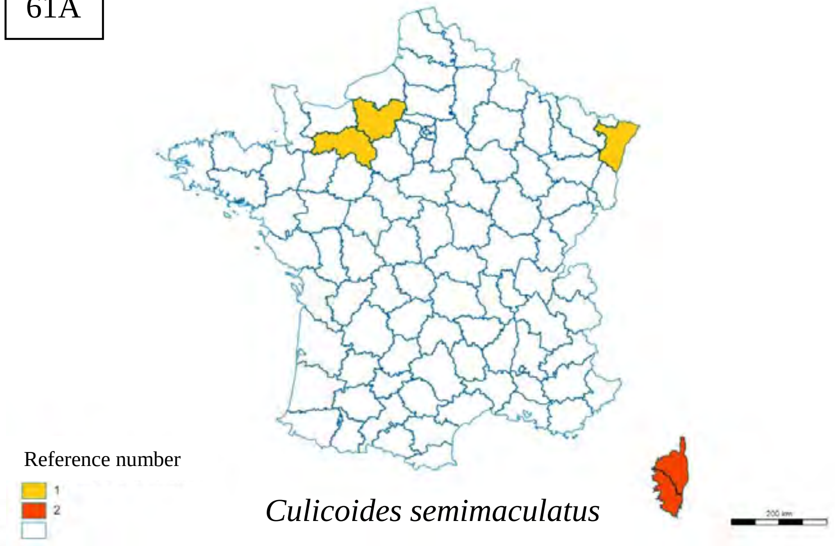

61B

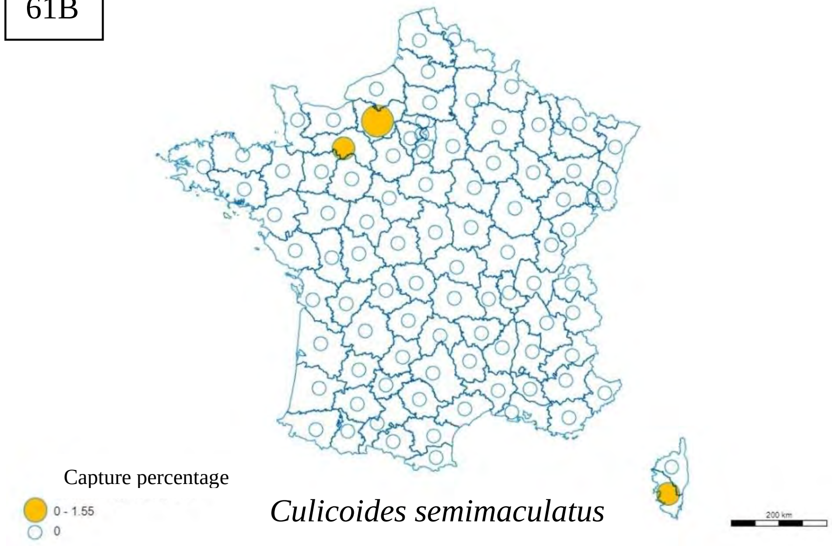

62A

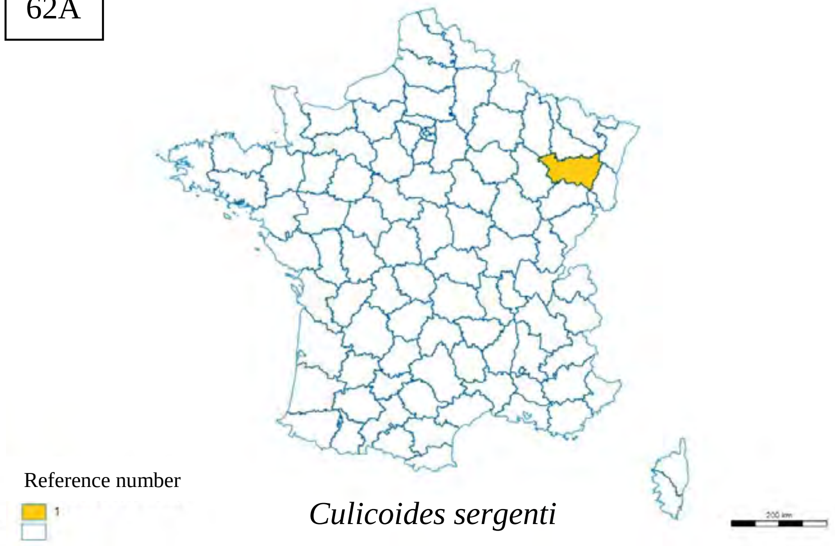

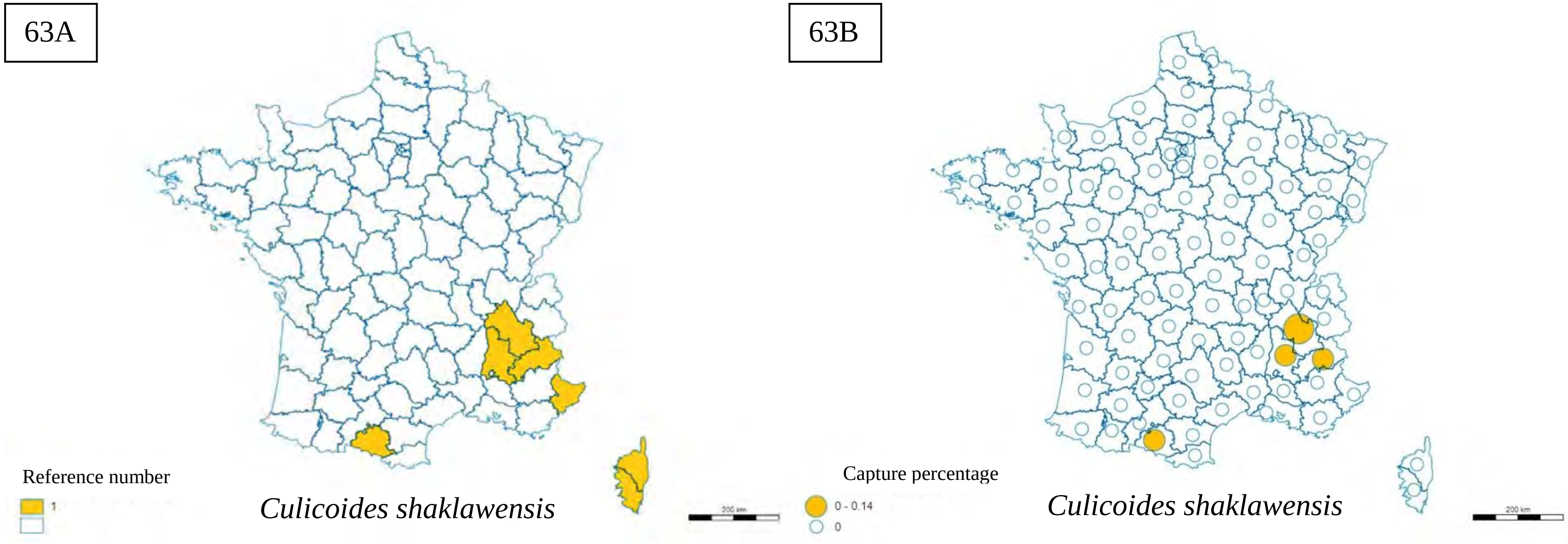
63A
Reference number
1
Culicoides shaklawensis
63B
Capture percentage
0 - 0.14
0
Culicoides shaklawensis

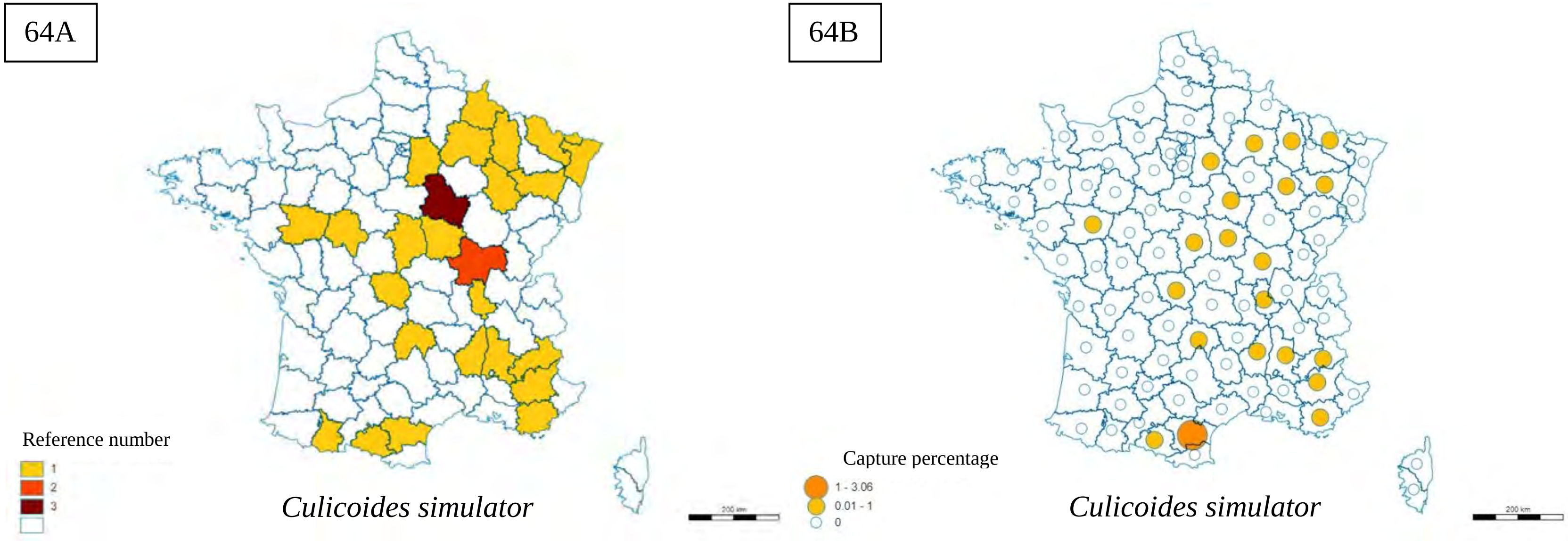
64A
Reference number
1
2
3
Culicoides simulator
64B
Capture percentage
1 - 3.06
0.01 - 1
0
Culicoides simulator

65A

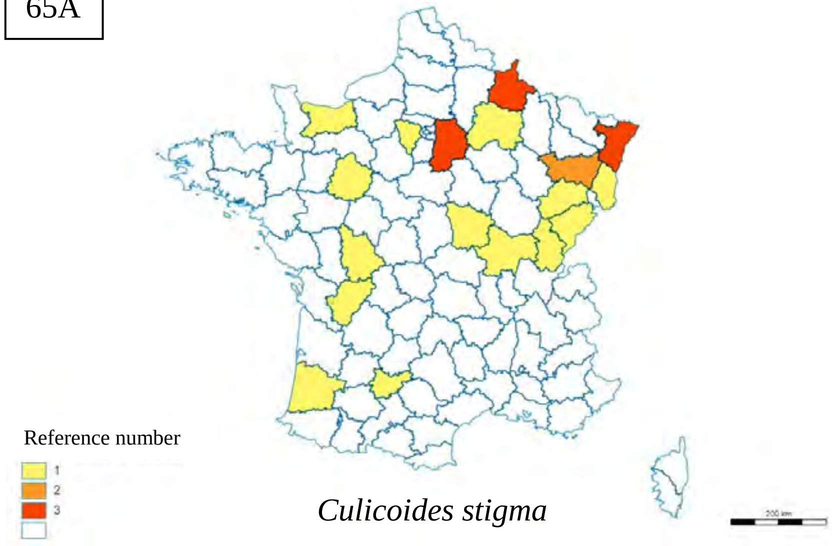

Culicoides stigma

65B

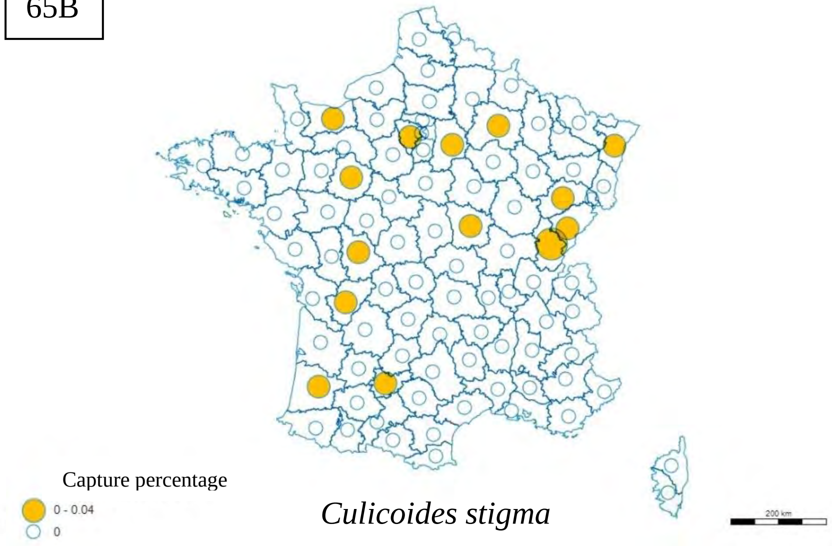

Culicoides stigma

66A

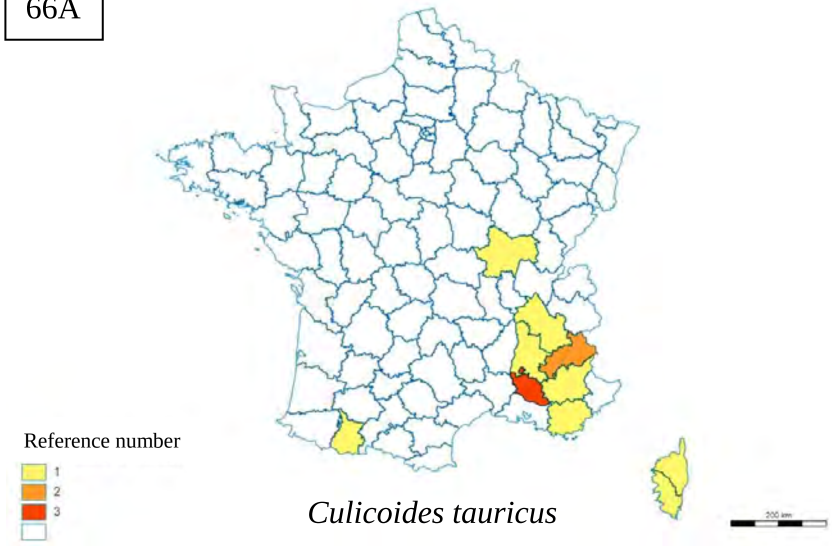

Culicoides tauricus

66B

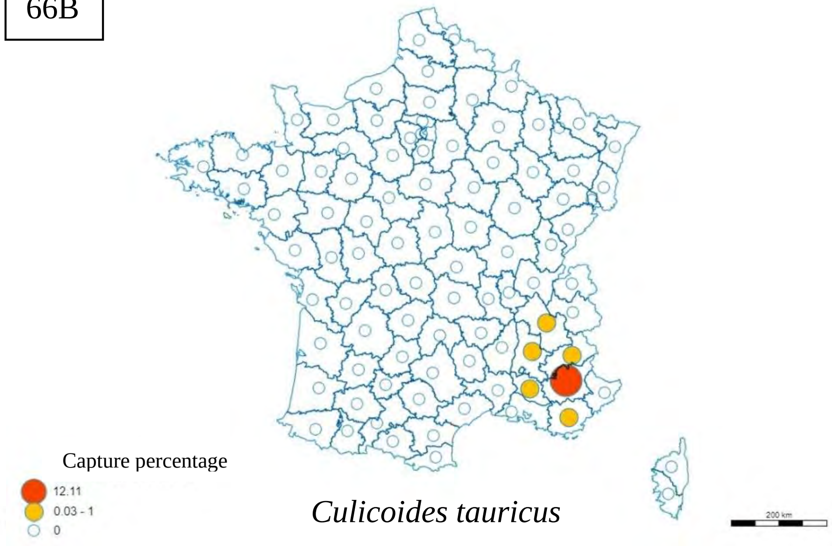

Culicoides tauricus

67A

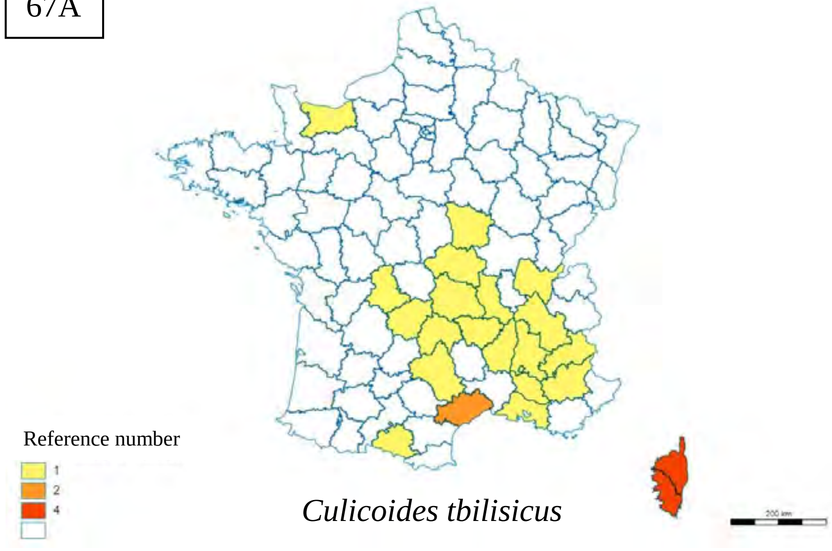

68A

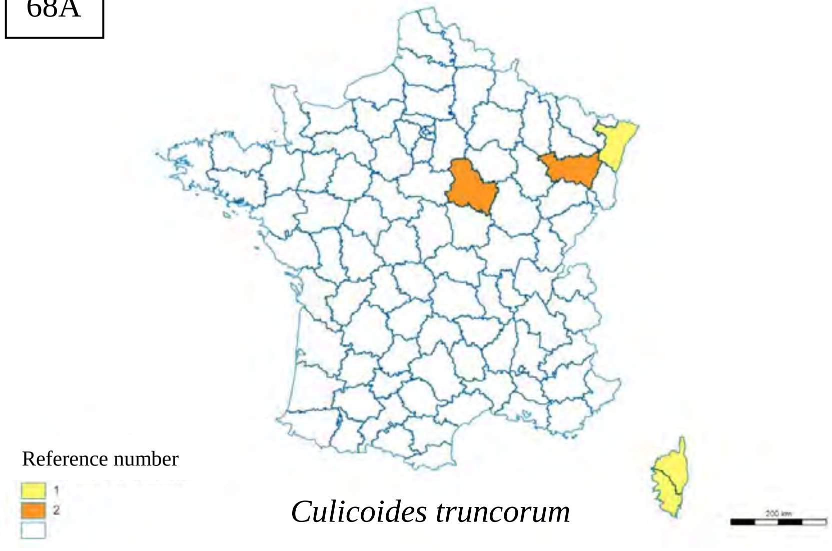

69A

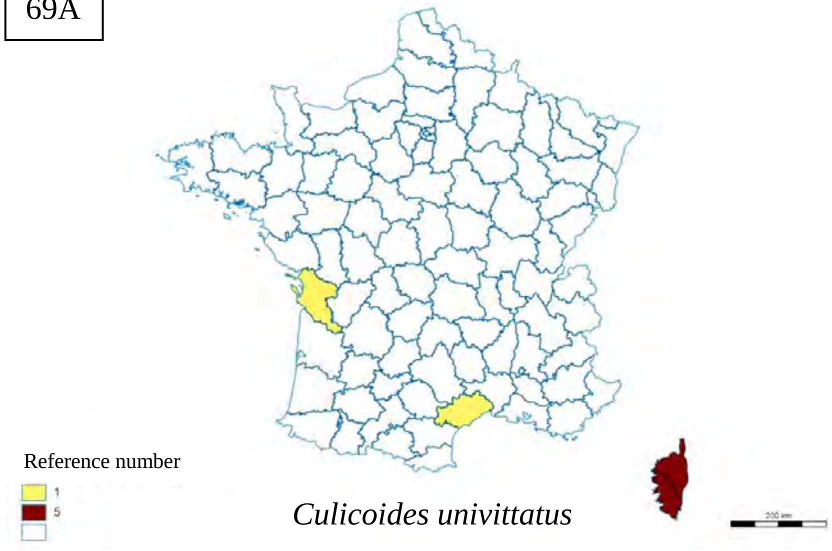

69B

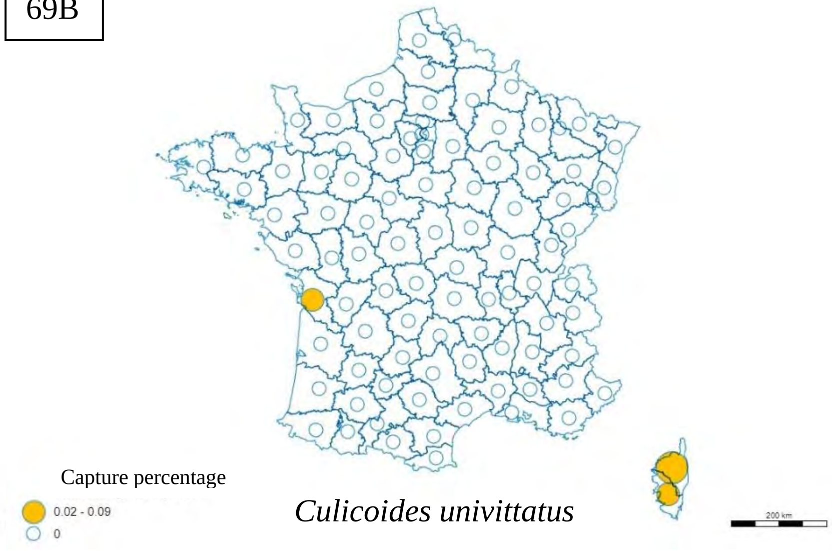

70A

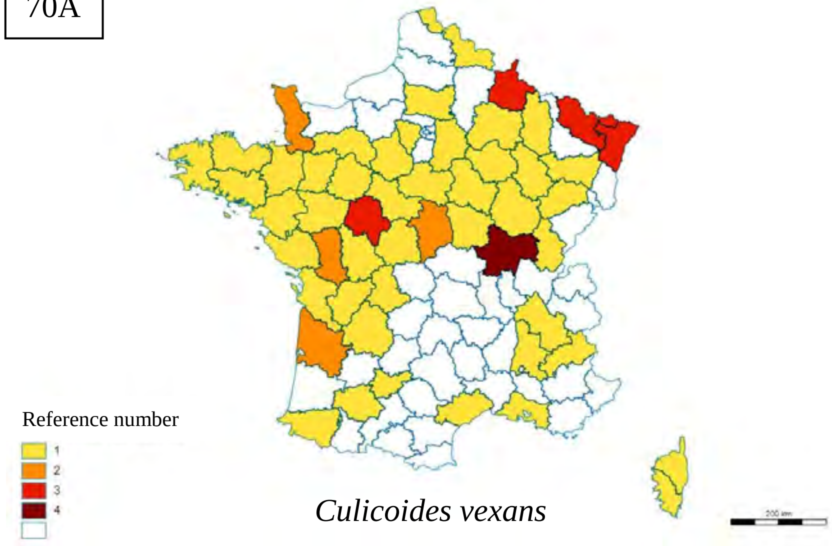

Culicoides vexans

70B

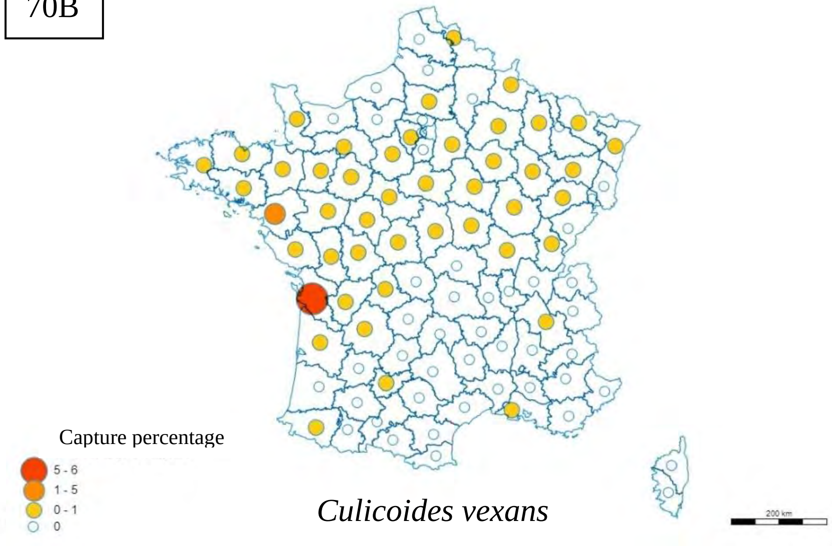

Culicoides vexans

71A

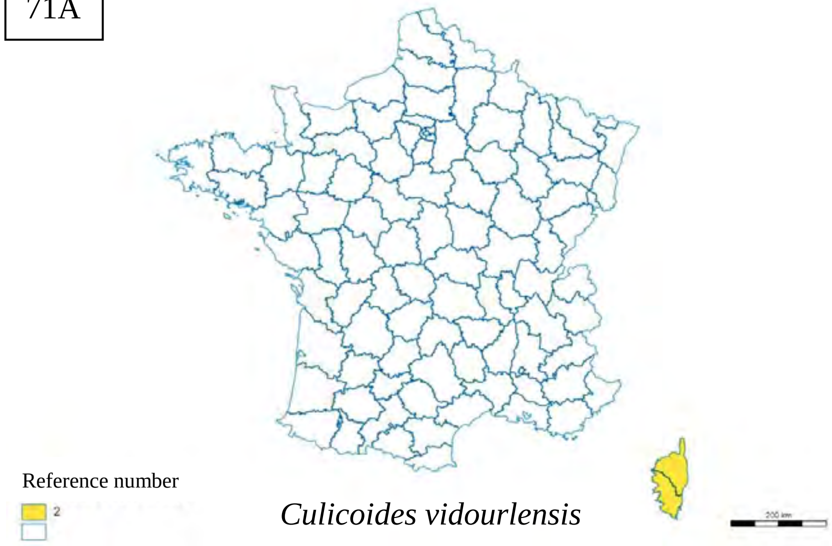

Culicoides vidourlensis

72A

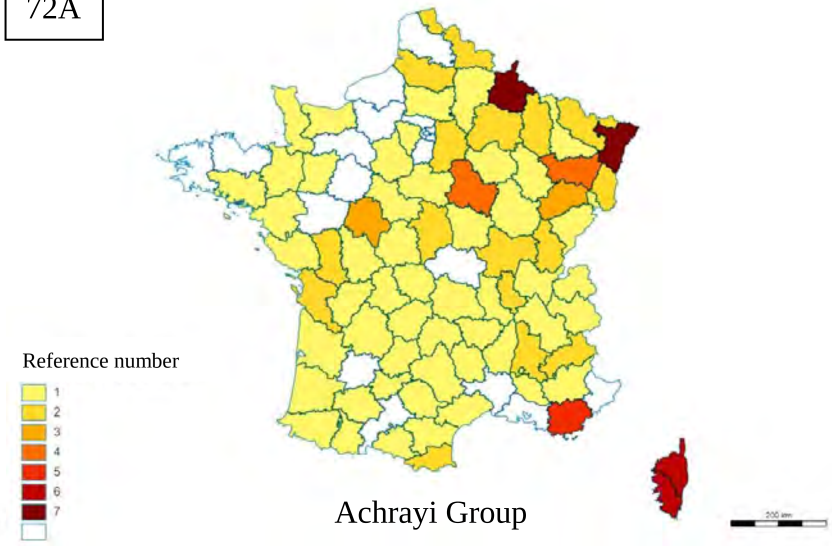

Achrayi Group

72B

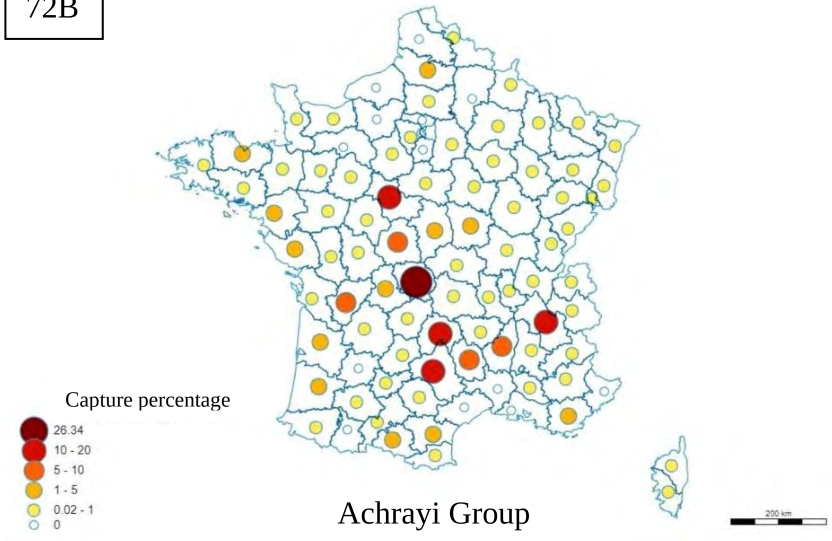

Achrayi Group

73A

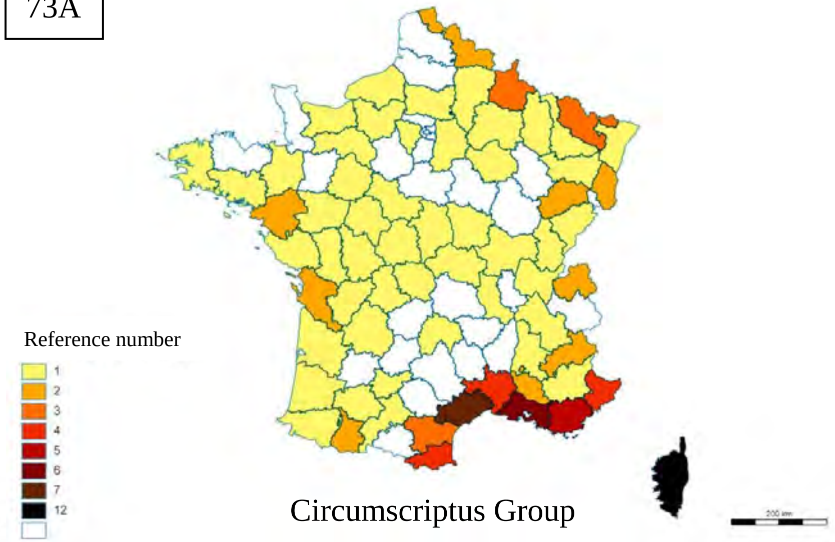

Circumscriptus Group

73B

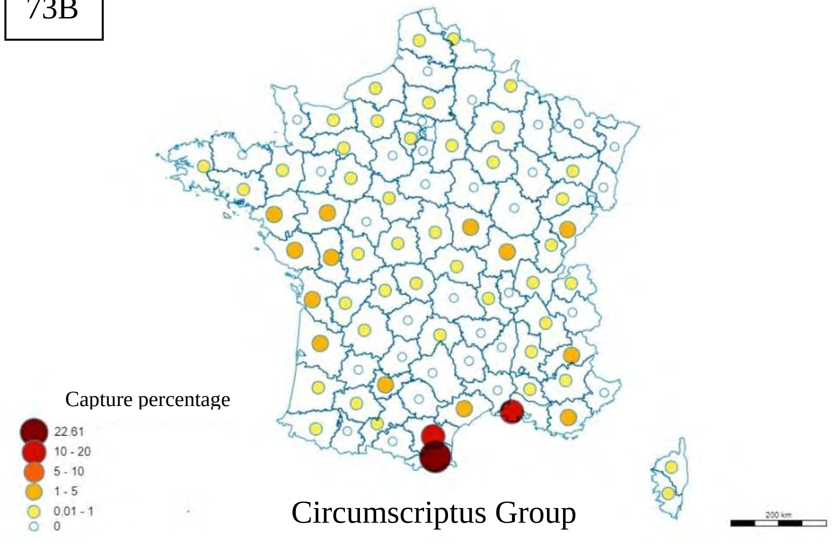

Circumscriptus Group

74A

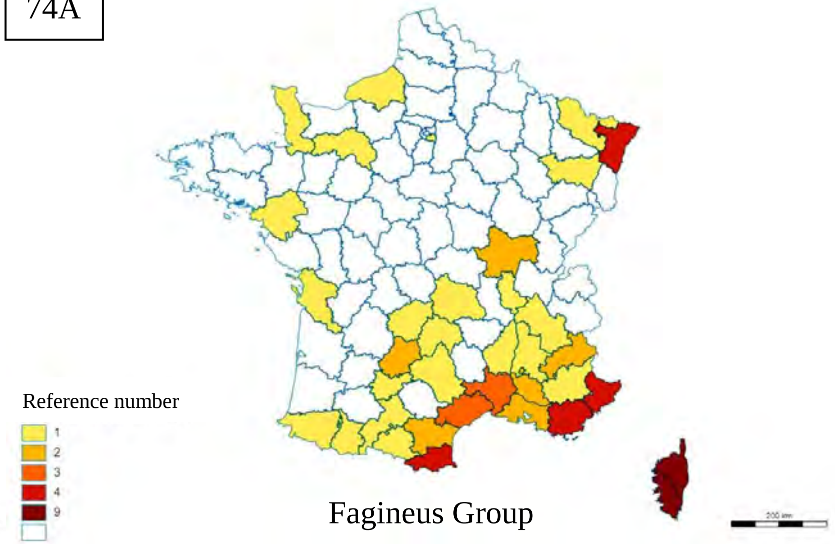

Fagineus Group

74B

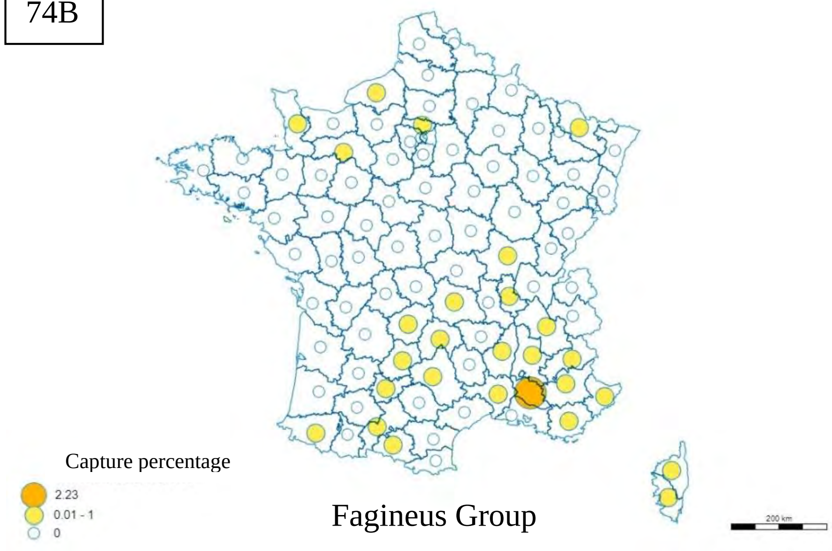

75A

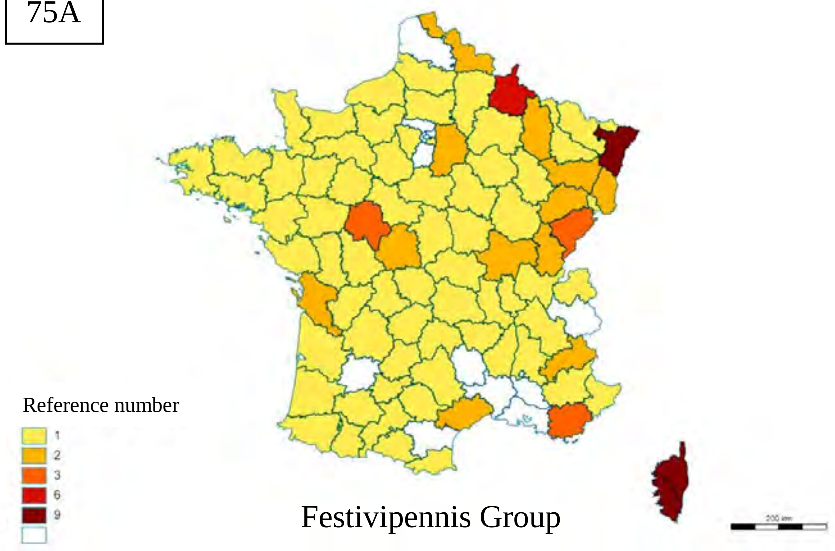

75B

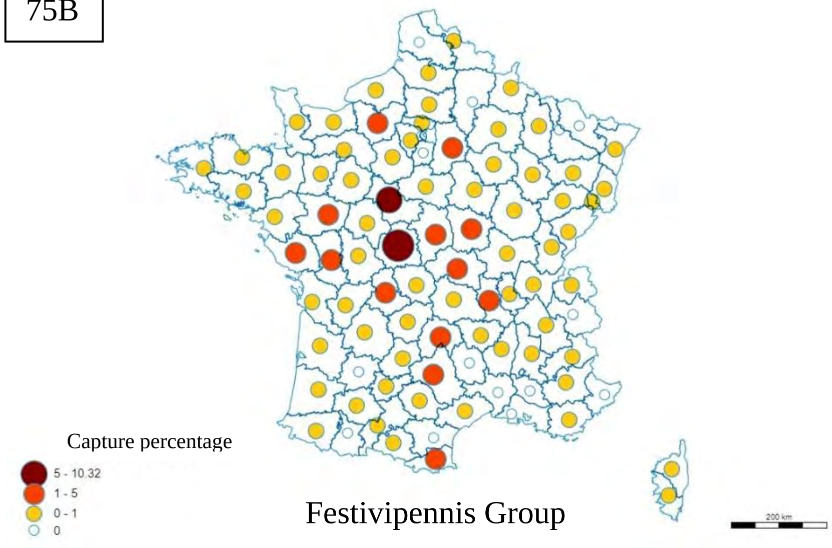

76A

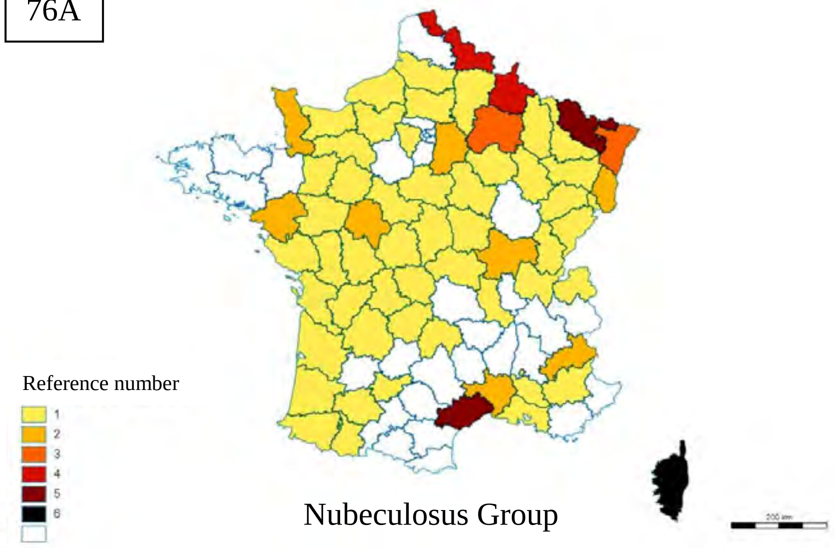

76B

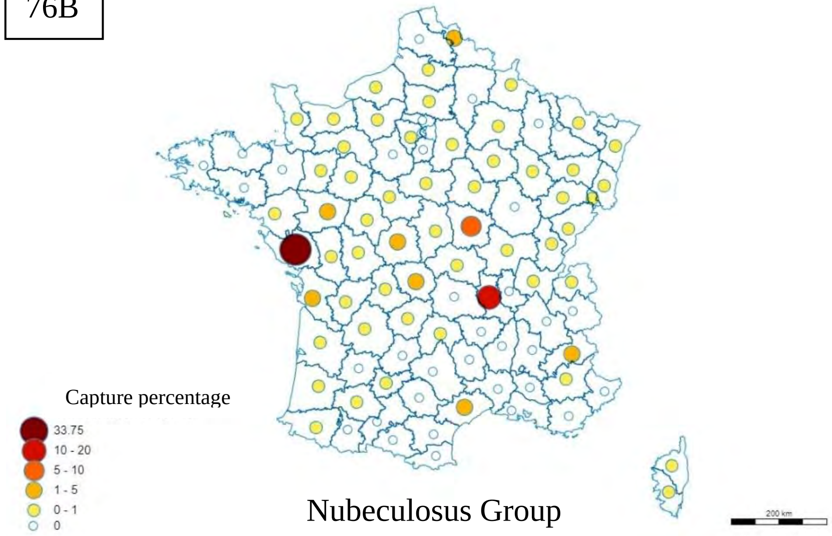

77A

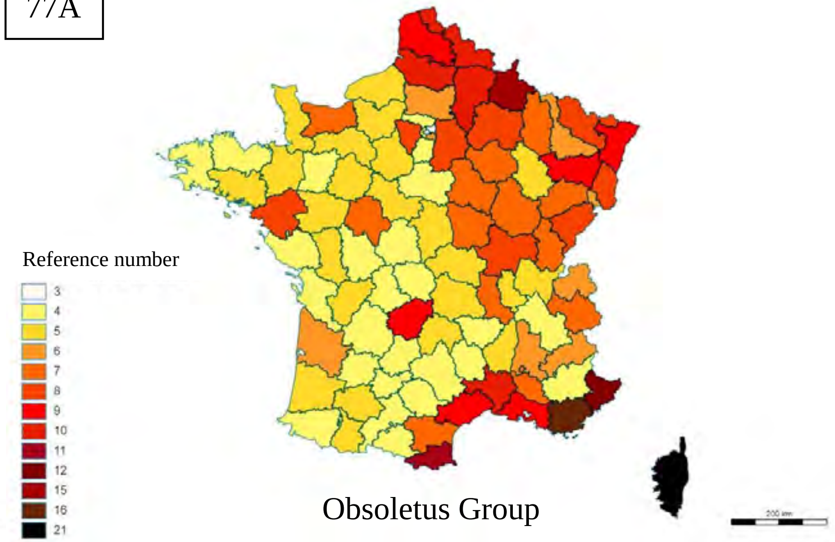

77B

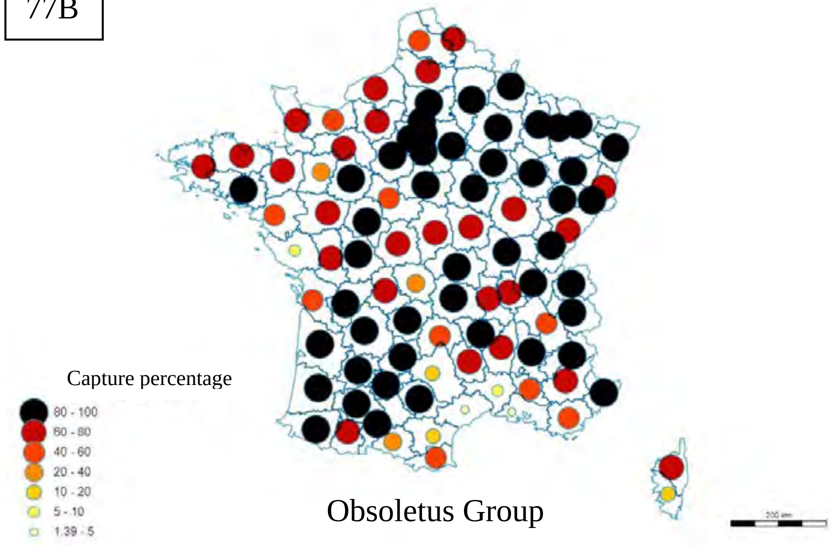

78A

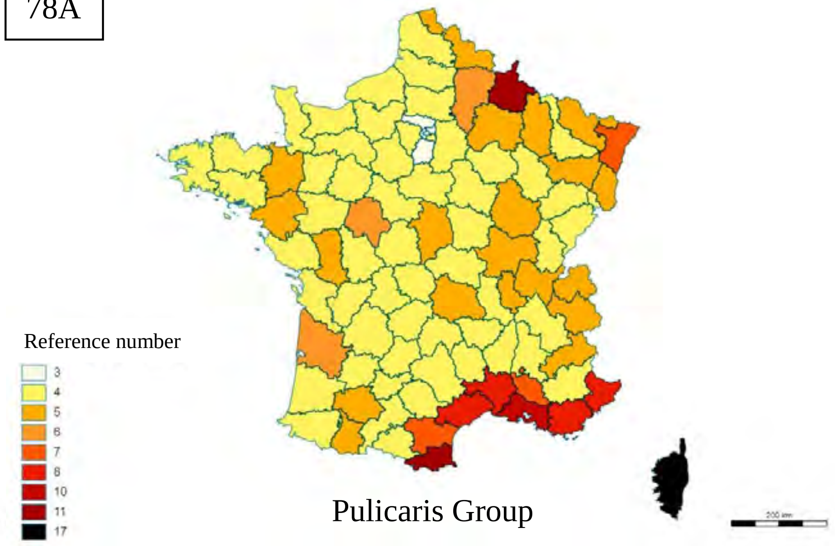

78B

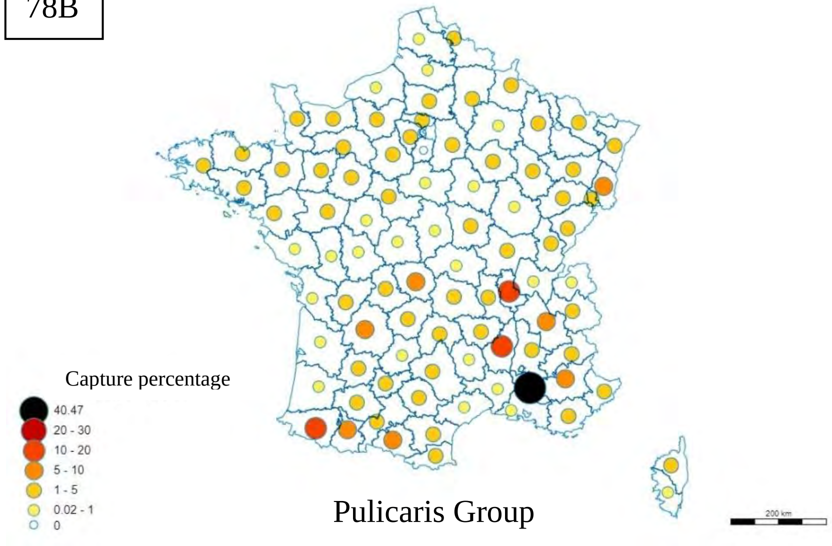

79A

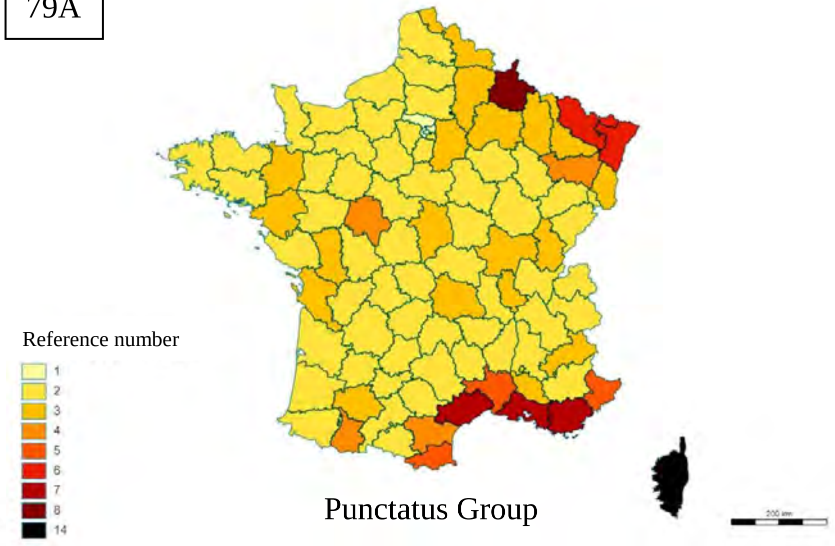

79B

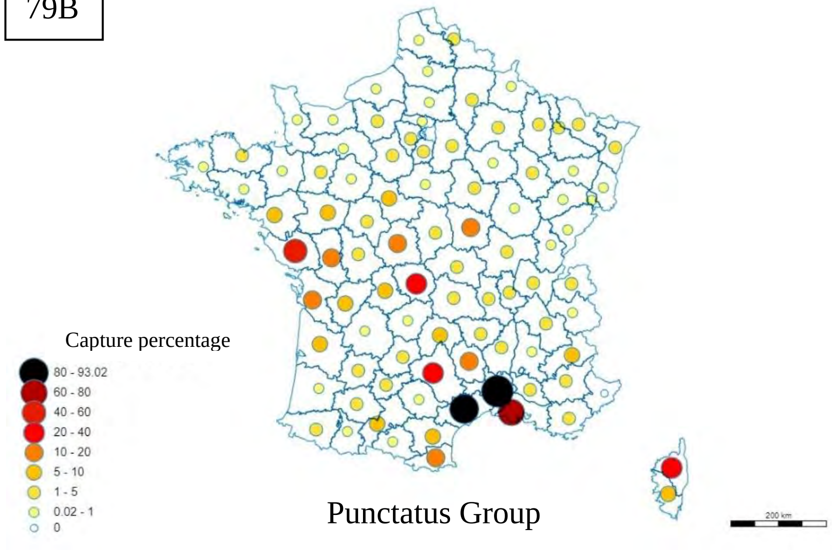

80A

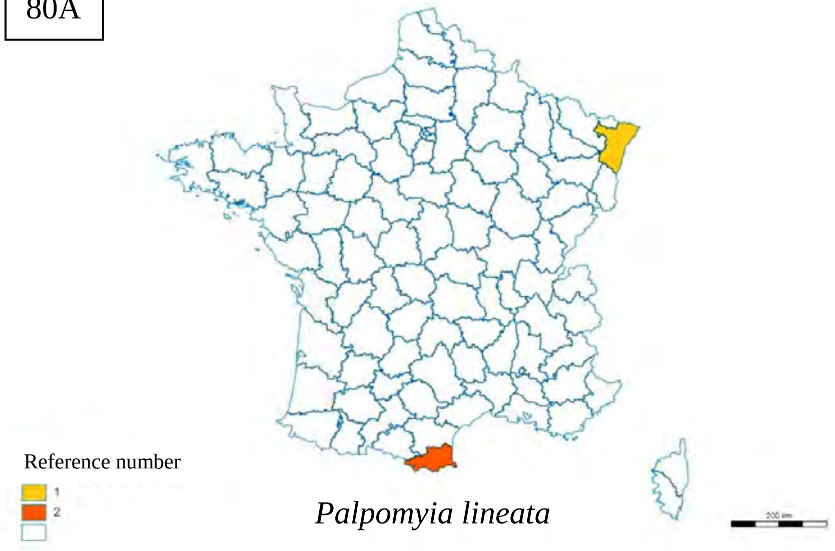

Supplementary Figure S9. Distribution map of *Alluaudomyia needhami* (1A), *Bezzia flavicornis* (2A), *Bezzia pygmaea* (3A), *Culicoides abchazicus* (4A, 4B), *Culicoides accraensis* (5A), *Culicoides alazanicus* (6A, 6B), *Culicoides albicans* (7A), *Culicoides albipennis* (8A), *Culicoides begueti* (9A, 9B), *Culicoides brunnicans* (10A, 10B), *Culicoides cameroni* (11A, 11B), *Culicoides cataneii / gejgelensis* (12A, 12B), *Culicoides caucoliberensis* (13A), *Culicoides chiopterus* (14A, 14B), *Culicoides clintoni* (15A), *Culicoides comosioculatus* (16A), *Culicoides corsicus* (17A), *Culicoides deltus* (18A, 18B), *Culicoides derisor* (19A), *Culicoides dewulfi* (20A, 20B), *Culicoides duddingstoni* (21A, 21B), *Culicoides dzhafarovi* (22A), *Culicoides furcillatus* (23A, 23B), *Culicoides gornostaevae* (24A), *Culicoides griseidorsum* (25A, 25B), *Culicoides grisescens* (26A, 26B), *Culicoides haranti* (27A, 27B), *Culicoides heliophilus* (28A, 28B), *Culicoides heteroclitus* (29A, 29B), *Culicoides ibericus* (30A), *Culicoides imicola* (31A, 31B), *Culicoides impunctatus* (32A, 32B), *Culicoides indistinctus* (33A, 33B), *Culicoides jumineri* (34A, 34B), *Culicoides jurensis* (35A), *Culicoides kibunensis* (36A, 36B), *Culicoides kurensis* (37A, 37B), *Culicoides longipennis* (38A, 38B), *Culicoides malevillei* (39A), *Culicoides manchuriensis* (40A, 40B), *Culicoides maritimus* (41A, 41B), *Culicoides minutissimus* (42A, 42B), *Culicoides montanus* (43A), *Culicoides odiatus* (44A, 44B), *Culicoides paradisionensis* (45A, 45B), *Culicoides paradoxalis* (46A), *Culicoides parroti* (47A, 47B), *Culicoides pictipennis* (48A, 48B), *Culicoides poperinghensis* (49A, 49B), *Culicoides pseudoheliophilus* (50A), *Culicoides pseudopallidus* (51A), *Culicoides pumilus* (52A), *Culicoides reconditus* (53A, 53B), *Culicoides riebi* (54A), *Culicoides riouxi* (55A, 55B), *Culicoides saevus* (56A, 56B), *Culicoides sahariensis* (57A), *Culicoides salinarius* (58A, 58B), *Culicoides santonicus* (59A, 59B), *Culicoides segnis* (60A, 60B), *Culicoides semimaculatus* (61A, 61B), *Culicoides sergenti* (62A), *Culicoides shaklawensis* (63A, 63B), *Culicoides simulator* (64A, 64B), *Culicoides stigma* (65A, 65B), *Culicoides tauricus* (66A, 66B), *Culicoides tbilisicus* (67A), *Culicoides truncorum* (68A), *Culicoides univittatus* (69A, 69B), *Culicoides vexans* (70A, 70B), *Culicoides vidourlensis* (71A), Achrayi Group (72A, 72B), Circumscriptus Group (73A, 73B), Fagineus Group (74A, 74B), Festivipennis Group (75A, 75B), Nubeculosus Group (76A, 76B), Obsoletus Group (77A, 77B), Pulicaris Group (78A, 78B), Punctatus Group (79A, 79B) and *Palpomyia lineata* (80A) by department according to the number of references (A) and, if available, the percentage of capture (B).

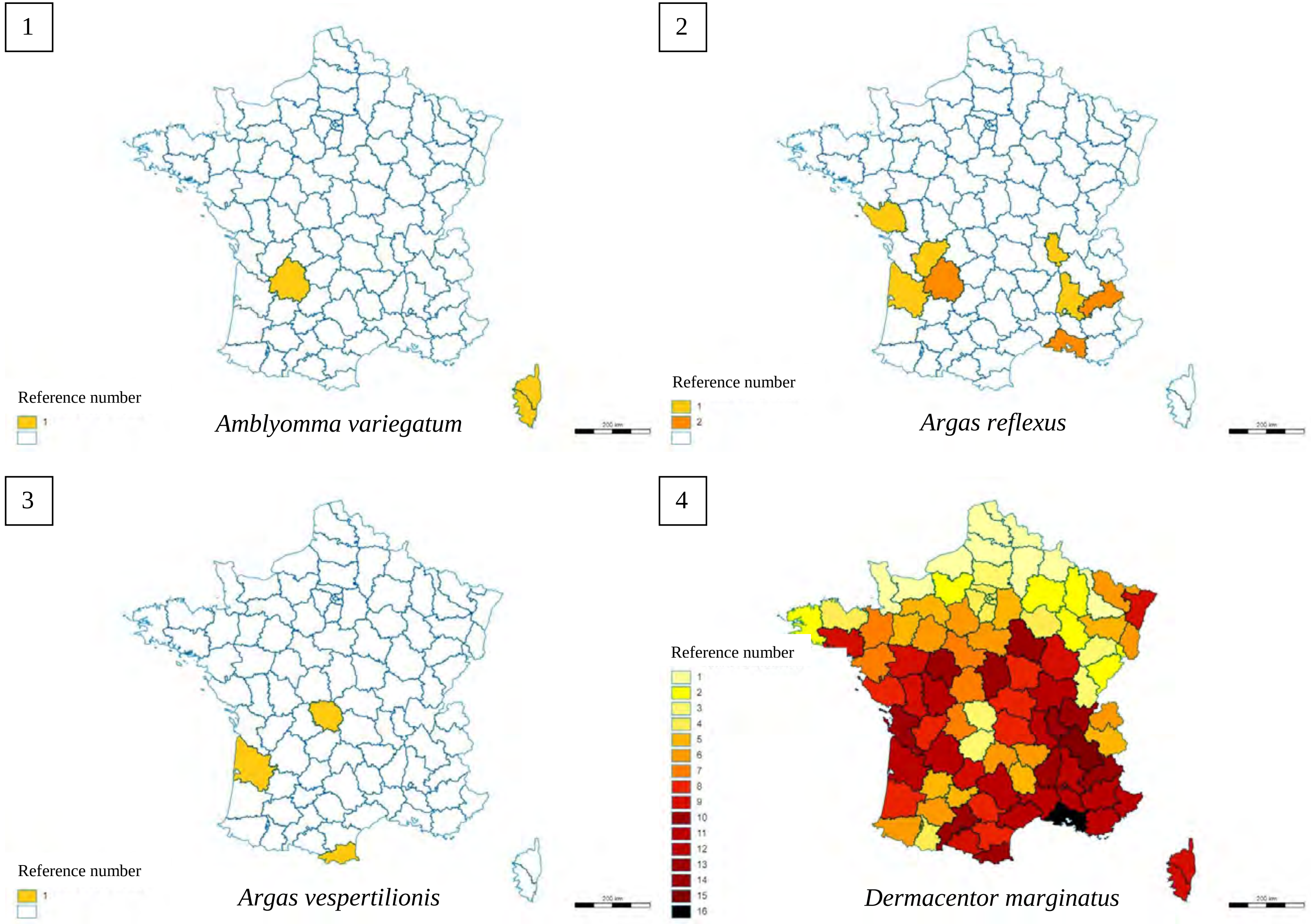
1
Reference number
1
Amblyomma variegatum
2
Reference number
1
2
Argas reflexus
3
Reference number
1
Argas vespertilionis
4
Reference number
1
2
3
4
5
6
7
8
9
10
11
12
13
14
15
16
Dermacentor marginatus

5

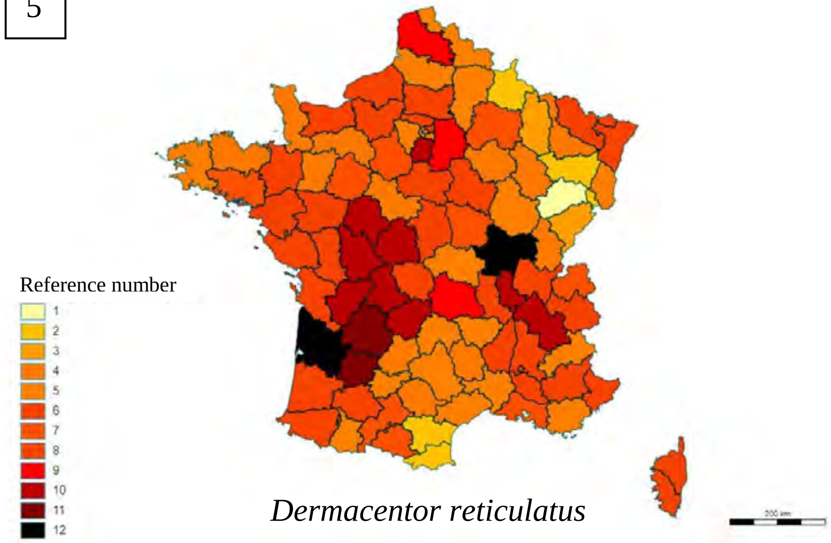

Dermacentor reticulatus

6

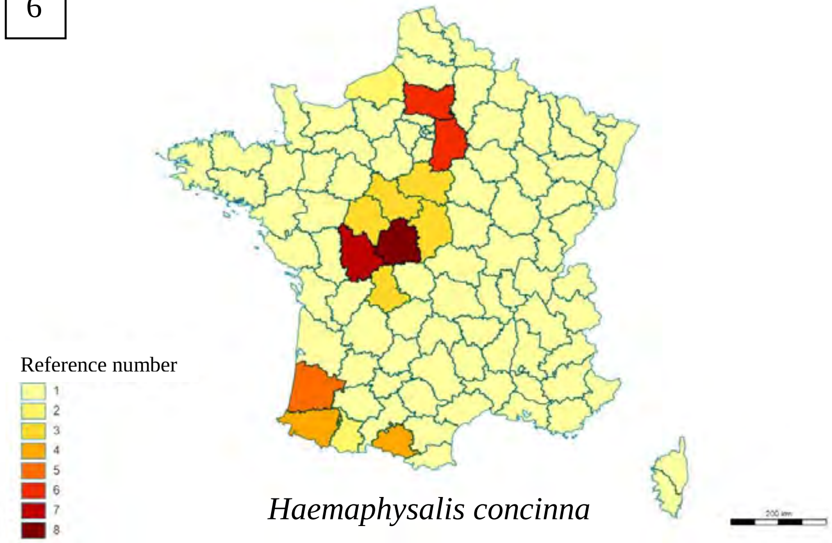

Haemaphysalis concinna

7

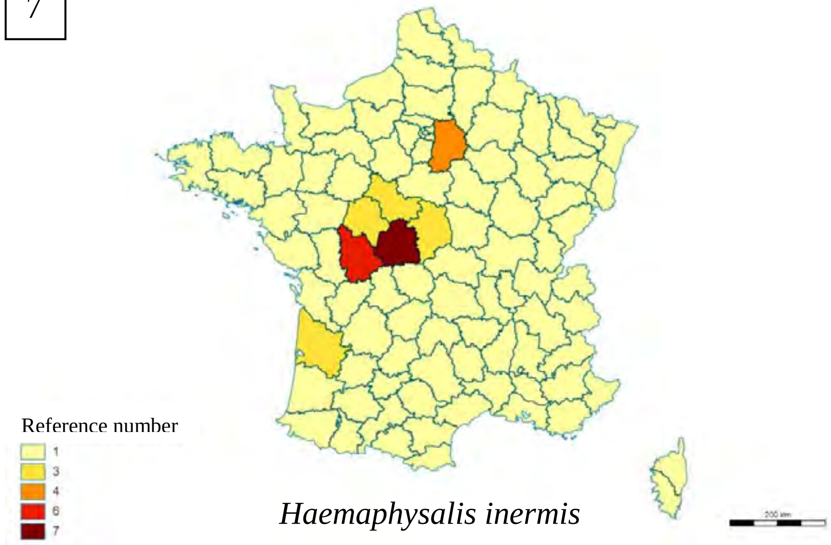

Haemaphysalis inermis

8

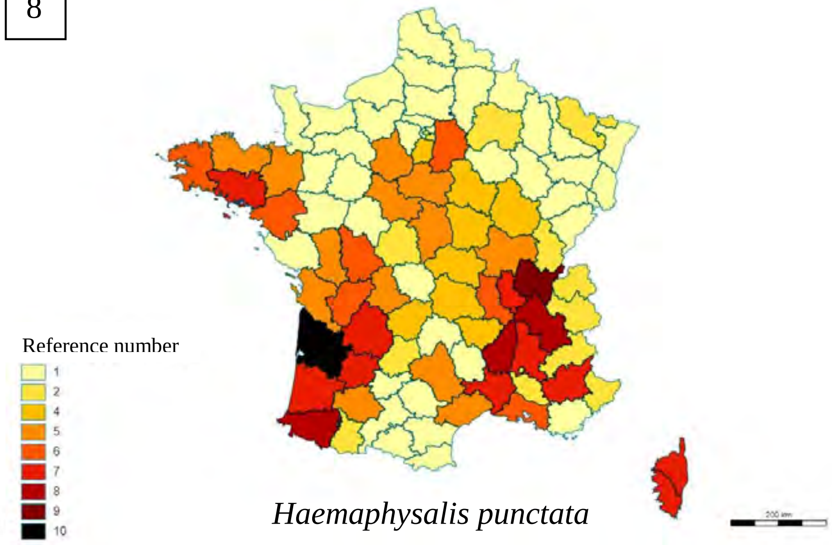

Haemaphysalis punctata

9

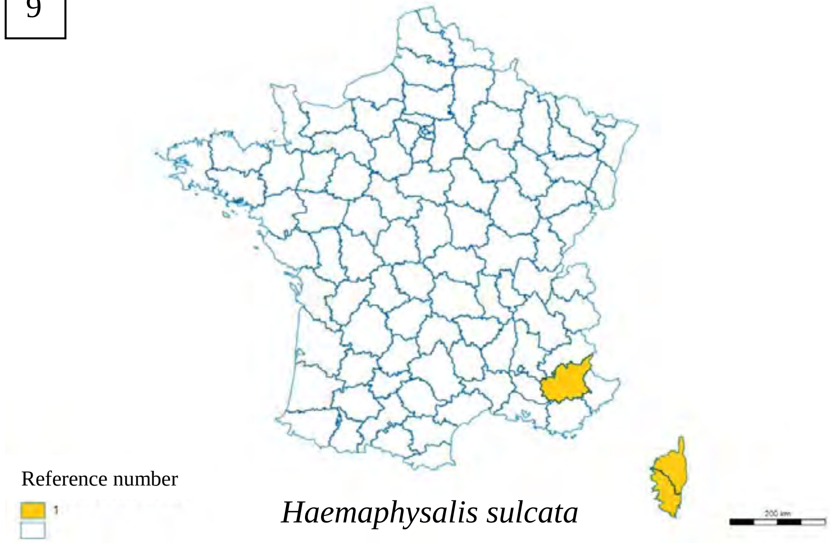

Haemaphysalis sulcata

10

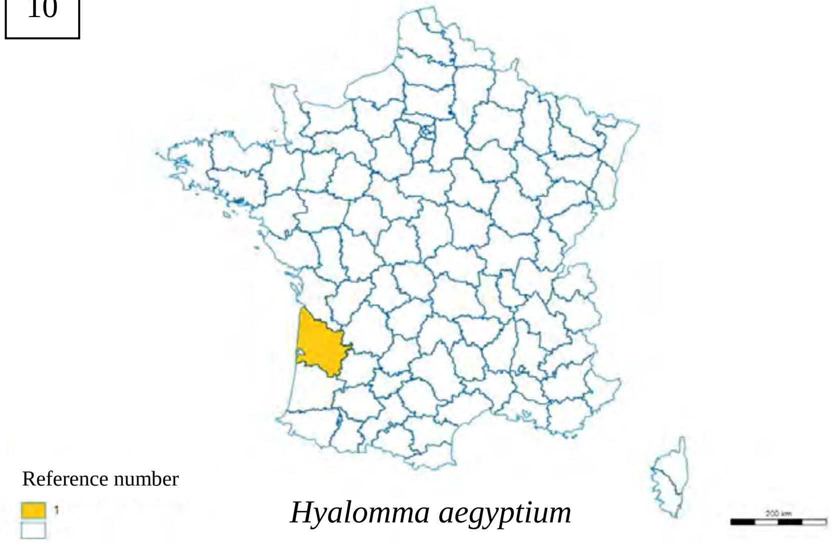

Hyalomma aegyptium

11

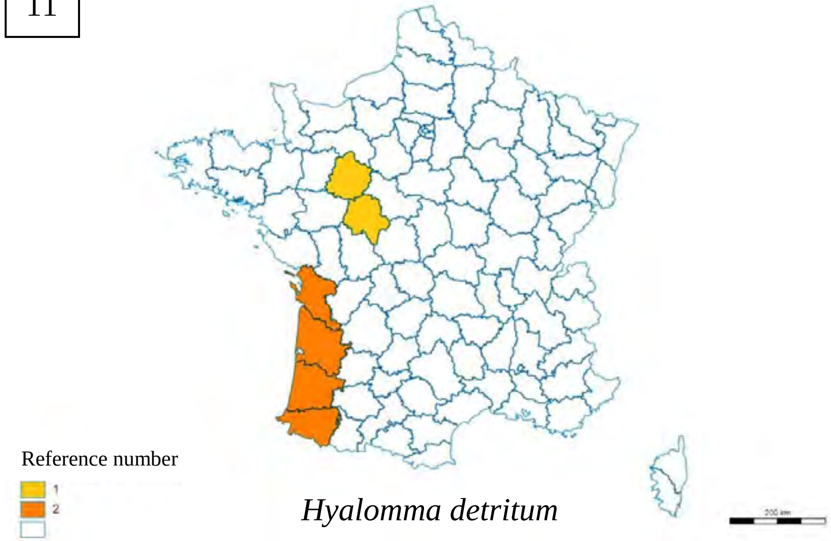

Hyalomma detritum

12

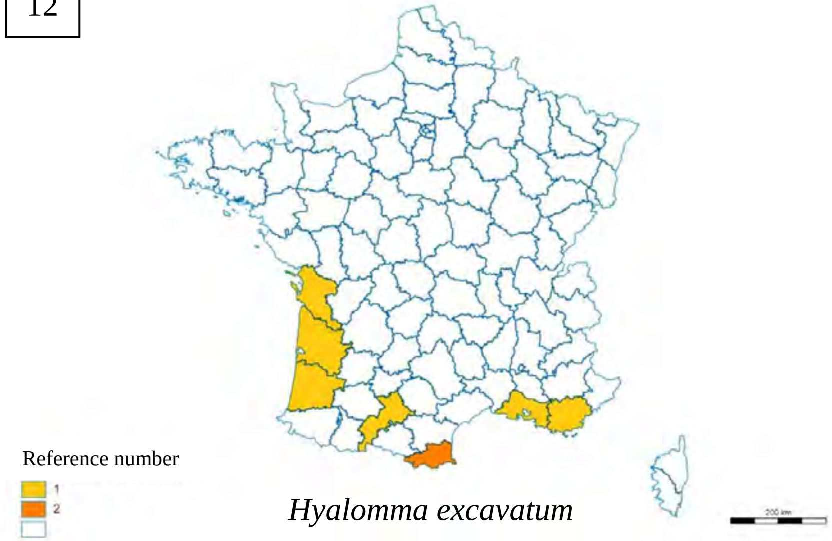

Hyalomma excavatum

13

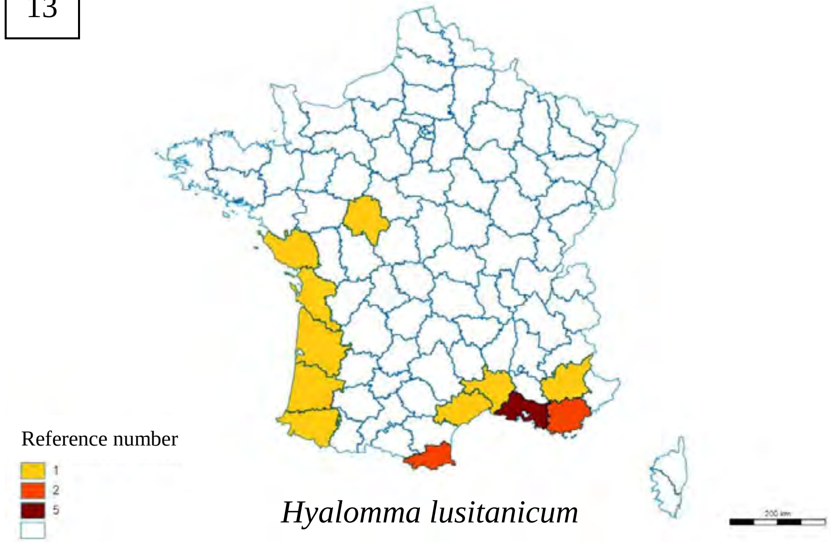

Hyalomma lusitanicum

14

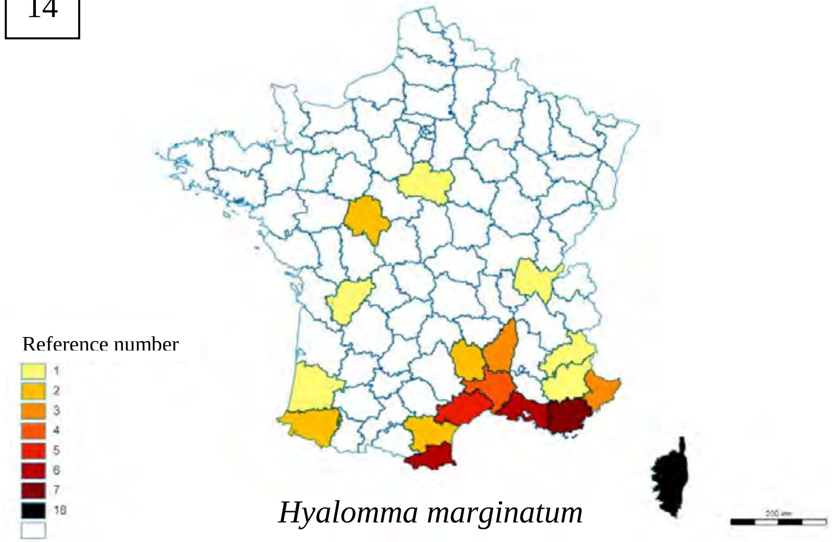

Hyalomma marginatum

15

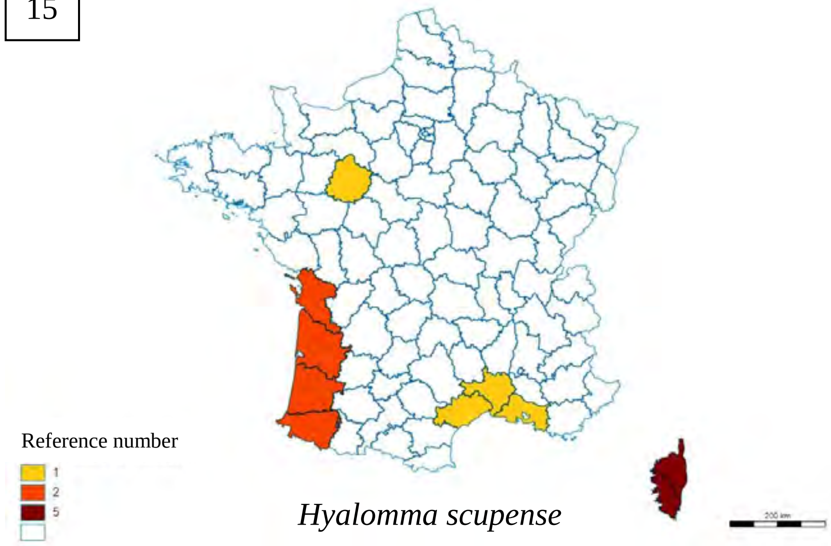

Hyalomma scupense

16

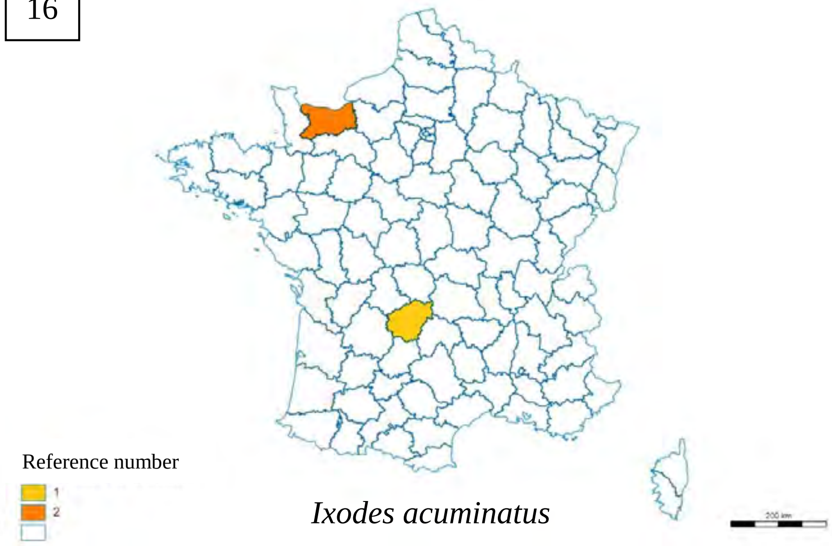

Ixodes acuminatus

17

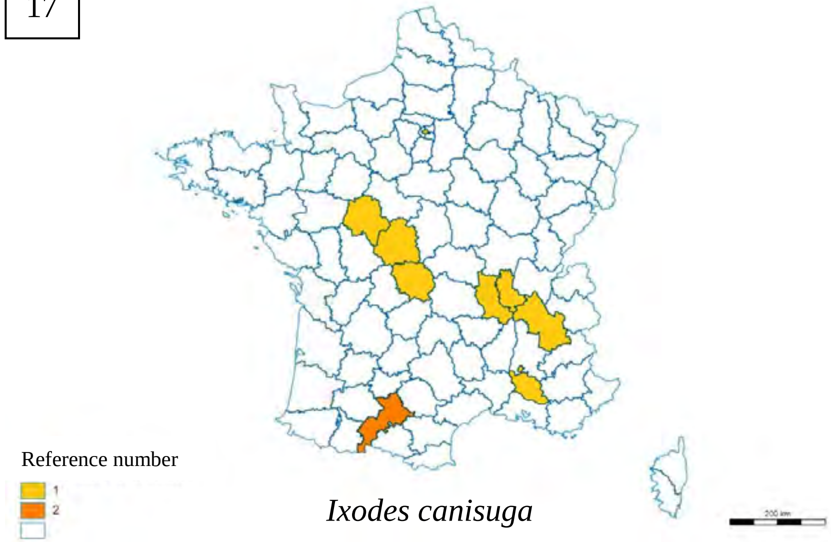

Ixodes canisuga

18

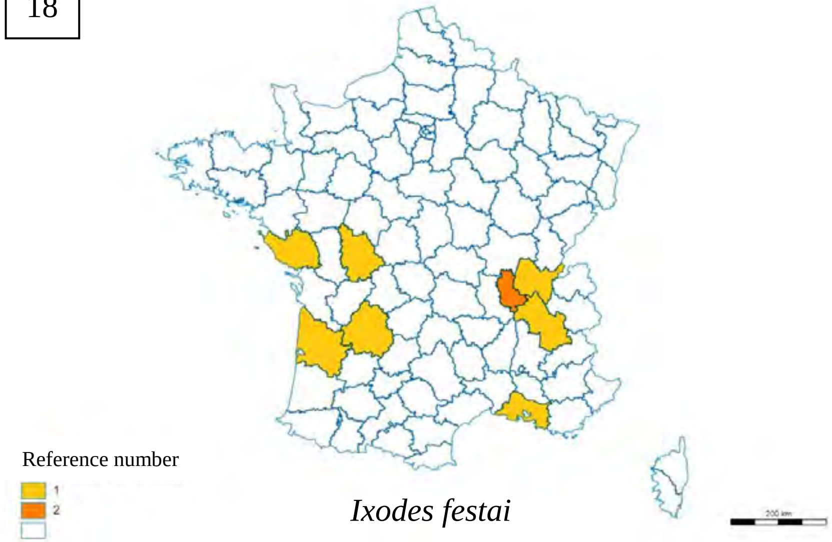

Ixodes festai

19

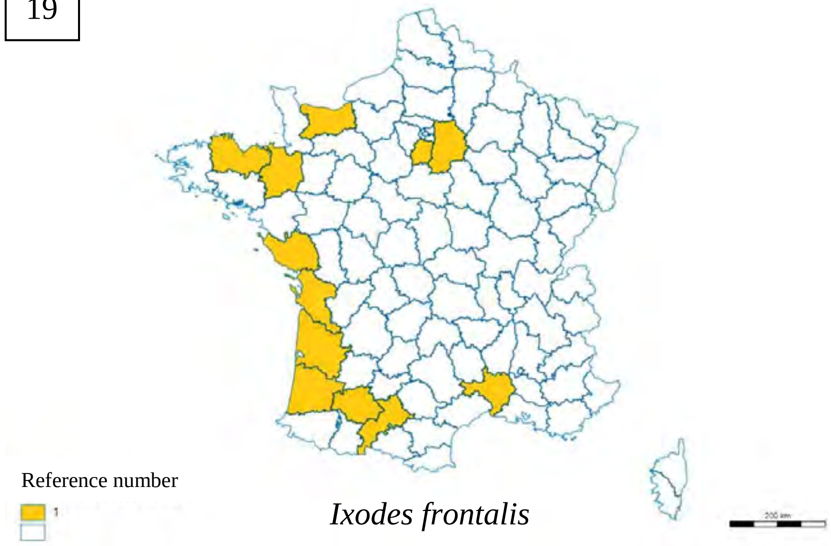

Ixodes frontalis

20

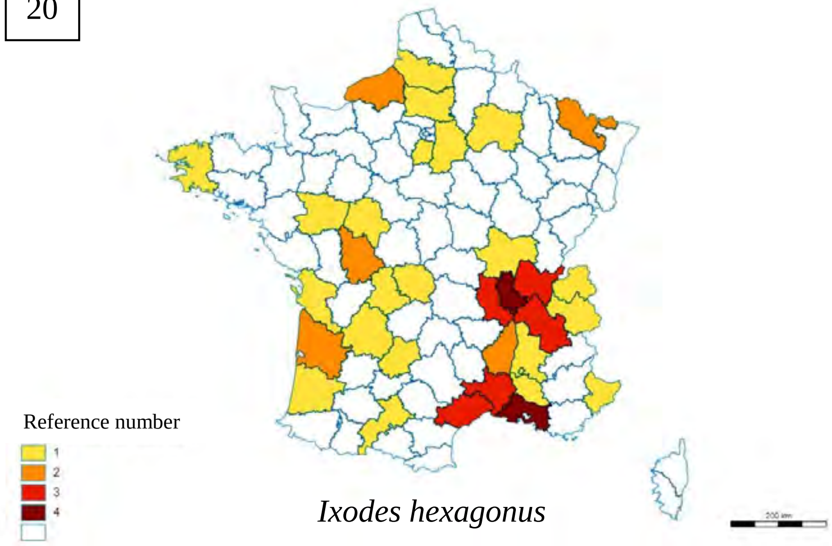

Ixodes hexagonus

21

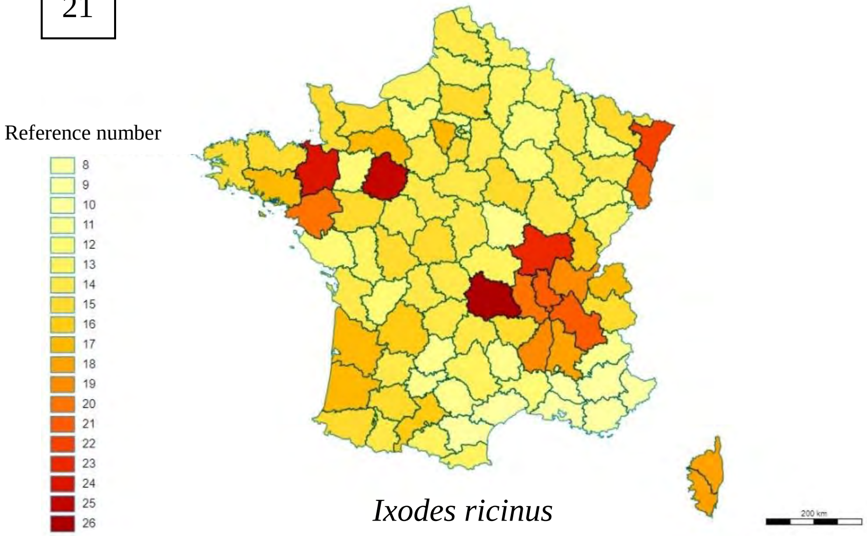
Reference number
8
9
10
11
12
13
14
15
16
17
18
19
20
21
22
23
24
25
26
Ixodes ricinus

22

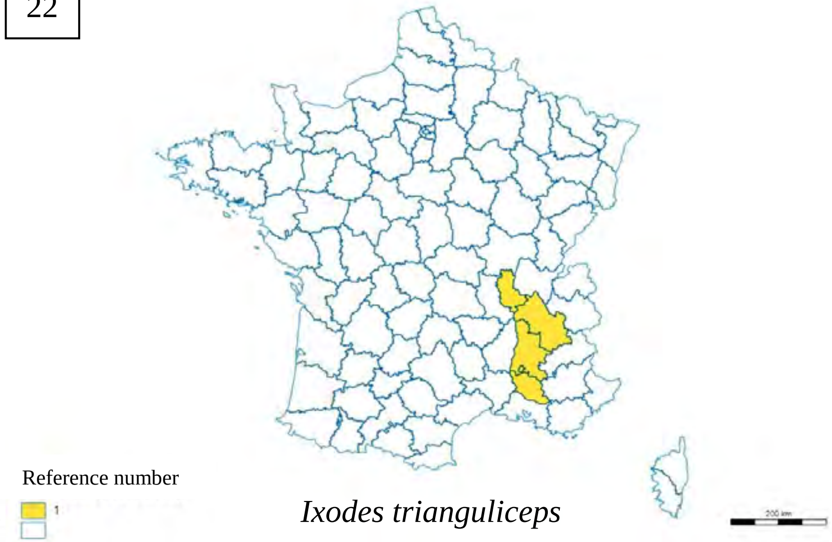
Reference number
1
Ixodes trianguliceps

23

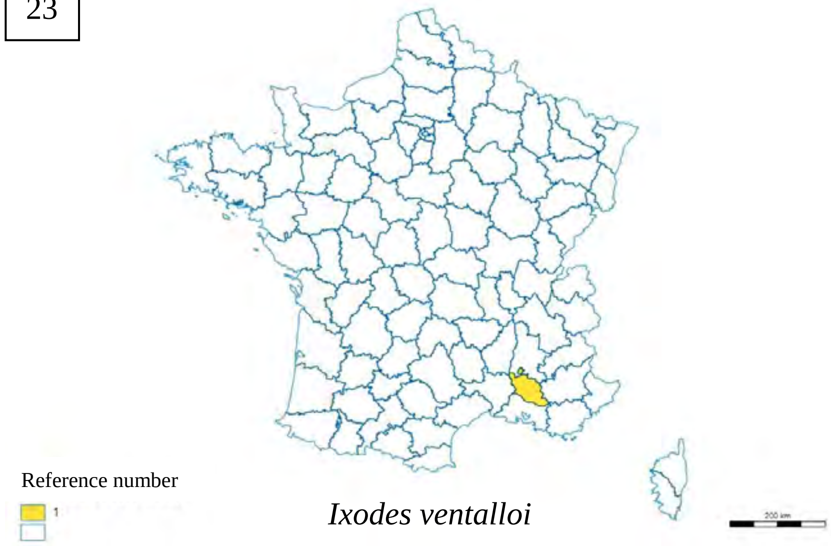
Reference number
1
Ixodes ventalloi

24

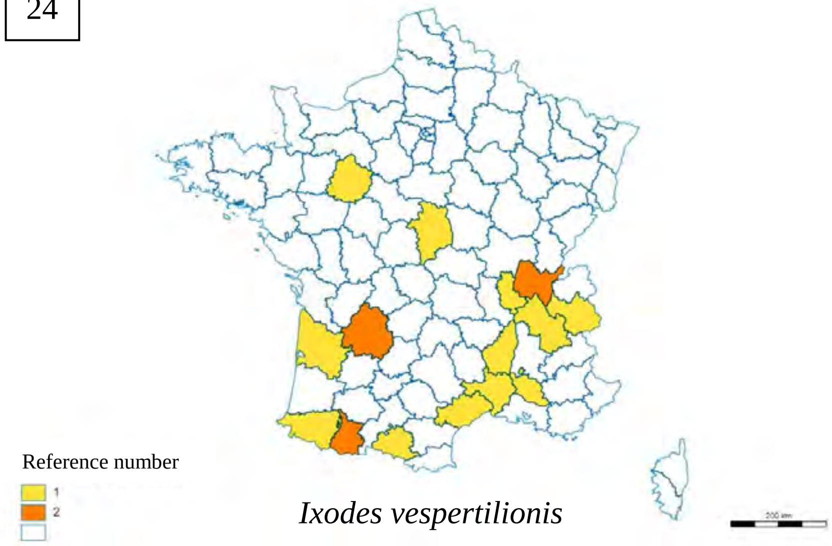
Reference number
1
2
Ixodes vespertilionis

25

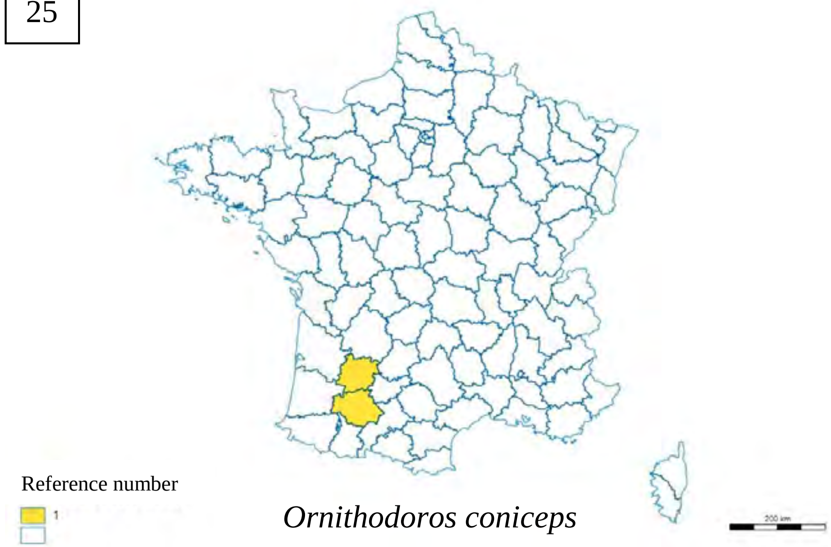

Ornithodoros coniceps

26

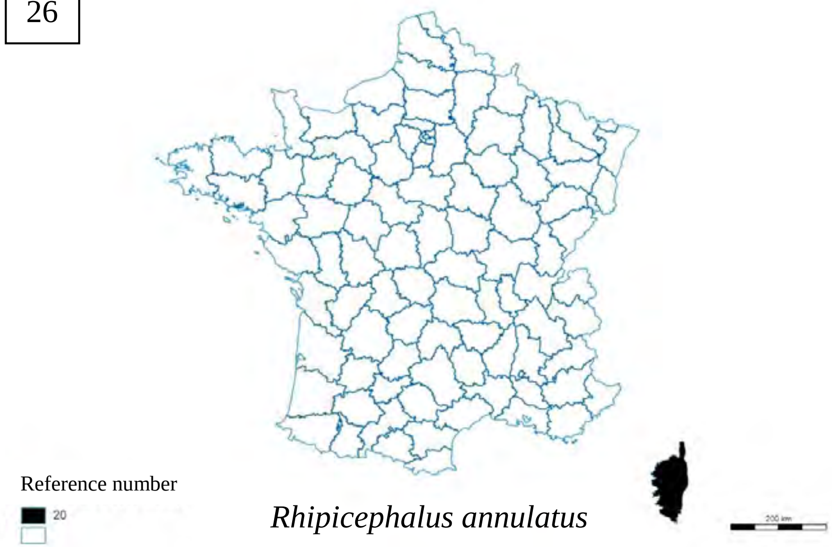

Rhipicephalus annulatus

27

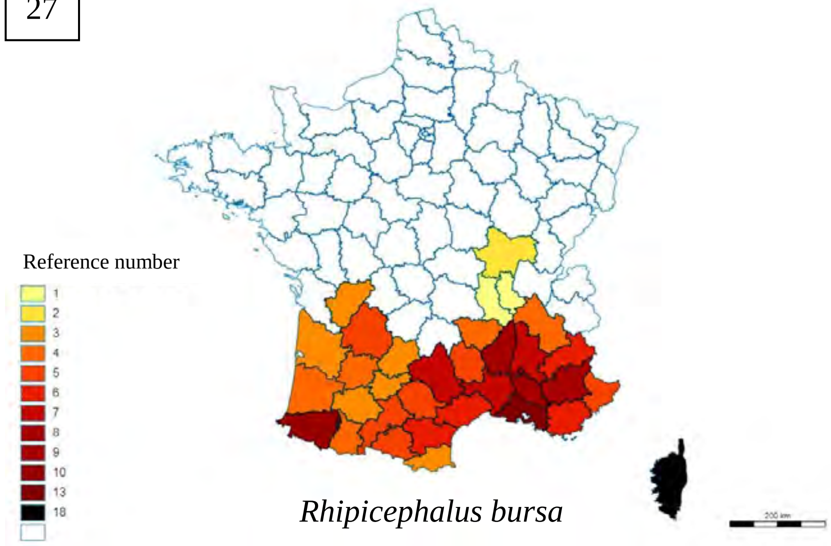

Rhipicephalus bursa

28

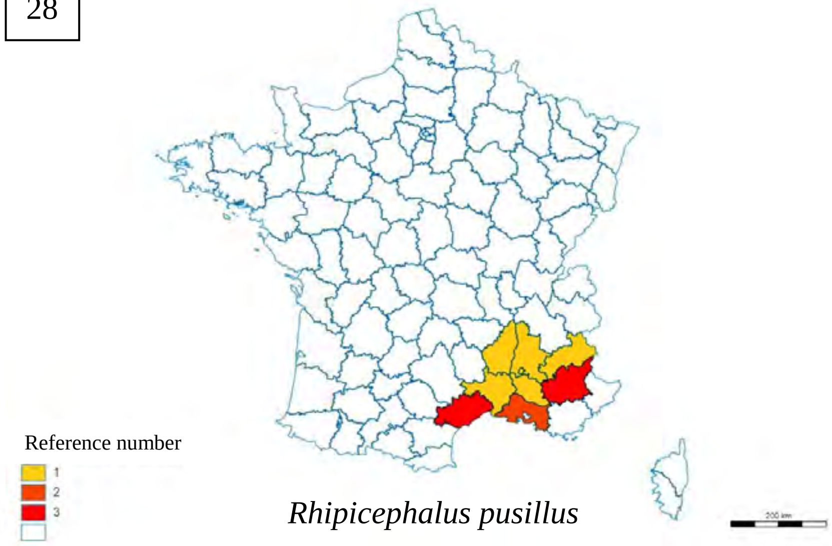

Rhipicephalus pusillus

29

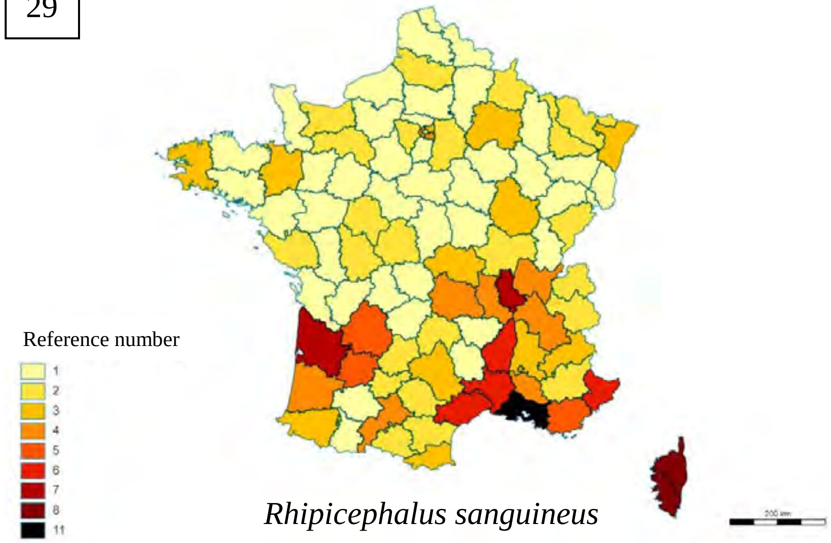

30

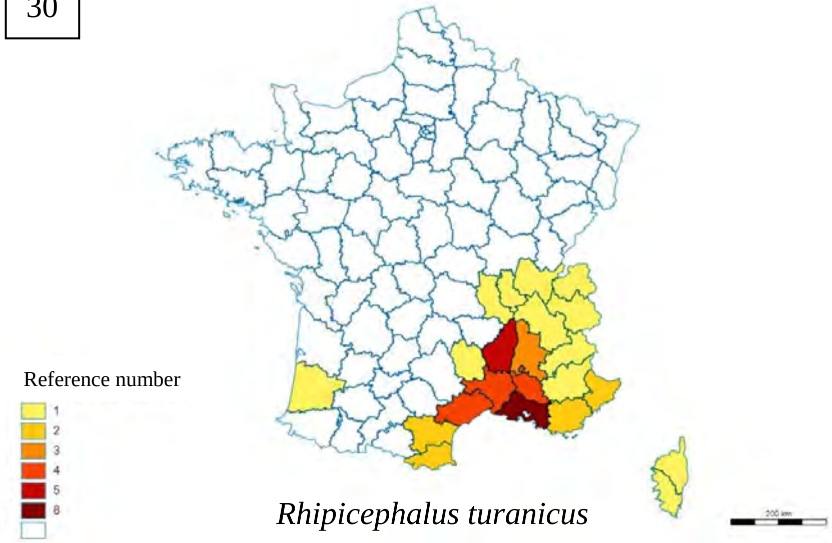

Supplementary Figure S10. Distribution map of *Amblyomma variegatum* (1), *Argas reflexus* (2), *Argas vespertilionis* (3), *Dermacentor marginatus* (4), *Dermacentor reticulatus* (5), *Haemaphysalis concinna* (6), *Haemaphysalis inermis* (7), *Haemaphysalis punctata* (8), *Haemaphysalis sulcata* (9), *Hyalomma aegyptium* (10), *Hyalomma detritum* (11), *Hyalomma excavatum* (12), *Hyalomma lusitanicum* (13), *Hyalomma marginatum* (14), *Hyalomma scupense* (15), *Ixodes acuminatus* (16), *Ixodes canisuga* (17), *Ixodes festai* (18), *Ixodes frontalis* (19), *Ixodes hexagonus* (20), *Ixodes ricinus* (21), *Ixodes trianguliceps* (22), *Ixodes ventalloi* (23), *Ixodes vespertilionis* (24), *Ornithodoros coniceps* (25), *Rhipicephalus annulatus* (26), *Rhipicephalus bursa* (27), *Rhipicephalus pusillus* (28), *Rhipicephalus sanguineus* (29) and *Rhipicephalus turanicus* (30) by department according to the number of references.
