# Supplementary material for: Systematic review of hematophagous arthropods present in cattle in France
Source: Parasite. 2023 Dec 12;30:56. doi: 10.1051/parasite/2023059 (PMC10714678; doi:10.1051/parasite/2023059)
Supplement: Supplementary file 1 — Supplementary Figure 1. Distribution map of the different hematophagous arthropods identified in the systematic review by department according to the number of references and, if available, the percentage of capture. Supplementary Table 1. Complete list of references included in the systematic review. Supplementary Table 2. Description of captures by species of the different hematophagous arthropods identified in the systematic review. [file parasite-30-56-s1.zip › Suppl_Tab_1.docx]

# References

- Abonnenc, E. (1972). Les phlébotomes de la région éthiopienne (Diptera, Psychodidae). Paris (France), Cahiers de l'ORSTOM, série Entomologie médicale et Parasitologie.

- Agoulon, A., L. Malandrin, F. Lepigeon, M. Vénisse, S. Bonnet, C. A. Becker, T. Hoch, S. Bastian, O. Plantard and F. Beaudeau (2012a). "A vegetation index qualifying pasture edges is related to *Ixodes ricinus* density and to *Babesia divergens* seroprevalence in dairy cattle herds." Veterinary Parasitology **185**(2-4): 101-109.

- Agoulon, A., O. Plantard and M. l'Hostis (2012b). "Tiques et maladies à tiques chez les bovins en France : effet des changements globaux ?" Le Point Vétérinaire: 118-123.

- Aitken, T. H. (1954). "The Culicidae of Sardinia and Corsica (Diptera)." Bulletin of Entomological Research **45**(3): 437-494.

- Alexander, N., A. Allepuz, B. Alten, R. Bødker, S. Bonnet, S. Carpenter, C. Cêtre-Sossah, E. Chirouze, J. Depaquit and K. Dressel (2015). The impact of a decade (2004-2015) of research on vector-borne diseases, CIRAD.

- Alten, B., C. Maia, M. O. Afonso, L. Campino, M. Jimenez, E. Gonzalez, R. Molina, A. L. Banuls, J. Prudhomme, B. Vergnes, C. Toty, C. Cassan, N. Rahola, M. Thierry, D. Sereno, G. Bongiorno, R. Bianchi, C. Khoury, N. Tsirigotakis, E. Dokianakis, M. Antoniou, V. Christodoulou, A. Mazeris, M. Karakus, Y. Ozbel, S. K. Arserim, O. Erisoz Kasap, F. Gunay, G. Oguz, S. Kaynas, N. Tsertsvadze, L. Tskhvaradze, E. Giorgobiani, M. Gramiccia, P. Volf and L. Gradoni (2016). "Seasonal dynamics of phlebotomine sand fly species proven vectors of Mediterranean leishmaniasis caused by *Leishmania infantum*." PLOS Neglected Tropical Diseases **10**(2): e0004458.

- Anderson, J., J. Doby, A. Coutarmanac'h, F. Hyde and R. Johnson (1986). "Différences antigéniques entre des souches de *Borrelia burgdorferi* isolées d'*Ixodes ricinus* en Bretagne." Médecine et Maladies Infectieuses **16**(3): 171-175.

- Arnold, P. and M. Kremer (1982). Preliminary study for a mosquito eradication operation in Northern Bas-Rhin (France); Possible effects on Ceratopogonodae. Proceedings of the Fifth International Symposium on Ceratopogonidae, Strasbourg.

- Augot, D., F. Sauvage, D. Jouet, E. Simphal, M. Veuille, A. Couloux, M. L. Kaltenbach and J. Depaquit (2010). "Discrimination of *Culicoides obsoletus* and *Culicoides scoticus*, potential bluetongue vectors, by morphometrical and mitochondrial cytochrome oxidase subunit I analysis." Infection, Genetics and Evolution **10**(5): 629-637.

- Baldacchino, F. (2013). Écologie des Tabanidae en zones pastorales méditerranéennes et perspectives de lutte. Ph.D, Université Paul-Valéry-Montpellier 3.

- Baldacchino, F., J. Cadier, A. Porciani, B. Buatois, L. Dormont and P. Jay‐Robert (2013a). "Behavioural and electrophysiological responses of females of two species of tabanid to volatiles in urine of different mammals." Medical and Veterinary Entomology **27**(1): 77-85.

- Baldacchino, F., L. Gardes, E. De Stordeur, P. Jay-Robert and C. Garros (2014a). "Blood-feeding patterns of horse flies in the French Pyrenees." Veterinary Parasitology **199**(3-4): 283-288.

- Baldacchino, F., S. Manon, L. Puech, B. Buatois, L. Dormont and P. Jay-Robert (2013b). "Olfactory and behavioural responses of tabanid horseflies to octenol, phenols and aged horse urine." Medical and Veterinary Entomology **28**(2): 201-209.

- Baldacchino, F., A. Porciani, C. Bernard and P. Jay-Robert (2014b). "Spatial and temporal distribution of Tabanidae in the Pyrenees Mountains: the influence of altitude and landscape structure." Bulletin of Entomological Research **104**(1): 1-11.

- Baldacchino, F., L. Puech, S. Manon, L. R. Hertzog and P. Jay-Robert (2014c). "Biting behaviour of Tabanidae on cattle in mountainous summer pastures, Pyrenees, France, and effects of weather variables." Bulletin of Entomological Research **104**(4): 471-479.

- Baldet, T., J. Delécolle, B. Mathieu, S. de La Rocque and F. Roger (2004). "Entomological surveillance of bluetongue in France in 2002." Veterinaria Italiana **40**(3): 226-231.

- Baldet, T., J. C. Delecolle, C. Cêtre-Sossah, B. Mathieu, R. Meiswinkel and G. Gerbier (2008). "Indoor activity of *Culicoides* associated with livestock in the bluetongue virus (BTV) affected region of northern France during autumn 2006." Preventive Veterinary Medicine **87**(1-2): 84-97.

- Baldet, T., B. Mathieu, J. Delecolle, G. Gerbier and F. Roger (2005). "Emergence de la fièvre catarrhale ovine dans le Bassin méditerranéen et surveillance entomologique en France." Revue d'Élevage et de Médecine Vétérinaire des Pays Tropicaux **58**(3): 125-132.

- Balenghien, T., J.-C. Delécolle, M.-L. Setier-Rio, I. Rakotaoarivony, X. Allène, R. Venail, D. Delécolle, J. Lhoir, L. Gardès and D. Chavernac (2010). "Bluetongue-report on entomological surveillance in France in 2010." Bulletin Epidémiologique, Santé Animale et Alimentation **46**: 26-31.

- Balenghien, T., J. C. Delecolle, M.-L. Setier-Rio, D. Delécolle, X. Allène, I. Rakotoarivony, B. Scheid, B. Mathieu, D. Chavernac and J.-B. Perrin (2013). "L'activité des populations de *Culicoides* en 2012 et bilan des quatre années du dispositif de surveillance." Bulletin Epidémiologique, Santé Animale et Alimentation **59**: 39-40.

- Balenghien, T., F. Fouque, P. Sabatier and D. J. Bicout (2006). "Horse-, bird-, and human-seeking behavior and seasonal abundance of mosquitoes in a West-Nile virus focus of Southern France." Journal of Medical Entomology **43**(5): 936-946.

- Balenghien, T., I. Rakotoarivony, X. Allène, J.-B. Perrin and C. Garros (2014a). "L’activité des populations de *Culicoides* en Corse en 2013." Bulletin Epidémiologique, Santé Animale et Alimentation **64**: 45-46.

- Balenghien, T., I. Rakotoarivony, X. Allène, J.-B. Perrin and C. Garros (2014b). "L’activité des populations de *Culicoides* en Corse en 2014." Bulletin Epidémiologique, Santé Animale et Alimentation **71**: 45-46.

- Beati, L., J. Finidori and D. Raoult (1993). "First isolation of *Rickettsia slovaca* from *Dermacentor marginatus* in France." American Journal of Tropical Medicine and Hygiene **48**(2): 257-268.

- Beaucornu, J.-C. (1976). "Notes sur les Ceratophyllidae (Siphonaptera) de la faune française, inféodés aux mammifères." Annales de la Société Entomologique de France **12**: 199-213.

- Beaucournu-Saguez, F. (1977). "Contribution à l’étude des simulies (Diptera, Nematocera) du complexe “*Aureum*” en France." Annales de Parasitologie Humaine et Comparée **52**(2): 181-194.

- Becker, C. A., A. Bouju-Albert, M. Jouglin, A. Chauvin and L. Malandrin (2009). "Natural transmission of zoonotic *Babesia* spp. by *Ixodes ricinus* ticks." Emerging Infectious Diseases **15**(2): 320-322.

- Beugnet, F., K. Chalvet-Monfray and H. Loukos (2009). "FleaTickRisk: a meteorological model developed to monitor and predict the activity and density of three tick species and the cat flea in Europe." Geospatial health **4**(1): 97-113.

- Beytout, J., J. George, J. Malaval, M. Garnier, M. Beytout, G. Baranton, E. Ferquel and D. Postic (2007). "Lyme borreliosis incidence in two French departments: correlation with infection of *Ixodes ricinus* ticks by *Borrelia burgdorferi* sensu lato." Vector-Borne and Zoonotic Diseases **7**(4): 507-518.

- Blanc-Debrune, N. (2019). Impact économique des principales espèces de diptères sur l'élevage bovin français et méthodes de luttes associées. Ph.D, Ecole Nationale Vétérinaire de Lyon.

- Boiteux, P. and C. Noirtin (1979). "[Death of 25 farm animals by blackfly [*Simulium*] stings in Vosges (France)]." Bulletin de l'Académie et de la Société Lorraines des Sciences: 75-80.

- Bonnet, S., J. De La Fuente, P. Nicollet, X. Liu, N. Madani, B. Blanchard, C. Maingourd, A. Alongi, A. Torina and I. Fernández de Mera (2013). "Prevalence of tick-borne pathogens in adult *Dermacentor* spp. ticks from nine collection sites in France." Vector-Borne and Zoonotic Diseases **13**(4): 226-236.

- Bonnet, S., L. Michelet, S. Moutailler, J. Cheval, C. Hébert, M. Vayssier-Taussat and M. Eloit (2014). "Identification of parasitic communities within European ticks using next-generation sequencing." PLOS Neglected Tropical Diseases **8**(3): e2753.

- Bouchet, F., F. Lavaud, I. Girardin and C. Prévoteau (1993). "Contribution à l'étude des simulies dans la Région champenoise (Diptera, Simuliidae)." Bulletin de la Société Entomologique de France **98**(5): 425-434.

- Bourdeau, P. (1993). "Les tiques d'importance vétérinaire et médicale : 2e partie : Principales espèces de tiques dures (Ixodidae et Amblyommidae)." Le Point Vétérinaire **25**(151): 27-41.

- Boyard, C. (2007). Facteurs environnementaux de variation de l'abondance des tiques *Ixodes ricinus* dans des zones d'étude modèles en Auvergne, Université Blaise Pascal-Clermont-Ferrand II.

- Boyard, C., J. Barnouin, S. Bord, P. Gasqui and G. Vourc’h (2011). "Reproducibility of local environmental factors for the abundance of questing *Ixodes ricinus* nymphs on pastures." Ticks and Tick-borne Diseases **2**(2): 104-110.

- Boyard, C., J. Barnouin, P. Gasqui and G. Vourc'h (2007a). "Local environmental factors characterizing *Ixodes ricinus* nymph abundance in grazed permanent pastures for cattle." Parasitology **134**(7): 987-994.

- Boyard, C., P. Gasqui, J. Barnouin and V. H. Gwenaël (2007b). "Comment diminuer le risque de maladies transmises par les tiques chez les bovins au paturage ?" Bulletin des GTV(41): 67-72.

- Boyard, C., G. Vourc’h and J. Barnouin (2008). "The relationships between *Ixodes ricinus* and small mammal species at the woodland–pasture interface." Experimental and Applied Acarology **44**(1): 61-76.

- Brumpt, E. (1925). "Les anophèles de Corse." Bulletin de l'Académie Nationale de Médecine **94**: 811-815.

- Brumpt, E. (1942). "Notes parasitologiques concernant l’aménagement agricole de la Crau." Annales de Parasitologie Humaine et Comparée **19**(1-2-3): 74-84.

- Brumpt, E. (1944a). "Anophélisme sans paludisme et régression spontanée du paludisme." Annales de Parasitologie **XX**(1-2): 67-91.

- Brumpt, E. (1944b). "Zooprophylaxie du paludisme." Annales de Parasitologie **XX**(3-4): 191-206.

- Brutus, L., J. Guilloteau, D. Gauvrit, J. Mas and M. Marjolet (1993). "Les aires culicidogènes des marais littoraux du Morbihan : éléments cartographiques." Bulletin de la Société Française de Parasitologie **11**(2): 237-244.

- Bussieras, J. and X. l. Pannerer (1980). "Tests for the control of cattle flies by means of a synthetic pyrethroid." Recueil de Médecine Vétérinaire **156**(5): 283-286.

- Callot, J. and V. T. Dao (1944). "Contribution à l’étude des moustiques français Culicidés de Richelieu (Indre-et-Loire)." Annales de Parasitologie Humaine et Comparée **20**(1-2): 43-66.

- Carnevale, P. and V. Robert (2009). Les anophèles. Biologie, transmission du *Plasmodium* et lutte antivectorielle. Marseille (France), IRD Editions.

- Carrer, H. (2007). Évaluation de la prévalence d'infection des tiques *Ixodes ricinus* par des protozoaires babésiidés en élevage bovin. Ph.D, Faculté de Medecine de Nantes.

- Cetre-Sossah, C. (2010). Des parasites humains aux virus animaux, une histoire de vecteurs. HDR, Université de Montpellier 2.

- Cêtre-Sossah, C., T. Baldet, J.-C. Delécolle, B. Mathieu, A. Perrin, C. Grillet and E. Albina (2004). "Molecular detection of *Culicoides* spp. and *Culicoides imicola*, the principal vector of bluetongue (BT) and African horse sickness (AHS) in Africa and Europe." Veterinary Research **35**(3): 325-337.

- Chacker, E. (1982). Description of larvae of six species of *Culicoides*. Proceedings of the Fifth International Symposium on Ceratopogonidae, Strasbourg.

- Charrel, R. N., G. Moureau, S. Temmam, A. Izri, P. Marty, P. Parola, A. T. da Rosa, R. B. Tesh and X. de Lamballerie (2009). "Massilia virus, a novel *Phlebovirus* (*Bunyaviridae*) isolated from sandflies in the Mediterranean." Vector-Borne and Zoonotic Diseases **9**(5): 519-530.

- Chaskopoulou, A., G. L'Ambert, D. Petric, R. Bellini, M. Zgomba, T. A. Groen, L. Marrama and D. J. Bicout (2016). "Ecology of West Nile virus across four European countries: review of weather profiles, vector population dynamics and vector control response." Parasites and Vectors **9**(1): s13071.

- Chastagner, A., A. Pion, H. Verheyden, B. Lourtet, B. Cargnelutti, D. Picot, V. Poux, É. Bard, O. Plantard and K. D. McCoy (2017). "Host specificity, pathogen exposure, and superinfections impact the distribution of *Anaplasma phagocytophilum* genotypes in ticks, roe deer, and livestock in a fragmented agricultural landscape." Infection, Genetics and Evolution **55**: 31-44.

- Chastel, C., B. Devau, F. Le Goff, A. Simitzis-Le Flohic, R. Gruffaz, G. Kerdraon and B. Gilot (1987). "Mosquito spiroplasmas from France and their ecology." Israel Journal of Medical Sciences **23**(6): 683-686.

- Chastel, C., B. Gilot, F. Le Goff, R. Gruffaz and M.-L. Abalain-Colloc (1985). "Isolement de spiroplasmes en France (Savoie, Alpes du Nord) à partir de moustiques du genre *Aedes*." Comptes Rendus de l'Académie des Sciences de Paris Série 3 **300**(7): 261-266.

- Chauvet, S. (2004). Étude dynamique des populations de tiques dans des élevages bovins en Corrèze. DVM thesis, École Nationale Vétérinaire de Nantes.

- Chauvet, S. and M. L'Hostis (2005). "Bovine ticks: biology, distribution and their role as vectors." Le Point Vétérinaire **255**: 1-7.

- Cicculli, V., N. Ayhan, L. Luciani, L. Pezzi, A. Maitre, D. Decarreaux, X. De Lamballerie, J. c. Paoli, L. Vial and R. Charrel (2022). "Molecular detection of parapoxvirus in Ixodidae ticks collected from cattle in Corsica, France." Veterinary Medicine and Science **8**(2): 907-916.

- Cicculli, V., L. Capai, Y. Quilichini, S. Masse, A. Fernández-Alvarez, L. Minodier, P. Bompard, R. Charrel and A. Falchi (2019a). "Molecular investigation of tick-borne pathogens in ixodid ticks infesting domestic animals (cattle and sheep) and small rodents (black rats) of Corsica, France." Ticks and Tick-borne Diseases **10**(3): 606-613.

- Cicculli, V., X. de Lamballerie, R. Charrel and A. Falchi (2019b). "First molecular detection of *Rickettsia africae* in a tropical bont tick, *Amblyomma variegatum*, collected in Corsica, France." Experimental and Applied Acarology **77**: 207-214.

- Cicculli, V., D. DeCarreaux, N. Ayhan, F. Casabianca, X. De Lamballerie, R. Charrel and A. Falchi (2020). "Molecular screening of Anaplasmataceae in ticks collected from cattle in Corsica, France." Experimental and Applied Acarology **81**: 561-574.

- Cicculli, V., O. Maestrini, F. Casabianca, N. Villechenaud, R. Charrel, X. De Lamballerie and A. Falchi (2019c). "Molecular detection of spotted-fever group rickettsiae in ticks collected from domestic and wild animals in Corsica, France." Pathogens **8**(138): 8030138.

- Cicculli, V., S. Masse, L. Capai, X. de Lamballerie, R. Charrel and A. Falchi (2019d). "First detection of *Ehrlichia minasensis* in *Hyalomma marginatum* ticks collected from cattle in Corsica, France." Veterinary Medicine and Science **5**(2): 243-248.

- Cosson, J.-F., L. Michelet, J. Chotte, E. Le Naour, M. Cote, E. Devillers, M.-L. Poulle, D. Huet, M. Galan and J. Geller (2014). "Genetic characterization of the human relapsing fever spirochete *Borrelia miyamotoi* in vectors and animal reservoirs of Lyme disease spirochetes in France." Parasites and Vectors **7**(1): 1-5.

- Cotté, V., S. Bonnet, M. Cote and M. Vayssier-Taussat (2010). "Prevalence of five pathogenic agents in questing *Ixodes ricinus* ticks from western France." Vector-Borne and Zoonotic Diseases **10**(8): 723-730.

- Cotteaux-Lautard, C., I. Leparc-Goffart, J. M. Berenger, S. Plumet and F. Pages (2016). "Phenology and host preferences *Phlebotomus perniciosus* (Diptera: Phlebotominae) in a focus of Toscana virus (TOSV) in South of France." Acta Tropica **153**: 64-69.

- Courouble, F., T. Geurden and D. Bartram (2012). "[Assessment of the efficacy of Cydectine Pour-on® in the fight against phthiriasis in cattle]." Bulletin des GTV(67): 79-86.

- Cuéllar, A. C., L. J. Kjær, C. Kirkeby, H. Skovgard, S. A. Nielsen, A. Stockmarr, G. Andersson, A. Lindstrom, J. Chirico and R. Lühken (2018). "Spatial and temporal variation in the abundance of *Culicoides* biting midges (Diptera: Ceratopogonidae) in nine European countries." Parasites and Vectors **11**(1): 1-18.

- Cuillé, M. J., P. L. Chelle and F. Berlureau (1936). "L'anaplasmose bovine en France." Révue Générale de Médecine Vétérinaire **529**: 1-13.

- Dahmani, M., B. Davoust, D. Tahir, D. Raoult, F. Fenollar and O. Mediannikov (2017). "Molecular investigation and phylogeny of Anaplasmataceae species infecting domestic animals and ticks in Corsica, France." Parasites and Vectors **10**(1): 1-12.

- Dedet, J.-P., B. Carme, N. Desbois, G. Bourdoiseau, L. Lachaud and F. Pratlong (2013). "Épidémiologie des leishmanioses autochtones en France métropolitaine et d’outre-mer." La Presse Médicale **42**(11): 1469-1481.

- Delécolle, J.-C. and S. d. La Rocque (2002). "Contribution à l'étude des *Culicoides* de Corse. Liste des espèces recensées en 2000/2001 et redescription du principal vecteur de la Fièvre Catarrhale Ovine : *Culicoides imicola* Kieffer, 1913 (Diptera, Ceratopogonidae)." Bulletin de la Société Entomologique de France **107**(4): 371-379.

- Devos, J. (2002). Étude épidémiologique de *Babesia divergens* dans une clientèle vétérinaire du centre-est de la France. M.Sc., Institut de Médecine Tropicale Prince Léopold.

- Devos, J., C. Jakobczyk and L. Zenner (2018). "[Phthiriasis in dairy cattle: epidemiological survey in the Monts du Lyonnais area]." Bulletin des GTV(91): 71-78.

- Doby, J., J. Anderson and A. Couatarmanac'h (1985). "Observation de spirochètes chez *Ixodes ricinus* en Bretagne. Note préliminaire." Médecine et Maladies Infectieuses **15**(10): 556-557.

- Doby, J., S. Chevrier, A. Couatarmanac'h and C. Imbert-Hameurt (1989). "Infection de *Ixodes ricinus* (Acarina, ixodidae) par *Borrelia burgdorferi*, agent des spirochetoses à tique (maladie de Lyme et autres formes cliniques) dans l'ouest de la France. II : Résultats détaillés et commentaires." Bulletin de la Société Française de Parasitologie **7**(2): 277-287.

- Dorffer, M. (1998). "[Lice, warble flies and liver flukes: means of success]." PLM : Production Laitière Moderne: 58-62.

- Dugat, T., A. Leblond, N. Keck, A.-C. Lagrée, I. Desjardins, A. Joulié, S. Pradier, B. Durand, H.-J. Boulouis and N. Haddad (2017). "One particular *Anaplasma phagocytophilum* ecotype infects cattle in the Camargue, France." Parasites and Vectors **10**(1): 1-6.

- ECDC. (2022a). "European Centre for Disease Prevention and Control. Biting midge maps." Access date 10/01/2023, Available from <https://www.ecdc.europa.eu/en/disease-vectors/surveillance-and-disease-data/biting-midge-maps>.

- ECDC. (2022b). "European Centre for Disease Prevention and Control. Mosquito maps." Access date 10/01/2023, Available from <https://www.ecdc.europa.eu/en/disease-vectors/surveillance-and-disease-data/mosquito-maps>.

- ECDC. (2022c). "European Centre for Disease Prevention and Control. Phlebotomine sandfly maps." Access date 10/01/2023, Available from <https://www.ecdc.europa.eu/en/disease-vectors/surveillance-and-disease-data/phlebotomine-maps>.

- ECDC. (2022d). "European Centre for Disease Prevention and Control. Ticks maps." Access date 10/01/2023, Available from <https://www.ecdc.europa.eu/en/disease-vectors/surveillance-and-disease-data/tick-maps>.

- EFSA (2010). "Panel on Animal Health Welfare. Scientific opinion on the role of tick vectors in the epidemiology of Crimean‐Congo Hemorrhagic Fever and African Swine Fever in Eurasia." EFSA Journal **8**(8): 1703-1859.

- Eiden, M., P. Gil, U. Ziegler, I. Rakotoarivony, A. Marie, B. Frances, G. l'Ambert, Y. Simonin, V. Foulongne and M. H. Groschup (2018). "Emergence of two Usutu virus lineages in *Culex pipiens* mosquitoes in the Camargue, France, 2015." Infection, Genetics and Evolution **61**: 151-154.

- Euzéby, J. and P. Rancien (1966). "Sur une endémie de babesiellose bovine." Bulletin de la Société Scientifique Vétérinaire et Médicale Comparée de Lyon **68**: 309-324.

- Euzeby, J. and P. H. Simon (1984). Épidémiologie de la babésiose bovine a *Babesia divergens* en France. Cinétique comparée des populations d'*Ixodes ricinus* et de fréquence de la babésiose. Conséquences pour la prophylaxie de la babésiose. Chimioprévention par un dérivé de la carbanilide, le 4 A 65 ou H.R. 2073 (Imidocarb). Agriculture. Some important parasitic infections in bovines considered from economic and social (zoonosis) points of view É. n. v. d. Lyon.

- Falcoz, L. (1926). Faune de France. 14. Diptères pupipares. Paris (France), Lechevalier P.

- Ferquel, E., M. Garnier, J. Marie, C. Bernede-Bauduin, G. Baranton, C. Pérez-Eid and D. Postic (2006). "Prevalence of *Borrelia burgdorferi* sensu lato and *Anaplasmataceae* members in *Ixodes ricinus* ticks in Alsace, a focus of Lyme borreliosis endemicity in France." Applied and Environmental Microbiology **72**(4): 3074-3078.

- Franc, M. (1986). "Essais de lutte contre les mouches du bétail par un spray au fenvalerate." Revue de Médecine Vétérinaire **137**(6): 403-406.

- Franc, M. and M. C. Cadiergues (1994). "Investigations on the control of flies in grazing-cattle - a field trial with deltamethrin as pour-on." Revue de Médecine Vétérinaire **145**(5): 337-342.

- François, J.-B. (2008). Les tiques chez les bovins en France, UHP-Université Henri Poincaré.

- Frédéric, E. (2005). Babésiose bovine à *babesia divergens* : étude d'un cas d'émergence en Corrèze, Faculté de Médecine de Nantes.

- Garros, C. (2022). Complete data of *Culicoides* captures realized by the surveillance network in France in 2010.

- Garros, C., L. Gardes, X. Allene, I. Rakotoarivony, E. Viennet, S. Rossi and T. Balenghien (2011). "Adaptation of a species-specific multiplex PCR assay for the identification of blood meal source in *Culicoides* (Ceratopogonidae: Diptera): applications on Palaearctic biting midge species, vectors of Orbiviruses." Infection, Genetics and Evolution **11**(5): 1103-1110.

- Gauchard, F. and A.-M. Hattenberger (2005). Rapport sur l'évaluation du risque d'apparition et de développement de maladies animales compte tenu d'un éventuel réchauffement climatique. Paris (France), AFSSA.

- GBIF. (2023). "Global Biodiversity Information Facility." Access date 20/02/2023, Available from <https://www.gbif.org/fr/>.

- George, J. and C. Chastel (2002). "Epidemiology-Tick-borne diseases and ecosystem changes in Lorraine." Bulletin de la Société de Pathologie Exotique **95**(2): 95-99.

- Gerbier, G., F. Biteau‐Coroller, C. Grillet, J. Parodi, S. Zientara, T. Baldet, H. Guis and F. Roger (2008). "Description of the outbreak of Bluetongue in Corsica in 2003, and lessons for surveillance." Veterinary Record **162**(6): 173-176.

- Gerbier, G., J. Parodi, F. Biteau-Coroller, T. Baldet, B. Mathieu, S. Zientara, C. Cêtre-Sossah and F. Roger (2006). "Surveillance de la Fiévre Catarrhale Ovine (Bluetongue) en France et dans l'ouest méditerranéen : Bilan et perspectives." Epidémiologie et Santé Animale **49**: 37-44.

- Gilot, B. (1985). "Répartition et écologie d'*Haemaphysalis punctata* (Canestrini et Fanzago, 1877) (Acarina, Ixodoidea) dans les Alpes françaises et leur avant-pays." Revista Iberica de Parasitología **45**(1): 25-40.

- Gilot, B. (1987). Note préliminaire sur les tiques (Acarina, Ixodidae) du Mont Ventoux. Études vauclusiennes. **no spécial 3:** 153-155. .

- Gilot, B., G. Ain, G. Pautou and R. Gruffaz (1976). "Les Culicidés de la Région Rhône-Alpes : bilan de dix années d'observation." Bulletin de la Société Entomologique de France **81**(7): 235-245.

- Gilot, B., J. Gillet, M. Quilici, S. Dunan, B. Lachet and J. Ranque (1983). "Le foyer Marseillais de leishmaniose viscérale : cartographie épidémiologique à 1/50 000. Essai méthodologique." Documents de Cartographie Ecologique **26**: 3-27.

- Gilot, B. and G. Pautou (1981). "Répartition et intérêt épidémiologique de *Rhipicephalus turanicus* (Pomerantsev, Matikasvili, Lototzki, 1940) (Acarina, Ixodoidea) écologie de cette espèce dans le midi méditerranéen français." Annales de Parasitologie **56**(1): 547-558.

- Gilot, B. and G. Pautou (1982). "L'évolution des populations de tiques (Ixodidae et Argasidae) en relation avec l'artificialisation des milieux dans les Alpes françaises." Acta Tropica **39**: 337-354.

- Gilot, B., G. Pautou and E. Moncada (1975a). "Première contribution à l'étude écologique d'*Ixodes ricinus* (Linne, 1758) (Acarina, Ixodoidea) dans le sud-est de la France." Acta Tropica **XXXII**(3): 232-258.

- Gilot, B., G. Pautou, E. Moncada, B. Lachet and J. Christin (1979). "La cartographie des populations de tiques exophiles par le biais de la végétation : bases écologiques, intérêt épidémiologique." Documents de Cartographie Ecologique **22**: 65-80.

- Gilot, B. and C. Pérez-Eid (1998). "Bio-écologie des tiques induisant les pathologies les plus importantes en France." Médecine et Maladies Infectieuses **28**(4): 325-334.

- Gilot, B., J. Pichot and B. Doche (1989). "The ticks of the Massif Central (France). I. The ixodides (Acariens, Ixodoidea) infesting domestic Carnivores and Ungulata in the Eastern border of the massif." Acarologia **30**(3): 191-207.

- Gilot, B., Y. Robin, G. Pantou, E. Moncada and F. Vigny (1975b). "Ecology and pathogenic role of *Dermacentor reticulatus* (Fabricius, 1794) (Ixodoidea) in South-Eastern France." Acarologia **16**(2): 220-249.

- Giroud, P., M. Capponi, N. Dumas and J. M. Rageau (1965). "Résultats concernant *Dermacentor marginatus* et *reticulatus* prélevés dans différentes régions de France et leur contamination avec des rickettsies ou des éléments proches." Comptes Rendus de l'Académie des Sciences de Paris **260**: 5419-5421.

- Grech-Angelini, S. (2017). "Ticks affecting cattle and other animals in Corsica." Le Point Vétérinaire **48**(379 (Part 2)): 44-50.

- Grech-Angelini, S., F. Stachurski, R. Lancelot, J. Boissier, J.-F. Allienne, M. Gharbi and G. Uilenberg (2016a). "First report of the tick *Hyalomma scupense* (natural vector of bovine tropical theileriosis) on the french Mediterranean island of Corsica." Veterinary Parasitology **216**: 33-37.

- Grech-Angelini, S., F. Stachurski, R. Lancelot, J. Boissier, J.-F. Allienne, S. Marco, O. Maestrini and G. Uilenberg (2016b). "Ticks (Acari: Ixodidae) infesting cattle and some other domestic and wild hosts on the french Mediterranean island of Corsica." Parasites and Vectors **9**(582): s13071.

- Grech‐Angelini, S., F. Stachurski, M. Vayssier‐Taussat, E. Devillers, F. Casabianca, R. Lancelot, G. Uilenberg and S. Moutailler (2020). "Tick‐borne pathogens in ticks (Acari: Ixodidae) collected from various domestic and wild hosts in Corsica (France), a Mediterranean island environment." Transboundary and Emerging Diseases **67**(2): 745-757.

- Grenier, P. (1949). "Contribution à l'étude biologique des Simuliides de France." Physiologia Comparata et Oecologia **1**: 165-330.

- Grenier, P. and H. Bertrand (1951). "Récoltes de Diptères Simuliims et Blépharocêrides dans les Pyrênées." Vie et Milieu: 488-500.

- Grimeau, V. (2012). Étude de populations de diptères potentiellement vecteurs de *Besnoitia Besnoitii* en pays de la Loire. Ph.D, École Nationale Vétérinaire, Agroalimentaire et de l'Alimentation Nantes Atlantique.

- Guilloteau, J. (1990). "Culicidés du littoral Atlantique. Méthodes actuelles de lutte-gestion des milieux humides." Bulletin de la Société des Sciences Naturelles de l'Ouest de la France **12**(2): 72-78.

- Guy, M. W., R. Killick-Kendrick, G. S. Gill, J. A. Rioux and R. S. Bray (1984). "Ecology of leishmaniasis in the south of France. 19. Determination of the hosts of *Phlebotomus ariasi* Tonnoir, 1921 in the Cevennes by bloodmeal analyses." Annales de Parasitologie Humaine et Comparée **59**(5): 449-458.

- Hajd Henni, L., F. Sauvage, C. Ninio, J. Depaquit and D. Augot (2014). "Wing geometry as a tool for discrimination of Obsoletus group (Diptera: Ceratopogonidae: *Culicoides*) in France." Infection, Genetics and Evolution **21**: 110-117.

- Halos, L., S. Bord, V. Cotté, P. Gasqui, D. Abrial, J. Barnouin, H.-J. Boulouis, M. Vayssier-Taussat and G. Vourc'h (2010). "Ecological factors characterizing the prevalence of bacterial tick-borne pathogens in *Ixodes ricinus* ticks in pastures and woodlands." Applied and Environmental Microbiology **76**(13): 4413-4420.

- Halos, L., G. Vourc'h, V. Cotté, P. Gasqui, J. Barnouin, H. J. Boulous and M. Vayssier‐Taussat (2006). "Prevalence of *Anaplasma phagocytophilum*, *Rickettsia* sp. and *Borrelia burgdorferi* sensu lato DNA in questing *Ixodes ricinus* ticks from France." Annals of the New York Academy of Sciences **1078**(1): 316-319.

- Hannoun, C., R. Panthier, J. Mouchet and J. Eouzan (1964). "Isolement en France du virus West-Nile à partir de malades et du vecteur *Culex modestus* Ficalbi." Comptes Rendus Hebdomadaires Des Seances De L'Académie Des Sciences **259**(22): 4170-4172.

- Harant, H., M. Attisso and J.-A. Rioux (1955). "Sur l’écologie des diptères Culicidés. 3. Les espèces limno-dendrophiles et leur biotope. ." Comptes Rendus de la Société de Biologie **149**(15-8): 1626-1631.

- Hélias, C., M. Vazeille-Falcoz, F. Le Goff, M.-L. Abalain-Colloc, F. Rodhain, P. Carle, R. F. Whitcomb, D. L. Williamson, J. G. Tully and J. M. Bové (1998). "*Spiroplasma turonicum* sp. nov. from *Haematopota* horse flies (Diptera: Tabanidae) in France." International Journal of Systematic and Evolutionary Microbiology **48**(2): 457-461.

- Hendrikx, P. (2003). "Adaptation des réseaux de surveillance épidémiologique aux conditions de l'émergence." Epidémiologie et Santé Animale **44**: 51-59.

- INPN. (2023). "Inventaire National du Patrimoine Naturel. Synthèse de données pour les espèces." Access date 20/02/2023, Available from <https://inpn.mnhn.fr/espece/indicateur/FR/ES/7/CL/PH/Arthropoda>.

- Izri, M., P. Marty, P. Fauran, Y. Le Fichoux and J.-J. Rousset (1994). "*Phlebotomus perfiliewi* Parrot, 1930 (Diptera, Psychodidae) dans le sud-est de la France." Parasite **1**(3): 286-286.

- Jacquet, S., C. Garros, E. Lombaert, C. Walton, J. Restrepo, X. Allene, T. Baldet, C. Cetre‐Sossah, A. Chaskopoulou and J. C. Delecolle (2015). "Colonization of the Mediterranean basin by the vector biting midge species *Culicoides imicola*: an old story." Molecular Ecology **24**(22): 5707-5725.

- Jacquet, S., K. Huber, H. Guis, M.-L. Setier-Rio, M. Goffredo, X. Allène, I. Rakotoarivony, C. Chevillon, J. Bouyer and T. Baldet (2016a). "Spatio-temporal genetic variation of the biting midge vector species *Culicoides imicola* (Ceratopogonidae) Kieffer in France." Parasites and Vectors **9**(1): 1-12.

- Jacquet, S., K. Huber, S. Talavera, L. E. Burgin, S. Carpenter, C. Sanders, A. H. Dicko, M. Djerbal, M. Goffredo and Y. Lhor (2016b). "Range expansion of the Bluetongue vector, *Culicoides imicola*, in continental France likely due to rare wind-transport events." Scientific Reports **6**(1): 1-14.

- Jacquiet, P., D. Rouet, E. Bouhsira, A. Salem, E. Lienard and M. Franc (2014). "Population dynamics of *Stomoxys calcitrans* (L.) (Diptera: Muscidae) in southwestern France." Revue de Médecine Vétérinaire **165**(9/10): 267-271.

- Jacquiet, P., S. Shukri, E. Liénard, M. Franc and É. Bouhsira (2015). "Stable flies and horseflies: biology, pathogenic roles and control in cattle." Le Nouveau Praticien Vétérinaire Élevages et Santé(32): 13-20.

- Jetten, T. H. and W. Takken (1994). Anophelism without malaria in Europe: a review of the ecology and distribution of the genus *Anopheles* in Europe. Wageningen (Pays-Bas), Wageningen Agricultural University.

- Joncour, G., C. Brard, B. Courtay and J.-F. Labbe (2006). Dairy cows as bio-indicators of *Anasplasma phagocytophilum* agent of tick-borne fever In France. XIVth World Buiatrics Congress Proceedings, Nice.

- Jouglin, M., G. Perez, A. Butet, L. Malandrin and S. Bastian (2017). "Low prevalence of zoonotic *Babesia* in small mammals and *Ixodes ricinus* in Brittany, France." Veterinary Parasitology **238**: 58-60.

- Kempf, F., T. De Meeûs, C. Arnathau, B. Degeilh and K. D. Mccoy (2009). "Assortative pairing in *Ixodes ricinus* (Acari: Ixodidae), the European vector of lyme borreliosis." Journal of Medical Entomology **46**(3): 471-474.

- Kempf, F., K. D. McCoy and T. De Meeûs (2010). "Wahlund effects and sex-biased dispersal in *Ixodes ricinus*, the European vector of lyme borreliosis: new tools for old data." Infection, Genetics and Evolution **10**(7): 989-997.

- Kieffer, J.-J. (1925). Diptères (Nématocères piqueurs) : Chironomidae Ceratopogoninae. Paris (France), Lechevalier P.

- Killick-Kendrick, R., J.-A. Rioux, M. Ratify, M. Guy, T. Wilkes, F. Guy, I. Davidson, R. Knechtli, R. Ward and E. Guilvard (1984). "Ecology of leishmaniasis in the south of France. 20. Dispersal of *Phlebotomus ariasi* Tonnoir, 1921 as a factor in the spread of visceral leishmaniasis in the Cévennes." Annales de Parasitologie Humaine et Comparée **59**(6): 555-572.

- Kirk, A. A. (1992). "The effect of the dung pad fauna on the emergence of *Musca tempestiva* [Dipt.: Muscidae] from dung pads in southern France." Entomophaga **37**: 507-514.

- Kluiters, G., S. Carpenter, L. Gardes, H. Guis, M. Baylis and C. Garros (2016). "Morphometric discrimination of two sympatric sibling species in the Palaearctic region, *Culicoides obsoletus* Meigen and *C. scoticus* Downes & Kettle (Diptera: Ceratopogonidae), vectors of Bluetongue and Schmallenberg viruses." Parasites and Vectors **9**(1): 1-15.

- Kremer, M. (1965). Contribution à l'étude du genre *Culicoides* Latreille, particulièrement en France. Paris (France), Lechevalier P.

- Kremer, M., G. Leberre and F. Beaucournu-Saguez (1971). "Notes sur les *Culicoides* (Dipt. Ceratopogonidae) de Corse. Description de *C. corsicus* n. sp." Annales de Parasitologie Humaine et Comparée **46**(5): 653-660.

- Kremer, M., J. Rieb and C. Rebholtz (1978). "[Ecology of the Ceratopogonids of the Alsace plain. I. The genus *Culicoides* from the humid soils of the Ried]." Annales de Parasitologie Humaine et Comparée **53**(1): 101-115.

- l'Ambert, G., J. B. Ferré, F. Schaffner and D. Fontenille (2012). "Comparison of different trapping methods for surveillance of mosquito vectors of West Nile virus in Rhône Delta, France." Journal of Vector Ecology **37**(2): 269-275.

- L'Hostis, M., A. Bureaud and A. Gorenflot (1996). "Female *Ixodes ricinus* (Acari, Ixodidae) in cattle of Western France: infestation level and seasonality." Veterinary Research **27**(6): 589-597.

- L'Hostis, M., S. Chauvet, P. Devers and A. Chauvin (2007). "Tiques et babésioses : transmission et aspects cliniques chez les bovins." Le Nouveau Praticien Vétérinaire Élevages et Santé: 21-26.

- L'Hostis, M., A. Chauvin, H. Seegers and S. Memeteau (1997). "*Babesia divergens* et *Ixodes ricinus* : étude épidémiologique dans des exploitations de l'ouest de la France." Epidémiologie et Santé Animale: 31-32.

- L'Hostis, M., O. Diarra and H. Seegers (1994). "Sites of attachment and density assessment of female *Ixodes ricinus* (Acari: Ixodidae) on dairy cows." Experimental and Applied Acarology **18**: 681-689.

- L'Hostis, M., H. Dumon, B. Dorchies, F. Boisdron and A. Gorenflot (1995). "Seasonal incidence and ecology of the tick *Ixodes ricinus* (Acari: Ixodidae) on grazing pastures in Western France." Experimental and Applied Acarology **19**(4): 211-220.

- Lamontellerie, M. (1965). "Les tiques (Acarina, Ixodoidea) du sud-ouest de la France." Annales de Parasitologie Humaine et Comparée **40**(1): 87-100.

- Lantuejoul, C. (2015). "Trapping *Besnoitia* in cattle tests in Loire-Atantique." Le Nouveau Praticien Vétérinaire Élevages et Santé(32): 43-47.

- Le Goff, F., I. Humphery‐Smith, M. Leclercq and C. Chastel (1991). "Spiroplasmas from European Tabanidae." Medical and Veterinary Entomology **5**(1): 143-144.

- Le Goff, F., M. Marjolet, J. Guilloteau, I. Humphery-Smith and C. Chastel (1990). "Characterization and ecology of mosquito spiroplasmas from atlantic biotopes in France." Annales de Parasitologie Humaine et Comparée **65**(3): 107-110.

- Le Goff, F., M. Marjolet, I. Humphery-Smith, M. Leclercq, C. Hellas, F. Supplisson and C. Chastel (1993). "[Tabanid spiroplasmas from France: characterization, ecology and experimental study]." Annales de Parasitologie Humaine et Comparée **68**(3): 150-153.

- Lebert, I., A. Agoulon, S. Bastian, A. Butet, B. Cargnelutti, N. Cèbe, A. Chastagner, E. Léger, B. Lourtet and S. Masseglia (2020). "Distribution of ticks, tick-borne pathogens and the associated local environmental factors including small mammals and livestock, in two French agricultural sites: the OSCAR database." Biodiversity Data Journal **8**: e50123.

- Leblond, A., A. Chastagner, S. Pradier, X. Bailly, S. Masseglia and V. H. Gwenaël (2012). "La prévalence de l’anaplamose dans le sud de la France." Bulletin Epidémiologique, Santé Animale et Alimentation(49): 30-31.

- Leclercq, M. (1970). "Tabanidae (Diptera) des Pyrénées (Étude préliminaire)." Pirineos **95**: 75-90.

- Leclercq, M. (1971). "Tabanidae (Diptera) des Basses-Alpes." Bulletin des Recherches Agronomiques de Gembloux **6**: 424-431.

- Leclercq, M. (1987). "Attaques massives des animaux et de l’homme par les simulies (Diptères)." Revue Médicale de Liège **42**: 327-334.

- Leger, L. (1920). "Moustiques de Camargue. Un anopheliné nouveau pour la faune française, le *Myzorhynchus sinensis* Wied." Comptes Rendus de la Société de Biologie **83**: 1609-1610.

- Levasseur, G. (1997). "Les tiques dans le sud du Charolais : contrôle de l'infestation avec la moxidectine 1 % injectable " Bulletin des GTV **1**: 39-47.

- Liénard, E., A. Salem, C. Grisez, F. Prévot, J. P. Bergeaud, M. Franc, B. Gottstein, J. P. Alzieu, Y. Lagalisse and P. Jacquiet (2011). "A longitudinal study of *Besnoitia besnoiti* infections and seasonal abundance of *Stomoxys calcitrans* in a dairy cattle farm of Southwest France." Veterinary Parasitology **177**(1/2): 20-27.

- Macaigne, F. and C. Pérez-Eid (1993). "*Hyalomma scupense* Schulze, 1919 (Acarina, Ixodoidea) tique autochtone du sud-ouest de la France." Annales de Parasitologie Humaine et Comparée **68**(4): 199-200.

- Marhic, D. (1973). Contribution à l'étude de la désinsectisation chimique des locaux d'élevage, École Nationale Vétérinaire d'Alfort.

- Martinet, J.-P. (2021). Les moustiques de la région Grand-Est : biodiversité et compétence vectorielle pour des virus zoonotiques, Université de Reims Champagne-Ardenne.

- Martinod, S. and L. Joubert (1981). "L'écoépidémiologie prospective et les gradients de risques pathologiques spécifiques. Cartographie phyto-entomo-zoologique des piroplasmoses canines, bovines et équines du Jura méridional." Bulletin de la Société Scientifique Vétérinaire et Médicale Comparée de Lyon **83**(6): 291-297.

- Marty, P., A. Izri, C. Ozon, P. Haas, E. Rosenthal, P. Del Giudice, J. Godenir, E. Coulibaly, M. Gari-Toussaint, P. Delaunay, B. Ferrua, H. Haas, F. Pratlong and Y. Le Fichoux (2007). "A century of leishmaniasis in Alpes-Maritimes, France." Annals of Tropical Medicine and Parasitology **101**(7): 563-574.

- Marty, P. and Y. Le Fichoux (1988). "Épidémiologie de la leishmaniose dans le sud de la France." Pratique Médicale et Chirurgicale de l'Animal de Compagnie **23**: 11-15.

- Mathieu, B. (2011). Les espèces de *Culicoides* du sous-genre *Avaritia* (Diptera : Ceratopogonidae) dans le monde: révision systématique et taxonomique des espèces d'intérêt dans la transmission d'*Orbivirus*, Université de Strasbourg.

- Mathieu, B., J.-C. Delecolle, C. Garros, T. Balenghien, M.-L. Setier-Rio, E. Candolfi and C. Cêtre-Sossah (2011). "Simultaneous quantification of the relative abundance of species complex members: application to *Culicoides obsoletus* and *Culicoides scoticus* (Diptera: Ceratopogonidae), potential vectors of bluetongue virus." Veterinary Parasitology **182**(2-4): 297-306.

- Matsumoto, K., P. Parola, P. Brouqui and D. Raoult (2004). "*Rickettsia aeschlimannii* in *Hyalomma* ticks from Corsica." European Journal of Clinical Microbiology and Infectious Diseases **23**(9): 732-734.

- Medlock, J. M., K. M. Hansford, F. Schaffner, V. Versteirt, G. Hendrickx, H. Zeller and W. V. Bortel (2012). "A review of the invasive mosquitoes in Europe: ecology, public health risks, and control options." Vector-Borne and Zoonotic Diseases **12**(6): 435-447.

- Mehlhorn, H. (2012). Arthropods as vectors of emerging diseases. Londres (Angleterre), Springer Science & Business Media.

- Meiswinkel, R., T. Baldet, R. De Deken, W. Takken, J. Delécolle and P. Mellor (2007). Epidemiological analysis of the 2006 blue tongue virus serotype 8 epidemic in North-Western Europe. Distribution and dynamics of vector species**:** 88.

- Mellor, P. (2004). "Infection of the vectors and Bluetongue epidemiology in Europe." Veterinaria Italiana **40**(3): 176-181.

- Mellor, P. S. and E. J. Wittmann (2002). "Bluetongue virus in the Mediterranean basin 1998–2001." Veterinary journal **164**(1): 20-37.

- Mémeteau, S., H. Seegers, F. Jolivet and M. L'Hostis (1998). "Assessment of the risk of infestation of pastures by *Ixodes ricinus* due to their phyto-ecological characteristics." Veterinary Research **29**(5): 487-496.

- Menier, K. and J.-C. Beaucournu (1999). "Fleas of the genus *Ctenocephalides* Siles et Collins, 1930: medical and veterinary importance." Revue de Médecine Vétérinaire **150**(8-9): 675-680.

- Menier, K., T. Lejeune, F. Ple and J.-C. Beaucournu (1997). "*Ctenocephalides felis* (Bouche, 1835) (Insecta, Siphonaptera) heavy infestation on calves in Jura (France)." Revue de Médecine Vétérinaire **148**(7): 619-620.

- Meunier, A. (2007). Étude épidémiologique de la leishmaniose canine et de l'influence des facteurs environnementaux (en France depuis 1965, dans le Sud-Ouest en 2006). Ph.D, Ecole Nationale Vétérinaire de Lyon.

- Mialhe, E., C. Louis, J.-P. Quiot, J.-P. Rieb and C. Vaga (1982). Evidence and study of a Chlamydial infection in *Culicoides* sp. Proceedings of the Fifth International Symposium on Ceratopogonidae, Strasbourg.

- Michelet, L., S. Delannoy, E. Devillers, G. Umhang, A. Aspan, M. Juremalm, J. Chirico, F. J. van der Wal, H. Sprong and T. P. Boye Pihl (2014). "High-throughput screening of tick-borne pathogens in Europe." Frontiers in Cellular and Infection Microbiology **4**: Article 103.

- Michelet, L., G. Joncour, E. Devillers, A. Torina, M. Vayssier-Taussat, S. I. Bonnet and S. Moutailler (2016). "Tick species, tick-borne pathogens and symbionts in an insular environment off the coast of Western France." Ticks and Tick-borne Diseases **7**(6): 1109-1115.

- Mignotte, A. (2020). Maladies émergentes en santé animale: diversité génétique et dispersion d'une espèce de moucheron vectrice des virus de la Fièvre Catarrhale Ovine et de Schmallenberg en région paléarctique, *Culicoides obsoletus* (Diptera: Ceratopogonidae), Université de Montpellier.

- Mignotte, A., C. Garros, S. Dellicour, M. Jacquot, M. Gilbert, L. Gardès, T. Balenghien, M. Duhayon, I. Rakotoarivony and M. de Wavrechin (2021). "High dispersal capacity of *Culicoides obsoletus* (Diptera: Ceratopogonidae), vector of bluetongue and Schmallenberg viruses, revealed by landscape genetic analyses." Parasites and Vectors **14**(1): 1-14.

- Mignotte, A., C. Garros, L. Gardès, T. Balenghien, M. Duhayon, I. Rakotoarivony, L. Tabourin, L. Poujol, B. Mathieu and A. Ibañez-Justicia (2020). "The tree that hides the forest: cryptic diversity and phylogenetic relationships in the Palaearctic vector Obsoletus/Scoticus Complex (Diptera: Ceratopogonidae) at the European level." Parasites and Vectors **13**(1): 1-13.

- Moreau, A., J. Buisson and G. Bonjour (1987). "Study of deltametrhin "pour on" efficacy against flies and lice, cattle ectoparasites." Recueil de Médecine Vétérinaire: 245-257.

- Morel, P.-C. (1959). "Les *Hyalomma* (Acariens, Ixodidae) de France." Annales de Parasitologie Humaine et Comparée **34**(4): 552-555.

- Mouchet, J., J. Rageau and A. Chippaux (1969). "Hibernation de *Culex modestus* Ficalbi (Diptera, Culicidae) en Camargue." Cahier ORSTOM: Série Entomologie Médicale et Parasitologie **7**(1): 35-37.

- Mouchet, J., J. Rageau, C. Laumond, C. Hannoun, D. Beytout, J. Oudar, B. Corniou and A. Chippaux (1970). "Épidémiologie du virus West Nile: étude d’un foyer en Camargue. V. Le vecteur : *Culex modestus* Ficalbi Diptera." Annales de l'Institut Pasteur **118**: 839-855.

- Moussiegt, O. (1986). Moustiques de France. Bibliographie et répartition. Paris (France), Secretariat de la Faune et de la Flore.

- Moutailler, S., G. Krida, F. Schaffner, M. Vazeille and A.-B. Failloux (2008). "Potential vectors of Rift Valley fever virus in the Mediterranean region." Vector-Borne and Zoonotic Diseases **8**(6): 749-754.

- Mullen, G. R. and L. A. Durden (2009). Medical and veterinary entomology. Londres (Angleterre), Academic press.

- Neveu, A. and L. Lapchin (1978). "Écologie des principaux invertébrés filtreurs de la Basse Nivelle (Pyrénées-Atlantiques). I. Simuliidae (Diptera, Nematocera)." Annales de Limnologie **14**(3): 225-244.

- Ninio, C. (2011). Fièvre Catarrhale Ovine dans les Ardennes : étude de la biologie des *Culicoides* et de leur rôle épidémiologique, Reims.

- Ninio, C., D. Augot, J. C. Delecolle, B. Dufour and J. Depaquit (2011a). "Contribution to the knowledge of *Culicoides* (Diptera: Ceratopogonidae) host preferences in France." Parasitology Research **108**(3): 657-663.

- Ninio, C., D. Augot, B. Dufour and J. Depaquit (2011b). "Emergence of *Culicoides obsoletus* from indoor and outdoor breeding sites." Veterinary Parasitology **183**(1-2): 125-129.

- Noirtin, C. and P. Boiteux (1979). "[Death of 25 farm animals (including 24 bovines) as a result of simuliid bites in the Vosges]." Bulletin de la Société Vétérinaire Pratique de France **63**: 41-54.

- Noirtin, C., P. Boiteux, P. Guillet, C. Dejoux, F. Beaucournu-Saguez and J. Mouchet (1981). "Les simulies, nuisance pour le bétail dans les Vosges : les origines de leur pullulation et les méthodes de lutte." Cahier ORSTOM: Série Entomologie Médicale et Parasitologie **19**: 101-112.

- Noureddine, R., A. Chauvin and O. Plantard (2011). "Lack of genetic structure among Eurasian populations of the tick *Ixodes ricinus* contrasts with marked divergence from north-African populations." International Journal for Parasitology **41**(2): 183-192.

- Nuttall, G. H. F., W. F. Cooper, C. Warburton and L. E. Robinson (1911). Ticks, a monograph of the Ixodoidea. Londres (Angleterre), Welcolme collection.

- OIE (2000). "Bluetongue in France in the island of Corsica." OIE news **13**(43): 195-197.

- Pannerer, X. l. (1980). Les nouveaux pyréthrinoïde et les mouches du betail. Essai clinique de la permethrine, Ecole Nationale Vétérinaire d'Alfort.

- Parola, P., L. Beati, M. Cambon and D. Raoult (1998). "First isolation of *Rickettsia helvetica* from *Ixodes ricinus* ticks in France." European Journal of Clinical Microbiology and Infectious Diseases **17**(2): 95-100.

- Parola, P. and D. Raoult (2001). "Molecular tools in the epidemiology of tick-borne bacterial diseases." Annales de Biologie Clinique **59**(2): 177-182.

- Pellerin, J. (2003). Épidémiologie de la babésiose bovine à *Babesia divergens* : étude spécifique dans le département du Calvados, Faculté de Médecine de Nantes.

- Perez-Eid, C. (1989). "Dynamique saisonnière des nymphes et adultes d'*Ixodes ricinus* en phase libre sur la végétation, dans le foyer alsacien d'encéphalite à tiques." Acarologia **30**(4): 355-360.

- Pérez-Eid, C. (2007). Les tiques : identification, biologie, importance médicale et vétérinaire. Paris (France), Editions Tec&Doc.

- Pernot-Visentin, O. and F. Beaucournu-Saguez (1974). "Les Tabanidae (Diptera) de France." Publications de la Société Linnéenne de Lyon **43**(5): 142-155.

- Perrin, A., C. Cetre‐Sossah, B. Mathieu, T. Baldet, J. C. Delecolle and E. Albina (2006). "Phylogenetic analysis of *Culicoides* species from France based on nuclear ITS1‐rDNA sequences." Medical and Veterinary Entomology **20**(2): 219-228.

- Personne, F. (2002). "Maîtrise d'une infestation de tiques avec la Versatrine® en élevages bovins de race charolaise." Bulletin de la Société Vétérinaire Pratique de France **86**(5): 292-295.

- Pichon, B., L. Mousson, C. Figureau, F. Rodhain and C. Perez-Eid (1999). "Density of deer in relation to the prevalence of *Borrelia burgdorferi* s.l. in *Ixodes ricinus* nymphs in Rambouillet forest, France." Experimental and Applied Acarology **23**(3): 267-275.

- Pichot, J., B. Gilot, V. Soulier, A. Rey-Coquais, B. Degeilh and B. Doche (1994). "Ecoépidemiologie de la borréliose de lyme dans la région Rhône-Alpes. Répartition, contexte écologique, relations avec la distribution d'*Ixodes* *ricinus* (Linné, 1758)." Parasite **1**(4): 335-342.

- Ponçon, N. (2008). Étude des risques de ré-émergence du paludisme en Camargue, Université de Montpellier 2.

- Ponçon, N., T. Balenghien, C. Toty, J. B. Ferré, C. Thomas, A. Dervieux, G. L’Ambert, F. Schaffner, O. Bardin and D. Fontenille (2007a). "Effects of local anthropogenic changes on potential malaria vector *Anopheles hyrcanus* and West Nile virus vector *Culex modestus*, Camargue, France." Emerging Infectious Diseases **13**(12): 1810-1815.

- Ponçon, N., C. Toty, G. L'Ambert, G. Le Goff, C. Brengues, F. Schaffner and D. Fontenille (2007b). "Biology and dynamics of potential malaria vectors in Southern France." Malaria Journal **6**(1): 1-9.

- Pradel, J., T. Martin, D. Rey, R. Foussadier and D. Bicout (2008). "*Culex modestus* (Ficalbi), vecteur de virus West Nile en Camargue : une espèce en augmentation dans la Dombe ?" Epidémiologie et Santé Animale **53**: 105-119.

- Prudhomme, J., C. Cassan, M. Hide, C. Toty, N. Rahola, B. Vergnes, J. P. Dujardin, B. Alten, D. Sereno and A. L. Banuls (2016). "Ecology and morphological variations in wings of *Phlebotomus ariasi* (Diptera: Psychodidae) in the region of Roquedur (Gard, France): a geometric morphometrics approach." Parasites and Vectors **9**(1): 578-570.

- Prudhomme, J., T. De Meeus, C. Toty, C. Cassan, N. Rahola, B. Vergnes, R. Charrel, B. Alten, D. Sereno and A. L. Banuls (2020). "Altitude and hillside orientation shapes the population structure of the *Leishmania infantum* vector *Phlebotomus ariasi*." Scientific Reports **10**(1): 14443-14454.

- Prudhomme, J., N. Rahola, C. Toty, C. Cassan, D. Roiz, B. Vergnes, M. Thierry, J. A. Rioux, B. Alten, D. Sereno and A. L. Banuls (2015). "Ecology and spatiotemporal dynamics of sandflies in the Mediterranean Languedoc region (Roquedur area, Gard, France)." Parasites and Vectors **8**(1): 642-655.

- Quessada, T., F. Martial-Convert, S. Arnaud, H. Leudet De La Vallee, B. Gilot and J. Pichot (2003). "Prevalence of *Borrelia burgdorferi* species and identification of *Borrelia valaisiana* in questing *Ixodes ricinus* in the Lyon region of France as determined by polymerase chain reaction-restriction fragment length polymorphism." European Journal of Clinical Microbiology and Infectious Diseases **22**(3): 165-173.

- Rageau, J. (1972). "Répartition géographique et rôle pathogène des tiques (acariens : Argasidae et Ixodidae) en France." Wiadomości Parazytologiczne **18**(4-5-6): 707-719.

- Rageau, J. and J. Mouchet (1967). "Les arthropodes hématophages de Camargue." Cahier ORSTOM: Série Entomologie Médicale et Parasitologie **5**(4): 263-281.

- Rageau, J. and J. Mouchet (1970a). "Les tiques de Camargue (Acariens, Ixodoidea) et leur rôle dans l’épidémiologie d’arbovirus." Folia Parasitologica **17**: 349-353.

- Rageau, J., J. Mouchet and E. Abonnenc (1970b). "Répartition géographique des moustiques (Diptera : Culicidae) en France." Cahier ORSTOM: Série Entomologie Médicale et Parasitologie **8**: 289-316.

- Ramilo, D., C. Garros, B. Mathieu, C. Benedet, X. Allene, E. Silva, G. Alexandre-Pires, I. P. Da Fonseca, S. Carpenter and J. Radrova (2013). "Description of *Culicoides paradoxalis* sp. nov. from France and Portugal (Diptera: Ceratopogonidae)." Zootaxa **3745**(2): 243-256.

- Ramsdale, C. and K. Snow (2000). "Distribution of the genus *Anopheles* in Europe." European Mosquito Bulletin(7): 1-26.

- Raymond, H.-L. (1979). "Répartition écologique des Tabanidae (Diptera) adultes du département des Hautes-Alpes." Publications de la Société Linnéenne de Lyon **48**(7): 453-456.

- Raymond, H. (1978). "Horse flies (Tabanidae) of Camargue - Preliminary report." Revue d'Ecologie - la Terre et la Vie **32**(2): 291-303.

- Raynal, J. H. (1954). "Les phlébotomes de France et leur distribution régionale." Annales de Parasitologie Humaine et Comparée **29**(3): 297-323.

- Reis, C., M. Cote, R. E. Paul and S. Bonnet (2011). "Questing ticks in suburban forest are infected by at least six tick-borne pathogens." Vector-Borne and Zoonotic Diseases **11**(7): 907-916.

- Richter, D. and F.-R. Matuschka (2006). "Modulatory effect of cattle on risk for lyme disease." Emerging Infectious Diseases **12**(12): 1919-1923.

- Rieb, J.-P. (1987). "L'estivo-hibernation et le contrôle de la dynamique du cycle évolutif dans le genre *Culicoides* (Diptères, Cératopogonidés)." Vie et Milieu **37**(1): 23-37.

- Rieb, J.-P., E. Mialhe and J.-M. Quiot (1982). Ceratopogonidae larvae infected by an Iridovirus. Proceedings of the Fifth International Symposium on Ceratopogonidae, Strasbourg.

- Rioux, J.-A. and M. Arnold (1955). "Les Culicidés de Camargue (étude systématique et écologique)." Revue d'Ecologie - la Terre et la Vie(4): 244-251.

- Rioux, J.-A., H. Croset, J.-J. Corre, P. Simoneau and G. Gras (1967). "Les bases phyto-écologiques de la lutte anticulicidienne - Cartographie des biotopes larvaires. Ses applications opérationnelles dans le “Midi” méditerranéen." Annales de Parasitologie Humaine et Comparée **42**(6): 665-680.

- Rioux, J.-A., S. Descous and J. Pech (1959). "Un nouveau Cératopogonide arboricole : *Culicoides haranti* n. sp. (Diptera, Heleidae)." Annales de Parasitologie Humaine et Comparée **34**(3): 432-438.

- Rioux, J.-A., Y. Golvan, H. Croset, S. Tour, R. Houin, E. Abonnenc, M. Petitdidier, Y. Vollhardt, J. P. Dedet, J. L. Albaret, G. Lanotte, M. Quilici, A. Martini-Dumas, M. Maistre, A. Brès, T. Roviralta and F. Vila (1969). Épidémiologie des leishmanioses dans le Sud de la France. Paris (France), Monographie de l'Institut National de la Santé et de la Recherche Médicale.

- Rioux, J.-A., R. Killick-Kendrick, A. Leaney, D. Turner, M. Bailly and C. Young (1979). "Écologie des leishmanioses dans le Sud de la France. 12. Dispersion horizontale de *Phlebotomus ariasi* Tonnoir, 1921. Expériences préliminaires." Annales de Parasitologie Humaine et Comparée **54**(6): 673-682.

- Rioux, J. A., S. Carron, J. Dereure, J. Perieres, L. Zeraia, E. Franquet, M. Babinot, M. Gallego and J. Prudhomme (2013). "Ecology of leishmaniasis in the South of France. 22. Reliability and representativeness of 12 *Phlebotomus ariasi*, *P*. *perniciosus* and *Sergentomyia minuta* (Diptera: Psychodidae) sampling stations in Vallespir (Eastern French Pyrenees region)." Parasite **20**: 34-43.

- Rizzoli, A., C. Silaghi, A. Obiegala, I. Rudolf, Z. Hubálek, G. Földvári, O. Plantard, M. Vayssier-Taussat, S. Bonnet and E. Špitalská (2014). "*Ixodes ricinus* and its transmitted pathogens in urban and peri-urban areas in Europe: new hazards and relevance for public health." Frontiers in Public Health **2**: Article 251.

- Roche, B., L. Léger, G. L’Ambert, G. Lacour, R. Foussadier, G. Besnard, H. Barré-Cardi, F. Simard and D. Fontenille (2015). "The spread of *Aedes albopictus* in metropolitan France: contribution of environmental drivers and human activities and predictions for a near future." PLOS One **10**(5): e0125600.

- Roman, E. and J. Pichot (1973). "Étude biogéographique et écologique sur les tiques (Acariens Ixodoidea) de la Région Lyonnaise." Publications de la Société Linnéenne de Lyon **42**(10): 65-73.

- Rossi, P., P. Guislain and J. Bissoéras (1955). "Un nouveau foyer d'anaplasmose bovine en Saône-et-Loire." Bulletin de l'Académie Vétérinaire de France: 121-129.

- Rossi, S., T. Balenghien, C. Viarouge, E. Faure, G. Zanella, C. Sailleau, B. Mathieu, J.-C. Delécolle, C. Ninio, C. Garros, L. Gardès, C. Tholoniat, A. Ariston, D. Gauthier, S. Mondoloni, A. Barboiron, M. Pellerin, P. Gibert, C. Novella, S. Barbier, E. Guillaumat, S. Zientara, D. Vitour and E. Bréard (2019). "Red deer (*Cervus elaphus*) did not play the role of maintenance host for Bluetongue virus in France: the burden of proof by long-term wildlife monitoring and *Culicoides* snapshots." Viruses **11**(10): 903-929.

- Roubaud, E. (1918). "Recherches sur la transmission du paludisme par les anophèles français de régions non palustres (Yonne et région parisienne)." Annales de l'Institut Pasteur **32**(430): 427-462.

- Roubaud, E. (1920). "Les conditions de nutrition des anophèles en France (*A. maculipennis*) et le rôle du bétail dans la prophylaxie du paludisme." Annales de l'Institut Pasteur **34**: 181-228.

- Roubaud, E. (1921). "La différenciation des races zootropiques d'anophèles et la régression spontanée du paludisme." Bulletin de la Société de Pathologie Exotique **14**: 577-595.

- Roubaud, E. (1928). "Nouvelles recherches sur l’évolution zoophile des faunes d’anophèles en Europe (*A. maculipennis*) d’après les données de l’armement maxillaire." Annales de l'Institut Pasteur **XLII**: 553-619.

- Rouet, D. (2011). Dynamique des populations de *Stomoxys calcitrans* dans un site urbain, l'École Nationale Vétérinaire de Toulouse. Ph.D, École Nationale Vétérinaire de Toulouse.

- Rubel, F., K. Brugger, M. Pfeffer, L. Chitimia-Dobler, Y. M. Didyk, S. Leverenz, H. Dautel and O. Kahl (2016). "Geographical distribution of *Dermacentor marginatus* and *Dermacentor reticulatus* in Europe." Ticks and Tick-borne Diseases **7**(1): 224-233.

- Ruffié, J. (1957). Contribution à l'étude bio-systématique des Culicinae du sous-bassin aquitain. Ph.D, Faculté de Médecine de Toulouse.

- Sailleau, C., E. Bréard, G. Gerbier, J. Parodil, A. Bouchot and S. Zinetara (2005). "Épidémiologie descriptive et moléculaire de la Bluetongue en Corse en 2004." Epidémiologie et Santé Animale **48**: 9-14.

- Salem, A. (2012). *Stomoxys calcitrans* (L. 1758) : morphologie, biologie, rôle vecteur et moyens de lutte. Ph.D, École Nationale Vétérinaire de Toulouse.

- Sautet, J. (1944). "A propos d'une épidémie de paludisme en Camargue." Marseille Médical **2**(15): 53-64.

- Schaffner, F. and S. Karch (2000). "First record of *Aedes albopictus* (Skuse, 1894) in metropolitan France." Comptes Rendus de l'Académie des Sciences de Paris serie 3 **323**(4): 373-376.

- Séguy, E. (1923). Histoire naturelle des moustiques de France : étude systématique et biologique des moustiques de l'Europe centrale et septentrionale et de leurs parasites. Paris (France), Lechevalier P.

- Séguy, E. (1924). Les insectes parasites de l'Homme et des animaux domestiques. Paris (France), Lechevalier P.

- Séguy, E. (1926). Faune de France. 13. Diptères (Brachycères) (Stratiomuiidae, Erinnidae, Cœnomyiidae, Rhagionidae, Tabanidae, Codidae, Nemestrinidae, Mydaidae, Bombyliidae, Therevidae, Omphralidae). Paris (France), Lechevalier P.

- Séguy, E. (1944). Faune de France. 43. Insectes ectoparasites (Mallophages, Anoploures, Siphonaptères). Paris (France), Lechevalier P. et fils.

- Senevet, G. (1937). Faune de France. 32. Ixodoidés. Paris (France), Lechevalier P.

- Sharif, S. (2018). *Stomoxys calcitrans* : évaluation du rôle dans la transmission de *Besnoitia besnoiti* et nouveaux moyens de contrôle. Ph.D, École Nationale Vétérinaire de Toulouse.

- Sharif, S., E. Liénard, G. Duvallet, L. Etienne, C. Mongellaz, C. Grisez, M. Franc, E. Bouhsira and P. Jacquiet (2020). "Attractiveness and specificity of different polyethylene blue screens on *Stomoxys calcitrans* (Diptera: Muscidae)." Insects **11**(9): 574-586.

- Simon, P. H., P. Rancien and J. Euzeby (1974). "A propos de l'endémie charollaise de piroplasmose bovine. II. Note : étude épidémiologique et conséquences prophylactiques." Bulletin de la Société Scientifique Vétérinaire et Médicale Comparée de Lyon **76**(6): 411-421.

- Stachurski, F. and L. Vial (2018). "Installation de la tique *Hyalomma marginatum*, vectrice du virus de la Fièvre Hémorragique de Crimée-Congo, en France continentale." Bulletin Epidémiologique, Santé Animale et Alimentation **84**(9): 1-5.

- Surcouf, J. (1921). "Notes biologiques sur certains Diptères." Bulletin de la Musée d’Histoire Naturelle de Paris **27**: 67-74.

- Takken, W. and B. G. Knols (2007). Emerging pests and vector-borne diseases in Europe. Wageningen (Pays-Bas), Wageningen Academic Publishers.

- Temmam, S., T. Bigot, D. Chrétien, M. Gondard, P. Pérot, V. Pommelet, E. Dufour, S. Petres, E. Devillers and T. Hoem (2019). "Insights into the host range, genetic diversity, and geographical distribution of Jingmenviruses." MSphere **4**(6): e00645-00619.

- Theodorides, J. (1954). "Première contribution à l'étude des ectoparasites de Vertébrés des Pyrénées-Orientales." Vie et Milieu **4**: 753-756.

- Tran, A., F. Biteau-Coroller, H. Guis and F. Roger (2005). "Modélisation des maladies vectorielles." Epidémiologie et Santé Animale **47**: 35-51.

- Treillard, M. (1937). "Anophèles de Camargue." Bulletin de la Société de Pathologie Exotique **XXX**: 136-139.

- Vassallo, M., R. Paul and C. Perez-Eid (2000a). "Temporal distribution of the annual nymphal stock of *Ixodes ricinus* ticks." Experimental and Applied Acarology **24**(12): 941-949.

- Vassallo, M., B. Pichon, J. Cabaret, C. Figureau and C. Pérez-Eid (2000b). "Methodology for sampling questing nymphs of *Ixodes ricinus* (Acari: Ixodidae), the principal vector of lyme disease in Europe." Journal of Medical Entomology **37**(3): 335-339.

- Vayssier-Taussat, M., S. Moutailler, L. Michelet, E. Devillers, S. Bonnet, J. Cheval, C. Hébert and M. Eloit (2013). "Next generation sequencing uncovers unexpected bacterial pathogens in ticks in Western Europe." PLOS One **8**(11): e81439.

- Venail, R. (2014). Sensibilité aux insecticides et évaluation préliminaire des méthodes de lutte antivectorielle disponibles contre les *Culicoides* (Diptera : Ceratopogonidae) Paléarctiques, vecteurs de virus émergents d'intérêt en santé animale, Université de Montpellier 2.

- Viennet, E. (2011). Insectes et maladies émergentes : Contacts hôte/*Culicoides* en région Paléarctique et leurs implications dans la transmission de la Fièvre Catarrhale Ovine, Université de Montpellier 2.

- Viennet, E., C. Garros, L. Gardes, I. Rakotoarivony, X. Allene, R. Lancelot, D. Crochet, C. Moulia, T. Baldet and T. Balenghien (2013). "Host preferences of Palaearctic *Culicoides* biting midges: implications for transmission of orbiviruses." Medical and Veterinary Entomology **27**(3): 255-266.

- Viennet, E., C. Garros, I. Rakotoarivony, X. Allene, L. Gardès, J. Lhoir, I. Fuentes, R. Venail, D. Crochet and R. Lancelot (2012). "Host-seeking activity of Bluetongue virus vectors: endo/exophagy and circadian rhythm of *Culicoides* in Western Europe." PLOS One **7**(10): e48120.

- Vinçon, G. and M. Clergue-Gazeau (1988). "Étude hydrobiologique de la vallée d'Ossau (Pyrénées-Atlantiques, France). III. Simuliidae (Diptera, Nematocera) : leur originalité biogéographique et écologique." Annales de Limnologie **24**(1): 67-81.

- Vourc’h, G., C. Boyard and J. Barnouin (2008). "Mammal and bird species distribution at the woodland–pasture interface in relation to the circulation of ticks and pathogens." Annals of the New York Academy of Sciences **1149**(1): 322-325.

- Wailly, P. (1993). "La maladie de lyme." Bulletin de la Société Vétérinaire Pratique de France **77**(8): 387-392.

- Waller, J., M. Kremer and D. J.C. (1982). Turf-moss *Culicoides* in the Hautes-Vosges (France). Proceedings of the Fifth International Symposium on Ceratopogonidae, Strasbourg.

- Zientara, S., T. Baldet, B. Durand, J. Hars, C. Lagneau, X. de Lamballerie, B. Murgue, P. Reiter, A. Hattenberger and F. Gauchard (2004). Rapport sur la surveillance de l'infection à virus West-Nile en France. Paris (France), AFSSA**:** 48.

- Zientara, S., S. De La Rocque, J.-M. Gourreau, M. Grégory, A. Diallo, P. Hendrikx, G. Libeau, C. Sailleau and J. C. Delecolle (2000). "La Fièvre Catarrhale Ovine en Corse en 2000." Epidémiologie et Santé Animale **38**: 133-144.

- Zientara, S., C. Grillet, S. De la Rocque, J. M. Gourreau, M. Grégory, P. Hendrikx, G. Libeau, C. Sailleau, E. Albina, E. Bréard and J. C. Delécolle (2001). "La Fièvre Catarrhale Ovine en Corse en 2001." Epidémiologie et Santé Animale **40**: 129-134.
