# Supplementary material for: Systematic review of hematophagous arthropods present in cattle in France
Source: Parasite. 2023 Dec 12;30:56. doi: 10.1051/parasite/2023059 (PMC10714678; doi:10.1051/parasite/2023059)
Supplement: Supplementary file 1 — Supplementary Figure 1. Distribution map of the different hematophagous arthropods identified in the systematic review by department according to the number of references and, if available, the percentage of capture. Supplementary Table 1. Complete list of references included in the systematic review. Supplementary Table 2. Description of captures by species of the different hematophagous arthropods identified in the systematic review. [file parasite-30-56-s1.zip › Suppl_Tab_2.docx]

# Supplementary Table Legend

[Supplementary Table S1. Captures of *Ctenocephalides* *felis* in cattle farms in France between 1957 and 2016 (NA: data not provided). 2](#_Toc134519758)

[Supplementary Table S2. Captures of lice in cattle farms in France between 1635 and 2017 (NA: data not provided). 3](#_Toc134519759)

[Supplementary Table S3. Captures of horse fly in cattle farms in France between 1758 and 2022 (NA: data not provided). 4](#_Toc134519760)

[Supplementary Table S4. Captures of *Hippobosca* *equina* in cattle farms in France between 1758 and 2022 (NA: data not provided). 39](#_Toc134519761)

[Supplementary Table S5. Captures of biting flies in cattle farms in France between 1920 and 2022 (NA: data not provided). 40](#_Toc134519762)

[Supplementary Table S6. Captures of black flies in cattle farms in France between 1868 and 2017 (NA: data not provided). 44](#_Toc134519763)

[Supplementary Table S7. Captures of sand flies in cattle farms in France between 1909 and 2013 (NA: data not provided). 55](#_Toc134519764)

[Supplementary Table S8. Captures of mosquitoes in cattle farms in France between 1758 and 2022 (NA: data not provided). 61](#_Toc134519765)

[Supplementary Table S9. Captures of biting midges in cattle farms in France between 1953 and 2016 (NA: data not provided). 129](#_Toc134519766)

[Supplementary Table S10. Captures of ticks in cattle farms in France between 1888 and 2023 (NA: data not provided). 191](#_Toc134519767)

Supplementary Table S1. Captures of Ctenocephalides felis in cattle farms in France between 1957 and 2016 (NA: data not provided).

| Capture | | | | | | | Reference |
| --- | --- | --- | --- | --- | --- | --- | --- |
| Department | Year | Study  duration  (month) | Month | Season | Altitude  (m) | Trapping  method |
| Jura | 1995-1996 | 2 | September, April | Autumn,  Spring | 250 | Comb | Menier et al. (1997) |
| Jura | 1995-1996 | NA | NA | NA | NA | Comb | Menier et al. (1999) |
| Alpes-de-Haute-Provence, Bouches-du-Rhône, Charente-Maritime, Drôme, Gard, Corse, Ille-et-Vilaine, Indre-et-Loire, Jura, Loire-Atlantique, Loiret, Maine-et-Loire, Nord, Paris, Puy-de-Dôme, Rhône, Sarthe, Seine-et-Marne, Val-de-Marne, Yonne, Yvelines | 1957-1959, 1961-1963, 1969, 1971, 1973-1974, 1977, 1983-1984, 1986, 1989, 1994-1995, 2001, 2008-2009, 2012-2015 | NA | January, February, March, April, May, July, August, September, October, November, December | Winter,  Spring,  Summer,  Autumn | NA | NA | INPN (2023) |
| All France | NA | NA | NA | NA | NA | NA | Mullen et al. (2009) |
| Alpes-de-Haute-Provence, Alpes-Maritimes, Bouches-du-Rhône, Charente-Maritime, Corse, Drôme, Gard, Ille-et-Vilaine, Indre-et-Loire, Jura, Loire-Atlantique, Loiret, Maine-et-Loire, Nord, Paris, Puy-de-Dôme, Rhône, Sarthe, Seine-et-Marne, Seine-Saint-Denis, Val-de-Marne, Yonne, Yvelines | 1957-1959, 1961-1963, 1969, 1971, 1973-1974, 1977, 1986, 1989, 1994-1995, 2008-2009, 2013-2016 | NA | January, February, March, April, July, August, September, October, November, December | Winter,  Spring,  Summer,  Autumn | NA | NA | GBIF (2023) |

Supplementary Table S2. Captures of lice in cattle farms in France between 1635 and 2017 (NA: data not provided).

| Species | Capture | | | | | | | Reference |
| --- | --- | --- | --- | --- | --- | --- | --- | --- |
|  | Department | Year | Study  duration  (month) | Month | Season | Altitude  (m) | Trapping  method |  |
| *Haematopinus*  *eurysternus* | Rhône, Loire | 2016-2017 | 2 | December, February | Winter | NA | Comb | Devos et al. (2018) |
|  | Saône-et-Loire | 1985-1986 | 2 | July, August, September, December, January | Summer,  Winter | 260 | Manual capture,  Comb | Moreau et al. (1987) |
|  | All France | NA | NA | NA | NA | NA | Manual capture | Séguy (1944) |
|  | Saône-et-Loire | 2011 | 1 | December | Winter | NA | Comb | Courouble et al. (2012) |
| *Linognathus*  *vituli* | Rhône, Loire | 2016-2017 | 2 | December, February | Winter | NA | Comb | Devos et al. (2018) |
|  | Saône-et-Loire | 2011 | 1 | December | Winter | NA | Comb | Courouble et al. (2012) |
|  | Saône-et-Loire | 1985-1986 | 2 | July, August, September, December, January | Summer,  Winter | 260 | Manual capture,  Comb | Moreau et al. (1987) |
|  | Corrèze | 1997 | NA | NA | NA | NA | Comb | Dorffer (1998) |
|  | Hauts-de-Seine, Corse | 1635, 1986 | NA | January, May | Winter,  Spring | NA | NA | INPN (2023) |
|  | All France | NA | NA | NA | NA | NA | NA | Mullen et al. (2009) |
|  | All France | NA | NA | NA | NA | NA | Manual capture | Séguy (1944) |
| *Solenopotes*  *capillatus* | Rhône, Loire | 2016-2017 | 2 | December, February | Winter | NA | Comb | Devos et al. (2018) |
|  | All France | NA | NA | NA | NA | NA | NA | Mullen et al. (2009) |
|  | All France | NA | NA | NA | NA | NA | Manual capture | Séguy (1944) |

Supplementary Table S3. Captures of horse fly in cattle farms in France between 1758 and 2022 (NA: data not provided).

| Species | Capture | | | | | | | Pathogen | Reference |
| --- | --- | --- | --- | --- | --- | --- | --- | --- | --- |
|  | Department | Year | Study  duration  (month) | Month | Season | Altitude  (m) | Trapping  method |  |  |
| *Atylotus*  *agrestis* | Pyrénées-Orientales | 1955-1968 | NA | June, July, August | Summer | NA | NA | NA | Leclercq (1970) |
|  | Hérault, Gard, Bouches-du-Rhône | 1953-1966 | NA | January, February, March, April, May, June, July, August, September, October, November, December | Winter,  Spring,  Summer,  Autumn | NA | Manual capture,  Larvae capture,  Malaise trap | NA | Rageau et al. (1967) |
|  | Gard | 1834-1926 | NA | April, May, June, July, August, September | Spring,  Summer,  Autumn | NA | NA | NA | Séguy (1926) |
| *Atylotus*  *flavoguttatus* | Bouches-du-Rhône | 2011 | 1 | June | Summer | 10 | N’zi trap | NA | Baldacchino et al. (2013a) |
|  | Bouches-du-Rhône | 2012 | 1 | June, July | Summer | 10 | N’zi trap (with and without odor bait) | NA | Baldacchino et al. (2013b) |
|  | Pyrénées-Orientales | 1955-1968 | NA | June, July, August | Summer | NA | NA | NA | Leclercq (1970) |
|  | Hérault, Gard, Bouches-du-Rhône | 1953-1966 | NA | January, February, March, April, May, June, July, August, September, October, November, December | Winter,  Spring,  Summer,  Autumn | NA | Manual capture,  Larvae capture,  Malaise trap | NA | Rageau et al. (1967) |
|  | Bouches-du-Rhône | 1972-1973 | 6 | May, June, July, August, September | Spring,  Summer | NA | Manitoba trap,  Net capture,  Larvae capture | NA | Raymond (1978) |
| *Atylotus*  *fulvus* | Pyrénées-Orientales | 2012 | 2 | July, August | Summer | NA | N’zi trap | NA | Baldacchino et al. (2014a) |
|  | Pyrénées-Orientales | 2011 | 0.2 | July | Summer | 1450-2700 | N’zi trap | NA | Baldacchino et al. (2014c) |
|  | Pyrénées-Orientales | 2011 | 5 | May, June, July, August, September | Spring,  Summer | 850-2300,  1450-2700 | N’zi and  Vavoua traps | NA | Baldacchino et al. (2014b) |
|  | Loire-Atlantique | 2012 | 5 | April, May, June, July, August | Summer | NA | N’zi trap | NA | Lantuejoul (2015) |
|  | Loire-Atlantique | 2013-2014 | NA | NA | NA | NA | NA | NA | Jacquiet et al. (2015) |
|  | Maine-et-Loire | 2008 | 1 | July | Summer | 71 | Vavoua trap | NA | Grimeau (2012) |
|  | Alpes-de-Haute-Provence, Cantal, Cher, Haut-Rhin, Loire, Manche, Nièvre, Vendée | 2006, 2010, 2014-2015, 2017 | NA | January, May, June, July, August | Winter,  Spring,  Summer | NA | NA | NA | INPN (2023) |
|  | Hautes-Pyrénées, Ariège, Haute-Garonne, Pyrénées-Orientales | 1955-1968 | NA | June, July, August | Summer | NA | NA | NA | Leclercq (1970) |
|  | Alpes-de-Haute-Provence | 1957-1969 | NA | June, July, August | Summer | 250-3400 | NA | NA | Leclercq (1971) |
|  | Corrèze, Doubs | 1953-1971 | NA | NA | NA | NA | NA | NA | Pernot-Visentin et al. (1974) |
|  | Bouches-du-Rhône | 1972-1973 | 6 | May, June, July, August, September | Spring,  Summer | NA | Manitoba trap,  Net capture,  Larvae capture | NA | Raymond (1978) |
|  | Hautes-Alpes | 1972-1973, 1978 | 3 | July, August | Summer | NA | Manitoba trap,  Manual capture | NA | Raymond (1979) |
|  | All France | NA | NA | NA | NA | NA | NA | NA | Séguy (1924) |
|  | All France | 1834-1926 | NA | April, May, June, July, August, September | Spring,  Summer,  Autumn | NA | NA | NA | Séguy (1926) |
|  | Alpes-de-Haute-Provence, Cantal, Cher, Haut-Rhin, Loire, Manche, Nièvre, Vaucluse, Vendée | 2006, 2010, 2012, 2014-2015, 2017 | NA | May, June, July, August | Spring,  Summer | NA | NA | NA | GBIF (2023) |
| *Atylotus*  *intermedius* | Côtes-d’Armor, Morbihan, Côte-d'Or, Puy-de-Dôme, Hautes-Alpes, Alpes-Maritimes, Var, Hautes-Pyrénées, Corse | 1834-1926 | NA | April, May, June, July, August, September | Spring,  Summer,  Autumn | NA | NA | NA | Séguy (1926) |
| *Atylotus*  *latistriatus* | Gironde, Côtes-d'Armor | 2015, 2019 | NA | July, August | Summer | NA | NA | NA | INPN (2023) |
|  | Pyrénées-Orientales | 1955-1968 | NA | June, July, August | Summer | NA | NA | NA | Leclercq (1970) |
|  | Hérault, Gard, Bouches-du-Rhône | 1953-1966 | NA | January, February, March, April, May, June, July, August, September, October, November, December | Winter,  Spring,  Summer,  Autumn | NA | Manual capture,  Larvae capture,  Malaise trap | NA | Rageau et al. (1967) |
|  | Bouches-du-Rhône | 1972, 1973 | 6 | May, June, July, August, September | Spring,  Summer | NA | Manitoba trap,  Net capture,  Larvae capture | NA | Raymond (1978) |
|  | Morbihan, Landes, Pyrénées-Orientales | 1834-1926 | NA | April, May, June, July, August, September | Spring,  Summer,  Autumn | NA | NA | NA | Séguy (1926) |
|  | Côtes-d'Armor | 2019 | NA | August | Summer | NA | NA | NA | GBIF (2023) |
| *Atylotus*  *loewianus* | Alpes-de-Haute-Provence, Alpes-Maritimes, Ariège, Hérault, Hautes-Alpes, Loir-et-Cher, Maine-et-Loire | 1968, 2006, 2010, 2013, 2015-2016 | NA | May, June, July, August, September | Spring,  Summer | NA | NA | NA | INPN (2023) |
|  | Pyrénées-Orientales | 1955-1968 | NA | June, July, August | Summer | NA | NA | NA | Leclercq (1970) |
|  | Alpes-de-Haute-Provence | 1957-1969 | NA | June, July, August | Summer | 250-3400 | NA | NA | Leclercq (1971) |
|  | Alpes-Maritimes, Alpes-de-Haute-Provence, Corrèze | 1953-1971 | NA | NA | NA | NA | NA | NA | Pernot-Visentin et al. (1974) |
|  | Hautes-Alpes | 1972-1973, 1978 | 3 | July, August | Summer | NA | Manitoba traps,  Manual capture | NA | Raymond (1979) |
|  | Alpes-de-Haute-Provence, Alpes-Maritimes, Ariège, Hérault, Loir-et-Cher, Maine-et-Loire | 1968, 2010, 2013, 2016, 2021 | NA | June, July, August | Summer | NA | NA | NA | GBIF (2023) |
| *Atylotus*  *plebeius* | Hérault, Gard, Bouches-du-Rhône | 1953-1966 | NA | January, February, March, April, May, June, July, August, September, October, November, December | Winter,  Spring,  Summer,  Autumn | NA | Manual capture,  Larvae capture,  Malaise trap | NA | Rageau et al. (1967) |
|  | Vosges | 1834-1926 | NA | April, May, June, July, August, September | Spring,  Summer,  Autumn | NA | NA | NA | Séguy (1926) |
|  | Bouches-du-Rhône | 1944 | NA | July | Summer | NA | NA | NA | GBIF (2023) |
| *Atylotus*  *quadrifarius* | Bouches-du-Rhône | 2011 | 1 | June | Summer | 10 | N’zi trap | NA | Baldacchino et al. (2013a) |
|  | Bouches-du-Rhône | 2012 | 1 | June, July | Summer | 10 | N’zi trap (with and without odor bait) | NA | Baldacchino et al. (2013b) |
|  | Bouches-du-Rhône | 1972-1973 | 6 | May, June, July, August, September | Spring,  Summer | NA | Manitoba trap,  Net capture,  Larvae capture | NA | Raymond (1978) |
| *Atylotus*  *rusticus* | Maine-et-Loire | 2008 | 1 | July | Summer | 71 | Vavoua trap | NA | Grimeau (2012) |
|  | Doubs, Jura, Loire, Vendée, Meuse | 2010, 2014, 2018-2019 | NA | June, July | Summer | NA | NA | NA | INPN (2023) |
|  | Hautes-Pyrénées | 1955-1968 | NA | June, July, August | Summer | NA | NA | NA | Leclercq (1970) |
|  | Alpes-de-Haute-Provence | 1957-1969 | NA | June, July, August | Summer | 250-3400 | NA | NA | Leclercq (1971) |
|  | Hérault, Gard, Bouches-du-Rhône | 1953-1966 | NA | January, February, March, April, May, June, July, August, September, October, November, December | Winter,  Spring,  Summer,  Autumn | NA | Manual capture,  Larvae capture,  Malaise trap | NA | Rageau et al. (1967) |
|  | Hautes-Alpes | 1972-1973, 1978 | 3 | July, August | Summer | NA | Manitoba trap,  Manual capture | NA | Raymond (1979) |
|  | All France | NA | NA | NA | NA | NA | NA | NA | Séguy (1924) |
|  | Ardennes, Meurthe-et-Moselle, Haute-Saône, Aube, Côte-d'Or, Saône-Et-Loire, Var | 1834-1926 | NA | April, May, June, July, August, September | Spring,  Summer,  Autumn | NA | NA | NA | Séguy (1926) |
|  | Loire, Meuse, Vendée, Jura, Doubs | 2010, 2014, 2018-2019 | NA | June, July | Summer | NA | NA | NA | GBIF (2023) |
| *Chrysops*  *caecutiens* | Pyrénées-Orientales | 2011 | 5 | May, June, July, August, September | Spring,  Summer | 850-2300,  1450-2700 | N’zi and  Vavoua traps | NA | Baldacchino et al. (2014b) |
|  | Ille-et-Vilaine, Loire-Atlantique | 1989-1990 | NA | NA | Summer | 164, 20 | Net capture,  Manitoba trap,  Car-net | NA | Le Goff et al. (1993) |
|  | Bouches-du-Rhône | 2012 | 1 | June, July | Summer | 10 | N’zi trap (with and without odor bait) | NA | Baldacchino et al. (2013b) |
|  | Ain, Aisne, Alpes-de-Haute-Provence, Alpes-Maritimes, Ardèche, Ariège, Aube, Calvados, Cantal, Cher, Corrèze, Creuse, Dordogne, Doubs, Drôme, Essonne, Eure-et-Loir, Finistère, Gers, Gironde, Corse, Ille-et-Vilaine, Indre, Isère, Loire, Loiret, Loir-et-Cher, Lot, Lot-et-Garonne, Lozère, Manche, Morbihan, Oise, Orne, Pas-de-Calais, Puy-de-Dôme, Pyrénées-Orientales, Savoie, Seine-et-Marne, Seine-Maritime, Somme, Val-d'Oise, Var, Vosges, Yonne, Yvelines | 1758, 1989, 1993, 1998, 2001, 2004-2022 | NA | January, February, April, May, June, July, August, December | Winter,  Spring,  Summer | NA | NA | NA | INPN (2023) |
|  | Ille-et-Vilaine | 1989 | 1 | July | Summer | 71-258 | Net capture | *Spiroplasma*  sp. | Le Goff et al. (1991) |
|  | Pyrénées-Atlantiques, Hautes-Pyrénées, Ariège, Pyrénées-Orientales | 1955-1968 | NA | June, July, August | Summer | NA | NA | NA | Leclercq (1970) |
|  | Alpes-de-Haute-Provence | 1957-1969 | NA | June, July, August | Summer | 250-3400 | NA | NA | Leclercq (1971) |
|  | Ain, Allier, Ardèche, Aveyron, Charente-Maritime, Corse, Ille-et-Vilaine, Lozère, Pyrénées-Orientales, Bas-Rhin, Haut-Rhin, Haute-Savoie | 1953-1971 | NA | NA | NA | NA | NA | NA | Pernot-Visentin et al. (1974) |
|  | Hérault, Gard, Bouches-du-Rhône | 1953-1966 | NA | January, February, March, April, May, June, July, August, September, October, November, December | Winter,  Spring,  Summer,  Autumn | NA | Manual capture,  Larvae capture,  Malaise trap | NA | Rageau et al. (1967) |
|  | Bouches-du-Rhône | 1972-1973 | 6 | May, June, July, August, September | Spring,  Summer | NA | Manitoba trap,  Net capture,  Larvae capture | NA | Raymond (1978) |
|  | Hautes-Alpes | 1972-1973, 1978 | 3 | July, August | Summer | NA | Manitoba trap,  Manual capture | NA | Raymond (1979) |
|  | All France | NA | NA | NA | NA | NA | NA | NA | Séguy (1924) |
|  | All France | 1834-1926 | NA | April, May, June, July, August, September | Spring,  Summer,  Autumn | NA | NA | NA | Séguy (1926) |
|  | Ain, Aisne, Alpes-de-Haute-Provence, Alpes-Maritimes, Ardèche, Ardennes, Ariège, Aube, Aveyron, Bas-Rhin, Calvados, Cantal, Cher, Corrèze, Corse, Côtes-d’Armor, Côte-d'Or, Creuse, Dordogne, Doubs, Drôme, Essonne, Eure-et-Loir, Finistère, Gard, Gers, Gironde, Haute-Garonne, Haute-Vienne, Haut-Rhin, Ille-et-Vilaine, Indre, Indre-et-Loire, Isère, Loire, Loiret, Loir-et-Cher, Lot, Lot-et-Garonne, Lozère, Maine-et-Loire, Manche, Meuse, Morbihan, Moselle, Nord, Oise, Orne, Puy-de-Dôme, Pyrénées-Orientales, Saône-et-Loire, Savoie, Seine-et-Marne, Seine-Maritime, Somme, Val-d'Oise, Var, Vaucluse, Vosges, Yonne, Yvelines | 1758, 1989, 1993, 1998, 2001, 2004-2022 | NA | January, February, April, May, June, July, August, September, December | Winter,  Spring,  Summer | NA | NA | NA | GBIF (2023) |
| *Chrysops*  *flavipes* | Bouches-du-Rhône | 2011 | NA | June | Summer | NA | NA | NA | INPN (2023) |
|  | Hérault, Gard, Bouches-du-Rhône | 1953-1966 | NA | January, February, March, April, May, June, July, August, September, October, November, December | Winter,  Spring,  Summer,  Autumn | NA | Manual capture,  Larvae capture,  Malaise trap | NA | Rageau et al. (1967) |
|  | Bouches-du-Rhône | 1972-1973 | 6 | May, June, July, August, September | Spring,  Summer | NA | Manitoba trap,  Net capture,  Larvae capture | NA | Raymond (1978) |
|  | Meurthe-et-Moselle, Landes, Var, Ardennes, Seine-Maritime, Yvelines, Val-d'Oise, Hauts-de-Seine, Essonne, Seine-Saint-Denis, Val-de-Marne | 1834-1926 | NA | April, May, June, July, August, September | Spring,  Summer,  Autumn | NA | NA | NA | Séguy (1926) |
|  | Vaucluse | NA | NA | NA | NA | NA | NA | NA | GBIF (2023) |
| *Chrysops*  *italicus* | Côtes-d'Armor, Pyrénées-Orientales | 2005, 2014 | NA | January, July, August | Winter,  Summer | NA | NA | NA | INPN (2023) |
|  | Pyrénées-Orientales | 1955-1968 | NA | June, July, August | Summer | NA | NA | NA | Leclercq (1970) |
|  | Meurthe-et-Moselle, Allier, Var, Pyrénées-Orientales, Aude, Landes, Corse | 1834-1926 | NA | April, May, June, July, August, September | Spring,  Summer,  Autumn | NA | NA | NA | Séguy (1926) |
|  | Pyrénées-Orientales, Corse, Haut-Rhin, Côtes-d'Armor | 2005, 2014-2015, 2021 | NA | January, July, August | Winter,  Summer | NA | NA | NA | GBIF (2023) |
| *Chrysops*  *parallelogrammus* | Cher | 2013 | NA | May | Spring | NA | NA | NA | INPN (2023) |
|  | Maine-et-Loire | 1834-1926 | NA | April, May, June, July, August, September | Spring,  Summer,  Autumn | NA | NA | NA | Séguy (1926) |
|  | Cher | 2013 | NA | May | Spring | NA | NA | NA | GBIF (2023) |
| *Chrysops*  *pictus* | Ille-et-Vilaine, Loire-Atlantique | 1989-1990 | NA | NA | Summer | 164, 20 | Net capture,  Manitoba trap,  Car-net | NA | Le Goff et al. (1993) |
|  | Pyrénées-Orientales, Ariège, Pyrénées-Atlantiques | 1955-1968 | NA | June, July, August | Summer | NA | NA | NA | Leclercq (1970) |
|  | Allier, Ariège, Corse, Doubs, Ille-et-Vilaine, Mayenne, Loire-Atlantique, Pas-de-Calais, Pyrénées-Orientales, Bas-Rhin, Haute-Savoie, Seine-Maritime | 1953-1971 | NA | NA | NA | NA | NA | NA | Pernot-Visentin et al. (1974) |
|  | Hérault, Gard, Bouches-du-Rhône | 1953-1966 | NA | January, February, March, April, May, June, July, August, September, October, November, December | Winter,  Spring,  Summer,  Autumn | NA | Manual capture,  Larvae capture,  Malaise trap | NA | Rageau et al. (1967) |
|  | Oise, Meurthe-et-Moselle, Seine-et-Marne, Yvelines, Val-d'Oise, Hauts-de-Seine, Essonne, Seine-Saint-Denis, Val-de-Marne, Orne, Côtes-d’Armor, Haute-Savoie | 1834-1926 | NA | April, May, June, July, August, September | Spring,  Summer,  Autumn | NA | NA | NA | Séguy (1926) |
| *Chrysops*  *relictus* | Pyrénées-Orientales | 2011 | 5 | May, June, July, August, September | Spring,  Summer | 850-2300,  1450-2700 | N’zi and  Vavoua trap | NA | Baldacchino et al. (2014b) |
|  | Ain, Ardennes, Aveyron, Bas-Rhin, Côtes-d'Armor, Cher, Doubs, Finistère, Haute-Savoie, Hautes-Pyrénées, Isère, Jura, Loire-Atlantique, Loir-et-Cher, Meurthe-et-Moselle, Morbihan, Moselle, Nièvre, Nord, Pas-de-Calais, Pyrénées-Orientales, Saône-et-Loire, Seine-et-Marne, Somme, Val-d'Oise, Yonne | 1983, 2000-2001, 2004-2010, 2012-2022 | NA | January, May, June, July, August, September | Winter,  Spring,  Summer | NA | NA | NA | INPN (2023) |
|  | Hautes-Pyrénées, Ariège, Pyrénées-Orientales | 1955-1968 | NA | June, July, August | Summer | NA | NA | NA | Leclercq (1970) |
|  | Doubs, Nord, Pas-de-Calais, Pyrénées-Orientales, Bas-Rhin | 1953-1971 | NA | NA | NA | NA | NA | NA | Pernot-Visentin et al. (1974) |
|  | Bouches-du-Rhône | 1972-1973 | 6 | May, June, July, August, September | Spring,  Summer | NA | Manitoba trap,  Net capture,  Larvae capture | NA | Raymond (1978) |
|  | Ain, Ardennes, Aveyron, Bas-Rhin, Cher, Côtes-d'Armor, Doubs, Finistère, Hautes-Pyrénées, Haut-Rhin, Isère, Jura, Loire-Atlantique, Loir-et-Cher, Manche, Meurthe-et-Moselle, Morbihan, Moselle, Nièvre, Nord, Pas-de-Calais, Pyrénées-Orientales, Saône-et-Loire, Savoie, Seine-et-Marne, Somme, Val-d'Oise | 2000-2001, 2004-2022 | NA | January, May, June, July, August, September | Winter,  Spring,  Summer | NA | NA | NA | GBIF (2023) |
| *Chrysops*  *rufipes* | Yvelines, Val-d'Oise, Hauts-de-Seine, Essonne, Seine-Saint-Denis, Val-de-Marne, Eure, Orne, Maine-et-Loire, Landes, Hautes-Pyrénées | 1834-1926 | NA | April, May, June, July, August, September | Spring,  Summer,  Autumn | NA | NA | NA | Séguy (1926) |
| *Chrysops*  *sepulcralis* | Pyrénées-Orientales | 2011 | 5 | May, June, July, August, September | Spring,  Summer | 850-2300,  1450-2700 | N’zi and  Vavoua trap | NA | Baldacchino et al. (2014b) |
|  | Pyrénées-Orientales | 1953-1971 | NA | NA | NA | NA | NA | NA | Pernot-Visentin et al. (1974) |
| *Chrysops*  *viduatus* | Aisne, Ardennes, Bouches-du-Rhône, Côtes-d'Armor, Cher, Dordogne, Doubs, Finistère, Gironde, Corse, Haute-Garonne, Haute-Saône, Haute-Vienne, Indre, Landes, Loire, Loir-et-Cher, Lozère, Morbihan, Moselle, Nièvre, Nord, Oise, Pas-de-Calais, Pyrénées-Orientales, Savoie, Seine-et-Marne, Somme, Vosges | 1995, 2000-2002, 2004, 2006, 2008-2010, 2012-2022 | NA | January, March, May, June, July, August, September | Winter,  Spring,  Summer | NA | NA | NA | INPN (2023) |
|  | Ille-et-Vilaine | 1989 | 1 | July | Summer | 71-258 | Net capture | *Spiroplasma*  sp. | Le Goff et al. (1991) |
|  | Bouches-du-Rhône | 1972-1973 | 6 | May, June, July, August, September | Spring,  Summer | NA | Manitoba trap,  Net capture,  Larvae capture | NA | Raymond (1978) |
|  | Aisne, Allier, Ardennes, Bouches-du-Rhône, Cher, Corse, Côtes-d'Armor, Dordogne, Doubs, Finistère, Gironde, Haute-Garonne, Haute-Saône, Haute-Vienne, Haut-Rhin, Indre, Landes, Loire, Loiret, Loir-et-Cher, Lozère, Morbihan, Moselle, Nièvre, Nord, Oise, Pas-de-Calais, Pyrénées-Orientales, Saône-et-Loire, Savoie, Seine-et-Marne, Somme, Vosges | 1951, 1995, 2001-2002, 2004, 2006, 2008-2010, 2012-2022 | NA | January, March, May, June, July, August, September | Winter,  Spring,  Summer | NA | NA | NA | GBIF (2023) |
| *Dasyrhamphis*  *anthracinus* | Jura, Corse | 2013, 2021 | NA | May, July | Spring,  Summer | NA | NA | NA | INPN (2023) |
|  | Pyrénées-Orientales | 1955-1968 | NA | June, July, August | Summer | NA | NA | NA | Leclercq (1970) |
|  | Alpes-Maritimes, Var, Pyrénées-Orientales | 1834-1926 | NA | April, May, June, July, August, September | Spring,  Summer,  Autumn | NA | NA | NA | Séguy (1926) |
| *Dasyrhamphis*  *ater* | Pyrénées-Orientales | 2011 | 0.2 | July | Summer | 1450-2700 | N’zi trap | NA | Baldacchino et al. (2014c) |
|  | Ain, Aisne, Alpes-de-Haute-Provence, Alpes-Maritimes, Ariège, Corrèze, Drôme, Gard, Hérault, Isère, Loire, Lot-et-Garonne, Lozère, Pyrénées-Atlantiques, Pyrénées-Orientales, Tarn, Var | 2005, 2008-2010, 2013, 2015-2017, 2019-2021 | NA | May, June, July | Spring,  Summer | NA | NA | NA | INPN (2023) |
|  | Hautes-Pyrénées, Ariège, Pyrénées-Orientales | 1955-1968 | NA | June, July, August | Summer | NA | NA | NA | Leclercq (1970) |
|  | Alpes-de-Haute-Provence | 1957-1969 | NA | June, July, August | Summer | 250-3400 | NA | NA | Leclercq (1971) |
|  | Hérault, Gard, Bouches-du-Rhône | 1953-1966 | NA | January, February, March, April, May, June, July, August, September, October, November, December | Winter,  Spring,  Summer,  Autumn | NA | Manual capture,  Larvae capture,  Malaise trap | NA | Rageau et al. (1967) |
|  | Bouches-du-Rhône | 1972-1973 | 6 | May, June, July, August, September | Spring,  Summer | NA | Manitoba trap,  Net capture,  Larvae capture | NA | Raymond (1978) |
|  | Hautes-Alpes | 1972-1973, 1978 | 3 | July, August | Summer | NA | Manitoba trap,  Manual capture | NA | Raymond (1979) |
|  | All France | 1834-1926 | NA | April, May, June, July, August, September | Spring,  Summer,  Autumn | NA | NA | NA | Séguy (1926) |
|  | Ain, Aisne, Alpes-de-Haute-Provence, Alpes-Maritimes, Ariège, Cantal, Corrèze, Drôme, Gard, Haute-Garonne, Hautes-Alpes, Hérault, Isère, Loire, Lot-et-Garonne, Lozère, Pyrénées-Atlantiques, Pyrénées-Orientales, Tarn, Var | 2005, 2008-2010, 2013-2017, 2019-2021 | NA | May, June, July | Spring,  Summer | NA | NA | NA | GBIF (2023) |
| *Haematopota*  *bigoti* | Doubs, Dordogne, Gironde, Bouches-du-Rhône | 2008, 2010-2011, 2014 | NA | May, July, August, October | Spring,  Summer,  Autumn | NA | NA | NA | INPN (2023) |
|  | Pyrénées-Orientales | 1955-1968 | NA | June, July, August | Summer | NA | NA | NA | Leclercq (1970) |
|  | Hérault, Gard, Bouches-du-Rhône | 1953-1966 | NA | January, February, March, April, May, June, July, August, September, October, November, December | Winter,  Spring,  Summer,  Autumn | NA | Manual capture,  Larvae capture,  Malaise trap | NA | Rageau et al. (1967) |
|  | Bouches-du-Rhône | 1972-1973 | 6 | May, June, July, August, September | Spring,  Summer | NA | Manitoba trap,  Net capture,  Larvae capture | NA | Raymond (1978) |
|  | Côtes-d’Armor, Gard, Hérault | 1834-1926 | NA | April, May, June, July, August, September | Spring,  Summer,  Autumn | NA | NA | NA | Séguy (1926) |
|  | Bouches-du-Rhône, Gironde, Dordogne, Doubs | 2008, 2010-2011, 2014 | NA | May, July, August, October | Spring,  Summer,  Autumn | NA | NA | NA | GBIF (2023) |
| *Haematopota*  *crassicornis* | Maine-et-Loire | 2008 | 1 | July | Summer | 71 | Vavoua trap | NA | Grimeau (2012) |
|  | Doubs, Essonne | 1999, 2014 | NA | January, July | Winter,  Summer | NA | NA | NA | INPN (2023) |
|  | Hautes-Pyrénées | 1955-1968 | NA | June, July, August | Summer | NA | NA | NA | Leclercq (1970) |
|  | Alpes-de-Haute-Provence | 1957-1969 | NA | June, July, August | Summer | 250-3400 | NA | NA | Leclercq (1971) |
|  | Loire-Atlantique, Morbihan, Nord, Pyrénées-Orientales, Haute-Savoie | 1953-1971 | NA | NA | NA | NA | NA | NA | Pernot-Visentin et al. (1974) |
|  | Hérault, Gard, Bouches-du-Rhône | 1953-1966 | NA | January, February, March, April, May, June, July, August, September, October, November, December | Winter,  Spring,  Summer,  Autumn | NA | Manual capture,  Larvae capture,  Malaise trap | NA | Rageau et al. (1967) |
|  | Seine-et-Marne, Hautes-Alpes | 1834-1926 | NA | April, May, June, July, August, September | Spring,  Summer,  Autumn | NA | NA | NA | Séguy (1926) |
|  | Essonne, Doubs | 1999, 2014 | NA | January, July | Winter,  Summer | NA | NA | NA | GBIF (2023) |
| *Haematopota*  *grandis* | Gironde, Indre, Dordogne | 2008, 2010-2011 | NA | August, September, October | Summer,  Autumn | NA | NA | NA | INPN (2023) |
|  | Pyrénées-Orientales | 1953-1971 | NA | NA | NA | NA | NA | NA | Pernot-Visentin et al. (1974) |
|  | Hérault, Gard, Bouches-du-Rhône | 1953-1966 | NA | January, February, March, April, May, June, July, August, September, October, November, December | Winter,  Spring,  Summer,  Autumn | NA | Manual capture,  Larvae capture,  Malaise trap | NA | Rageau et al. (1967) |
|  | Bouches-du-Rhône | 1972, 1973 | 6 | May, June, July, August, September | Spring,  Summer | NA | Manitoba trap,  Net capture,  Larvae capture | NA | Raymond (1978) |
|  | Seine-et-Marne, Seine-Maritime, Côte-d'Or, Maine-et-Loire | 1834-1926 | NA | April, May, June, July, August, September | Spring,  Summer,  Autumn | NA | NA | NA | Séguy (1926) |
|  | Gironde, Dordogne, Indre | 2008, 2010-2011 | NA | August, September, October | Summer,  Autumn | NA | NA | NA | GBIF (2023) |
| *Haematopota*  *italica* | Alpes-de-Haute-Provence, Ariège, Doubs, Manche, Pyrénées-Orientales | 1961, 2001, 2005, 2007, 2009, 2011 | NA | January, July, August | Winter,  Summer | NA | NA | NA | INPN (2023) |
|  | Ariège, Pyrénées-Orientales | 1955-1968 | NA | June, July, August | Summer | NA | NA | NA | Leclercq (1970) |
|  | Alpes-de-Haute-Provence | 1957-1969 | NA | June, July, August | Summer | 250-3400 | NA | NA | Leclercq (1971) |
|  | Alpes-Maritimes, Alpes-de-Haute-Provence, Bas-Rhin, Somme | 1953-1971 | NA | NA | NA | NA | NA | NA | Pernot-Visentin et al. (1974) |
|  | Bouches-du-Rhône | 1972-1973 | 6 | May, June, July, August, September | Spring,  Summer | NA | Manitoba trap,  Net capture,  Larvae capture | NA | Raymond (1978) |
|  | Hautes-Alpes | 1972-1973, 1978 | 3 | July, August | Summer | NA | Manitoba trap,  Manual capture | NA | Raymond (1979) |
|  | All France | 1834-1926 | NA | April, May, June, July, August, September | Spring,  Summer,  Autumn | NA | NA | NA | Séguy (1926) |
|  | Pyrénées-Orientales, Alpes-de-Haute-Provence, Doubs, Ariège, Nord, Manche, Haute-Saône | 1961, 2001, 2005, 2007, 2009, 2011 | NA | January, July, August | Winter,  Summer | NA | NA | NA | GBIF (2023) |
| *Haematopota*  *lambi* | Bouches-du-Rhône | 1972-1973 | 6 | May, June, July, August, September | Spring,  Summer | NA | Manitoba trap,  Net capture,  Larvae capture | NA | Raymond (1978) |
|  | Bouches-du-Rhône | 1980 | NA | July | Summer | NA | NA | NA | GBIF (2023) |
| *Haematopota*  *ocelligera* | Hautes-Alpes, Gironde | 1968, 2008 | NA | August, September | Summer | NA | NA | NA | INPN (2023) |
|  | Hautes-Pyrénées, Ariège, Pyrénées-Orientales | 1955-1968 | NA | June, July, August | Summer | NA | NA | NA | Leclercq (1970) |
|  | Bouches-du-Rhône | 1972-1973 | 6 | May, June, July, August, September | Spring,  Summer | NA | Manitoba trap,  Net capture,  Larvae capture | NA | Raymond (1978) |
|  | Hautes-Alpes | 1972-1973, 1978 | 3 | July, August | Summer | NA | Manitoba trap,  Manual capture | NA | Raymond (1979) |
|  | Hautes-Alpes, Gironde | 1968, 2008 | NA | August, September | Summer | NA | NA | NA | GBIF (2023) |
| *Haematopota*  *pluvialis* | Pyrénées-Orientales | 2012 | 2 | July, August | Summer | NA | N’zi trap | NA | Baldacchino et al. (2014a) |
|  | Pyrénées-Orientales | 2011 | 0.2 | July | Summer | 1450-2700 | N’zi trap | NA | Baldacchino et al. (2014c) |
|  | Pyrénées-Orientales | 2011 | 5 | May, June, July, August, September | Spring,  Summer | 850-2300,  1450-2700 | N’zi and  Vavoua traps | NA | Baldacchino et al. (2014b) |
|  | Loire-Atlantique | 2012 | 5 | April, May, June, July, August | Summer | NA | N’zi trap | NA | Lantuejoul (2015) |
|  | Loire-Atlantique | 2013-2014 | NA | NA | NA | NA | NA | NA | Jacquiet et al. (2015) |
|  | Ille-et-Vilaine, Loire-Atlantique | 1989-1990 | NA | NA | Summer | 164, 20 | Net Capture,  Manitoba trap,  Car-net | NA | Le Goff et al. (1993) |
|  | Indre-et-Loire | 1991 | 1 | August | Summer | NA | NA | *Spiroplasma*  *turonicum* | Hélias et al. (1998) |
|  | Aisne, Alpes-de-Haute-Provence, Ariège, Bas-Rhin, Bouches-du-Rhône, Calvados, Charente-Maritime, Cher, Dordogne, Doubs, Eure, Gironde, Hautes-Pyrénées, Ille-et-Vilaine, Indre, Indre-et-Loire, Isère, Landes, Loire-Atlantique, Loiret, Loir-et-Cher, Lot-et-Garonne, Lozère, Maine-et-Loire, Manche, Moselle, Oise, Orne, Pas-de-Calais, Pyrénées-Atlantiques, Pyrénées-Orientales, Seine-et-Marne, Seine-Maritime, Somme, Val-d'Oise, Yonne | 1946, 1993-1996, 1998, 2000-2002, 2004-2022 | NA | January, February, May, June, July, August, September, October, December | Winter,  Spring,  Summer,  Autumn | NA | NA | NA | INPN (2023) |
|  | Haute-Garonne, Pyrénées-Atlantiques, Hautes-Pyrénées, Ariège, Pyrénées-Orientales | 1955-1968 | NA | June, July, August | Summer | NA | NA | NA | Leclercq (1970) |
|  | Alpes-de-Haute-Provence | 1957-1969 | NA | June, July, August | Summer | 250-3400 | NA | NA | Leclercq (1971) |
|  | Ain, Allier, Ardennes, Ariège, Aisne, Doubs, Ille-et-Vilaine, Isère, Loire-Atlantique, Haute-Loire, Maine-et-Loire, Meuse, Morbihan, Nord, Pyrénées-Atlantiques, Bas-Rhin, Rhône, Haute-Savoie, Somme | 1953-1971 | NA | NA | NA | NA | NA | NA | Pernot-Visentin et al. (1974) |
|  | Hérault, Gard, Bouches-du-Rhône | 1953-1966 | NA | January, February, March, April, May, June, July, August, September, October, November, December | Winter,  Spring,  Summer,  Autumn | NA | Manual capture,  Larvae capture,  Malaise trap | NA | Rageau et al. (1967) |
|  | Bouches-du-Rhône | 1972-1973 | 6 | May, June, July, August, September | Spring,  Summer | NA | Manitoba trap,  Net capture,  Larvae capture | NA | Raymond (1978) |
|  | Hautes-Alpes | 1972-1973, 1978 | 3 | July, August | Summer | NA | Manitoba trap,  Manual capture | NA | Raymond (1979) |
|  | All France | NA | NA | NA | NA | NA | NA | NA | Séguy (1924) |
|  | All France | 1834-1926 | NA | April, May, June, July, August, September | Spring,  Summer,  Autumn | NA | NA | NA | Séguy (1926) |
|  | Aisne, Alpes-de-Haute-Provence, Alpes-Maritimes, Ariège, Bas-Rhin, Bouches-du-Rhône, Calvados, Charente-Maritime, Cher, Dordogne, Doubs, Eure, Gironde, Haute-Loire, Hautes-Pyrénées, Haut-Rhin, Ille-et-Vilaine, Indre, Indre-et-Loire, Isère, Landes, Loire-Atlantique, Loiret, Loir-et-Cher, Lot-et-Garonne, Lozère, Maine-et-Loire, Manche, Meurthe-et-Moselle, Moselle, Nord, Oise, Orne, Pas-de-Calais, Pyrénées-Atlantiques, Pyrénées-Orientales, Seine-et-Marne, Seine-Maritime, Somme, Val-d'Oise, Yonne | 1946, 1993-1996, 1998, 2000-2002, 2004-2022 | NA | January, February, May, June, July, August, September, October, December | Winter,  Spring,  Summer,  Autumn | NA | NA | NA | GBIF (2023) |
| *Haematopota*  *scutellata* | Pyrénées-Orientales | 2011 | 0,2 | July | Summer | 1450-2700 | N’zi trap | NA | Baldacchino et al. (2014c) |
|  | Pyrénées-Orientales | 2011 | 5 | May, June, July, August, September | Spring,  Summer | 850-2300,  1450-2700 | N’zi and  Vavoua traps | NA | Baldacchino et al. (2014b) |
| *Heptatoma*  *pellucens* | Ille-et-Vilaine, Loire-Atlantique | 1989-1990 | NA | NA | Summer | 164, 20 | Net Capture,  Manitoba trap,  Car-net | NA | Le Goff et al. (1993) |
|  | Aisne, Alpes-de-Haute-Provence, Ardennes, Dordogne, Essonne, Indre, Indre-et-Loire, Jura, Loiret, Nord, Oise, Pas-de-Calais, Seine-et-Marne, Seine-Maritime, Somme, Val-de-Marne | 1976, 2005, 2009, 2012, 2014-2021 | NA | May, June, July, August, September | Spring,  Summer | NA | NA | NA | INPN (2023) |
|  | Ille-et-Vilaine | 1989 | 1 | July | Summer | 71-258 | Capture au filet | *Spiroplasma*  sp. | Le Goff et al. (1991) |
|  | Bouches-du-Rhône | 1972-1973 | 6 | May, June, July, August, September | Spring,  Summer | NA | Manitoba trap,  Net capture,  Larvae capture | NA | Raymond (1978) |
|  | Hautes-Alpes | 1972-1973, 1978 | 3 | July, August | Summer | NA | Manitoba trap,  Manual capture | NA | Raymond (1979) |
|  | Aisne, Alpes-de-Haute-Provence, Ardennes, Dordogne, Essonne, Haute-Marne, Haut-Rhin, Indre, Indre-et-Loire, Jura, Loiret, Nord, Oise, Pas-de-Calais, Seine-et-Marne, Seine-Maritime, Somme, Val-de-Marne | 1976, 2004, 2009, 2012, 2014-2022 | NA | May, June, July, August, September | Spring,  Summer | NA | NA | NA | GBIF (2023) |
| *Hybomitra*  *acuminata* | Hérault, Gard, Bouches-du-Rhône | 1953-1966 | NA | January, February, March, April, May, June, July, August, September, October, November, December | Winter,  Spring,  Summer,  Autumn | NA | Manual capture,  Larvae capture,  Malaise trap | NA | Rageau et al. (1967) |
|  | Bouches-du-Rhône | 1972-1973 | 6 | May, June, July, August, September | Spring,  Summer | NA | Manitoba trap,  Net capture,  Larvae capture | NA | Raymond (1978) |
| *Hybomitra*  *aterrima* | Alpes-Maritimes, Ariège, Isère, Doubs, Jura | 1946, 2010-2011, 2013-2014, 2017 | NA | June, July, August | Summer | NA | NA | NA | INPN (2023) |
|  | Pyrénées-Orientales, Ariège | 1955-1968 | NA | June, July, August | Summer | NA | NA | NA | Leclercq (1970) |
|  | Alpes-de-Haute-Provence | 1957-1969 | NA | June, July, August | Summer | 250-3400 | NA | NA | Leclercq (1971) |
|  | Pyrénées-Orientales | 1953-1971 | NA | NA | NA | NA | NA | NA | Pernot-Visentin et al. (1974) |
|  | Puy-de-Dôme, Hautes-Alpes, Savoie, Isère, Hautes-Pyrénées | 1834-1926 | NA | April, May, June, July, August, September | Spring,  Summer,  Autumn | NA | NA | NA | Séguy (1926) |
|  | Ariège, Alpes-Maritimes, Isère, Jura, Doubs | 1946, 2010-2011, 2013-2014, 2017 | NA | June, July, August | Summer | NA | NA | NA | GBIF (2023) |
| *Hybomitra*  *auripila* | Pyrénées-Orientales | 2012 | 2 | July, August | Summer | NA | N’zi trap | NA | Baldacchino et al. (2014a) |
|  | Pyrénées-Orientales | 2011 | 0.2 | July | Summer | 1450-2700 | N’zi trap | NA | Baldacchino et al. (2014c) |
|  | Pyrénées-Orientales | 2011 | 5 | May, June, July, August, September | Spring,  Summer | 850-2300,  1450-2700 | N’zi and  Vavoua traps | NA | Baldacchino et al. (2014b) |
|  | Pyrénées-Orientales | 2013-2014 | NA | NA | NA | NA | NA | NA | Jacquiet et al. (2015) |
|  | Pyrénées-Atlantiques, Ariège, Alpes-Maritimes, Alpes-de-Haute-Provence, Doubs | 1946, 2010-2011, 2018-2019 | NA | June, July | Summer | NA | NA | NA | INPN (2023) |
|  | Hautes-Pyrénées, Ariège, Pyrénées-Orientales | 1955-1968 | NA | June, July, August | Summer | NA | NA | NA | Leclercq (1970) |
|  | Alpes-de-Haute-Provence | 1957-1969 | NA | June, July, August | Summer | 250-3400 | NA | NA | Leclercq (1971) |
|  | Ain, Pyrénées-Orientales, Haute-Savoie | 1953-1971 | NA | NA | NA | NA | NA | NA | Pernot-Visentin et al. (1974) |
|  | Hautes-Alpes | 1972-1973, 1978 | 3 | July, August | Summer | NA | Manitoba trap,  Manual capture | NA | Raymond (1979) |
|  | Isère, Pyrénées-Orientales, Hautes-Pyrénées | 1834-1926 | NA | April, May, June, July, August, September | Spring,  Summer,  Autumn | NA | NA | NA | Séguy (1926) |
|  | Ariège, Pyrénées-Atlantiques, Alpes-Maritimes, Alpes-de-Haute-Provence, Doubs | 1946, 2010-2011, 2018-2019 | NA | June, July | Summer | NA | NA | NA | GBIF (2023) |
| *Hybomitra*  *bimaculata* | Loire-Atlantique | 2012 | 5 | April, May, June, July, August | Summer | NA | N’zi trap | NA | Lantuejoul (2015) |
|  | Loire-Atlantique | 2013-2014 | NA | NA | NA | NA | NA | NA | Jacquiet et al. (2015) |
|  | Ille-et-Vilaine, Loire-Atlantique | 1989-1990 | NA | NA | Summer | 164, 20 | Net Capture,  Manitoba trap,  Car-net | NA | Le Goff et al. (1993) |
|  | Bouches-du-Rhône | 2012 | 1 | June, July | Summer | 10 | N’zi trap (with and without odor bait) | NA | Baldacchino et al. (2013b) |
|  | Aisne, Alpes-Maritimes, Bouches-du-Rhône, Cher, Doubs, Indre, Loire-Atlantique, Maine-et-Loire, Manche, Oise, Orne, Pas-de-Calais, Pyrénées-Orientales, Seine-et-Marne, Vosges | 1913, 1934, 1980, 2001, 2003, 2005, 2007-2008, 2010-2011, 2013-2017 | NA | January, May, June, July, December | Winter,  Spring,  Summer | NA | NA | NA | INPN (2023) |
|  | Ille-et-Vilaine | 1989 | 1 | July | Summer | 71-258 | Net capture | *Spiroplasma*  sp. | Le Goff et al. (1991) |
|  | Bouches-du-Rhône | 1972-1973 | 6 | May, June, July, August, September | Spring,  Summer | NA | Manitoba trap,  Net capture,  Larvae capture | NA | Raymond (1978) |
|  | Ardennes, Meurthe-et-Moselle, Eure-et-Loir, Essonne, Hauts-de-Seine, Val-d'Oise | 1834-1926 | NA | April, May, June, July, August, September | Spring,  Summer,  Autumn | NA | NA | NA | Séguy (1926) |
|  | Aisne, Alpes-Maritimes, Bouches-du-Rhône, Cher, Doubs, Indre, Loire-Atlantique, Maine-et-Loire, Manche, Oise, Orne, Pas-de-Calais, Pyrénées-Orientales, Seine-et-Marne, Vosges | 1913, 1934, 1980, 2001, 2003, 2005, 2007-2008, 2010-2011, 2013, 2015-2017 | NA | January, May, June, July, December | Winter,  Spring,  Summer | NA | NA | NA | GBIF (2023) |
| *Hybomitra*  *borealis* | Hautes-Pyrénées | 1955-1968 | NA | June, July, August | Summer | NA | NA | NA | Leclercq (1970) |
|  | Ardennes, Hautes-Pyrénées | 1834-1926 | NA | April, May, June, July, August, September | Spring,  Summer,  Autumn | NA | NA | NA | Séguy (1926) |
| *Hybomitra*  *caucasica* | Pyrénées-Orientales | 2011 | 0.2 | July | Summer | 1450-2700 | N’zi trap | NA | Baldacchino et al. (2014c) |
|  | Pyrénées-Orientales | 2011 | 5 | May, June, July, August, September | Spring,  Summer | 850-2300,  1450-2700 | N’zi and  Vavoua traps | NA | Baldacchino et al. (2014b) |
|  | Pyrénées-Orientales | 2013-2014 | NA | NA | NA | NA | NA | NA | Jacquiet et al. (2015) |
|  | Hautes-Alpes | 1972-1973, 1978 | 3 | July, August | Summer | NA | Manitoba trap,  Manual capture | NA | Raymond (1979) |
| *Hybomitra*  *ciureai* | Bouches-du-Rhône | 2011 | 1 | June | Summer | 10 | N’zi trap | NA | Baldacchino et al. (2013a) |
|  | Bouches-du-Rhône | 2012 | 1 | June, July | Summer | 10 | N’zi trap (with and without odor bait) | NA | Baldacchino et al. (2013b) |
|  | Aisne, Bouches-du-Rhône, Doubs, Oise, Pyrénées-Atlantiques, Pyrénées-Orientales, Vendée | 2009-2011, 2013-2015, 2017, 2019 | NA | January, May, June, July, August | Winter,  Spring,  Summer | NA | NA | NA | INPN (2023) |
|  | Doubs, Ille-et-Vilaine, Seine-et-Oise | 1953-1971 | NA | NA | NA | NA | NA | NA | Pernot-Visentin et al. (1974) |
|  | Hérault, Gard, Bouches-du-Rhône | 1953-1966 | NA | January, February, March, April, May, June, July, August, September, October, November, December | Winter,  Spring,  Summer,  Autumn | NA | Manual capture,  Larvae capture,  Malaise trap | NA | Rageau et al. (1967) |
|  | Bouches-du-Rhône | 1972-1973 | 6 | May, June, July, August, September | Spring,  Summer | NA | Manitoba trap,  Net capture,  Larvae capture | NA | Raymond (1978) |
|  | Aisne, Bouches-du-Rhône, Doubs, Oise, Pyrénées-Atlantiques, Pyrénées-Orientales, Vendée | 2009-2011, 2013-2015, 2017, 2019 | NA | May, June, July, August | Spring,  Summer | NA | NA | NA | GBIF (2023) |
| *Hybomitra*  *distinguenda* | Pyrénées-Orientales | 2011 | 0.2 | July | Summer | 1450-2700 | N’zi trap | NA | Baldacchino et al. (2014c) |
|  | Pyrénées-Orientales | 2011 | 5 | May, June, July, August, September | Spring,  Summer | 850-2300,  1450-2700 | N’zi and  Vavoua traps | NA | Baldacchino et al. (2014b) |
|  | Ille-et-Vilaine, Loire-Atlantique | 1989-1990 | NA | NA | Summer | 164, 20 | Net Capture,  Manitoba trap,  Car-net | NA | Le Goff et al. (1993) |
|  | Doubs, Gironde, Jura, Pyrénées-Orientales | 2001, 2009, 2013 | NA | January, June, July, August | Winter,  Summer | NA | NA | NA | 2023 INPN (2023) |
|  | Hautes-Pyrénées | 1955-1968 | NA | June, July, August | Summer | NA | NA | NA | Leclercq (1970) |
|  | Alpes-de-Haute-Provence | 1957-1969 | NA | June, July, August | Summer | 250-3400 | NA | NA | Leclercq (1971) |
|  | Hérault, Gard, Bouches-du-Rhône | 1953-1966 | NA | January, February, March, April, May, June, July, August, September, October, November, December | Winter,  Spring,  Summer,  Autumn | NA | Manual capture,  Larvae capture,  Malaise trap | NA | Rageau et al. (1967) |
|  | Hautes-Alpes | 1972-1973, 1978 | 3 | July, August | Summer | NA | Manitoba trap,  Manual capture | NA | Raymond (1979) |
|  | Ardennes, Meurthe-et-Moselle, Vosges, Essonne, Val-d'Oise, Côtes-d’Armor | 1834-1926 | NA | April, May, June, July, August, September | Spring,  Summer,  Autumn | NA | NA | NA | Séguy (1926) |
|  | Pyrénées-Orientales, Gironde, Jura, Doubs | 2001, 2009, 2013 | NA | January, June, July, August | Winter,  Summer | NA | NA | NA | GBIF (2023) |
| *Hybomitra*  *erberi* | Pyrénées-Orientales | 1953-1971 | NA | NA | NA | NA | NA | NA | Pernot-Visentin et al. (1974) |
|  | Bouches-du-Rhône | 1972-1973 | 6 | May, June, July, August, September | Spring,  Summer | NA | Manitoba trap,  Net capture,  Larvae capture | NA | Raymond (1978) |
| *Hybomitra*  *expollicata* | Bouches-du-Rhône | 2011 | 1 | June | Summer | 10 | N’zi trap | NA | Baldacchino et al. (2013a) |
|  | Loire-Atlantique | 2012 | 5 | April, May, June, July, August | Summer | NA | N’zi trap | NA | Lantuejoul (2015) |
|  | Loire-Atlantique | 2013-2014 | NA | NA | NA | NA | NA | NA | Jacquiet et al. (2015) |
|  | Bouches-du-Rhône | 2012 | 1 | June, July | Summer | 10 | N’zi trap (with and without odor bait) | NA | Baldacchino et al. (2013b) |
|  | Lozère | 1992 | NA | July | Summer | NA | NA | NA | INPN (2023) |
|  | Pyrénées-Orientales | 1955-1968 | NA | June, July, August | Summer | NA | NA | NA | Leclercq (1970) |
|  | Hérault, Gard, Bouches-du-Rhône | 1953-1966 | NA | January, February, March, April, May, June, July, August, September, October, November, December | Winter,  Spring,  Summer,  Autumn | NA | Manual capture,  Larvae capture,  Malaise trap | NA | Rageau et al. (1967) |
|  | Bouches-du-Rhône | 1972-1973 | 6 | May, June, July, August, September | Spring,  Summer | NA | Manitoba trap,  Net capture,  Larvae capture | NA | Raymond (1978) |
|  | Rhône | 1834-1926 | NA | April, May, June, July, August, September | Spring,  Summer,  Autumn | NA | NA | NA | Séguy (1926) |
|  | Lozère | 1992 | NA | July | Summer | NA | NA | NA | GBIF (2023) |
| *Hybomitra*  *lundbecki* | Pyrénées-Orientales | 2005 | NA | January | Winter | NA | NA | NA | INPN (2023) |
|  | Ariège | 1955-1968 | NA | June, July, August | Summer | NA | NA | NA | Leclercq (1970) |
|  | Isère | 1953-1971 | NA | NA | NA | NA | NA | NA | Pernot-Visentin et al. (1974) |
|  | Bouches-du-Rhône | 1972-1973 | 6 | May, June, July, August, September | Spring,  Summer | NA | Manitoba trap,  Net capture,  Larvae capture | NA | Raymond (1978) |
|  | Pyrénées-Orientales | 2005 | NA | January | Winter | NA | NA | NA | GBIF (2023) |
| *Hybomitra*  *lurida* | Doubs | 2011 | NA | May | Spring | NA | NA | NA | INPN (2023) |
|  | Haute-Saône, Landes | 1834-1926 | NA | April, May, June, July, August, September | Spring,  Summer,  Autumn | NA | NA | NA | Séguy (1926) |
|  | Doubs | 2011 | NA | May | Spring | NA | NA | NA | GBIF (2023) |
| *Hybomitra*  *micans* | Doubs, Jura | 2009, 2013-2014 | NA | June | Summer | NA | NA | NA | INPN (2023) |
|  | Pyrénées-Orientales | 1955-1968 | NA | June, July, August | Summer | NA | NA | NA | Leclercq (1970) |
|  | Bouches-du-Rhône | 1972-1973 | 6 | May, June, July, August, September | Spring,  Summer | NA | Manitoba trap,  Net capture,  Larvae capture | NA | Raymond (1978) |
|  | All France | NA | NA | NA | NA | NA | NA | NA | Séguy (1924) |
|  | All France | 1834-1926 | NA | April, May, June, July, August, September | Spring,  Summer,  Autumn | NA | NA | NA | Séguy (1926) |
|  | Loire, Jura, Puy-de-Dôme, Doubs | 2009, 2012-2014 | NA | June | Summer | NA | NA | NA | GBIF (2023) |
| *Hybomitra*  *montana* | Pyrénées-Orientales | 2012 | 2 | July, August | Summer | NA | N’zi trap | NA | Baldacchino et al. (2014a) |
|  | Pyrénées-Orientales | 2011 | 0.2 | July | Summer | 1450-2700 | N’zi trap | NA | Baldacchino et al. (2014c) |
|  | Pyrénées-Orientales | 2011 | 5 | May, June, July, August, September | Spring,  Summer | 850-2300,  1450-2700 | N’zi and  Vavoua traps | NA | Baldacchino et al. (2014b) |
|  | Pyrénées-Orientales | 2013-2014 | NA | NA | NA | NA | NA | NA | Jacquiet et al. (2015) |
|  | Pyrénées-Orientales | 1955-1968 | NA | June, July, August | Summer | NA | NA | NA | Leclercq (1970) |
|  | Alpes-de-Haute-Provence | 1957-1969 | NA | June, July, August | Summer | 250-3400 | NA | NA | Leclercq (1971) |
|  | Bouches-du-Rhône | 1972-1973 | 6 | May, June, July, August, September | Spring,  Summer | NA | Manitoba trap,  Net capture,  Larvae capture | NA | Raymond (1978) |
|  | Hautes-Alpes | 1972-1973, 1978 | 3 | July, August | Summer | NA | Manitoba trap,  Manual capture | NA | Raymond (1979) |
|  | Hautes-Pyrénées | 1834-1926 | NA | April, May, June, July, August, September | Spring,  Summer,  Autumn | NA | NA | NA | Séguy (1926) |
|  | Haute-Saône | NA | NA | NA | NA | NA | NA | NA | GBIF (2023) |
| *Hybomitra*  *muhlfeldi* | Aveyron, Doubs, Ille-et-Vilaine, Morbihan | 1953-1971 | NA | NA | NA | NA | NA | NA | Pernot-Visentin et al. (1974) |
| *Hybomitra*  *olsufievina* | Hautes-Pyrénées, Pyrénées-Orientales | 1955-1968 | NA | June, July, August | Summer | NA | NA | NA | Leclercq (1970) |
|  | Alpes-de-Haute-Provence | 1957-1969 | NA | June, July, August | Summer | 250-3400 | NA | NA | Leclercq (1971) |
| *Hybomitra*  *solstitialis* | Pyrénées-Orientales | 2009 | NA | July | Summer | NA | NA | NA | INPN (2023) |
|  | Hautes-Pyrénées, Ariège | 1955-1968 | NA | June, July, August | Summer | NA | NA | NA | Leclercq (1970) |
|  | All France | 1834-1926 | NA | April, May, June, July, August, September | Spring,  Summer,  Autumn | NA | NA | NA | Séguy (1926) |
|  | Pas-de-Calais, Pyrénées-Orientales | 2009, 2021 | NA | July, August | Summer | NA | NA | NA | GBIF (2023) |
| *Hybomitra*  *tropica* | Ille-et-Vilaine, Loire-Atlantique | 1989-1990 | NA | NA | Summer | 164, 20 | Net Capture,  Manitoba trap,  Car-net | NA | Le Goff et al. (1993) |
|  | Creuse, Maine-et-Loire | 1758, 1913 | NA | January, June | Winter,  Summer | NA | NA | NA | INPN (2023) |
|  | Hautes-Pyrénées | 1955-1968 | NA | June, July, August | Summer | NA | NA | NA | Leclercq (1970) |
|  | Doubs, Ille-et-Vilaine, Meurthe-et-Moselle, Morbihan, Nord, Ardennes | 1953-1971 | NA | NA | NA | NA | NA | NA | Pernot-Visentin et al. (1974) |
|  | Bouches-du-Rhône | 1972-1973 | 6 | May, June, July, August, September | Spring,  Summer | NA | Manitoba trap,  Net capture,  Larvae capture | NA | Raymond (1978) |
|  | Hautes-Alpes | 1972-1973, 1978 | 3 | July, August | Summer | NA | Manitoba trap,  Manual capture | NA | Raymond (1979) |
|  | Ardennes, Somme, Meurthe-et-Moselle, Vosges, Eure-et-Loir | 1834-1926 | NA | April, May, June, July, August, September | Spring,  Summer,  Autumn | NA | NA | NA | Séguy (1926) |
|  | Maine-et-Loire, Creuse | 1758, 1913 | NA | January, June | Winter,  Summer | NA | NA | NA | GBIF (2023) |
| *Hybomitra*  *vittata* | Bouches-du-Rhône, Hérault | 1834-1926 | NA | April, May, June, July, August, September | Spring,  Summer,  Autumn | NA | NA | NA | Séguy (1926) |
| *Nemorius*  *vitripennis* | Pyrénées-Orientales | 1955-1968 | NA | June, July, August | Summer | NA | NA | NA | Leclercq (1970) |
|  | Alpes-de-Haute-Provence | 1957-1969 | NA | June, July, August | Summer | 250-3400 | NA | NA | Leclercq (1971) |
|  | Corse | 1953-1971 | NA | NA | NA | NA | NA | NA | Pernot-Visentin et al. (1974) |
| *Pangonius*  *haustellatus* | Bouches-du-Rhône | 2012 | 1 | June, July | Summer | 10 | N’zi trap (with and without odor bait) | NA | Baldacchino et al. (2013b) |
| *Pangonius*  *micans* | Alpes-de-Haute-Provence, Bouches-du-Rhône, Drôme, Gard, Haute-Garonne, Lozère, Var, Vaucluse | 1991, 2007, 2011-2015, 2020-2021 | NA | May, June, July | Spring,  Summer | NA | NA | NA | INPN (2023) |
|  | Ariège | 1955-1968 | NA | June, July, August | Summer | NA | NA | NA | Leclercq (1970) |
|  | Alpes-de-Haute-Provence | 1957-1969 | NA | June, July, August | Summer | 250-3400 | NA | NA | Leclercq (1971) |
|  | Hérault, Gard, Bouches-du-Rhône | 1953-1966 | NA | January, February, March, April, May, June, July, August, September, October, November, December | Winter,  Spring,  Summer,  Autumn | NA | Manual capture,  Larvae capture,  Malaise trap | NA | Rageau et al. (1967) |
|  | Bouches-du-Rhône | 1972-1973 | 6 | May, June, July, August, September | Spring,  Summer | NA | Manitoba trap,  Net capture,  Larvae capture | NA | Raymond (1978) |
|  | Alpes-de-Haute-Provence, Aveyron, Bouches-du-Rhône, Drôme, Gard, Haute-Garonne, Hérault, Isère, Lozère, Var, Vaucluse | 1991, 2007, 2011-2016, 2019-2022 | NA | May, June, July | Spring,  Summer | NA | NA | NA | GBIF (2023) |
| *Philipomyia*  *aprica* | Pyrénées-Orientales | 2012 | 2 | July, August | Summer | NA | N’zi trap | NA | Baldacchino et al. (2014a) |
|  | Pyrénées-Orientales | 2011 | 0.2 | July | Summer | 1450-2700 | N’zi trap | NA | Baldacchino et al. (2014c) |
|  | Pyrénées-Orientales | 2011 | 5 | May, June, July, August, September | Spring,  Summer | 850-2300,  1450-2700 | N’zi and  Vavoua traps | NA | Baldacchino et al. (2014b) |
|  | Pyrénées-Orientales | 2013-2014 | NA | NA | NA | NA | NA | NA | Jacquiet et al. (2015) |
|  | Alpes-de-Haute-Provence, Alpes-Maritimes, Ardèche, Ariège, Bouches-du-Rhône, Dordogne, Drôme, Haute-Loire, Hautes-Alpes, Haute-Savoie, Hautes-Pyrénées, Isère, Jura, Lot, Pyrénées-Atlantiques, Savoie | 1946, 1955, 1966, 2006-2007, 2010, 2012-2016, 2018-2021 | NA | January, May, June, July, August | Winter,  Spring,  Summer | NA | NA | NA | INPN (2023) |
|  | Hautes-Alpes | 1972-1973, 1978 | 3 | July, August | Summer | NA | Manitoba trap,  Manual capture | NA | Raymond (1979) |
|  | Ardennes, Somme, Marne, Meurthe-et-Moselle, Aube, Haute-Marne, Corrèze, Isère, Haute-Savoie, Hautes-Alpes, Var, Pyrénées-Orientales, Hautes-Pyrénées | 1834-1926 | NA | April, May, June, July, August, September | Spring,  Summer,  Autumn | NA | NA | NA | Séguy (1926) |
|  | Alpes-de-Haute-Provence, Alpes-Maritimes, Ardèche, Ariège, Bouches-du-Rhône, Côte-d'Or, Dordogne, Drôme, Gard, Haute-Loire, Hautes-Alpes, Haute-Savoie, Hautes-Pyrénées, Isère, Jura, Loire, Lot, Pyrénées-Atlantiques, Pyrénées-Orientales, Savoie | 1929, 1946, 1955, 1966, 2006-2007, 2010, 2012-2016, 2018-2022 | NA | January, May, June, July, August | Winter,  Spring,  Summer | NA | NA | NA | GBIF (2023) |
| *Philipomyia*  *graeca* | Alpes-de-Haute-Provence, Dordogne, Haute-Savoie | 2013-2015 | NA | June, July, December | Summer,  Winter | NA | NA | NA | INPN (2023) |
|  | Ardennes | 1834-1926 | NA | April, May, June, July, August, September | Spring,  Summer,  Autumn | NA | NA | NA | Séguy (1926) |
|  | Pyrénées-Orientales, Alpes-Maritimes, Alpes-de-Haute-Provence, Dordogne, Haute-Savoie | 2013-2015, 2022 | NA | May, June, July | Spring,  Summer | NA | NA | NA | GBIF (2023) |
| *Silvius*  *algirus* | Alpes-de-Haute-Provence | 1957-1969 | NA | June, July, August | Summer | 250-3400 | NA | NA | Leclercq (1971) |
| *Silvius*  *alpinus* | Ariège, Alpes-Maritimes, Isère, Doubs, Orne, Haute-Garonne, Jura | 2007, 2010-2013, 2022 | NA | June, July, August | Summer | NA | NA | NA | INPN (2023) |
|  | Hautes-Pyrénées, Haute-Garonne | 1955-1968 | NA | June, July, August | Summer | NA | NA | NA | Leclercq (1970) |
|  | Alpes-de-Haute-Provence | 1957-1969 | NA | June, July, August | Summer | 250-3400 | NA | NA | Leclercq (1971) |
|  | Aveyron | 1953-1971 | NA | NA | NA | NA | NA | NA | Pernot-Visentin et al. (1974) |
|  | Alpes-Maritimes, Ariège, Doubs, Haute-Garonne, Isère, Jura, Savoie | 2007, 2010-2013, 2021 | NA | June, July, August | Summer | NA | NA | NA | GBIF (2023) |
| *Silvius*  *variegatus* | Hautes-Pyrénées | 1955-1968 | NA | June, July, August | Summer | NA | NA | NA | Leclercq (1970) |
|  | Alpes-de-Haute-Provence | 1957-1969 | NA | June, July, August | Summer | 250-3400 | NA | NA | Leclercq (1971) |
|  | Aisne, Yvelines, Val-d'Oise, Hauts-de-Seine, Essonne, Seine-Saint-Denis, Val-de-Marne, Seine-et-Marne, Mayenne, Puy-de-Dôme, Hautes-Alpes, Drôme, Alpes-Maritimes, Aude, Hautes-Pyrénées | 1834-1926 | NA | April, May, June, July, August, September | Spring,  Summer,  Autumn | NA | NA | NA | Séguy (1926) |
| *Tabanus*  *autumnalis* | Maine-et-Loire | 2008 | 1 | July | Summer | 71 | Vavoua trap | NA | Grimeau (2012) |
|  | Aisne, Alpes-de-Haute-Provence, Bouches-du-Rhône, Calvados, Doubs, Finistère, Indre, Morbihan, Oise, Pas-de-Calais, Pyrénées-Orientales, Seine-et-Marne, Val-d'Oise, Vendée | 1978, 1995, 2001, 2005, 2008-2010, 2013-2017 | NA | January, May, June, July, August | Winter,  Spring,  Summer | NA | NA | NA | INPN (2023) |
|  | Ariège, Pyrénées-Orientales | 1955-1968 | NA | June, July, August | Summer | NA | NA | NA | Leclercq (1970) |
|  | Alpes-de-Haute-Provence | 1957-1969 | NA | June, July, August | Summer | 250-3400 | NA | NA | Leclercq (1971) |
|  | Corse, Nord | 1953-1971 | NA | NA | NA | NA | NA | NA | Pernot-Visentin et al. (1974) |
|  | Hérault, Gard, Bouches-du-Rhône | 1953-1966 | NA | January, February, March, April, May, June, July, August, September, October, November, December | Winter,  Spring,  Summer,  Autumn | NA | Manual capture,  Larvae capture,  Malaise trap | NA | Rageau et al. (1967) |
|  | Bouches-du-Rhône | 1972-1973 | 6 | May, June, July, August, September | Spring,  Summer | NA | Manitoba trap,  Net capture,  Larvae capture | NA | Raymond (1978) |
|  | All France | NA | NA | NA | NA | NA | NA | NA | Séguy (1924) |
|  | All France | 1834-1926 | NA | April, May, June, July, August, September | Spring,  Summer,  Autumn | NA | NA | NA | Séguy (1926) |
|  | Ain, Aisne, Alpes-de-Haute-Provence, Bouches-du-Rhône, Calvados, Doubs, Finistère, Gers, Haute-Garonne, Indre, Loire-Atlantique, Manche, Mayenne, Morbihan, Nord, Oise, Pas-de-Calais, Pyrénées-Orientales, Seine-et-Marne, Val-d'Oise, Vendée | 1978, 1995, 2001, 2005, 2008-2010, 2013-2017, 2020, 2022 | NA | January, May, June, July, August | Winter,  Spring,  Summer | NA | NA | NA | GBIF (2023) |
| *Tabanus*  *bifarius* | Pyrénées-Orientales | 2009 | NA | June | Summer | NA | NA | NA | INPN (2023) |
|  | Pyrénées-Orientales | 1955-1968 | NA | June, July, August | Summer | NA | NA | NA | Leclercq (1970) |
|  | Alpes-de-Haute-Provence | 1957-1969 | NA | June, July, August | Summer | 250-3400 | NA | NA | Leclercq (1971) |
|  | Bouches-du-Rhône | 1972-1973 | 6 | May, June, July, August, September | Spring,  Summer | NA | Manitoba trap,  Net capture,  Larvae capture | NA | Raymond (1978) |
|  | Rhône, Hautes-Alpes, Var, Aude, Corse | 1834-1926 | NA | April, May, June, July, August, September | Spring,  Summer,  Autumn | NA | NA | NA | Séguy (1926) |
|  | Pyrénées-Orientales | 2009 | NA | June | Summer | NA | NA | NA | GBIF (2023) |
| *Tabanus*  *bovinus* | Ille-et-Vilaine, Loire-Atlantique | 1989-1990 | NA | NA | Summer | 164, 20 | Net Capture,  Manitoba trap,  Car-net | NA | Le Goff et al. (1993) |
|  | Tarn | 1984 | 3 | July, August, September | Summer | NA | Manual capture | NA | Franc (1986) |
|  | Aisne, Allier, Alpes-de-Haute-Provence, Alpes-Maritimes, Ariège, Côtes-d'Armor, Cher, Creuse, Dordogne, Doubs, Finistère, Gironde, Haute-Loire, Haute-Marne, Ille-et-Vilaine, Landes, Loire-Atlantique, Loiret, Lozère, Maine-et-Loire, Nord, Oise, Orne, Puy-de-Dôme, Pyrénées-Atlantiques, Pyrénées-Orientales, Seine-et-Marne, Somme, Vosges, Yonne | 1758, 1913, 1923, 1934, 1988, 1991, 1995-1997, 2002-2004, 2006, 2008-2013, 2015-2021 | NA | January, May, June, July, August, September, December | Winter,  Spring,  Summer | NA | NA | NA | INPN (2023) |
|  | Ille-et-Vilaine | 1989 | 1 | July | Summer | 71-258 | Net capture | *Spiroplasma*  sp. | Le Goff et al. (1991) |
|  | Pyrénées-Atlantiques, Ariège, Pyrénées-Orientales | 1955-1968 | NA | June, July, August | Summer | NA | NA | NA | Leclercq (1970) |
|  | Alpes-Maritimes, Bouches-du-Rhône, Corse, Doubs, Gard, Meuse | 1953-1971 | NA | NA | NA | NA | NA | NA | Pernot-Visentin et al. (1974) |
|  | Hérault, Gard, Bouches-du-Rhône | 1953-1966 | NA | January, February, March, April, May, June, July, August, September, October, November, December | Winter,  Spring,  Summer,  Autumn | NA | Manual capture,  Larvae capture,  Malaise trap | NA | Rageau et al. (1967) |
|  | Bouches-du-Rhône | 1972-1973 | 6 | May, June, July, August, September | Spring,  Summer | NA | Manitoba trap,  Net capture,  Larvae capture | NA | Raymond (1978) |
|  | Hautes-Alpes | 1972-1973, 1978 | 3 | July, August | Summer | NA | Manitoba trap,  Manual capture | NA | Raymond (1979) |
|  | All France | NA | NA | NA | NA | NA | NA | NA | Séguy (1924) |
|  | All France | 1834-1926 | NA | April, May, June, July, August, September | Spring,  Summer,  Autumn | NA | NA | NA | Séguy (1926) |
|  | Aisne, Allier, Alpes-de-Haute-Provence, Alpes-Maritimes, Ariège, Cher, Côtes-d'Armor, Creuse, Dordogne, Doubs, Finistère, Gironde, Haute-Loire, Haute-Marne, Haute-Savoie, Ille-et-Vilaine, Landes, Loire-Atlantique, Loiret, Lozère, Maine-et-Loire, Nord, Oise, Orne, Pas-de-Calais, Pyrénées-Orientales, Seine-et-Marne, Somme, Vosges, Yonne | 1758, 1913, 1923, 1934-1935, 1988, 1991, 1995-1997, 2002-2004, 2006, 2008-2013, 2015-2022 | NA | January, May, June, July, August, September, December | Winter,  Spring,  Summer | NA | NA | NA | GBIF (2023) |
| *Tabanus*  *briani* | Pyrénées-Orientales | 2009 | NA | June, July | Summer | NA | NA | NA | INPN (2023) |
|  | Pyrénées-Orientales | 1955-1968 | NA | June, July, August | Summer | NA | NA | NA | Leclercq (1970) |
|  | Alpes-de-Haute-Provence | 1957-1969 | NA | June, July, August | Summer | 250-3400 | NA | NA | Leclercq (1971) |
|  | Hautes-Alpes | 1972-1973, 1978 | 3 | July, August | Summer | NA | Manitoba trap,  Manual Capture | NA | Raymond (1979) |
|  | Pyrénées-Orientales | 2009 | NA | June, July | Summer | NA | NA | NA | GBIF (2023) |
| *Tabanus*  *bromius* | Pyrénées-Orientales | 2012 | 2 | July, August | Summer | NA | N’zi trap | NA | Baldacchino et al. (2014a) |
|  | Pyrénées-Orientales | 2011 | 0.2 | July | Summer | 1450-2700 | N’zi trap | NA | Baldacchino et al. (2014c) |
|  | Pyrénées-Orientales | 2011 | 5 | May, June, July, August, September | Spring,  Summer | 850-2300,  1450-2700 | N’zi and  Vavoua trap | NA | Baldacchino et al. (2014b) |
|  | Bouches-du-Rhône | 2011 | 1 | June | Summer | 10 | N’zi trap | NA | Baldacchino et al. (2013a) |
|  | Loire-Atlantique | 2012 | 5 | April, May, June, July, August | Summer | NA | N’zi trap | NA | Lantuejoul (2015) |
|  | Loire-Atlantique, Pyrénées-Orientales | 2013-2014 | NA | NA | NA | NA | NA | NA | Jacquiet et al. (2015) |
|  | Ille-et-Vilaine, Loire-Atlantique | 1989-1990 | NA | NA | Summer | 164, 20 | Net Capture,  Manitoba trap,  Car-net | NA | Le Goff et al. (1993) |
|  | Bouches-du-Rhône | 2012 | 1 | June, July | Summer | 10 | N’zi trap (with and without odor bait) | NA | Baldacchino et al. (2013b) |
|  | Ain, Aisne, Alpes-de-Haute-Provence, Alpes-Maritimes, Ariège, Bouches-du-Rhône, Calvados, Charente-Maritime, Cher, Dordogne, Doubs, Drôme, Haute-Vienne, Indre-et-Loire, Isère, Loiret, Lozère, Maine-et-Loire, Morbihan, Nièvre, Oise, Orne, Pyrénées-Atlantiques, Pyrénées-Orientales, Seine-et-Marne, Somme, Vaucluse | 1913, 1955, 1957, 1992-1993, 1995, 2001, 2003, 2005-2006, 2008-2020 | NA | January, May, June, July, August, September, October, December | Winter,  Spring,  Summer,  Autumn | NA | NA | NA | INPN (2023) |
|  | Ille-et-Vilaine | 1989 | 1 | July | Summer | 71-258 | Net capture | *Spiroplasma*  sp. | Le Goff et al. (1991) |
|  | Pyrénées-Atlantiques, Ariège, Pyrénées-Orientales, Haute-Garonne, Hautes-Pyrénées | 1955-1968 | NA | June, July, August | Summer | NA | NA | NA | Leclercq (1970) |
|  | Alpes-de-Haute-Provence | 1957-1969 | NA | June, July, August | Summer | 250-3400 | NA | NA | Leclercq (1971) |
|  | Ain, Alpes-Maritimes, Aveyron, Alpes-de-Haute-Provence, Bouches-du-Rhône, Doubs, Gard, Ille-et-Vilaine, Isère, Les Landes, Loire-Atlantique, Morbihan, Puy-de-Dôme, Pyrénées-Atlantiques, Pyrénées-Orientales, Bas-Rhin, Haut-Rhin, Savoie, Haute-Savoie | 1953-1971 | NA | NA | NA | NA | NA | NA | Pernot-Visentin et al. (1974) |
|  | Hérault, Gard, Bouches-du-Rhône | 1953-1966 | NA | January, February, March, April, May, June, July, August, September, October, November, December | Winter,  Spring,  Summer,  Autumn | NA | Manual capture,  Larvae capture,  Malaise trap | NA | Rageau et al. (1967) |
|  | Bouches-du-Rhône | 1972-1973 | 6 | May, June, July, August, September | Spring,  Summer | NA | Manitoba trap,  Net capture,  Larvae capture | NA | Raymond (1978) |
|  | Hautes-Alpes | 1972-1973, 1978 | 3 | July, August | Summer | NA | Manitoba trap,  Manual capture | NA | Raymond (1979) |
|  | All France | NA | NA | NA | NA | NA | NA | NA | Séguy (1924) |
|  | All France | 1834-1926 | NA | April, May, June, July, August, September | Spring,  Summer,  Autumn | NA | NA | NA | Séguy (1926) |
|  | Ain, Aisne, Alpes-de-Haute-Provence, Alpes-Maritimes, Ariège, Bas-Rhin, Bouches-du-Rhône, Calvados, Charente-Maritime, Dordogne, Doubs, Drôme, Haute-Saône, Haute-Vienne, Haut-Rhin, Ille-et-Vilaine, Indre-et-Loire, Isère, Loire, Loiret, Lot-et-Garonne, Lozère, Maine-et-Loire, Morbihan, Moselle, Nièvre, Nord, Oise, Orne, Pas-de-Calais, Pyrénées-Atlantiques, Pyrénées-Orientales, Rhône, Sarthe, Savoie, Seine-et-Marne, Somme, Tarn, Vaucluse, Yvelines | 1913, 1955, 1957, 1992-1993, 1995, 2001, 2003, 2005-2006, 2008-2022 | NA | January, May, June, July, August, September, October, December | Winter,  Spring,  Summer,  Autumn | NA | NA | NA | GBIF (2023) |
| *Tabanus*  *cordiger* | Bouches-du-Rhône | 2011 | 1 | June | Summer | 10 | N’zi trap | NA | Baldacchino et al. (2013a) |
|  | Bouches-du-Rhône | 2012 | 1 | June, July | Summer | 10 | N’zi trap (with and without odor bait) | NA | Baldacchino et al. (2013b) |
|  | Alpes-de-Haute-Provence, Pyrénées-Orientales, Vaucluse | 2001, 2009, 2011, 2017 | NA | January, May, June, July, August, September | Winter,  Spring,  Summer | NA | NA | NA | INPN (2023) |
|  | Hautes-Pyrénées, Pyrénées-Orientales | 1955-1968 | NA | June, July, August | Summer | NA | NA | NA | Leclercq (1970) |
|  | Alpes-de-Haute-Provence | 1957-1969 | NA | June, July, August | Summer | 250-3400 | NA | NA | Leclercq (1971) |
|  | Corse, Haute-Savoie | 1953-1971 | NA | NA | NA | NA | NA | NA | Pernot-Visentin et al. (1974) |
|  | Hérault, Gard, Bouches-du-Rhône | 1953-1966 | NA | January, February, March, April, May, June, July, August, September, October, November, December | Winter,  Spring,  Summer,  Autumn | NA | Manual capture,  Larvae capture,  Malaise trap | NA | Rageau et al. (1967) |
|  | Bouches-du-Rhône | 1972-1973 | 6 | May, June, July, August, September | Spring,  Summer | NA | Manitoba trap,  Net capture,  Larvae capture | NA | Raymond (1978) |
|  | Hautes-Alpes | 1972-1973, 1978 | 3 | July, August | Summer | NA | Manitoba trap,  Manual capture | NA | Raymond (1979) |
|  | Ardennes, Jura, Haute-Marne, Allier, Rhône, Isère, Hautes-Alpes, Vaucluse, Alpes-de-Haute-Provence, Var, Bouches-du-Rhône, Aude, Pyrénées-Orientales, Hautes-Pyrénées | 1834-1926 | NA | April, May, June, July, August, September | Spring,  Summer,  Autumn | NA | NA | NA | Séguy (1926) |
|  | Alpes-de-Haute-Provence | 1967 | NA | August | Summer | NA | NA | NA | GBIF (2023) |
| *Tabanus*  *darimonti* | Bouches-du-Rhône | 2012 | 1 | June, July | Summer | 10 | N’zi trap (with and without odor bait) | NA | Baldacchino et al. (2013b) |
|  | Bouches-du-Rhône | 1980 | NA | July | Summer | NA | NA | NA | GBIF (2023) |
| *Tabanus*  *eggeri* | Bouches-du-Rhône | 2011 | 1 | June | Summer | 10 | N’zi trap | NA | Baldacchino et al. (2013a) |
|  | Bouches-du-Rhône | 2012 | 1 | June, July | Summer | 10 | N’zi trap (with and without odor bait) | NA | Baldacchino et al. (2013b) |
|  | Cher, Dordogne, Indre, Jura, Loiret, Lozère, Oise, Var | 1995, 2008, 2012-2013, 2016, 2018, 2020, 2022 | NA | January, July, August, September | Winter,  Summer | NA | NA | NA | INPN (2023) |
|  | Pyrénées-Atlantiques, Ariège, Pyrénées-Orientales, Hautes-Pyrénées | 1955-1968 | NA | June, July, August | Summer | NA | NA | NA | Leclercq (1970) |
|  | Alpes-de-Haute-Provence | 1957-1969 | NA | June, July, August | Summer | 250-3400 | NA | NA | Leclercq (1971) |
|  | Bouches-du-Rhône | 1972-1973 | 6 | May, June, July, August, September | Spring,  Summer | NA | Manitoba trap,  Net capture,  Larvae capture | NA | Raymond (1978) |
|  | Ardèche, Cher, Dordogne, Indre, Jura, Loiret, Lot-et-Garonne, Lozère, Oise | 1995, 2008-2009, 2012-2013, 2016, 2018, 2020-2021 | NA | January, July, August, September | Winter,  Summer | NA | NA | NA | GBIF (2023) |
| *Tabanus*  *exclusus* | Pyrénées-Orientales | 2011 | 5 | May, June, July, August, September | Spring,  Summer | 850-2300,  1450-2700 | N’zi and  Vavoua traps | NA | Baldacchino et al. (2014b) |
|  | Vaucluse | 2014 | NA | August | Summer | NA | NA | NA | INPN (2023) |
|  | Alpes-de-Haute-Provence | 1957-1969 | NA | June, July, August | Summer | 250-3400 | NA | NA | Leclercq (1971) |
|  | Alpes-Maritimes, Alpes-de-Haute-Provence | 1953-1971 | NA | NA | NA | NA | NA | NA | Pernot-Visentin et al. (1974) |
|  | Hautes-Alpes | 1972-1973, 1978 | 3 | July, August | Summer | NA | Manitoba trap,  Manual capture | NA | Raymond (1979) |
|  | Côte-d'Or, Rhône, Ain, Vaucluse, Bouches-du-Rhône, Var, Aude | 1834-1926 | NA | April, May, June, July, August, September | Spring,  Summer,  Autumn | NA | NA | NA | Séguy (1926) |
|  | Vaucluse | NA | NA | NA | NA | NA | NA | NA | GBIF (2023) |
| *Tabanus*  *glaucopis* | Pyrénées-Orientales | 2012 | 2 | July, August | Summer | NA | N’zi trap | NA | Baldacchino et al. (2014a) |
|  | Pyrénées-Orientales | 2011 | 0.2 | July | Summer | 1450-2700 | N’zi trap | NA | Baldacchino et al. (2014c) |
|  | Pyrénées-Orientales | 2011 | 5 | May, June, July, August, September | Spring,  Summer | 850-2300,  1450-2700 | N’zi and  Vavoua traps | NA | Baldacchino et al. (2014b) |
|  | Alpes-de-Haute-Provence, Alpes-Maritimes, Ariège, Doubs, Drôme, Hérault, Hautes-Alpes, Lozère, Pyrénées-Atlantiques, Pyrénées-Orientales, Somme, Vaucluse | 1946, 1955, 1968, 1973, 1995, 2008-2010, 2012-2013, 2015, 2017, 2019-2020 | NA | January, June, July, August, September | Winter,  Summer | NA | NA | NA | INPN (2023) |
|  | Haute-Garonne, Ariège, Pyrénées-Orientales, Hautes-Pyrénées | 1955-1968 | NA | June, July, August | Summer | NA | NA | NA | Leclercq (1970) |
|  | Alpes-de-Haute-Provence | 1957-1969 | NA | June, July, August | Summer | 250-3400 | NA | NA | Leclercq (1971) |
|  | Ain, Alpes-Maritimes, Alpes-de-Haute-Provence, Bouches-du-Rhône, Doubs, Gard, Isère, Meuse, Pyrénées-Atlantiques, Pyrénées-Orientales, Rhône, Haute-Savoie | 1953-1971 | NA | NA | NA | NA | NA | NA | Pernot-Visentin et al. (1974) |
|  | Hérault, Gard, Bouches-du-Rhône | 1953-1966 | NA | January, February, March, April, May, June, July, August, September, October, November, December | Winter,  Spring,  Summer,  Autumn | NA | Manual capture,  Larvae capture,  Malaise trap | NA | Rageau et al. (1967) |
|  | Hautes-Alpes | 1972-1973, 1978 | 3 | July, August | Summer | NA | Manitoba trap,  Manual capture | NA | Raymond (1979) |
|  | All France | 1834-1926 | NA | April, May, June, July, August, September | Spring,  Summer,  Autumn | NA | NA | NA | Séguy (1926) |
|  | Alpes-de-Haute-Provence, Alpes-Maritimes, Ariège, Doubs, Drôme, Gard, Hautes-Alpes, Hautes-Pyrénées, Hérault, Lozère, Pyrénées-Orientales, Savoie, Seine-et-Marne, Somme, Vaucluse | 1929, 1946, 1955, 1968, 1973, 1995, 2008-2015, 2017-2020 | NA | January, June, July, August, September | Winter,  Summer | NA | NA | NA | GBIF (2023) |
| *Tabanus*  *lateralis* | Pyrénées-Orientales | 1955-1968 | NA | June, July, August | Summer | NA | NA | NA | Leclercq (1970) |
| *Tabanus*  *lunatus* | Bouches-du-Rhône | 1972-1973 | 6 | May, June, July, August, September | Spring,  Summer | NA | Manitoba trap,  Net capture,  Larvae capture | NA | Raymond (1978) |
|  | Vaucluse, Var | 1834-1926 | NA | April, May, June, July, August, September | Spring,  Summer,  Autumn | NA | NA | NA | Séguy (1926) |
| *Tabanus*  *maculicornis* | Pyrénées-Orientales | 2011 | 0.2 | July | Summer | 1450-2700 | N’zi trap | NA | Baldacchino et al. (2014c) |
|  | Pyrénées-Orientales | 2011 | 5 | May, June, July, August, September | Spring,  Summer | 850-2300,  1450-2700 | N’zi and  Vavoua traps | NA | Baldacchino et al. (2014b) |
|  | Oise, Aisne, Doubs, Jura, Alpes-de-Haute-Provence, Pyrénées-Orientales | 2009, 2011, 2013-2014, 2017-2018 | NA | June, July, August | Summer | NA | NA | NA | INPN (2023) |
|  | Ariège, Pyrénées-Orientales, Hautes-Pyrénées | 1955-1968 | NA | June, July, August | Summer | NA | NA | NA | Leclercq (1970) |
|  | Alpes-de-Haute-Provence | 1957-1969 | NA | June, July, August | Summer | 250-3400 | NA | NA | Leclercq (1971) |
|  | Corrèze : Gard, Loire-Atlantique, Haute-Savoie | 1953-1971 | NA | NA | NA | NA | NA | NA | Pernot-Visentin et al. (1974) |
|  | Bouches-du-Rhône | 1972-1973 | 6 | May, June, July, August, September | Spring,  Summer | NA | Manitoba trap,  Net capture,  Larvae capture | NA | Raymond (1978) |
|  | Hautes-Alpes | 1972-1973, 1978 | 3 | July, August | Summer | NA | Manitoba trap,  Manual capture | NA | Raymond (1979) |
|  | All France | 1834-1926 | NA | April, May, June, July, August, September | Spring,  Summer,  Autumn | NA | NA | NA | Séguy (1926) |
|  | Alpes-de-Haute-Provence, Pyrénées-Orientales, Vaucluse, Jura, Doubs, Oise, Aisne | 2009, 2011, 2013, 2017, 2018 | NA | June, July, August | Summer | NA | NA | NA | GBIF (2023) |
| *Tabanus*  *miki* | Pyrénées-Orientales | 2011 | 5 | May, June, July, August, September | Spring,  Summer | 850-2300,  1450-2700 | N’zi and  Vavoua traps | NA | Baldacchino et al. (2014b) |
|  | Hérault, Alpes-Maritimes, Doubs, Var | 2010, 2012, 2014 | NA | June, July | Summer | NA | NA | NA | INPN (2023) |
|  | Haute-Garonne, Ariège, Pyrénées-Orientales, Hautes-Pyrénées, Pyrénées-Atlantiques | 1955-1968 | NA | June, July, August | Summer | NA | NA | NA | Leclercq (1970) |
|  | Alpes-de-Haute-Provence | 1957-1969 | NA | June, July, August | Summer | 250-3400 | NA | NA | Leclercq (1971) |
|  | Alpes-Maritimes, Pyrénées-Orientales, Savoie | 1953-1971 | NA | NA | NA | NA | NA | NA | Pernot-Visentin et al. (1974) |
|  | Hautes-Alpes | 1972-1973, 1978 | 3 | July, August | Summer | NA | Manitoba trap,  Manual capture | NA | Raymond (1979) |
|  | Meurthe-et-Moselle, Alpes-Maritimes | 1834-1926 | NA | April, May, June, July, August, September | Spring,  Summer,  Autumn | NA | NA | NA | Séguy (1926) |
|  | Var, Hérault, Alpes-Maritimes, Doubs | 2010, 2012, 2014 | NA | June, July | Summer | NA | NA | NA | GBIF (2023) |
| *Tabanus*  *nemoralis* | Alpes-de-Haute-Provence, Pyrénées-Atlantiques, Alpes-Maritimes | 2010, 2015-2019 | NA | May, June, July | Spring,  Summer | NA | NA | NA | INPN (2023) |
|  | Pyrénées-Orientales | 1955-1968 | NA | June, July, August | Summer | NA | NA | NA | Leclercq (1970) |
|  | Alpes-de-Haute-Provence | 1957-1969 | NA | June, July, August | Summer | 250-3400 | NA | NA | Leclercq (1971) |
|  | Hérault, Gard, Bouches-du-Rhône | 1953-1966 | NA | January, February, March, April, May, June, July, August, September, October, November, December | Winter,  Spring,  Summer,  Autumn | NA | Manual capture,  Larvae capture,  Malaise trap | NA | Rageau et al. (1967) |
|  | Bouches-du-Rhône | 1972-1973 | 6 | May, June, July, August, September | Spring,  Summer | NA | Manitoba trap,  Net capture,  Larvae capture | NA | Raymond (1978) |
|  | Hautes-Alpes | 1972-1973, 1978 | 3 | July, August | Summer | NA | Manitoba trap,  Manual capture | NA | Raymond (1979) |
|  | Seine-Maritime, Yvelines, Eure, Lozère, Alpes-de-Haute-Provence, Bouches-du-Rhône | 1834-1926 | NA | April, May, June, July, August, September | Spring,  Summer,  Autumn | NA | NA | NA | Séguy (1926) |
|  | Alpes-de-Haute-Provence, Alpes-Maritimes, Puy-de-Dôme, Pyrénées-Atlantiques | 1962, 2010, 2015-2019 | NA | May, June, July | Spring,  Summer | NA | NA | NA | GBIF (2023) |
| *Tabanus*  *paradoxus* | Lozère, Var, Pyrénées-Orientales, Alpes-de-Haute-Provence | 1992, 2008-2009, 2011, 2013 | NA | February, June, July, August | Winter,  Summer | NA | NA | NA | INPN (2023) |
|  | Alpes-de-Haute-Provence | 1957-1969 | NA | June, July, August | Summer | 250-3400 | NA | NA | Leclercq (1971) |
|  | Alpes-Maritimes | 1953-1971 | NA | NA | NA | NA | NA | NA | Pernot-Visentin et al. (1974) |
|  | Isère, Gard, Var | 1834-1926 | NA | April, May, June, July, August, September | Spring,  Summer,  Autumn | NA | NA | NA | Séguy (1926) |
|  | Pyrénées-Orientales, Var, Vaucluse, Lozère, Savoie, Alpes-de-Haute-Provence | 1992, 2008-2009, 2011-2013 | NA | February, June, July, August | Winter,  Summer | NA | NA | NA | GBIF (2023) |
| *Tabanus*  *quatuornotatus* | Pyrénées-Orientales | 2011 | 5 | May, June, July, August, September | Spring,  Summer | 850-2300,  1450-2700 | N’zi and  Vavoua traps | NA | Baldacchino et al. (2014b) |
|  | Aveyron, Hautes-Alpes, Pyrénées-Atlantiques, Hautes-Alpes, Oise, Alpes-de-Haute-Provence | 1887, 1980, 2014, 2019 | NA | June | Summer | NA | NA | NA | INPN (2023) |
|  | Haute-Garonne | 1955-1968 | NA | June, July, August | Summer | NA | NA | NA | Leclercq (1970) |
|  | Alpes-de-Haute-Provence | 1957-1969 | NA | June, July, August | Summer | 250-3400 | NA | NA | Leclercq (1971) |
|  | Hérault, Gard, Bouches-du-Rhône | 1953-1966 | NA | January, February, March, April, May, June, July, August, September, October, November, December | Winter,  Spring,  Summer,  Autumn | NA | Manual capture,  Larvae capture,  Malaise trap | NA | Rageau et al. (1967) |
|  | Hautes-Alpes | 1972-1973, 1978 | 3 | July, August | Summer | NA | Manitoba trap,  Manual capture | NA | Raymond (1979) |
|  | Somme, Haute-Marne, Marne, Val-d'Oise, Yvelines, Eure, Côte-d'Or, Hautes-Alpes, Drôme | 1834-1926 | NA | April, May, June, July, August, September | Spring,  Summer,  Autumn | NA | NA | NA | Séguy (1926) |
|  | Oise, Aveyron, Savoie, Hautes-Alpes, Pyrénées-Atlantiques, Alpes-de-Haute-Provence | 1887, 1981, 2013, 2019 | NA | June | Summer | NA | NA | NA | GBIF (2023) |
| *Tabanus*  *rectus* | Bouches-du-Rhône | 2011 | 1 | June | Summer | 10 | N’zi trap | NA | Baldacchino et al. (2013a) |
|  | Bouches-du-Rhône | 2012 | 1 | June, July | Summer | 10 | N’zi trap (with and without odor bait) | NA | Baldacchino et al. (2013b) |
|  | Lot-et-Garonne, Cher, Lozère, Ariège, Aveyron, Doubs, Gironde, Vienne | 2006, 2009, 2011, 2013, 2017, 2019-2020 | NA | June, July, August | Summer | NA | NA | NA | INPN (2023) |
|  | Pyrénées-Orientales | 1955-1968 | NA | June, July, August | Summer | NA | NA | NA | Leclercq (1970) |
|  | Alpes-de-Haute-Provence | 1957-1969 | NA | June, July, August | Summer | 250-3400 | NA | NA | Leclercq (1971) |
|  | Vienne | 1953-1971 | NA | NA | NA | NA | NA | NA | Pernot-Visentin et al. (1974) |
|  | Hérault, Gard, Bouches-du-Rhône | 1953-1966 | NA | January, February, March, April, May, June, July, August, September, October, November, December | Winter,  Spring,  Summer,  Autumn | NA | Manual capture,  Larvae capture,  Malaise trap | NA | Rageau et al. (1967) |
|  | Bouches-du-Rhône | 1972-1973 | 6 | May, June, July, August, September | Spring,  Summer | NA | Manitoba trap,  Net capture,  Larvae capture | NA | Raymond (1978) |
|  | Hautes-Alpes | 1972-1973, 1978 | 3 | July, August | Summer | NA | Manitoba trap,  Manual capture | NA | Raymond (1979) |
|  | Maine-et-Loire, Charente, Tarn-et-Garonne, Ardèche, Aude, Var, Bouches-du-Rhône | 1834-1926 | NA | April, May, June, July, August, September | Spring,  Summer,  Autumn | NA | NA | NA | Séguy (1926) |
|  | Ain, Ariège, Aveyron, Cher, Doubs, Eure, Gironde, Lot-et-Garonne, Lozère, Var, Vienne | 1930, 2009, 2011, 2017, 2019, 2021 | NA | June, July, August | Summer | NA | NA | NA | GBIF (2023) |
| *Tabanus*  *regularis* | Bouches-du-Rhône | 2011 | 1 | June | Summer | 10 | N’zi trap | NA | Baldacchino et al. (2013a) |
|  | Bouches-du-Rhône | 2012 | 1 | June, July | Summer | 10 | N’zi trap (with and without odor bait) | NA | Baldacchino et al. (2013b) |
|  | Pyrénées-Atlantiques, Pyrénées-Orientales, Landes | 2009, 2019 | NA | January, June, July, August | Winter,  Summer | NA | NA | NA | INPN (2023) |
|  | Haute-Garonne, Ariège | 1955-1968 | NA | June, July, August | Summer | NA | NA | NA | Leclercq (1970) |
|  | Alpes-de-Haute-Provence | 1957-1969 | NA | June, July, August | Summer | 250-3400 | NA | NA | Leclercq (1971) |
|  | Hérault, Gard, Bouches-du-Rhône | 1953-1966 | NA | January, February, March, April, May, June, July, August, September, October, November, December | Winter,  Spring,  Summer,  Autumn | NA | Manual capture,  Larvae capture,  Malaise trap | NA | Rageau et al. (1967) |
|  | Bouches-du-Rhône | 1972-1973 | 6 | May, June, July, August, September | Spring,  Summer | NA | Manitoba trap,  Net capture,  Larvae capture | NA | Raymond (1978) |
|  | Hautes-Alpes | 1972-1973, 1978 | 3 | July, August | Summer | NA | Manitoba trap,  Manual capture | NA | Raymond (1979) |
|  | Rhône, Var, Bouches-du-Rhône, Hautes-Pyrénées | 1834-1926 | NA | April, May, June, July, August, September | Spring,  Summer,  Autumn | NA | NA | NA | Séguy (1926) |
|  | Pyrénées-Orientales, Pyrénées-Atlantiques, Landes | 2009, 2019 | NA | January, June, July, August | Winter,  Summer | NA | NA | NA | GBIF (2023) |
| *Tabanus*  *rupium* | Pyrénées-Orientales | 2011 | 0.2 | July | Summer | 1450-2700 | N’zi trap | NA | Baldacchino et al. (2014c) |
|  | Pyrénées-Orientales | 2011 | 5 | May, June, July, August, September | Spring,  Summer | 850-2300,  1450-2700 | N’zi and  Vavoua traps | NA | Baldacchino et al. (2014b) |
|  | Hautes-Alpes | 1968 | NA | July | Summer | NA | NA | NA | INPN (2023) |
|  | Hautes-Pyrénées | 1955-1968 | NA | June, July, August | Summer | NA | NA | NA | Leclercq (1970) |
|  | Alpes-de-Haute-Provence | 1957-1969 | NA | June, July, August | Summer | 250-3400 | NA | NA | Leclercq (1971) |
|  | Hautes-Alpes | 1972-1973, 1978 | 3 | July, August | Summer | NA | Manitoba trap,  Manual capture | NA | Raymond (1979) |
|  | Hautes-Alpes | 1968 | NA | July | Summer | NA | NA | NA | GBIF (2023) |
| *Tabanus*  *spectabilis* | Yvelines, Haute-Marne, Indre, Pyrénées-Orientales | 1834-1926 | NA | April, May, June, July, August, September | Spring,  Summer,  Autumn | NA | NA | NA | Séguy (1926) |
| *Tabanus*  *spodopterus* | Hautes-Alpes, Pyrénées-Orientales, Hautes-Alpes, Dordogne | 1985, 1989, 2009, 2013 | NA | June, July, August | Summer | NA | NA | NA | INPN (2023) |
|  | Pyrénées-Orientales | 1955-1968 | NA | June, July, August | Summer | NA | NA | NA | Leclercq (1970) |
|  | Alpes-de-Haute-Provence | 1957-1969 | NA | June, July, August | Summer | 250-3400 | NA | NA | Leclercq (1971) |
|  | Ain, Gard, Isère, Puy-de-Dôme | 1953-1971 | NA | NA | NA | NA | NA | NA | Pernot-Visentin et al. (1974) |
|  | Bouches-du-Rhône | 1972-1973 | 6 | May, June, July, August, September | Spring,  Summer | NA | Manitoba trap,  Net capture,  Larvae capture | NA | Raymond (1978) |
|  | Hautes-Alpes | 1972-1973, 1978 | 3 | July, August | Summer | NA | Manitoba trap,  Manual capture | NA | Raymond (1979) |
|  | Yvelines, Val-d'Oise, Hauts-de-Seine, Essonne, Seine-Saint-Denis, Val-de-Marne, Seine-et-Marne, Indre, Puy-de-Dôme, Hautes-Alpes, Isère, Landes, Aveyron | 1834-1926 | NA | April, May, June, July, August, September | Spring,  Summer,  Autumn | NA | NA | NA | Séguy (1926) |
|  | Pyrénées-Orientales, Hautes-Alpes, Dordogne | 1985, 1989, 2009, 2013 | NA | June, July, August | Summer | NA | NA | NA | GBIF (2023) |
| *Tabanus*  *sudeticus* | Pyrénées-Orientales | 2012 | 2 | July, August | Summer | NA | N’zi trap | NA | Baldacchino et al. (2014a) |
|  | Pyrénées-Orientales | 2011 | 0.2 | July | Summer | 1450-2700 | N’zi trap | NA | Baldacchino et al. (2014c) |
|  | Pyrénées-Orientales | 2011 | 5 | May, June, July, August, September | Spring,  Summer | 850-2300,  1450-2700 | N’zi and  Vavoua traps | NA | Baldacchino et al. (2014b) |
|  | Bouches-du-Rhône | 2011 | 1 | June | Summer | 10 | N’zi trap | NA | Baldacchino et al. (2013a) |
|  | Ille-et-Vilaine, Loire-Atlantique | 1989-1990 | NA | NA | Summer | 164, 20 | Net Capture,  Manitoba trap,  Car-net | NA | Le Goff et al. (1993) |
|  | Tarn | 1984 | 3 | July, August, September | Summer | NA | Manual capture | NA | Franc (1986) |
|  | Bouches-du-Rhône | 2012 | 1 | June, July | Summer | 10 | N’zi trap (with and without odor bait) | NA | Baldacchino et al. (2013b) |
|  | Maine-et-Loire | 2008 | 1 | July | Summer | 71 | Vavoua trap | NA | Grimeau (2012) |
|  | Aisne, Ariège, Bouches-du-Rhône, Côtes-d'Armor, Calvados, Cher, Corrèze, Dordogne, Doubs, Finistère, Corse, Haute-Vienne, Haut-Rhin, Indre, Loire-Atlantique, Lozère, Manche, Nièvre, Orne, Pyrénées-Orientales, Seine-et-Marne | 1946, 1981, 1995, 2002, 2007-2022 | NA | January, April, May, June, July, August | Winter,  Spring,  Summer | NA | NA | NA | INPN (2023) |
|  | Ille-et-Vilaine | 1989 | 1 | July | Summer | 71-258 | Net capture | *Spiroplasma*  sp. | Le Goff et al. (1991) |
|  | Ariège, Pyrénées-Orientales, Hautes-Pyrénées | 1955-1968 | NA | June, July, August | Summer | NA | NA | NA | Leclercq (1970) |
|  | Alpes-de-Haute-Provence | 1957-1969 | NA | June, July, August | Summer | 250-3400 | NA | NA | Leclercq (1971) |
|  | Aveyron, Corse, Doubs, Finistère, Gard, Landes, Savoie | 1953-1971 | NA | NA | NA | NA | NA | NA | Pernot-Visentin et al. (1974) |
|  | Hérault, Gard, Bouches-du-Rhône | 1953-1966 | NA | January, February, March, April, May, June, July, August, September, October, November, December | Winter,  Spring,  Summer,  Autumn | NA | Manual capture,  Larvae capture,  Malaise trap | NA | Rageau et al. (1967) |
|  | Bouches-du-Rhône | 1972-1973 | 6 | May, June, July, August, September | Spring,  Summer | NA | Manitoba trap,  Net capture,  Larvae capture | NA | Raymond (1978) |
|  | Hautes-Alpes | 1972-1973, 1978 | 3 | July, August | Summer | NA | Manitoba trap,  Manual capture | NA | Raymond (1979) |
|  | All France | NA | NA | NA | NA | NA | NA | NA | Séguy (1924) |
|  | Yvelines, Val-d'Oise, Hauts-de-Seine, Essonne, Seine-Saint-Denis, Val-de-Marne, Seine-et-Marne, Puy-de-Dôme, Creuse, Haute-Vienne, Cantal, Hautes-Pyrénées | 1834-1926 | NA | April, May, June, July, August, September | Spring,  Summer,  Autumn | NA | NA | NA | Séguy (1926) |
|  | Aisne, Ariège, Bouches-du-Rhône, Calvados, Cher, Corrèze, Corse, Côtes-d'Armor, Dordogne, Doubs, Finistère, Haute-Marne, Haute-Vienne, Haut-Rhin, Indre, Landes, Loire-Atlantique, Lozère, Manche, Nièvre, Orne, Pyrénées-Orientales, Savoie, Seine-et-Marne, Yonne | 1946, 1981, 1988, 1995, 2002, 2007-2022 | NA | January, April, May, June, July, August | Winter,  Spring,  Summer | NA | NA | NA | GBIF (2023) |
| *Tabanus*  *tergestinus* | Pyrénées-Orientales | 2011 | 0.2 | July | Summer | 1450-2700 | N’zi trap | NA | Baldacchino et al. (2014c) |
|  | Alpes-Maritimes, Doubs, Drôme, Gard, Indre, Maine-et-Loire, Nièvre, Pyrénées-Orientales, Var, Vaucluse | 2009, 2010-2011, 2014-2016, 2019 | NA | May, June, July | Spring,  Summer | NA | NA | NA | INPN (2023) |
|  | Pyrénées-Orientales, Hautes-Pyrénées | 1955-1968 | NA | June, July, August | Summer | NA | NA | NA | Leclercq (1970) |
|  | Alpes-de-Haute-Provence | 1957-1969 | NA | June, July, August | Summer | 250-3400 | NA | NA | Leclercq (1971) |
|  | Aveyron, Corse, Gard, Gironde | 1953-1971 | NA | NA | NA | NA | NA | NA | Pernot-Visentin et al. (1974) |
|  | Hérault, Gard, Bouches-du-Rhône | 1953-1966 | NA | January, February, March, April, May, June, July, August, September, October, November, December | Winter,  Spring,  Summer,  Autumn | NA | Manual capture,  Larvae capture,  Malaise trap | NA | Rageau et al. (1967) |
|  | Hautes-Alpes | 1972-1973, 1978 | 3 | July, August | Summer | NA | Manitoba trap,  Manual capture | NA | Raymond (1979) |
|  | Yvelines, Essonne, Val-d'Oise, Seine-et-Marne, Maine-et-Loire, Meurthe-et-Moselle, Haute-Saône, Allier, Var, Landes | 1834-1926 | NA | April, May, June, July, August, September | Spring,  Summer,  Autumn | NA | NA | NA | Séguy (1926) |
|  | Alpes-Maritimes, Doubs, Drôme, Gard, Indre, Isère, Nièvre, Pyrénées-Orientales, Savoie, Var, Vaucluse | 2009-2011, 2014-2016, 2019, 2022 | NA | May, June, July | Spring,  Summer | NA | NA | NA | GBIF (2023) |
| *Tabanus*  *tinctus* | Ille-et-Vilaine | 1953-1971 | NA | NA | NA | NA | NA | NA | Pernot-Visentin et al. (1974) |
|  | Hautes-Alpes | 1972-1973, 1978 | 3 | July, August | Summer | NA | Manitoba trap,  Manual capture | NA | Raymond (1979) |
| *Tabanus*  *unifasciatus* | Doubs, Vaucluse | 2010, 2012 | NA | June, August | Summer | NA | NA | NA | INPN (2023) |
|  | Ariège | 1955-1968 | NA | June, July, August | Summer | NA | NA | NA | Leclercq (1970) |
|  | Alpes-de-Haute-Provence | 1957-1969 | NA | June, July, August | Summer | 250-3400 | NA | NA | Leclercq (1971) |
|  | Hautes-Alpes | 1972-1973, 1978 | 3 | July, August | Summer | NA | Manitoba trap,  Manual capture | NA | Raymond (1979) |
|  | Saône-et-Loire, Rhône, Isère, Drôme, Alpes-de-Haute-Provence, Bouches-du-Rhône | 1834-1926 | NA | April, May, June, July, August, September | Spring,  Summer,  Autumn | NA | NA | NA | Séguy (1926) |
|  | Doubs, Vaucluse | 2010, 2012 | NA | June, August | Summer | NA | NA | NA | GBIF (2023) |
| *Therioplectes*  *gigas* | Aisne, Pas-de-Calais, Doubs, Oise, Indre | 1887, 2010, 2013-2014, 2017, 2019-2020 | NA | May, June, July | Spring,  Summer | NA | NA | NA | INPN (2023) |
|  | Alpes-de-Haute-Provence | 1957-1969 | NA | June, July, August | Summer | 250-3400 | NA | NA | Leclercq (1971) |
|  | Aisne, Indre, Doubs, Moselle, Rhône, Oise, Pas-de-Calais | 1887, 2010, 2013-2014, 2017, 2019-2020, 2022 | NA | May, June, July | Spring,  Summer | NA | NA | NA | GBIF (2023) |

Supplementary Table S4. Captures of Hippobosca equina in cattle farms in France between 1758 and 2022 (NA: data not provided).

| Capture | | | | | | | Reference |
| --- | --- | --- | --- | --- | --- | --- | --- |
| Department | Year | Study  duration  (month) | Month | Season | Altitude  (m) | Trapping  method |
| Saône-et-Loire | 1985-1986 | 2 | July, August, September, December, January | Summer,  Winter | 260 | Manual  capture | Moreau et al. (1987) |
| Lot, Ariège | 1991 | 4 | June, July, August, September | Summer | NA | Manual  capture | Franc et al. (1994) |
| Corse, Pyrénées-Atlantiques, Sarthe, Isère, Bouches-du-Rhône | 1956, 1958, 1960-1961, 1964, 1969, 1976 | NA | May, July, August, September | Spring,  Summer | NA | NA | Beaucornu (1976) |
| All France | NA | NA | NA | NA | NA | Manual  capture | Falcoz (1926) |
| Alpes-de-Haute-Provence, Ardèche, Aveyron, Bouches-du-Rhône, Cantal, Cher, Creuse, Dordogne, Gard, Gers, Gironde, Hérault, Corse, Haute-Loire, Hautes-Alpes, Haut-Rhin, Indre-et-Loire, Lot, Lot-et-Garonne, Maine-et-Loire, Pyrénées-Orientales, Saône-et-Loire, Seine-et-Marne, Tarn, Var | 1758, 1974, 1989, 1996, 1998, 2003-2004, 2006-2015, 2017-2022 | NA | January, April, May, June, July, August, September, October, December | Winter,  Spring,  Summer,  Autumn | NA | NA | INPN (2023) |
| All France | NA | NA | NA | NA | NA | NA | Mullen et al. (2009) |
| Hérault, Gard, Bouches-du-Rhône | 1953-1966 | NA | January, February, March, April, May, June, July, August, September, October, November, December | Winter,  Spring,  Summer,  Autumn | NA | Manual  capture | Rageau et al. (1967) |
| All France | NA | NA | NA | NA | NA | NA | Séguy (1924) |
| Pyrénées-Orientales | 1950, 1952 | NA | April, August, September, November | Spring,  Summer,  Autumn | NA | Manual  capture | Theodorides (1954) |
| Alpes-de-Haute-Provence, Ardèche, Aveyron, Bouches-du-Rhône, Cantal, Cher, Corse, Creuse, Dordogne, Gard, Gers, Gironde, Haute-Garonne, Haute-Loire, Hautes-Alpes, Haute-Vienne, Haut-Rhin, Hérault, Indre-et-Loire, Landes, Lot, Lot-et-Garonne, Maine-et-Loire, Nièvre, Pyrénées-Orientales, Saône-et-Loire, Seine-et-Marne, Tarn, Var, Vaucluse, Yonne | 1758, 1974, 1989, 1996, 1998, 2003-2004, 2006-2015, 2017-2022 | NA | January, April, May, June, July, August, September, October, December | Winter,  Spring,  Summer,  Autumn | NA | NA | GBIF (2023) |

Supplementary Table S5. Captures of biting flies in cattle farms in France between 1920 and 2022 (NA: data not provided).

| Species | Capture | | | | | | | Pathogen | Reference |
| --- | --- | --- | --- | --- | --- | --- | --- | --- | --- |
|  | Department | Year | Study  duration  (month) | Month | Season | Altitude  (m) | Trapping  method |  |  |
| *Haematobia*  *irritans* | Ariège | 2008-2009 | 12 | May, June, July, August, September, October, November, December, January, February, March, April | Spring,  Summer,  Autumn,  Winter | 400 | Vavoua trap | NA | Liénard et al. (2011) |
|  | Hérault | 1979, 1981 | 5 | June, July, August, September | Summer | 5 | Emergence Trap | NA | Kirk (1992) |
|  | Saône-et-Loire | 1985-1986 | 2 | July, August, September, December, January | Summer,  Winter | 260 | Manual capture | NA | Moreau et al. (1987) |
|  | Lot, Ariège | 1991 | 4 | June, July, August, September | Summer | NA | Manual capture | NA | Franc et al. (1994) |
|  | Tarn | 1984 | 3 | July, August, September | Summer | NA | Manual capture | NA | Franc (1986) |
|  | Puy-de-Dôme, Côtes-d'Armor, Eure | 1977-1978 | 9 | July, August, September, October | Summer | NA | NA | NA | Bussieras et al. (1980) |
|  | Côtes-d'Armor | 1977-1978 | 2 | August, September | Summer | NA | Manual capture | NA | Pannerer (1980) |
|  | All France | NA | NA | NA | NA | NA | NA | NA | Blanc-Debrune (2019) |
|  | Indre | 2008 | NA | September | Autumn | NA | NA | NA | INPN (2023) |
|  | All France | NA | NA | NA | NA | NA | NA | NA | Mullen et al. (2009) |
|  | Hérault, Gard, Bouches-du-Rhône | 1953-1966 | NA | January, February, March, April, May, June, July, August, September, October, November, December | Winter,  Spring,  Summer,  Autumn | NA | Manual capture,  CDC trap,  Malaise trap | NA | Rageau et al. (1967) |
|  | Indre | 2008 | NA | September | Autumn | NA | NA | NA | GBIF (2023) |
| *Haematobia*  *stimulans* | Lot, Ariège | 1991 | 4 | June, July, August, September | Summer | NA | Manual capture | NA | Franc et al. (1994) |
|  | Hérault, Gard, Bouches-du-Rhône | 1953-1966 | NA | January, February, March, April, May, June, July, August, September, October, November, December | Winter,  Spring,  Summer,  Autumn | NA | Manual capture,  CDC trap,  Malaise trap | NA | Rageau et al. (1967) |
|  | All France | NA | NA | NA | NA | NA | NA | NA | Séguy (1924) |
|  | Ariège | 1946 | NA | July | Summer | NA | NA | NA | GBIF (2023) |
| *Stomoxys*  *calcitrans* | Ariège | 2008-2009 | 12 | May, June, July, August, September, October, November, December, January, February, March, April | Spring,  Summer,  Autumn,  Winter | 400 | Vavoua trap | *Besnoitia besnoiti* | Liénard et al. (2011) |
|  | Loire-Atlantique | 2012 | 5 | April, May, June, July, August | Summer | NA | N’zi trap | *Besnoitia besnoiti* | Lantuejoul (2015) |
|  | Loire-Atlantique, Pyrénées-Orientales | 2013-2014 | NA | NA | NA | NA | NA | NA | Jacquiet et al. (2015) |
|  | Finistère, Côtes-d'Armor, Morbihan, Ille-et-Vilaine, Loire-Atlantique | 2000-2002 | NA | NA | NA | NA | Aspirator | NA | Joncour et al. (2006) |
|  | Haute-Garonne | 2009 | 12 | January, February, March, April, May, June, July, August, September, October, November, December | Spring,  Summer | 140 | Vavoua trap | NA | Jacquiet et al. (2014) |
|  | Lot, Ariège | 1991 | 4 | June, July, August, September | Summer | NA | Manual capture | NA | Franc et al. (1994) |
|  | Tarn | 1984 | 3 | July, August, September | Summer | NA | Manual capture | NA | Franc (1986) |
|  | Puy-de-Dôme, Côtes-d'Armor, Eure | 1977-1978 | 9 | July, August, September, October | Summer | NA | NA | NA | Bussieras et al. (1980) |
|  | Finistère, Charente | 1972 | 2 | July, August | Summer | NA | Attractive  sticky trap | NA | Marhic (1973) |
|  | Côtes-d'Armor | 1977-1978 | 2 | August, September | Summer | NA | Manual capture | NA | Pannerer (1980) |
|  | All France | 2011 | NA | NA | NA | NA | NA | NA | Baldacchino (2013) |
|  | All France | 2016 | 12 | January, February, March, April, May, June, July, August, September, October, November, December | Winter,  Spring,  Summer,  Autumn | NA | NA | *Dermatophilus congolensis,*  *Anaplasma marginale,*  *Trypanosoma* sp.*,*  *Besnoitia* sp. | Blanc-Debrune (2019) |
|  | Hautes-Alpes | 2016-2017 | 14 | January, February, March, April, May, June, July, August, September, October, November, December | Winter,  Spring,  Summer,  Autumn | 1990  -  2400 | TDV screen,  Burma Screen,  Russell IPM,  Russell pheromone,  Transparent screen,  Insectrons,  Screen impregnated with deltametrin,  Vavoua and N'zi traps | *Besnoitia* sp. | Blanc-Debrune (2019) |
|  | Maine-et-Loire | 2008 | 1 | July | Summer | 71 | Vavoua trap | *Besnoitia besnoiti* | Grimeau (2012) |
|  | Ariège, Bouches-du-Rhône, Côte-d’Or, Calvados, Cher, Creuse, Deux-Sèvres, Dordogne, Finistère, Ille-et-Vilaine, Indre, Jura, Loire, Loire-Atlantique, Loiret, Lot-et-Garonne, Maine-et-Loire, Morbihan, Moselle, Nord, Orne, Paris, Pyrénées-Atlantiques, Pyrénées-Orientales, Seine-et-Marne, Seine-Maritime | 1996, 2000, 2007-2021 | NA | January, March, April, May, June, July, August, September, October, November, December | Winter,  Spring,  Summer,  Autumn | NA | NA | NA | INPN (2023) |
|  | All France | NA | NA | NA | NA | NA | NA | NA | Mullen et al. (2009) |
|  | Hérault, Gard, Bouches-du-Rhône | 1953-1966 | NA | January, February, March, April, May, June, July, August, September, October, November, December | Winter,  Spring,  Summer,  Autumn | NA | Manual capture,  CDC trap,  Malaise trap | NA | Rageau et al. (1967) |
|  | All France | NA | NA | NA | NA | NA | NA | *Anaplasma marginale,*  *Bacillus anthracis,*  *Dermatophilus congolensis,*  Bovine viral diarrhea virus,  Lumpy skin disease virus,  West Nile virus | Rouet (2011) |
|  | Haute-Garonne | 2009 | 12 | January, February, March, April, May, June, July, August, September, October, November, December | Winter,  Spring,  Summer,  Autumn | 140 | Vavoua trap | NA | Rouet (2011) |
|  | All France | NA | NA | NA | NA | NA | NA | *Trypanosoma evansi,*  *Anaplasma marginale,*  *Dermatophilus congolensis,*  Bovine leukemia virus*,*  Rift Valley Fever virus,  West Nile virus | Salem (2012) |
|  | All France | NA | NA | NA | NA | NA | NA | NA | Séguy (1924) |
|  | All France | 2018 | 12 | January, February, March, April, May, June, July, August, September, October, November, December | Winter,  Spring,  Summer,  Autumn | NA | NA | *Besnoitia besnoiti,*  *Bacillus anthracis,*  *Anaplasma marginale,*  *Dermatophilus congolensis,*  *Trypanosoma evansi,*  Lumpy skin disease virus,  West Nile virus,  Bovine leukemia virus,  Rift Valley fever virus,  Bovine viral diarrhea virus | Sharif (2018) |
|  | Ille-et-Vilaine, Puy-de-Dôme, Marne, Alpes-Maritimes, Paris | 1920 | NA | May, June, July, October | Summer,  Autumn | NA | NA | NA | Surcouf (1921) |
|  | Ariège, Bouches-du-Rhône, Calvados, Cher, Côte-d'Or, Côtes-d'Armor, Creuse, Deux-Sèvres, Dordogne, Finistère, Haute-Garonne, Haute-Savoie, Haute-Vienne, Haut-Rhin, Hérault, Ille-et-Vilaine, Indre, Jura, Loire, Loire-Atlantique, Loiret, Lot-et-Garonne, Maine-et-Loire, Morbihan, Moselle, Nord, Oise, Orne, Paris, Pas-de-Calais, Pyrénées-Atlantiques, Pyrénées-Orientales, Saône-et-Loire, Savoie, Seine-et-Marne, Seine-Maritime, Yonne | 1996, 2000, 2006-2022 | NA | January, March, April, May, June, July, August, September, October, November, December | Winter,  Spring,  Summer,  Autumn | NA | NA | NA | GBIF (2023) |
|  | Haute-Garonne | 2016 | 2 | July, August, September | Summer | 189 | Polyethylene  blue screen | NA | Sharif et al. (2020) |

Supplementary Table S6. Captures of black flies in cattle farms in France between 1868 and 2017 (NA: data not provided).

| Species | Capture | | | | | | | Pathogen | Reference |
| --- | --- | --- | --- | --- | --- | --- | --- | --- | --- |
|  | Department | Year | Study  duration  (month) | Month | Season | Altitude  (m) | Trapping  method |  |  |
| *Prosimulium*  *hirtipes* | Vosges, Savoie, Isère, Hautes-Alpes, Hautes-Pyrénées, Corse, Puy-de-Dôme | 1943-1947 | NA | January, February, March, April, May, June, July, August, September, October, November, December | Winter,  Spring,  Summer,  Autumn | NA | Larvae and adult  manual capture | NA | Grenier (1949) |
|  | Pyrénées-Atlantiques, Hautes-Pyrénées, Ariège, Pyrénées-Orientales | NA | NA | NA | NA | 0-2500 | Larvae manual  capture | NA | Grenier et al. (1951) |
|  | Ariège, Pyrénées-Atlantiques, Pyrénées-Orientales | 1946, 1955, 1968, 1988, 2001, 2004-2005, 2008, 2013 | NA | January, April, July | Winter,  Spring,  Summer | NA | NA | NA | INPN (2023) |
|  | Pyrénées-Atlantiques | 1983-1984 | 5 | April, June, July, September, November | Spring,  Summer,  Autumn | 450-2150 | Drift,  Benthic sampling,  Adult capture | NA | Vinçon et al. (1988) |
|  | Pyrénées-Orientales, Ariège, Pyrénées-Atlantiques | 1946, 1955, 1958, 1988, 2001, 2004-2005, 2008, 2013 | NA | January, April, July | Winter,  Spring,  Summer | NA | NA | NA | GBIF (2023) |
| *Prosimulium*  *latimucro* | Alpes-de-Haute-Provence, Alpes-Maritimes, Pyrénées-Orientales | 1997, 2001-2006, 2008 | NA | January, May, June, July, August, September, October | Winter,  Spring,  Summer,  Autumn | NA | NA | NA | INPN (2023) |
|  | Pyrénées-Atlantiques | 1983-1984 | 5 | April, June, July, September, November | Spring,  Summer,  Autumn | 450-2150 | Drift,  Benthic sampling,  Adult capture | NA | Vinçon et al. (1988) |
|  | Alpes-de-Haute-Provence, Alpes-Maritimes, Pyrénées-Orientales | 1997, 2001-2006, 2008 | NA | January, May, June, July, August, September, October | Winter,  Spring,  Summer,  Autumn | NA | NA | NA | GBIF (2023) |
| *Prosimulium*  *rufipes* | Alpes-de-Haute-Provence, Alpes-Maritimes, Pyrénées-Orientales | 1986-1987, 2001-2006, 2008, 2013, 2017 | NA | January, March, May, June, July, August, October | Winter,  Spring,  Summer,  Autumn | NA | NA | NA | INPN (2023) |
|  | Pyrénées-Atlantiques | 1983-1984 | 5 | April, June, July, September, November | Spring,  Summer,  Autumn | 450-2150 | Drift,  Benthic sampling,  Adult capture | NA | Vinçon et al. (1988) |
|  | Alpes-de-Haute-Provence, Alpes-Maritimes, Pyrénées-Orientales | 1986-1987, 2001-2006, 2008, 2013, 2017 | NA | January, March, May, June, July, August, October | Winter,  Spring,  Summer,  Autumn | NA | NA | NA | GBIF (2023) |
| *Prosimulium*  *tomosvaryi* | Pyrénées-Orientales, Var | 1986-1987, 1997, 2001-2002, 2004-2005, 2013 | NA | January, July | Winter,  Summer | NA | NA | NA | INPN (2023) |
|  | Pyrénées-Atlantiques | 1983-1984 | 5 | April, June, July, September, November | Spring,  Summer,  Autumn | 450-2150 | Drift,  Benthic sampling,  Adult capture | NA | Vinçon et al. (1988) |
|  | Puy-de-Dôme, Pyrénées-Orientales, Var | 1986-1987, 1997, 2001-2002, 2004-2005, 2013 | NA | January, July | Winter,  Summer | NA | NA | NA | GBIF (2023) |
| *Simulium*  *angustipes* | Ille-et-Vilaine, Côtes-d’Armor, Loire-Atlantique, Lozère | 1956-1957, 1960-1961, 1963, 1966, 1970 | NA | March, April, May, July, August, November, December | Winter,  Spring,  Summer,  Autumn | NA | Larvae manual  capture | NA | Beaucournu-Saguez (1977) |
|  | Pyrénées-Orientales | 2013 | NA | January | Winter | NA | NA | NA | INPN (2023) |
|  | Pyrénées-Orientales | 2013 | NA | January | Winter | NA | NA | NA | GBIF (2023) |
| *Simulium*  *angustitarse* | Marne, Aisne | 1988-1992 | NA | February, March, April, May, June, September | Winter,  Spring,  Summer,  Autumn | NA | Larvae and nymph  manual capture | NA | Bouchet et al. (1993) |
|  | Oise, Paris | 1943-1947 | NA | January, February, March, April, May, June, July, August, September, October, November, December | Winter,  Spring,  Summer,  Autumn | NA | Larvae and adult  manual capture | NA | Grenier (1949) |
|  | Pyrénées-Atlantiques | NA | NA | NA | NA | 0-2500 | Larvae manual  capture | NA | Grenier et al. (1951) |
|  | Gironde, Pyrénées-Atlantiques, Pyrénées-Orientales | 1988, 1997, 2001, 2005, 2008, 2012 | NA | January, February | Winter | NA | NA | NA | INPN (2023) |
|  | Pyrénées-Atlantiques | 1972-1973 | 12 | February, March, April, May, June, July, August, September, October, November, December, January | Winter,  Spring,  Summer,  Autumn | NA | CDC trap,  Larvae manual  capture | NA | Neveu et al. (1978) |
|  | Pyrénées-Atlantiques | 1983-1984 | 5 | April, June, July, September, November | Spring,  Summer,  Autumn | 450-2150 | Drift,  Benthic sampling,  Adult capture | NA | Vinçon et al. (1988) |
|  | Gironde, Pyrénées-Atlantiques, Pyrénées-Orientales | 1988, 1997, 2001, 2005, 2008, 2012 | NA | January, February | Winter | NA | NA | NA | GBIF (2023) |
| *Simulium*  *argenteostriatum* | Hautes-Pyrénées, Hautes-Alpes, Isère, Savoie, Pyrénées-Atlantiques | 1943-1947 | NA | January, February, March, April, May, June, July, August, September, October, November, December | Winter,  Spring,  Summer,  Autumn | NA | Larvae and adult  manual capture | NA | Grenier (1949) |
|  | Pyrénées-Atlantiques, Hautes-Pyrénées, Ariège | NA | NA | NA | NA | 0-2500 | Larvae manual  capture | NA | Grenier et al. (1951) |
|  | Pyrénées-Atlantiques | 1983-1984 | 5 | April, June, July, September, November | Spring,  Summer,  Autumn | 450-2150 | Drift,  Benthic sampling,  Adult capture | NA | Vinçon et al. (1988) |
|  | Alpes-de-Haute-Provence, Alpes-Maritimes, Hautes-Pyrénées, Pyrénées-Orientales | 2002-2005 | NA | January, June, July, August, September | Winter,  Summer | NA | NA | NA | GBIF (2023) |
| *Simulium*  *argyreatum* | Aisne, Pyrénées-Orientales | 1986-1987, 1997, 2001, 2004, 2008, 2013 | NA | January, July | Winter,  Summer | NA | NA | NA | INPN (2023) |
|  | Pyrénées-Atlantiques | 1983-1984 | 5 | April, June, July, September, November | Spring,  Summer,  Autumn | 450-2150 | Drift,  Benthic sampling,  Adult capture | NA | Vinçon et al. (1988) |
|  | Aisne, Pyrénées-Orientales | 1986-1987, 1997, 2001, 2004, 2008, 2013 | NA | January, July | Winter,  Summer | NA | NA | NA | GBIF (2023) |
| *Simulium*  *aureum* | Ille-et-Vilaine, Finistère | 1956-1957, 1960-1961, 1963, 1966, 1970 | NA | March, April, May, July, August, November, December | Winter,  Spring,  Summer,  Autumn | NA | Larvae manual  capture | NA | Beaucournu-Saguez (1977) |
|  | Marne, Aisne, Pas-de-Calais, Hautes-Pyrénées | 1988-1992 | NA | February, March, April, May, June, September | Winter,  Spring,  Summer,  Autumn | NA | Larvae and nymph  manual capture | NA | Bouchet et al. (1993) |
|  | Moselle, Meurthe-et-Moselle, Vosges, Isère, Pyrénées-Orientales, Hautes-Pyrénées, Côtes-d'Armor, Yonne, Pas-de-Calais, Finistère, Oise | 1943-1947 | NA | January, February, March, April, May, June, July, August, September, October, November, December | Winter,  Spring,  Summer, Autumn | NA | Larvae and adult  manual capture | NA | Grenier (1949) |
|  | Pyrénées-Atlantiques, Hautes-Pyrénées, Ariège, Pyrénées-Orientales | NA | NA | NA | NA | 0-2500 | Larvae manual  capture | NA | Grenier et al. (1951) |
|  | Pyrénées-Orientales | 1940, 1958 | NA | July, October | Summer,  Autumn | NA | NA | NA | INPN (2023) |
|  | Pyrénées-Atlantiques | 1972-1973 | 12 | February, March, April, May, June, July, August, September, October, November, December, January | Winter,  Spring,  Summer,  Autumn | NA | CDC trap,  Larvae manual  capture | NA | Neveu et al. (1978) |
|  | Pyrénées-Atlantiques | 1983-1984 | 5 | April, June, July, September, November | Spring,  Summer,  Autumn | 450-2150 | Drift,  Benthic sampling,  Adult capture | NA | Vinçon et al. (1988) |
|  | Pyrénées-Orientales, Vaucluse | 1947, 1958 | NA | July, October | Summer,  Autumn | NA | NA | NA | GBIF (2023) |
| *Simulium*  *auricoma* | Hautes-Pyrénées, Pyrénées-Orientales | NA | NA | NA | NA | 0-2500 | Larvae manual  capture | NA | Grenier et al. (1951) |
|  | Alpes-Maritimes, Pyrénées-Orientales | 1986-1987, 2001-2002, 2004-2005, 2013 | NA | January, May, July | Winter,  Spring,  Summer | NA | NA | NA | INPN (2023) |
|  | Pyrénées-Atlantiques | 1983-1984 | 5 | April, June, July, September, November | Spring,  Summer,  Autumn | 450-2150 | Drift,  Benthic sampling,  Adult capture | NA | Vinçon et al. (1988) |
|  | Alpes-Maritimes, Pyrénées-Orientales | 1986-1987, 2001-2002, 2004-2005, 2013 | NA | January, May, July | Winter,  Spring,  Summer | NA | NA | NA | GBIF (2023) |
| *Simulium*  *bertrandi* | Alpes-Maritimes | 2002-2004 | NA | May, June, July | Summer | NA | NA | NA | INPN (2023) |
|  | Pyrénées-Atlantiques | 1983-1984 | 5 | April, June, July, September, November | Spring,  Summer,  Autumn | 450-2150 | Drift,  Benthic sampling,  Adult capture | NA | Vinçon et al. (1988) |
|  | Alpes-Maritimes | 2002-2004 | NA | May, June, July | Summer | NA | NA | NA | GBIF (2023) |
| *Simulium*  *Bezzii* | Hautes-Alpes, Isère, Savoie, Hautes-Pyrénées, Pyrénées-Orientales, Puy-de-Dôme | 1943-1947 | NA | January, February, March, April, May, June, July, August, September, October, November, December | Winter,  Spring,  Summer,  Autumn | NA | Larvae and adult  manual capture | NA | Grenier (1949) |
|  | Pyrénées-Atlantiques, Pyrénées-Orientales | NA | NA | NA | NA | 0-2500 | Larvae manual  capture | NA | Grenier et al. (1951) |
|  | Alpes-de-Haute-Provence, Alpes-Maritimes, Hautes-Alpes, Hautes-Pyrénées, Isère, Pyrénées-Orientales | 1868, 1918, 1946-1947, 1997, 2001, 2003-2006, 2008, 2013 | NA | January, May, June, July, August, September, October | Winter,  Spring,  Summer,  Autumn | NA | NA | NA | INPN (2023) |
|  | Pyrénées-Atlantiques | 1983-1984 | 5 | April, June, July, September, November | Spring,  Summer,  Autumn | 450-2150 | Drift,  Benthic sampling,  Adult capture | NA | Vinçon et al. (1988) |
|  | Alpes-de-Haute-Provence, Alpes-Maritimes, Hautes-Alpes, Hautes-Pyrénées, Indre-et-Loire, Pyrénées-Orientales | 1868, 1918, 1946-1947, 1997, 2001, 2003-2006, 2008, 2013 | NA | January, May, June, July, August, September, October | Winter,  Spring,  Summer,  Autumn | NA | NA | NA | GBIF (2023) |
| *Simulium*  *brevidens* | Alpes-de-Haute-Provence, Pyrénées-Orientales | 2003-2005, 2017 | NA | January, June, July, October | Winter,  Summer,  Autumn | NA | NA | NA | INPN (2023) |
|  | Pyrénées-Atlantiques | 1983-1984 | 5 | April, June, July, September, November | Spring,  Summer,  Autumn | 450-2150 | Drift,  Benthic sampling,  Adult capture | NA | Vinçon et al. (1988) |
|  | Pyrénées-Orientales, Alpes-de-Haute-Provence | 2003-2005, 2017 | NA | January, June, July, October | Winter,  Summer,  Autumn | NA | NA | NA | GBIF (2023) |
| *Simulium*  *carthusiense* | Alpes-de-Haute-Provence, Pyrénées-Orientales, Alpes-Maritimes | 2001-2006, 2013 | NA | January, June, July, September, October | Winter,  Summer,  Autumn | NA | NA | NA | INPN (2023) |
|  | Pyrénées-Atlantiques | 1983-1984 | 5 | April, June, July, September, November | Spring,  Summer,  Autumn | 450-2150 | Drift,  Benthic sampling,  Adult capture | NA | Vinçon et al. (1988) |
|  | Alpes-de-Haute-Provence, Alpes-Maritimes, Pyrénées-Orientales | 2001-2006, 2013 | NA | January, June, July, September, October | Winter,  Summer,  Autumn | NA | NA | NA | GBIF (2023) |
| *Simulium*  *costatum* | Marne, Aisne, Hauts-de-Seine, Oise, Savoie | 1988-1992 | NA | February, March, April, May, June, September | Winter,  Spring,  Summer,  Autumn | NA | Larvae and nymph  manual capture | NA | Bouchet et al. (1993) |
|  | Hauts-de-Seine, Marne | 1943-1947 | NA | January, February, March, April, May, June, July, August, September, October, November, December | Winter,  Spring,  Summer,  Autumn | NA | Larvae and adult  manual capture | NA | Grenier (1949) |
|  | Alpes-Maritimes, Pyrénées-Orientales | 2001, 2003-2005 | NA | January, July | Winter,  Summer | NA | NA | NA | INPN (2023) |
|  | Pyrénées-Orientales, Alpes-Maritimes | 2001, 2003-2005 | NA | January, July | Winter,  Summer | NA | NA | NA | GBIF (2023) |
| *Simulium*  *cryophilum* | Alpes-Maritimes, Gironde, Pyrénées-Atlantiques, Pyrénées-Orientales | 1986-1988, 2001-2006, 2008, 2012-2013 | NA | January, February, June, July | Winter,  Summer | NA | NA | NA | INPN (2023) |
|  | Pyrénées-Atlantiques | 1983-1984 | 5 | April, June, July, September, November | Spring,  Summer,  Autumn | 450-2150 | Drift,  Benthic sampling,  Adult capture | NA | Vinçon et al. (1988) |
|  | Alpes-Maritimes, Gironde, Pyrénées-Atlantiques, Pyrénées-Orientales | 1986-1988, 2001-2006, 2008, 2012-2013 | NA | January, February, June, July | Winter,  Summer | NA | NA | NA | GBIF (2023) |
| *Simulium*  *equinum* | Marne, Aisne | 1988-1992 | NA | February, March, April, May, June, September | Winter,  Spring,  Summer,  Autumn | NA | Larvae and nymph  manual capture | NA | Bouchet et al. (1993) |
|  | Oise, Paris, Somme, Puy-de-Dôme, Aveyron | 1943-1947 | NA | January, February, March, April, May, June, July, August, September, October, November, December | Winter,  Spring,  Summer,  Autumn | NA | Larvae and adult  manual capture | NA | Grenier (1949) |
|  | Seine-et-Marne, Lozère, Loiret | 1995, 2002, 2009, 2014 | NA | January, March, April, May, July, September | Winter,  Spring,  Summer,  Autumn | NA | NA | NA | INPN (2023) |
|  | Pyrénées-Atlantiques | 1972-1973 | 12 | February, March, April, May, June, July, August, September, October, November, December, January | Winter,  Spring,  Summer,  Autumn | NA | CDC trap,  Larvae manual  capture | NA | Neveu et al. (1978) |
|  | Hérault, Gard, Bouches-du-Rhône | 1953-1966 | NA | January, February, March, April, May, June, July, August, September, October, November, December | Winter,  Spring,  Summer,  Autumn | NA | Larvae manual  capture,  CDC trap | NA | Rageau et al. (1967) |
|  | Loiret, Lozère, Seine-et-Marne | 1995, 2002, 2009, 2014 | NA | January, March, April, May, July, September | Winter,  Spring,  Summer | NA | NA | NA | GBIF (2023) |
| *Simulium*  *erythrocephalum* | Vosges | 1978 | 4 | April, May, June, July | Summer | 560 | Larvae manual  capture | NA | Noirtin et al. (1981) |
|  | Marne, Aisne | 1988-1992 | NA | February, March, April, May, June, September | Winter,  Spring,  Summer,  Autumn | NA | Larvae and nymph  manual capture | NA | Bouchet et al. (1993) |
|  | Vienne | 1943-1947 | NA | January, February, March, April, May, June, July, August, September, October, November, December | Winter,  Spring,  Summer,  Autumn | NA | Larvae and adult  manual capture | NA | Grenier (1949) |
| *Simulium*  *intermedium* | Pyrénées-Atlantiques | 1983-1984 | 5 | April, June, July, September, November | Spring,  Summer,  Autumn | 450-2150 | Drift,  Benthic sampling,  Adult capture | NA | Vinçon et al. (1988) |
| *Simulium*  *latigonium* | Pyrénées-Atlantiques | 1983-1984 | 5 | April, June, July, September, November | Spring,  Summer,  Autumn | 450-2150 | Drift,  Benthic sampling,  Adult capture | NA | Vinçon et al. (1988) |
| *Simulium*  *latipes* | Vosges | 1978 | 4 | April, May, June, July | Summer | 560 | Larvae manual  capture | NA | Noirtin et al. (1981) |
|  | Marne, Aisne | 1988-1992 | NA | February, March, April, May, June, September | Winter,  Spring,  Summer,  Autumn | NA | Larvae and nymph  manual capture | NA | Bouchet et al. (1993) |
|  | Hautes-Alpes, Isère, Savoie, Hautes-Pyrénées, Vosges, Puy-de-Dôme, Corse, Marne, Maine-et-Loire, Vienne, Yvelines, Ille-et-Vilaine, Puy-de-Dôme | 1943-1947 | NA | January, February, March, April, May, June, July, August, September, October, November, December | Winter,  Spring,  Summer,  Autumn | NA | Larvae and adult  manual capture | NA | Grenier (1949) |
|  | Pyrénées-Atlantiques, Hautes-Pyrénées | NA | NA | NA | NA | 0-2500 | Larvae manual  capture | NA | Grenier et al. (1951) |
|  | Alpes-Maritimes, Gironde, Hautes-Pyrénées, Pyrénées-Orientales | 1946, 1955, 2004, 2012 | NA | January, February, May | Winter,  Spring | NA | NA | NA | INPN (2023) |
|  | Pyrénées-Atlantiques | 1972-1973 | 12 | February, March, April, May, June, July, August, September, October, November, December, January | Winter,  Spring,  Summer,  Autumn | NA | CDC trap,  Larvae manual  capture | NA | Neveu et al. (1978) |
|  | Alpes-Maritimes, Ardennes, Gironde, Hautes-Pyrénées, Pyrénées-Orientales | 1946, 1955, 2004, 2012 | NA | January, February, May | Winter,  Spring | NA | NA | NA | GBIF (2023) |
| *Simulium*  *lineatum* | Marne, Aisne | 1988-1992 | NA | February, March, April, May, June, September | Winter,  Spring,  Summer,  Autumn | NA | Larvae and nymph  manual capture | NA | Bouchet et al. (1993) |
|  | Oise, Paris, Somme, Puy-de-Dôme, Aveyron, Vienne | 1943-1947 | NA | January, February, March, April, May, June, July, August, September, October, November, December | Winter,  Spring,  Summer,  Autumn | NA | Larvae and adult  manual capture | NA | Grenier (1949) |
|  | Pyrénées-Orientales | NA | NA | NA | NA | 0-2500 | Larvae manual  capture | NA | Grenier et al. (1951) |
|  | Pyrénées-Orientales | 1997 | NA | January | Winter | NA | NA | NA | INPN (2023) |
|  | Pyrénées-Atlantiques | 1972-1973 | 12 | February, March, April, May, June, July, August, September, October, November, December, January | Winter,  Spring,  Summer,  Autumn | NA | CDC trap,  Larvae manual  capture | NA | Neveu et al. (1978) |
|  | Pyrénées-Atlantiques | 1983-1984 | 5 | April, June, July, September, November | Spring,  Summer,  Autumn | 450-2150 | Drift,  Benthic sampling,  Adult capture | NA | Vinçon et al. (1988) |
|  | Saône-et-Loire, Pyrénées-Orientales | 1997, 2005 | NA | January, September | Winter,  Summer | NA | NA | NA | GBIF (2023) |
| *Simulium*  *monticola* | Hautes-Pyrénées, Vosges, Jura, Corse, Puy-de-Dôme, Pyrénées-Orientales | 1943-1947 | NA | January, February, March, April, May, June, July, August, September, October, November, December | Winter,  Spring,  Summer,  Autumn | NA | Larvae and adult  manual capture | NA | Grenier (1949) |
|  | Pyrénées-Atlantiques, Hautes-Pyrénées, Ariège, Pyrénées-Orientales | NA | NA | NA | NA | 0-2500 | Larvae manual  capture | NA | Grenier et al. (1951) |
|  | Alpes-de-Haute-Provence, Alpes-Maritimes, Hautes-Pyrénées, Pyrénées-Orientales | 1946, 1958, 1986-1987, 1997, 2001-2006, 2008, 2013 | NA | January, May, July, August, September, October, November, December | Winter,  Spring,  Summer,  Autumn | NA | NA | NA | INPN (2023) |
|  | Pyrénées-Atlantiques | 1983-1984 | 5 | April, June, July, September, November | Spring,  Summer,  Autumn | 450-2150 | Drift,  Benthic sampling,  Adult capture | NA | Vinçon et al. (1988) |
|  | Alpes-de-Haute-Provence, Alpes-Maritimes, Hautes-Pyrénées, Pyrénées-Orientales | 1946, 1958, 1986-1987, 1997, 2001-2006, 2008, 2013 | NA | January, May, July, August, September, October, November, December | Winter,  Spring,  Summer,  Autumn | NA | NA | NA | GBIF (2023) |
| *Simulium*  *noelleri* | Marne, Aisne | 1988-1992 | NA | February, March, April, May, June, September | Winter,  Spring,  Summer,  Autumn | NA | Larvae and nymph  manual capture | NA | Bouchet et al. (1993) |
|  | Yvelines, Yonne, Allier, Ille-et-Vilaine, Oise, Essonne, Paris | 1943-1947 | NA | January, February, March, April, May, June, July, August, September, October, November, December | Winter,  Spring,  Summer,  Autumn | NA | Larvae and adult  manual capture | NA | Grenier (1949) |
|  | Aisne | 2008 | NA | July | Summer | NA | NA | NA | INPN (2023) |
|  | Aisne | 2008 | NA | July | Summer | NA | NA | NA | GBIF (2023) |
| *Simulium*  *ornatum* | Vosges | 1978 | 4 | June | Summer | 560 | Manual capture | NA | Noirtin et al. (1979) |
|  | Vosges | 1978 | NA | NA | NA | NA | NA | NA | Leclercq (1987) |
|  | Vosges | 1978 | 4 | April, May, June, July | Summer | 560 | Larvae manual  capture | *Onchocerca*  *lienalis* | Noirtin et al. (1981) |
|  | Vosges | 1978 | NA | NA | Spring | NA | NA | NA | Boiteux et al. (1979) |
|  | Marne, Aisne, Vosges | 1988-1992 | NA | February, March, April, May, June, September | Winter,  Spring,  Summer,  Autumn | NA | Larvae and nymph  manual capture | NA | Bouchet et al. (1993) |
|  | Hauts-de-Seine, Puy-de-Dôme, Pyrénées-Orientales, Hautes-Pyrénées, Oise, Paris, Côtes-d'Armor, Vosges, Jura, Isère, Savoie, Corse, Marne, Vienne, Moselle, Meurthe-et-Moselle | 1943-1947 | NA | January, February, March, April, May, June, July, August, September, October, November, December | Winter,  Spring,  Summer,  Autumn | NA | Larvae and adult  manual capture | NA | Grenier (1949) |
|  | Pyrénées-Atlantiques, Hautes-Pyrénées, Pyrénées-Orientales | NA | NA | NA | NA | 0-2500 | Larvae manual  capture | NA | Grenier et al. (1951) |
|  | Pyrénées-Orientales, Pyrénées-Atlantiques | 1947, 1986-1988, 1997 | NA | January, July | Winter,  Summer | NA | NA | NA | INPN (2023) |
|  | Pyrénées-Atlantiques | 1972-1973 | 12 | February, March, April, May, June, July, August, September, October, November, December, January | Winter,  Spring,  Summer,  Autumn | NA | CDC trap,  Larvae manual  capture | NA | Neveu et al. (1978) |
|  | Pyrénées-Atlantiques | 1983-1984 | 5 | April, June, July, September, November | Spring,  Summer,  Autumn | 450-2150 | Drift,  Benthic sampling,  Adult capture | NA | Vinçon et al. (1988) |
|  | Ain, Corrèze, Pyrénées-Atlantiques, Pyrénées-Orientales | 1947, 1986-1988, 1997 | NA | January, July | Winter,  Summer | NA | NA | NA | GBIF (2023) |
|  | All France | NA | NA | NA | NA | NA | NA | NA | 1924 Séguy (1924) |
| *Simulium*  *posticatum* | Eure | 1988-1992 | NA | February, March, April, May, June, September | Winter,  Spring,  Summer,  Autumn | NA | Larvae and nymph  manual capture | NA | Bouchet et al. (1993) |
|  | Pyrénées-Orientales | 2001, 2004-2005, 2013 | NA | January | Winter | NA | NA | NA | INPN (2023) |
|  | Pyrénées-Orientales | 2001, 2004-2005, 2013 | NA | January | Winter | NA | NA | NA | GBIF (2023) |
| *Simulium*  *pseudequinum* | Pyrénées-Atlantiques | 1972-1973 | 12 | February, March, April, May, June, July, August, September, October, November, December, January | Winter,  Spring,  Summer,  Autumn | NA | CDC trap,  Larvae manual  capture | NA | Neveu et al. (1978) |
| *Simulium*  *reptans* | Marne, Aisne, Ardennes | 1988-1992 | NA | February, March, April, May, June, September | Winter,  Spring,  Summer,  Autumn | NA | Larvae and nymph  manual capture | NA | Bouchet et al. (1993) |
|  | Isère | 1943-1947 | NA | January, February, March, April, May, June, July, August, September, October, November, December | Winter,  Spring,  Summer,  Autumn | NA | Larvae and adult  manual capture | NA | Grenier (1949) |
|  | Hautes-Pyrénées | NA | NA | NA | NA | 0-2500 | Larvae manual  capture | NA | Grenier et al. (1951) |
|  | Pyrénées-Atlantiques | 1972-1973 | 12 | February, March, April, May, June, July, August, September, October, November, December, January | Winter,  Spring,  Summer,  Autumn | NA | CDC trap,  Larvae manual  capture | NA | Neveu et al. (1978) |
|  | All France | NA | NA | NA | NA | NA | NA | NA | Séguy (1924) |
| *Simulium*  *rheophilum* | Pyrénées-Atlantiques | 1972-1973 | 12 | February, March, April, May, June, July, August, September, October, November, December, January | Winter,  Spring,  Summer,  Autumn | NA | CDC trap,  Larvae manual  capture | NA | Neveu et al. (1978) |
| *Simulium*  *rubzovianum* | Ille-et-Vilaine, Côtes-d’Armor, Loire-Atlantique, Lozère | 1956-1957, 1960-1961, 1963, 1966, 1970 | NA | March, April, May, July, August, November, December | Winter,  Spring,  Summer,  Autumn | NA | Larvae manual  capture | NA | Beaucournu-Saguez (1977) |
|  | Alpes-Maritimes | 2004 | NA | July | Summer | NA | NA | NA | INPN (2023) |
| *Simulium*  *trifasciatum* | Pyrénées-Orientales | 2001, 2005 | NA | January | Winter | NA | NA | NA | INPN (2023) |
|  | Pyrénées-Atlantiques | 1983-1984 | 5 | April, June, July, September, November | Spring,  Summer,  Autumn | 450-2150 | Drift,  Benthic sampling,  Adult capture | NA | Vinçon et al. (1988) |
|  | Pyrénées-Orientales | 2001, 2005 | NA | January | Winter | NA | NA | NA | GBIF (2023) |
| *Simulium*  *tuberosum* | Pyrénées-Orientales | NA | NA | NA | NA | 0-2500 | Larvae manual  capture | NA | Grenier et al. (1951) |
|  | Pyrénées-Atlantiques | 1983-1984 | 5 | April, June, July, September, November | Spring,  Summer,  Autumn | 450-2150 | Drift,  Benthic sampling,  Adult capture | NA | Vinçon et al. (1988) |
| *Simulium*  *variegatum* | Hautes-Pyrénées, Hautes-Alpes, Isère, Savoie, Jura, Corse, Puy-de-Dôme, Pyrénées-Orientales | 1943-1947 | NA | January, February, March, April, May, June, July, August, September, October, November, December | Winter,  Spring,  Summer,  Autumn | NA | Larvae and adult  manual capture | NA | Grenier (1949) |
|  | Pyrénées-Atlantiques, Hautes-Pyrénées, Ariège | NA | NA | NA | NA | 0-2500 | Larvae manual  capture | NA | Grenier et al. (1951) |
|  | Alpes-de-Haute-Provence, Alpes-Maritimes, Gironde, Pyrénées-Atlantiques, Hautes-Pyrénées, Pyrénées-Orientales | 1946, 1986-1988, 2001-2006, 2008, 2012-2013 | NA | January, February, March, May, July, August, September | Winter,  Spring,  Summer | NA | NA | NA | INPN (2023) |
|  | Pyrénées-Atlantiques | 1972-1973 | 12 | February, March, April, May, June, July, August, September, October, November, December, January | Winter,  Spring,  Summer,  Autumn | NA | CDC trap,  Larvae manual  capture | NA | Neveu et al. (1978) |
|  | Pyrénées-Atlantiques | 1983-1984 | 5 | April, June, July, September, November | Spring,  Summer,  Autumn | 450-2150 | Drift,  Benthic sampling,  Adult capture | NA | Vinçon et al. (1988) |
|  | Alpes-Maritimes, Gironde, Hautes-Pyrénées, Pyrénées-Atlantiques, Pyrénées-Orientales | 1946, 1986-1988, 2001-2006, 2008, 2012-2013 | NA | January, February, March, May, July, August, September | Winter,  Spring,  Summer | NA | NA | NA | GBIF (2023) |
| *Simulium*  *vernum* | Pyrénées-Orientales | 1986-1987 | NA | January | Winter | NA | NA | NA | INPN (2023) |
|  | Pyrénées-Atlantiques | 1983-1984 | 5 | April, June, July, September, November | Spring,  Summer,  Autumn | 450-2150 | Drift,  Benthic sampling,  Adult capture | NA | Vinçon et al. (1988) |
| *Simulium*  *xanthinum* | Pyrénées-Atlantiques | 1983-1984 | 5 | April, June, July, September, November | Spring,  Summer,  Autumn | 450-2150 | Drift,  Benthic sampling,  Adult capture | NA | Vinçon et al. (1988) |

Supplementary Table S7. Captures of sand flies in cattle farms in France between 1909 and 2013 (NA: data not provided).

| Species | Capture | | | | | | | Pathogen | Reference |
| --- | --- | --- | --- | --- | --- | --- | --- | --- | --- |
|  | Department | Year | Study  duration  (Month) | Month | Season | Altitude  (m) | Trapping  method |  |  |
| *Phlebotomus*  *ariasi* | Bouches-du-Rhône | 2009-2011 | 17 | March, April, May, June, July, August, September, October, November | Autumn,  Spring,  Summer | 197 | CDC trap | NA | Cotteaux-Lautard et al. (2016) |
|  | Gard | 1980 | 2 | July, August | Summer | 170-600 | CDC trap  (Bloodfed ♀) | NA | Guy et al. (1984) |
|  | Ardèche, Hérault, Gard, Aveyron, Pyrénées-Orientales | NA | NA | NA | NA | NA | NA | *Leishmania*  *infantum* | Marty et al. (1988) |
|  | Hautes-Pyrénées, Hérault, Gard, Alpes-de-Haute-Provence, Pyrénées-Orientales | NA | NA | NA | NA | NA | NA | NA | Abonnenc (1972) |
|  | Bouches-du-Rhône, Alpes-Maritimes | 2005 | 0.2 | July | Summer | 223, 450 | CDC trap | NA | Charrel et al. (2009) |
|  | Sarthe, Deux-Sèvres, Charente, Gironde, Dordogne, Landes, Pyrénées-Atlantiques, Lot-et-Garonne, Gers, Hautes-Pyrénées, Lot, Tarn-et-Garonne, Haute-Garonne, Tarn, Aveyron, Hérault, Lozère, Haute-Loire, Loire, Rhône, Isère, Ardèche, Gard, Vaucluse, Bouches-du-Rhône, Var, Alpes-de-Haute-Provence, Alpes-Maritimes, Hautes-Alpes | NA | NA | NA | NA | NA | NA | NA | ECDC (2022c) |
|  | Bouches-du-Rhône | 1981 | 6 | June, July, August, September, October, November | Summer,  Autumn | NA | Sticky trap | NA | Gilot et al. (1983) |
|  | Hérault | 1980 | 2 | July, August | Summer | 450 | Oral aspirator | NA | Killick-Kendrick et al. (1984) |
|  | Alpes-Maritimes | NA | NA | NA | NA | 0-3143 | NA | NA | Marty et al. (2007) |
|  | Gers, Haute-Garonne, Hautes-Pyrénées, Ariège, Aude, Pyrénées-Orientales | 2005-2005 | NA | NA | Summer | NA | Sticky trap | NA | Meunier (2007) |
|  | Gard, Pyrénées-Orientales, Ariège, Lot-et-Garonne, Alpes-Maritimes, Vienne, Sarthe, Aveyron, Lozère, Haute-Loire | 1966-1996 | NA | NA | NA | 150-300 | NA | NA | Meunier (2007) |
|  | Alpes-Maritimes, Var, Ardèche, Hautes-Pyrénées, Pyrénées-Atlantiques, Gironde, Dordogne, Charente, Vienne | 1909-1952 | NA | June, July, August, September, October | Summer,  Autumn | NA | NA | NA | Raynal (1954) |
|  | Ariège, Aude, Aveyron, Alpes-de-Haute-Provence, Pyrénées-Atlantiques, Bouches-du-Rhône, Charente, Dordogne, Drôme, Gard, Hautes-Alpes, Haute-Garonne, Hautes-Pyrénées, Hérault, Lot, Lot-et-Garonne, Lozère, Pyrénées-Orientales, Sarthe, Tarn, Var, Vaucluse, Vienne | 1909-1969 | NA | January, February, March, April, May, June, July, August, September, October, November, December | Winter,  Spring,  Summer,  Autumn | 50-1230 | Manual capture,  Sticky trap,  CDC trap,  New Jersey Trap | NA | Rioux et al. (1969) |
|  | Gard | 1977 | 2 | July, August | Summer | 400 | CDC trap | NA | Rioux et al. (1979) |
|  | Gard | 2011 | 7 | May, June, July, August, September, October, November | Spring,  Summer,  Autumn | 175-606 | Sticky trap,  CDC trap | *Leishmania*  *infantum* | Prudhomme et al. (2016) |
|  | Gard | 2011 | 5 | May, June, July, August, September | Spring,  Summer | 228-603 | Sticky trap | *Leishmania*  *infantum* | Prudhomme et al. (2020) |
|  | Gard | 2011-2013 | 24 | April, May, June, July, August, September, October, November | Spring,  Summer,  Autumn | 175-606 | Sticky trap,  CDC trap | *Leishmania*  *infantum* | Prudhomme et al. (2015) |
|  | Pyrénées-Orientales | 1981 | 6 | June, July, August, September, October, November | Spring,  Summer,  Autumn | 150-280 | Sticky trap | NA | Rioux et al. (2013) |
|  | Gard | 2011-2013 | 24 | April, May, June, July, August, September, October, November | Spring,  Summer,  Autumn | 603 | Sticky trap,  CDC trap | *Leishmania*  *infantum* | Alten et al. (2016) |
| *Phlebotomus*  *mascittii* | Gard | 1980 | 2 | July, August | Summer | 170-600 | CDC trap  (Bloodfed ♀) | NA | Guy et al. (1984) |
|  | Gard | NA | NA | NA | NA | NA | NA | NA | Abonnenc (1972) |
|  | Bouches-du-Rhône, Alpes-Maritimes | 2005 | 0.2 | July | Summer | 223, 450 | CDC trap | NA | Charrel et al. (2009) |
|  | Calvados, Corse, Oise, Aisne, Seine-et-Marne, Aube, Haute-Marne, Côte-d'Or, Deux-Sèvres, Vienne, Charente, Charente-Maritime, Corrèze, Allier, Puy-de-Dôme, Loire, Rhône, Aveyron, Lozère, Ardèche, Hautes-Pyrénées, Haute-Garonne, Ariège, Aube, Hérault, Gard, Bouches-du-Rhône, Var, Alpes-Maritimes | NA | NA | NA | NA | NA | NA | NA | ECDC (2022c) |
|  | Hérault | 1980 | 2 | July, August | Summer | 450 | Oral aspirator | NA | Killick-Kendrick et al. (1984) |
|  | Alpes-Maritimes | NA | NA | NA | NA | 0-3143 | NA | NA | Marty et al. (2007) |
|  | Eure, Allier, Puy-de-Dôme | 1966-1996 | NA | NA | NA | 150-300 | NA | NA | Meunier (2007) |
|  | Bouches-du-Rhône, Hautes-Pyrénées, Charente, Vienne, Corrèze, Puy-de-Dôme, Rhône, Côte-d’Or, Haute-Marne, Bas-Rhin, Seine-et-Marne, Oise, Calvados | 1909-1952 | NA | June, July, August, September, October | Summer,  Autumn | NA | NA | NA | Raynal (1954) |
|  | Aveyron, Bas-Rhin, Bouches-du-Rhône, Charente, Charente-Maritime, Corrèze, Corse, Côte-d'Or, Gard, Haute-Garonne, Haute-Marne, Hautes-Pyrénées, Hauts-de-Seine, Hérault, Oise, Puy-de-Dôme, Rhône, Seine-et-Marne, Tarn, Var, Vienne | 1909-1969 | NA | January, February, March, April, May, June, July, August, September, October, November, December | Winter,  Spring,  Summer,  Autumn | 50-1230 | Manual capture,  Sticky trap,  CDC trap,  New Jersey Trap | NA | Rioux et al. (1969) |
|  | Gard | 1977 | 2 | July, August | Summer | 400 | CDC trap | NA | Rioux et al. (1979) |
|  | Gard | 2011-2013 | 24 | April, May, June, July, August, September, October, November | Spring,  Summer,  Autumn | 175-606 | Sticky trap,  CDC trap | NA | Prudhomme et al. (2015) |
|  | Corse, Vienne | NA | NA | NA | NA | NA | NA | NA | GBIF (2023) |
|  | Gard | 2011-2013 | 24 | April, May, June, July, August, September, October, November | Spring,  Summer,  Autumn | 603 | Sticky trap,  CDC trap | NA | Alten et al. (2016) |
| *Phlebotomus*  *papatasi* | Hérault, Bouches-du-Rhône, Vaucluse, Var | NA | NA | NA | NA | NA | NA | NA | ECDC (2022c) |
|  | Var, Bouches-du-Rhône, Vaucluse, Hérault | 1909-1952 | NA | June, July, August, September, October | Summer,  Autumn | NA | NA | NA | Raynal (1954) |
|  | Bouches-du-Rhône, Corse, Gard, Hérault, Var, Vaucluse | 1909-1969 | NA | January, February, March, April, May, June, July, August, September, October, November, December | Winter,  Spring,  Summer,  Autumn | 50-1230 | Manual capture,  Sticky trap,  CDC trap,  New Jersey Trap | NA | Rioux et al. (1969) |
|  | Somme, Hauts-de-Seine, Côte-d'Or, Rhône, Savoie, Hautes-Alpes, Haute-Garonne, Hérault, Bouches-du-Rhône | NA | NA | NA | NA | NA | NA | NA | Séguy (1924) |
| *Phlebotomus*  *perfiliewi* | Alpes-Maritimes | NA | NA | NA | NA | NA | NA | NA | ECDC (2022c) |
|  | Alpes-Maritimes | 1993 | 1 | July | Summer | 60 | CDC trap | NA | Izri et al. (1994) |
|  | Alpes-Maritimes | NA | NA | NA | NA | 0-3143 | NA | *Leishmania*  *infantum* | Marty et al. (2007) |
| *Phlebotomus*  *perniciosus* | Bouches-du-Rhône | 2009-2011 | 17 | March, April, May, June, July, August, September, October, November | Autumn,  Spring,  Summer | 197 | CDC trap | NA | Cotteaux-Lautard et al. (2016) |
|  | Ardèche, Hérault, Gard, Aveyron, Pyrénées-Orientales | NA | NA | NA | NA | NA | NA | *Leishmania*  *infantum* | Marty et al. (1988) |
|  | Corse | NA | NA | NA | NA | NA | NA | Toscana virus | Alexander et al. (2015) |
|  | Bouches-du-Rhône, Alpes-Maritimes | 2005 | 0.2 | July | Summer | 223, 450 | CDC trap | Toscana virus,  Massilia virus | Charrel et al. (2009) |
|  | Haute-Marne, Seine-et-Marne, Val-d’Oise | NA | NA | NA | NA | NA | NA | NA | Dedet et al. (2013) |
|  | Hauts-de-Seine, Seine-Saint-Denis, Val-de-Marne, Paris, Yvelines, Val-d'Oise, Essonne, Eure, Eure-et-Loir, Loiret, Mayenne, Sarthe, Maine-et-Loire, Haute-Marne, Côte-d'Or, Saône-et-Loire, Rhône, Allier, Indre, Vienne, Charente, Charente-Maritime, Gironde, Lot-et-Garonne, Lot, Tarn-et-Garonne, Tarn, Haute-Garonne, Pyrénées-Orientales, Hérault, Lozère, Ardèche, Gard, Bouches-du-Rhône, Var, Alpes-de-Haute-Provence, Alpes-Maritimes | NA | NA | NA | NA | NA | NA | NA | ECDC (2022c) |
|  | Corse | 2000, 2004 | NA | NA | NA | NA | NA | NA | ECDC (2022c) |
|  | Bouches-du-Rhône | 1980 | 0.5 | August | Summer | 0-1041 | Sticky trap | NA | Gilot et al. (1983) |
|  | Bouches-du-Rhône | 1975 | NA | NA | NA | NA | NA | NA | Gilot et al. (1983) |
|  | Var | 1981 | NA | NA | NA | NA | NA | NA | Gilot et al. (1983) |
|  | Bouches-du-Rhône | 1933 | NA | NA | NA | NA | NA | NA | Gilot et al. (1983) |
|  | Bouches-du-Rhône | 1981 | 6 | June, July, August, September, October, November | Summer,  Autumn | NA | Sticky trap | NA | Gilot et al. (1983) |
|  | Alpes-Maritimes | 2012 | NA | May | Spring | NA | NA | NA | INPN (2023) |
|  | Hérault | 1980 | 2 | July, August | Summer | 450 | Oral aspirator | NA | Killick-Kendrick et al. (1984) |
|  | Alpes-Maritimes | NA | NA | NA | NA | 0-3143 | NA | *Leishmania*  *infantum* | Marty et al. (2007) |
|  | Gers, Haute-Garonne, Hautes-Pyrénées, Ariège, Aude, Pyrénées-Orientales | 2005-2005 | NA | NA | Summer | NA | Sticky trap | NA | Meunier (2007) |
|  | Bouches-du-Rhône, Var, Vaucluse, Pyrénées-Orientales, Ariège, Lot-et-Garonne, Alpes-Maritimes, Indre-et-Loire, Mayenne, Maine-et-Loire, Loire, Sarthe, Allier, Puy-de-Dôme | 1966-1996 | NA | NA | NA | 150-300 | NA | NA | Meunier (2007) |
|  | Corse, Alpes-Maritimes, Var, Bouches-du-Rhône, Gard, Ardèche, Pyrénées-Orientales, Haute-Garonne, Lot, Gironde, Dordogne, Charente, Charente-Maritime, Vienne, Indre, Puy-de-Dôme, Rhône, Saône-et-Loire, Côte-d’Or, Loiret, Indre-et-Loire, Maine-et-Loire, Mayenne, Eure-et-Loir, Seine-et-Marne, Yvelines | 1909-1952 | NA | June, July, August, September, October | Summer,  Autumn | NA | NA | NA | Raynal (1954) |
|  | Allier, Alpes-Maritimes, Ardèche, Ariège, Aude, Alpes-de-Haute-Provence, Bouches-du-Rhône, Charente, Charente-Maritime, Corse, Côte-d'Or, Dordogne, Essonne, Eure-et-Loir, Gard, Gironde, Haute-Garonne, Haute-Marne, Hauts-de-Seine, Hérault, Indre, Indre-et-Loire, Loiret, Lot, Lozère, Maine-et-Loire, Mayenne, Puy-de-Dôme, Pyrénées-Orientales, Rhône, Saône-et-Loire, Sarthe, Seine-et-Marne, Tarn, Val-d'Oise, Var, Vienne, Haute-Garonne | 1909-1969 | NA | January, February, March, April, May, June, July, August, September, October, November, December | Winter,  Spring,  Summer,  Autumn | 50-1230 | Manual capture,  Sticky trap,  CDC trap,  New Jersey Trap | NA | Rioux et al. (1969) |
|  | Gard | 2011 | 7 | May, June, July, August, September, October, November | Spring,  Summer,  Autumn | 175-606 | Sticky trap,  CDC trap | *Leishmania*  *infantum* | Prudhomme et al. (2016) |
|  | Gard | 2011 | 5 | May, June, July, August, September | Spring,  Summer | 228-603 | Sticky trap | *Leishmania*  *infantum* | Prudhomme et al. (2020) |
|  | Gard | 2011-2013 | 24 | April, May, June, July, August, September, October, November | Spring,  Summer,  Autumn | 175-606 | Sticky trap,  CDC trap | *Leishmania*  *infantum* | Prudhomme et al. (2015) |
|  | Pyrénées-Orientales | 1981 | 6 | June, July, August, September, October, November | Spring,  Summer,  Autumn | 150-280 | Sticky trap | NA | Rioux et al. (2013) |
|  | Vienne, Alpes-Maritimes | 2012 | NA | May | Spring | NA | NA | NA | GBIF (2023) |
|  | Gard | 2011-2013 | 24 | April, May, June, July, August, September, October, November | Spring,  Summer,  Autumn | 603 | Sticky trap,  CDC trap | NA | Alten et al. (2016) |
|  | Hauts-de-Seine, Oise, Alpes-Maritimes, Bouches-du-Rhône, Puy-de-Dôme, Rhône, Corse | NA | NA | NA | NA | NA | NA | NA | Séguy (1924) |
| *Phlebotomus*  *sergenti* | Bouches-du-Rhône, Ariège | NA | NA | NA | NA | NA | NA | NA | ECDC (2022c) |
|  | Bouches-du-Rhône | 1909-1952 | NA | June, July, August, September, October | Summer,  Autumn | NA | NA | NA | Raynal (1954) |
|  | Bouches-du-Rhône | 1909-1969 | NA | January, February, March, April, May, June, July, August, September, October, November, December | Winter,  Spring,  Summer,  Autumn | 50-1230 | Manual capture,  Sticky trap,  CDC trap,  New Jersey Trap | NA | Rioux et al. (1969) |
|  | Bouches-du-Rhône | NA | NA | NA | NA | NA | NA | NA | Séguy (1924) |
| *Sergentomyia*  *minuta* | Bouches-du-Rhône | 2009-2011 | 17 | March, April, May, June, July, August, September, October, November | Autumn,  Spring,  Summer | 197 | CDC trap | NA | Cotteaux-Lautard et al. (2016) |
|  | Bouches-du-Rhône, Alpes-Maritimes | 2005 | 0.2 | July | Summer | 223, 450 | CDC trap | NA | Charrel et al. (2009) |
|  | Bouches-du-Rhône | 1981 | 6 | June, July, August, September, October, November | Summer,  Autumn | NA | Sticky trap | NA | Gilot et al. (1983) |
|  | Hérault | 1980 | 2 | July, August | Summer | 450 | Oral aspirator | NA | Killick-Kendrick et al. (1984) |
|  | Alpes-Maritimes | NA | NA | NA | NA | 0-3143 | NA | NA | Marty et al. (2007) |
|  | Allier, Puy-de-Dôme | 1966-1996 | NA | NA | NA | 150-300 | NA | NA | Meunier (2007) |
|  | Var, Bouches-du-Rhône, Pyrénées-Orientales | 1909-1952 | NA | June, July, August, September, October | Summer,  Autumn | NA | NA | NA | Raynal (1954) |
|  | Ardèche, Aude, Bouches-du-Rhône, Corse, Gard, Haute-Garonne, Hérault, Lozère, Pyrénées-Orientales, Var, Tarn | 1909-1969 | NA | January, February, March, April, May, June, July, August, September, October, November, December | Winter,  Spring,  Summer,  Autumn | 50-1230 | Manual capture,  Sticky trap,  CDC trap,  New Jersey Trap | NA | Rioux et al. (1969) |
|  | Gard | 2011-2013 | 24 | April, May, June, July, August, September, October, November | Spring,  Summer,  Autumn | 175-606 | Sticky trap,  CDC trap | NA | Prudhomme et al. (2015) |
|  | Pyrénées-Orientales | 1981 | 6 | June, July, August, September, October, November | Spring,  Summer,  Autumn | 140-280 | Sticky trap | NA | Rioux et al. (2013) |
|  | Corse | NA | NA | NA | NA | NA | NA | NA | GBIF (2023) |
|  | Gard | 2011-2013 | 24 | April, May, June, July, August, September, October, November | Spring,  Summer,  Autumn | 603 | Sticky trap,  CDC trap | NA | Alten et al. (2016) |
|  | Bouches-du-Rhône | NA | NA | NA | NA | NA | NA | NA | Séguy (1924) |

Supplementary Table S8. Captures of mosquitoes in cattle farms in France between 1758 and 2022 (NA: data not provided).

| Species | Capture | | | | | | | Pathogen | Reference |
| --- | --- | --- | --- | --- | --- | --- | --- | --- | --- |
|  | Department | Year | Study  duration  (month) | Month | Season | Altitude  (m) | Trapping  method |  |  |
| *Aedes*  *aegypti* | Corse | 1944, 1946-1950, 1952 | NA | NA | NA | NA | NA | NA | Aitken (1954) |
|  | Gironde, Var, Finistère, Loire-Atlantique, Bouches-du-Rhône | 1902-1921 | NA | January, February, March, April, May, June, July, August, September, October, November, December | Winter,  Spring,  Summer,  Autumn | NA | NA | NA | Séguy (1923) |
|  | Var, Gironde | NA | NA | NA | NA | NA | NA | NA | Séguy (1924) |
| *Aedes*  *albopictus* | Lot-et-Garonne, Bouches-du-Rhône, Var, Alpes-Maritimes, Haute-Garonne, Tarn, Aude, Pyrénées-Orientales, Hérault, Gard, Vaucluse, Alpes-de-Haute-Provence, Ardèche, Drôme, Isère, Rhône, Loire | 2011, 2015 | NA | NA | NA | NA | NA | NA | Alexander et al. (2015) |
|  | All France | NA | NA | NA | NA | NA | NA | NA | ECDC (2022b) |
|  | Ain, Aisne, Alpes-de-Haute-Provence, Alpes-Maritimes, Ardèche, Ariège, Aude, Aveyron, Bas-Rhin, Bouches-du-Rhône, Charente-Maritime, Corrèze, Corse, Dordogne, Drôme, Gard, Gers, Gironde, Hérault, Haute-Garonne, Haute-Savoie, Hautes-Pyrénées, Haut-Rhin, Indre, Isère, Landes, Loir-et-Cher, Lot, Lot-et-Garonne, Lozère, Maine-et-Loire, Orne, Paris, Puy-de-Dôme, Pyrénées-Atlantiques, Pyrénées-Orientales, Rhône, Saône-et-Loire, Savoie, Seine-et-Marne, Tarn, Tarn-et-Garonne, Val-de-Marne, Val-d'Oise, Var, Vaucluse, Vendée | 1999-2000, 2009-2022 | NA | January, February, April, May, June, July, August, September, October, November | Winter,  Spring,  Summer,  Autumn | NA | NA | NA | INPN (2023) |
|  | Ain, Aisne, Alpes-de-Haute-Provence, Hautes-Alpes, Alpes-Maritimes, Ardèche, Ariège, Aude, Aveyron, Bouches-du-Rhône, Cantal, Charente, Charente-Maritime, Cher, Corrèze, Corse, Côte-d'Or, Dordogne, Doubs, Drôme, Gard, Haute-Garonne, Gers, Gironde, Hérault, Indre, Indre-et-Loire, Isère, Jura, Landes, Loire, Loire-Atlantique, Lot, Lot-et-Garonne, Lozère, Maine-et-Loire, Mayenne, Nièvre, Puy-de-Dôme, Pyrénées-Atlantiques, Hautes-Pyrénées, Pyrénées-Orientales, Bas-Rhin, Haut-Rhin, Rhône, Saône-et-Loire, Savoie, Haute-Savoie, Paris, Seine-et-Marne, Yvelines, Deux-Sèvres, Tarn, Tarn-et-Garonne, Var, Vaucluse, Vendée, Vienne, Haute-Vienne, Territoire-de-Belfort, Essonne, Hauts-de-Seine, Seine-Saint-Denis, Val-de-Marne | 2021 | NA | NA | NA | NA | NA | NA | Martinet (2021) |
|  | Bas-Rhin | 2017-2020 | NA | January, February, March, April, May, June, July, August, September, October, November, December | Winter,  Spring,  Summer,  Autumn | NA | CDC trap,  BG-Sentinel,  Larval capture,  Aspirator | NA | Martinet (2021) |
|  | Bas-Rhin | 2018-2019 | NA | NA | NA | NA | BG-Sentinel,  Human landing  capture | NA | Martinet (2021) |
|  | Alpes-Maritimes | NA | NA | NA | NA | NA | NA | NA | Martinet (2021) |
|  | Alpes-Maritimes, Var, Alpes-de-Haute-Provence, Bouches-du-Rhône, Pyrénées-Orientales, Saône-et-Loire, Corse | 2011 | NA | September | Autumn | NA | Ovitrap | Chikingunya virus | Medlock et al. (2012) |
|  | Alpes-Maritimes, Var | NA | NA | NA | NA | NA | NA | NA | Mehlhorn (2012) |
|  | Corrèze, Lot, Tarn-et-Garonne, Haute-Garonne, Aude, Pyrénées-Orientales, Aveyron, Hérault, Gard, Lozère, Ardèche, Bouches-du-Rhône, Var, Alpes-Maritimes, Corse, Alpes-de-Haute-Provence, Hautes-Alpes, Savoie, Isère, Drôme, Haute-Savoie, Ain, Rhône, Loire, Saône-et-Loire, Côte-d'Or, Jura, Doubs | 2006-2012 | 36 | June, July, August, September, October, November | Summer,  Autumn | NA | Ovitrap | NA | Roche et al. (2015) |
|  | Orne | 1999 | 0.1 | October | Autumn | 145 | Larvae capture | NA | Schaffner et al. (2000) |
|  | Corse, Alpes-Maritimes | 1997-2007 | NA | NA | NA | NA | NA | Chikungunya virus,  Dengue virus | Takken et al. (2007) |
|  | Alpes-Maritimes, Corse, Var, Bouches-du-Rhône | 2004, 2006-2007, 2009 | NA | NA | NA | NA | NA | Chikungunya virus,  Dengue virus | Viennet (2011) |
|  | Ain, Aisne, Alpes-de-Haute-Provence, Alpes-Maritimes, Ardèche, Ariège, Aude, Aveyron, Bas-Rhin, Bouches-du-Rhône, Calvados, Charente, Charente-Maritime, Corrèze, Corse, Dordogne, Doubs, Drôme, Essonne, Gard, Gers, Gironde, Haute-Garonne, Hautes-Alpes, Haute-Savoie, Hautes-Pyrénées, Haut-Rhin, Hérault, Indre, Isère, Jura, Landes, Loire, Loire-Atlantique, Loiret, Loir-et-Cher, Lot, Lot-et-Garonne, Lozère, Maine-et-Loire, Moselle, Oise, Orne, Paris, Puy-de-Dôme, Pyrénées-Atlantiques, Pyrénées-Orientales, Rhône, Saône-et-Loire, Savoie, Seine-et-Marne, Tarn, Tarn-et-Garonne, Val-de-Marne, Var, Vaucluse, Vendée, Vienne | 1999-2000, 2009-2022 | NA | January, February, April, May, June, July, August, September, October, November | Winter,  Spring,  Summer,  Autumn | NA | NA | NA | GBIF (2023) |
| *Aedes*  *annulipes* | Loire-Atlantique | 1993 | NA | NA | NA | NA | Larvae capture | NA | Brutus et al. (1993) |
|  | Bouches-du-Rhône, Hérault | 2004 | 6 | May, June, July, August, September, October | Spring,  Summer,  Autumn | 1, 25 | Hosted capture | NA | Balenghien et al. (2006) |
|  | Indre-et-Loire | 1936-1943 | NA | January, February, March, April, May, June, July, August, September, October, November, December | Winter,  Spring,  Summer,  Autumn | NA | NA | NA | Callot et al. (1944) |
|  | Ain, Isère, Rhône, Savoie, Vaucluse, Hautes-Alpes | 1966-1976 | NA | NA | NA | NA | NA | NA | Gilot et al. (1976) |
|  | Moselle, Haute-Garonne | 1995, 2012 | NA | May, June, September | Spring,  Summer | NA | NA | NA | INPN (2023) |
|  | Marne, Haute-Marne | 2017-2020 | NA | January, February, March, April, May, June, July, August, September, October, November, December | Winter,  Spring,  Summer,  Autumn | NA | CDC trap,  BG-Sentinel,  Larvae capture,  Aspirator | NA | Martinet (2021) |
|  | Ain, Hautes-Alpes, Ardennes, Charente-Maritime, Cote d'Or, Creuse, Indre-et-Loire, Isère, Loire-Atlantique, Oise, Bas-Rhin, Haut-Rhin, Rhône, Haute-Saône, Savoie, Seine-et-Marne, Vienne, Yvelines, Territoire de Belfort, Paris | NA | NA | NA | NA | NA | NA | NA | Moussiegt (1986) |
|  | Paris, Hauts-de-Seine, Seine-Saint-Denis, Val-de-Marne, Yvelines, Essonne, Val-d'Oise, Seine-et-Marne, Oise, Ardennes, Indre-et-Loire, Vienne, Isère, Creuse, Côte-d’Or, Haute-Saône, Ain, Rhône, Savoie, Bas-Rhin, Haut-Rhin | NA | NA | NA | NA | NA | NA | NA | Rageau et al. (1970b) |
|  | Ardennes, Yvelines, Hauts-de-Seine, Seine-et-Marne, Côte-d'Or, Haute-Saône, Aube | 1902-1921 | NA | January, February, March, April, May, June, July, August, September, October, November, December | Winter,  Spring,  Summer,  Autumn | NA | NA | NA | Séguy (1923) |
|  | All France | NA | NA | NA | NA | NA | NA | NA | Séguy (1924) |
|  | Haute-Garonne, Essonne, Moselle, Oise | 1995, 2012 | NA | May, June, September | Spring,  Summer | NA | NA | NA | GBIF (2023) |
| *Aedes*  *berlandi* | Corse | 1944, 1946-1950, 1952 | NA | NA | NA | NA | NA | NA | Aitken (1954) |
|  | Ain, Isère, Rhône, Savoie | 1966-1976 | NA | NA | NA | NA | NA | NA | Gilot et al. (1976) |
|  | Hérault | 1955 | 1 | June | Summer | 27 | NA | NA | Harant et al. (1955) |
|  | Aude, Bouches-du-Rhône, Corse, Gard, Haute-Garonne, Hérault, Pyrénées-Orientales, Rhône, Var, Vaucluse, Paris | NA | NA | NA | NA | NA | NA | NA | Moussiegt (1986) |
|  | Paris, Hauts-de-Seine, Seine-Saint-Denis, Val-de-Marne, Rhône, Haute-Garonne, Pyrénées-Orientales, Aude, Hérault, Gard, Bouches-du-Rhône, Var, Vaucluse, Corse | NA | NA | NA | NA | NA | NA | NA | Rageau et al. (1970b) |
|  | Hérault, Gard, Bouches-du-Rhône | 1965 | 1 | April | Spring | NA | Egg collection | NA | Rioux et al. (1967) |
|  | Paris, Hauts-de-Seine, Var | 1902-1921 | NA | January, February, March, April, May, June, July, August, September, October, November, December | Winter,  Spring,  Summer,  Autumn | NA | NA | NA | Séguy (1923) |
|  | Bouches-du-Rhône, Gard | NA | NA | NA | NA | NA | Larvae and  adult capture | NA | Rioux et al. (1955) |
|  | Hérault | NA | NA | NA | NA | NA | NA | NA | GBIF (2023) |
| *Aedes*  *cantans* | Morbihan, Loire-Atlantique | 1993 | NA | NA | NA | NA | Larvae capture | NA | Brutus et al. (1993) |
|  | Corse | 1944, 1946-1950, 1952 | NA | NA | NA | NA | NA | NA | Aitken (1954) |
|  | Indre-et-Loire | 1936-1943 | NA | January, February, March, April, May, June, July, August, September, October, November, December | Winter,  Spring,  Summer,  Autumn | NA | NA | NA | Callot et al. (1944) |
|  | Savoie | 1983 | 0.03 | July | Summer | 280-325,  286-656 | Human landing  capture | *Spiroplasma* sp. | Chastel et al. (1985) |
|  | Ain, Isère, Rhône, Savoie, Vaucluse, Hautes-Alpes | 1966-1976 | NA | NA | NA | NA | NA | NA | Gilot et al. (1976) |
|  | Dordogne, Gironde, Savoie | 2000, 2004, 2010, 2014-2017 | NA | January, March, June, July | Winter,  Spring,  Summer | NA | NA | NA | INPN (2023) |
|  | Marne, Ardennes, Haute-Marne, Moselle | 2017-2020 | NA | January, February, March, April, May, June, July, August, September, October, November, December | Winter,  Spring,  Summer,  Autumn | NA | CDC trap,  BG-Sentinel,  Larvae capture,  Aspirator | NA | Martinet (2021) |
|  | Marne | 2018-2019 | NA | NA | NA | NA | BG-Sentinel,  Human landing  capture | NA | Martinet (2021) |
|  | Marne | 2019 | 2 | April | Spring | NA | Larvae capture | NA | Martinet (2021) |
|  | Ain, Alpes-Maritimes, Ardèche, Ardennes, Calvados, Charente-Maritime, Corrèze, Corse, Côte d'Or, Creuse, Drôme, Haute-Garonne, Gironde, Hérault, Ille-et-Vilaine, Indre-et-Loire, Isère, Jura, Loire, Haute-Loire, Loire-Atlantique, Mayenne, Nièvre, Oise, Puy-de-Dôme, Pyrénées-Atlantiques, Bas-Rhin, Haut-Rhin, Rhône, Savoie, Seine-et-Marne, Deux-Sèvres, Var, Vaucluse, Vendée, Vienne, Haute-Vienne, Yvelines, Paris | NA | NA | NA | NA | NA | NA | NA | Moussiegt (1986) |
|  | Paris, Hauts-de-Seine, Seine-Saint-Denis, Val-de-Marne, Yvelines, Essonne, Val-d'Oise, Seine-et-Marne, Oise, Ardennes, Calvados, Bas-Rhin, Haut-Rhin, Nièvre, Ille-et-Vilaine, Mayenne, Loire-Atlantique, Indre-et-Loire, Vienne, Creuse, Haute-Garonne, Corse, Alpes-Maritimes, Haute-Loire, Rhône, Ain, Isère, Savoie | NA | NA | NA | NA | NA | NA | NA | Rageau et al. (1970b) |
|  | Aveyron, Lot, Dordogne, Ariège, Haute-Garonne, Tarn, Tarn-et-Garonne, Lot-et-Garonne, Gers | NA | NA | March, April, May, June, July, August, September, October, November, December, January | Spring,  Summer,  Autumn,  Winter | NA | Larvae capture | NA | Ruffié (1957) |
|  | Oise, Yonne, Seine-et-Marne, Aube, Yvelines, Val-de-Marne, Hauts-de-Seine, Mayenne, Nièvre, Creuse, Vienne, Var | 1902-1921 | NA | January, February, March, April, May, June, July, August, September, October, November, December | Winter,  Spring,  Summer,  Autumn | NA | NA | NA | Séguy (1923) |
|  | All France | NA | NA | NA | NA | NA | NA | NA | Séguy (1924) |
|  | Landes, Gironde, Savoie, Dordogne, Oise, Vendée | 2000, 2004, 2010, 2014-2017 | NA | March, June, July | Spring,  Summer | NA | NA | NA | GBIF (2023) |
| *Aedes*  *cantans /*  *annulipes* | Savoie | 1983-1985 | 12 | May, June, July | Summer | 310, 1538 | Human landing  capture,  Net capture | NA | Chastel et al. (1987) |
| *Aedes*  *caspius* | Morbihan, Loire-Atlantique | 1993 | NA | NA | NA | NA | Larvae capture | NA | Brutus et al. (1993) |
|  | Bouches-du-Rhône | 1926 | 1 | July | Summer | 24 | NA | NA | Brumpt (1942) |
|  | Corse | 1944, 1946-1950, 1952 | NA | NA | NA | NA | NA | NA | Aitken (1954) |
|  | Bouches-du-Rhône, Hérault | 2004 | 6 | May, June, July, August, September, October | Spring,  Summer,  Autumn | 1, 25 | Hosted capture | NA | Balenghien et al. (2006) |
|  | Bouches-du-Rhône | 2011-2014 | 48 | January, February, March, April, May, June, July, August, September, October, November, December | Winter,  Spring,  Summer,  Autumn | 0-3 | CDC trap  with CO2 | NA | Chaskopoulou et al. (2016) |
|  | Finistère, Côtes-d’Armor, Morbihan, Ille-et-Vilaine, Loire-Atlantique, Vendée, Charente-Maritime, Gironde, Dordogne, Manche, Calvados, Seine-Maritime, Oise, Meuse, Meurthe-et-Moselle, Moselle, Bas-Rhin, Haut-Rhin, Territoire-de-Belfort, Landes, Pyrénées-Atlantiques, Pyrénées-Orientales, Aude, Hérault, Gard, Bouches-du-Rhône, Var, Alpes-Maritimes, Ardèche, Drôme, Isère, Rhône, Ain, Yvelines, Corse | NA | NA | NA | NA | NA | NA | NA | ECDC (2022b) |
|  | Isère, Rhône, Drôme | 1966-1976 | NA | NA | NA | NA | NA | NA | Gilot et al. (1976) |
|  | Charente-Maritime, Vendée, Loire-Atlantique, Gironde, Morbihan, Landes, Vienne | 1989 | NA | NA | NA | NA | Larvae capture | NA | Guilloteau (1990) |
|  | Bouches-du-Rhône | 1964 | 3 |  |  |  | NA | NA | Hannoun et al. (1964) |
|  | Hérault, Calvados, Pas-de-Calais, Gironde | 2010, 2013-2014, 2021 | NA | April, July, August, October | Spring,  Summer,  Autumn | NA | NA | NA | INPN (2023) |
|  | Bouches-du-Rhône | 2006 | 11 | February, March, April, May, June, July, August, September, October, November, December | Winter,  Spring,  Summer,  Autumn | 0-50 | CDC trap  with CO2,  Ovitrap,  Human landing  capture | NA | l'Ambert et al. (2012) |
|  | Loire-Atlantique | 1988 | 4 | May, June, August, September | Summer | 6-10 | Human landing  capture,  Hosted capture | *Spiroplasma*  *cantharis* sp. | Le Goff et al. (1990) |
|  | Bouches-du-Rhône | 1920 | NA | August | Summer | NA | NA | NA | Leger (1920) |
|  | Bouches-du-Rhône | NA | NA | NA | NA | NA | NA | NA | Martinet (2021) |
|  | Bouches-du-Rhône | 1964-1968 | 10 | February, March, April, May, June, July, August, September, October, December | Winter,  Spring,  Summer,  Autumn | NA | NA | NA | Mouchet et al. (1970) |
|  | Alpes-Maritimes, Aude, Bouches-du-Rhône, Calvados, Charente-Maritime, Corse, Côtes-d’Armor, Drôme, Finistère, Gard, Gironde, Hérault, Ille-et-Vilaine, Isère, Landes, Loire-Atlantique, Manche, Morbihan, Moselle, Oise, Pyrénées-Atlantiques, Pyrénées-Orientales, Bas-Rhin, Haut-Rhin, Rhône, Seine-Maritime, Seine-et-Marne, Var, Vendée, Yvelines, Paris, Territoire-de-Belfort | NA | NA | NA | NA | NA | NA | NA | Moussiegt (1986) |
|  | Bouches-du-Rhône | 2005-2007 | NA | NA | NA | NA | Larvae capture | NA | Moutailler et al. (2008) |
|  | Gard, Bouches-du-Rhône | 2005 | 8 | March, April, May, June, July, August, September, October | Spring,  Summer,  Autumn | NA | CDC trap | NA | Ponçon (2008) |
|  | Hérault, Gard, Bouches-du-Rhône | 1953-1966 | NA | January, February, March, April, May, June, July, August, September, October, November, December | Winter,  Spring,  Summer,  Autumn | NA | Larvae capture,  CDC trap | NA | Rageau et al. (1967) |
|  | Bas-Rhin, Haut-Rhin, Paris, Hauts-de-Seine, Seine-Saint-Denis, Val-de-Marne, Yvelines, Essonne, Val-d'Oise, Seine-et-Marne, Oise, Corse, Seine-Maritime, Calvados, Manche, Finistère, Côtes-d’Armor, Morbihan, Ille-et-Vilaine, Loire-Atlantique, Vendée, Charente-Maritime, Gironde, Landes, Pyrénées-Atlantiques, Pyrénées-Orientales, Aude, Hérault, Gard, Bouches-du-Rhône, Var, Alpes-Maritimes | NA | NA | NA | NA | NA | NA | NA | Rageau et al. (1970b) |
|  | Hérault, Gard, Bouches-du-Rhône | 1965 | 1 | April | Spring | NA | Egg collection | NA | Rioux et al. (1967) |
|  | Morbihan, Eure-et-Loir, Loire-Atlantique, Hérault, Charente-Maritime | 1902-1921 | NA | January, February, March, April, May, June, July, August, September, October, November, December | Winter,  Spring,  Summer,  Autumn | NA | NA | *Dirofilaria*  *immitis* | Séguy (1923) |
|  | All France | NA | NA | NA | NA | NA | NA | NA | Séguy (1924) |
|  | Bouches-du-Rhône, Gard | NA | NA | NA | NA | NA | Larvae and  adult capture | NA | Rioux et al. (1955) |
|  | Alpes-de-Haute-Provence, Seine-Saint-Denis, Pas-de-Calais, Loire-Atlantique, Hérault, Oise, Var, Gironde, Calvados, Gard, Bouches-du-Rhône | 2005, 2010, 2021 | NA | April, June, July, October | Spring,  Summer,  Autumn | NA | NA | NA | GBIF (2023) |
| *Aedes*  *cataphylla* | Savoie | 1983-1985 | 12 | May, June, July | Summer | 310, 1538 | Human landing  capture,  Net capture | NA | Chastel et al. (1987) |
|  | Ain, Isère, Rhône, Savoie, Drôme, Alpes-Maritimes | 1966-1976 | NA | NA | NA | NA | NA | NA | Gilot et al. (1976) |
|  | Savoie | 2014, 2016 | NA | January | Winter | NA | NA | NA | INPN (2023) |
|  | Ain, Alpes-de-Haute-Provence, Hautes-Alpes, Alpes-Maritimes, Doubs, Drôme, Indre-et-Loire, Isère, Jura, Puy-de-Dôme, Pyrénées-Orientales, Savoie, Haute-Savoie, Var | NA | NA | NA | NA | NA | NA | NA | Moussiegt (1986) |
|  | Jura, Doubs, Ain, Haute-Savoie, Savoie, Isère, Hautes-Alpes, Puy-de-Dôme, Pyrénées-Orientales | NA | NA | NA | NA | NA | NA | NA | Rageau et al. (1970b) |
|  | Pyrénées-Orientales | NA | NA | NA | NA | NA | NA | NA | Rioux et al. (1967) |
|  | Savoie | 2014, 2016 | NA | NA | NA | NA | NA | NA | GBIF (2023) |
| *Aedes*  *cinereus* | Corse | 1944, 1946-1950, 1952 | NA | NA | NA | NA | NA | NA | Aitken (1954) |
|  | Indre-et-Loire | 1936-1943 | NA | January, February, March, April, May, June, July, August, September, October, November, December | Winter,  Spring,  Summer,  Autumn | NA | NA | NA | Callot et al. (1944) |
|  | Savoie | 1983 | 0.03 | July | Summer | 280-325,  286-656 | Human landing  capture | NA | Chastel et al. (1985) |
|  | Ain, Isère, Rhône, Savoie | 1966-1976 | NA | NA | NA | NA | NA | NA | Gilot et al. (1976) |
|  | Aube, Savoie | 2012, 2015-2017 | NA | January, June | Winter,  Summer | NA | NA | NA | INPN (2023) |
|  | Marne, Haute-Marne, Moselle | 2017-2020 | NA | January, February, March, April, May, June, July, August, September, October, November, December | Winter,  Spring,  Summer,  Autumn | NA | CDC trap,  BG-Sentinel,  Larvae capture,  Aspirator | NA | Martinet (2021) |
|  | Marne | 2018-2019 | NA | NA | NA | NA | BG-Sentinel,  Human landing  capture | NA | Martinet (2021) |
|  | Ain, Ardennes, Ariège, Corse, Creuse, Dordogne, Drôme, Indre-et-Loire, Isère, Jura, Loir-et-Cher, Oise, Pyrénées-Atlantiques, Pyrénées-Orientales, Bas-Rhin, Haut-Rhin, Rhône, Savoie, Seine-et-Marne, Vienne, Yvelines, Territoire-de-Belfort, Paris | NA | NA | NA | NA | NA | NA | NA | Moussiegt (1986) |
|  | Paris, Hauts-de-Seine, Seine-Saint-Denis, Val-de-Marne, Yvelines, Essonne, Val-d'Oise, Oise, Bas-Rhin, Haut-Rhin, Indre-et-Loire, Vienne, Creuse, Dordogne, Ain, Isère, Pyrénées-Atlantiques, Ariège, Pyrénées-Orientales | NA | NA | NA | NA | NA | NA | NA | Rageau et al. (1970b) |
|  | Aveyron, Lot, Dordogne, Ariège, Haute-Garonne, Tarn, Tarn-et-Garonne, Lot-et-Garonne, Gers | NA | NA | March, April, May, June, July, August, September, October, November, December, January | Spring,  Summer,  Autumn,  Winter | NA | Larvae capture | NA | Ruffié (1957) |
|  | All France | 1902-1921 | NA | January, February, March, April, May, June, July, August, September, October, November, December | Winter,  Spring,  Summer,  Autumn | NA | NA | NA | Séguy (1923) |
|  | Gironde, Savoie, Aube, Marne | 2012, 2015-2018 | NA | June | Summer | NA | NA | NA | GBIF (2023) |
| *Aedes*  *cinereus /*  *geminus* | Savoie | 1983-1985 | 12 | May, June, July | Summer | 310, 1538 | Human landing  capture,  Net capture | NA | Chastel et al. (1987) |
| *Aedes*  *communis* | Loire-Atlantique | 1993 | NA | NA | NA | NA | Larvae capture | NA | Brutus et al. (1993) |
|  | Bouches-du-Rhône | 1926 | 1 | July | Summer | 24 | NA | NA | Brumpt (1942) |
|  | Corse | 1944, 1946-1950, 1952 | NA | NA | NA | NA | NA | NA | Aitken (1954) |
|  | Indre-et-Loire | 1936-1943 | NA | January, February, March, April, May, June, July, August, September, October, November, December | Winter,  Spring,  Summer,  Autumn | NA | NA | NA | Callot et al. (1944) |
|  | Ain, Isère, Rhône, Savoie | 1966-1976 | NA | NA | NA | NA | NA | NA | Gilot et al. (1976) |
|  | Gironde, Dordogne, Isère | 1969, 2004, 2010, 2014 | NA | April, May, July, August | Spring,  Summer | NA | NA | NA | INPN (2023) |
|  | Marne | 2017-2020 | NA | January, February, March, April, May, June, July, August, September, October, November, December | Winter,  Spring,  Summer,  Autumn | NA | CDC trap,  BG-Sentinel,  Larvae capture,  Aspirator | NA | Martinet (2021) |
|  | Marne | 2019 | 2 | April | Spring | NA | Larvae capture | NA | Martinet (2021) |
|  | Ain, Hautes-Alpes, Ardennes, Bouches-du-Rhône, Corse, Côte-d'Or, Gironde, Hérault, Ille-et-Vilaine, Indre-et-Loire, Isère, Landes, Loire, Haute-Loire, Loire-Atlantique, Oise, Puy-de-Dôme, Pyrénées-Atlantiques, Pyrénées-Orientales, Bas-Rhin, Haut-Rhin, Rhône, Saône-et-Loire, Savoie, Seine-Maritime, Seine-et-Marne, Vosges, Yvelines, Territoire-de-Belfort, Paris | NA | NA | NA | NA | NA | NA | NA | Moussiegt (1986) |
|  | Paris, Hauts-de-Seine, Seine-Saint-Denis, Val-de-Marne, Yvelines, Essonne, Val-d'Oise, Seine-et-Marne, Oise, Seine-Maritime, Bas-Rhin, Haut-Rhin, Haute-Garonne, Saône-et-Loire, Rhône, Isère, Savoie, Hautes-Alpes, Puy-de-Dôme, Haute-Loire, Gironde, Pyrénées-Orientales, Corse, Bouches-du-Rhône | NA | NA | NA | NA | NA | NA | NA | Rageau et al. (1970b) |
|  | Yonne, Hauts-de-Seine, Yvelines, Seine-et-Marne, Aube, Essonne | 1902-1921 | NA | January, February, March, April, May, June, July, August, September, October, November, December | Winter,  Spring,  Summer,  Autumn | NA | NA | NA | Séguy (1923) |
|  | All France | NA | NA | NA | NA | NA | NA | NA | Séguy (1924) |
|  | Gironde, Isère, Dordogne | 1969, 2004, 2010, 2014 | NA | April, May, July, August | Spring,  Summer | NA | NA | NA | GBIF (2023) |
| *Aedes*  *detritus /*  *coluzzii* | Morbihan, Loire-Atlantique | 1993 | NA | NA | NA | NA | Larvae capture | NA | Brutus et al. (1993) |
|  | Bouches-du-Rhône | 1926 | 1 | July | Summer | 24 | NA | NA | Brumpt (1942) |
|  | Corse | 1944, 1946-1950, 1952 | NA | NA | NA | NA | NA | NA | Aitken (1954) |
|  | Bouches-du-Rhône, Hérault | 2004 | 6 | May, June, July, August, September, October | Spring,  Summer,  Autumn | 1, 25 | Hosted capture | NA | Balenghien et al. (2006) |
|  | Meuse, Moselle, Meurthe-et-Moselle, Haut-Rhin, Alpes-Maritimes, Var, Bouches-du-Rhône, Gard, Hérault, Aude, Tarn, Pyrénées-Atlantiques, Pyrénées-Atlantiques, Landes, Gironde, Charente-Maritime, Vendée, Loire-Atlantique, Ille-et-Vilaine, Manche, Calvados, Morbihan, Côtes-d'Armor, Finistère | NA | NA | NA | NA | NA | NA | NA | ECDC (2022b) |
|  | Charente-Maritime, Vendée, Loire-Atlantique, Gironde, Morbihan, Landes, Vienne | 1989 | NA | NA | NA | NA | Larvae capture | NA | Guilloteau (1990) |
|  | Bouches-du-Rhône | 2010 | NA | December | Winter | NA | NA | NA | INPN (2023) |
|  | Bouches-du-Rhône | 2006 | 11 | February, March, April, May, June, July, August, September, October, November, December | Winter,  Spring,  Summer,  Autumn | 0-50 | CDC trap  with CO2,  Ovitrap,  Human landing  capture | NA | l'Ambert et al. (2012) |
|  | Loire-Atlantique | 1988 | 4 | May, June, August, September | Summer | 6-10 | Human landing  capture,  Hosted capture | *Spiroplasma*  *sabaudiense,*  *Spiroplasma*  *cantharis* sp. | Le Goff et al. (1990) |
|  | Alpes-Maritimes, Aude, Bouches-du-Rhône, Calvados, Charente-Maritime, Corse, Côtes-d’Armor, Finistère, Gard, Gironde, Hérault, Ille-et-Vilaine, Landes, Loire-Atlantique, Manche, Morbihan, Moselle, Pyrénées-Atlantiques, Pyrénées-Orientales, Haut-Rhin, Var, Vendée | NA | NA | NA | NA | NA | NA | NA | Moussiegt (1986) |
|  | Bouches-du-Rhône | 2005-2007 | NA | NA | NA | NA | Larvae capture | NA | Moutailler et al. (2008) |
|  | Gard, Bouches-du-Rhône | 2005 | 8 | March, April, May, June, July, August, September, October | Spring,  Summer,  Autumn | NA | CDC trap | NA | Ponçon (2008) |
|  | Hérault, Gard, Bouches-du-Rhône | 1953-1966 | NA | January, February, March, April, May, June, July, August, September, October, November, December | Winter,  Spring,  Summer,  Autumn | NA | Larvae capture,  CDC trap | NA | Rageau et al. (1967) |
|  | Calvados, Manche, Ille-et-Vilaine, Côtes-d’Armor, Finistère, Morbihan, Loire-Atlantique, Vendée, Charente-Maritime, Gironde, Landes, Pyrénées-Atlantiques, Corse, Alpes-Maritimes, Var, Bouches-du-Rhône, Gard, Hérault, Aude, Pyrénées-Orientales | NA | NA | NA | NA | NA | NA | NA | Rageau et al. (1970b) |
|  | Hérault, Gard, Bouches-du-Rhône | 1965 | 1 | April | Spring | NA | Egg collection | NA | Rioux et al. (1967) |
|  | Morbihan, Loire-Atlantique, Var, Gard, Manche | 1902-1921 | NA | January, February, March, April, May, June, July, August, September, October, November, December | Winter,  Spring,  Summer,  Autumn | NA | NA | NA | Séguy (1923) |
|  | Bouches-du-Rhône, Gard | NA | NA | NA | NA | NA | Larvae and  adult capture | NA | Rioux et al. (1955) |
|  | Pyrénées-Orientales, Gironde, Charente-Maritime, Morbihan, Bouches-du-Rhône | 2011-2012 | NA | May, October, November | Spring,  Autumn | NA | NA | NA | GBIF (2023) |
| *Aedes*  *diantaeus* | Bas-Rhin | NA | NA | NA | NA | NA | NA | NA | Moussiegt (1986) |
|  | Bas-Rhin | NA | NA | NA | NA | NA | NA | NA | Rageau et al. (1970b) |
| *Aedes*  *dorsalis* | Morbihan, Loire-Atlantique | 1993 | NA | NA | NA | NA | Larvae capture | NA | Brutus et al. (1993) |
|  | Bouches-du-Rhône, Charente-Maritime, Corse, Cote d'Or, Côtes-d’Armor, Finistère, Gironde, Ille-et-Vilaine, Jura, Loire-Atlantique, Morbihan, Moselle, Oise, Bas-Rhin, Haut-Rhin, Seine-et-Marne, Vendée, Territoire-de-Belfort | NA | NA | NA | NA | NA | NA | NA | Moussiegt (1986) |
|  | Oise, Bas-Rhin, Haut-Rhin, Jura, Bouches-du-Rhône, Finistère, Côtes-d’Armor, Morbihan, Ille-et-Vilaine, Loire-Atlantique, Vendée, Charente-Maritime | NA | NA | NA | NA | NA | NA | NA | Rageau et al. (1970b) |
|  | Ille-et-Vilaine, Côtes-d'Armor, Loire-Atlantique | 1902-1921 | NA | January, February, March, April, May, June, July, August, September, October, November, December | Winter,  Spring,  Summer,  Autumn | NA | NA | NA | Séguy (1923) |
|  | Bouches-du-Rhône, Gard | NA | NA | NA | NA | NA | Larvae and  adult capture | NA | Rioux et al. (1955) |
|  | Vaucluse, Loir-et-Cher, Oise | 2008 | NA | June | Summer | NA | NA | NA | GBIF (2023) |
| *Aedes*  *echinus* | Seine-et-Marne | NA | NA | NA | NA | NA | NA | NA | Moussiegt (1986) |
|  | Hauts-de-Seine | 1902-1921 | NA | January, February, March, April, May, June, July, August, September, October, November, December | Winter,  Spring,  Summer,  Autumn | NA | NA | NA | Séguy (1923) |
| *Aedes*  *excrucians* | Ain, Isère, Rhône, Savoie | 1966-1976 | NA | NA | NA | NA | NA | NA | Gilot et al. (1976) |
|  | Ain, Alpes-de-Haute-Provence, Hautes-Alpes, Drôme, Haute-Garonne, Indre-et-Loire, Isère, Jura, Loire, Puy-de-Dôme, Pyrénées-Orientales, Bas-Rhin, Rhône, Savoie, Seine-et-Marne, Yvelines, Paris | NA | NA | NA | NA | NA | NA | NA | Moussiegt (1986) |
|  | Paris, Hauts-de-Seine, Seine-Saint-Denis, Val-de-Marne, Yvelines, Essonne, Val-d'Oise, Indre-et-Loire, Bas-Rhin, Isère, Savoie, Rhône, Ain, Haute-Garonne, Pyrénées-Orientales, Puy-De-Dôme | NA | NA | NA | NA | NA | NA | NA | Rageau et al. (1970b) |
|  | Pyrénées-Orientales | NA | NA | NA | NA | NA | NA | NA | Rioux et al. (1967) |
|  | Val-de-Marne | 1902-1921 | NA | January, February, March, April, May, June, July, August, September, October, November, December | Winter,  Spring,  Summer,  Autumn | NA | NA | NA | Séguy (1923) |
| *Aedes*  *flavescens* | Loire-Atlantique | 1993 | NA | NA | NA | NA | Larvae capture | NA | Brutus et al. (1993) |
|  | Bouches-du-Rhône, Charente-Maritime, Côte-d'Or, Gironde, Indre-et-Loire, Loire-Atlantique, Oise, Puy-de-Dôme, Seine-et-Marne, Vendée, Yvelines, Paris | NA | NA | NA | NA | NA | NA | NA | Moussiegt (1986) |
|  | Paris, Hauts-de-Seine, Seine-Saint-Denis, Val-de-Marne, Yvelines, Essonne, Val-d'Oise, Indre-et-Loire, Saône-et-Loire, Charente-Maritime, Bouches-du-Rhône | NA | NA | NA | NA | NA | NA | NA | Rageau et al. (1970b) |
|  | Hauts-de-Seine, Val-de-Marne | 1902-1921 | NA | January, February, March, April, May, June, July, August, September, October, November, December | Winter,  Spring,  Summer,  Autumn | NA | NA | NA | Séguy (1923) |
|  | Bouches-du-Rhône, Gard | NA | NA | NA | NA | NA | Larvae and  adult capture | NA | Rioux et al. (1955) |
|  | Gironde | NA | NA | NA | NA | NA | NA | NA | GBIF (2023) |
| *Aedes*  *geminus* | Ain, Isère, Rhône, Savoie | 1966-1976 | NA | NA | NA | NA | NA | NA | Gilot et al. (1976) |
|  | Marne, Haute-Marne | 2017-2020 | NA | January, February, March, April, May, June, July, August, September, October, November, December | Winter,  Spring,  Summer,  Autumn | NA | CDC trap,  BG-Sentinel,  Larvae capture,  Aspirator | NA | Martinet (2021) |
|  | Ain, Isère, Haute-Savoie | NA | NA | NA | NA | NA | NA | NA | Moussiegt (1986) |
| *Aedes*  *geniculatus* | Corse | 1944, 1946-1950, 1952 | NA | NA | NA | NA | NA | NA | Aitken (1954) |
|  | Bouches-du-Rhône, Hérault | 2004 | 6 | May, June, July, August, September, October | Spring,  Summer,  Autumn | 1, 25 | Hosted capture | NA | Balenghien et al. (2006) |
|  | Indre-et-Loire | 1936-1943 | NA | January, February, March, April, May, June, July, August, September, October, November, December | Winter,  Spring,  Summer,  Autumn | NA | NA | NA | Callot et al. (1944) |
|  | Ain, Isère, Rhône, Savoie, Drôme, Alpes-de-Haute-Provence, Bouches-du-Rhône, Corse, Alpes-Maritimes, Vaucluse | 1966-1976 | NA | NA | NA | NA | NA | NA | Gilot et al. (1976) |
|  | Hérault | 1955 | 1 | June | Summer | 27 | NA | NA | Harant et al. (1955) |
|  | Bouches-du-Rhône, Calvados, Dordogne, Doubs, Gard, Jura, Loire, Loiret, Oise, Pyrénées-Orientales, Savoie | 1955, 1991-1992, 2004, 2010-2011, 2013-2014, 2016-2018, 2020-2021 | NA | January, April, May, June, July, September | Winter,  Spring,  Summer | NA | NA | NA | INPN (2023) |
|  | Bouches-du-Rhône | 2006 | 11 | February, March, April, May, June, July, August, September, October, November, December | Winter,  Spring,  Summer,  Autumn | 0-50 | CDC trap  with CO2,  Ovitrap,  Human landing  capture | NA | l'Ambert et al. (2012) |
|  | Marne, Haute-Marne, Moselle | 2017-2020 | NA | January, February, March, April, May, June, July, August, September, October, November, December | Winter,  Spring,  Summer,  Autumn | NA | CDC trap,  BG-Sentinel,  Larvae capture,  Aspirator | NA | Martinet (2021) |
|  | Ain, Alpes-de-Haute-Provence, Hautes-Alpes, Alpes-Maritimes, Ardennes, Ariège, Aude, Aveyron, Bouches-du-Rhône, Calvados, Charente-Maritime, Corse, Creuse, Dordogne, Drôme, Gard, Haute-Garonne, Gers, Gironde, Hérault, Indre-et-Loire, Isère, Loire, Loire-Atlantique, Lot, Lot-et-Garonne, Maine-et-Loire, Manche, Marne, Meuse, Morbihan, Oise, Puy-de-Dôme, Pyrénées-Atlantiques, Hautes-Pyrénées, Pyrénées-Orientales, Bas-Rhin, Haut-Rhin, Rhône, Haute-Saône, Saône-et-Loire, Savoie, Seine-et-Marne, Tarn, Tarn-et-Garonne, Var, Vaucluse, Vendée, Vienne, Haute-Vienne, Yvelines, Territoire de Belfort, Paris | NA | NA | NA | NA | NA | NA | NA | Moussiegt (1986) |
|  | Gard, Bouches-du-Rhône | 2005 | 8 | March, April, May, June, July, August, September, October | Spring,  Summer,  Autumn | NA | CDC trap | NA | Ponçon (2008) |
|  | Hérault, Gard, Bouches-du-Rhône | 1953-1966 | NA | January, February, March, April, May, June, July, August, September, October, November, December | Winter,  Spring,  Summer,  Autumn | NA | Larvae capture,  CDC trap | NA | Rageau et al. (1967) |
|  | Paris, Hauts-de-Seine, Seine-Saint-Denis, Val-de-Marne, Yvelines, Essonne, Val-d'Oise, Oise, Calvados, Bas-Rhin, Haut-Rhin, Territoire-de-Belfort, Haute-Saône, Indre-et-Loire, Vienne, Creuse, Puy-de-Dôme, Saône-et-Loire, Rhône, Ain, Drôme, Isère, Savoie, Hautes-Alpes, Alpes-Maritimes, Var, Bouches-du-Rhône, Gard, Hérault, Aude, Pyrénées-Orientales, Alpes-Maritimes, Pyrénées-Atlantiques, Hautes-Pyrénées, Gard, Haute-Garonne, Gironde, Lot-et-Garonne, Tarn, Tarn-et-Garonne, Dordogne, Lot, Aveyron, Charente-Maritime, Vendée, Loire-Atlantique, Morbihan, Corse | NA | NA | NA | NA | NA | NA | NA | Rageau et al. (1970b) |
|  | Hérault, Gard, Bouches-du-Rhône | 1965 | 1 | April | Spring | NA | Egg collection | NA | Rioux et al. (1967) |
|  | Aveyron, Lot, Dordogne, Ariège, Haute-Garonne, Tarn, Tarn-et-Garonne, Lot-et-Garonne, Gers | NA | NA | March, April, May, June, July, August, September, October, November, December, January | Spring,  Summer,  Autumn,  Winter | NA | Larvae capture | NA | Ruffié (1957) |
|  | Orne | 1999 | 0.1 | October | Autumn | 145 | Larvae capture | NA | Schaffner et al. (2000) |
|  | Hauts-de-Seine, Yonne, Seine-et-Marne, Yvelines, Marne, Haute-Saône, Haute-Vienne, Pyrénées-Atlantiques, Pyrénées-Orientales, Corse | 1902-1921 | NA | January, February, March, April, May, June, July, August, September, October, November, December | Winter,  Spring,  Summer,  Autumn | NA | NA | NA | Séguy (1923) |
|  | All France | NA | NA | NA | NA | NA | NA | NA | Séguy (1924) |
|  | Bouches-du-Rhône, Gard | NA | NA | NA | NA | NA | Larvae and  adult capture | NA | Rioux et al. (1955) |
|  | Alpes-Maritimes, Bas-Rhin, Bouches-du-Rhône, Calvados, Dordogne, Doubs, Gard, Gironde, Hérault, Jura, Loire, Loiret, Oise, Paris, Pyrénées-Orientales, Savoie | 1955, 1991-1992, 2004, 2008, 2010-2011, 2013-2014, 2016-2018, 2020-2021 | NA | January, April, May, June, July, September | Winter,  Spring,  Summer | NA | NA | NA | GBIF (2023) |
| *Aedes*  *japonicus* | Meurthe-et-Moselle, Moselle, Bas-Rhin, Vosges, Haut-Rhin, Haute-Saône, Doubs, Territoire-de-Belfort, Haute-Marne | NA | NA | NA | NA | NA | NA | NA | ECDC (2022b) |
|  | Moselle, Haut-Rhin, Haute-Savoie | 2013, 2018, 2021 | NA | January, September, October | Winter,  Summer,  Autumn | NA | NA | NA | INPN (2023) |
|  | Bas-Rhin, Haut-Rhin, Vosges, Meurthe-et-Moselle, Moselle, Doubs, Haute-Saône | 2021 | NA | NA | NA | NA | NA | NA | Martinet (2021) |
|  | Moselle, Bas-Rhin | 2017-2020 | NA | January, February, March, April, May, June, July, August, September, October, November, December | Winter,  Spring,  Summer,  Autumn | NA | CDC trap,  BG-Sentinel,  Larvae capture,  Aspirator | NA | Martinet (2021) |
|  | Bas-Rhin | 2018-2019 | NA | NA | NA | NA | BG-Sentinel,  Human landing  capture | NA | Martinet (2021) |
|  | Moselle, Haute-Savoie, Bas-Rhin, Haut-Rhin | 2013, 2018, 2020, 2021 | NA | January, July, September, October | Winter,  Summer,  Autumn | NA | NA | NA | GBIF (2023) |
| *Aedes*  *mariae* | Bouches-du-Rhône, Var | 1926 | 1 | July | Summer | 24 | NA | NA | Brumpt (1942) |
|  | Corse | 1944, 1946-1950, 1952 | NA | NA | NA | NA | NA | NA | Aitken (1954) |
|  | Alpes-Maritimes, Aude, Bouches-du-Rhône, Corse, Gard, Hérault, Pyrénées-Orientales, Var | NA | NA | NA | NA | NA | NA | NA | Moussiegt (1986) |
|  | Corse, Pyrénées-Orientales, Aude, Hérault, Gard, Bouches-du-Rhône, Var, Alpes-Maritimes | NA | NA | NA | NA | NA | NA | NA | Rageau et al. (1970b) |
|  | Hérault, Gard, Bouches-du-Rhône | 1965 | 1 | April | Spring | NA | Egg collection | NA | Rioux et al. (1967) |
|  | Var, Gironde | 1902-1921 | NA | January, February, March, April, May, June, July, August, September, October, November, December | Winter,  Spring,  Summer,  Autumn | NA | NA | *Plasmodium danilewskyi* | Séguy (1923) |
| *Aedes*  *nigrinus* | Bas-Rhin, Seine-et-Marne, Paris | NA | NA | NA | NA | NA | NA | NA | Moussiegt (1986) |
|  | Paris, Hauts-de-Seine, Seine-Saint-Denis, Val-de-Marne, Seine-et-Marne, Bas-Rhin | NA | NA | NA | NA | NA | NA | NA | Rageau et al. (1970b) |
| *Aedes*  *nigripes* | Seine-et-Marne | 1902-1921 | NA | January, February, March, April, May, June, July, August, September, October, November, December | Winter,  Spring,  Summer,  Autumn | NA | NA | NA | Séguy (1923) |
| *Aedes*  *pulcritarsis* | Ain, Isère, Rhône, Savoie | 1966-1976 | NA | NA | NA | NA | NA | NA | Gilot et al. (1976) |
|  | Bouches-du-Rhône, Corse, Haute-Garonne, Hérault, Pyrénées-Orientales, Rhône, Savoie, Var, Yvelines, Paris | NA | NA | NA | NA | NA | NA | NA | Moussiegt (1986) |
|  | Haute-Garonne, Corse, Pyrénées-Orientales, Hérault, Var, Rhône | NA | NA | NA | NA | NA | NA | NA | Rageau et al. (1970b) |
|  | Hérault, Gard, Bouches-du-Rhône | 1965 | 1 | April | Spring | NA | Egg collection | NA | Rioux et al. (1967) |
|  | Aveyron, Lot, Dordogne, Ariège, Haute-Garonne, Tarn, Tarn-et-Garonne, Lot-et-Garonne, Gers | NA | NA | March, April, May, June, July, August, September, October, November, December, January | Spring,  Summer,  Autumn,  Winter | NA | Larvae capture | NA | Ruffié (1957) |
| *Aedes*  *pullatus* | Ain, Isère, Rhône, Savoie, Hautes-Alpes, Alpes-de-Haute-Provence, Drôme | 1966-1976 | NA | NA | NA | NA | NA | NA | Gilot et al. (1976) |
|  | Ain, Alpes-de-Haute-Provence, Hautes-Alpes, Alpes-Maritimes, Ariège, Corse, Drôme, Haute-Garonne, Isère, Pyrénées-Atlantiques, Hautes-Pyrénées, Pyrénées-Orientales, Savoie, Haute-Savoie | NA | NA | NA | NA | NA | NA | NA | Moussiegt (1986) |
|  | Pyrénées-Atlantiques, Hautes-Pyrénées, Ariège, Pyrénées-Orientales, Haute-Garonne, Ain, Isère, Savoie, Haute-Savoie, Alpes-de-Haute-Provence, Hautes-Alpes, Alpes-Maritimes | NA | NA | NA | NA | NA | NA | NA | Rageau et al. (1970b) |
|  | Pyrénées-Orientales | NA | NA | NA | NA | NA | NA | NA | Rioux et al. (1967) |
|  | Hautes-Alpes, Hautes-Pyrénées | 1902-1921 | NA | January, February, March, April, May, June, July, August, September, October, November, December | Winter,  Spring,  Summer,  Autumn | NA | NA | NA | Séguy (1923) |
|  | Pyrénées-Orientales | NA | NA | NA | NA | NA | NA | NA | GBIF (2023) |
| *Aedes*  *punctor* | Loire-Atlantique | 1993 | NA | NA | NA | NA | Larvae capture | NA | Brutus et al. (1993) |
|  | Indre-et-Loire | 1936-1943 | NA | January, February, March, April, May, June, July, August, September, October, November, December | Winter,  Spring,  Summer,  Autumn | NA | NA | NA | Callot et al. (1944) |
|  | Ain, Isère, Rhône, Savoie, Ardèche | 1966-1976 | NA | NA | NA | NA | NA | NA | Gilot et al. (1976) |
|  | Ain, Ardèche, Ardennes, Bouches-du-Rhône, Calvados Côte-d'Or, Haute-Garonne, Gironde, Indre-et-Loire, Isère, Landes, Loire, Haute-Loire, Loire-Atlantique, Oise, Puy-de-Dôme, Pyrénées-Orientales, Bas-Rhin, Haut-Rhin, Rhône, Haute-Savoie, Savoie, Seine-Maritime, Seine-et-Marne, Vendée, Vosges, Yvelines, Territoire de Belfort, Paris | NA | NA | NA | NA | NA | NA | NA | Moussiegt (1986) |
|  | Paris, Hauts-de-Seine, Seine-Saint-Denis, Val-de-Marne, Yvelines, Essonne, Val-d'Oise, Seine-et-Marne, Oise, Seine-Maritime, Calvados, Ardennes, Bas-Rhin, Haut-Rhin, Vosges, Haute-Saône, Landes, Pyrénées-Orientales, Bouches-du-Rhône, Puy-de-Dôme, Haute-Loire, Rhône, Ain, Isère | NA | NA | NA | NA | NA | NA | NA | Rageau et al. (1970b) |
|  | Pyrénées-Orientales | NA | NA | NA | NA | NA | NA | NA | Rioux et al. (1967) |
|  | All France | 1902-1921 | NA | January, February, March, April, May, June, July, August, September, October, November, December | Winter,  Spring,  Summer,  Autumn | NA | NA | NA | Séguy (1923) |
|  | Puy-de-Dôme | NA | NA | NA | NA | NA | NA | NA | GBIF (2023) |
| *Aedes*  *refiki* | Ain, Isère, Rhône, Savoie, Bouches-du-Rhône | 1966-1976 | NA | NA | NA | NA | NA | NA | Gilot et al. (1976) |
|  | Marne | 2017-2020 | NA | January, February, March, April, May, June, July, August, September, October, November, December | Winter,  Spring,  Summer,  Autumn | NA | CDC trap,  BG-Sentinel,  Larvae capture,  Aspirator | NA | Martinet (2021) |
|  | Ain, Alpes-de-Haute-Provence, Hautes-Alpes, Alpes-Maritimes, Bouches-du-Rhône, Drôme, Isère, Puy-de-Dôme, Pyrénées-Orientales, Bas-Rhin, Rhône, Savoie, Var, Vaucluse | NA | NA | NA | NA | NA | NA | NA | Moussiegt (1986) |
|  | Bas-Rhin, Puy-de-Dôme, Ain, Isère, Savoie, Var, Pyrénées-Orientales | NA | NA | NA | NA | NA | NA | NA | Rageau et al. (1970b) |
| *Aedes*  *rusticus* | Morbihan, Loire-Atlantique | 1993 | NA | NA | NA | NA | Larvae capture | NA | Brutus et al. (1993) |
|  | Bouches-du-Rhône | 1926 | 1 | July | Summer | 24 | NA | NA | Brumpt (1942) |
|  | Corse | 1944, 1946-1950, 1952 | NA | NA | NA | NA | NA | NA | Aitken (1954) |
|  | Indre-et-Loire | 1936-1943 | NA | January, February, March, April, May, June, July, August, September, October, November, December | Winter,  Spring,  Summer,  Autumn | NA | NA | NA | Callot et al. (1944) |
|  | Ain, Isère, Rhône, Savoie, Var, Hautes-Alpes, Bouches-du-Rhône | 1966-1976 | NA | NA | NA | NA | NA | NA | Gilot et al. (1976) |
|  | Savoie | 2015-2017 | NA | January | Winter | NA | NA | NA | INPN (2023) |
|  | Bouches-du-Rhône | 2006 | 11 | February, March, April, May, June, July, August, September, October, November, December | Winter,  Spring,  Summer,  Autumn | 0-50 | CDC trap  with CO2,  Ovitrap,  Human landing  capture | NA | l'Ambert et al. (2012) |
|  | Marne, Ardennes, Haute-Marne, Moselle | 2017-2020 | NA | January, February, March, April, May, June, July, August, September, October, November, December | Winter,  Spring,  Summer,  Autumn | NA | CDC trap,  BG-Sentinel,  Larvae capture,  Aspirator | NA | Martinet (2021) |
|  | Marne | 2018-2019 | NA | NA | NA | NA | BG-Sentinel,  Human landing  capture | NA | Martinet (2021) |
|  | Marne | 2018, 2020 | 4 | March, May, September, October | Spring,  Autumn | NA | Larvae and  adult capture | NA | Martinet (2021) |
|  | Marne | 2019 | 2 | April | Spring | NA | Larvae capture | NA | Martinet (2021) |
|  | Ain, Allier, Alpes-de-Haute-Provence, Alpes-Maritimes, Ardennes, Aveyron, Bouches-du-Rhône, Charente-Maritime, Corse, Côte-d'Or, Drôme, Gard, Haute-Garonne, Gironde, Hérault, Ille-et-Vilaine, Indre-et-Loire, Isère, Jura, Loire-Atlantique, Mayenne, Morbihan, Oise, Puy-de-Dôme, Hautes-Pyrénées, Bas-Rhin, Haut-Rhin, Rhône, Savoie, Haute-Savoie, Seine-et-Marne, Deux-Sèvres, Tarn, Var, Vaucluse, Vendée, Vienne, Yvelines, Territoire de Belfort, Paris | NA | NA | NA | NA | NA | NA | NA | Moussiegt (1986) |
|  | Bouches-du-Rhône | 2005 | 8 | March, April, May, June, July, August, September, October | Spring,  Summer,  Autumn | NA | CDC trap | NA | Ponçon (2008) |
|  | Paris, Hauts-de-Seine, Seine-Saint-Denis, Val-de-Marne, Yvelines, Essonne, Val-d'Oise, Seine-et-Marne, Oise, Ardennes, Bas-Rhin, Haut-Rhin, Ille-et-Vilaine, Mayenne, Indre-et-Loire, Vienne, Allier, Jura, Ain, Rhône, Isère, Haute-Garonne, Tarn, Aveyron, Hérault, Gard, Bouches-du-Rhône, Corse | NA | NA | NA | NA | NA | NA | NA | Rageau et al. (1970b) |
|  | Aveyron, Lot, Dordogne, Ariège, Haute-Garonne, Tarn, Tarn-et-Garonne, Lot-et-Garonne, Gers | NA | NA | March, April, May, June, July, August, September, October, November, December, January | Spring,  Summer,  Autumn,  Winter | NA | Larvae capture | NA | Ruffié (1957) |
|  | Paris, Mayenne, Ille-et-Vilaine, Jura, Allier | 1902-1921 | NA | January, February, March, April, May, June, July, August, September, October, November, December | Winter,  Spring,  Summer,  Autumn | NA | NA | NA | Séguy (1923) |
|  | All France | NA | NA | NA | NA | NA | NA | NA | Séguy (1924) |
|  | Landes, Gironde, Corrèze, Savoie, Loir-et-Cher, Orne, Seine-Saint-Denis, Marne, Oise | 2000, 2015-2018 | NA | January, March | Winter,  Spring | NA | NA | NA | GBIF (2023) |
| *Aedes*  *sticticus* | Loire-Atlantique | 1993 | NA | NA | NA | NA | Larvae capture | NA | Brutus et al. (1993) |
|  | Indre-et-Loire | 1936-1943 | NA | January, February, March, April, May, June, July, August, September, October, November, December | Winter,  Spring,  Summer,  Autumn | NA | NA | NA | Callot et al. (1944) |
|  | Savoie | 1983 | 0.03 | July | Summer | 280-325,  286-656 | Human landing  capture | NA | Chastel et al. (1985) |
|  | Ain, Isère, Rhône, Savoie, Vaucluse | 1966-1976 | NA | NA | NA | NA | NA | NA | Gilot et al. (1976) |
|  | Savoie, Pas-de-Calais | 2015-2017, 2021 | NA | January, July | Winter,  Summer | NA | NA | NA | INPN (2023) |
|  | Marne | 2017-2020 | NA | January, February, March, April, May, June, July, August, September, October, November, December | Winter,  Spring,  Summer,  Autumn | NA | CDC trap,  BG-Sentinel,  Larvae capture,  Aspirator | NA | Martinet (2021) |
|  | Marne | 2018-2019 | NA | NA | NA | NA | BG-Sentinel,  Human landing  capture | NA | Martinet (2021) |
|  | Ain, Ariège, Aveyron, Côte-d'Or, Creuse, Doubs, Drôme, Haute-Garonne, Gers, Indre-et-Loire, Isère, Jura, Loire-Atlantique, Lot, Lot-Et-Garonne, Moselle, Nord, Puy-de-Dôme, Hautes-Pyrénées, Bas-Rhin, Haut-Rhin, Rhône, Savoie, Seine-et-Marne, Tarn, Tarn-et-Garonne, Vaucluse, Yvelines, Paris | NA | NA | NA | NA | NA | NA | NA | Moussiegt (1986) |
|  | Paris, Hauts-de-Seine, Seine-Saint-Denis, Val-de-Marne, Yvelines, Essonne, Val-d'Oise, Isère, Rhône, Ain, Drôme, Creuse, Dordogne, Lot, Aveyron, Lot-et-Garonne, Tarn-et-Garonne, Tarn, Gers, Hautes-Pyrénées, Haute-Garonne, Ariège | NA | NA | NA | NA | NA | NA | NA | Rageau et al. (1970b) |
|  | Aveyron, Lot, Dordogne, Ariège, Haute-Garonne, Tarn, Tarn-et-Garonne, Lot-et-Garonne, Gers | NA | NA | March, April, May, June, July, August, September, October, November, December, January | Spring,  Summer,  Autumn,  Winter | NA | Larvae capture | NA | Ruffié (1957) |
|  | Seine-et-Marne, Haut-Rhin, Rhône, Creuse | 1902-1921 | NA | January, February, March, April, May, June, July, August, September, October, November, December | Winter,  Spring,  Summer,  Autumn | NA | NA | NA | Séguy (1923) |
|  | Pas-de-Calais, Marne, Savoie | 2015-2018, 2021 | NA | June, July | Summer | NA | NA | NA | GBIF (2023) |
| *Aedes*  *stictitus /*  *vexans* | Savoie | 1983 | 0.03 | July | Summer | 280-325,  286-656 | Human landing  capture | NA | Chastel et al. (1985) |
|  | Savoie | 1983-1985 | 12 | May, June, July | Summer | 310, 1538 | Human landing  capture,  Net capture | NA | Chastel et al. (1987) |
| *Aedes*  *surcoufi* | Isère, Pyrénées-Orientales, Seine-et-Marne, Paris | NA | NA | NA | NA | NA | NA | NA | Moussiegt (1986) |
|  | Seine-et-Marne, Hauts-de-Seine | 1902-1921 | NA | January, February, March, April, May, June, July, August, September, October, November, December | Winter,  Spring,  Summer,  Autumn | NA | NA | NA | Séguy (1923) |
| *Aedes*  *vexans* | Loire-Atlantique | 1993 | NA | NA | NA | NA | Larvae capture | NA | Brutus et al. (1993) |
|  | Bas-Rhin | 1981-1982 | NA | NA | NA | NA | NA | NA | Arnold et al. (1982) |
|  | Bouches-du-Rhône | 1926 | 1 | July | Summer | 24 | NA | NA | Brumpt (1942) |
|  | Corse | 1944, 1946-1950, 1952 | NA | NA | NA | NA | NA | NA | Aitken (1954) |
|  | Bouches-du-Rhône | 2004 | 6 | May, June, July, August, September, October | Spring,  Summer,  Autumn | 1, 25 | Hosted capture | NA | Balenghien et al. (2006) |
|  | Indre-et-Loire | 1936-1943 | NA | January, February, March, April, May, June, July, August, September, October, November, December | Winter,  Spring,  Summer,  Autumn | NA | NA | NA | Callot et al. (1944) |
|  | Savoie | 1983 | 0.03 | July | Summer | 280-325,  286-656 | Human landing  capture | NA | Chastel et al. (1985) |
|  | Hauts-de-Seine, Seine-Saint-Denis, Val-de-Marne, Val-d'Oise, Yvelines, Essonne, Seine-et-Marne, Oise, Paris, Ardennes, Meurthe-et-Moselle, Bas-Rhin, Haut-Rhin, Haute-Saône, Côte-d'Or, Territoire-de-Belfort, Jura, Creuse, Allier, Puy-de-Dôme, Loire, Rhône, Isère, Savoie, Haute-Savoie, Ain, Drôme, Vaucluse, Alpes-de-Haute-Provence, Alpes-Maritimes, Var, Bouches-du-Rhône, Gard, Hérault, Aude, Pyrénées-Atlantiques, Ariège, Haute-Garonne, Tarn, Aveyron, Lot, Tarn-et-Garonne, Gers, Hautes-Pyrénées, Pyrénées-Atlantiques, Lot-et-Garonne, Dordogne, Gironde, Charente-Maritime, Vendée, Deux-Sèvres, Vienne, Indre-et-Loire, Loire-Atlantique, Morbihan, Ille-et-Vilaine, Mayenne | NA | NA | NA | NA | NA | NA | NA | ECDC (2022b) |
|  | Ain, Isère, Rhône, Savoie, Drôme | 1966-1976 | NA | NA | NA | NA | NA | NA | Gilot et al. (1976) |
|  | Haute-Loire, Nord, Loiret, Bouches-du-Rhône, Calvados, Savoie, Alpes-Maritimes | 2010, 2012, 2014-2017, 2020 | NA | January, May, June, August, October | Winter,  Spring,  Summer,  Autumn | NA | NA | NA | INPN (2023) |
|  | Bouches-du-Rhône | 2006 | 11 | February, March, April, May, June, July, August, September, October, November, December | Winter,  Spring,  Summer,  Autumn | 0-50 | CDC trap  with CO2,  Ovitrap,  Human landing  capture | NA | l'Ambert et al. (2012) |
|  | Ain, Alpes-Maritimes, Ariège, Aude, Aveyron, Bouches du-Rhône, Charente-Maritime, Corse, Côte-d'Or, Creuse, Dordogne, Drôme, Gard, Haute-Garonne, Gers, Gironde, Hérault, Indre-et-Loire, Isère, Jura, Loire, Lot, Lot-et-Garonne, Oise, Puy-de-Dôme, Pyrénées-Atlantiques, Hautes-Pyrénées, Pyrénées-Orientales, Bas-Rhin, Haut-Rhin, Rhône, Haute-Saône, Savoie, Seine-et-Marne, Tarn, Tarn-Et-Garonne, Var, Vienne, Yvelines, Territoire de Belfort, Paris | NA | NA | NA | NA | NA | NA | NA | Moussiegt (1986) |
|  | Gard, Bouches-du-Rhône | 2005 | 8 | March, April, May, June, July, August, September, October | Spring,  Summer,  Autumn | NA | CDC trap | NA | Ponçon (2008) |
|  | Hérault, Gard, Bouches-du-Rhône | 1953-1966 | NA | January, February, March, April, May, June, July, August, September, October, November, December | Winter,  Spring,  Summer,  Autumn | NA | Larvae capture,  CDC trap | NA | Rageau et al. (1967) |
|  | Paris, Hauts-de-Seine, Seine-Saint-Denis, Val-de-Marne, Yvelines, Essonne, Val-d'Oise, Oise, Bas-Rhin, Haut-Rhin, Haute-Saône, Indre-et-Loire, Vienne, Creuse, Loire, Rhône, Ain, Isère, Drôme, Corse, Dordogne, Lot-et-Garonne, Gers, Lot, Tarn-et-Garonne, Haute-Garonne, Pyrénées-Atlantiques, Hautes-Pyrénées, Ariège, Pyrénées-Orientales, Aude, Lot, Aveyron, Tarn, Tarn-et-Garonne, Hérault, Gard, Bouches-du-Rhône, Var, Alpes-Maritimes | NA | NA | NA | NA | NA | NA | NA | Rageau et al. (1970b) |
|  | Aveyron, Lot, Dordogne, Ariège, Haute-Garonne, Tarn, Tarn-et-Garonne, Lot-et-Garonne, Gers | NA | NA | March, April, May, June, July, August, September, October, November, December, January | Spring,  Summer,  Autumn,  Winter | NA | Larvae capture | NA | Ruffié (1957) |
|  | Alpes-de-Haute-Provence, Haute-Saône, Hautes-Pyrénées, Creuse, Rhône, Bas-Rhin, Haut-Rhin | 1902-1921 | NA | January, February, March, April, May, June, July, August, September, October, November, December | Winter,  Spring,  Summer,  Autumn | NA | NA | NA | Séguy (1923) |
|  | All France | NA | NA | NA | NA | NA | NA | NA | Séguy (1924) |
|  | Bouches-du-Rhône, Gard | NA | NA | NA | NA | NA | Larvae and  adult capture | NA | Rioux et al. (1955) |
|  | Alpes-Maritimes, Bouches-du-Rhône, Calvados, Gironde, Haute-Loire, Hérault, Loiret, Nord, Oise, Puy-de-Dôme, Savoie, Var, Vaucluse | 2008, 2010, 2012, 2014-2017, 2020 | NA | January, May, June, August, October | Winter,  Spring,  Summer,  Autumn | NA | NA | NA | GBIF (2023) |
| *Aedes*  *vittatus* | Corse | 1944, 1946-1950, 1952 | NA | NA | NA | NA | NA | NA | Aitken (1954) |
|  | Hautes-Alpes, Alpes-Maritimes, Aude, Bouches-Du-Rhône, Corse, Gard, Hérault, Hautes-Pyrénées, Pyrénées-Orientales, Var, Vaucluse | NA | NA | NA | NA | NA | NA | NA | Moussiegt (1986) |
|  | Corse, Pyrénées-Orientales, Aude, Hérault, Gard, Bouches-du-Rhône, Var, Alpes-Maritimes | NA | NA | NA | NA | NA | NA | NA | Rageau et al. (1970b) |
|  | Hérault, Gard, Bouches-du-Rhône | 1965 | 1 | April | Spring | NA | Egg collection | NA | Rioux et al. (1967) |
|  | Corse | 1902-1921 | NA | January, February, March, April, May, June, July, August, September, October, November, December | Winter,  Spring,  Summer,  Autumn | NA | NA | NA | Séguy (1923) |
|  | Pyrénées-Orientales | NA | NA | NA | NA | NA | NA | NA | GBIF (2023) |
| *Aedes*  *zammiti* | Pyrénées-Orientales | 1902-1921 | NA | January, February, March, April, May, June, July, August, September, October, November, December | Winter,  Spring,  Summer,  Autumn | NA | NA | NA | Séguy (1923) |
| *Anopheles*  *algeriensis* | Gard | 2005 | 8 | March, April, May, June, July, August, September, October | Spring,  Summer,  Autumn | 5 | CDC trap,  Human landing  capture  Hosted capture,  Aspirator | NA | Ponçon et al. (2007b) |
|  | Corse | 1944, 1946-1950, 1952 | NA | NA | NA | NA | NA | NA | Aitken (1954) |
|  | Corse | 1924 | 2 | June, July | Summer | NA | NA | NA | Brumpt (1925) |
|  | Bouches-du-Rhône | 2006 | 11 | February, March, April, May, June, July, August, September, October, November, December | Winter,  Spring,  Summer,  Autumn | 0-50 | CDC trap  with CO2,  Ovitrap,  Human landing  capture | NA | l'Ambert et al. (2012) |
|  | Aude, Bouches-du-Rhône, Charente-Maritime, Corse, Gard, Gironde, Hérault, Loire-Atlantique, Morbihan, Pyrénées-Orientales | NA | NA | NA | NA | NA | NA | NA | Moussiegt (1986) |
|  | Gard, Bouches-du-Rhône | 2005 | 10 | March, April, May, June, July, August, September, October, November, December | NA | NA | Larvae and  adult capture | NA | Ponçon (2008) |
|  | Bouches-du-Rhône | 2005 | 8 | March, April, May, June, July, August, September, October | Spring,  Summer,  Autumn | NA | CDC trap | NA | Ponçon (2008) |
|  | Finistère, Morbihan, Ille-et-Vilaine, Loire-Atlantique, Maine-et-Loire, Vendée, Deux-Sèvres, Vienne, Charente-Maritime, Charente, Gironde, Dordogne, Landes, Lot-et-Garonne, Lot, Pyrénées-Atlantiques, Tarn-et-Garonne, Gers, Hautes-Pyrénées, Haute-Garonne, Ariège, Tarn, Aude, Pyrénées-Orientales, Hérault, Aveyron, Lozère, Gard, Ardèche, Drôme, Vaucluse, Bouches-du-Rhône, Var, Vaucluse, Hautes-Alpes, Alpes-de-Haute-Provence, Alpes-Maritimes, Corse | 1963-2007 | NA | NA | NA | NA | NA | NA | Ponçon (2008) |
|  | Hérault, Gard, Bouches-du-Rhône | 1953-1966 | NA | January, February, March, April, May, June, July, August, September, October, November, December | Winter,  Spring,  Summer,  Autumn | NA | Larvae capture,  CDC trap | NA | Rageau et al. (1967) |
|  | Morbihan, Loire-Atlantique, Charente-Maritime, Corse, Hérault, Aude, Pyrénées-Orientales, Bouches-du-Rhône | NA | NA | NA | NA | NA | NA | NA | Rageau et al. (1970b) |
|  | Aude, Bouche-de-Rhône, Charente-Maritime, Gard, Gironde, Hérault, Loire-Atlantique, Morbihan, Pyrénées-Orientales, Corse | NA | NA | NA | NA | NA | NA | NA | Ramsdale et al. (2000) |
|  | Hérault, Gard, Bouches-du-Rhône | 1965 | 1 | April | Spring | NA | Egg collection | NA | Rioux et al. (1967) |
|  | Bouches-du-Rhône, Gard | NA | NA | NA | NA | NA | Larvae and  adult capture | NA | Rioux et al. (1955) |
|  | Aube | NA | NA | NA | NA | NA | NA | NA | GBIF (2023) |
| *Anopheles*  *beklemishevi* | All France | NA | NA | NA | NA | NA | NA | NA | Carnevale et al. (2009) |
| *Anopheles*  *claviger* | Corse | 1944, 1946-1950, 1952 | NA | NA | NA | NA | NA | NA | Aitken (1954) |
|  | Indre-et-Loire | 1936-1943 | NA | January, February, March, April, May, June, July, August, September, October, November, December | Winter,  Spring,  Summer,  Autumn | NA | NA | NA | Callot et al. (1944) |
|  | Ain, Isère, Rhône, Savoie | 1966-1976 | NA | NA | NA | NA | NA | NA | Gilot et al. (1976) |
|  | Moselle, Pas-de-Calais, Pyrénées-Orientales, Savoie | 1955, 1958, 1991, 1995, 2014-2017 | NA | January, April, August, September | Winter,  Spring,  Summer | NA | NA | NA | INPN (2023) |
|  | Marne | 2017-2020 | NA | January, February, March, April, May, June, July, August, September, October, November, December | Winter,  Spring,  Summer,  Autumn | NA | CDC trap,  BG-Sentinel,  Larvae capture,  Aspirator | NA | Martinet (2021) |
|  | Ain, Aisne, Allier, Hautes-Alpes, Alpes-Maritimes Ardennes, Ariège, Aube, Aude, Aveyron, Bouches-du-Rhône, Calvados, Charente-Maritime, Corse, Côte-d'Or, Dordogne, Drôme, Eure-et-Loir, Gard, Haute-Garonne, Gironde, Hérault, Ille-et-Vilaine, Indre-et-Loire, Isère, Landes, Loire, Haute-Loire, Loire-Atlantique, Loiret, Lot, Maine-et-Loire, Manche, Marne, Meurthe-et-Moselle, Meuse, Morbihan, Moselle, Nord, Oise, Pas-de-Calais, Puy-de-Dôme, Pyrénées-Atlantiques, Hautes-Pyrénées, Pyrénées-Orientales, Bas-Rhin, Haut-Rhin, Rhône, Haute-Saône, Savoie, Seine-Maritime, Seine-et-Marne, Deux-Sèvres, Somme, Tarn, Var, Vaucluse, Vendée, Vienne, Yonne, Yvelines, Territoire de Belfort, Paris | NA | NA | NA | NA | NA | NA | NA | Moussiegt (1986) |
|  | All France | 1963-2007 | NA | NA | NA | NA | NA | NA | Ponçon (2008) |
|  | Hérault, Gard, Bouches-du-Rhône | 1953-1966 | NA | January, February, March, April, May, June, July, August, September, October, November, December | Winter,  Spring,  Summer,  Autumn | NA | Larvae capture,  CDC trap | NA | Rageau et al. (1967) |
|  | Ain, Aisne, Allier, Alpes-Maritimes, Ardennes, Ariège, Aube, Aveyron, Bouches-du-Rhône, Calvados, Charente-Maritime, Corse, Côte-d'Or, Côtes-d'Armor, Dordogne, Finistère, Gard, Haute-Garonne, Gironde, Hérault, Ille-et-Vilaine, Indre-et-Loire, Isère, Loire, Haute-Loire, Loire-Atlantique, Loiret, Lot, Maine-et-Loire, Manche, Meurthe-et-Moselle, Meuse, Morbihan, Moselle, Nord, Oise, Puy-de-Dôme, Pyrénées-Atlantiques, Hautes-Pyrénées, Pyrénées-Orientales, Bas-Rhin, Haut-Rhin, Rhône, Savoie, Haute-Savoie, Paris, Seine-et-Marne, Yvelines, Deux-Sèvres, Var, Vaucluse, Vendée, Vienne, Yonne, Territoire-de-Belfort, Essonne, Hauts-de-Seine, Seine-Saint-Denis, Val-de-Marne, Val-d'Oise | NA | NA | NA | NA | NA | NA | NA | Rageau et al. (1970b) |
|  | Ain, Aisne, Allier, Hautes-Alpes, Alpes-Maritimes, Ardennes, Ariège, Aube, Aude, Aveyron, Bouches-du-Rhône, Calvados, Charente-Maritime, Cote d'Or, Dordogne, Drôme, Eure-et-Loir, Gard, Haute-Garonne, Gironde, Hérault, Ille-et-Vilaine, Indre-et-Loire, Isère, Landes, Loire, Haute-Loire, Loire-Atlantique, Loiret, Lot, Maine-et-Loire, Manche, Marne, Meurthe-et-Moselle, Meuse, Morbihan, Moselle, Nord, Oise, Pas-de-Calais, Puy-de-Dôme, Pyrénées-Atlantiques, Hautes-Pyrénées, Pyrénées-Orientales, Bas-Rhin, Haut-Rhin, Rhône, Haute-Saône, Savoie, Seine-Maritime, Seine-et-Marne, Deux-Sèvres, Somme, Tarn, Var, Vaucluse, Vendée, Vienne, Yonne, Yvelines, Territoire de Belfort, Paris, Corse | NA | NA | NA | NA | NA | NA | NA | Ramsdale et al. (2000) |
|  | Aveyron, Lot, Dordogne, Ariège, Haute-Garonne, Tarn, Tarn-et-Garonne, Lot-et-Garonne, Gers | NA | NA | March, April, May, June, July, August, September, October, November, December, January | Spring,  Summer,  Autumn,  Winter | NA | Larvae capture | NA | Ruffié (1957) |
|  | Orne | 1999 | 0.1 | October | Autumn | 145 | Larvae capture | NA | Schaffner et al. (2000) |
|  | Ardennes, Yvelines, Seine-et-Marne, Hauts-de-Seine, Maine-et-Loire, Côte-d'Or, Meuse, Côtes-d’Armor | 1902-1921 | NA | January, February, March, April, May, June, July, August, September, October, November, December | Winter,  Spring,  Summer,  Autumn | NA | NA | *Plasmodium vivax, Plasmodium falciparum, Dorofilaria immitis* | Séguy (1923) |
|  | All France | NA | NA | NA | NA | NA | NA | NA | Séguy (1924) |
|  | Corse | NA | NA | NA | NA | NA | NA | NA | Takken et al. (2007) |
|  | Bouches-du-Rhône, Gard | NA | NA | NA | NA | NA | Larvae and  adult capture | NA | Rioux et al. (1955) |
|  | Corse, Haute-Loire, Loire-Atlantique, Moselle, Pas-de-Calais, Puy-de-Dôme, Pyrénées-Orientales, Savoie | 1944, 1955, 1958, 1991, 1995, 2014-2017 | NA | January, April, August, September | Spring,  Summer,  Winter | NA | NA | NA | GBIF (2023) |
| *Anopheles*  *hyrcanus* | Gard | 2005 | 8 | March, April, May, June, July, August, September, October | Spring,  Summer,  Autumn | 5 | CDC trap,  Human landing  capture  Hosted capture,  Aspirator | NA | Ponçon et al. (2007b) |
|  | Bouches-du-Rhône | 1926 | 1 | July | Summer | 24 | NA | NA | Brumpt (1942) |
|  | Corse | 1944, 1946-1950, 1952 | NA | NA | NA | NA | NA | NA | Aitken (1954) |
|  | Bouches-du-Rhône | 2004 | 6 | May, June, July, August, September, October | Spring,  Summer,  Autumn | 1, 25 | Hosted capture | NA | Balenghien et al. (2006) |
|  | Corse | 1924 | 2 | June, July | Summer | NA | NA | NA | Brumpt (1925) |
|  | Bouches-du-Rhône | 1964 | 3 |  |  |  | NA | NA | Hannoun et al. (1964) |
|  | Bouches-du-Rhône | 2006 | 11 | February, March, April, May, June, July, August, September, October, November, December | Winter,  Spring,  Summer,  Autumn | 0-50 | CDC trap  with CO2,  Ovitrap,  Human landing  capture | NA | l'Ambert et al. (2012) |
|  | Bouches-du-Rhône | 1965-1967 | 15 | December, January, February, March, April | Winter | NA | NA | NA | Mouchet et al. (1969) |
|  | Bouches-du-Rhône | 1964-1968 | 10 | February, March, April, May, June, July, August, September, October, December | Winter,  Spring,  Summer,  Autumn | NA | NA | NA | Mouchet et al. (1970) |
|  | Bouches-du-Rhône, Corse, Gard, Haute-Garonne, Hérault | NA | NA | NA | NA | NA | NA | NA | Moussiegt (1986) |
|  | Gard, Bouches-du-Rhône | 2005 | 10 | March, April, May, June, July, August, September, October, November, December | NA | NA | Larvae and  adult capture | NA | Ponçon (2008) |
|  | Bouches-du-Rhône, Gard | 2005-2006 | 15 | March, April, May, June, July, August, September, October | Spring,  Summer,  Autumn | NA | Larvae capture,  CDC trap | NA | Ponçon (2008) |
|  | Gard, Bouches-du-Rhône | 2005 | 8 | March, April, May, June, July, August, September, October | Spring,  Summer,  Autumn | NA | CDC trap | NA | Ponçon (2008) |
|  | Gard, Bouches-du-Rhône | 2004-2005 | 40 | March, April, May, June, July, August, September, October | Spring,  Summer,  Autumn | NA | CDC trap  with and  without CO2, | NA | Ponçon (2008) |
|  | Haute-Garonne, Ariège, Pyrénées-Orientales, Aude, Tarn, Hautes-Pyrénées, Tarn-et-Garonne, Aveyron, Lozère, Hérault, Gard, Vaucluse, Bouches-du-Rhône, Vaucluse, Var | 1963-2007 | NA | NA | NA | NA | NA | NA | Ponçon (2008) |
|  | Bouches-du-Rhône | 1969-2006 | NA | June, July, August, September, October | Summer,  Autumn | NA | Human landing  capture | NA | Ponçon et al. (2007a) |
|  | Hérault, Gard, Bouches-du-Rhône | 1953-1966 | NA | January, February, March, April, May, June, July, August, September, October, November, December | Winter,  Spring,  Summer,  Autumn | NA | Larvae capture,  CDC trap | NA | Rageau et al. (1967) |
|  | Hérault, Gard, Bouches-du-Rhône, Corse | NA | NA | NA | NA | NA | NA | NA | Rageau et al. (1970b) |
|  | Ain, Bouches-du-Rhône, Gard, Haut Garonne, Hérault, Corse | NA | NA | NA | NA | NA | NA | NA | Ramsdale et al. (2000) |
|  | Hérault, Gard, Bouches-du-Rhône | 1965 | 1 | April | Spring | NA | Egg collection | NA | Rioux et al. (1967) |
|  | Bouches-du-Rhône | 1936 | NA | NA | Summer | 0-7 | Larvae and  adult capture | NA | Treillard (1937) |
|  | Gard, Hérault, Bouches-du-Rhône | 2001 | 7 | April, May, June, July, August, September, October | Spring,  Summer,  Autumn | NA | CDC trap  with CO2 | NA | Zientara et al. (2004) |
|  | Bouches-du-Rhône, Gard | NA | NA | NA | NA | NA | Larvae and  adult capture | NA | Rioux et al. (1955) |
|  | Bouches-du-Rhône, Oise | 2005 | NA | January | Winter | NA | NA | NA | GBIF (2023) |
| *Anopheles*  *maculipennis*  s.l. | Gard | 2005 | 8 | March, April, May, June, July, August, September, October | Spring,  Summer,  Autumn | 5 | CDC trap,  Human landing  capture  Hosted capture,  Aspirator | NA | Ponçon et al. (2007b) |
|  | Corse, Gard, Bouches-du-Rhône | NA | NA | NA | NA | NA | Larvae capture | NA | Brumpt (1944b) |
|  | Manche, Calvados | 1945 | NA | NA | NA | NA | NA | NA | Brumpt (1944a) |
|  | Bouches-du-Rhône, Corse | 1926 | 1 | July | Summer | 24 | NA | NA | Brumpt (1942) |
|  | Corse | 1944, 1946-1950, 1952 | NA | NA | NA | NA | NA | NA | Aitken (1954) |
|  | Bouches-du-Rhône, Hérault | 2004 | 6 | May, June, July, August, September, October | Spring,  Summer,  Autumn | 1, 25 | Hosted capture | NA | Balenghien et al. (2006) |
|  | Corse | 1924 | 2 | June, July | Summer | NA | NA | NA | Brumpt (1925) |
|  | Indre-et-Loire | 1936-1943 | NA | January, February, March, April, May, June, July, August, September, October, November, December | Winter,  Spring,  Summer,  Autumn | NA | NA | NA | Callot et al. (1944) |
|  | All France (Except Corsica) | NA | NA | NA | NA | NA | NA | NA | Carnevale et al. (2009) |
|  | Ain, Aisne, Allier, Alpes-de-Haute-Provence, Hautes-Alpes, Alpes-Maritimes, Ardèche, Ardennes, Ariège, Aube, Aude, Aveyron, Bouches-du-Rhône, Calvados, Cantal, Charente, Charente-Maritime, Corrèze, Corse, Côte-d'Or, Côtes-d'Armor, Creuse, Dordogne, Drôme, Eure-et-Loir, Finistère, Gard, Haute-Garonne, Gers, Gironde, Hérault, Ille-et-Vilaine, Indre-et-Loire, Isère, Jura, Landes, Loire, Haute-Loire, Loire-Atlantique, Loiret, Lot, Lot-et-Garonne, Maine-et-Loire, Manche, Marne, Meurthe-et-Moselle, Meuse, Morbihan, Moselle, Nord, Oise, Pas-de-Calais, Puy-de-Dôme, Pyrénées-Atlantiques, Hautes-Pyrénées, Pyrénées-Orientales, Bas-Rhin, Haut-Rhin, Rhône, Haute-Saône, Saône-et-Loire, Savoie, Haute-Savoie, Paris, Seine-Maritime, Seine-et-Marne, Yvelines, Deux-Sèvres, Somme, Tarn, Tarn-et-Garonne, Var, Vaucluse, Vendée, Vienne, Haute-Vienne, Yonne, Territoire-de-Belfort, Hauts-de-Seine | NA | NA | NA | NA | NA | NA | NA | ECDC (2022b) |
|  | Ain, Isère, Rhône, Savoie | 1966-1976 | NA | NA | NA | NA | NA | NA | Gilot et al. (1976) |
|  | Aisne, Var, Dordogne, Savoie, Pyrénées-Orientales | 1944, 1952, 2004, 2012, 2016-2018 | NA | January, May, August, September | Winter,  Spring,  Summer | NA | NA | NA | INPN (2023) |
|  | All France | 1994 | NA | NA | NA | NA | NA | NA | Jetten et al. (1994) |
|  | Bouches-du-Rhône | 2006 | 11 | February, March, April, May, June, July, August, September, October, November, December | Winter,  Spring,  Summer,  Autumn | 0-50 | CDC trap  with CO2,  Ovitrap,  Human landing  capture | NA | l'Ambert et al. (2012) |
|  | Loire-Atlantique | 1988 | 4 | May, June, August, September | Summer | 6-10 | Human landing  Capture  Hosted capture | NA | Le Goff et al. (1990) |
|  | Bouches-du-Rhône | 1920 | NA | August | Summer | NA | NA | NA | Leger (1920) |
|  | Marne, Haut-Rhin, Moselle | 2017-2020 | NA | January, February, March, April, May, June, July, August, September, October, November, December | Winter,  Spring,  Summer,  Autumn | NA | CDC trap,  BG-Sentinel,  Larvae capture,  Aspirator | NA | Martinet (2021) |
|  | Corse | NA | NA | NA | NA | NA | NA | NA | Mehlhorn (2012) |
|  | Bouches-du-Rhône | 1965-1967 | 15 | December, January, February, March, April | Winter | NA | NA | NA | Mouchet et al. (1969) |
|  | Bouches-du-Rhône | 1964-1968 | 10 | February, March, April, May, June, July, August, September, October, December | Winter,  Spring,  Summer,  Autumn | NA | NA | NA | Mouchet et al. (1970) |
|  | All France | NA | NA | NA | NA | NA | NA | NA | Moussiegt (1986) |
|  | Gard, Bouches-du-Rhône | 2005 | 10 | March, April, May, June, July, August, September, October, November, December | NA | NA | Larvae and  adult capture | NA | Ponçon (2008) |
|  | Gard, Bouches-du-Rhône | 2005 | 8 | March, April, May, June, July, August, September, October | Spring,  Summer,  Autumn | NA | CDC trap | NA | Ponçon (2008) |
|  | All France | 1963-2007 | NA | NA | NA | NA | NA | NA | Ponçon (2008) |
|  | Hérault, Gard, Bouches-du-Rhône | 1953-1966 | NA | January, February, March, April, May, June, July, August, September, October, November, December | Winter,  Spring,  Summer,  Autumn | NA | Larvae capture,  CDC trap | NA | Rageau et al. (1967) |
|  | Ain, Aisne, Allier, Alpes-de-Haute-Provence, Hautes-Alpes, Alpes-Maritimes, Ardèche, Ardennes, Ariège, Aude, Bouches-du-Rhône, Calvados, Cantal, Charente, Charente-Maritime, Corrèze, Corse, Côte-d'Or, Côtes-d'Armor, Creuse, Dordogne, Drôme, Finistère, Gard, Haute-Garonne, Gironde, Hérault, Ille-et-Vilaine, Indre-et-Loire, Isère, Loire, Haute-Loire, Loire-Atlantique, Loiret, Maine-et-Loire, Manche, Meuse, Morbihan, Nord, Oise, Puy-de-Dôme, Pyrénées-Atlantiques, Hautes-Pyrénées, Pyrénées-Orientales, Bas-Rhin, Haut-Rhin, Rhône, Saône-et-Loire, Savoie, Haute-Savoie, Paris, Seine-et-Marne, Yvelines, Deux-Sèvres, Somme, Var, Vaucluse, Vendée, Vienne, Yonne, Territoire-de-Belfort, Essonne, Hauts-de-Seine, Seine-Saint-Denis, Val-de-Marne, Val-d'Oise | NA | NA | NA | NA | NA | NA | NA | Rageau et al. (1970b) |
|  | All France | NA | NA | NA | NA | NA | NA | NA | Ramsdale et al. (2000) |
|  | Hérault, Gard, Bouches-du-Rhône | 1965 | 1 | April | Spring | NA | Egg collection | NA | Rioux et al. (1967) |
|  | Paris, Hauts-de-Seine, Isère, Essonne, Rhône, Yonne, Yvelines, Somme, Ain | NA | NA | NA | NA | NA | NA | NA | Roubaud (1918) |
|  | Vendée, Paris, Essonne, Corse | 1919 | NA | June, July, August, February | Summer,  Winter | NA | Larvae and  adult capture | *Plasmodium vivax* | Roubaud (1920) |
|  | Vendée, Paris, Savoie, Marne, Ardennes, Meuse, Corse | 1920 | NA | NA | NA | NA | NA | *Plasmodium* sp. | Roubaud (1921) |
|  | Corse, Vendée | 1921-1927 | NA | NA | Summer | NA | NA | NA | Roubaud (1928) |
|  | Aveyron, Lot, Dordogne, Ariège, Haute-Garonne, Tarn, Tarn-et-Garonne, Lot-et-Garonne, Gers | NA | NA | March, April, May, June, July, August, September, October, November, December, January | Spring,  Summer,  Autumn,  Winter | NA | Larvae capture | NA | Ruffié (1957) |
|  | Bouches-du-Rhône | 1943 | NA | August, November | NA | NA | NA | NA | Sautet (1944) |
|  | All France | 1902-1921 | NA | January, February, March, April, May, June, July, August, September, October, November, December | Winter,  Spring,  Summer,  Autumn | NA | NA | NA | Séguy (1923) |
|  | All France | NA | NA | NA | NA | NA | NA | NA | Séguy (1924) |
|  | Corse, Bouches-du-Rhône | NA | NA | NA | NA | NA | NA | NA | Takken et al. (2007) |
|  | Bouches-du-Rhône | 1936 | NA | NA | Summer | 0-7 | Larvae and  adult capture | NA | Treillard (1937) |
|  | Gard, Hérault, Bouches-du-Rhône | 2001 | 7 | April, May, June, July, August, September, October | Spring,  Summer,  Autumn | NA | CDC trap  With CO2 | NA | Zientara et al. (2004) |
|  | Bouches-du-Rhône, Gard | NA | NA | NA | NA | NA | Larvae and  adult capture | NA | Rioux et al. (1955) |
|  | Pyrénées-Orientales, Haute-Garonne, Var, Dordogne, Savoie, Saône-et-Loire, Haut-Rhin, Oise, Aisne, Hérault, Bouches-du-Rhône, Seine-Saint-Denis, Charente-Maritime, Haut-Rhin, Corse | 1944, 1952, 2004, 2012, 2016-2018, 2022 | NA | January, May, July, August, September | Winter,  Spring,  Summer | NA | NA | NA | GBIF (2023) |
| *Anopheles*  *marteri* | Corse | 1944, 1946-1950, 1952 | NA | NA | NA | NA | NA | NA | Aitken (1954) |
|  | Corse | NA | NA | NA | NA | NA | NA | NA | Moussiegt (1986) |
|  | Corse | NA | NA | NA | NA | NA | NA | NA | Rageau et al. (1970b) |
|  | Corse | NA | NA | NA | NA | NA | NA | NA | Ramsdale et al. (2000) |
| *Anopheles*  *petragnani* | Hautes-Pyrénées, Gers, Haute-Garonne, Tarn-et-Garonne, Tarn, Aude, Pyrénées-Orientales, Aveyron, Hérault, Lozère, Gard, Ardèche, Drôme, Hautes-Alpes, Alpes-de-Haute-Provence, Alpes-Maritimes, Var, Vaucluse, Bouches-du-Rhône, Corse | 1963-2007 | NA | NA | NA | NA | NA | NA | Ponçon (2008) |
|  | Corse | NA | NA | NA | NA | NA | NA | NA | Ramsdale et al. (2000) |
|  | Gard | NA | NA | NA | NA | NA | NA | NA | GBIF (2023) |
| *Anopheles*  *plumbeus* | Loire-Atlantique | 1993 | NA | NA | NA | NA | Larvae capture | NA | Brutus et al. (1993) |
|  | Corse | 1944, 1946-1950, 1952 | NA | NA | NA | NA | NA | NA | Aitken (1954) |
|  | Bouches-du-Rhône, Hérault | 2004 | 6 | May, June, July, August, September, October | Spring,  Summer,  Autumn | 1, 25 | Hosted capture | NA | Balenghien et al. (2006) |
|  | Indre-et-Loire | 1936-1943 | NA | January, February, March, April, May, June, July, August, September, October, November, December | Winter,  Spring,  Summer,  Autumn | NA | NA | NA | Callot et al. (1944) |
|  | Paris, Essonne, Hauts-de-Seine, Seine-Saint-Denis, Val-de-Marne, Val-d'Oise, Seine-et-Marne, Yvelines, Oise, Aisne, Marne, Ardennes, Seine-Maritime, Calvados, Orne, Haute-Marne, Côte-d'Or, Saône-et-Loire, Loire, Rhône, Ain, Puy-de-Dôme, Savoie, Isère, Drôme, Alpes-Maritimes, Var, Bouches-du-Rhône, Gard, Hérault, Aveyron, Pyrénées-Orientales, Ariège, Haute-Garonne, Hautes-Pyrénées, Pyrénées-Atlantiques, Dordogne, Charente-Maritime, Vendée, Deux-Sèvres, Vienne, Indre-et-Loire, Loire-Atlantique | NA | NA | NA | NA | NA | NA | NA | ECDC (2022b) |
|  | Ain, Isère, Rhône, Savoie | 1966-1976 | NA | NA | NA | NA | NA | NA | Gilot et al. (1976) |
|  | Hérault | 1955 | 1 | June | Summer | 27 | NA | NA | Harant et al. (1955) |
|  | Pyrénées-Orientales, Pyrénées-Atlantiques, Savoie, Dordogne | 1955, 1992, 2004, 2016-2017, 2020 | NA | January, July, August | Winter,  Summer | NA | NA | NA | INPN (2023) |
|  | Marne, Moselle | 2017-2020 | NA | January, February, March, April, May, June, July, August, September, October, November, December | Winter,  Spring,  Summer,  Autumn | NA | CDC trap,  BG-Sentinel,  Larvae capture,  Aspirator | NA | Martinet (2021) |
|  | Marne | 2018, 2020 | 4 | March, May, September, October | Spring,  Autumn | NA | Larvae and  adult capture | NA | Martinet (2021) |
|  | Ain, Aisne, Alpes-Maritimes, Ardennes, Ariège, Aveyron, Bouches-du-Rhône, Calvados, Charente-Maritime, Corse, Côte-d'Or, Dordogne, Drôme, Gard, Haute-Garonne, Hérault, Indre-et-Loire, Isère, Loire, Loire-Atlantique, Marne, Haute-Marne, Oise, Puy-de-Dôme, Pyrénées-Atlantiques, Hautes-Pyrénées, Pyrénées-Orientales, Bas-Rhin, Haut-Rhin, Rhône, Savoie, Seine-et-Marne, Deux-Sèvres, Var, Vendée, Vienne, Yvelines, Territoire de Belfort, Paris | NA | NA | NA | NA | NA | NA | NA | Moussiegt (1986) |
|  | All France | 1963-2007 | NA | NA | NA | NA | NA | NA | Ponçon (2008) |
|  | Hérault, Gard, Bouches-du-Rhône | 1953-1966 | NA | January, February, March, April, May, June, July, August, September, October, November, December | Winter,  Spring,  Summer,  Autumn | NA | Larvae capture,  CDC trap | NA | Rageau et al. (1967) |
|  | Paris, Hauts-de-Seine, Seine-Saint-Denis, Val-de-Marne, Seine-et-Marne, Yvelines, Essonne, Val-d'Oise, Oise, Aisne, Ardennes, Marne, Calvados, Haute-Marne, Vienne, Bas-Rhin, Haut-Rhin, Dordogne, Corse, Pyrénées-Atlantiques, Hautes-Pyrénées, Haute-Garonne, Pyrénées-Orientales, Ain, Loire, Rhône, Isère, Alpes-Maritimes, Var, Bouches-du-Rhône, Gard, Hérault, Aveyron | NA | NA | NA | NA | NA | NA | NA | Rageau et al. (1970b) |
|  | Ain, Aisne, Alpes-Maritimes, Ardennes, Ariège, Aveyron, Bouches-du-Rhône, Calvados, Charente-Maritime, Côte-d'Or, Dordogne, Drôme, Gard, Haute-Garonne, Hérault, Indre-et-Loire, Isère, Loire, Loire-Atlantique, Marne, Haute-Marne, Oise, Puy-de-Dôme, Pyrénées-Atlantiques, Haut-Pyrénées, Pyrénées-Orientales, Bas-Rhin, Haut-Rhin, Rhône, Savoie, Seine-et-Marne, Deux-Sèvres, Var, Vendée, Vienne, Yvelines, Territoire de Belfort, Paris, Corse | NA | NA | NA | NA | NA | NA | NA | Ramsdale et al. (2000) |
|  | Hérault, Gard, Bouches-du-Rhône | 1965 | 1 | April | Spring | NA | Egg collection | NA | Rioux et al. (1967) |
|  | Paris | 1919 | NA | June, July, August, February | Summer,  Winter | NA | Larvae and  adult capture | NA | Roubaud (1920) |
|  | Aveyron, Lot, Dordogne, Ariège, Haute-Garonne, Tarn, Tarn-et-Garonne, Lot-et-Garonne, Gers | NA | NA | March, April, May, June, July, August, September, October, November, December, January | Spring,  Summer,  Autumn,  Winter | NA | Larvae capture | NA | Ruffié (1957) |
|  | Orne | 1999 | 0.1 | October | Autumn | 145 | Larvae capture | NA | Schaffner et al. (2000) |
|  | All France | 1902-1921 | NA | January, February, March, April, May, June, July, August, September, October, November, December | Winter,  Spring,  Summer,  Autumn | NA | NA | NA | Séguy (1923) |
|  | All France | NA | NA | NA | NA | NA | NA | NA | Séguy (1924) |
|  | Corse, Bouches-du-Rhône | NA | NA | NA | NA | NA | NA | NA | Takken et al. (2007) |
|  | Bouches-du-Rhône, Gard | NA | NA | NA | NA | NA | Larvae and  adult capture | NA | Rioux et al. (1955) |
|  | Pyrénées-Orientales, Pyrénées-Atlantiques, Dordogne, Isère, Savoie, Haute-Savoie, Oise, Hérault | 1955, 1992, 2002, 2004, 2016-2017, 2022 | NA | January, July, August, September | Winter,  Summer | NA | NA | NA | GBIF (2023) |
| *Anopheles*  *pseudopictus* | Corse | NA | NA | NA | NA | NA | NA | NA | Ponçon (2008) |
|  | Hérault, Bouches-du-Rhône | 2005 | NA | January | Winter | NA | NA | NA | GBIF (2023) |
| *Anopheles*  *sinensis* | Bouches-du-Rhône | 1920 | NA | August | Summer | NA | NA | NA | Leger (1920) |
|  | Bouches-du-Rhône | 1902-1921 | NA | January, February, March, April, May, June, July, August, September, October, November, December | Winter,  Spring,  Summer,  Autumn | NA | NA | NA | Séguy (1923) |
| *Anopheles*  *superpictus* | Corse | 1944, 1946-1950, 1952 | NA | NA | NA | NA | NA | NA | Aitken (1954) |
|  | Corse | NA | NA | NA | NA | NA | NA | NA | Moussiegt (1986) |
|  | Corse | NA | NA | NA | NA | NA | NA | NA | Rageau et al. (1970b) |
|  | Corse | NA | NA | NA | NA | NA | NA | NA | Ramsdale et al. (2000) |
|  | Corse | NA | NA | NA | NA | NA | NA | NA | Takken et al. (2007) |
|  | Corse | 1994 | NA | NA | NA | NA | NA | NA | Jetten et al. (1994) |
| *Coquillettidia*  *buxtoni* | Corse | 1944, 1946-1950, 1952 | NA | NA | NA | NA | NA | NA | Aitken (1954) |
|  | Ain | 1966-1976 | NA | NA | NA | NA | NA | NA | Gilot et al. (1976) |
|  | Savoie | 2016-2017 | NA | January | Winter | NA | NA | NA | INPN (2023) |
|  | Ain, Aude, Bouches-du-Rhône, Charente-Maritime, Corse, Haute-Garonne, Gironde, Hérault, Isère, Rhône | NA | NA | NA | NA | NA | NA | NA | Moussiegt (1986) |
|  | Corse, Bouches-du-Rhône, Charente-Maritime, Rhône | NA | NA | NA | NA | NA | NA | NA | Rageau et al. (1970b) |
|  | Savoie | 2016-2017 | NA | NA | NA | NA | NA | NA | GBIF (2023) |
| *Coquillettidia*  *richiardii* | Savoie | 1983-1985 | 12 | May, June, July | Summer | 310, 1538 | Human landing  capture,  Net capture | NA | Chastel et al. (1987) |
|  | Corse | 1944, 1946-1950, 1952 | NA | NA | NA | NA | NA | NA | Aitken (1954) |
|  | Bouches-du-Rhône | 2004 | 6 | May, June, July, August, September, October | Spring,  Summer,  Autumn | 1, 25 | Hosted capture | NA | Balenghien et al. (2006) |
|  | Corse | 1924 | 2 | June, July | Summer | NA | NA | NA | Brumpt (1925) |
|  | Indre-et-Loire | 1936-1943 | NA | January, February, March, April, May, June, July, August, September, October, November, December | Winter,  Spring,  Summer,  Autumn | NA | NA | NA | Callot et al. (1944) |
|  | Manche, Indre-et-Loire, Vienne, Charente-Maritime, Gironde, Oise, Aisne, Marne, Ardennes, Bas-Rhin, Haut-Rhin, Haute-Saône, Territoire-de-Belfort, Rhône, Ain, Isère, Haute-Savoie, Savoie, Drôme, Bouches-du-Rhône, Gard, Hérault, Aude, Pyrénées-Orientales, Haute-Garonne, Seine-et-Marne, Yvelines, Corse | NA | NA | NA | NA | NA | NA | NA | ECDC (2022b) |
|  | Ain, Isère, Rhône, Savoie, Drôme | 1966-1976 | NA | NA | NA | NA | NA | NA | Gilot et al. (1976) |
|  | Savoie | 2016-2017 | NA | January | Winter | NA | NA | NA | INPN (2023) |
|  | Bouches-du-Rhône | 2006 | 11 | February, March, April, May, June, July, August, September, October, November, December | Winter,  Spring,  Summer,  Autumn | 0-50 | CDC trap  with CO2,  Ovitrap,  Human landing  capture | NA | l'Ambert et al. (2012) |
|  | Marne | 2017-2020 | NA | January, February, March, April, May, June, July, August, September, October, November, December | Winter,  Spring,  Summer,  Autumn | NA | CDC trap,  BG-Sentinel,  Larvae capture,  Aspirator | NA | Martinet (2021) |
|  | Ain, Aisne, Ardennes, Aude, Bouches-du-Rhône, Charente-Maritime, Corse, Drôme, Gard, Haute-Garonne, Gironde, Hérault, Indre-et-Loire, Isère, Marne, Oise, Pyrénées-Orientales, Bas-Rhin, Haut-Rhin, Rhône, Haute-Saône, Savoie, Haute-Savoie, Seine-et-Marne, Vienne, Yvelines, Territoire de Belfort, Paris, Manche | NA | NA | NA | NA | NA | NA | NA | Moussiegt (1986) |
|  | Gard, Bouches-du-Rhône | 2005 | 8 | March, April, May, June, July, August, September, October | Spring,  Summer,  Autumn | NA | CDC trap | NA | Ponçon (2008) |
|  | Hérault, Gard, Bouches-du-Rhône | 1953-1966 | NA | January, February, March, April, May, June, July, August, September, October, November, December | Winter,  Spring,  Summer,  Autumn | NA | Larvae capture,  CDC trap | NA | Rageau et al. (1967) |
|  | Paris, Hauts-de-Seine, Seine-Saint-Denis, Val-de-Marne, Yvelines, Essonne, Val-d'Oise, Seine-et-Marne, Oise, Aisne, Haut-Rhin, Bas-Rhin, Haute-Saône, Vienne, Indre-et-Loire, Charente-Maritime, Isère, Savoie, Ain, Rhône, Pyrénées-Orientales, Aude, Hérault, Gard, Bouches-du-Rhône, Corse | NA | NA | NA | NA | NA | NA | NA | Rageau et al. (1970b) |
|  | All France | 1902-1921 | NA | January, February, March, April, May, June, July, August, September, October, November, December | Winter,  Spring,  Summer,  Autumn | NA | NA | NA | Séguy (1923) |
|  | All France | NA | NA | NA | NA | NA | NA | NA | Séguy (1924) |
|  | Savoie, Bouches-du-Rhône | 2016-2017, 2020 | NA | July | Summer | NA | NA | NA | GBIF (2023) |
| *Culex*  *apicalis* | Indre-et-Loire | 1936-1943 | NA | January, February, March, April, May, June, July, August, September, October, November, December | Winter,  Spring,  Summer,  Autumn | NA | NA | NA | Callot et al. (1944) |
|  | Essonne, Pyrénées-Orientales, Gironde | NA | NA | NA | NA | NA | NA | NA | GBIF (2023) |
| *Culex*  *brumpti* | Corse | NA | NA | NA | NA | NA | NA | NA | Rageau et al. (1970b) |
| *Culex*  *hortensis* | Corse | 1944, 1946-1950, 1952 | NA | NA | NA | NA | NA | NA | Aitken (1954) |
|  | Indre-et-Loire | 1936-1943 | NA | January, February, March, April, May, June, July, August, September, October, November, December | Winter,  Spring,  Summer,  Autumn | NA | NA | NA | Callot et al. (1944) |
|  | Ain, Isère, Rhône, Savoie | 1966-1976 | NA | NA | NA | NA | NA | NA | Gilot et al. (1976) |
|  | Loiret, Lot-et-Garonne, Moselle, Pyrénées-Orientales, Savoie | 1955, 1958, 2014-2017, 2020 | NA | January, April, August, September, November | Winter,  Spring,  Summer,  Autumn | NA | NA | NA | INPN (2023) |
|  | Marne, Moselle, Haut-Rhin, Bas-Rhin | 2017-2020 | NA | January, February, March, April, May, June, July, August, September, October, November, December | Winter,  Spring,  Summer,  Autumn | NA | CDC trap,  BG-Sentinel,  Larvae capture,  Aspirator | NA | Martinet (2021) |
|  | Bouches-du-Rhône | 1965-1967 | 15 | December, January, February, March, April | Winter | NA | NA | NA | Mouchet et al. (1969) |
|  | Ain, Aisne, Allier, Hautes-Alpes, Alpes-Maritimes, Aude, Aveyron, Bouches-du-Rhône, Calvados, Cantal, Charente, Charente-Maritime, Corse, Côte-d'Or, Creuse, Dordogne, Doubs, Drôme, Finistère, Gard, Haute-Garonne, Gironde, Hérault, Ille-et-Vilaine, Indre, Indre-et-Loire, Isère, Jura, Loire, Haute-Loire, Loire-Atlantique, Maine-et-Loire, Manche, Mayenne, Morbihan, Nièvre, Oise, Puy-de-Dôme, Pyrénées-Atlantiques, Hautes-Pyrénées, Pyrénées-Orientales, Bas-Rhin, Rhône, Haute-Saône, Saône-et-Loire, Sarthe, Savoie, Haute-Savoie, Seine-et-Marne, Deux-Sèvres, Tarn, Var, Vaucluse, Vendée, Vienne, Vosges, Yvelines, Paris | NA | NA | NA | NA | NA | NA | NA | Moussiegt (1986) |
|  | Ain, Aisne, Allier, Hautes-Alpes, Alpes-Maritimes, Ariège, Aude, Aveyron, Bouches-du-Rhône, Calvados, Cantal, Charente, Charente-Maritime, Corse, Côte-d’Or, Côtes-d’Armor, Creuse, Dordogne, Drôme, Finistère, Gard, Haute-Garonne, Hérault, Ille-et-Vilaine, Indre, Indre-et-Loire, Isère, Loire, Haute-Loire, Loire-Atlantique, Maine-et-Loire, Manche, Mayenne, Morbihan, Nièvre, Oise, Puy-de-Dôme, Pyrénées-Atlantiques, Hautes-Pyrénées, Pyrénées-Orientales, Bas-Rhin, Rhône, Haute-Saône, Saône-et-Loire, Savoie, Haute-Savoie, Paris, Seine-et-Marne, Yvelines, Deux-Sèvres, Var, Vaucluse, Vendée, Vienne, Essonne, Hauts-de-Seine, Seine-Saint-Denis, Val-de-Marne, Val-d’Oise | NA | NA | NA | NA | NA | NA | NA | Rageau et al. (1970b) |
|  | Aveyron, Lot, Dordogne, Ariège, Haute-Garonne, Tarn, Tarn-et-Garonne, Lot-et-Garonne, Gers | NA | NA | March, April, May, June, July, August, September, October, November, December, January | Spring,  Summer,  Autumn,  Winter | NA | Larvae capture | NA | Ruffié (1957) |
|  | Orne | 1999 | 0.1 | October | Autumn | 145 | Larvae capture | NA | Schaffner et al. (2000) |
|  | All France | 1902-1921 | NA | January, February, March, April, May, June, July, August, September, October, November, December | Winter,  Spring,  Summer,  Autumn | NA | NA | *Plasmodium relictum* | Séguy (1923) |
|  | All France | NA | NA | NA | NA | NA | NA | NA | Séguy (1924) |
|  | Bouches-du-Rhône, Gard | NA | NA | NA | NA | NA | Larvae and  adult capture | NA | Rioux et al. (1955) |
|  | Alpes-Maritimes, Bouches-du-Rhône, Côte-d'Or, Essonne, Gard, Hérault, Loiret, Lot-et-Garonne, Morbihan, Moselle, Pyrénées-Orientales, Saône-et-Loire, Savoie | 1955, 1958, 2014, 2016-2018, 2020 | NA | January, April, August, September, October | Winter,  Spring,  Summer,  Autumn | NA | NA | NA | GBIF (2023) |
| *Culex*  *impudicus* | Loire-Atlantique | 1993 | NA | NA | NA | NA | Larvae capture | NA | Brutus et al. (1993) |
|  | Corse | 1944, 1946-1950, 1952 | NA | NA | NA | NA | NA | NA | Aitken (1954) |
|  | Pyrénées-Orientales | 1955, 1958 | NA | January, April, August, September | Winter,  Spring,  Summer | NA | NA | NA | INPN (2023) |
|  | Bouches-du-Rhône | 1965-1967 | 15 | December, January, February, March, April | Winter | NA | NA | NA | Mouchet et al. (1969) |
|  | Bouches-du-Rhône | 1964-1968 | 10 | February, March, April, May, June, July, August, September, October, December | Winter,  Spring,  Summer,  Autumn | NA | NA | NA | Mouchet et al. (1970) |
|  | Alpes-Maritimes, Aude, Bouches-du-Rhône, Charente-Maritime, Corse, Dordogne, Drôme, Gard, Haute-Garonne, Gironde, Hérault, Hautes-Pyrénées, Pyrénées-Orientales, Var, Vaucluse, Vendée | NA | NA | NA | NA | NA | NA | NA | Moussiegt (1986) |
|  | Dordogne, Corse, Hautes-Pyrénées, Haute-Garonne, Pyrénées-Orientales, Aude, Hérault, Gard, Bouches-du-Rhône, Var, Alpes-Maritimes, Vaucluse, Drôme | NA | NA | NA | NA | NA | NA | NA | Rageau et al. (1970b) |
|  | Aveyron, Lot, Dordogne, Ariège, Haute-Garonne, Tarn, Tarn-et-Garonne, Lot-et-Garonne, Gers | NA | NA | March, April, May, June, July, August, September, October, November, December, January | Spring,  Summer,  Autumn,  Winter | NA | Larvae capture | NA | Ruffié (1957) |
|  | Bouches-du-Rhône, Gard | NA | NA | NA | NA | NA | Larvae and  adult capture | NA | Rioux et al. (1955) |
|  | Hérault, Pyrénées-Orientales | 1955, 1958 | NA | January, April, August, September | Winter,  Spring,  Summer | NA | NA | NA | GBIF (2023) |
| *Culex*  *laticinctus* | Corse | 1944, 1946-1950, 1952 | NA | NA | NA | NA | NA | NA | Aitken (1954) |
|  | Corse, Seine-et-Marne, Var | NA | NA | NA | NA | NA | NA | NA | Moussiegt (1986) |
|  | Seine-et-Marne | 1902-1921 | NA | January, February, March, April, May, June, July, August, September, October, November, December | Winter,  Spring,  Summer,  Autumn | NA | NA | NA | Séguy (1923) |
| *Culex*  *martinii* | Bouches-du-Rhône, Gard, Hérault | NA | NA | NA | NA | NA | NA | NA | Moussiegt (1986) |
|  | Hérault, Gard, Bouches-du-Rhône | 1953-1966 | NA | January, February, March, April, May, June, July, August, September, October, November, December | Winter,  Spring,  Summer,  Autumn | NA | Larvae capture,  CDC trap | NA | Rageau et al. (1967) |
|  | Hérault, Gard, Bouches-du-Rhône | NA | NA | NA | NA | NA | NA | NA | Rageau et al. (1970b) |
|  | Corse | NA | NA | NA | NA | NA | NA | NA | GBIF (2023) |
| *Culex*  *mimeticus* | Corse | 1944, 1946-1950, 1952 | NA | NA | NA | NA | NA | NA | Aitken (1954) |
|  | Alpes-Maritimes, Ardèche, Aude, Bouches-du-Rhône, Corse, Gard, Hérault, Haute-Loire, Pyrénées-Orientales, Var | NA | NA | NA | NA | NA | NA | NA | Moussiegt (1986) |
|  | Hérault, Gard, Bouches-du-Rhône | 1953-1966 | NA | January, February, March, April, May, June, July, August, September, October, November, December | Winter,  Spring,  Summer,  Autumn | NA | Larvae capture,  CDC trap | NA | Rageau et al. (1967) |
|  | Corse, Pyrénées-Orientales, Aude, Hérault, Gard, Bouches-du-Rhône, Var, Alpes-Maritimes, Ardèche, Haute-Loire | NA | NA | NA | NA | NA | NA | NA | Rageau et al. (1970b) |
|  | Bouches-du-Rhône | 1902-1921 | NA | January, February, March, April, May, June, July, August, September, October, November, December | Winter,  Spring,  Summer,  Autumn | NA | NA | NA | Séguy (1923) |
|  | Bouches-du-Rhône, Gard | NA | NA | NA | NA | NA | Larvae and  adult capture | NA | Rioux et al. (1955) |
| *Culex*  *modestus* | Loire-Atlantique | 1993 | NA | NA | NA | NA | Larvae capture | NA | Brutus et al. (1993) |
|  | Corse | 1944, 1946-1950, 1952 | NA | NA | NA | NA | NA | NA | Aitken (1954) |
|  | Bouches-du-Rhône, Hérault | 2004 | 6 | May, June, July, August, September, October | Spring,  Summer,  Autumn | 1, 25 | Hosted capture | NA | Balenghien et al. (2006) |
|  | Indre-et-Loire | 1936-1943 | NA | January, February, March, April, May, June, July, August, September, October, November, December | Winter,  Spring,  Summer,  Autumn | NA | NA | NA | Callot et al. (1944) |
|  | Bouches-du-Rhône | 2011-2014 | 48 | January, February, March, April, May, June, July, August, September, October, November, December | Winter,  Spring,  Summer,  Autumn | 0-3 | CDC trap  with CO2 | NA | Chaskopoulou et al. (2016) |
|  | Loire-Atlantique, Vendée, Charente-Maritime, Gironde, Indre-et-Loire, Vienne, Eure, Yvelines, Haut-Rhin, Ain, Rhône, Loire, Saône-et-Loire, Alpes-de-Haute-Provence, Haute-Garonne, Corse, Aude, Pyrénées-Orientales, Hérault, Gard, Bouches-du-Rhône | NA | NA | NA | NA | NA | NA | NA | ECDC (2022b) |
|  | Ain, Isère, Rhône, Savoie | 1966-1976 | NA | NA | NA | NA | NA | NA | Gilot et al. (1976) |
|  | Bouches-du-Rhône | 1964 | 3 | June, September, November | Summer,  Autumn | NA | NA | Virus du Nil Occidental | Hannoun et al. (1964) |
|  | Côtes-d'Armor | 2010 | NA | January | Winter | NA | NA | NA | INPN (2023) |
|  | Bouches-du-Rhône | 2006 | 11 | February, March, April, May, June, July, August, September, October, November, December | Winter,  Spring,  Summer,  Autumn | 0-50 | CDC trap  with CO2,  Ovitrap,  Human landing  capture | NA | l'Ambert et al. (2012) |
|  | Bouches-du-Rhône | NA | NA | NA | NA | NA | NA | NA | Martinet (2021) |
|  | Bouches-du-Rhône | 1965-1967 | 15 | December, January, February, March, April | Winter | NA | NA | Virus du Nil Occidental | Mouchet et al. (1969) |
|  | Indre-et-Loire, Loire, Vendée, Charente-Maritime, Gironde, Pyrénées-Orientales, Aude, Haute-Garonne, Hérault, Gard, Rhône, Ain, Bouches-du-Rhône | 1964-1968 | 10 | February, March, April, May, June, July, August, September, October, December | Winter,  Spring,  Summer,  Autumn | NA | NA | Virus du Nil Occidental | Mouchet et al. (1970) |
|  | Ain, Aude, Bouches-du-Rhône, Charente-Maritime, Corse, Gard, Haute-Garonne, Gironde, Hérault, Indre-et-Loire, Loire, Loire-Atlantique Pyrénées-Orientales, Rhône, Vendée, Vienne | NA | NA | NA | NA | NA | NA | NA | Moussiegt (1986) |
|  | Gard, Bouches-du-Rhône | 2005 | 8 | March, April, May, June, July, August, September, October | Spring,  Summer,  Autumn | NA | CDC trap | NA | Ponçon (2008) |
|  | Bouches-du-Rhône | 1969-2006 | NA | June, July, August, September, October | Summer,  Autumn | NA | Human landing  capture | NA | Ponçon et al. (2007a) |
|  | Ain | 2007 | 7 | April, May, June, July, August, September, October | Spring,  Summer,  Autumn | 285 | Larvae capture,  CDC trap | NA | Pradel et al. (2008) |
|  | Hérault, Gard, Bouches-du-Rhône | 1953-1966 | NA | January, February, March, April, May, June, July, August, September, October, November, December | Winter,  Spring,  Summer,  Autumn | NA | Larvae capture,  CDC trap | Virus du Nil Occidental | Rageau et al. (1967) |
|  | Vendée, Charente-Maritime, Gironde, Indre-et-Loire, Vienne, Loire, Rhône, Ain, Corse, Haute-Loire, Ardèche, Pyrénées-Orientales, Aude, Hérault, Gard, Bouches-du-Rhône, Var, Alpes-Maritimes | NA | NA | NA | NA | NA | NA | NA | Rageau et al. (1970b) |
|  | Hérault, Gard, Bouches-du-Rhône | 1965 | 1 | April | Spring | NA | Egg collection | NA | Rioux et al. (1967) |
|  | Aveyron, Lot, Dordogne, Ariège, Haute-Garonne, Tarn, Tarn-et-Garonne, Lot-et-Garonne, Gers | NA | NA | March, April, May, June, July, August, September, October, November, December, January | Spring,  Summer,  Autumn,  Winter | NA | Larvae capture | NA | Ruffié (1957) |
|  | Gard, Hérault, Bouches-du-Rhône | 2001 | 7 | April, May, June, July, August, September, October | Spring,  Summer,  Autumn | NA | CDC trap  with CO2 | NA | Zientara et al. (2004) |
|  | Bouches-du-Rhône, Gard | NA | NA | NA | NA | NA | Larvae and  adult capture | NA | Rioux et al. (1955) |
|  | Côtes-d'Armor, Bouches-du-Rhône, Oise, Gironde, Gard, Hérault | 2010, 2016 | NA | January, May, June, July | Winter,  Summer | NA | NA | NA | GBIF (2023) |
| *Culex*  *pipiens* | Morbihan, Loire-Atlantique | 1993 | NA | NA | NA | NA | Larvae capture | NA | Brutus et al. (1993) |
|  | Corse | 1944, 1946-1950, 1952 | NA | NA | NA | NA | NA | NA | Aitken (1954) |
|  | Bouches-du-Rhône, Hérault | 2004 | 6 | May, June, July, August, September, October | Spring,  Summer,  Autumn | 1, 25 | Hosted capture | NA | Balenghien et al. (2006) |
|  | Indre-et-Loire | 1936-1943 | NA | January, February, March, April, May, June, July, August, September, October, November, December | Winter,  Spring,  Summer,  Autumn | NA | NA | NA | Callot et al. (1944) |
|  | Bouches-du-Rhône | 2011-2014 | 48 | January, February, March, April, May, June, July, August, September, October, November, December | Winter,  Spring,  Summer,  Autumn | 0-3 | CDC trap  with CO2 | NA | Chaskopoulou et al. (2016) |
|  | Bouches-du-Rhône, Gard | 2015 | 4 | June, July, August, September | Summer,  Autumn | NA | CDC trap | Virus Usutu | Eiden et al. (2018) |
|  | Ain, Aisne, Allier, Alpes-de-Haute-Provence, Hautes-Alpes, Alpes-Maritimes, Ardèche, Ardennes, Ariège, Aube, Aude, Aveyron, Bouches-du-Rhône, Calvados, Cantal, Charente-Maritime, Corrèze, Corse, Côte-d'Or, Côtes-d'Armor, Creuse, Dordogne, Doubs, Drôme, Eure-et-Loir, Finistère, Gard, Haute-Garonne, Gironde, Hérault, Ille-et-Vilaine, Indre, Indre-et-Loire, Isère, Jura, Landes, Loir-et-Cher, Loire, Haute-Loire, Loire-Atlantique, Loiret, Lozère, Maine-et-Loire, Manche, Marne, Haute-Marne, Mayenne, Meurthe-et-Moselle, Meuse, Morbihan, Moselle, Nord, Oise, Pas-de-Calais, Puy-de-Dôme, Pyrénées-Atlantiques, Hautes-Pyrénées, Pyrénées-Orientales, Bas-Rhin, Haut-Rhin, Rhône, Haute-Saône, Saône-et-Loire, Sarthe, Savoie, Haute-Savoie, Seine-Maritime, Seine-et-Marne, Yvelines, Deux-Sèvres, Somme, Tarn, Tarn-et-Garonne, Var, Vaucluse, Vendée, Vienne, Haute-Vienne, Vosges, Yonne, Territoire-de-Belfort, Hauts-de-Seine | NA | NA | NA | NA | NA | NA | NA | ECDC (2022b) |
|  | Ain, Isère, Rhône, Savoie | 1966-1976 | NA | NA | NA | NA | NA | NA | Gilot et al. (1976) |
|  | Ain, Alpes-Maritimes, Bas-Rhin, Creuse, Essonne, Gironde, Hérault, Haute-Marne, Isère, Jura, Loiret, Loir-et-Cher, Morbihan, Moselle, Nord, Oise, Paris, Pyrénées-Atlantiques, Pyrénées-Orientales, Savoie, Seine-et-Marne, Somme, Val-d'Oise, Vendée, Yonne, Yvelines | 1758, 1952, 1955, 1958, 1965, 1969, 2000, 2002, 2004, 2006, 2008-2011, 2013-2021 | NA | January, February, March, April, May, June, July, August, September, October, November, December | Winter,  Spring,  Summer,  Autumn | NA | NA | NA | INPN (2023) |
|  | Bouches-du-Rhône | 2006 | 11 | February, March, April, May, June, July, August, September, October, November, December | Winter,  Spring,  Summer,  Autumn | 0-50 | CDC trap  with CO2,  Ovitrap,  Human landing  capture | NA | l'Ambert et al. (2012) |
|  | All France | 2021 | NA | NA | NA | NA | NA | Virus Usutu | Martinet (2021) |
|  | Marne, Moselle, Ardennes, Haute-Marne, Haut-Rhin, Bas-Rhin | 2017-2020 | NA | January, February, March, April, May, June, July, August, September, October, November, December | Winter,  Spring,  Summer,  Autumn | NA | CDC trap,  BG-Sentinel,  Larvae capture,  Aspirator | NA | Martinet (2021) |
|  | Bouches-du-Rhône | NA | NA | NA | NA | NA | NA | NA | Martinet (2021) |
|  | Marne, Ardennes | 2018, 2020 | 4 | March, May, September, October | Spring,  Autumn | NA | Larvae and  adult capture | NA | Martinet (2021) |
|  | Bouches-du-Rhône | 1965-1967 | 15 | December, January, February, March, April | Winter | NA | NA | NA | Mouchet et al. (1969) |
|  | Bouches-du-Rhône | 1964-1968 | 10 | February, March, April, May, June, July, August, September, October, December | Winter,  Spring,  Summer,  Autumn | NA | NA | NA | Mouchet et al. (1970) |
|  | Ain, Aisne, Allier, Hautes-Alpes, Alpes-Maritimes, Ardèche, Ardennes, Ariège, Aube, Aude, Aveyron, Bouches-du-Rhône, Calvados, Cantal, Charente-Maritime, Corrèze, Corse, Côte-d'Or, Côtes-d'Armor, Creuse, Dordogne, Drôme, Finistère, Gard, Haute-Garonne, Gironde, Hérault, Ille-et-Vilaine, Indre, Indre-et-Loire, Isère, Jura, Loir-et-Cher, Loire, Haute-Loire, Loire-Atlantique, Loiret, Maine-et-Loire, Manche, Marne, Mayenne, Morbihan, Moselle, Nord, Oise, Puy-de-Dôme, Pyrénées-Atlantiques, Hautes-Pyrénées, Pyrénées-Orientales, Bas-Rhin, Haut-Rhin, Rhône, Haute-Saône, Saône-et-Loire, Savoie, Haute-Savoie, Paris, Seine-Maritime, Seine-et-Marne, Yvelines, Deux-Sèvres, Somme, Tarn, Tarn-et-Garonne, Var, Vaucluse, Vendée, Vienne, Haute-Vienne, Vosges, Yonne, Territoire-de-Belfort, Essonne, Hauts-de-Seine, Seine-Saint-Denis, Val-de-Marne, Val-d'Oise | NA | NA | NA | NA | NA | NA | NA | Moussiegt (1986) |
|  | Bouches-du-Rhône, Hérault | 2005-2007 | NA | NA | NA | NA | Larvae capture | NA | Moutailler et al. (2008) |
|  | All France | NA | NA | NA | NA | NA | NA | NA | Mullen et al. (2009) |
|  | Gard, Bouches-du-Rhône | 2005 | 8 | March, April, May, June, July, August, September, October | Spring,  Summer,  Autumn | NA | CDC trap | NA | Ponçon (2008) |
|  | Hérault, Gard, Bouches-du-Rhône | 1953-1966 | NA | January, February, March, April, May, June, July, August, September, October, November, December | Winter,  Spring,  Summer,  Autumn | NA | Larvae capture,  CDC trap | NA | Rageau et al. (1967) |
|  | Ain, Aisne, Allier, Hautes-Alpes, Alpes-Maritimes, Ardèche, Ardennes, Ariège, Aube, Aude, Aveyron, Bouches-du-Rhône, Calvados, Cantal, Charente-Maritime, Corse, Côte-d’Or, Côtes-d’Armor, Creuse, Dordogne, Finistère, Gard, Haute-Garonne, Gironde, Hérault, Ille-et-Vilaine, Indre, Indre-et-Loire, Isère, Loire, Haute-Loire, Loire-Atlantique, Loiret, Maine-et-Loire, Manche, Marne, Mayenne, Morbihan, Oise, Puy-de-Dôme, Pyrénées-Atlantiques, Hautes-Pyrénées, Pyrénées-Orientales, Bas-Rhin, Haut-Rhin, Rhône, Haute-Saône, Saône-et-Loire, Savoie, Haute-Savoie, Paris, Seine-Maritime, Seine-et-Marne, Yvelines, Deux-Sèvres, Somme, Tarn-et-Garonne, Var, Vaucluse, Vendée, Vienne, Vosges, Yonne, Territoire-de-Belfort, Essonne, Hauts-de-Seine, Seine-Saint-Denis, Val-de-Marne, Val-d’Oise | NA | NA | NA | NA | NA | NA | NA | Rageau et al. (1970b) |
|  | Paris | 1919 | NA | June, July, August, February | Summer,  Winter | NA | Larvae and  adult capture | NA | Roubaud (1920) |
|  | Aveyron, Lot, Dordogne, Ariège, Haute-Garonne, Tarn, Tarn-et-Garonne, Lot-et-Garonne, Gers | NA | NA | March, April, May, June, July, August, September, October, November, December, January | Spring,  Summer,  Autumn,  Winter | NA | Larvae capture | NA | Ruffié (1957) |
|  | Orne | 1999 | 0.1 | October | Autumn | 145 | Larvae capture | NA | Schaffner et al. (2000) |
|  | All France | 1902-1921 | NA | January, February, March, April, May, June, July, August, September, October, November, December | Winter,  Spring,  Summer,  Autumn | NA | NA | *Dirofilaria immitis, Filaria brancrofti, Plasmodium danilewskyi* | Séguy (1923) |
|  | All France | NA | NA | NA | NA | NA | NA | NA | Séguy (1924) |
|  | Gard, Hérault, Bouches-du-Rhône | 2001 | 7 | April, May, June, July, August, September, October | Spring,  Summer,  Autumn | NA | CDC trap  with CO2 | NA | Zientara et al. (2004) |
|  | Bouches-du-Rhône, Gard | NA | NA | NA | NA | NA | Larvae and  adult capture | NA | Rioux et al. (1955) |
|  | Ain, Alpes-Maritimes, Bas-Rhin, Bouches-du-Rhône, Creuse, Essonne, Gard, Gironde, Haute-Garonne, Haute-Marne, Hérault, Isère, Jura, Loiret, Loir-et-Cher, Morbihan, Moselle, Nord, Oise, Paris, Pyrénées-Atlantiques, Pyrénées-Orientales, Savoie, Seine-et-Marne, Somme, Vendée, Yonne, Yvelines | 1758, 1937, 1947, 1952, 1955, 1958, 1965, 1969, 2000, 2002, 2004, 2007-2022 | NA | January, February, March, April, May, June, July, August, September, October, November, December | Winter,  Spring,  Summer,  Autumn | NA | NA | NA | GBIF (2023) |
| *Culex*  *territans* | Loire-Atlantique | 1993 | NA | NA | NA | NA | Larvae capture | NA | Brutus et al. (1993) |
|  | Ain, Isère, Rhône, Savoie | 1966-1976 | NA | NA | NA | NA | NA | NA | Gilot et al. (1976) |
|  | Isère, Savoie, Loiret | 1969, 2014, 2017 | NA | January, June, August | Winter,  Summer | NA | NA | NA | INPN (2023) |
|  | Ain, Alpes-Maritimes, Ariège, Aude, Aveyron, Bouches-du-Rhône, Cantal, Charente-Maritime, Corse, Côtes-d’Armor, Creuse, Dordogne, Haute-Garonne, Gironde, Indre-et-Loire, Isère, Haute-Loire, Loire-Atlantique, Maine-Et-Loire, Moselle, Oise, Pyrénées-Atlantiques, Hautes-Pyrénées, Pyrénées-Orientales, Bas-Rhin, Rhône, Savoie, Haute-Savoie, Seine-et-Marne, Somme, Tarn-et-Garonne, Var, Vienne, Haute-Vienne, Yvelines, Paris | NA | NA | NA | NA | NA | NA | NA | Moussiegt (1986) |
|  | Paris, Hauts-de-Seine, Seine-Saint-Denis, Val-de-Marne, Seine-et-Marne, Yvelines, Essonne, Val-d'Oise, Oise, Somme, Bas-Rhin, Côtes-d’Armor, Loire-Atlantique, Maine-et-Loire, Indre-et-Loire, Vienne, Charente-Maritime, Dordogne, Creuse, Rhône, Ain, Savoie, Haute-Savoie, Isère, Alpes-Maritimes, Haute-Loire, Cantal, Aveyron, Tarn-et-Garonne, Haute-Garonne, Pyrénées-Atlantiques, Hautes-Pyrénées, Ariège, Pyrénées-Orientales, Aude | NA | NA | NA | NA | NA | NA | NA | Rageau et al. (1970b) |
|  | Aveyron, Lot, Dordogne, Ariège, Haute-Garonne, Tarn, Tarn-et-Garonne, Lot-et-Garonne, Gers | NA | NA | March, April, May, June, July, August, September, October, November, December, January | Spring,  Summer,  Autumn,  Winter | NA | Larvae capture | NA | Ruffié (1957) |
|  | All France | 1902-1921 | NA | January, February, March, April, May, June, July, August, September, October, November, December | Winter,  Spring,  Summer,  Autumn | NA | NA | NA | Séguy (1923) |
|  | All France | NA | NA | NA | NA | NA | NA | NA | Séguy (1924) |
|  | Isère, Savoie, Loiret, Essonne, Hérault | 1969, 2014, 2017 | NA | June, August | Summer | NA | NA | NA | GBIF (2023) |
| *Culex*  *theileri* | Corse | 1944, 1946-1950, 1952 | NA | NA | NA | NA | NA | NA | Aitken (1954) |
|  | Bouches-du-Rhône, Hérault | 2004 | 6 | May, June, July, August, September, October | Spring,  Summer,  Autumn | 1, 25 | Hosted capture | NA | Balenghien et al. (2006) |
|  | Allier, Ariège, Aude, Bouches-du-Rhône, Charente-Maritime, Corse, Dordogne, Gard, Haute-Garonne, Gironde, Hérault, Jura, Pyrénées-Orientales, Bas-Rhin | NA | NA | NA | NA | NA | NA | NA | Moussiegt (1986) |
|  | Bouches-du-Rhône | 2005 | 8 | March, April, May, June, July, August, September, October | Spring,  Summer,  Autumn | NA | CDC trap | NA | Ponçon (2008) |
|  | Hérault, Gard, Bouches-du-Rhône | 1953-1966 | NA | January, February, March, April, May, June, July, August, September, October, November, December | Winter,  Spring,  Summer,  Autumn | NA | Larvae capture,  CDC trap | NA | Rageau et al. (1967) |
|  | Bas-Rhin, Jura, Allier, Charente-Maritime, Gironde, Dordogne, Haute-Garonne, Ariège, Pyrénées-Orientales, Hérault, Corse | NA | NA | NA | NA | NA | NA | NA | Rageau et al. (1970b) |
|  | Aveyron, Lot, Dordogne, Ariège, Haute-Garonne, Tarn, Tarn-et-Garonne, Lot-et-Garonne, Gers | NA | NA | March, April, May, June, July, August, September, October, November, December, January | Spring,  Summer,  Autumn,  Winter | NA | Larvae capture | NA | Ruffié (1957) |
|  | Bouches-du-Rhône, Gard | NA | NA | NA | NA | NA | Larvae and  adult capture | NA | Rioux et al. (1955) |
|  | Corse | NA | NA | NA | NA | NA | NA | NA | GBIF (2023) |
| *Culex*  *torrentium* | Ain, Isère, Rhône, Savoie | 1966-1976 | NA | NA | NA | NA | NA | NA | Gilot et al. (1976) |
|  | Marne | 2017-2020 | NA | January, February, March, April, May, June, July, August, September, October, November, December | Winter,  Spring,  Summer,  Autumn | NA | CDC trap,  BG-Sentinel,  Larvae capture,  Aspirator | NA | Martinet (2021) |
|  | Ain, Alpes-de-Haute-Provence, Hautes-Alpes, Ariège, Cantal, Corse, Côtes-d’Armor, Doubs, Eure-et-Loir, Finistère, Haute-Garonne, Gironde, Ille-et-Vilaine, Indre-et-Loire, Isère, Jura, Landes, Loir-et-Cher, Haute-Loire, Maine-et-Loire, Morbihan, Oise, Puy-de-Dôme, Pyrénées-Atlantiques, Hautes-Pyrénées, Pyrénées-Orientales, Bas-Rhin, Rhône, Sarthe, Savoie, Haute-Savoie, Seine-et-Marne, Vienne, Vosges, Yvelines, Paris | NA | NA | NA | NA | NA | NA | NA | Moussiegt (1986) |
|  | Finistère, Côtes-d’Armor, Morbihan, Ille-et-Vilaine, Maine-et-Loire, Sarthe, Vienne, Eure-et-Loir, Indre-et-Loire, Loir-et-Cher, Eure-et-Loir, Oise, Paris, Hauts-de-Seine, Seine-Saint-Denis, Val-de-Marne, Yvelines, Essonne, Val-d'Oise, Bas-Rhin, Vosges, Jura, Isère, Savoie, Haute-Savoie, Hautes-Alpes, Alpes-de-Haute-Provence, Puy-de-Dôme, Haute-Loire, Corse, Gard, Landes, Pyrénées-Atlantiques, Hautes-Pyrénées, Haute-Garonne, Ariège, Pyrénées-Orientales | NA | NA | NA | NA | NA | NA | NA | Rageau et al. (1970b) |
|  | Bouches-du-Rhône, Marne | 2007, 2017 | NA | January, May, July | Winter,  Spring,  Summer | NA | NA | NA | GBIF (2023) |
| *Culex*  *univittatus* | Corse | NA | NA | NA | NA | NA | NA | NA | Moussiegt (1986) |
| *Culiseta*  *alaskaensis* | Ain, Isère, Rhône, Savoie | 1966-1976 | NA | NA | NA | NA | NA | NA | Gilot et al. (1976) |
|  | Ain, Bas-Rhin | NA | NA | NA | NA | NA | NA | NA | Moussiegt (1986) |
|  | Bas-Rhin | NA | NA | NA | NA | NA | NA | NA | Rageau et al. (1970b) |
| *Culiseta*  *annulata* | Morbihan, Loire-Atlantique | 1993 | NA | NA | NA | NA | Larvae capture | NA | Brutus et al. (1993) |
|  | Corse | 1944, 1946-1950, 1952 | NA | NA | NA | NA | NA | NA | Aitken (1954) |
|  | Bouches-du-Rhône, Hérault | 2004 | 6 | May, June, July, August, September, October | Spring,  Summer,  Autumn | 1, 25 | Hosted capture | NA | Balenghien et al. (2006) |
|  | Indre-et-Loire | 1936-1943 | NA | January, February, March, April, May, June, July, August, September, October, November, December | Winter,  Spring,  Summer,  Autumn | NA | NA | NA | Callot et al. (1944) |
|  | Ain, Isère, Rhône, Savoie | 1966-1976 | NA | NA | NA | NA | NA | NA | Gilot et al. (1976) |
|  | Aisne, Bas-Rhin, Bouches-du-Rhône, Calvados, Finistère, Hérault, Haute-Savoie, Isère, Loiret, Manche, Morbihan, Nord, Oise, Pas-de-Calais, Rhône, Savoie, Val-d'Oise | 2009-2017, 2019-2022 | NA | January, February, April, June, July, August, September, October, November, December | Winter,  Spring,  Summer,  Autumn | NA | NA | NA | INPN (2023) |
|  | Bouches-du-Rhône | 2006 | 11 | February, March, April, May, June, July, August, September, October, November, December | Winter,  Spring,  Summer,  Autumn | 0-50 | CDC trap  with CO2,  Ovitrap,  Human landing  capture | NA | l'Ambert et al. (2012) |
|  | Marne, Moselle, Haut-Rhin, Bas-Rhin | 2017-2020 | NA | January, February, March, April, May, June, July, August, September, October, November, December | Winter,  Spring,  Summer,  Autumn | NA | CDC trap,  BG-Sentinel,  Larvae capture,  Aspirator | NA | Martinet (2021) |
|  | Bouches-du-Rhône | 1965-1967 | 15 | December, January, February, March, April | Winter | NA | NA | NA | Mouchet et al. (1969) |
|  | Bouches-du-Rhône | 1964-1968 | 10 | February, March, April, May, June, July, August, September, October, December | Winter,  Spring,  Summer,  Autumn | NA | NA | NA | Mouchet et al. (1970) |
|  | Ain, Allier, Hautes-Alpes, Alpes-Maritimes, Ardennes, Ariège, Aude, Aveyron, Bouches-du-Rhône, Calvados, Charente, Charente-Maritime, Corrèze, Côtes-d’Armor, Creuse, Dordogne, Finistère, Gard, Haute-Garonne, Gironde, Hérault, Ille-et-Vilaine, Indre-et-Loire, Isère, Landes, Loire, Haute-Loire, Loire-Atlantique, Loiret, Maine-et-Loire, Manche, Meuse, Morbihan, Moselle, Oise, Puy- de-Dôme, Pyrénées-Atlantiques, Hautes-Pyrénées, Pyrénées-Orientales, Bas-Rhin, Haut-Rhin, Rhône, Savoie, Haute-Savoie, Seine-et-Marne, Deux-Sèvres, Tarn, Var, Vaucluse, Vendée, Vienne, Haute-Vienne, Yvelines, Paris, Corse | NA | NA | NA | NA | NA | NA | NA | Moussiegt (1986) |
|  | Gard, Bouches-du-Rhône | 2005 | 8 | March, April, May, June, July, August, September, October | Spring,  Summer,  Autumn | NA | CDC trap | NA | Ponçon (2008) |
|  | Hérault, Gard, Bouches-du-Rhône | 1953-1966 | NA | January, February, March, April, May, June, July, August, September, October, November, December | Winter,  Spring,  Summer,  Autumn | NA | Larvae capture,  CDC trap | NA | Rageau et al. (1967) |
|  | Ain, Allier, Alpes-Maritimes, Ardennes, Ariège, Aude, Aveyron, Bouches-du-Rhône, Calvados, Charente-Maritime, Corse, Côtes-d’Armor, Dordogne, Finistère, Gard, Gironde, Hérault, Ille-et-Vilaine, Indre-et-Loire, Isère, Landes, Loire, Haute-Loire, Loire-Atlantique, Loiret, Maine-et-Loire, Manche, Morbihan, Oise, Puy-de-Dôme, Pyrénées-Atlantiques, Hautes-Pyrénées, Pyrénées-Orientales, Bas-Rhin, Haut-Rhin, Rhône, Savoie, Haute-Savoie, Paris, Seine-et-Marne, Yvelines, Tarn, Tarn-et-Garonne, Var, Vaucluse, Vendée, Vienne, Territoire-de-Belfort, Essonne, Hauts-de-Seine, Seine-Saint-Denis, Val-de-Marne, Val-d’Oise | NA | NA | NA | NA | NA | NA | NA | Rageau et al. (1970b) |
|  | Aveyron, Lot, Dordogne, Ariège, Haute-Garonne, Tarn, Tarn-et-Garonne, Lot-et-Garonne, Gers | NA | NA | March, April, May, June, July, August, September, October, November, December, January | Spring,  Summer,  Autumn,  Winter | NA | Larvae capture | NA | Ruffié (1957) |
|  | Orne | 1999 | 0.1 | October | Autumn | 145 | Larvae capture | NA | Schaffner et al. (2000) |
|  | All France | 1902-1921 | NA | January, February, March, April, May, June, July, August, September, October, November, December | Winter,  Spring,  Summer,  Autumn | NA | NA | NA | Séguy (1923) |
|  | All France | NA | NA | NA | NA | NA | NA | NA | Séguy (1924) |
|  | Bouches-du-Rhône, Gard | NA | NA | NA | NA | NA | Larvae and  adult capture | NA | Rioux et al. (1955) |
|  | Aisne, Alpes-de-Haute-Provence, Bas-Rhin, Bouches-du-Rhône, Calvados, Finistère, Gard, Gironde, Haute-Savoie, Haut-Rhin, Hérault, Indre, Isère, Loiret, Manche, Morbihan, Nord, Oise, Pas-de-Calais, Puy-de-Dôme, Pyrénées-Orientales, Rhône, Savoie, Val-d’Oise, Vendée | 2005, 2009, 2011, 2013-2017, 2019-2022 | NA | January, February, April, June, July, August, September, October, November, December | Winter,  Spring,  Summer,  Autumn | NA | NA | NA | GBIF (2023) |
| *Culiseta*  *fumipennis* | Indre-et-Loire | 1936-1943 | NA | January, February, March, April, May, June, July, August, September, October, November, December | Winter,  Spring,  Summer,  Autumn | NA | NA | NA | Callot et al. (1944) |
|  | Ain, Isère, Rhône, Savoie, Alpes-de-Haute-Provence | 1966-1976 | NA | NA | NA | NA | NA | NA | Gilot et al. (1976) |
|  | Gironde | 2010, 2012 | NA | March, May | Spring | NA | NA | NA | INPN (2023) |
|  | Ain, Alpes-de-Haute-Provence, Alpes-Maritimes, Bouches-du-Rhône, Charente-Maritime, Corse, Drôme, Haute-Garonne, Gironde, Indre-et-Loire, Isère, Loire-Atlantique, Manche, Meuse, Morbihan, Nord, Oise, Pyrénées-Atlantiques, Bas-Rhin, Haut-Rhin, Rhône, Seine-et-Marne, Var, Vienne, Yvelines, Territoire de Belfort, Paris | NA | NA | NA | NA | NA | NA | NA | Moussiegt (1986) |
|  | Paris, Hauts-de-Seine, Seine-Saint-Denis, Val-de-Marne, Yvelines, Essonne, Val-d'Oise, Seine-et-Marne, Oise, Nord, Meuse, Bas-Rhin, Haut-Rhin, Territoire-de-Belfort, Manche, Morbihan, Loire-Atlantique, Indre-et-Loire, Vienne, Charente-Maritime, Alpes-Maritimes, Haute-Garonne, Pyrénées-Atlantiques | NA | NA | NA | NA | NA | NA | NA | Rageau et al. (1970b) |
|  | Aveyron, Lot, Dordogne, Ariège, Haute-Garonne, Tarn, Tarn-et-Garonne, Lot-et-Garonne, Gers | NA | NA | March, April, May, June, July, August, September, October, November, December, January | Spring,  Summer,  Autumn,  Winter | NA | Larvae capture | NA | Ruffié (1957) |
|  | Val-de-Marne | 1902-1921 | NA | January, February, March, April, May, June, July, August, September, October, November, December | Winter,  Spring,  Summer,  Autumn | NA | NA | NA | Séguy (1923) |
|  | Landes, Gironde, Loir-et-Cher | 2010, 2012 | NA | March, May | Spring | NA | NA | NA | GBIF (2023) |
| *Culiseta*  *glaphyroptera* | Alpes-Maritimes, Bas-Rhin | NA | NA | NA | NA | NA | NA | NA | Moussiegt (1986) |
|  | Alpes-Maritimes, Bas-Rhin | NA | NA | NA | NA | NA | NA | NA | Rageau et al. (1970b) |
|  | Bas-Rhin | 1902-1921 | NA | January, February, March, April, May, June, July, August, September, October, November, December | Winter,  Spring,  Summer,  Autumn | NA | NA | NA | Séguy (1923) |
| *Culiseta*  *litorea* | Morbihan, Loire-Atlantique | 1993 | NA | NA | NA | NA | Larvae capture | NA | Brutus et al. (1993) |
|  | Alpes-Maritimes, Aude, Bouches-du-Rhône, Charente-Maritime, Corse, Gard, Haute-Garonne, Gironde, Hérault, Loire-Atlantique, Manche, Morbihan, Pyrénées-Orientales, Var, Vendée | NA | NA | NA | NA | NA | NA | NA | Moussiegt (1986) |
|  | Hérault, Gard, Bouches-du-Rhône | 1953-1966 | NA | January, February, March, April, May, June, July, August, September, October, November, December | Winter,  Spring,  Summer,  Autumn | NA | Larvae capture,  CDC trap | NA | Rageau et al. (1967) |
|  | Morbihan, Loire-Atlantique, Gironde, Vendée, Charente-Maritime, Pyrénées-Orientales, Aude, Hérault, Gard, Bouches-du-Rhône, Var, Alpes-Maritimes, Corse | NA | NA | NA | NA | NA | NA | NA | Rageau et al. (1970b) |
|  | Bouches-du-Rhône, Gard | NA | NA | NA | NA | NA | Larvae and  adult capture | NA | Rioux et al. (1955) |
|  | Gironde, Corrèze | NA | NA | NA | NA | NA | NA | NA | GBIF (2023) |
| *Culiseta*  *longiareolata* | Corse | 1944, 1946-1950, 1952 | NA | NA | NA | NA | NA | NA | Aitken (1954) |
|  | Hérault | 2004 | 6 | May, June, July, August, September, October | Spring,  Summer,  Autumn | 1, 25 | Hosted capture | NA | Balenghien et al. (2006) |
|  | Indre-et-Loire | 1936-1943 | NA | January, February, March, April, May, June, July, August, September, October, November, December | Winter,  Spring,  Summer,  Autumn | NA | NA | NA | Callot et al. (1944) |
|  | Ain, Isère, Rhône, Savoie | 1966-1976 | NA | NA | NA | NA | NA | NA | Gilot et al. (1976) |
|  | Alpes-Maritimes, Côte-d'Or, Calvados, Charente-Maritime, Hérault, Hauts-de-Seine, Loire, Moselle, Paris, Pyrénées-Orientales | 1952, 1955, 2006, 2011, 2014, 2018, 2020-2021 | NA | January, April, May, June, July, August, September, October, November, December | Winter,  Spring,  Summer,  Autumn | NA | NA | NA | INPN (2023) |
|  | Bouches-du-Rhône | 2006 | 11 | February, March, April, May, June, July, August, September, October, November, December | Winter,  Spring,  Summer,  Autumn | 0-50 | CDC trap  with CO2,  Ovitrap,  Human landing  capture | NA | l'Ambert et al. (2012) |
|  | Marne, Ardennes | 2017-2020 | NA | January, February, March, April, May, June, July, August, September, October, November, December | Winter,  Spring,  Summer,  Autumn | NA | CDC trap,  BG-Sentinel,  Larvae capture,  Aspirator | NA | Martinet (2021) |
|  | Ain, Alpes-de-Haute-Provence, Hautes-Alpes, Alpes-Maritimes, Ariège, Aude, Bouches-du-Rhône, Calvados, Charente-Maritime, Corse, Dordogne, Drôme, Gard, Haute-Garonne, Gironde, Hérault, Indre-et-Loire, Isère, Loire-Atlantique, Puy-de-Dôme, Pyrénées-Atlantiques, Hautes-Pyrénées, Pyrénées-Orientales, Bas-Rhin, Haut-Rhin, Rhône, Savoie, Seine-et-Marne, Tarn, Var, Vaucluse, Yvelines, Territoire de Belfort, Paris | NA | NA | NA | NA | NA | NA | NA | Moussiegt (1986) |
|  | Paris, Hauts-de-Seine, Seine-Saint-Denis, Val-de-Marne, Yvelines, Essonne, Val-d'Oise, Bas-Rhin, Haut-Rhin, Calvados, Loire-Atlantique, Indre-et-Loire, Charente-Maritime, Dordogne, Rhône, Isère, Drôme, Alpes-de-Haute-Provence, Hautes-Alpes, Alpes-Maritimes, Var, Bouches-du-Rhône, Gard, Haute-Garonne, Aude, Pyrénées-Orientales, Haute-Garonne, Tarn, Ariège, Hautes-Pyrénées, Pyrénées-Atlantiques | NA | NA | NA | NA | NA | NA | NA | Rageau et al. (1970b) |
|  | Aveyron, Lot, Dordogne, Ariège, Haute-Garonne, Tarn, Tarn-et-Garonne, Lot-et-Garonne, Gers | NA | NA | March, April, May, June, July, August, September, October, November, December, January | Spring,  Summer,  Autumn,  Winter | NA | Larvae capture | NA | Ruffié (1957) |
|  | Paris, Yvelines, Seine-et-Marne, Hautes-Pyrénées, Var, Hautes-Alpes, Alpes-Maritimes | 1902-1921 | NA | January, February, March, April, May, June, July, August, September, October, November, December | Winter,  Spring,  Summer,  Autumn | NA | NA | *Plasmodium danilewskyi* | Séguy (1923) |
|  | Bouches-du-Rhône, Gard | NA | NA | NA | NA | NA | Larvae and  adult capture | NA | Rioux et al. (1955) |
|  | Alpes-Maritimes, Calvados, Côte-d'Or, Gironde, Hautes-Alpes, Hérault, Loire, Moselle, Paris, Pyrénées-Orientales, Savoie, Vaucluse, Vendée | 1952, 1955, 2006-2007, 2011, 2014, 2018, 2020-2022 | NA | January, April, May, June, July, August, September, October, November, December | Winter,  Spring,  Summer,  Autumn | NA | NA | NA | GBIF (2023) |
| *Culiseta*  *morsitans* | Corse | 1944, 1946-1950, 1952 | NA | NA | NA | NA | NA | NA | Aitken (1954) |
|  | Indre-et-Loire | 1936-1943 | NA | January, February, March, April, May, June, July, August, September, October, November, December | Winter,  Spring,  Summer,  Autumn | NA | NA | NA | Callot et al. (1944) |
|  | Ain, Isère, Rhône, Savoie | 1966-1976 | NA | NA | NA | NA | NA | NA | Gilot et al. (1976) |
|  | Gironde, Moselle, Savoie | 1995, 2010, 2016 | NA | January, March, September | Winter,  Spring,  Summer | NA | NA | NA | INPN (2023) |
|  | Ardennes, Marne, Haute-Marne | 2017-2020 | NA | January, February, March, April, May, June, July, August, September, October, November, December | Winter,  Spring,  Summer,  Autumn | NA | CDC trap,  BG-Sentinel,  Larvae capture,  Aspirator | NA | Martinet (2021) |
|  | Ain, Bouches-du-Rhône, Calvados, Charente-Maritime Corse, Creuse, Haute-Garonne, Gironde, Ille-et-Vilaine, Indre-et-Loire, Isère, Loire, Haute-Loire, Loire-Atlantique, Maine-et-Loire, Meuse, Morbihan, Oise, Puy-de-Dôme, Pyrénées-Atlantiques, Bas-Rhin, Haut-Rhin, Rhône, Savoie, Seine-et-Marne, Vienne, Yvelines, Territoire de Belfort, Paris | NA | NA | NA | NA | NA | NA | NA | Moussiegt (1986) |
|  | Paris, Hauts-de-Seine, Seine-Saint-Denis, Val-de-Marne, Yvelines, Essonne, Val-d'Oise, Seine-et-Marne, Oise, Meuse, Bas-Rhin, Haut-Rhin, Calvados, Morbihan, Ille-et-Vilaine, Loire-Atlantique, Maine-et-Loire, Indre-et-Loire, Vienne, Creuse, Rhône, Ain, Isère, Haute-Loire, Haute-Garonne, Gironde, Pyrénées-Atlantiques, Charente-Maritime | NA | NA | NA | NA | NA | NA | NA | Rageau et al. (1970b) |
|  | Aveyron, Lot, Dordogne, Ariège, Haute-Garonne, Tarn, Tarn-et-Garonne, Lot-et-Garonne, Gers | NA | NA | March, April, May, June, July, August, September, October, November, December, January | Spring,  Summer,  Autumn,  Winter | NA | Larvae capture | NA | Ruffié (1957) |
|  | Yvelines, Hauts-de-Seine, Maine-et-Loire, Meuse, Creuse | 1902-1921 | NA | January, February, March, April, May, June, July, August, September, October, November, December | Winter,  Spring,  Summer,  Autumn | NA | NA | NA | Séguy (1923) |
|  | All France | NA | NA | NA | NA | NA | NA | NA | Séguy (1924) |
|  | Landes, Gironde, Savoie, Moselle, Loir-et-Cher | 1995, 2010, 2016 | NA | March, September | Spring,  Summer | NA | NA | NA | GBIF (2023) |
| *Culiseta*  *subochrea* | Morbihan, Loire-Atlantique | 1993 | NA | NA | NA | NA | Larvae capture | NA | Brutus et al. (1993) |
|  | Corse | 1944, 1946-1950, 1952 | NA | NA | NA | NA | NA | NA | Aitken (1954) |
|  | Bouches-du-Rhône | 2004 | 6 | May, June, July, August, September, October | Spring,  Summer,  Autumn | 1, 25 | Hosted capture | NA | Balenghien et al. (2006) |
|  | Ain, Isère, Rhône, Savoie | 1966-1976 | NA | NA | NA | NA | NA | NA | Gilot et al. (1976) |
|  | Bouches-du-Rhône | 2006 | 11 | February, March, April, May, June, July, August, September, October, November, December | Winter,  Spring,  Summer,  Autumn | 0-50 | CDC trap  with CO2,  Ovitrap,  Human landing  capture | NA | l'Ambert et al. (2012) |
|  | Bouches-du-Rhône | 1965-1967 | 15 | December, January, February, March, April | Winter | NA | NA | NA | Mouchet et al. (1969) |
|  | Bouches-du-Rhône | 1964-1968 | 10 | February, March, April, May, June, July, August, September, October, December | Winter,  Spring,  Summer,  Autumn | NA | NA | NA | Mouchet et al. (1970) |
|  | Ain, Ardèche, Aube, Aude, Bouches-du-Rhône, Charente-Maritime, Corse, Gard, Haute-Garonne, Gironde, Hérault, Indre-et-Loire, Loire-Atlantique, Meuse, Morbihan, Moselle, Puy-de-Dôme, Pyrénées-Orientales, Bas-Rhin, Rhône, Seine-Maritime, Var | NA | NA | NA | NA | NA | NA | NA | Moussiegt (1986) |
|  | Bouches-du-Rhône | 2005 | 8 | March, April, May, June, July, August, September, October | Spring,  Summer,  Autumn | NA | CDC trap | NA | Ponçon (2008) |
|  | Hérault, Gard, Bouches-du-Rhône | 1953-1966 | NA | January, February, March, April, May, June, July, August, September, October, November, December | Winter,  Spring,  Summer,  Autumn | NA | Larvae capture,  CDC trap | NA | Rageau et al. (1967) |
|  | Morbihan, Seine-Maritime, Aube, Meuse, Moselle, Bas-Rhin, Ain, Rhône, Puy-de-Dôme, Haute-Garonne, Aude, Hérault, Gard, Bouches-du-Rhône, Corse, Ardèche, Var | NA | NA | NA | NA | NA | NA | NA | Rageau et al. (1970b) |
|  | Hérault, Gard, Bouches-du-Rhône | 1965 | 1 | April | Spring | NA | Egg collection | NA | Rioux et al. (1967) |
|  | Aveyron, Lot, Dordogne, Ariège, Haute-Garonne, Tarn, Tarn-et-Garonne, Lot-et-Garonne, Gers | NA | NA | March, April, May, June, July, August, September, October, November, December, January | Spring,  Summer,  Autumn,  Winter | NA | Larvae capture | NA | Ruffié (1957) |
|  | Aube | 1902-1921 | NA | January, February, March, April, May, June, July, August, September, October, November, December | Winter,  Spring,  Summer,  Autumn | NA | NA | NA | Séguy (1923) |
|  | Bouches-du-Rhône, Gard | NA | NA | NA | NA | NA | Larvae and  adult capture | NA | Rioux et al. (1955) |
|  | Alpes-de-Haute-Provence, Gironde, Oise | NA | NA | NA | NA | NA | NA | NA | GBIF (2023) |
| *Orthopodomyia*  *pulcripalpis* | Indre-et-Loire | 1936-1943 | NA | January, February, March, April, May, June, July, August, September, October, November, December | Winter,  Spring,  Summer,  Autumn | NA | NA | NA | Callot et al. (1944) |
|  | Hérault | 1955 | 1 | June | Summer | 27 | NA | NA | Harant et al. (1955) |
|  | Pyrénées-Orientales | 1955 | NA | January | Winter | NA | NA | NA | INPN (2023) |
|  | Aude, Bouches-du-Rhône, Charente-Maritime, Corse, Côte-d'Or, Gard, Hérault, Indre-et-Loire, Oise, Pyrénées-Orientales, Seine-et-Marne, Var, Yvelines, Paris | NA | NA | NA | NA | NA | NA | NA | Moussiegt (1986) |
|  | Hérault, Gard, Bouches-du-Rhône | 1953-1966 | NA | January, February, March, April, May, June, July, August, September, October, November, December | Winter,  Spring,  Summer,  Autumn | NA | Larvae capture,  CDC trap | NA | Rageau et al. (1967) |
|  | Paris, Hauts-de-Seine, Seine-Saint-Denis, Val-de-Marne, Yvelines, Essonne, Val-d'Oise, Oise, Hérault, Pyrénées-Orientales, Bouches-du-Rhône, Aude, Gard, Corse | NA | NA | NA | NA | NA | NA | NA | Rageau et al. (1970b) |
|  | Hérault, Gard, Bouches-du-Rhône | 1965 | 1 | April | Spring | NA | Egg collection | NA | Rioux et al. (1967) |
|  | Paris, Moselle | 1902-1921 | NA | January, February, March, April, May, June, July, August, September, October, November, December | Winter,  Spring,  Summer,  Autumn | NA | NA | NA | Séguy (1923) |
|  | Bouches-du-Rhône, Gard | NA | NA | NA | NA | NA | Larvae and  adult capture | NA | Rioux et al. (1955) |
|  | Pyrénées-Orientales | 1955 | NA | January | Winter | NA | NA | NA | GBIF (2023) |
| *Uranotaenia*  *unguiculata* | Corse | 1944, 1946-1950, 1952 | NA | NA | NA | NA | NA | NA | Aitken (1954) |
|  | Bouches-du-Rhône | 1965-1967 | 15 | December, January, February, March, April | Winter | NA | NA | NA | Mouchet et al. (1969) |
|  | Bouches-du-Rhône | 1964-1968 | 10 | February, March, April, May, June, July, August, September, October, December | Winter,  Spring,  Summer,  Autumn | NA | NA | NA | Mouchet et al. (1970) |
|  | Aude, Bouches-du-Rhône, Corse, Gard, Hérault, Pyrénées-Orientales | NA | NA | NA | NA | NA | NA | NA | Moussiegt (1986) |
|  | Gard, Bouches-du-Rhône | 2005 | 8 | March, April, May, June, July, August, September, October | Spring,  Summer,  Autumn | NA | CDC trap | NA | Ponçon (2008) |
|  | Hérault, Gard, Bouches-du-Rhône | 1953-1966 | NA | January, February, March, April, May, June, July, August, September, October, November, December | Winter,  Spring,  Summer,  Autumn | NA | Larvae capture,  CDC trap | NA | Rageau et al. (1967) |
|  | Corse, Pyrénées-Orientales, Hérault, Gard, Bouches-du-Rhône | NA | NA | NA | NA | NA | NA | NA | Rageau et al. (1970b) |
|  | Bouches-du-Rhône, Gard | NA | NA | NA | NA | NA | Larvae and  adult capture | NA | Rioux et al. (1955) |
|  | Bouches-du-Rhône | NA | NA | NA | NA | NA | NA | NA | GBIF (2023) |

Supplementary Table S9. Captures of biting midges in cattle farms in France between 1953 and 2016 (NA: data not provided).

| Species | Capture | | | | | | | Pathogen | Reference |
| --- | --- | --- | --- | --- | --- | --- | --- | --- | --- |
|  | Department | Year | Study  duration  (month) | Month | Season | Altitude  (m) | Trapping  method |  |  |
| *Alluaudomyia*  *needhami* | Bas-Rhin | 1981-1982 | NA | NA | NA | NA | NA | NA | Arnold et al. (1982) |
| *Bezzia*  *flavicornis* | Bas-Rhin | 1981-1982 | NA | NA | NA | NA | NA | NA | Arnold et al. (1982) |
| *Bezzia*  *pygmaea* | Bas-Rhin | 1977 | NA | NA | NA | 142 | NA | Iridovirus | Rieb et al. (1982) |
|  | Pyrénées-Orientales | 2004 | NA | January | Winter | NA | NA | NA | INPN (2023) |
|  | Pyrénées-Orientales | 2004 | NA | January | Winter | NA | NA | NA | GBIF (2023) |
| *Culicoides*  *abchazicus* | Corse | 2012 | 12 | January, February, March, April, May, June, July, August, September, October, November, December | Winter,  Spring,  Summer,  Autumn | NA | OVI trap | NA | Balenghien et al. (2013) |
|  | Vaucluse | 2010 | 12 | January, February, March, April, May, June, July, August, September, October, November, December | Winter,  Spring,  Summer,  Autumn | NA | OVI trap | NA | Garros (2022) |
|  | Savoie | 2009 | 2 | April, June | Spring | NA | OVI trap | NA | Mathieu (2011) |
|  | Savoie | 2004-2006, 2008-2009 | NA | April, May, June, July, August, September | Spring,  Summer | NA | OVI trap | NA | Mathieu (2011) |
|  | Savoie | NA | NA | NA | NA | NA | OVI trap | NA | Mathieu et al. (2011) |
|  | Savoie | 2011 | NA | May | Spring | NA | NA | NA | GBIF (2023) |
| *Culicoides*  *accraensis* | Bas-Rhin, Haut-Rhin, Vosges | NA | NA | NA | NA | NA | NA | NA | Kremer (1965) |
| *Culicoides*  *alazanicus* | Indre-et-Loire | 2009 | 1 | July, August | Summer | 120 | Cattle bait trap,  OVI trap | NA | Viennet et al. (2013) |
|  | Bas-Rhin | NA | NA | NA | NA | NA | Larvae capture | NA | Chacker (1982) |
|  | Saône-et-Loire, Hérault, Hautes-Pyrénées | 2008-2012 | 3 | June, July, August | Summer | 215-1500 | OVI trap | NA | Rossi et al. (2019) |
|  | Corse | 2000 | 12 | October, November, December, January, February, March, April, May, June, July, August, September | Winter,  Spring,  Summer,  Autumn | NA | OVI trap | NA | Delécolle et al. (2002) |
|  | Ain, Aube, Aude, Charente, Charente-Maritime, Cher, Dordogne, Corse, Corse, Haute-Garonne, Hérault, Indre, Indre-et-Loire, Loir-et-Cher, Loire-Atlantique, Maine-et-Loire, Marne, Haute-Marne, Mayenne, Nièvre, Nord, Hautes-Pyrénées, Saône-et-Loire, Sarthe, Seine-et-Marne, Yvelines, Deux-Sèvres, Tarn-et-Garonne, Var, Vendée, Vosges, Yonne | 2010 | 12 | January, February, March, April, May, June, July, August, September, October, November, December | Winter,  Spring,  Summer,  Autumn | NA | OVI trap | NA | Garros (2022) |
|  | Bas-Rhin, Seine-et-Marne, Charente-Maritime | NA | NA | NA | NA | NA | NA | NA | Kremer (1965) |
|  | Bas-Rhin, Haut-Rhin | 1976 | 8 | March, April, May, June, July, August, September, October | Spring,  Summer,  Autumn | NA | Larvae capture | NA | Kremer et al. (1978) |
|  | Corse | 2009 | 2 | April, June | Spring | NA | OVI trap | NA | Mathieu (2011) |
|  | Corse | NA | NA | NA | NA | NA | OVI trap | NA | Mathieu et al. (2011) |
|  | Corse | 2002-2009 | 120 | January, February, March, April, May, June, July, August, September, October, November, December | Winter,  Spring,  Summer,  Autumn | NA | OVI trap | NA | Mehlhorn (2012) |
|  | Bas-Rhin | 1977-1978 | 17 | April, May, June, July, August, September, October, November, December, January, February, March | Spring,  Summer,  Autumn,  Winter | 150 | CDC trap,  Soil sampling | NA | Rieb (1987) |
|  | Corse | 2000 | 0.13 | October | Autumn | NA | OVI trap | NA | Zientara et al. (2000) |
| *Culicoides*  *albicans* | Gironde, Vosges | NA | NA | NA | NA | NA | NA | NA | Kremer (1965) |
| *Culicoides*  *albipennis* | Moselle | NA | NA | NA | NA | NA | NA | NA | Kieffer (1925) |
| *Culicoides*  *begueti* | Saône-et-Loire, Hautes-Alpes, Hautes-Pyrénées | 2008-2012 | 3 | June, July, August | Summer | 215-1500 | OVI trap | NA | Rossi et al. (2019) |
|  | Hautes-Alpes, Corse, Haute-Garonne, Var, Vendée | 2010 | 12 | January, February, March, April, May, June, July, August, September, October, November, December | Winter,  Spring,  Summer,  Autumn | NA | OVI trap | NA | Garros (2022) |
|  | Var | NA | NA | NA | NA | NA | NA | NA | Kremer (1965) |
|  | Var | 2009 | 2 | April, June | Spring | NA | OVI trap | NA | Mathieu (2011) |
|  | Var | NA | NA | NA | NA | NA | OVI trap | NA | Mathieu et al. (2011) |
|  | Corse | 2002-2009 | 120 | January, February, March, April, May, June, July, August, September, October, November, December | Winter,  Spring,  Summer,  Autumn | NA | OVI trap | NA | Mehlhorn (2012) |
| *Culicoides*  *brunnicans* | Indre-et-Loire | 2009 | 1 | July, August | Summer | 120 | Cattle bait trap,  OVI trap | NA | Viennet et al. (2013) |
|  | Ille-et-Vilaine, Cher | 2009 | 5 | February, March, April, May, June | Winter,  Spring | NA | OVI trap | NA | Garros et al. (2011) |
|  | Ardennes, Marne, Deux-Sèvres | 2008-2009 | NA | NA | NA | NA | UV CDC trap,  CDC trap | NA | Ninio et al. (2011a) |
|  | Indre-et-Loire | 2010 | 5 | April, May, June, September, October | Spring,  Summer,  Autumn | NA | OVI trap | NA | Viennet et al. (2012) |
|  | Bas-Rhin, Saône-et-Loire, Hautes-Alpes, Hérault, Hautes-Pyrénées | 2008-2012 | 3 | June, July, August | Summer | 215-1500 | OVI trap | NA | Rossi et al. (2019) |
|  | Ain, Allier, Alpes-de-Haute-Provence, Hautes-Alpes, Ardèche, Ardennes, Ariège, Aube, Aude, Aveyron, Calvados, Cantal, Charente, Charente-Maritime, Cher, Corrèze, Côte-d'Or, Côtes-d’Armor, Dordogne, Finistère, Haute-Garonne, Gironde, Ille-et-Vilaine, Indre, Indre-et-Loire, Isère, Jura, Loir-et-Cher, Loire, Loire-Atlantique, Loiret, Lot, Lozère, Maine-et-Loire, Manche, Marne, Haute-Marne, Mayenne, Meuse, Morbihan, Moselle, Nièvre, Orne, Pyrénées-Atlantiques, Bas-Rhin, Haute-Saône, Saône-et-Loire, Sarthe, Seine-et-Marne, Yvelines, Deux-Sèvres, Tarn, Tarn-et-Garonne, Var, Vaucluse, Vendée, Vienne, Haute-Vienne, Vosges, Yonne, Territoire-de-Belfort | 2010 | 12 | January, February, March, April, May, June, July, August, September, October, November, December | Winter,  Spring,  Summer,  Autumn | NA | OVI trap | NA | Garros (2022) |
|  | Manche, Gironde | NA | NA | NA | NA | NA | NA | NA | Kremer (1965) |
|  | Saône-et-Loire | 2009 | 2 | April, June | Spring | NA | OVI trap | NA | Mathieu (2011) |
|  | Saône-et-Loire | NA | NA | NA | NA | NA | OVI trap | NA | Mathieu et al. (2011) |
|  | Corse | 2002-2009 | 120 | January, February, March, April, May, June, July, August, September, October, November, December | Winter,  Spring,  Summer,  Autumn | NA | OVI trap | NA | Mehlhorn (2012) |
|  | Ardennes | 2009-2010 | 18 | January, February, March, April, May, June, July, August, September, October, November, December | Winter,  Spring,  Summer,  Autumn | 271, 243 | CDC trap | NA | Ninio (2011) |
|  | Ardennes | 2009-2010 | 26 | January, February, March, April, May, June, July, August, September, October, November, December | Winter,  Spring,  Summer,  Autumn | NA | UV CDC trap,  Soil sampling | NA | Ninio (2011) |
| *Culicoides*  *cameroni* | Hautes-Alpes | 2008-2012 | 3 | June, July, August | Summer | 215-1500 | OVI trap | NA | Rossi et al. (2019) |
|  | Hérault | 2010 | 12 | January, February, March, April, May, June, July, August, September, October, November, December | Winter,  Spring,  Summer,  Autumn | NA | OVI trap | NA | Garros (2022) |
|  | Hautes-Alpes | 2009 | 2 | April, June | Spring | NA | OVI trap | NA | Mathieu (2011) |
|  | Hautes-Alpes | NA | NA | NA | NA | NA | OVI trap | NA | Mathieu et al. (2011) |
|  | Corse | 2002-2009 | 120 | January, February, March, April, May, June, July, August, September, October, November, December | Winter,  Spring,  Summer,  Autumn | NA | OVI trap | NA | Mehlhorn (2012) |
| *Culicoides*  *cataneii /*  *gejgelensis* | Indre-et-Loire | 2009 | 1 | July, August | Summer | 120 | Cattle bait trap,  OVI trap | NA | Viennet et al. (2013) |
|  | Saône-et-Loire, Hautes-Alpes, Hérault, Hautes-Pyrénées | 2008-2012 | 3 | June, July, August | Summer | 215-1500 | OVI trap | NA | Rossi et al. (2019) |
|  | Alpes-Maritimes, Bouches-du-Rhône, Gard, Haute-Savoie, Hautes-Pyrénées, Hérault, Loire-Atlantique, Var, Vaucluse | 2004 | 8 | April, May, June, July, August, September, October, November | NA | NA | UV CDC trap | NA | Cetre-Sossah (2010) |
|  | Corse | 2000 | 12 | October, November, December, January, February, March, April, May, June, July, August, September | Winter,  Spring,  Summer,  Autumn | NA | OVI trap | NA | Delécolle et al. (2002) |
|  | Ain, Alpes-de-Haute-Provence, Hautes-Alpes, Ardèche, Ariège, Aude, Bouches-du-Rhône, Cantal, Charente-Maritime, Cher, Dordogne, Drôme, Eure, Corse, Corse, Gard, Haute-Garonne, Gers, Gironde, Hérault, Indre-et-Loire, Isère, Loir-et-Cher, Loire-Atlantique, Loiret, Lot, Maine-et-Loire, Marne, Meurthe-et-Moselle, Nièvre, Oise, Pyrénées-Atlantiques, Pyrénées-Orientales, Haute-Saône, Saône-et-Loire, Sarthe, Haute-Savoie, Seine-et-Marne, Yvelines, Tarn-et-Garonne, Var, Vaucluse, Vendée, Vienne, Yonne | 2010 | 12 | January, February, March, April, May, June, July, August, September, October, November, December | Winter,  Spring,  Summer,  Autumn | NA | OVI trap | NA | Garros (2022) |
|  | Corse | 2009 | 2 | April, June | Spring | NA | OVI trap | NA | Mathieu (2011) |
|  | Corse | NA | NA | NA | NA | NA | OVI trap | NA | Mathieu et al. (2011) |
|  | Corse | 2002-2009 | 120 | January, February, March, April, May, June, July, August, September, October, November, December | Winter,  Spring,  Summer,  Autumn | NA | OVI trap | NA | Mehlhorn (2012) |
|  | Alpes-Maritimes | 2002 | 8 | April, May, June, July, August, September, October, November | Spring,  Summer,  Autumn | NA | OVI trap | NA | Perrin et al. (2006) |
|  | Hérault, Gard, Bouches-du-Rhône | 1953-1966 | NA | January, February, March, April, May, June, July, August, September, October, November, December | Winter,  Spring,  Summer,  Autumn | NA | Manual capture,  Larvae capture,  CDC trap,  Malaise trap | NA | Rageau et al. (1967) |
|  | Corse | 2000 | 0.13 | October | Autumn | NA | OVI trap | NA | Zientara et al. (2000) |
|  | Marne | 2011 | NA | April | Spring | NA | NA | NA | GBIF (2023) |
| *Culicoides*  *caucoliberensis* | Corse | 2002-2009 | 120 | January, February, March, April, May, June, July, August, September, October, November, December | Winter,  Spring,  Summer,  Autumn | NA | OVI trap | NA | Mehlhorn (2012) |
| *Culicoides*  *chiopterus* | Indre-et-Loire | 2009 | 1 | July, August | Summer | 120 | Cattle bait trap,  OVI trap | NA | Viennet et al. (2013) |
|  | Gers, Ille-et-Vilaine, Cher | 2009 | 5 | February, March, April, May, June | Winter,  Spring | NA | OVI trap | NA | Garros et al. (2011) |
|  | Ardennes, Marne, Deux-Sèvres | 2008-2009 | NA | NA | NA | NA | UV CDC trap,  CDC trap | NA | Ninio et al. (2011a) |
|  | Ardennes | 2006 | 4 | September, October, November, December | Autumn | NA | OVI trap | NA | Baldet et al. (2008) |
|  | Vosges | 1981 | NA | NA | NA | NA | Larvae capture | NA | Waller et al. (1982) |
|  | Bas-Rhin, Saône-et-Loire, Hautes-Alpes, Hérault, Hautes-Pyrénées | 2008-2012 | 3 | June, July, August | Summer | 215-1500 | OVI trap | NA | Rossi et al. (2019) |
|  | Corse | 2009 | 12 | January, February, March, April, May, June, July, August, September, October, November, December | Winter,  Spring,  Summer,  Autumn | NA | OVI trap | NA | Balenghien et al. (2010) |
|  | Corse | 2012 | 12 | January, February, March, April, May, June, July, August, September, October, November, December | Winter,  Spring,  Summer,  Autumn | NA | OVI trap | NA | Balenghien et al. (2013) |
|  | Bas-Rhin | 2002-2005 | 5 | May, June, July, August, October | Summer,  Autumn | NA | UV CDC trap | NA | Cetre-Sossah (2010) |
|  | Corse | 2000 | 12 | October, November, December, January, February, March, April, May, June, July, August, September | Winter,  Spring,  Summer,  Autumn | NA | OVI trap | NA | Delécolle et al. (2002) |
|  | Ain, Aisne, Allier, Alpes-de-Haute-Provence, Hautes-Alpes, Alpes-Maritimes, Ardèche, Ardennes, Ariège, Aube, Aveyron, Calvados, Cantal, Charente, Charente-Maritime, Cher, Corrèze, Corse, Côte-d'Or, Côtes-d'Armor, Creuse, Dordogne, Doubs, Drôme, Eure, Eure-et-Loir, Finistère, Gard, Haute-Garonne, Gers, Gironde, Hérault, Ille-et-Vilaine, Indre, Indre-et-Loire, Isère, Jura, Landes, Loir-et-Cher, Loire, Haute-Loire, Loire-Atlantique, Loiret, Lot, Lot-et-Garonne, Lozère, Maine-et-Loire, Manche, Marne, Haute-Marne, Mayenne, Meurthe-et-Moselle, Meuse, Morbihan, Moselle, Nièvre, Nord, Oise, Orne, Pas-de-Calais, Puy-de-Dôme, Pyrénées-Atlantiques, Hautes-Pyrénées, Bas-Rhin, Haut-Rhin, Rhône, Haute-Saône, Saône-et-Loire, Sarthe, Savoie, Haute-Savoie, Seine-Maritime, Seine-et-Marne, Yvelines, Deux-Sèvres, Somme, Tarn, Tarn-et-Garonne, Var, Vaucluse, Vendée, Vienne, Haute-Vienne, Vosges, Yonne, Territoire-de-Belfort, Essonne, Val-d'Oise | NA | NA | NA | NA | NA | NA | NA | ECDC (2022a) |
|  | Ain, Aisne, Allier, Alpes-de-Haute-Provence, Hautes-Alpes, Alpes-Maritimes, Ardèche, Ardennes, Ariège, Aube, Aveyron, Calvados, Cantal, Charente, Charente-Maritime, Cher, Corrèze, Côte-d'Or, Côtes-d’Armor, Creuse, Dordogne, Doubs, Eure, Eure-et-Loir, Finistère, Gard, Gers, Gironde, Ille-et-Vilaine, Indre, Indre-et-Loire, Isère, Jura, Landes, Loir-et-Cher, Loire, Haute-Loire, Loire-Atlantique, Loiret, Lot, Maine-et-Loire, Manche, Marne, Haute-Marne, Mayenne, Meurthe-et-Moselle, Meuse, Morbihan, Moselle, Nièvre, Nord, Oise, Orne, Pas-de-Calais, Puy-de-Dôme, Pyrénées-Atlantiques, Bas-Rhin, Haut-Rhin, Rhône, Haute-Saône, Saône-et-Loire, Sarthe, Savoie, Haute-Savoie, Seine-Maritime, Seine-et-Marne, Yvelines, Deux-Sèvres, Somme, Var, Vendée, Vienne, Haute-Vienne, Vosges, Yonne, Territoire-de-Belfort, Essonne, Val-d’Oise | 2010 | 12 | January, February, March, April, May, June, July, August, September, October, November, December | Winter,  Spring,  Summer,  Autumn | NA | OVI trap | NA | Garros (2022) |
|  | Aisne, Ardennes | 2008-2011 | NA | NA | NA | 114-230 | UV CDC trap | NA | Hajd Henni et al. (2014) |
|  | Indre-et-Loire, Vosges, Bas-Rhin | NA | NA | NA | NA | NA | NA | NA | Kremer (1965) |
|  | Corse | 1964, 1970 | 2 | July | Summer | 900-1080 | CDC trap,  Soil sampling | NA | Kremer et al. (1971) |
|  | Haut-Rhin, Aube | 2009 | 2 | April, June | Spring | NA | OVI trap | NA | Mathieu (2011) |
|  | Ain, Yonne, Yvelines | 2004-2006, 2008-2009 | NA | April, May, June, July, August, September | Spring,  Summer | NA | OVI trap | NA | Mathieu (2011) |
|  | Haut-Rhin | NA | NA | NA | NA | NA | OVI trap | NA | Mathieu et al. (2011) |
|  | Corse | 2002-2009 | 120 | January, February, March, April, May, June, July, August, September, October, November, December | Winter,  Spring,  Summer,  Autumn | NA | OVI trap | Bluetongue  virus | Mehlhorn (2012) |
|  | Nord, Aisne, Ardennes, Meuse, Meurthe-et-Moselle, Moselle | 2006 | 5 | August, September, October, November, December | Summer,  Autumn | NA | OVI trap | NA | Meiswinkel et al. (2007) |
|  | Ardennes | 2009-2010 | 26 | January, February, March, April, May, June, July, August, September, October, November, December | Winter,  Spring,  Summer,  Autumn | NA | UV CDC trap,  Soil sampling | NA | Ninio (2011) |
|  | Ardennes | NA | NA | NA | NA | NA | NA | NA | Ninio (2011) |
|  | Corse | 1971 | NA | NA | NA | NA | NA | NA | Zientara et al. (2000) |
|  | Ardennes, Aube, Haut-Rhin, Orne, Yonne | 2009-2010 | NA | January, April, May, July | Winter,  Spring,  Summer | NA | NA | NA | GBIF (2023) |
| *Culicoides*  *clintoni* | Bas-Rhin | 2008-2012 | 3 | June, July, August | Summer | 215-1500 | OVI trap | NA | Rossi et al. (2019) |
| *Culicoides*  *comosioculatus* | Vosges, Bas-Rhin | NA | NA | NA | NA | NA | NA | NA | Kremer (1965) |
|  | Saône-et-Loire | 2009 | 2 | April, June | Spring | NA | OVI trap | NA | Mathieu (2011) |
|  | Saône-et-Loire | NA | NA | NA | NA | NA | OVI trap | NA | Mathieu et al. (2011) |
| *Culicoides*  *corsicus* | Corse | 2000 | 12 | October, November, December, January, February, March, April, May, June, July, August, September | Winter,  Spring,  Summer,  Autumn | NA | OVI trap | NA | Delécolle et al. (2002) |
|  | Corse | 1964, 1970 | 2 | July | Summer | 900-1080 | CDC trap,  Soil sampling | NA | Kremer et al. (1971) |
|  | Corse | 2002-2009 | 120 | January, February, March, April, May, June, July, August, September, October, November, December | Winter,  Spring,  Summer,  Autumn | NA | OVI trap | NA | Mehlhorn (2012) |
|  | Corse | 1971 | NA | NA | NA | NA | NA | NA | Zientara et al. (2000) |
| *Culicoides*  *deltus* | Corse | 2000 | 12 | October, November, December, January, February, March, April, May, June, July, August, September | Winter,  Spring,  Summer,  Autumn | NA | OVI trap | NA | Delécolle et al. (2002) |
|  | Alpes-de-Haute-Provence, Hautes-Alpes, Alpes-Maritimes, Ardèche, Ardennes, Ariège, Aveyron, Cantal, Corrèze, Creuse, Doubs, Drôme, Corse, Corse, Isère, Lozère, Nièvre, Oise, Puy-de-Dôme, Haut-Rhin, Rhône, Haute-Saône, Savoie, Haute-Savoie, Seine-et-Marne, Tarn, Var, Haute-Vienne, Vosges | 2010 | 12 | January, February, March, April, May, June, July, August, September, October, November, December | Winter,  Spring,  Summer,  Autumn | NA | OVI trap | NA | Garros (2022) |
|  | Corse | 2002-2009 | 120 | January, February, March, April, May, June, July, August, September, October, November, December | Winter,  Spring,  Summer,  Autumn | NA | OVI trap | NA | Mehlhorn (2012) |
|  | Nord, Aisne, Ardennes, Meuse, Meurthe-et-Moselle, Moselle | 2006 | 5 | August, September, October, November, December | Summer,  Autumn | NA | OVI trap | NA | Meiswinkel et al. (2007) |
|  | Ardennes | 2009-2010 | 26 | January, February, March, April, May, June, July, August, September, October, November, December | Winter,  Spring,  Summer,  Autumn | NA | UV CDC trap,  Soil sampling | NA | Ninio (2011) |
|  | Corse | 2000 | 0.13 | October | Autumn | NA | OVI trap | NA | Zientara et al. (2000) |
| *Culicoides*  *derisor* | Corse | 2000 | 12 | October, November, December, January, February, March, April, May, June, July, August, September | Winter,  Spring,  Summer,  Autumn | NA | OVI trap | NA | Delécolle et al. (2002) |
|  | Var | NA | NA | NA | NA | NA | NA | NA | Kremer (1965) |
|  | Var | 2009 | 2 | April, June | Spring | NA | OVI trap | NA | Mathieu (2011) |
|  | Var | NA | NA | NA | NA | NA | OVI trap | NA | Mathieu et al. (2011) |
|  | Corse | 2002-2009 | 120 | January, February, March, April, May, June, July, August, September, October, November, December | Winter,  Spring,  Summer,  Autumn | NA | OVI trap | NA | Mehlhorn (2012) |
|  | Var | 2002 | 8 | April, May, June, July, August, September, October, November | Spring,  Summer,  Autumn | NA | OVI trap | NA | Perrin et al. (2006) |
| *Culicoides*  *dewulfi* | Indre-et-Loire | 2009 | 1 | July, August | Summer | 120 | Cattle bait trap,  OVI trap | NA | Viennet et al. (2013) |
|  | Gers, Ille-et-Vilaine, Cher | 2009 | 5 | February, March, April, May, June | Winter,  Spring | NA | OVI trap | NA | Garros et al. (2011) |
|  | Ardennes, Marne, Deux-Sèvres | 2008-2009 | NA | NA | NA | NA | UV CDC trap,  CDC trap | NA | Ninio et al. (2011a) |
|  | Ardennes | 2006 | 4 | September, October, November, December | Autumn | NA | OVI trap | NA | Baldet et al. (2008) |
|  | Bas-Rhin, Saône-et-Loire, Hautes-Alpes, Hérault, Hautes-Pyrénées | 2008-2012 | 3 | June, July, August | Summer | 215-1500 | OVI trap | NA | Rossi et al. (2019) |
|  | Aisne, Gard | 2008 | 3 | July, August, September | Summer | 110, 200 | UV CDC trap,  CDC trap | NA | Augot et al. (2010) |
|  | Corse | 2009 | 12 | January, February, March, April, May, June, July, August, September, October, November, December | Winter,  Spring,  Summer,  Autumn | NA | OVI trap | NA | Balenghien et al. (2010) |
|  | Corse | 2012 | 12 | January, February, March, April, May, June, July, August, September, October, November, December | Winter,  Spring,  Summer,  Autumn | NA | OVI trap | NA | Balenghien et al. (2013) |
|  | Vaucluse | 2002-2005 | 5 | May, June, July, August, October | Summer,  Autumn | NA | UV CDC trap | NA | Cetre-Sossah (2010) |
|  | Ain, Aisne, Allier, Alpes-de-Haute-Provence, Hautes-Alpes, Alpes-Maritimes, Ardèche, Ardennes, Ariège, Aube, Aveyron, Calvados, Cantal, Charente, Charente-Maritime, Cher, Corrèze, Corse, Côte-d'Or, Côtes-d'Armor, Creuse, Dordogne, Doubs, Drôme, Eure, Eure-et-Loir, Finistère, Haute-Garonne, Gers, Gironde, Hérault, Ille-et-Vilaine, Indre, Indre-et-Loire, Isère, Jura, Landes, Loir-et-Cher, Loire, Haute-Loire, Loire-Atlantique, Loiret, Lot, Lot-et-Garonne, Lozère, Maine-et-Loire, Manche, Marne, Haute-Marne, Mayenne, Meurthe-et-Moselle, Meuse, Morbihan, Moselle, Nièvre, Nord, Oise, Orne, Pas-de-Calais, Puy-de-Dôme, Pyrénées-Atlantiques, Hautes-Pyrénées, Bas-Rhin, Haut-Rhin, Rhône, Haute-Saône, Saône-et-Loire, Sarthe, Savoie, Haute-Savoie, Seine-Maritime, Seine-et-Marne, Yvelines, Deux-Sèvres, Somme, Tarn, Tarn-et-Garonne, Var, Vaucluse, Vendée, Vienne, Haute-Vienne, Vosges, Yonne, Territoire-de-Belfort, Essonne, Val-d'Oise | NA | NA | NA | NA | NA | NA | NA | ECDC (2022a) |
|  | Ain, Aisne, Allier, Alpes-de-Haute-Provence, Hautes-Alpes, Ardennes, Ariège, Aube, Aveyron, Calvados, Cantal, Charente, Charente-Maritime, Cher, Corrèze, Côte-d'Or, Côtes-d’Armor, Dordogne, Doubs, Eure, Eure-et-Loir, Finistère, Corse, Haute-Garonne, Gers, Gironde, Ille-et-Vilaine, Indre, Indre-et-Loire, Isère, Jura, Landes, Loir-et-Cher, Loire, Haute-Loire, Loire-Atlantique, Loiret, Lot, Lozère, Maine-et-Loire, Manche, Marne, Haute-Marne, Mayenne, Meuse, Morbihan, Moselle, Nièvre, Nord, Oise, Orne, Pas-de-Calais, Pyrénées-Atlantiques, Hautes-Pyrénées, Bas-Rhin, Haut-Rhin, Rhône, Haute-Saône, Saône-et-Loire, Sarthe, Savoie, Haute-Savoie, Seine-Maritime, Seine-et-Marne, Yvelines, Deux-Sèvres, Somme, Tarn, Tarn-et-Garonne, Vaucluse, Vendée, Vienne, Vosges, Yonne, Territoire-de-Belfort, Val-d’Oise | 2010 | 12 | January, February, March, April, May, June, July, August, September, October, November, December | Winter,  Spring,  Summer,  Autumn | NA | OVI trap | NA | Garros (2022) |
|  | Aisne, Ardennes | 2008-2011 | NA | NA | NA | 114-230 | UV CDC trap | NA | Hajd Henni et al. (2014) |
|  | Calvados, Landes | 2010 | 0.13 | April, July, November | Spring,  Summer,  Autumn | NA | OVI trap | NA | Kluiters et al. (2016) |
|  | Bas-Rhin, Vosges | NA | NA | NA | NA | NA | NA | NA | Kremer (1965) |
|  | Meuse, Ardennes | 2009 | 2 | April, June | Spring | NA | OVI trap | NA | Mathieu (2011) |
|  | Aisne, Haute-Saône, Ardennes | 2004-2006, 2008-2009 | NA | April, May, June, July, August, September | Spring,  Summer | NA | OVI trap | NA | Mathieu (2011) |
|  | Meuse | NA | NA | NA | NA | NA | OVI trap | NA | Mathieu et al. (2011) |
|  | Corse | 2002-2009 | 120 | January, February, March, April, May, June, July, August, September, October, November, December | Winter,  Spring,  Summer,  Autumn | NA | OVI trap | NA | Mehlhorn (2012) |
|  | Nord, Aisne, Ardennes, Meuse, Meurthe-et-Moselle, Moselle | 2006 | 5 | August, September, October, November, December | Summer,  Autumn | NA | OVI trap | NA | Meiswinkel et al. (2007) |
|  | Ardennes | 2009-2010 | 18 | January, February, March, April, May, June, July, August, September, October, November, December | Winter,  Spring,  Summer,  Autumn | 271, 243 | CDC trap | NA | Ninio (2011) |
|  | Ardennes | 2009-2010 | 26 | January, February, March, April, May, June, July, August, September, October, November, December | Winter,  Spring,  Summer,  Autumn | NA | UV CDC trap,  Soil sampling | NA | Ninio (2011) |
|  | Ardennes | NA | NA | NA | NA | NA | NA | NA | Ninio (2011) |
|  | Aisne, Ardennes, Doubs, Haute-Saône, Yonne | 2009-2011 | NA | January, April, July | Winter,  Spring,  Summer | NA | NA | NA | GBIF (2023) |
| *Culicoides*  *duddingstoni* | Indre-et-Loire | 2009 | 1 | July, August | Summer | 120 | Cattle bait trap,  OVI trap | NA | Viennet et al. (2013) |
|  | Ardennes | 2006 | 4 | September, October, November, December | Autumn | NA | OVI trap | NA | Baldet et al. (2008) |
|  | Bas-Rhin, Hautes-Pyrénées | 2008-2012 | 3 | June, July, August | Summer | 215-1500 | OVI trap | NA | Rossi et al. (2019) |
|  | Alpes-de-Haute-Provence, Ardèche, Somme, Var, Vendée | 2010 | 12 | January, February, March, April, May, June, July, August, September, October, November, December | Winter,  Spring,  Summer,  Autumn | NA | OVI trap | NA | Garros (2022) |
|  | Moselle | NA | NA | NA | NA | NA | NA | NA | Kremer (1965) |
|  | Corse | 2002-2009 | 120 | January, February, March, April, May, June, July, August, September, October, November, December | Winter,  Spring,  Summer,  Autumn | NA | OVI trap | NA | Mehlhorn (2012) |
|  | Nord, Aisne, Ardennes, Meuse, Meurthe-et-Moselle, Moselle | 2006 | 5 | August, September, October, November, December | Summer,  Autumn | NA | OVI trap | NA | Meiswinkel et al. (2007) |
|  | Hérault, Gard, Bouches-du-Rhône | 1953-1966 | NA | January, February, March, April, May, June, July, August, September, October, November, December | Winter,  Spring,  Summer,  Autumn | NA | Manual capture,  Larvae capture,  CDC trap,  Malaise trap | NA | Rageau et al. (1967) |
| *Culicoides*  *dzhafarovi* | Corse | 2002-2009 | 120 | January, February, March, April, May, June, July, August, September, October, November, December | Winter,  Spring,  Summer,  Autumn | NA | OVI trap | NA | Mehlhorn (2012) |
| *Culicoides*  *furcillatus* | Ardennes, Marne, Deux-Sèvres | 2008-2009 | NA | NA | NA | NA | UV CDC trap,  CDC trap | NA | Ninio et al. (2011a) |
|  | Bas-Rhin, Saône-et-Loire, Hautes-Alpes, Hérault, Hautes-Pyrénées | 2008-2012 | 3 | June, July, August | Summer | 215-1500 | OVI trap | NA | Rossi et al. (2019) |
|  | Corse | 2000 | 12 | October, November, December, January, February, March, April, May, June, July, August, September | Winter,  Spring,  Summer,  Autumn | NA | OVI trap | NA | Delécolle et al. (2002) |
|  | Alpes-de-Haute-Provence, Hautes-Alpes, Alpes-Maritimes, Ardèche, Ariège, Aveyron, Calvados, Cantal, Corrèze, Côte-d'Or, Côtes-d’Armor, Creuse, Dordogne, Corse, Corse, Gard, Gers, Ille-et-Vilaine, Isère, Jura, Haute-Loire, Lozère, Marne, Haute-Marne, Meuse, Moselle, Nièvre, Oise, Pyrénées-Atlantiques, Haut-Rhin, Haute-Saône, Saône-et-Loire, Savoie, Haute-Savoie, Seine-et-Marne, Yvelines, Tarn, Vaucluse, Vienne, Haute-Vienne, Vosges, Yonne, Val-d’Oise | 2010 | 12 | January, February, March, April, May, June, July, August, September, October, November, December | Winter,  Spring,  Summer,  Autumn | NA | OVI trap | NA | Garros (2022) |
|  | Alpes-Maritimes | 2009 | 2 | April, June | Spring | NA | OVI trap | NA | Mathieu (2011) |
|  | Alpes-Maritimes | NA | NA | NA | NA | NA | OVI trap | NA | Mathieu et al. (2011) |
|  | Corse | 2002-2009 | 120 | January, February, March, April, May, June, July, August, September, October, November, December | Winter,  Spring,  Summer,  Autumn | NA | OVI trap | NA | Mehlhorn (2012) |
|  | Ardennes | 2009-2010 | 26 | January, February, March, April, May, June, July, August, September, October, November, December | Winter,  Spring,  Summer,  Autumn | NA | UV CDC trap,  Soil sampling | NA | Ninio (2011) |
|  | Aube | 2012 | NA | June | Summer | NA | NA | NA | GBIF (2023) |
| *Culicoides*  *gornostaevae* | Hautes-Pyrénées | 2008-2012 | 3 | June, July, August | Summer | 215-1500 | OVI trap | NA | Rossi et al. (2019) |
| *Culicoides*  *griseidorsum* | Alpes-Maritimes, Var, Bouches-du-Rhône | 2002 | 8 | April, May, June, July, August, September, October, November | Spring,  Summer,  Autumn | NA | UV CDC trap | NA | Cêtre-Sossah et al. (2004) |
|  | Corse, Pyrénées-Orientales, Aude, Hérault, Gard, Bouches-du-Rhône, Var, Alpes-Maritimes | 2002-2003 | 38 | February, March, April, May, June, July, August, September, October, November, December | Winter,  Spring,  Summer,  Autumn | NA | UV CDC trap | NA | Baldet et al. (2005) |
|  | Corse, Alpes-Maritimes, Var, Bouches-du-Rhône, Gard, Hérault, Aude, Pyrénées-Orientales | 2002 | 11 | February, March, April, May, June, July, August, September, October, November, December | Winter,  Spring,  Summer,  Autumn | NA | OVI trap | NA | Baldet et al. (2004) |
|  | Alpes-Maritimes, Bouches-du-Rhône, Hérault, Loire-Atlantique, Pyrénées-Orientales | 2004 | 8 | April, May, June, July, August, September, October, November | NA | NA | UV CDC trap | NA | Cetre-Sossah (2010) |
|  | Corse | 2000 | 12 | October, November, December, January, February, March, April, May, June, July, August, September | Winter,  Spring,  Summer,  Autumn | NA | OVI trap | NA | Delécolle et al. (2002) |
|  | Charente-Maritime, Corse, Corse, Vendée, Vienne | 2010 | 12 | January, February, March, April, May, June, July, August, September, October, November, December | Winter,  Spring,  Summer,  Autumn | NA | OVI trap | NA | Garros (2022) |
|  | Corse | 2009 | 2 | April, June | Spring | NA | OVI trap | NA | Mathieu (2011) |
|  | Corse | NA | NA | NA | NA | NA | OVI trap | NA | Mathieu et al. (2011) |
|  | Corse | 2002-2009 | 120 | January, February, March, April, May, June, July, August, September, October, November, December | Winter,  Spring,  Summer,  Autumn | NA | OVI trap | NA | Mehlhorn (2012) |
|  | Var | 2002 | 8 | April, May, June, July, August, September, October, November | Spring,  Summer,  Autumn | NA | OVI trap | NA | Perrin et al. (2006) |
|  | Corse | 2010 | NA | May | Spring | NA | NA | NA | GBIF (2023) |
| *Culicoides*  *grisescens* | Vosges | 1981 | NA | NA | NA | NA | Larvae capture | NA | Waller et al. (1982) |
|  | Hautes-Pyrénées | 2008-2012 | 3 | June, July, August | Summer | 215-1500 | OVI trap | NA | Rossi et al. (2019) |
|  | Ariège, Ille-et-Vilaine, Manche, Savoie | 2010 | 12 | January, February, March, April, May, June, July, August, September, October, November, December | Winter,  Spring,  Summer,  Autumn | NA | OVI trap | NA | Garros (2022) |
|  | Bas-Rhin, Haut-Rhin, Puy-de-Dôme, Pyrénées-Orientales | NA | NA | NA | NA | NA | NA | NA | Kremer (1965) |
|  | Corse | 2002-2009 | 120 | January, February, March, April, May, June, July, August, September, October, November, December | Winter,  Spring,  Summer,  Autumn | NA | OVI trap | NA | Mehlhorn (2012) |
|  | Ardennes | 2009-2010 | 26 | January, February, March, April, May, June, July, August, September, October, November, December | Winter,  Spring,  Summer,  Autumn | NA | UV CDC trap,  Soil sampling | NA | Ninio (2011) |
| *Culicoides*  *haranti* | Corse, Var | 2010 | 12 | January, February, March, April, May, June, July, August, September, October, November, December | Winter,  Spring,  Summer,  Autumn | NA | OVI trap | NA | Garros (2022) |
|  | Hérault | NA | NA | NA | NA | NA | NA | NA | Kremer (1965) |
|  | Corse | 2002-2009 | 120 | January, February, March, April, May, June, July, August, September, October, November, December | Winter,  Spring,  Summer,  Autumn | NA | OVI trap | NA | Mehlhorn (2012) |
|  | Hérault | 1957 | NA | February | Winter | 30 | Larvae capture | NA | Rioux et al. (1959) |
| *Culicoides*  *heliophilus* | Alpes-de-Haute-Provence, Ariège | 2010 | 12 | January, February, March, April, May, June, July, August, September, October, November, December | Winter,  Spring,  Summer,  Autumn | NA | OVI trap | NA | Garros (2022) |
|  | Savoie, Vosges | NA | NA | NA | NA | NA | NA | NA | Kremer (1965) |
|  | Nièvre | 2009 | 2 | April, June | Spring | NA | OVI trap | NA | Mathieu (2011) |
|  | Nièvre | NA | NA | NA | NA | NA | OVI trap | NA | Mathieu et al. (2011) |
| *Culicoides*  *heteroclitus* | Var, Vaucluse | 2004 | 8 | April, May, June, July, August, September, October, November | NA | NA | UV CDC trap | NA | Cetre-Sossah (2010) |
|  | Ardèche, Var | 2010 | 12 | January, February, March, April, May, June, July, August, September, October, November, December | Winter,  Spring,  Summer,  Autumn | NA | OVI trap | NA | Garros (2022) |
|  | Var | 2009 | 2 | April, June | Spring | NA | OVI trap | NA | Mathieu (2011) |
|  | Var | NA | NA | NA | NA | NA | OVI trap | NA | Mathieu et al. (2011) |
|  | Var | 2002 | 8 | April, May, June, July, August, September, October, November | Spring,  Summer,  Autumn | NA | OVI trap | NA | Perrin et al. (2006) |
| *Culicoides*  *ibericus* | Corse | 2012 | 12 | January, February, March, April, May, June, July, August, September, October, November, December | Winter,  Spring,  Summer,  Autumn | NA | OVI trap | NA | Balenghien et al. (2013) |
| *Culicoides*  *imicola* | Var, Corse | 2010-2012 | 28 | September, October, November, December, January, February, March, April, May, June, July, August | Autumn,  Winter,  Spring,  Summer | NA | OVI trap | Bluetongue  virus | Jacquet et al. (2016a) |
|  | Var | 2005 | NA | May | Spring | NA | NA | NA | Sailleau et al. (2005) |
|  | Corse | 2002-2003 | 38 | February, March, April, May, June, July, August, September, October, November, December | Winter,  Spring,  Summer,  Autumn | NA | UV CDC trap | NA | Baldet et al. (2005) |
|  | Var, Alpes-Maritimes, Pyrénées-Orientales, Corse | 2005 | NA | NA | NA | NA | NA | Bluetongue  virus | Gerbier et al. (2006) |
|  | Corse, Var | 2000, 2004 | NA | NA | NA | NA | NA | Bluetongue  virus | Tran et al. (2005) |
|  | Corse | 2000 | 1 | September | Summer | NA | NA | Bluetongue  virus | Hendrikx (2003) |
|  | Corse | 2000 | NA | NA | NA | NA | NA | Bluetongue  virus | OIE (2000) |
|  | Corse | 2002-2003 | 24 | January, February, March, April, May, June, July, August, September, October, November, December | Winter,  Spring,  Summer,  Autumn | NA | CDC trap | Bluetongue  virus | Gerbier et al. (2008) |
|  | Corse, Pyrénées-Orientales, Var | 2008, 2012 | NA | NA | NA | NA | OVI trap | NA | Jacquet et al. (2015) |
|  | Corse | 2002 | 11 | February, March, April, May, June, July, August, September, October, November, December | Winter,  Spring,  Summer,  Autumn | NA | OVI trap | NA | Baldet et al. (2004) |
|  | Corse | 2009 | 12 | January, February, March, April, May, June, July, August, September, October, November, December | Winter,  Spring,  Summer,  Autumn | NA | OVI trap | NA | Balenghien et al. (2010) |
|  | Corse | 2013 | 12 | January, February, March, April, May, June, July, August, September, October, November, December | Winter,  Spring,  Autumn,  Summer | NA | OVI trap | NA | Balenghien et al. (2014a) |
|  | Corse | 2014 | 12 | January, February, March, April, May, June, July, August, September, October, November, December | Winter,  Spring,  Summer,  Autumn | NA | OVI trap | NA | Balenghien et al. (2014b) |
|  | Corse, Var | 2004 | 8 | April, May, June, July, August, September, October, November | NA | NA | UV CDC trap | Bluetongue  virus | Cetre-Sossah (2010) |
|  | Pyrénées-Orientales, Var, Corse | 2007-2013 | 12 | January, February, March, April, May, June, July, August, September, October, November, December | NA | NA | OVI trap | NA | Cuéllar et al. (2018) |
|  | Corse | 2000 | 12 | October, November, December, January, February, March, April, May, June, July, August, September | Winter,  Spring,  Summer,  Autumn | NA | OVI trap | NA | Delécolle et al. (2002) |
|  | Corse, Alpes-Maritimes, Var | NA | NA | NA | NA | NA | NA | NA | ECDC (2022a) |
|  | Corse, Corse, Var | 2010 | 12 | January, February, March, April, May, June, July, August, September, October, November, December | Winter,  Spring,  Summer,  Autumn | NA | OVI trap | NA | Garros (2022) |
|  | Corse, Var | 2000, 2004 | NA | NA | NA | NA | NA | Bluetongue  virus | Gauchard et al. (2005) |
|  | Pyrénées-Orientales, Var, Corse | 2008-2012 | 2 | September, October | Autumn | NA | OVI trap | NA | Jacquet et al. (2016b) |
|  | Corse | 2009 | 2 | April, June | Spring | NA | OVI trap | NA | Mathieu (2011) |
|  | Corse, Var | 2004-2006, 2008-2009 | NA | April, May, June, July, August, September | Spring,  Summer | NA | OVI trap | NA | Mathieu (2011) |
|  | Corse, Var | 2004 | NA | NA | NA | NA | NA | Bluetongue  virus | Mathieu (2011) |
|  | Corse | NA | NA | NA | NA | NA | OVI trap | NA | Mathieu et al. (2011) |
|  | Corse, Var, Alpes-Maritimes | 2002-2009 | 120 | January, February, March, April, May, June, July, August, September, October, November, December | Winter,  Spring,  Summer,  Autumn | NA | OVI trap | Bluetongue  virus | Mehlhorn (2012) |
|  | Corse | 2002 | NA | NA | NA | NA | NA | Bluetongue  virus | Mellor (2004) |
|  | Corse | 2000 | NA | NA | NA | NA | NA | Bluetongue  virus | Mellor et al. (2002) |
|  | Corse | 2016 | NA | NA | NA | NA | NA | NA | Mignotte (2020) |
|  | Corse | NA | NA | NA | NA | NA | NA | NA | Ninio (2011) |
|  | Corse | 2002 | 8 | April, May, June, July, August, September, October, November | Spring,  Summer,  Autumn | NA | OVI trap | NA | Perrin et al. (2006) |
|  | Corse | 2012-2013 | 4 | June, August, September | Spring,  Summer | NA | OVI trap | NA | Venail (2014) |
|  | Corse | 2012-2013 | 6 | May, June, August, September | Spring,  Summer | NA | OVI trap | NA | Venail (2014) |
|  | Corse, Var | 2002, 2004 | NA | NA | NA | NA | NA | Bluetongue  virus | Viennet (2011) |
|  | Corse | 2000 | 0.13 | October | Autumn | NA | OVI trap | Bluetongue  virus | Zientara et al. (2000) |
|  | Corse | 2000-2001 | 4 | November, December, January, June | Winter | NA | OVI trap | Bluetongue  virus | Zientara et al. (2001) |
|  | Corse, Pyrénées-Orientales, Var | 2005-2006, 2008, 2012 | NA | January, March, June, September | Winter,  Spring,  Summer | NA | NA | NA | GBIF (2023) |
| *Culicoides*  *impunctatus* | Vosges | 1981 | NA | NA | NA | NA | Larvae capture | NA | Waller et al. (1982) |
|  | Bas-Rhin, Hérault | 2008-2012 | 3 | June, July, August | Summer | 215-1500 | OVI trap | NA | Rossi et al. (2019) |
|  | Ardennes, Haute-Garonne, Nièvre, Pyrénées-Atlantiques, Saône-et-Loire, Yvelines, Vosges | 2010 | 12 | January, February, March, April, May, June, July, August, September, October, November, December | Winter,  Spring,  Summer,  Autumn | NA | OVI trap | NA | Garros (2022) |
|  | Vosges, Pyrénées-Orientales | NA | NA | NA | NA | NA | NA | NA | Kremer (1965) |
|  | Nièvre | 2009 | 2 | April, June | Spring | NA | OVI trap | NA | Mathieu (2011) |
|  | Nièvre | NA | NA | NA | NA | NA | OVI trap | NA | Mathieu et al. (2011) |
|  | Ardennes | 2009-2010 | 26 | January, February, March, April, May, June, July, August, September, October, November, December | Winter,  Spring,  Summer,  Autumn | NA | UV CDC trap,  Soil sampling | NA | Ninio (2011) |
| *Culicoides*  *indistinctus* | Cantal | 2010 | 12 | January, February, March, April, May, June, July, August, September, October, November, December | Winter,  Spring,  Summer,  Autumn | NA | OVI trap | NA | Garros (2022) |
|  | Alpes-Maritimes | 2009 | 2 | April, June | Spring | NA | OVI trap | NA | Mathieu (2011) |
|  | Alpes-Maritimes | NA | NA | NA | NA | NA | OVI trap | NA | Mathieu et al. (2011) |
|  | Corse | 2002-2009 | 120 | January, February, March, April, May, June, July, August, September, October, November, December | Winter,  Spring,  Summer,  Autumn | NA | OVI trap | NA | Mehlhorn (2012) |
|  | Alpes-Maritimes | 2002 | 8 | April, May, June, July, August, September, October, November | Spring,  Summer,  Autumn | NA | OVI trap | NA | Perrin et al. (2006) |
| *Culicoides*  *jumineri* | Hautes-Alpes, Hautes-Pyrénées | 2008-2012 | 3 | June, July, August | Summer | 215-1500 | OVI trap | NA | Rossi et al. (2019) |
|  | Corse | 2000 | 12 | October, November, December, January, February, March, April, May, June, July, August, September | Winter,  Spring,  Summer,  Autumn | NA | OVI trap | NA | Delécolle et al. (2002) |
|  | Ardèche, Corse, Corse | 2010 | 12 | January, February, March, April, May, June, July, August, September, October, November, December | Winter,  Spring,  Summer,  Autumn | NA | OVI trap | NA | Garros (2022) |
|  | Bouches-du-Rhône | 2009 | 2 | April, June | Spring | NA | OVI trap | NA | Mathieu (2011) |
|  | Bouches-du-Rhône | NA | NA | NA | NA | NA | OVI trap | NA | Mathieu et al. (2011) |
|  | Corse | 2002-2009 | 120 | January, February, March, April, May, June, July, August, September, October, November, December | Winter,  Spring,  Summer,  Autumn | NA | OVI trap | NA | Mehlhorn (2012) |
| *Culicoides*  *jumineri /*  *kurensis* | Alpes-Maritimes, Bouches-du-Rhône, Hautes-Pyrénées, Hérault, Loire-Atlantique | 2004 | 8 | April, May, June, July, August, September, October, November | NA | NA | UV CDC trap | NA | Cetre-Sossah (2010) |
| *Culicoides*  *jurensis* | Doubs, Haut-Rhin | NA | NA | NA | NA | NA | NA | NA | Kremer (1965) |
| *Culicoides*  *kibunensis* | Indre-et-Loire | 2009 | 1 | July, August | Summer | 120 | Cattle bait trap,  OVI trap | NA | Viennet et al. (2013) |
|  | Indre-et-Loire | 2010 | 5 | April, May, June, September, October | Spring,  Summer,  Autumn | NA | OVI trap | NA | Viennet et al. (2012) |
|  | Bas-Rhin | 1977 | NA | NA | NA | 142 | NA | Iridovirus | Rieb et al. (1982) |
|  | Vosges | 1981 | NA | NA | NA | NA | Larvae capture | NA | Waller et al. (1982) |
|  | Bas-Rhin, Saône-et-Loire, Hautes-Alpes, Hérault, Hautes-Pyrénées | 2008-2012 | 3 | June, July, August | Summer | 215-1500 | OVI trap | NA | Rossi et al. (2019) |
|  | Alpes-Maritimes, Vaucluse | 2004 | 8 | April, May, June, July, August, September, October, November | NA | NA | UV CDC trap | NA | Cetre-Sossah (2010) |
|  | Corse | 2000 | 12 | October, November, December, January, February, March, April, May, June, July, August, September | Winter,  Spring,  Summer,  Autumn | NA | OVI trap | NA | Delécolle et al. (2002) |
|  | Ain, Allier, Alpes-de-Haute-Provence, Hautes-Alpes, Ardèche, Ardennes, Ariège, Aube, Aude, Aveyron, Bouches-du-Rhône, Cantal, Charente, Charente-Maritime, Cher, Corrèze, Côte-d'Or, Creuse, Dordogne, Doubs, Drôme, Eure, Eure-et-Loir, Finistère, Corse, Corse, Gard, Gironde, Ille-et-Vilaine, Indre-et-Loire, Isère, Jura, Loire, Haute-Loire, Loire-Atlantique, Loiret, Maine-et-Loire, Manche, Marne, Haute-Marne, Meuse, Morbihan, Moselle, Nièvre, Nord, Oise, Orne, Pas-de-Calais, Puy-de-Dôme, Pyrénées-Atlantiques, Bas-Rhin, Haut-Rhin, Rhône, Haute-Saône, Saône-et-Loire, Sarthe, Savoie, Haute-Savoie, Seine-et-Marne, Yvelines, Deux-Sèvres, Somme, Tarn, Tarn-et-Garonne, Var, Vaucluse, Vienne, Vosges, Yonne, Territoire-de-Belfort | 2010 | 12 | January, February, March, April, May, June, July, August, September, October, November, December | Winter,  Spring,  Summer,  Autumn | NA | OVI trap | NA | Garros (2022) |
|  | Bas-Rhin, Haut-Rhin, Vosges, Ardennes, Aisne, Jura, Var | NA | NA | NA | NA | NA | NA | NA | Kremer (1965) |
|  | Bas-Rhin, Haut-Rhin | 1976 | 8 | March, April, May, June, July, August, September, October | Spring,  Summer,  Autumn | NA | Larvae capture | NA | Kremer et al. (1978) |
|  | Corse | 2009 | 2 | April, June | Spring | NA | OVI trap | NA | Mathieu (2011) |
|  | Corse | NA | NA | NA | NA | NA | OVI trap | NA | Mathieu et al. (2011) |
|  | Corse | 2002-2009 | 120 | January, February, March, April, May, June, July, August, September, October, November, December | Winter,  Spring,  Summer,  Autumn | NA | OVI trap | NA | Mehlhorn (2012) |
|  | Ardennes | 2009-2010 | 18 | January, February, March, April, May, June, July, August, September, October, November, December | Winter,  Spring,  Summer,  Autumn | 271, 243 | CDC trap | NA | Ninio (2011) |
|  | Ardennes | 2009-2010 | 26 | January, February, March, April, May, June, July, August, September, October, November, December | Winter,  Spring,  Summer,  Autumn | NA | UV CDC trap,  Soil sampling | NA | Ninio (2011) |
|  | Hérault | 2002 | 8 | April, May, June, July, August, September, October, November | Spring,  Summer,  Autumn | NA | OVI trap | NA | Perrin et al. (2006) |
|  | Bas-Rhin | 1977-1978 | 17 | April, May, June, July, August, September, October, November, December, January, February, March | Spring,  Summer,  Autumn,  Winter | 150 | CDC trap,  Soil sampling | NA | Rieb (1987) |
|  | Corse | 2000 | 0.13 | October | Autumn | NA | OVI trap | NA | Zientara et al. (2000) |
|  | Haute-Marne | 2012 | NA | July | Summer | NA | NA | NA | GBIF (2023) |
| *Culicoides*  *kurensis* | Corse | 2000 | 12 | October, November, December, January, February, March, April, May, June, July, August, September | Winter,  Spring,  Summer,  Autumn | NA | OVI trap | NA | Delécolle et al. (2002) |
|  | Aveyron, Cantal, Corse, Puy-de-Dôme, Var | 2010 | 12 | January, February, March, April, May, June, July, August, September, October, November, December | Winter,  Spring,  Summer,  Autumn | NA | OVI trap | NA | Garros (2022) |
|  | Bouches-du-Rhône | 2009 | 2 | April, June | Spring | NA | OVI trap | NA | Mathieu (2011) |
|  | Corse | 2002-2009 | 120 | January, February, March, April, May, June, July, August, September, October, November, December | Winter,  Spring,  Summer,  Autumn | NA | OVI trap | NA | Mehlhorn (2012) |
|  | Bouches-du-Rhône | 2002 | 8 | April, May, June, July, August, September, October, November | Spring,  Summer,  Autumn | NA | OVI trap | NA | Perrin et al. (2006) |
|  | Corse | 2000 | 0.13 | October | Autumn | NA | OVI trap | NA | Zientara et al. (2000) |
| *Culicoides*  *longipennis* | Saône-et-Loire, Hautes-Pyrénées | 2008-2012 | 3 | June, July, August | Summer | 215-1500 | OVI trap | NA | Rossi et al. (2019) |
|  | Hérault, Var, Vaucluse | 2004 | 8 | April, May, June, July, August, September, October, November | NA | NA | UV CDC trap | NA | Cetre-Sossah (2010) |
|  | Corse | 2000 | 12 | October, November, December, January, February, March, April, May, June, July, August, September | Winter,  Spring,  Summer,  Autumn | NA | OVI trap | NA | Delécolle et al. (2002) |
|  | Alpes-de-Haute-Provence, Hautes-Alpes, Ardèche, Ariège, Aude, Bouches-du-Rhône, Drôme, Corse, Isère, Var | 2010 | 12 | January, February, March, April, May, June, July, August, September, October, November, December | Winter,  Spring,  Summer,  Autumn | NA | OVI trap | NA | Garros (2022) |
|  | Var | NA | NA | NA | NA | NA | NA | NA | Kremer (1965) |
|  | Corse | 1964, 1970 | 2 | July | Summer | 900-1080 | CDC trap,  Soil sampling | NA | Kremer et al. (1971) |
|  | Vaucluse | 2009 | 2 | April, June | Spring | NA | OVI trap | NA | Mathieu (2011) |
|  | Vaucluse | NA | NA | NA | NA | NA | OVI trap | NA | Mathieu et al. (2011) |
|  | Corse | 2002-2009 | 120 | January, February, March, April, May, June, July, August, September, October, November, December | Winter,  Spring,  Summer,  Autumn | NA | OVI trap | NA | Mehlhorn (2012) |
|  | Corse | 1971 | NA | NA | NA | NA | NA | NA | Zientara et al. (2000) |
| *Culicoides*  *malevillei* | Var | 2004 | 8 | April, May, June, July, August, September, October, November | NA | NA | UV CDC trap | NA | Cetre-Sossah (2010) |
|  | Corse | 2000 | 12 | October, November, December, January, February, March, April, May, June, July, August, September | Winter,  Spring,  Summer,  Autumn | NA | OVI trap | NA | Delécolle et al. (2002) |
|  | Var | 2009 | 2 | April, June | Spring | NA | OVI trap | NA | Mathieu (2011) |
|  | Var | NA | NA | NA | NA | NA | OVI trap | NA | Mathieu et al. (2011) |
|  | Corse | 2002-2009 | 120 | January, February, March, April, May, June, July, August, September, October, November, December | Winter,  Spring,  Summer,  Autumn | NA | OVI trap | NA | Mehlhorn (2012) |
|  | Var | 2002 | 8 | April, May, June, July, August, September, October, November | Spring,  Summer,  Autumn | NA | OVI trap | NA | Perrin et al. (2006) |
|  | Corse | 2000 | 0.13 | October | Autumn | NA | OVI trap | NA | Zientara et al. (2000) |
| *Culicoides*  *manchuriensis* | Corse | 2012 | 12 | January, February, March, April, May, June, July, August, September, October, November, December | Winter,  Spring,  Summer,  Autumn | NA | OVI trap | NA | Balenghien et al. (2013) |
|  | Marne, Seine-et-Marne, Yvelines | 2010 | 12 | January, February, March, April, May, June, July, August, September, October, November, December | Winter,  Spring,  Summer,  Autumn | NA | OVI trap | NA | Garros (2022) |
|  | Loir-et-Cher | 2011 | NA | May, June | Spring,  Summer | NA | NA | NA | GBIF (2023) |
| *Culicoides*  *maritimus* | Alpes-Maritimes, Var, Bouches-du-Rhône | 2002 | 8 | April, May, June, July, August, September, October, November | Spring,  Summer,  Autumn | NA | UV CDC trap | NA | Cêtre-Sossah et al. (2004) |
|  | Hautes-Alpes, Hautes-Pyrénées | 2008-2012 | 3 | June, July, August | Summer | 215-1500 | OVI trap | NA | Rossi et al. (2019) |
|  | Alpes-Maritimes, Var, Bouches-du-Rhône, Gard, Hérault, Aude, Pyrénées-Orientales | 2002 | 11 | February, March, April, May, June, July, August, September, October, November, December | Winter,  Spring,  Summer,  Autumn | NA | OVI trap | NA | Baldet et al. (2004) |
|  | Bouches-du-Rhône, Gard, Hérault, Loire-Atlantique, Pyrénées-Orientales | 2004 | 8 | April, May, June, July, August, September, October, November | NA | NA | UV CDC trap | NA | Cetre-Sossah (2010) |
|  | Alpes-de-Haute-Provence, Aude, Bouches-du-Rhône, Cantal, Drôme, Corse, Corse, Hérault, Isère, Pyrénées-Orientales, Tarn, Var, Haute-Vienne | 2010 | 12 | January, February, March, April, May, June, July, August, September, October, November, December | Winter,  Spring,  Summer,  Autumn | NA | OVI trap | NA | Garros (2022) |
|  | Var | NA | NA | NA | NA | NA | NA | NA | Kremer (1965) |
|  | Corse, Pyrénées-Orientales | 2009 | 2 | April, June | Spring | NA | OVI trap | NA | Mathieu (2011) |
|  | Corse, Pyrénées-Orientales | NA | NA | NA | NA | NA | OVI trap | NA | Mathieu et al. (2011) |
|  | Corse | 2002-2009 | 120 | January, February, March, April, May, June, July, August, September, October, November, December | Winter,  Spring,  Summer,  Autumn | NA | OVI trap | NA | Mehlhorn (2012) |
|  | Pyrénées-Orientales | 2002 | 8 | April, May, June, July, August, September, October, November | Spring,  Summer,  Autumn | NA | OVI trap | NA | Perrin et al. (2006) |
|  | Pyrénées-Orientales | 2002 | 8 | April, May, June, July, August, September, October, November | Spring,  Summer,  Autumn | NA | OVI trap | NA | Perrin et al. (2006) |
|  | Hérault, Gard, Bouches-du-Rhône | 1953-1966 | NA | January, February, March, April, May, June, July, August, September, October, November, December | Winter,  Spring,  Summer,  Autumn | NA | Manual capture,  Larvae capture,  CDC trap,  Malaise trap | NA | Rageau et al. (1967) |
|  | Corse | 2000 | 0.13 | October | Autumn | NA | OVI trap | NA | Zientara et al. (2000) |
| *Culicoides*  *minutissimus* | Indre-et-Loire | 2009 | 1 | July, August | Summer | 120 | Cattle bait trap,  OVI trap | NA | Viennet et al. (2013) |
|  | Bas-Rhin, Saône-et-Loire, Hérault, Hautes-Pyrénées | 2008-2012 | 3 | June, July, August | Summer | 215-1500 | OVI trap | NA | Rossi et al. (2019) |
|  | Corse | 2000 | 12 | October, November, December, January, February, March, April, May, June, July, August, September | Winter,  Spring,  Summer,  Autumn | NA | OVI trap | NA | Delécolle et al. (2002) |
|  | Hautes-Alpes, Creuse, Val-d’Oise | 2010 | 12 | January, February, March, April, May, June, July, August, September, October, November, December | Winter,  Spring,  Summer,  Autumn | NA | OVI trap | NA | Garros (2022) |
|  | Manche | NA | NA | NA | NA | NA | NA | NA | Kremer (1965) |
|  | Corse | 2002-2009 | 120 | January, February, March, April, May, June, July, August, September, October, November, December | Winter,  Spring,  Summer,  Autumn | NA | OVI trap | NA | Mehlhorn (2012) |
| *Culicoides*  *montanus* | Corse | 2002-2005 | 5 | May, June, July, August, October | Summer,  Autumn | NA | UV CDC trap | NA | Cetre-Sossah (2010) |
|  | Corse | 2009 | 2 | April, June | Spring | NA | OVI trap | NA | Mathieu (2011) |
|  | Corse | 2004-2006, 2008-2009 | NA | April, May, June, July, August, September | Spring,  Summer | NA | OVI trap | NA | Mathieu (2011) |
|  | Corse | NA | NA | NA | NA | NA | OVI trap | NA | Mathieu et al. (2011) |
|  | Corse | 2002-2009 | 120 | January, February, March, April, May, June, July, August, September, October, November, December | Winter,  Spring,  Summer,  Autumn | NA | OVI trap | NA | Mehlhorn (2012) |
|  | Corse | 2004-2005, 2008 | NA | June, August, September | Summer | NA | NA | NA | GBIF (2023) |
| *Culicoides*  *odiatus* | Saône-et-Loire, Hautes-Alpes, Hérault, Hautes-Pyrénées | 2008-2012 | 3 | June, July, August | Summer | 215-1500 | OVI trap | NA | Rossi et al. (2019) |
|  | Ain, Alpes-de-Haute-Provence, Hautes-Alpes, Alpes-Maritimes, Ardèche, Ariège, Aude, Cantal, Corrèze, Creuse, Drôme, Corse, Corse, Gard, Isère, Haute-Loire, Lozère, Puy-de-Dôme, Rhône, Savoie, Haute-Savoie, Tarn, Var, Vaucluse | 2010 | 12 | January, February, March, April, May, June, July, August, September, October, November, December | Winter,  Spring,  Summer,  Autumn | NA | OVI trap | NA | Garros (2022) |
|  | Corse | 1964, 1970 | 2 | July | Summer | 900-1080 | CDC trap,  Soil sampling | NA | Kremer et al. (1971) |
|  | Corse | 2009 | 2 | April, June | Spring | NA | OVI trap | NA | Mathieu (2011) |
|  | Corse | NA | NA | NA | NA | NA | OVI trap | NA | Mathieu et al. (2011) |
|  | Corse | 2002-2009 | 120 | January, February, March, April, May, June, July, August, September, October, November, December | Winter,  Spring,  Summer,  Autumn | NA | OVI trap | NA | Mehlhorn (2012) |
|  | Corse | 1971 | NA | NA | NA | NA | NA | NA | Zientara et al. (2000) |
|  | Corse | 2000 | 0.13 | October | Autumn | NA | OVI trap | NA | Zientara et al. (2000) |
| *Culicoides*  *paradisionensis* | Corse, Corse | 2010 | 12 | January, February, March, April, May, June, July, August, September, October, November, December | Winter,  Spring,  Summer,  Autumn | NA | OVI trap | NA | Garros (2022) |
|  | Corse | 2002-2009 | 120 | January, February, March, April, May, June, July, August, September, October, November, December | Winter,  Spring,  Summer,  Autumn | NA | OVI trap | NA | Mehlhorn (2012) |
| *Culicoides*  *paradoxalis* | Corse | 2003 | NA | June | Summer | NA | NA | NA | INPN (2023) |
|  | Corse, Var | 2000, 2002-2005, 2012 | NA | June, August, September, October, | Summer | NA | CDC trap | NA | Ramilo et al. (2013) |
|  | Corse | 2003 | NA | June | Summer | NA | NA | NA | GBIF (2023) |
| *Culicoides*  *parroti* | Bas-Rhin, Hérault, Hautes-Pyrénées | 2008-2012 | 3 | June, July, August | Summer | 215-1500 | OVI trap | NA | Rossi et al. (2019) |
|  | Bouches-du-Rhône, Vaucluse | 2004 | 8 | April, May, June, July, August, September, October, November | NA | NA | UV CDC trap | NA | Cetre-Sossah (2010) |
|  | Corse | 2000 | 12 | October, November, December, January, February, March, April, May, June, July, August, September | Winter,  Spring,  Summer,  Autumn | NA | OVI trap | NA | Delécolle et al. (2002) |
|  | Ain, Alpes-de-Haute-Provence, Ardèche, Ardennes, Aveyron, Calvados, Cantal, Charente, Corrèze, Creuse, Drôme, Corse, Ille-et-Vilaine, Isère, Marne, Meuse, Nièvre, Haute-Savoie, Seine-et-Marne, Yvelines, Tarn-et-Garonne, Vendée, Haute-Vienne | 2010 | 12 | January, February, March, April, May, June, July, August, September, October, November, December | Winter,  Spring,  Summer,  Autumn | NA | OVI trap | NA | Garros (2022) |
|  | Bouches-du-Rhône | 2009 | 2 | April, June | Spring | NA | OVI trap | NA | Mathieu (2011) |
|  | Bouches du Rhône | NA | NA | NA | NA | NA | OVI trap | NA | Mathieu et al. (2011) |
|  | Corse | 2002-2009 | 120 | January, February, March, April, May, June, July, August, September, October, November, December | Winter,  Spring,  Summer,  Autumn | NA | OVI trap | NA | Mehlhorn (2012) |
|  | Nord, Aisne, Ardennes, Meuse, Meurthe-et-Moselle, Moselle | 2006 | 5 | August, September, October, November, December | Summer,  Autumn | NA | OVI trap | NA | Meiswinkel et al. (2007) |
|  | Ardennes | 2009-2010 | 26 | January, February, March, April, May, June, July, August, September, October, November, December | Winter,  Spring,  Summer,  Autumn | NA | UV CDC trap,  Soil sampling | NA | Ninio (2011) |
|  | Bouches-du-Rhône, Aude | 2002 | 8 | April, May, June, July, August, September, October, November | Spring,  Summer,  Autumn | NA | OVI trap | NA | Perrin et al. (2006) |
| *Culicoides*  *pictipennis* | Gers | 2009 | 5 | February, March, April, May, June | Winter,  Spring | NA | OVI trap | NA | Garros et al. (2011) |
|  | Indre-et-Loire | 2010 | 5 | April, May, June, September, October | Spring,  Summer,  Autumn | NA | OVI trap | NA | Viennet et al. (2012) |
|  | Bas-Rhin | 1981-1982 | NA | NA | NA | NA | NA | NA | Arnold et al. (1982) |
|  | Bas-Rhin, Hérault, Hautes-Pyrénées | 2008-2012 | 3 | June, July, August | Summer | 215-1500 | OVI trap | NA | Rossi et al. (2019) |
|  | Ain, Alpes-de-Haute-Provence, Hautes-Alpes, Ardèche, Ariège, Aube, Aude, Bouches-du-Rhône, Cantal, Charente, Charente-Maritime, Côtes-d’Armor, Creuse, Dordogne, Eure-et-Loir, Finistère, Corse, Corse, Haute-Garonne, Gers, Gironde, Hérault, Ille-et-Vilaine, Indre, Indre-et-Loire, Jura, Landes, Loir-et-Cher, Loire-Atlantique, Lot, Maine-et-Loire, Manche, Marne, Haute-Marne, Meuse, Morbihan, Nièvre, Orne, Pyrénées-Atlantiques, Hautes-Pyrénées, Haute-Saône, Saône-et-Loire, Sarthe, Deux-Sèvres, Somme, Tarn-et-Garonne, Var, Vendée, Vienne, Haute-Vienne, Vosges, Yonne | 2010 | 12 | January, February, March, April, May, June, July, August, September, October, November, December | Winter,  Spring,  Summer,  Autumn | NA | OVI trap | NA | Garros (2022) |
|  | Bas-Rhin, Haut-Rhin, Vosges | NA | NA | NA | NA | NA | NA | NA | Kremer (1965) |
|  | Bas-Rhin, Haut-Rhin | 1976 | 8 | March, April, May, June, July, August, September, October | Spring,  Summer,  Autumn | NA | Larvae capture | NA | Kremer et al. (1978) |
|  | Haute-Saône | 2009 | 2 | April, June | Spring | NA | OVI trap | NA | Mathieu (2011) |
|  | Haute-Saône | NA | NA | NA | NA | NA | OVI trap | NA | Mathieu et al. (2011) |
|  | Corse | 2002-2009 | 120 | January, February, March, April, May, June, July, August, September, October, November, December | Winter,  Spring,  Summer,  Autumn | NA | OVI trap | NA | Mehlhorn (2012) |
|  | Ardennes | 2009-2010 | 26 | January, February, March, April, May, June, July, August, September, October, November, December | Winter,  Spring,  Summer,  Autumn | NA | UV CDC trap,  Soil sampling | NA | Ninio (2011) |
|  | Bas-Rhin | 2002 | 8 | April, May, June, July, August, September, October, November | Spring,  Summer,  Autumn | NA | OVI trap | NA | Perrin et al. (2006) |
| *Culicoides*  *poperinghensis* | Gers, Ille-et-Vilaine, Cher | 2009 | 5 | February, March, April, May, June | Winter,  Spring | NA | OVI trap | NA | Garros et al. (2011) |
|  | Ardennes, Marne, Deux-Sèvres | 2008-2009 | NA | NA | NA | NA | UV CDC trap,  CDC trap | NA | Ninio et al. (2011a) |
|  | Indre-et-Loire | 2010 | 5 | April, May, June, September, October | Spring,  Summer,  Autumn | NA | OVI trap | NA | Viennet et al. (2012) |
|  | Bas-Rhin, Saône-et-Loire, Hautes-Alpes, Hérault, Hautes-Pyrénées | 2008-2012 | 3 | June, July, August | Summer | 215-1500 | OVI trap | NA | Rossi et al. (2019) |
|  | Ain, Allier, Alpes-de-Haute-Provence, Hautes-Alpes, Ardèche, Ariège, Aube, Aude, Bouches-du-Rhône, Cantal, Charente, Charente-Maritime, Côtes-d’Armor, Dordogne, Corse, Corse, Gers, Indre, Isère, Loir-et-Cher, Haute-Loire, Lozère, Manche, Marne, Haute-Marne, Mayenne, Meuse, Nièvre, Oise, Orne, Puy-de-Dôme, Pyrénées-Atlantiques, Saône-et-Loire, Sarthe, Savoie, Haute-Savoie, Seine-et-Marne, Yvelines, Tarn, Tarn-et-Garonne, Var, Vaucluse, Vienne, Haute-Vienne, Vosges, Yonne | 2010 | 12 | January, February, March, April, May, June, July, August, September, October, November, December | Winter,  Spring,  Summer,  Autumn | NA | OVI trap | NA | Garros (2022) |
|  | Bas-Rhin, Haut-Rhin | 1976 | 8 | March, April, May, June, July, August, September, October | Spring,  Summer,  Autumn | NA | Larvae capture | NA | Kremer et al. (1978) |
|  | Aube, Meurthe-et-Moselle | 2009 | 2 | April, June | Spring | NA | OVI trap | NA | Mathieu (2011) |
|  | Aube | NA | NA | NA | NA | NA | OVI trap | NA | Mathieu et al. (2011) |
|  | Corse | 2002-2009 | 120 | January, February, March, April, May, June, July, August, September, October, November, December | Winter,  Spring,  Summer,  Autumn | NA | OVI trap | NA | Mehlhorn (2012) |
|  | Ardennes | 2009-2010 | 26 | January, February, March, April, May, June, July, August, September, October, November, December | Winter,  Spring,  Summer,  Autumn | NA | UV CDC trap,  Soil sampling | NA | Ninio (2011) |
| *Culicoides*  *pseudoheliophilus* | Vosges | NA | NA | NA | NA | NA | NA | NA | Kremer (1965) |
|  | Corse | 2002-2009 | 120 | January, February, March, April, May, June, July, August, September, October, November, December | Winter,  Spring,  Summer,  Autumn | NA | OVI trap | NA | Mehlhorn (2012) |
| *Culicoides*  *pumilus* | Bas-Rhin, Haut-Rhin | 1976 | 8 | March, April, May, June, July, August, September, October | Spring,  Summer,  Autumn | NA | Larvae capture | NA | Kremer et al. (1978) |
|  | Bas-Rhin | 1977-1978 | 17 | April, May, June, July, August, September, October, November, December, January, February, March | Spring,  Summer,  Autumn,  Winter | 150 | CDC trap,  Soil sampling | NA | Rieb (1987) |
|  | Bas-Rhin | NA | NA | NA | NA | NA | NA | NA | Kremer (1965) |
| *Culicoides*  *reconditus* | Vosges | 1981 | NA | NA | NA | NA | Larvae capture | NA | Waller et al. (1982) |
|  | Ariège, Savoie, Var | 2010 | 12 | January, February, March, April, May, June, July, August, September, October, November, December | Winter,  Spring,  Summer,  Autumn | NA | OVI trap | NA | Garros (2022) |
|  | Vosges | NA | NA | NA | NA | NA | NA | NA | Kremer (1965) |
|  | Haut-Rhin | 2009 | 2 | April, June | Spring | NA | OVI trap | NA | Mathieu (2011) |
|  | Haut-Rhin | NA | NA | NA | NA | NA | OVI trap | NA | Mathieu et al. (2011) |
| *Culicoides*  *riebi* | Corse | 2002-2009 | 120 | January, February, March, April, May, June, July, August, September, October, November, December | Winter,  Spring,  Summer,  Autumn | NA | OVI trap | NA | Mehlhorn (2012) |
| *Culicoides*  *riouxi* | Aveyron, Creuse, Doubs | 2010 | 12 | January, February, March, April, May, June, July, August, September, October, November, December | Winter,  Spring,  Summer,  Autumn | NA | OVI trap | NA | Garros (2022) |
|  | Bas-Rhin | NA | NA | NA | NA | NA | NA | NA | Kremer (1965) |
|  | Ardennes | 2009 | 2 | April, June | Spring | NA | OVI trap | NA | Mathieu (2011) |
|  | Ardennes | NA | NA | NA | NA | NA | OVI trap | NA | Mathieu et al. (2011) |
|  | Corse | 2002-2009 | 120 | January, February, March, April, May, June, July, August, September, October, November, December | Winter,  Spring,  Summer,  Autumn | NA | OVI trap | NA | Mehlhorn (2012) |
|  | Ardennes | 2009-2010 | 26 | January, February, March, April, May, June, July, August, September, October, November, December | Winter,  Spring,  Summer,  Autumn | NA | UV CDC trap,  Soil sampling | NA | Ninio (2011) |
|  | Ardennes | 2009 | NA | May | Spring | NA | NA | NA | GBIF (2023) |
| *Culicoides*  *saevus* | Alpes-de-Haute-Provence | 2010 | 12 | January, February, March, April, May, June, July, August, September, October, November, December | Winter,  Spring,  Summer,  Autumn | NA | OVI trap | NA | Garros (2022) |
|  | Corse | 2002-2009 | 120 | January, February, March, April, May, June, July, August, September, October, November, December | Winter,  Spring,  Summer,  Autumn | NA | OVI trap | NA | Mehlhorn (2012) |
| *Culicoides*  *sahariensis* | Hérault, Pyrénées-Orientales, Vaucluse | 2004 | 8 | April, May, June, July, August, September, October, November | NA | NA | UV CDC trap | NA | Cetre-Sossah (2010) |
|  | Hérault | 2009 | 2 | April, June | Spring | NA | OVI trap | NA | Mathieu (2011) |
|  | Hérault | NA | NA | NA | NA | NA | OVI trap | NA | Mathieu et al. (2011) |
|  | Corse | 2002-2009 | 120 | January, February, March, April, May, June, July, August, September, October, November, December | Winter,  Spring,  Summer,  Autumn | NA | OVI trap | NA | Mehlhorn (2012) |
|  | Hérault | 2002 | 8 | April, May, June, July, August, September, October, November | Spring,  Summer,  Autumn | NA | OVI trap | NA | Perrin et al. (2006) |
| *Culicoides*  *salinarius* | Indre-et-Loire | 2009 | 1 | July, August | Summer | 120 | Cattle bait trap,  OVI trap | NA | Viennet et al. (2013) |
|  | Indre-et-Loire | 2010 | 5 | April, May, June, September, October | Spring,  Summer,  Autumn | NA | OVI trap | NA | Viennet et al. (2012) |
|  | Hautes-Pyrénées | 2008-2012 | 3 | June, July, August | Summer | 215-1500 | OVI trap | NA | Rossi et al. (2019) |
|  | Ain, Ardennes, Aube, Calvados, Cantal, Charente, Doubs, Corse, Ille-et-Vilaine, Indre, Jura, Loir-et-Cher, Loire, Loire-Atlantique, Maine-et-Loire, Marne, Nièvre, Nord, Orne, Pas-de-Calais, Haute-Saône, Saône-et-Loire, Haute-Savoie, Seine-et-Marne, Yvelines, Deux-Sèvres, Vendée, Haute-Vienne, Vosges | 2010 | 12 | January, February, March, April, May, June, July, August, September, October, November, December | Winter,  Spring,  Summer,  Autumn | NA | OVI trap | NA | Garros (2022) |
|  | Moselle, Haut-Rhin | NA | NA | NA | NA | NA | NA | NA | Kremer (1965) |
|  | Bas-Rhin, Haut-Rhin | 1976 | 8 | March, April, May, June, July, August, September, October | Spring,  Summer,  Autumn | NA | Larvae capture | NA | Kremer et al. (1978) |
|  | Corse | 2002-2009 | 120 | January, February, March, April, May, June, July, August, September, October, November, December | Winter,  Spring,  Summer,  Autumn | NA | OVI trap | NA | Mehlhorn (2012) |
|  | Nord, Aisne, Ardennes, Meuse, Meurthe-et-Moselle, Moselle | 2006 | 5 | August, September, October, November, December | Summer,  Autumn | NA | OVI trap | NA | Meiswinkel et al. (2007) |
|  | Ardennes | 2009-2010 | 26 | January, February, March, April, May, June, July, August, September, October, November, December | Winter,  Spring,  Summer,  Autumn | NA | UV CDC trap,  Soil sampling | NA | Ninio (2011) |
| *Culicoides*  *santonicus* | Indre-et-Loire | 2010 | 5 | April, May, June, September, October | Spring,  Summer,  Autumn | NA | OVI trap | NA | Viennet et al. (2012) |
|  | Hérault, Hautes-Pyrénées | 2008-2012 | 3 | June, July, August | Summer | 215-1500 | OVI trap | NA | Rossi et al. (2019) |
|  | Charente-Maritime, Dordogne, Corse, Haute-Garonne, Gers, Gironde, Indre, Indre-et-Loire, Landes, Loir-et-Cher, Loire-Atlantique, Morbihan, Orne, Pyrénées-Atlantiques, Hautes-Pyrénées, Sarthe, Tarn-et-Garonne, Vendée | 2010 | 12 | January, February, March, April, May, June, July, August, September, October, November, December | Winter,  Spring,  Summer,  Autumn | NA | OVI trap | NA | Garros (2022) |
|  | Corse | 2009 | 2 | April, June | Spring | NA | OVI trap | NA | Mathieu (2011) |
|  | Corse | NA | NA | NA | NA | NA | OVI trap | NA | Mathieu et al. (2011) |
|  | Corse | 2002-2009 | 120 | January, February, March, April, May, June, July, August, September, October, November, December | Winter,  Spring,  Summer,  Autumn | NA | OVI trap | NA | Mehlhorn (2012) |
| *Culicoides*  *segnis* | Bas-Rhin, Saône-et-Loire, Hautes-Alpes, Hérault | 2008-2012 | 3 | June, July, August | Summer | 215-1500 | OVI trap | NA | Rossi et al. (2019) |
|  | Ain, Aveyron, Cantal, Doubs, Haut-Rhin, Tarn, Vosges | 2010 | 12 | January, February, March, April, May, June, July, August, September, October, November, December | Winter,  Spring,  Summer,  Autumn | NA | OVI trap | NA | Garros (2022) |
|  | Bas-Rhin, Vosges | NA | NA | NA | NA | NA | NA | NA | Kremer (1965) |
|  | Haut-Rhin | 2009 | 2 | April, June | Spring | NA | OVI trap | NA | Mathieu (2011) |
|  | Haut-Rhin | NA | NA | NA | NA | NA | OVI trap | NA | Mathieu et al. (2011) |
|  | Corse | 2002-2009 | 120 | January, February, March, April, May, June, July, August, September, October, November, December | Winter,  Spring,  Summer,  Autumn | NA | OVI trap | NA | Mehlhorn (2012) |
|  | Ardennes | 2009-2010 | 26 | January, February, March, April, May, June, July, August, September, October, November, December | Winter,  Spring,  Summer,  Autumn | NA | UV CDC trap,  Soil sampling | NA | Ninio (2011) |
|  | Haut-Rhin | 2009 | NA | June | Summer | NA | NA | NA | GBIF (2023) |
| *Culicoides*  *semimaculatus* | Bas-Rhin | NA | NA | NA | NA | NA | Larvae capture | NA | Chacker (1982) |
|  | Eure, Corse, Orne | 2010 | 12 | January, February, March, April, May, June, July, August, September, October, November, December | Winter,  Spring,  Summer,  Autumn | NA | OVI trap | NA | Garros (2022) |
|  | Corse | 2002-2009 | 120 | January, February, March, April, May, June, July, August, September, October, November, December | Winter,  Spring,  Summer,  Autumn | NA | OVI trap | NA | Mehlhorn (2012) |
| *Culicoides*  *sergenti* | Vosges | NA | NA | NA | NA | NA | NA | NA | Kremer (1965) |
| *Culicoides*  *shaklawensis* | Alpes-Maritimes | 2004 | 8 | April, May, June, July, August, September, October, November | NA | NA | UV CDC trap | NA | Cetre-Sossah (2010) |
|  | Hautes-Alpes, Ariège, Drôme, Isère | 2010 | 12 | January, February, March, April, May, June, July, August, September, October, November, December | Winter,  Spring,  Summer,  Autumn | NA | OVI trap | NA | Garros (2022) |
|  | Corse | 2002-2009 | 120 | January, February, March, April, May, June, July, August, September, October, November, December | Winter,  Spring,  Summer,  Autumn | NA | OVI trap | NA | Mehlhorn (2012) |
| *Culicoides*  *simulator* | Indre-et-Loire | 2010 | 5 | April, May, June, September, October | Spring,  Summer,  Autumn | NA | OVI trap | NA | Viennet et al. (2012) |
|  | Bas-Rhin, Saône-et-Loire, Hautes-Pyrénées | 2008-2012 | 3 | June, July, August | Summer | 215-1500 | OVI trap | NA | Rossi et al. (2019) |
|  | Alpes-de-Haute-Provence, Hautes-Alpes, Ardèche, Ariège, Aude, Cantal, Cher, Creuse, Drôme, Maine-et-Loire, Marne, Haute-Marne, Meuse, Moselle, Nièvre, Rhône, Saône-et-Loire, Seine-et-Marne, Var, Vosges, Yonne | 2010 | 12 | January, February, March, April, May, June, July, August, September, October, November, December | Winter,  Spring,  Summer,  Autumn | NA | OVI trap | NA | Garros (2022) |
|  | Yonne | 2009 | 2 | April, June | Spring | NA | OVI trap | NA | Mathieu (2011) |
|  | Yonne | NA | NA | NA | NA | NA | OVI trap | NA | Mathieu et al. (2011) |
|  | Ardennes | 2009-2010 | 26 | January, February, March, April, May, June, July, August, September, October, November, December | Winter,  Spring,  Summer,  Autumn | NA | UV CDC trap,  Soil sampling | NA | Ninio (2011) |
| *Culicoides*  *stigma* | Vosges | 1981 | NA | NA | NA | NA | Larvae capture | NA | Waller et al. (1982) |
|  | Bas-Rhin, Saône-et-Loire | 2008-2012 | 3 | June, July, August | Summer | 215-1500 | OVI trap | NA | Rossi et al. (2019) |
|  | Calvados, Charente, Doubs, Jura, Landes, Marne, Nièvre, Bas-Rhin, Haute-Saône, Sarthe, Seine-et-Marne, Yvelines, Tarn-et-Garonne, Vienne | 2010 | 12 | January, February, March, April, May, June, July, August, September, October, November, December | Winter,  Spring,  Summer,  Autumn | NA | OVI trap | NA | Garros (2022) |
|  | Vosges, Ardennes | NA | NA | NA | NA | NA | NA | NA | Kremer (1965) |
|  | Bas-Rhin, Haut-Rhin | 1976 | 8 | March, April, May, June, July, August, September, October | Spring,  Summer,  Autumn | NA | Larvae capture | NA | Kremer et al. (1978) |
|  | Seine-et-Marne | 2009 | 2 | April, June | Spring | NA | OVI trap | NA | Mathieu (2011) |
|  | Seine-et-Marne | NA | NA | NA | NA | NA | OVI trap | NA | Mathieu et al. (2011) |
|  | Ardennes | 2009-2010 | 18 | January, February, March, April, May, June, July, August, September, October, November, December | Winter,  Spring,  Summer,  Autumn | 271, 243 | CDC trap | NA | Ninio (2011) |
|  | Ardennes | 2009-2010 | 26 | January, February, March, April, May, June, July, August, September, October, November, December | Winter,  Spring,  Summer,  Autumn | NA | UV CDC trap,  Soil sampling | NA | Ninio (2011) |
| *Culicoides*  *tauricus* | Saône-et-Loire, Hautes-Pyrénées | 2008-2012 | 3 | June, July, August | Summer | 215-1500 | OVI trap | NA | Rossi et al. (2019) |
|  | Alpes-de-Haute-Provence, Hautes-Alpes, Drôme, Isère, Var, Vaucluse | 2010 | 12 | January, February, March, April, May, June, July, August, September, October, November, December | Winter,  Spring,  Summer,  Autumn | NA | OVI trap | NA | Garros (2022) |
|  | Vaucluse, Hautes-Alpes | 2009 | 2 | April, June | Spring | NA | OVI trap | NA | Mathieu (2011) |
|  | Vaucluse | NA | NA | NA | NA | NA | OVI trap | NA | Mathieu et al. (2011) |
|  | Corse | 2002-2009 | 120 | January, February, March, April, May, June, July, August, September, October, November, December | Winter,  Spring,  Summer,  Autumn | NA | OVI trap | NA | Mehlhorn (2012) |
| *Culicoides*  *tbilisicus* | Hérault | 2008-2012 | 3 | June, July, August | Summer | 215-1500 | OVI trap | NA | Rossi et al. (2019) |
|  | Ain, Allier, Alpes-de-Haute-Provence, Hautes-Alpes, Ardèche, Ariège, Aveyron, Bouches-du-Rhône, Calvados, Cantal, Corrèze, Drôme, Corse, Hérault, Isère, Loire, Haute-Loire, Nièvre, Puy-de-Dôme, Vaucluse, Haute-Vienne | 2010 | 12 | January, February, March, April, May, June, July, August, September, October, November, December | Winter,  Spring,  Summer,  Autumn | NA | OVI trap | NA | Garros (2022) |
|  | Corse | 2009 | 2 | April, June | Spring | NA | OVI trap | NA | Mathieu (2011) |
|  | Corse | NA | NA | NA | NA | NA | OVI trap | NA | Mathieu et al. (2011) |
|  | Corse | 2002-2009 | 120 | January, February, March, April, May, June, July, August, September, October, November, December | Winter,  Spring,  Summer,  Autumn | NA | OVI trap | NA | Mehlhorn (2012) |
| *Culicoides*  *truncorum* | Bas-Rhin | NA | NA | NA | NA | NA | Larvae capture | NA | Chacker (1982) |
|  | Vosges | 1981 | NA | NA | NA | NA | Larvae capture | NA | Waller et al. (1982) |
|  | Vosges | NA | NA | NA | NA | NA | NA | NA | Kremer (1965) |
|  | Yonne | 2009 | 2 | April, June | Spring | NA | OVI trap | NA | Mathieu (2011) |
|  | Yonne | NA | NA | NA | NA | NA | OVI trap | NA | Mathieu et al. (2011) |
|  | Corse | 2002-2009 | 120 | January, February, March, April, May, June, July, August, September, October, November, December | Winter,  Spring,  Summer,  Autumn | NA | OVI trap | NA | Mehlhorn (2012) |
| *Culicoides*  *univittatus* | Charente-Maritime, Corse, Corse | 2010 | 12 | January, February, March, April, May, June, July, August, September, October, November, December | Winter,  Spring,  Summer,  Autumn | NA | OVI trap | NA | Garros (2022) |
|  | Hérault | NA | NA | NA | NA | NA | NA | NA | Kremer (1965) |
|  | Corse | 2009 | 2 | April, June | Spring | NA | OVI trap | NA | Mathieu (2011) |
|  | Corse | NA | NA | NA | NA | NA | OVI trap | NA | Mathieu et al. (2011) |
|  | Corse | 2002-2009 | 120 | January, February, March, April, May, June, July, August, September, October, November, December | Winter,  Spring,  Summer,  Autumn | NA | OVI trap | NA | Mehlhorn (2012) |
| *Culicoides*  *vexans* | Indre-et-Loire | 2009 | 1 | July, August | Summer | 120 | Cattle bait trap,  OVI trap | NA | Viennet et al. (2013) |
|  | Gers, Cher | 2009 | 5 | February, March, April, May, June | Winter,  Spring | NA | OVI trap | NA | Garros et al. (2011) |
|  | Indre-et-Loire | 2010 | 5 | April, May, June, September, October | Spring,  Summer,  Autumn | NA | OVI trap | NA | Viennet et al. (2012) |
|  | Bas-Rhin, Saône-et-Loire, Hautes-Alpes, Hérault | 2008-2012 | 3 | June, July, August | Summer | 215-1500 | OVI trap | NA | Rossi et al. (2019) |
|  | Ardennes, Aube, Bouches-du-Rhône, Charente, Charente-Maritime, Cher, Côte-d'Or, Côtes-d’Armor, Dordogne, Eure-et-Loir, Finistère, Gironde, Ille-et-Vilaine, Indre, Indre-et-Loire, Isère, Jura, Loir-et-Cher, Loire-Atlantique, Loiret, Maine-et-Loire, Manche, Marne, Haute-Marne, Mayenne, Meuse, Morbihan, Moselle, Nièvre, Nord, Oise, Orne, Pyrénées-Atlantiques, Bas-Rhin, Haute-Saône, Saône-et-Loire, Sarthe, Seine-et-Marne, Yvelines, Deux-Sèvres, Tarn-et-Garonne, Vendée, Vienne, Haute-Vienne, Vosges, Yonne | 2010 | 12 | January, February, March, April, May, June, July, August, September, October, November, December | Winter,  Spring,  Summer,  Autumn | NA | OVI trap | NA | Garros (2022) |
|  | Moselle | 1995 | NA | May | Spring | NA | NA | NA | INPN (2023) |
|  | Deux-Sèvres | NA | NA | NA | NA | NA | NA | NA | Kieffer (1925) |
|  | Gironde, Bas-Rhin, Manche, Drôme | NA | NA | NA | NA | NA | NA | NA | Kremer (1965) |
|  | Saône-et-Loire | 2009 | 2 | April, June | Spring | NA | OVI trap | NA | Mathieu (2011) |
|  | Saône-et-Loire | NA | NA | NA | NA | NA | OVI trap | NA | Mathieu et al. (2011) |
|  | Corse | 2002-2009 | 120 | January, February, March, April, May, June, July, August, September, October, November, December | Winter,  Spring,  Summer,  Autumn | NA | OVI trap | NA | Mehlhorn (2012) |
|  | Ardennes | 2009-2010 | 18 | January, February, March, April, May, June, July, August, September, October, November, December | Winter,  Spring,  Summer,  Autumn | 271, 243 | CDC trap | NA | Ninio (2011) |
|  | Ardennes | 2009-2010 | 26 | January, February, March, April, May, June, July, August, September, October, November, December | Winter,  Spring,  Summer,  Autumn | NA | UV CDC trap,  Soil sampling | NA | Ninio (2011) |
|  | Moselle | 1995 | NA | May | Spring | NA | NA | NA | GBIF (2023) |
| *Culicoides*  *vidourlensis* | Corse | 2002-2009 | 120 | January, February, March, April, May, June, July, August, September, October, November, December | Winter,  Spring,  Summer,  Autumn | NA | OVI trap | NA | Mehlhorn (2012) |
|  | Corse | 2000 | 0.13 | October | Autumn | NA | OVI trap | NA | Zientara et al. (2000) |
| Achrayi  Group | Indre-et-Loire | 2009 | 1 | July, August | Summer | 120 | Cattle bait trap,  OVI trap | NA | Viennet et al. (2013) |
|  | Cher | 2009 | 5 | February, March, April, May, June | Winter,  Spring | NA | OVI trap | NA | Garros et al. (2011) |
|  | Ardennes, Marne, Deux-Sèvres | 2008-2009 | NA | NA | NA | NA | UV CDC trap,  CDC trap | NA | Ninio et al. (2011a) |
|  | Ardennes | 2006 | 4 | September, October, November, December | Autumn | NA | OVI trap | NA | Baldet et al. (2008) |
|  | Indre-et-Loire | 2010 | 5 | April, May, June, September, October | Spring,  Summer,  Autumn | NA | OVI trap | NA | Viennet et al. (2012) |
|  | Vosges | 1981 | NA | NA | NA | NA | Larvae capture | NA | Waller et al. (1982) |
|  | Bas-Rhin, Saône-et-Loire, Hautes-Alpes, Hérault, Hautes-Pyrénées | 2008-2012 | 3 | June, July, August | Summer | 215-1500 | OVI trap | NA | Rossi et al. (2019) |
|  | Corse | 2009 | 12 | January, February, March, April, May, June, July, August, September, October, November, December | Winter,  Spring,  Summer,  Autumn | NA | OVI trap | NA | Balenghien et al. (2010) |
|  | Var | 2004 | 8 | April, May, June, July, August, September, October, November | NA | NA | UV CDC trap | NA | Cetre-Sossah (2010) |
|  | Corse | 2000 | 12 | October, November, December, January, February, March, April, May, June, July, August, September | Winter,  Spring,  Summer,  Autumn | NA | OVI trap | NA | Delécolle et al. (2002) |
|  | Ain, Alpes-de-Haute-Provence, Ardèche, Ardennes, Ariège, Aube, Aude, Aveyron, Bas-Rhin, Calvados, Cantal, Charente, Charente-Maritime, Cher, Corrèze, Corse, Côte-d'Or, Creuse, Deux-Sèvres, Dordogne, Doubs, Drôme, Eure-et-Loir, Gers, Gironde, Haute-Loire, Haute-Marne, Hautes-Alpes, Haute-Saône, Haute-Savoie, Haute-Vienne, Haut-Rhin, Ille-et-Vilaine, Indre, Indre-et-Loire, Isère, Jura, Landes, Loire, Loire-Atlantique, Loiret, Loir-et-Cher, Lot, Lozère, Marne, Mayenne, Meuse, Morbihan, Moselle, Nièvre, Nord, Oise, Puy-de-Dôme, Pyrénées-Atlantiques, Pyrénées-Orientales, Rhône, Saône-et-Loire, Savoie, Seine-et-Marne, Somme, Tarn, Tarn-et-Garonne, Territoire-de-Belfort, Var, Vaucluse, Vendée, Vienne, Vosges, Yonne, Yvelines | 2010 | 12 | January, February, March, April, May, June, July, August, September, October, November, December | Winter,  Spring,  Summer,  Autumn | NA | OVI trap | NA | Garros (2022) |
|  | Charente-Maritime | NA | NA | NA | NA | NA | NA | NA | Kieffer (1925) |
|  | Ardennes, Bas-Rhin, Drôme, Jura, Manche, Pyrénées-Orientales, Rhône, Seine-et-Marne, Vosges | NA | NA | NA | NA | NA | NA | NA | Kremer (1965) |
|  | Corse | 1964, 1970 | 2 | July | Summer | 900-1080 | CDC trap,  Soil sampling | NA | Kremer et al. (1971) |
|  | Bas-Rhin, Haut-Rhin | 1976 | 8 | March, April, May, June, July, August, September, October | Spring,  Summer,  Autumn | NA | Larvae capture | NA | Kremer et al. (1978) |
|  | Yonne, Haute-Saône, Bas-Rhin, Var | 2009 | 2 | April, June | Spring | NA | OVI trap | NA | Mathieu (2011) |
|  | Yonne, Haute-Saône, Bas-Rhin, Var | NA | NA | NA | NA | NA | OVI trap | NA | Mathieu et al. (2011) |
|  | Corse | 2002-2009 | 120 | January, February, March, April, May, June, July, August, September, October, November, December | Winter,  Spring,  Summer,  Autumn | NA | OVI trap | NA | Mehlhorn (2012) |
|  | Nord, Aisne, Ardennes, Meuse, Meurthe-et-Moselle, Moselle | 2006 | 5 | August, September, October, November, December | Summer,  Autumn | NA | OVI trap | NA | Meiswinkel et al. (2007) |
|  | Ardennes | 2009-2010 | 18 | January, February, March, April, May, June, July, August, September, October, November, December | Winter,  Spring,  Summer,  Autumn | 271, 243 | CDC trap | NA | Ninio (2011) |
|  | Ardennes | 2009-2010 | 26 | January, February, March, April, May, June, July, August, September, October, November, December | Winter,  Spring,  Summer,  Autumn | NA | UV CDC trap,  Soil sampling | NA | Ninio (2011) |
|  | Var | 2002 | 8 | April, May, June, July, August, September, October, November | Spring,  Summer,  Autumn | NA | OVI trap | NA | Perrin et al. (2006) |
|  | Bas-Rhin | 1977-1978 | 17 | April, May, June, July, August, September, October, November, December, January, February, March | Spring,  Summer,  Autumn,  Winter | 150 | CDC trap,  Soil sampling | NA | Rieb (1987) |
|  | Corse | 1971 | NA | NA | NA | NA | NA | NA | Zientara et al. (2000) |
|  | Yonne, Vosges, Somme | 2009, 2012 | NA | June, July, September | Summer | NA | NA | NA | GBIF (2023) |
| Circumscriptus  Group | Indre-et-Loire | 2009 | 1 | July, August | Summer | 120 | Cattle bait trap,  OVI trap | NA | Viennet et al. (2013) |
|  | Alpes-Maritimes, Var, Bouches-du-Rhône | 2002 | 8 | April, May, June, July, August, September, October, November | Spring,  Summer,  Autumn | NA | UV CDC trap | NA | Cêtre-Sossah et al. (2004) |
|  | Corse, Pyrénées-Orientales, Aude, Hérault, Gard, Bouches-du-Rhône, Var, Alpes-Maritimes | 2002-2003 | 38 | February, March, April, May, June, July, August, September, October, November, December | Winter,  Spring,  Summer,  Autumn | NA | UV CDC trap | NA | Baldet et al. (2005) |
|  | Hautes-Alpes, Hérault, Hautes-Pyrénées | 2008-2012 | 3 | June, July, August | Summer | 215-1500 | OVI trap | NA | Rossi et al. (2019) |
|  | Corse, Alpes-Maritimes, Var, Bouches-du-Rhône, Gard, Hérault, Aude, Pyrénées-Orientales | 2002 | 11 | February, March, April, May, June, July, August, September, October, November, December | Winter,  Spring,  Summer,  Autumn | NA | OVI trap | NA | Baldet et al. (2004) |
|  | Corse | 2009 | 12 | January, February, March, April, May, June, July, August, September, October, November, December | Winter,  Spring,  Summer,  Autumn | NA | OVI trap | NA | Balenghien et al. (2010) |
|  | Alpes-Maritimes, Bouches-du-Rhône, Gard, Haute-Savoie, Hautes-Pyrénées, Hérault, Loire-Atlantique, Pyrénées-Orientales, Var, Vaucluse | 2004 | 8 | April, May, June, July, August, September, October, November | NA | NA | UV CDC trap | NA | Cetre-Sossah (2010) |
|  | Corse | 2000 | 12 | October, November, December, January, February, March, April, May, June, July, August, September | Winter,  Spring,  Summer,  Autumn | NA | OVI trap | NA | Delécolle et al. (2002) |
|  | Ain, Allier, Alpes-de-Haute-Provence, Hautes-Alpes, Aube, Aude, Bouches-du-Rhône, Calvados, Cantal, Charente, Charente-Maritime, Cher, Creuse, Dordogne, Doubs, Drôme, Eure, Finistère, Corse, Corse, Haute-Garonne, Gers, Gironde, Hérault, Ille-et-Vilaine, Indre, Isère, Landes, Loir-et-Cher, Loire, Loire-Atlantique, Maine-et-Loire, Marne, Morbihan, Nièvre, Nord, Oise, Orne, Pyrénées-Atlantiques, Pyrénées-Orientales, Haute-Saône, Saône-et-Loire, Sarthe, Haute-Savoie, Seine-Maritime, Seine-et-Marne, Yvelines, Deux-Sèvres, Tarn-et-Garonne, Var, Vaucluse, Vendée, Vienne, Haute-Vienne | 2010 | 12 | January, February, March, April, May, June, July, August, September, October, November, December | Winter,  Spring,  Summer,  Autumn | NA | OVI trap | NA | Garros (2022) |
|  | Haut-Rhin, Vosges, Haute-Saône, Jura, Moselle, Charente-Maritime, Hérault | NA | NA | NA | NA | NA | NA | NA | Kremer (1965) |
|  | Corse | 1964, 1970 | 2 | July | Summer | 900-1080 | CDC trap,  Soil sampling | NA | Kremer et al. (1971) |
|  | Bas-Rhin, Haut-Rhin | 1976 | 8 | March, April, May, June, July, August, September, October | Spring,  Summer,  Autumn | NA | Larvae capture | NA | Kremer et al. (1978) |
|  | Corse | 2009 | 2 | April, June | Spring | NA | OVI trap | NA | Mathieu (2011) |
|  | Corse | NA | NA | NA | NA | NA | OVI trap | NA | Mathieu et al. (2011) |
|  | Corse | 2002-2009 | 120 | January, February, March, April, May, June, July, August, September, October, November, December | Winter,  Spring,  Summer,  Autumn | NA | OVI trap | NA | Mehlhorn (2012) |
|  | Nord, Aisne, Ardennes, Meuse, Meurthe-et-Moselle, Moselle | 2006 | 5 | August, September, October, November, December | Summer,  Autumn | NA | OVI trap | NA | Meiswinkel et al. (2007) |
|  | Ardennes | 2009-2010 | 18 | January, February, March, April, May, June, July, August, September, October, November, December | Winter,  Spring,  Summer,  Autumn | 271, 243 | CDC trap | NA | Ninio (2011) |
|  | Ardennes | 2009-2010 | 26 | January, February, March, April, May, June, July, August, September, October, November, December | Winter,  Spring,  Summer,  Autumn | NA | UV CDC trap,  Soil sampling | NA | Ninio (2011) |
|  | Moselle | 2002 | 8 | April, May, June, July, August, September, October, November | Spring,  Summer,  Autumn | NA | OVI trap | NA | Perrin et al. (2006) |
|  | Hérault, Gard, Bouches-du-Rhône | 1953-1966 | NA | January, February, March, April, May, June, July, August, September, October, November, December | Winter,  Spring,  Summer,  Autumn | NA | Manual capture,  Larvae capture,  CDC trap,  Malaise trap | NA | Rageau et al. (1967) |
|  | Corse | 1971 | NA | NA | NA | NA | NA | NA | Zientara et al. (2000) |
|  | Corse | 2000 | 0.13 | October | Autumn | NA | OVI trap | NA | Zientara et al. (2000) |
| Fagineus  Group | Corse, Pyrénées-Orientales, Aude, Hérault, Gard, Bouches-du-Rhône, Var, Alpes-Maritimes | 2002-2003 | 38 | February, March, April, May, June, July, August, September, October, November, December | Winter,  Spring,  Summer,  Autumn | NA | UV CDC trap | NA | Baldet et al. (2005) |
|  | Bas-Rhin | NA | NA | NA | NA | NA | Larvae capture | NA | Chacker (1982) |
|  | Bas-Rhin, Hérault, Saône-et-Loire, Hautes-Alpes, Hautes-Pyrénées | 2008-2012 | 3 | June, July, August | Summer | 215-1500 | OVI trap | NA | Rossi et al. (2019) |
|  | Corse, Alpes-Maritimes, Var, Bouches-du-Rhône, Gard, Hérault, Aude, Pyrénées-Orientales | 2002 | 11 | February, March, April, May, June, July, August, September, October, November, December | Winter,  Spring,  Summer,  Autumn | NA | OVI trap | NA | Baldet et al. (2004) |
|  | Corse | 2009 | 12 | January, February, March, April, May, June, July, August, September, October, November, December | Winter,  Spring,  Summer,  Autumn | NA | OVI trap | NA | Balenghien et al. (2010) |
|  | Alpes-Maritimes, Loire-Atlantique, Pyrénées-Orientales, Var, Vaucluse | 2004 | 8 | April, May, June, July, August, September, October, November | NA | NA | UV CDC trap | NA | Cetre-Sossah (2010) |
|  | Corse | 2000 | 12 | October, November, December, January, February, March, April, May, June, July, August, September | Winter,  Spring,  Summer,  Autumn | NA | OVI trap | NA | Delécolle et al. (2002) |
|  | Alpes-de-Haute-Provence, Aveyron, Gard, Lot, Manche, Moselle, Orne, Val-d’Oise, Hautes-Alpes, Alpes-Maritimes, Ardèche, Ariège, Cantal, Corrèze, Drôme, Corse, Haute-Garonne, Isère, Lot, Puy-de-Dôme, Pyrénées-Atlantiques, Rhône, Saône-et-Loire, Seine-Maritime, Tarn-et-Garonne, Var, Vaucluse | 2010 | 12 | January, February, March, April, May, June, July, August, September, October, November, December | Winter,  Spring,  Summer,  Autumn | NA | OVI trap | NA | Garros (2022) |
|  | Charente-Maritime, Vosges | NA | NA | NA | NA | NA | NA | NA | Kremer (1965) |
|  | Bas-Rhin, Corse | 2009 | 2 | April, June | Spring | NA | OVI trap | NA | Mathieu (2011) |
|  | Bas-Rhin, Corse | NA | NA | NA | NA | NA | OVI trap | NA | Mathieu et al. (2011) |
|  | Corse | 2002-2009 | 120 | January, February, March, April, May, June, July, August, September, October, November, December | Winter,  Spring,  Summer,  Autumn | NA | OVI trap | NA | Mehlhorn (2012) |
|  | Pyrénées-Orientales | 2002 | 8 | April, May, June, July, August, September, October, November | Spring,  Summer,  Autumn | NA | OVI trap | NA | Perrin et al. (2006) |
|  | Corse | 2000 | 0.13 | October | Autumn | NA | OVI trap | NA | Zientara et al. (2000) |
| Festivipennis  Group | Indre-et-Loire | 2009 | 1 | July, August | Summer | 120 | Cattle bait trap,  OVI trap | NA | Viennet et al. (2013) |
|  | Ardennes | 2006 | 4 | September, October, November, December | Autumn | NA | OVI trap | NA | Baldet et al. (2008) |
|  | Indre-et-Loire | 2010 | 5 | April, May, June, September, October | Spring,  Summer,  Autumn | NA | OVI trap | NA | Viennet et al. (2012) |
|  | Bas-Rhin | 1981-1982 | NA | NA | NA | NA | NA | NA | Arnold et al. (1982) |
|  | Bas-Rhin | NA | NA | NA | NA | NA | Larvae capture | NA | Chacker (1982) |
|  | Bas-Rhin | NA | NA | NA | NA | 151 | NA | *Chlamydia* sp. | Mialhe et al. (1982) |
|  | Bas-Rhin | 1977 | NA | NA | NA | 142 | NA | Iridovirus | Rieb et al. (1982) |
|  | Bas-Rhin, Saône-et-Loire, Hautes-Alpes, Hérault, Hautes-Pyrénées | 2008-2012 | 3 | June, July, August | Summer | 215-1500 | OVI trap | NA | Rossi et al. (2019) |
|  | Corse | 2009 | 12 | January, February, March, April, May, June, July, August, September, October, November, December | Winter,  Spring,  Summer,  Autumn | NA | OVI trap | NA | Balenghien et al. (2010) |
|  | Alpes-Maritimes, Var | 2004 | 8 | April, May, June, July, August, September, October, November | NA | NA | UV CDC trap | NA | Cetre-Sossah (2010) |
|  | Corse | 2000 | 12 | October, November, December, January, February, March, April, May, June, July, August, September | Winter,  Spring,  Summer,  Autumn | NA | OVI trap | NA | Delécolle et al. (2002) |
|  | Ain, Allier, Alpes-de-Haute-Provence, Ardèche, Ardennes, Ariège, Aube, Aveyron, Bas-Rhin, Calvados, Cantal, Charente, Charente-Maritime, Cher, Corrèze, Corse, Côte-d'Or, Côtes-d’Armor, Creuse, Deux-Sèvres, Dordogne, Doubs, Drôme, Eure, Eure-et-Loir, Finistère, Gers, Gironde, Haute-Garonne, Haute-Loire, Haute-Marne, Hautes-Alpes, Haute-Saône, Haute-Savoie, Haute-Vienne, Haut-Rhin, Hérault, Ille-et-Vilaine, Indre, Indre, Indre-et-Loire, Isère, Jura, Landes, Loire, Loire-Atlantique, Loiret, Loir-et-Cher, Lot, Maine-et-Loire, Manche, Marne, Mayenne, Meuse, Morbihan, Nièvre, Nord, Oise, Orne, Puy-de-Dôme, Pyrénées-Atlantiques, Pyrénées-Orientales, Rhône, Saône-et-Loire, Sarthe, Seine-et-Marne, Seine-Maritime, Somme, Tarn, Tarn-et-Garonne, Territoire-de-Belfort, Val-d’Oise, Var, Vendée, Vienne, Vosges, Yonne, Yvelines | 2010 | 12 | January, February, March, April, May, June, July, August, September, October, November, December | Winter,  Spring,  Summer,  Autumn | NA | OVI trap | NA | Garros (2022) |
|  | Bas-Rhin, Vosges, Charente-Maritime, Jura, Seine-et-Marne, Haute-Saône, Ardennes | NA | NA | NA | NA | NA | NA | NA | Kremer (1965) |
|  | Corse | 1964, 1970 | 2 | July | Summer | 900-1080 | CDC trap,  Soil sampling | NA | Kremer et al. (1971) |
|  | Bas-Rhin, Haut-Rhin | 1976 | 8 | March, April, May, June, July, August, September, October | Spring,  Summer,  Autumn | NA | Larvae capture | NA | Kremer et al. (1978) |
|  | Doubs, Corse | 2009 | 2 | April, June | Spring | NA | OVI trap | NA | Mathieu (2011) |
|  | Doubs, Corse | NA | NA | NA | NA | NA | OVI trap | NA | Mathieu et al. (2011) |
|  | Corse | 2002-2009 | 120 | January, February, March, April, May, June, July, August, September, October, November, December | Winter,  Spring,  Summer,  Autumn | NA | OVI trap | NA | Mehlhorn (2012) |
|  | Nord, Aisne, Ardennes, Meuse, Meurthe-et-Moselle, Moselle | 2006 | 5 | August, September, October, November, December | Summer,  Autumn | NA | OVI trap | NA | Meiswinkel et al. (2007) |
|  | Ardennes | 2009-2010 | 18 | January, February, March, April, May, June, July, August, September, October, November, December | Winter,  Spring,  Summer,  Autumn | 271, 243 | CDC trap | NA | Ninio (2011) |
|  | Ardennes | 2009-2010 | 26 | January, February, March, April, May, June, July, August, September, October, November, December | Winter,  Spring,  Summer,  Autumn | NA | UV CDC trap,  Soil sampling | NA | Ninio (2011) |
|  | Var | 2002 | 8 | April, May, June, July, August, September, October, November | Spring,  Summer,  Autumn | NA | OVI trap | NA | Perrin et al. (2006) |
|  | Bas-Rhin | 1977-1978 | 17 | April, May, June, July, August, September, October, November, December, January, February, March | Spring,  Summer,  Autumn,  Winter | 150 | CDC trap,  Soil sampling | NA | Rieb (1987) |
|  | Corse | 1971 | NA | NA | NA | NA | NA | NA | Zientara et al. (2000) |
|  | Corse | 2000 | 0.13 | October | Autumn | NA | OVI trap | NA | Zientara et al. (2000) |
| Nubeculosus  Group | Indre-et-Loire | 2010 | 5 | April, May, June, September, October | Spring,  Summer,  Autumn | NA | OVI trap | NA | Viennet et al. (2012) |
|  | Saône-et-Loire, Hautes-Alpes, Hérault, Hautes-Pyrénées | 2008-2012 | 3 | June, July, August | Summer | 215-1500 | OVI trap | NA | Rossi et al. (2019) |
|  | Corse | 2009 | 12 | January, February, March, April, May, June, July, August, September, October, November, December | Winter,  Spring,  Summer,  Autumn | NA | OVI trap | NA | Balenghien et al. (2010) |
|  | Hérault, Loire-Atlantique, Vaucluse | 2004 | 8 | April, May, June, July, August, September, October, November | NA | NA | UV CDC trap | NA | Cetre-Sossah (2010) |
|  | Corse | 2000 | 12 | October, November, December, January, February, March, April, May, June, July, August, September | Winter,  Spring,  Summer,  Autumn | NA | OVI trap | NA | Delécolle et al. (2002) |
|  | Ain, Allier, Alpes-de-Haute-Provence, Hautes-Alpes, Aube, Calvados, Cantal, Charente, Charente-Maritime, Cher, Corrèze, Creuse, Dordogne, Doubs, Gironde, Hérault, Indre, Jura, Landes, Loir-et-Cher, Loire, Loire-Atlantique, Maine-et-Loire, Manche, Marne, Haute-Marne, Moselle, Nièvre, Nord, Oise, Orne, Pyrénées-Atlantiques, Bas-Rhin, Haut-Rhin, Haute-Saône, Saône-et-Loire, Sarthe, Haute-Savoie, Seine-Maritime, Seine-et-Marne, Yvelines, Deux-Sèvres, Somme, Tarn-et-Garonne, Vendée, Vienne, Vosges, Yonne, Territoire-de-Belfort, Ardennes, Eure, Corse, Gers, Loiret, Mayenne, Haute-Vienne, Indre-et-Loire | 2010 | 12 | January, February, March, April, May, June, July, August, September, October, November, December | Winter,  Spring,  Summer,  Autumn | NA | OVI trap | NA | Garros (2022) |
|  | Moselle | 1995 | NA | May | Spring | NA | NA | NA | INPN (2023) |
|  | Moselle, Manche, Bas-Rhin, Seine-et-Marne | NA | NA | NA | NA | NA | NA | *Onchocerca*  *cervicalis,*  *Onchocerca*  *reticulata* | Kremer (1965) |
|  | Bas-Rhin, Haut-Rhin | 1976 | 8 | March, April, May, June, July, August, September, October | Spring,  Summer,  Autumn | NA | Larvae capture | NA | Kremer et al. (1978) |
|  | Nord, Corse, Marne | 2009 | 2 | April, June | Spring | NA | OVI trap | NA | Mathieu (2011) |
|  | Nord, Corse, Marne | NA | NA | NA | NA | NA | OVI trap | NA | Mathieu et al. (2011) |
|  | Corse | 2002-2009 | 120 | January, February, March, April, May, June, July, August, September, October, November, December | Winter,  Spring,  Summer,  Autumn | NA | OVI trap | NA | Mehlhorn (2012) |
|  | Nord, Aisne, Ardennes, Meuse, Meurthe-et-Moselle, Moselle | 2006 | 5 | August, September, October, November, December | Summer,  Autumn | NA | OVI trap | NA | Meiswinkel et al. (2007) |
|  | Ardennes | 2009-2010 | 18 | January, February, March, April, May, June, July, August, September, October, November, December | Winter,  Spring,  Summer,  Autumn | 271, 243 | CDC trap | NA | Ninio (2011) |
|  | Ardennes | 2009-2010 | 26 | January, February, March, April, May, June, July, August, September, October, November, December | Winter,  Spring,  Summer,  Autumn | NA | UV CDC trap,  Soil sampling | NA | Ninio (2011) |
|  | Hérault | 2002 | 8 | April, May, June, July, August, September, October, November | Spring,  Summer,  Autumn | NA | OVI trap | NA | Perrin et al. (2006) |
|  | Hérault, Gard, Bouches-du-Rhône | 1953-1966 | NA | January, February, March, April, May, June, July, August, September, October, November, December | Winter,  Spring,  Summer,  Autumn | NA | Manual capture,  Larvae capture,  CDC trap,  Malaise trap | *Onchorerca*  *reticulata* | Rageau et al. (1967) |
|  | Moselle, Gard | 1995, 2009 | NA | May | Spring | NA | NA | NA | GBIF (2023) |
| Obsoletus  Group | Ain, Allier, Ardèche, Aube, Cantal, Côte-d'Or, Doubs, Drôme, Eure, Eure-et-Loir, Indre-et-Loire, Jura, Loir-et-Cher, Maine-et-Loire, Manche, Marne, Haute-Marne, Morbihan, Moselle, Nièvre, Nord, Orne, Pas-de-Calais, Bas-Rhin, Haut-Rhin, Haute-Saône, Saône-et-Loire, Sarthe, Savoie, Haute-Savoie, Seine-et-Marne, Yvelines, Somme, Vosges, Yonne, Val-d'Oise | 2011 | 1 | April | Spring | NA | OVI trap | NA | Mignotte et al. (2021) |
|  | Indre-et-Loire | 2009 | 1 | July, August | Summer | 120 | Cattle bait trap,  OVI trap | NA | Viennet et al. (2013) |
|  | Gers, Ille-et-Vilaine, Cher | 2009 | 5 | February, March, April, May, June | Winter,  Spring | NA | OVI trap | NA | Garros et al. (2011) |
|  | Ardennes, Marne, Deux-Sèvres | 2008-2009 | NA | NA | NA | NA | UV CDC trap,  CDC trap | NA | Ninio et al. (2011a) |
|  | Ardennes | 2006 | 4 | September, October, November, December | Autumn | NA | OVI trap | NA | Baldet et al. (2008) |
|  | Alpes-Maritimes, Var, Bouches-du-Rhône | 2002 | 8 | April, May, June, July, August, September, October, November | Spring,  Summer,  Autumn | NA | UV CDC trap | NA | Cêtre-Sossah et al. (2004) |
|  | Indre-et-Loire | 2010 | 5 | April, May, June, September, October | Spring,  Summer,  Autumn | NA | OVI trap | NA | Viennet et al. (2012) |
|  | Corse, Pyrénées-Orientales, Aude, Hérault, Gard, Bouches-du-Rhône, Var, Alpes-Maritimes | 2002-2003 | 38 | February, March, April, May, June, July, August, September, October, November, December | Winter,  Spring,  Summer,  Autumn | NA | UV CDC trap | NA | Baldet et al. (2005) |
|  | Vosges | 1981 | NA | NA | NA | NA | Larvae capture | NA | Waller et al. (1982) |
|  | Var, Somme, Loire, Corrèze, Loire-Atlantique | 2016 | NA | NA | NA | 10-370 | OVI trap | NA | Mignotte et al. (2020) |
|  | Ardennes | 2010 | 2 | August, September | Summer | NA | Emergence trap | NA | Ninio et al. (2011b) |
|  | Bas-Rhin, Saône-et-Loire, Hautes-Alpes, Hérault, Hautes-Pyrénées | 2008-2012 | 3 | June, July, August | Summer | 215-1500 | OVI trap | NA | Rossi et al. (2019) |
|  | Aisne, Gard | 2008 | 3 | July, August, September | Summer | 110, 200 | UV CDC trap,  CDC trap | NA | Augot et al. (2010) |
|  | Corse, Alpes-Maritimes, Var, Bouches-du-Rhône, Gard, Hérault, Aude, Pyrénées-Orientales | 2002 | 11 | February, March, April, May, June, July, August, September, October, November, December | Winter,  Spring,  Summer,  Autumn | NA | OVI trap | NA | Baldet et al. (2004) |
|  | Corse | 2009 | 12 | January, February, March, April, May, June, July, August, September, October, November, December | Winter,  Spring,  Summer,  Autumn | NA | OVI trap | NA | Balenghien et al. (2010) |
|  | Corse | 2012 | 12 | January, February, March, April, May, June, July, August, September, October, November, December | Winter,  Spring,  Summer,  Autumn | NA | OVI trap | NA | Balenghien et al. (2013) |
|  | Corse | 2013 | 12 | January, February, March, April, May, June, July, August, September, October, November, December | Winter,  Spring,  Autumn,  Summer | NA | OVI trap | NA | Balenghien et al. (2014a) |
|  | Corse | 2014 | 12 | January, February, March, April, May, June, July, August, September, October, November, December | Winter,  Spring,  Summer,  Autumn | NA | OVI trap | NA | Balenghien et al. (2014b) |
|  | Alpes-Maritimes, Bouches-du-Rhône, Gard, Haute-Savoie, Hérault, Loire-Atlantique, Pyrénées-Orientales, Var, Vaucluse | 2004 | 8 | April, May, June, July, August, September, October, November | NA | NA | UV CDC trap | NA | Cetre-Sossah (2010) |
|  | Pyrénées-Orientales, Var, Vaucluse, Alpes-Maritimes | 2002-2005 | 5 | May, June, July, August, October | Summer,  Autumn | NA | UV CDC trap | NA | Cetre-Sossah (2010) |
|  | All France | 2007-2013 | 12 | January, February, March, April, May, June, July, August, September, October, November, December | NA | NA | OVI trap | NA | Cuéllar et al. (2018) |
|  | Corse | 2000 | 12 | October, November, December, January, February, March, April, May, June, July, August, September | Winter,  Spring,  Summer,  Autumn | NA | OVI trap | NA | Delécolle et al. (2002) |
|  | All France | NA | NA | NA | NA | NA | NA | NA | ECDC (2022a) |
|  | Ain, Aisne, Allier, Alpes-de-Haute-Provence, Alpes-Maritimes, Ardèche, Ardennes, Ariège, Aube, Aude, Aveyron, Bas-Rhin, Bouches-du-Rhône, Calvados, Cantal, Charente, Charente-Maritime, Cher, Corrèze, Corse, Côte-d'Or, Côtes-d’Armor, Creuse, Deux-Sèvres, Dordogne, Doubs, Drôme, Essonne, Eure, Eure-et-Loir, Finistère, Gard, Gers, Gironde, Haute-Garonne, Haute-Loire, Haute-Marne, Hautes-Alpes, Haute-Saône, Haute-Savoie, Hautes-Pyrénées, Haute-Vienne, Haut-Rhin, Hauts-de-Seine, Hauts-de-Seine, Hérault, Ille-et-Vilaine, Indre, Indre-et-Loire, Isère, Jura, Landes, Loire, Loire-Atlantique, Loiret, Loir-et-Cher, Lot, Lot-et-Garonne, Lozère, Maine-et-Loire, Manche, Marne, Mayenne, Meurthe-et-Moselle, Meuse, Morbihan, Moselle, Nièvre, Nord, Oise, Orne, Pas-de-Calais, Puy-de-Dôme, Pyrénées-Atlantiques, Pyrénées-Orientales, Rhône, Saône-et-Loire, Sarthe, Savoie, Seine-et-Marne, Seine-Maritime, Somme, Tarn, Tarn-et-Garonne, Territoire-de-Belfort, Val-d’Oise, Var, Vaucluse, Vendée, Vienne, Vosges, Yonne, Yvelines | 2010 | 12 | January, February, March, April, May, June, July, August, September, October, November, December | Winter,  Spring,  Summer,  Autumn | NA | OVI trap | NA | Garros (2022) |
|  | Aisne, Gard | 2008-2011 | NA | NA | NA | 114-250 | UV CDC trap | NA | Hajd Henni et al. (2014) |
|  | Moselle | NA | NA | NA | NA | NA | NA | NA | Kieffer (1925) |
|  | Calvados, Landes | 2010 | 0.13 | April, July, November | Spring,  Summer,  Autumn | NA | OVI trap | NA | Kluiters et al. (2016) |
|  | Vosges, Gironde, Rhône, Bas-Rhin, Haut-Rhin, Savoie, Seine-et-Marne, Pyrénées-Orientales, Puy-de-Dôme, Corse | NA | NA | NA | NA | NA | NA | NA | Kremer (1965) |
|  | Corse | 1964, 1970 | 2 | July | Summer | 900-1080 | CDC trap,  Soil sampling | NA | Kremer  1971 Kremer et al. (1971) |
|  | Aisne, Aisne, Alpes-Maritimes, Ardennes, Aube, Bas-Rhin, Calvados, Charente, Corse, Côte-d'Or, Doubs, Drôme, Haute-Saône, Haut-Rhin, Jura, Marne, Meuse, Moselle, Nièvre, Nord, Oise, Pas-de-Calais, Saône-et-Loire, Seine-et-Marne, Somme, Territoire de Belfort, Val-de-Marne, Val-d'Oise, Var, Vosges, Yonne, Yvelines | 2009 | 2 | April, June | Spring | NA | OVI trap | NA | Mathieu (2011) |
|  | Ardennes, Corse, Nord, Pas-de-Calais, Somme | 2004-2006, 2008-2009 | NA | April, May, June, July, August, September | Spring,  Summer | NA | OVI trap | NA | Mathieu (2011) |
|  | All France | 2004 | NA | NA | NA | NA | NA | Bluetongue  Virus | Mathieu (2011) |
|  | Doubs, Aube, Jura, Nord, Pas-de-Calais, Somme, Ardennes, Yvelines, Territoire-de-Belfort, Bas-Rhin, Seine-et-Marne, Oise, Côte-d'Or, Corse, Vosges, Haut-Rhin, Savoie, Yonne, Var, Saône-et-Loire, Hautes-Alpes, Var, Meuse, Haute-Saône, Alpes-Maritimes, Nièvre, Bouches-du-Rhône, Vaucluse, Meurthe-et-Moselle, Pyrénées-Orientales, Marne, Hérault | NA | NA | NA | NA | NA | OVI trap | NA | Mathieu et al. (2011) |
|  | Corse | 2002-2009 | 120 | January, February, March, April, May, June, July, August, September, October, November, December | Winter,  Spring,  Summer,  Autumn | NA | OVI trap | NA | Mehlhorn (2012) |
|  | Nord, Aisne, Ardennes, Meuse, Meurthe-et-Moselle, Moselle | 2006 | 5 | August, September, October, November, December | Summer,  Autumn | NA | OVI trap | NA | Meiswinkel et al. (2007) |
|  | Doubs, Var, Loire-Atlantique, Loire, Gironde | 2016 | NA | NA | NA | NA | NA | NA | Mignotte (2020) |
|  | Ardennes | 2009-2010 | 18 | January, February, March, April, May, June, July, August, September, October, November, December | Winter,  Spring,  Summer,  Autumn | 271, 243 | CDC trap | NA | Ninio (2011) |
|  | Ardennes | 2009-2010 | 26 | January, February, March, April, May, June, July, August, September, October, November, December | Winter,  Spring,  Summer,  Autumn | NA | UV CDC trap,  Soil sampling | NA | Ninio (2011) |
|  | Ardennes | NA | NA | NA | NA | NA | NA | NA | Ninio (2011) |
|  | Pyrénées-Orientales, Var, Aude, Alpes-Maritimes | 2002 | 8 | April, May, June, July, August, September, October, November | Spring,  Summer,  Autumn | NA | OVI trap | NA | Perrin et al. (2006) |
|  | Corrèze | 2012-2013 | 4 | June, August, September | Spring,  Summer | NA | OVI trap | NA | Venail (2014) |
|  | Corrèze | 2012 | 2 | May, June | Spring | NA | OVI trap | NA | Venail (2014) |
|  | Corrèze | 2012-2013 | 6 | May, June, August, September | Spring,  Summer | NA | OVI trap | NA | Venail (2014) |
|  | Corse | 1971 | NA | NA | NA | NA | NA | NA | Zientara et al. (2000) |
|  | Corse | 2000 | 0.13 | October | Autumn | NA | OVI trap | NA | Zientara et al. (2000) |
|  | Corse | 2000-2001 | 4 | November, December, January, June | Winter | NA | OVI trap | NA | Zientara et al. (2001) |
|  | Aisne, Ardennes, Calvados, Corrèze, Corse, Gard, Loire, Loire-Atlantique, Nord, Pas-de-Calais, Seine-Maritime, Somme, Var, Yvelines | 2004-2005, 2009-2010, 2016 | NA | January, February, April, May, June, July, August | Winter,  Spring,  Summer | NA | NA | NA | GBIF (2023) |
| Pulicaris  Group | Indre-et-Loire | 2009 | 1 | July, August | Summer | 120 | Cattle bait trap,  OVI trap | NA | Viennet et al. (2013) |
|  | Gers, Ille-et-Vilaine, Cher | 2009 | 5 | February, March, April, May, June | Winter,  Spring | NA | OVI trap | NA | Garros et al. (2011) |
|  | Ardennes, Marne, Deux-Sèvres | 2008-2009 | NA | NA | NA | NA | UV CDC trap,  CDC trap | NA | Ninio et al. (2011a) |
|  | Ardennes | 2006 | 4 | September, October, November, December | Autumn | NA | OVI trap | NA | Baldet et al. (2008) |
|  | Alpes-Maritimes, Var, Bouches-du-Rhône | 2002 | 8 | April, May, June, July, August, September, October, November | Spring,  Summer,  Autumn | NA | UV CDC trap | NA | Cêtre-Sossah et al. (2004) |
|  | Indre-et-Loire | 2010 | 5 | April, May, June, September, October | Spring,  Summer,  Autumn | NA | OVI trap | NA | Viennet et al. (2012) |
|  | Corse, Pyrénées-Orientales, Aude, Hérault, Gard, Bouches-du-Rhône, Var, Alpes-Maritimes | 2002-2003 | 38 | February, March, April, May, June, July, August, September, October, November, December | Winter,  Spring,  Summer,  Autumn | NA | UV CDC trap | NA | Baldet et al. (2005) |
|  | Bas-Rhin, Saône-et-Loire, Hautes-Alpes, Hérault, Hautes-Pyrénées | 2008-2012 | 3 | June, July, August | Summer | 215-1500 | OVI trap | NA | Rossi et al. (2019) |
|  | Corse, Alpes-Maritimes, Var, Bouches-du-Rhône, Gard, Hérault, Aude, Pyrénées-Orientales | 2002 | 11 | February, March, April, May, June, July, August, September, October, November, December | Winter,  Spring,  Summer,  Autumn | NA | OVI trap | NA | Baldet et al. (2004) |
|  | Corse | 2009 | 12 | January, February, March, April, May, June, July, August, September, October, November, December | Winter,  Spring,  Summer,  Autumn | NA | OVI trap | NA | Balenghien et al. (2010) |
|  | Corse | 2013 | 12 | January, February, March, April, May, June, July, August, September, October, November, December | Winter,  Spring,  Autumn,  Summer | NA | OVI trap | NA | Balenghien et al. (2014a) |
|  | Corse | 2014 | 12 | January, February, March, April, May, June, July, August, September, October, November, December | Winter,  Spring,  Summer,  Autumn | NA | OVI trap | NA | Balenghien et al. (2014b) |
|  | Alpes-Maritimes, Bouches-du-Rhône, Haute-Savoie, Loire-Atlantique, Pyrénées-Orientales, Var, Vaucluse, Gard | 2004 | 8 | April, May, June, July, August, September, October, November | NA | NA | UV CDC trap | NA | Cetre-Sossah (2010) |
|  | All France | 2007-2013 | 12 | January, February, March, April, May, June, July, August, September, October, November, December | NA | NA | OVI trap | NA | Cuéllar et al. (2018) |
|  | Corse | 2000 | 12 | October, November, December, January, February, March, April, May, June, July, August, September | Winter,  Spring,  Summer,  Autumn | NA | OVI trap | NA | Delécolle et al. (2002) |
|  | All France | NA | NA | NA | NA | NA | NA | NA | ECDC (2022a) |
|  | Ain, Ain, Aisne, Allier, Alpes-de-Haute-Provence, Alpes-Maritimes, Ardèche, Ardennes, Ariège, Aube, Aude, Aveyron, Bas-Rhin, Bouches-du-Rhône, Calvados, Cantal, Charente, Charente-Maritime, Cher, Corrèze, Corse, Côte-d'Or, Côtes-d’Armor, Creuse, Deux-Sèvres, Dordogne, Doubs, Drôme, Eure, Eure-et-Loir, Finistère, Gard, Gers, Gironde, Haute-Garonne, Haute-Loire, Haute-Marne, Hautes-Alpes, Haute-Saône, Haute-Savoie, Hautes-Pyrénées, Haute-Vienne, Haut-Rhin, Hérault, Ille-et-Vilaine, Indre, Indre-et-Loire, Isère, Jura, Landes, Loire, Loire-Atlantique, Loiret, Loir-et-Cher, Lot, Lot-et-Garonne, Lozère, Maine-et-Loire, Manche, Marne, Mayenne, Meuse, Morbihan, Moselle, Nièvre, Nord, Oise, Orne, Pas-de-Calais, Puy-de-Dôme, Pyrénées-Atlantiques, Pyrénées-Orientales, Rhône, Saône-et-Loire, Sarthe, Savoie, Seine-et-Marne, Seine-Maritime, Somme, Tarn, Tarn-et-Garonne, Territoire-de-Belfort, Val-d’Oise, Var, Vaucluse, Vendée, Vienne, Vosges, Yonne, Yvelines | 2010 | 12 | January, February, March, April, May, June, July, August, September, October, November, December | Winter,  Spring,  Summer,  Autumn | NA | OVI trap | NA | Garros (2022) |
|  | Gironde, Rhône, Vosges, Bas-Rhin, Ardennes, Savoie, Gironde, Bouches-du-Rhône, Pyrénées-Orientales, Puy-de-Dôme, Corse | NA | NA | NA | NA | NA | NA | NA | Kremer (1965) |
|  | Corse | 1964, 1970 | 2 | July | Summer | 900-1080 | CDC trap,  Soil sampling | NA | Kremer et al. (1971) |
|  | Bas-Rhin, Haut-Rhin | 1976 | 8 | March, April, May, June, July, August, September, October | Spring,  Summer,  Autumn | NA | Larvae capture | NA | Kremer et al. (1978) |
|  | Corse, Vaucluse, Pyrénées-Orientales | 2009 | 2 | April, June | Spring | NA | OVI trap | NA | Mathieu (2011) |
|  | All France | 2004 | NA | NA | NA | NA | NA | Bluetongue  Virus | Mathieu (2011) |
|  | Corse, Vaucluse, Pyrénées-Orientales | NA | NA | NA | NA | NA | OVI trap | NA | Mathieu et al. (2011) |
|  | Corse | 2002-2009 | 120 | January, February, March, April, May, June, July, August, September, October, November, December | Winter,  Spring,  Summer,  Autumn | NA | OVI trap | NA | Mehlhorn (2012) |
|  | Nord, Aisne, Ardennes, Meuse, Meurthe-et-Moselle, Moselle | 2006 | 5 | August, September, October, November, December | Summer,  Autumn | NA | OVI trap | NA | Meiswinkel et al. (2007) |
|  | Ardennes | 2009-2010 | 18 | January, February, March, April, May, June, July, August, September, October, November, December | Winter,  Spring,  Summer,  Autumn | 271, 243 | CDC trap | NA | Ninio (2011) |
|  | Ardennes | 2009-2010 | 26 | January, February, March, April, May, June, July, August, September, October, November, December | Winter,  Spring,  Summer,  Autumn | NA | UV CDC trap,  Soil sampling | NA | Ninio (2011) |
|  | Ardennes | NA | NA | NA | NA | NA | NA | NA | Ninio (2011) |
|  | Aude, Pyrénées-Orientales | 2002 | 8 | April, May, June, July, August, September, October, November | Spring,  Summer,  Autumn | NA | OVI trap | NA | Perrin et al. (2006) |
|  | Hérault, Gard, Bouches-du-Rhône | 1953-1966 | NA | January, February, March, April, May, June, July, August, September, October, November, December | Winter,  Spring,  Summer,  Autumn | NA | Manual capture,  Larvae capture,  CDC trap,  Malaise trap | NA | Rageau et al. (1967) |
|  | Corse | 1971 | NA | NA | NA | NA | NA | NA | Zientara et al. (2000) |
|  | Corse | 2000 | 0.13 | October | Autumn | NA | OVI trap | NA | Zientara et al. (2000) |
|  | Côte-d'Or, Aisne | 2014-2015 | NA | April, May | Spring | NA | NA | NA | GBIF (2023) |
| Punctatus  Group | Indre-et-Loire | 2009 | 1 | July, August | Summer | 120 | Cattle bait trap,  OVI trap | NA | Viennet et al. (2013) |
|  | Gers, Ille-et-Vilaine, Cher | 2009 | 5 | February, March, April, May, June | Winter,  Spring | NA | OVI trap | NA | Garros et al. (2011) |
|  | Ardennes, Marne, Deux-Sèvres | 2008-2009 | NA | NA | NA | NA | UV CDC trap,  CDC trap | NA | Ninio et al. (2011a) |
|  | Ardennes | 2006 | 4 | September, October, November, December | Autumn | NA | OVI trap | NA | Baldet et al. (2008) |
|  | Alpes-Maritimes, Var, Bouches-du-Rhône | 2002 | 8 | April, May, June, July, August, September, October, November | Spring,  Summer,  Autumn | NA | UV CDC trap | NA | Cêtre-Sossah et al. (2004) |
|  | Indre-et-Loire | 2010 | 5 | April, May, June, September, October | Spring,  Summer,  Autumn | NA | OVI trap | NA | Viennet et al. (2012) |
|  | Corse, Pyrénées-Orientales, Aude, Hérault, Gard, Bouches-du-Rhône, Var, Alpes-Maritimes | 2002-2003 | 38 | February, March, April, May, June, July, August, September, October, November, December | Winter,  Spring,  Summer,  Autumn | NA | UV CDC trap | NA | Baldet et al. (2005) |
|  | Vosges | 1981 | NA | NA | NA | NA | Larvae capture | NA | Waller et al. (1982) |
|  | Bas-Rhin, Saône-et-Loire, Hautes-Alpes, Hérault, Hautes-Pyrénées | 2008-2012 | 3 | June, July, August | Summer | 215-1500 | OVI trap | NA | Rossi et al. (2019) |
|  | Corse, Alpes-Maritimes, Var, Bouches-du-Rhône, Gard, Hérault, Aude, Pyrénées-Orientales | 2002 | 11 | February, March, April, May, June, July, August, September, October, November, December | Winter,  Spring,  Summer,  Autumn | NA | OVI trap | NA | Baldet et al. (2004) |
|  | Corse | 2009 | 12 | January, February, March, April, May, June, July, August, September, October, November, December | Winter,  Spring,  Summer,  Autumn | NA | OVI trap | NA | Balenghien et al. (2010) |
|  | Corse | 2013 | 12 | January, February, March, April, May, June, July, August, September, October, November, December | Winter,  Spring,  Autumn,  Summer | NA | OVI trap | NA | Balenghien et al. (2014a) |
|  | Corse | 2014 | 12 | January, February, March, April, May, June, July, August, September, October, November, December | Winter,  Spring,  Summer,  Autumn | NA | OVI trap | NA | Balenghien et al. (2014b) |
|  | Alpes-Maritimes, Bouches-du-Rhône, Hérault, Loire-Atlantique, Pyrénées-Orientales, Var, Vaucluse, Gard, Hautes-Pyrénées | 2004 | 8 | April, May, June, July, August, September, October, November | NA | NA | UV CDC trap | NA | Cetre-Sossah (2010) |
|  | Corse | 2000 | 12 | October, November, December, January, February, March, April, May, June, July, August, September | Winter,  Spring,  Summer,  Autumn | NA | OVI trap | NA | Delécolle et al. (2002) |
|  | All France | NA | NA | NA | NA | NA | NA | NA | ECDC (2022a) |
|  | Ain, Aisne, Allier, Alpes-de-Haute-Provence, Ardèche, Ardennes, Ariège, Aube, Aude, Aveyron, Bas-Rhin, Bouches-du-Rhône, Calvados, Cantal, Charente, Charente-Maritime, Cher, Corrèze, Corse, Côte-d'Or, Côtes-d’Armor, Creuse, Deux-Sèvres, Dordogne, Doubs, Drôme, Essonne, Eure, Eure-et-Loir, Finistère, Gard, Gers, Gironde, Haute-Garonne, Haute-Loire, Haute-Marne, Hautes-Alpes, Haute-Saône, Haute-Savoie, Hautes-Pyrénées, Haute-Vienne, Haut-Rhin, Hérault, Ille-et-Vilaine, Indre, Indre-et-Loire, Isère, Jura, Landes, Loire, Loire-Atlantique, Loiret, Loir-et-Cher, Lot, Lot-et-Garonne, Lozère, Maine-et-Loire, Manche, Marne, Mayenne, Meurthe-et-Moselle, Meuse, Morbihan, Moselle, Nièvre, Nord, Oise, Orne, Pas-de-Calais, Puy-de-Dôme, Pyrénées-Atlantiques, Pyrénées-Orientales, Rhône, Saône-et-Loire, Sarthe, Savoie, Seine-et-Marne, Seine-Maritime, Somme, Tarn, Tarn-et-Garonne, Territoire-de-Belfort, Val-d’Oise, Var, Vaucluse, Vendée, Vienne, Vosges, Yonne, Yvelines | 2010 | 12 | January, February, March, April, May, June, July, August, September, October, November, December | Winter,  Spring,  Summer,  Autumn | NA | OVI trap | NA | Garros (2022) |
|  | Moselle | 1995 | NA | May | Spring | NA | NA | NA | INPN (2023) |
|  | Seine-et-Marne, Jura, Ardennes, Charente-Maritime, Hérault, Bas-Rhin, Vosges, Puy-de-Dôme, Moselle, Rhône, Var | NA | NA | NA | NA | NA | NA | NA | Kremer (1965) |
|  | Bas-Rhin, Haut-Rhin | 1976 | 8 | March, April, May, June, July, August, September, October | Spring,  Summer,  Autumn | NA | Larvae capture | NA | Kremer et al. (1978) |
|  | Corse | 2009 | 2 | April, June | Spring | NA | OVI trap | NA | Mathieu (2011) |
|  | Corse | NA | NA | NA | NA | NA | OVI trap | NA | Mathieu et al. (2011) |
|  | Corse | 2002-2009 | 120 | January, February, March, April, May, June, July, August, September, October, November, December | Winter,  Spring,  Summer,  Autumn | NA | OVI trap | NA | Mehlhorn (2012) |
|  | Nord, Aisne, Ardennes, Meuse, Meurthe-et-Moselle, Moselle | 2006 | 5 | August, September, October, November, December | Summer,  Autumn | NA | OVI trap | NA | Meiswinkel et al. (2007) |
|  | Ardennes | 2009-2010 | 18 | January, February, March, April, May, June, July, August, September, October, November, December | Winter,  Spring,  Summer,  Autumn | 271, 243 | CDC trap | NA | Ninio (2011) |
|  | Ardennes | 2009-2010 | 26 | January, February, March, April, May, June, July, August, September, October, November, December | Winter,  Spring,  Summer,  Autumn | NA | UV CDC trap,  Soil sampling | NA | Ninio (2011) |
|  | Bouches-du-Rhône, Bas-Rhin | 2002 | 8 | April, May, June, July, August, September, October, November | Spring,  Summer,  Autumn | NA | OVI trap | NA | Perrin et al. (2006) |
|  | Corse | 2000 | 0.13 | October | Autumn | NA | OVI trap | NA | Zientara et al. (2000) |
|  | Corse | 2000-2001 | 4 | November, December, January, June | Winter | NA | OVI trap | NA | Zientara et al. (2001) |
|  | Corse, Moselle | 1995 | NA | May | Spring | NA | NA | NA | GBIF (2023) |
| *Palpomyia*  *lineata* | Bas-Rhin | 1981-1982 | NA | NA | NA | NA | NA | NA | Arnold et al. (1982) |
|  | Pyrénées-Orientales | 2001, 2004-2005 | NA | January | Winter | NA | NA | NA | INPN (2023) |
|  | Pyrénées-Orientales | 2001, 2004-2005 | NA | January | Winter | NA | NA | NA | GBIF (2023) |

Supplementary Table S10. Captures of ticks in cattle farms in France between 1888 and 2023 (NA: data not provided).

| Species | Capture | | | | | | | Pathogen | Reference |
| --- | --- | --- | --- | --- | --- | --- | --- | --- | --- |
|  | Department | Year | Study  duration  (month) | Month | Season | Altitude  (m) | Trapping  method |  |  |
| *Amblyomma*  *variegatum* | Corse | 2018 | 2 | July, August | Summer | 180 | Manual  capture | *Rickettsia*  *africae* | Cicculli et al. (2019b) |
|  | Dordogne | 1930-1985 | NA | March, April, May, June, July, August, September, October, November, December, January, February | Spring,  Summer,  Autumn,  Winter | NA | Manual  capture | NA | Lamontellerie (1965) |
| *Argas*  *reflexus* | Hautes-Alpes, Dordogne | 1950, 2009 | NA | January, December | Winter | NA | NA | NA | INPN (2023) |
|  | Vendée, Charente, Gironde | 1930-1985 | NA | March, April, May, June, July, August, September, October, November, December, January, February | Spring,  Summer,  Autumn,  Winter | NA | Manual  capture | NA | Lamontellerie (1965) |
|  | Bouches-du-Rhône | NA | NA | NA | NA | NA | NA | NA | Rageau et al. (1970a) |
|  | Hautes-Alpes, Drôme, Dordogne, Rhône, Bouches-du-Rhône | 1950, 2009, 2012, 2014 | NA | January, May, August, December | Winter,  Spring,  Summer | NA | NA | NA | GBIF (2023) |
| *Argas*  *vespertilionis* | Gironde | 1930-1985 | NA | March, April, May, June, July, August, September, October, November, December, January, February | Spring,  Summer,  Autumn,  Winter | NA | Manual  capture | NA | Lamontellerie (1965) |
|  | Pyrénées-Orientales | 1950, 1952 | NA | April, August, September, November | Spring,  Summer,  Autumn | NA | Manual  capture | NA | Theodorides (1954) |
|  | Creuse | 2021 | NA | May | Spring | NA | NA | NA | GBIF (2023) |
| *Dermacentor*  *marginatus* | Corse | 2014-2015 | 12 | May, June, July, August, September, October, November, December, January, February, March, April | Spring,  Summer,  Autumn,  Winter | NA | Manual  capture | NA | Grech-Angelini et al. (2016b) |
|  | Loire-Atlantique | 2006-2007 | 2 | April, May, June | Spring | NA | Flagging | *Babesia divergens* | Agoulon et al. (2012a) |
|  | Bas-Rhin, Haut-Rhin, Vosges, Morbihan, Côtes-d'Armor, Ille-et-Vilaine, Mayenne, Orne, Sarthe, Maine-et-Loire, Eure-et-Loir, Loir-et-Cher, Indre-et-Loire, Indre, Cher, Loiret, Vienne, Deux-Sèvres, Vendée, Charente, Charente-Maritime, Gironde, Dordogne, Nièvre, Yonne, Côte-d'Or, Saône-et-Loire, Puy-de-Dôme, Allier, Loire, Rhône, Ain, Cantal, Aveyron, Haute-Loire, Ardèche, Isère, Drôme, Hautes-Alpes, Alpes-de-Haute-Provence, Alpes-Maritimes, Var, Bouches-du-Rhône, Vaucluse, Gard, Hérault, Aude, Pyrénées-Orientales, Ariège, Haute-Garonne, Tarn | 2005 | NA | NA | NA | NA | Manual  capture | NA | Chauvet et al. (2005) |
|  | Allier, Cher, Loire-Atlantique, Moselle, Var, Yonne | NA | NA | NA | NA | NA | Manual  capture | *Rickettsia conori* | Giroud et al. (1965) |
|  | Morbihan | 2010-2011 | 12 | April, May, June, July, August, September, October, November, December, January, February, March | Spring,  Summer,  Autumn,  Winter | 70 | Flagging | *Borrelia* sp.,  *Rickettsia slovaca*,  *Babesia* sp. | Michelet et al. (2016) |
|  | Corse | 2014-2015 | 12 | May, June, July, August, September, October, November, December, January, February, March, April | Spring,  Summer,  Autumn,  Winter | 40-533 | Manual  capture | *Rickettsia slovaca*,  *Anaplasma phagocytophilum*,  *Bartonella henselae* | Grech‐Angelini et al. (2020) |
|  | Corse | 2014-2015 | 12 | May, June, July, August, September, October, November, December, January, February, March, April | Spring,  Summer,  Autumn,  Winter | 40-533 | Manual  capture | *Babesia caballi*,  *Anaplasma ovis*,  *Theileria buffeli* | Grech-Angelini (2017) |
|  | Bas-Rhin, Haut-Rhin, Vosges, Morbihan, Côtes-d'Armor, Ille-et-Vilaine, Mayenne, Orne, Sarthe, Maine-et-Loire, Eure-et-Loir, Loir-et-Cher, Indre-et-Loire, Indre, Cher, Loiret, Vienne, Deux-Sèvres, Vendée, Charente, Charente-Maritime, Gironde, Dordogne, Nièvre, Yonne, Côte-d'Or, Saône-et-Loire, Puy-de-Dôme, Allier, Loire, Rhône, Ain, Cantal, Aveyron, Haute-Loire, Ardèche, Isère, Drôme, Hautes-Alpes, Alpes-de-Haute-Provence, Alpes-Maritimes, Var, Bouches-du-Rhône, Vaucluse, Gard, Hérault, Aude, Pyrénées-Orientales, Ariège, Haute-Garonne, Tarn | 2005 | NA | NA | NA | NA | NA | *Anaplasma marginale* | Agoulon et al. (2012b) |
|  | Bas-Rhin, Haut-Rhin, Vosges, Morbihan, Côtes-d'Armor, Ille-et-Vilaine, Mayenne, Orne, Sarthe, Maine-et-Loire, Eure-et-Loir, Loir-et-Cher, Indre-et-Loire, Indre, Cher, Loiret, Vienne, Deux-Sèvres, Vendée, Charente, Charente-Maritime, Gironde, Dordogne, Nièvre, Yonne, Côte-d'Or, Saône-et-Loire, Puy-de-Dôme, Allier, Loire, Rhône, Ain, Cantal, Aveyron, Haute-Loire, Ardèche, Isère, Drôme, Hautes-Alpes, Alpes-de-Haute-Provence, Alpes-Maritimes, Var, Bouches-du-Rhône, Vaucluse, Gard, Hérault, Aude, Pyrénées-Orientales, Ariège, Haute-Garonne, Tarn | 2005 | NA | NA | NA | NA | NA | NA | L'Hostis et al. (2007) |
|  | Saône-et-Loire | 1996 | 2 | April, May | Spring | NA | Manual  capture | NA | Levasseur (1997) |
|  | Saône-et-Loire, Rhône, Loire, Ardèche, Gard, Bouches-du-Rhône | 1984-1986 | 30 | January, February, March, April, May, June, July, August, September, October, November, December | Spring,  Autumn,  Summer,  Winter | NA | Flagging,  Manual  capture | NA | Gilot et al. (1989) |
|  | Aveyron, Lot | 1991 | 1 | March | Spring | NA | Flagging | *Rickettsia slovaca* | Beati et al. (1993) |
|  | Marne, Moselle, Finistère, Vienne, Gironde, Rhône, Alpes-Maritimes, Bouches-du-Rhône, Gard | 2008-2009 | 12 | January, February, March, April, May, June, July, August, September, October, November, December | Winter,  Spring,  Summer,  Autumn | NA | Flagging | NA | Beugnet et al. (2009) |
|  | Loire-Atlantique, Deux-Sèvres, Yonne, Côte-d’Or, Saône-et-Loire, Aveyron, Isère, Bouches-du-Rhône | 2009 | 0.5 | April, May, September, October | Spring,  Autumn | NA | Flagging | *Anaplasma phagocytophilum*,  *Anaplasma marginale*,  *Bartonella* spp.,  *Coxiella burnetii*,  *Babesia* sp.,  *Theileria* sp.,  *Francisella philomiragia* | Bonnet et al. (2013) |
|  | Ain, Charente-Maritime, Côte-d'Or, Deux-Sèvres, Haute-Vienne, Indre, Indre-et-Loire, Isère, Nièvre, Bas-Rhin, Rhône, Vienne, Puy-de-Dôme | 1945-2002 | NA | January, February, March, April, May, June, July, August, September, October, November, December | Winter,  Spring,  Summer,  Autumn | NA | Flagging,  Manual  capture | NA | Chauvet (2004) |
|  | Loire | 2001-2002 | 14 | March, April, May, June, July, August, September, October, November, December, January, February | Spring,  Summer,  Winter,  Autumn | 370-1000 | Manual  capture | NA | Devos (2002) |
|  | Bas-Rhin, Dordogne, Gironde, Landes, Lot-Et-Garonne, Pyrénées-Atlantiques, Allier, Cantal, Puy-De-Dôme, Côte-d'Or, Nièvre, Saône-et-Loire, Yonne, Ille-Et-Vilaine, Morbihan, Cher, Eure-Et-Loir, Indre, Indre-Et-Loire, Loiret, Aube, Corse, Jura, Essonne, Hauts-De-Seine, Seine-Et-Marne, Seine-Saint-Denis, Val-D'Oise, Val-De-Marne, Paris, Yvelines, Aude, Gard, Hérault, Pyrénées-Orientales, Creuse, Haute-Vienne, Moselle, Ariège, Aveyron, Gers, Haute-Garonne, Hautes-Pyrénées, Lot, Tarn, Tarn-Et-Garonne, Loire-Atlantique, Maine-et-Loire, Mayenne, Vendée, Oise, Charente, Charente-Maritime, Deux-Sèvres, Vienne, Alpes-De-Haute-Provence, Alpes-Maritimes, Bouches-Du-Rhône, Hautes-Alpes, Var, Vaucluse, Ain, Ardèche, Drôme, Haute-Savoie, Isère, Loire, Rhône, Savoie | 1970-2000 | NA | NA | NA | NA | NA | NA | EFSA (2010) |
|  | Charente-Maritime, Charente, Haute-Vienne, Gironde, Dordogne, Corrèze, Lot, Lot-et-Garonne, Landes, Pyrénées-Atlantiques, Gers, Hautes-Pyrénées, Ariège, Haute-Garonne, Tarn, Tarn-et-Garonne, Aude, Pyrénées-Orientales, Hérault, Aveyron, Lozère, Haute-Loire, Gard, Ardèche, Drôme, Hautes-Alpes, Alpes-de-Haute-Provence, Vaucluse, Bouches-du-Rhône, Var, Alpes-Maritimes, Isère | NA | NA | January, February, March, April, May, June, July, August, September, October, November, December | Winter,  Spring,  Summer,  Autumn | NA | NA | NA | François (2008) |
|  | Moselle, Meuse, Vosges | NA | NA | NA | NA | NA | NA | NA | George et al. (2002) |
|  | Drôme, Ardèche, Hautes-Alpes, Alpes-de-Haute-Provence, Vaucluse | 1971-1972 | 12 | October, November, December, January, February, March, April, May, June, July, August, September | Autumn,  Winter,  Spring,  Summer | NA | Flagging,  Manual  capture | NA | Gilot (1985) |
|  | Aveyron | NA | NA | NA | NA | NA | NA | NA | Gilot (1985) |
|  | Vaucluse | 1978-1980, 1986 | NA | NA | NA | NA | Flagging,  Manual  capture | NA | Gilot (1987) |
|  | Ain, Ardèche, Drôme, Isère, Loire, Rhône, Savoie, Haute-Savoie, Vaucluse, Landes | 1962-1972 | NA | NA | NA | NA | NA | NA | Gilot et al. (1982) |
|  | Isère, Ain | 1977 | 2 | May, June | Summer | NA | Flagging,  Manual  capture | NA | Gilot et al. (1979) |
|  | Isère, Ain | 1970-1973 | NA | January, February, March, April, May, June, July, August, September, October, November, December | Winter,  Spring,  Summer,  Autumn | NA | Flagging,  Manual  capture | NA | Gilot et al. (1975b) |
|  | Alpes-de-Haute-Provence, Alpes-Maritimes, Ardèche, Ariège, Bas-Rhin, Bouches-du-Rhône, Cher, Dordogne, Gard, Gironde, Hérault, Corse, Haute-Garonne, Hautes-Alpes, Haute-Savoie, Indre-et-Loire, Isère, Loire, Loir-et-Cher, Lot, Lozère, Maine-et-Loire, Morbihan, Saône-et-Loire, Sarthe, Seine-et-Marne, Var, Vaucluse, Vendée, Vienne | 2006, 2008-2014, 2016-2022 | NA | January, February, March, April, May, June, August, September, October, November, December | Winter,  Spring,  Summer,  Autumn | NA | NA | NA | INPN (2023) |
|  | Deux-Sèvres, Vienne, Charente-Maritime, Gironde, Haute-Vienne, Landes, Dordogne, Lot, Pyrénées-Atlantiques | 1930-1985 | NA | March, April, May, June, July, August, September, October, November, December, January, February | Spring,  Summer,  Autumn,  Winter | NA | Manual  capture | NA | Lamontellerie (1965) |
|  | Bouches-du-Rhône | 2008, 2010 | NA | NA | NA | NA | NA | *Anaplasma phagocytophilum* | Leblond et al. (2012) |
|  | All France | NA | NA | NA | NA | NA | NA | NA | Pérez-Eid (2007) |
|  | Ain, Allier, Alpes-Maritimes, Ardèche, Aube, Aveyron, Bouches-du-Rhône, Charente, Charente-Maritime, Cher, Corse, Côte-d’Or, Dordogne, Drôme, Haute-Garonne, Gers, Gironde, Hérault, Ille-et-Vilaine, Indre-et-Loire, Isère, Landes, Loire-Atlantique, Lot, Lot-et-Garonne, Maine-et-Loire, Moselle, Nièvre, Puy-de-Dôme, Pyrénées-Atlantiques, Pyrénées-Orientales, Rhône, Paris, Yvelines, Essonne, Hauts-de-Seine, Seine-Saint-Denis, Val-de-Marne, Val-d’Oise, Tarn, Tarn-et-Garonne, Var, Vienne, Haute-Vienne, Yonne | 1962-1972 | NA | NA | NA | NA | NA | *Coxiella burnetii*,  *Francisella tularensis*,  *Babesia bovis* | Rageau (1972) |
|  | Hérault, Gard, Bouches-du-Rhône | 1953-1966 | NA | January, February, March, April, May, June, July, August, September, October, November, December | Winter,  Spring,  Summer,  Autumn | NA | Flagging  Manual  capture | NA | Rageau et al. (1967) |
|  | Bouches-du-Rhône | NA | NA | NA | NA | NA | NA | NA | Rageau et al. (1970a) |
|  | Alpes-de-Haute-Provence, Rhône, Ain, Drôme, Loire, Ardèche | NA | NA | NA | NA | NA | NA | NA | Roman et al. (1973) |
|  | Ain, Allier, Alpes-de-Haute-Provence, Hautes-Alpes, Alpes-Maritimes, Ardèche, Ariège, Aube, Aude, Aveyron, Bouches-du-Rhône, Cantal, Charente, Charente-Maritime, Cher, Corrèze, Corse, Côte-d'Or, Creuse, Dordogne, Doubs, Drôme, Eure, Eure-et-Loir, Gard, Haute-Garonne, Gers, Gironde, Hérault, Ille-et-Vilaine, Indre, Indre-et-Loire, Isère, Jura, Landes, Loir-et-Cher, Loire, Haute-Loire, Loire-Atlantique, Loiret, Lot, Lot-et-Garonne, Lozère, Maine-et-Loire, Haute-Marne, Morbihan, Nièvre, Oise, Orne, Puy-de-Dôme, Pyrénées-Atlantiques, Hautes-Pyrénées, Pyrénées-Orientales, Bas-Rhin, Haut-Rhin, Rhône, Haute-Saône, Saône-et-Loire, Savoie, Haute-Savoie, Paris, Seine-et-Marne, Yvelines, Deux-Sèvres, Tarn, Tarn-et-Garonne, Var, Vaucluse, Vendée, Vienne, Haute-Vienne, Yonne, Territoire-de-Belfort, Essonne, Hauts-de-Seine, Seine-Saint-Denis, Val-de-Marne, Val-d'Oise | 2010-2015 | NA | NA | NA | NA | NA | NA | Rubel et al. (2016) |
|  | Pyrénées-Orientales | 1950, 1952 | NA | April, August, September, November | Spring,  Summer,  Autumn | NA | Manual  capture | NA | Theodorides (1954) |
|  | Ain, Alpes-de-Haute-Provence, Alpes-Maritimes, Ardèche, Ariège, Aude, Bas-Rhin, Bouches-du-Rhône, Cher, Corse, Dordogne, Gard, Gers, Gironde, Haute-Garonne, Hautes-Alpes, Haute-Saône, Haute-Savoie, Haut-Rhin, Hérault, Indre-et-Loire, Isère, Landes, Loire, Loir-et-Cher, Lot, Lozère, Maine-et-Loire, Morbihan, Pyrénées-Orientales, Rhône, Saône-et-Loire, Sarthe, Savoie, Seine-et-Marne, Var, Vaucluse, Vendée, Vienne, Yonne | 1968, 2006, 2008-2023 | NA | January, February, March, April, May, June, August, September, October, November, December | Winter,  Spring,  Summer,  Autumn | NA | NA | NA | GBIF (2023) |
| *Dermacentor*  *reticulatus* | Pas-de-Calais, Somme, Seine-Maritime, Eure, Calvados, Manche, Orme, Oise, Morbihan, Loire-Atlantique, Mayenne, Sarthe, Eure-et-Loir, Loiret, Yonne, Nièvre, Cher, Indre, Indre-et-Loire, Maine-et-Loire, Vendée, Deux-Sèvres, Vienne, Charente, Charente-Maritime, Haute-Vienne, Creuse, Corrèze, Dordogne, Gironde, Lot-et-Garonne, Essonne, Seine-et-Marne | 2005 | NA | NA | NA | NA | Manual  capture | NA | Chauvet et al. (2005) |
|  | Savoie | 1983-1985 | 12 | May, June, July | Summer | 310, 1538 | Flagging | NA | Chastel et al. (1987) |
|  | Morbihan | 2010-2011 | 12 | April, May, June, July, August, September, October, November, December, January, February, March | Spring,  Summer,  Autumn,  Winter | 70 | Flagging | *Rickettsia raoultii*,  *Babesia* sp. | Michelet et al. (2016) |
|  | Pas-de-Calais, Somme, Seine-Maritime, Eure, Calvados, Manche, Orme, Oise, Morbihan, Loire-Atlantique, Mayenne, Sarthe, Eure-et-Loir, Loiret, Yonne, Nièvre, Cher, Indre, Indre-et-Loire, Maine-et-Loire, Vendée, Deux-Sèvres, Vienne, Charente, Charente-Maritime, Haute-Vienne, Creuse, Corrèze, Dordogne, Gironde, Lot-et-Garonne, Essonne, Seine-et-Marne | 2005 | NA | NA | NA | NA | NA | *Anaplasma marginale*,  *Borrelia burgdorferi* | Agoulon et al. (2012b) |
|  | Pas-de-Calais, Somme, Seine-Maritime, Eure, Calvados, Manche, Orme, Oise, Morbihan, Loire-Atlantique, Mayenne, Sarthe, Eure-et-Loir, Loiret, Yonne, Nièvre, Cher, Indre, Indre-et-Loire, Maine-et-Loire, Vendée, Deux-Sèvres, Vienne, Charente, Charente-Maritime, Haute-Vienne, Creuse, Corrèze, Dordogne, Gironde, Lot-et-Garonne, Essonne, Seine-et-Marne | 2005 | NA | NA | NA | NA | NA | NA | L'Hostis et al. (2007) |
|  | Saône-et-Loire | 1996 | 2 | April, May | Spring | NA | Manual  capture | NA | Levasseur (1997) |
|  | Puy-de-Dôme | 2005 | 5 | May, June, July, August, September | Spring,  Summer | 550-750 | Flagging | NA | Boyard et al. (2008) |
|  | Allier | 2001 | 2 | April, May | Spring | NA | Manual  capture | NA | Personne (2002) |
|  | Jura | 1974 | NA | NA | NA | NA | Flagging | NA | Martinod et al. (1981) |
|  | Saône-et-Loire | 1965-1966 | NA | NA | Winter,  Spring | NA | Manual  capture | NA | Euzéby et al. (1966) |
|  | Saône-et-Loire | 1972-1973 | 24 | February, March, April, May, June, July, August, September, October, November, December, January | Winter,  Spring,  Summer,  Autumn | NA | Manual  capture,  Flagging | NA | Simon et al. (1974) |
|  | Saône-et-Loire, Rhône, Loire, Ardèche | 1984-1986 | 30 | January, February, March, April, May, June, July, August, September, October, November, December | Spring,  Autumn,  Summer,  Winter | NA | Flagging,  Manual  capture | NA | Gilot et al. (1989) |
|  | Saône-et-Loire | NA | 24 | January, February, March, April, May, June, July, August, September, October, November, December | Winter,  Spring,  Summer,  Autumn | NA | Flagging,  Manual  capture | NA | Euzeby et al. (1984) |
|  | Haute-Garonne | 1934-1935 | NA | NA | Winter | NA | NA | *Anaplasma marginale* | Cuillé et al. (1936) |
|  | Bouches-du-Rhône, Gard, Hérault | 2013-2015 | NA | NA | NA | NA | Manual  capture | NA | Dugat et al. (2017) |
|  | Marne, Moselle, Finistère, Vienne, Gironde, Rhône, Alpes-Maritimes, Bouches-du-Rhône, Gard | 2008-2009 | 12 | January, February, March, April, May, June, July, August, September, October, November, December | Winter,  Spring,  Summer,  Autumn | NA | Flagging | NA | Beugnet et al. (2009) |
|  | Côte-d’Or, Saône-et-Loire, Cantal, Isère | 2009 | 0,5 | April, May, September, October | Spring,  Autumn | NA | Flagging | *Anaplasma marginale*,  *Borrelia burgdorferi*,  *Bartonella* sp.,  *Coxiella burnetii*,  *Francisella philomiragia* | Bonnet et al. (2013) |
|  | Puy-de-Dôme, Cantal | 2003-2004, 2006-2007 | 14 | May, April, June, July, August, September, October, November | Spring,  Summer,  Autumn | 300-850 | Flagging | NA | Boyard (2007) |
|  | Puy-de-Dôme | 2003 | 3 | April, May, June | Spring | 300-900 | Flagging | NA | Boyard et al. (2007b) |
|  | Charente, Dordogne, Indre, Indre-et-Loire, Vienne, Creuse, Haute-Vienne, Isère | 1945-2002 | NA | January, February, March, April, May, June, July, August, September, October, November, December | Winter,  Spring,  Summer,  Autumn | NA | Flagging,  Manual  capture | NA | Chauvet (2004) |
|  | Corrèze | 2004 | 5 | March, April, May, June, July | Spring,  Summer | NA | Flagging,  Manual  capture | *Babesia divergens*,  *Anaplasma marginale* | Chauvet (2004) |
|  | Loire | 2001-2002 | 14 | March, April, May, June, July, August, September, October, November, December, January, February | Spring,  Summer,  Winter,  Autumn | 370-1000 | Manual  capture | NA | Devos (2002) |
|  | Ain, Aisne, Allier, Hautes-Alpes, Alpes-Maritimes, Ardèche, Ariège, Aveyron, Bouches-du-Rhône, Cantal, Charente, Cher, Corrèze, Corse, Côte-d'Or, Côtes-d’Armor, Creuse, Dordogne, Doubs, Drôme, Eure, Finistère, Haute-Garonne, Gers, Gironde, Ille-et-Vilaine, Indre, Indre-et-Loire, Isère, Jura, Landes, Loire, Haute-Loire, Loire-Atlantique, Loiret, Lot-et-Garonne, Maine-et-Loire, Marne, Haute-Marne, Meurthe-et-Moselle, Morbihan, Moselle, Nièvre, Nord, Oise, Pas-de-Calais, Puy-de-Dôme, Pyrénées-Atlantiques, Hautes-Pyrénées, Bas-Rhin, Haut-Rhin, Rhône, Saône-et-Loire, Sarthe, Savoie, Haute-Savoie, Paris, Seine-Maritime, Seine-et-Marne, Yvelines, Deux-Sèvres, Somme, Tarn, Tarn-et-Garonne, Var, Vaucluse, Vendée, Vienne, Haute-Vienne, Yonne, Essonne, Hauts-de-Seine, Seine-Saint-Denis, Val-de-Marne, Val-d'Oise | NA | NA | NA | NA | NA | NA | NA | ECDC (2022d) |
|  | Charente, Charente-Maritime, Haute-Vienne, Creuse, Corrèze, Dordogne, Gironde, Landes, Lot, Lot-et-Garonne, Aveyron, Gers, Tarn, Tarn-et-Garonne, Pyrénées-Atlantiques, Hautes-Pyrénées, Ariège, Pyrénées-Orientales, Aude, Hérault, Lozère, Haute-Loire, Gard, Ardèche, Bouches-du-Rhône, Var, Alpes-Maritimes, Vaucluse, Drôme, Isère, Hautes-Alpes, Alpes-de-Haute-Provence | NA | NA | January, February, March, April, May, June, July, August, September, October, November, December | Winter,  Spring,  Summer,  Autumn | NA | NA | NA | François (2008) |
|  | Corrèze | 2005 | 4 | April, May, June, July | Spring,  Summer | NA | Flagging | NA | Frédéric (2005) |
|  | Moselle, Meuse, Vosges | NA | NA | NA | NA | NA | NA | NA | George et al. (2002) |
|  | Drôme, Ardèche, Hautes-Alpes, Alpes-de-Haute-Provence, Vaucluse | 1971-1972 | 12 | October, November, December, January, February, March, April, May, June, July, August, September | Autumn,  Winter,  Spring,  Summer | NA | Flagging,  Manual  capture | NA | Gilot (1985) |
|  | Ain, Ardèche, Drôme, Isère, Loire, Rhône, Savoie, Haute-Savoie, Moselle | 1962-1972 | NA | NA | NA | NA | NA | *Rickettsia* sp. | Gilot et al. (1982) |
|  | Isère, Ain | 1977 | 2 | May, June | Summer | NA | Flagging,  Manual  capture | NA | Gilot et al. (1979) |
|  | All France | NA | NA | NA | Spring,  Summer | NA | Flagging,  Manual  capture | *Borrelia burgdorferi*,  *Francisella tularensis* | Gilot et al. (1998) |
|  | Ain, Haute-Savoie, Savoie, Rhône, Isère, Drôme, Hautes-Alpes, Alpes-de-Haute-Provence, Vaucluse, Bouches-du-Rhône, Var, Alpes-Maritimes, Corse, Rhône | 1970-1973 | NA | January, February, March, April, May, June, July, August, September, October, November, December | Winter,  Spring,  Summer,  Autumn | NA | Flagging,  Manual  capture | NA | Gilot et al. (1975b) |
|  | Aisne, Alpes-de-Haute-Provence, Ariège, Aube, Bas-Rhin, Calvados, Cher, Essonne, Eure-et-Loir, Gers, Gironde, Hérault, Corse, Haute-Garonne, Haute-Savoie, Haut-Rhin, Ille-et-Vilaine, Indre, Indre-et-Loire, Loiret, Loir-et-Cher, Lot-et-Garonne, Lozère, Marne, Nord, Oise, Pas-de-Calais, Puy-de-Dôme, Saône-et-Loire, Sarthe, Seine-et-Marne, Seine-Saint-Denis, Val-d'Oise | 1910, 1967, 2007-2022 | NA | January, February, March, April, May, June, July, August, September, October, November, December | Winter,  Spring,  Summer,  Autumn | NA | NA | NA | INPN (2023) |
[truncated: 65,644 more chars]
